# Combined FGFR and Akt pathway inhibition abrogates growth of FGFR1 overexpressing EGFR-TKI-resistant NSCLC cells

**DOI:** 10.1038/s41698-021-00208-w

**Published:** 2021-07-15

**Authors:** Mikkel G. Terp, Kirstine Jacobsen, Miguel Angel Molina, Niki Karachaliou, Hans C. Beck, Jordi Bertran-Alamillo, Ana Giménez-Capitán, Andrés F. Cardona, Rafael Rosell, Henrik J. Ditzel

**Affiliations:** 1 Department of Cancer and Inflammation Research, Institute of Molecular Medicine, University of Southern Denmark, Odense C, Denmark; 2 Laboratory of Oncology, Pangaea Biotech, Quiron Dexeus University Hospital, Barcelona, Spain; 3 Instituto Oncológico Dr. Rosell, University Hospital Sagrat Cor, Barcelona, Spain; 4 Center for Clinical Proteomics, Odense University Hospital, Odense C, Denmark; 5 Thoracic Oncology Unit, Clinical and Translational Oncology Group, Clinica del Country, Bogotá, Colombia; 6 Instituto Oncológico Dr. Rosell, Quiron-Dexeus University Hospital, Barcelona, Spain; 7 Catalan Institute of Oncology, Hospital Germans Trias i Pujol, Badalona, Spain; 8 Germans Trias i Pujol, Health Sciences Institute and Hospital, Badalona, Spain; 9 Department of Oncology, Odense University Hospital, Odense C, Denmark; 10 Academy of Geriatric Cancer Research (AgeCare), Odense University Hospital, Odense C, Denmark; 11 Present Address: Global Clinical Development, Merck Healthcare KGaA, Darmstadt, Germany

**Keywords:** Cancer therapeutic resistance, Non-small-cell lung cancer

## Abstract

EGFR tyrosine kinase inhibitor (TKI) resistance in non-small cell lung cancer (NSCLC) patients is inevitable. Identification of resistance mechanisms and corresponding targeting strategies can lead to more successful later-line treatment in many patients. Using spectrometry-based proteomics, we identified increased fibroblast growth factor receptor 1 (FGFR1) expression and Akt activation across erlotinib, gefitinib, and osimertinib EGFR-TKI-resistant cell line models. We show that while combined EGFR-TKI and FGFR inhibition showed some efficacy, simultaneous inhibition of FGFR and Akt or PI3K induced superior synergistic growth inhibition of FGFR1-overexpressing EGFR-TKI-resistant NSCLC cells. This effect was confirmed in vivo. Only dual FGFR and Akt inhibition completely blocked the resistance-mediating signaling pathways downstream of Akt. Further, increased FGFR1 expression was associated with significantly lower PFS in EGFR-TKI-treated NSCLC patients, and increased FGFR1 were demonstrated in a few post- vs. pre-EGFR-TKI treatment clinical biopsies. The superior therapeutic benefit of combining FGFR and Akt inhibitors provide the rationale for clinical trials of this strategy.

## Introduction

EGFR tyrosine kinase inhibitors (TKIs), such as the 1st-generation erlotinib and gefitinib, the 2nd-generation afatinib, and the 3rd-generation osimertinib have revolutionized the treatment of non-small cell lung cancer (NSCLC)^1–4^. Unfortunately, many patients develop resistance, which limits the progression-free survival (PFS) to 9–13 months and the overall survival (OS) to 2 years^5^. The mechanisms of acquired resistance are complex and diverse and include both on-target resistance mutations such as the T790M mutation in EGFR^6^ and off-target mechanisms of Akt activation and HGF overexpression^7,8^ and PIK3CA mutations^9^. While many mechanisms of acquired resistance between the 3rd- and 1st-generation EGFR-TKI are shared, unique resistance mechanisms to osimertinib also exist^10^. Acquired resistance to EGFR-TKIs in NSCLC is a complex process and a considerable percentage of resistant cases are still mechanistically unexplained, warranting further investigation^11^.

Recently, fibroblast growth factor receptor 1 (FGFR1) overexpression has also been suggested as a mechanism of resistance towards EGFR-TKIs, and to this end high FGFR1 expression has been shown to be associated with reduced PFS in patients receiving EGFR-TKI treatment^12^.

Fibroblast growth factor receptors (FGFR1-4) are tyrosine kinase receptors (TKIs) associated to cell survival, migration, and angiogenesis^13,14^, and FGFR1 activation by FGF in an autocrine loop can drive tumorigenesis of multiple tumor types, including lung cancer^15–17^. Increased FGFR1 expression is frequent across various lung cancer histologies, including squamous cell carcinomas (SCC) (~25%), and adenocarcinomas (~15%), and FGFR inhibitors (FGFRi) are currently being evaluated in patients with lung SCC and other malignancies^18–22^. It has also been demonstrated that dual EGFR and FGFR targeting is a promising strategy to overcome acquired EGFR-TKI drug resistance in NSCLC^12,23^. Collectively, these findings demonstrate the potential of targeting FGFR to overcome EGFR-TKI resistance and warrant further investigation around this target. Interestingly, the activity of FGFR1 is tightly linked to their regulation of Ras-MAPK, PI3K-Akt, PLCγ-PIP2, and STAT signaling pathways, suggesting the existence of other potential co-targets that could further potentiate the therapeutic effect. In immediate continuation hereof, we recently showed that convergent activation of Akt was associated with EGFR-TKI^8^, thus providing a possible link between FGFR1 overexpression and Akt activation.

Here, in a comprehensive proteomic screen we identified several key members of the FGFR1-Akt pathway, including FGFR1 itself, to be upregulated in association with EGFR-TKI resistance in NSCLC. We confirmed our findings in three independent 1st- or 3rd-generation EGFR-TKI-resistant cell line models as well as in paired NSCLC biopsies obtained before and after progression on EGFR-TKI treatment. We demonstrate that the combination of a FGFRi with an Akt inhibitor (Akti) was necessary to completely inhibit growth in FGFR-overexpressing EGFR-TKI-resistant NSCLC cancer cells both in vitro and in vivo, and furthermore that dual FGFRi and Akti exhibited a significantly stronger synergistic effect compared to targeting FGFR1 and EGFR.

## Results

### Generation of EGFR-TKI-resistant cell lines from the HCC827 NSCLC cell line

**Fig. 1:**
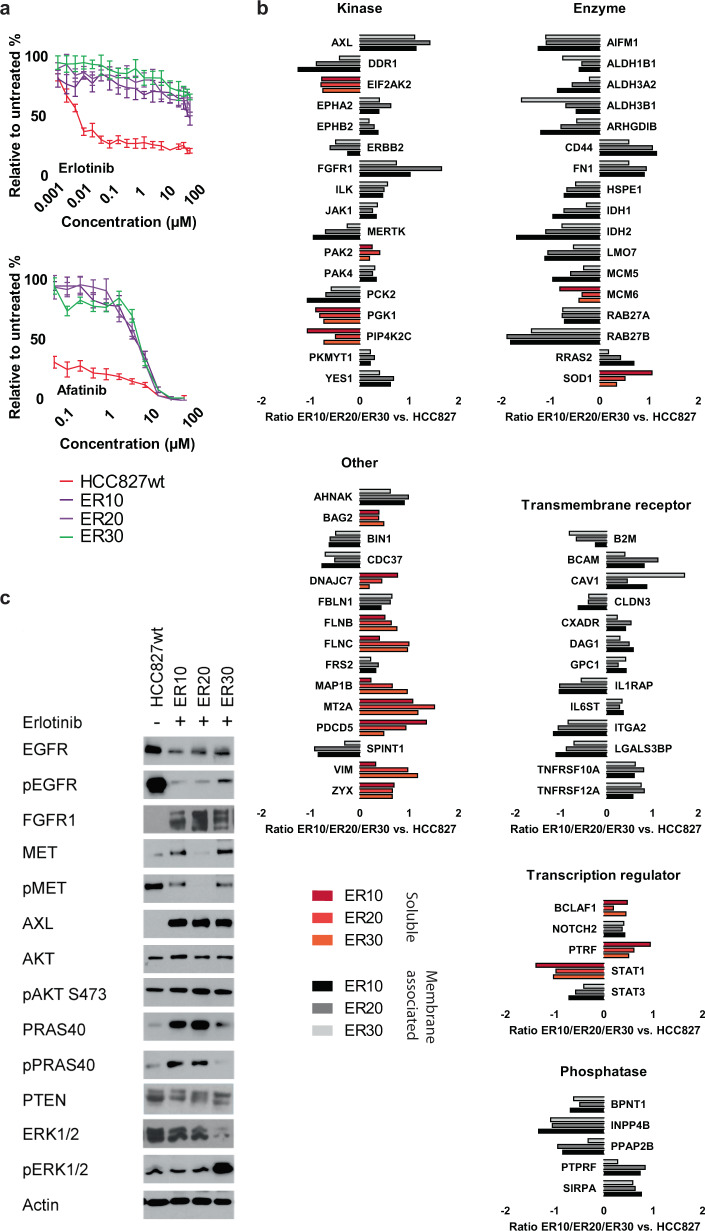
Characterization of erlotinib-resistant NSCLC HCC827 cell lines. **a** Tolerance to erlotinib and afatinib was assessed in the parental HCC827 and the three erlotinib-resistant cell lines, ER10, ER20, and ER30, by crystal violet assay after 72 h incubation. Data are presented as the average of four replicates and shown as mean ± SD. **b** Selected proteins significantly regulated across the three erlotinib-resistant cell lines compared to the parental HCC827, as assessed by mass spectrometry-based proteomics, were classified according to function. The dotted red line indicates 2-fold regulation. **c** Western blot analysis of total and phosphorylated levels of selected signaling proteins. The HCC827 cell lines were exposed to erlotinib (30 µM) for 4 h, the ER10, ER20, and ER30 samples without erlotinib were grown 24 h in the absence of erlotinib, while ER10, ER20, and ER30 samples with erlotinib were grown in media containing 10, 20, and 30 µM erlotinib, respectively, before being harvested for the western blotting in (**c**).

Three isogenic erlotinib-resistant cell lines (ER10, ER20, ER30) were generated from the adenocarcinoma NSCLC cell line, HCC827 (EGFR exon19del) as described in “Methods”. The IC_50_ value for the resistant cell lines to erlotinib were more than 1 × 10^4^-fold higher than the HCC827 wild-type and showed cross-resistance to afatinib (Fig. 1a). Erlotinib-resistant cells grown in the absence of erlotinib for 6 weeks maintained the same level of resistance following re-exposure to erlotinib. The T790M resistance mutation was not detected in any case, nor any EGFR or KRAS mutations other than the original exon19del (Supplementary Table 1). Next-generation sequencing revealed a TP53 p.V218del mutation, and *EGFR* and CDK4 amplification in all four cell lines. In addition, HER2 amplification (6.2 copies) was observed in ER10 and MET amplification (8.3 copies) in ER30. No FGFR amplification was apparent in any of the cell lines.

### Proteome analysis of parental and EGFR-TKI-resistant NSCLC cell lines by mass spectrometry

We performed quantitative proteome analysis to identify proteins exhibiting altered expression in ER10, ER20, and ER30 compared to the HCC827 cell line. A total of 736 proteins were significantly regulated across the panel of resistant cell lines vs. parental HCC827 after Benjamini Hochberg correction with 5% false discovery rate (FDR) (Supplementary Table 2a, b). Using the Ingenuity Pathway Analysis (IPA) platform we categorized the differential regulated proteins (Fig. 1b) based on function and compartment and showed that a very wide variety of cell processes are associated with the emergence of resistance. The receptor tyrosine kinase AXL exhibited the highest fold change in all three erlotinib-resistant cell lines, followed by FGFR1, which was upregulated between 1.7- and 3.6-fold (Fig. 1b). Furthermore, the immediate downstream effector of FGFR1 FRS-2, which propagates the signal to the PI3K-Akt pathway was also significantly upregulated. PRAS40 (AKT1S1), a substrate for Akt, which is a part of one of the downstream signaling pathways of FGFR1, was also upregulated in ER20 (1.7-fold) and ER30 (2.1-fold). ARHGDIB, also known as Rho GDP-dissociation inhibitor 2, was among the downregulated enzymes (Fig. 1b, enzyme), a protein previously demonstrated to inversely correlate with activity of the Akt-mTOR pathway in lung cancer^24^. The identification of several deregulated proteins in the FGFR1-Akt pathway prompted us to investigate whether this pathway was of particular importance for EGFR-TKI resistance in these cell lines.

### Akt and ERK1/2 signaling is maintained in response to erlotinib in EGFR-TKI-resistant NSCLC cell lines

The identified upregulation of FGFR1 in the proteomic analysis was confirmed using western blotting. Importantly, both Akt and phosphorylated Akt were strongly increased in the three resistant cell lines compared to HCC827. Furthermore, the Akt downstream target, PRAS40, displayed increased total and phosphorylated levels in ER10 and ER20 compared to HCC827, suggesting that the Akt pathway is indeed highly active in the resistant cell lines (Fig. 1c). In the ER30 cell lines, we observed an increase in pERK1/2 that might relate to the observed MET amplification.

### Growth inhibition of EGFR-TKI-resistant NSCLC cell lines following treatment with a panel of specific inhibitors

**Fig. 2:**
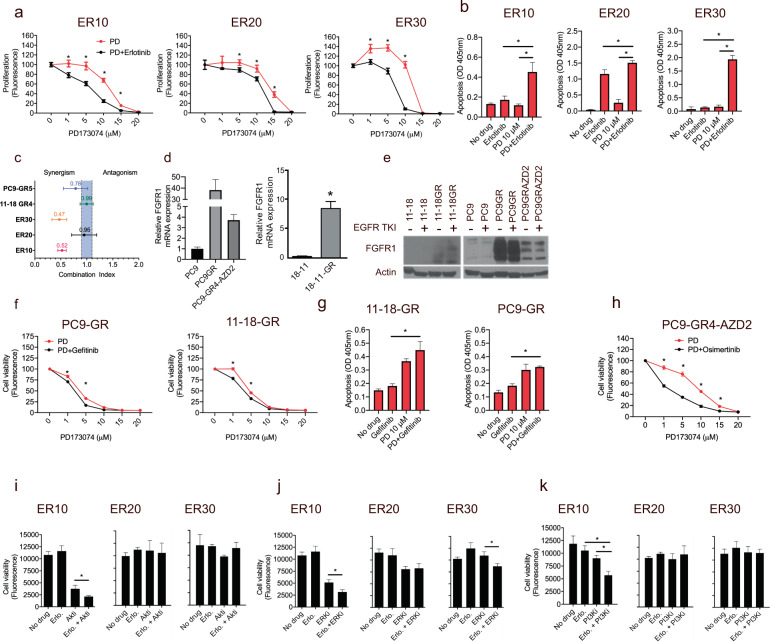
The ability of specific inhibitors to overcome erlotinib, gefitinib, and osimertinib resistance. **a** Viability assay of EGFR-TKI (erlotinib*) combined with the FGFR inhibitor (FGFRi, PD173074 (PD), 0–20 µM) in the three resistant cell lines ER10, ER20, and ER30 analyzed by CellTiterBlue^#^. **b** Apoptosis assay of the same drug combination^##^. **c** Synergisms between FGFRi and erlotinib* or gefitinib in EGFR-TKI-resistant cell lines as determined by a range of combination indexes using CellTiterBlue viability assay data. The blue band indicates the area corresponding to additive interaction. **d** Relative FGFR1 mRNA expression in parental (PC9 and 11-18) and corresponding EGFR-TKI-resistant (PC9-GR, 11-18GR, and PC9-GR4-AZD2) NSCLC cell lines. **e** Western blot assessing protein expression level of FGFR1 in parental and EGFR-TKI-resistant NSCLC cell lines. **f** Viability assay performed with CellTiterBlue of FGFRi (0–20 µM) and EGFR-TKI in the gefitinib-resistant PC9-GR and 11-18GR cell lines^#^. **g** Apoptosis assay of FGFRi and gefitinib in the two gefitinib-resistant cell lines PC9GR and 11-18GR^##^. **h** Viability assay performed with CellTiterBlue of FGFRi (0–20 µM) and osimertinib in the osimertinib-resistant PC9-GR4-AZD2^#^. **i–k** Viability assay performed with CellTiterBlue of EGFR-TKI (erlotinib (Erlo.)) combined with: **i** Akt inhibitor GSK2141795 (Akti 2 µM), **j** ERK1/2 inhibitor FR180204 (ERKi, 35 µM), and **k** PI3K-mTOR inhibitor GSK2126458 (PI3Ki, 0.01 µM)^##^. ^#^For CellTiterBlue assays, data are mean of seven replicates ±SD. Asterisks indicate significant difference in two-tailed *t*-test (*P* < 0.05) for the drug combination-treated cells compared to cells treated with PD173074 alone at the same time point. ^##^For apoptosis assays and single FGFR1 dosage viability assays, data are presented as mean of triplicates ±SD. Asterisks indicate significant differences in ANOVA one-way test (*P* < 0.05) for the drug combination-treated cells compared to cells treated with the same concentration of FGFRi alone. The zero-point of FGFR1 concentration represents untreated cells. *The concentration of erlotinib was 10, 20, or 30 µM in ER10, ER20, and ER30, respectively.

Next, we evaluated the effect of targeting FGFR1, ERK1/2, MET, and Akt with small molecule inhibitors (Supplementary Fig. 1a). Consistent with recent reports^12,23^, the FGFRi was able to overcome erlotinib resistance in ER10, ER20, and ER30 (Fig. 2a, c and Supplementary Fig. 1a), and apoptosis levels also significantly increased following treatment with FGFRi and erlotinib compared to either treatment alone (Fig. 2b). The effect of FGFRi was further tested in three additional FGFR1-overexpressing, EGFR-TKI-resistant cell line models, PC9GR, 11-18GR, and PC9-GR4-AZD2 (Fig. 2d, e). Of note, neither MET nor HER2 was amplified in these cell lines. Intriguingly, the combination of gefitinib and FGFRi only marginally decreased cell viability and proliferation compared to FGFRi alone in PC9GR, 11-18GR (Fig. 2f and Supplementary Fig. 1c) and apoptosis levels only modestly increased following treatment with FGFRi and gefitinib compared to either treatment alone (Fig. 2g). 11-18GR and PC9GR were more sensitive to FGFRi alone compared to the corresponding gefitinib-sensitive cell lines, further pointing to FGFR1 dependence in EGFR-TKI resistance (Supplementary Fig. 1c, d). In the osimertinib-resistant cell line, PC9-GR4-AZD2, we observed a modest but significant effect following treatment with FGFRi and osimertinib compared to FGFRi alone on cell viability and proliferation (Fig. 2h and Supplementary Fig. 1c). Surprisingly, targeting the RAS-MAPK pathway, MET and the Akt and PI3K/mTOR pathways only elicited a minor effect on erlotinib sensitivity (Fig. 2i–k and Supplementary Fig. 1e, f). Collectively, this shows that FGFR1 overexpression plays a central role in EGFR-TKI resistance across multiple EGFR-TKI-resistant NSCLC cell line models, but the growth inhibition by combined FGFRi and EGFR-TKI seems to be inconsistent between different FGFR1-overexpressing EGFR-TKI resistance models, and thus this combination might not be optimal to overcome EGFR-TKI resistance in all FGFR1-expressing NSCLC tumors.

### Co-treatment with a FGFR inhibitor and an Akt inhibitor elicits synergistic growth inhibition of EGFR-TKI-resistant cell lines

**Fig. 3:**
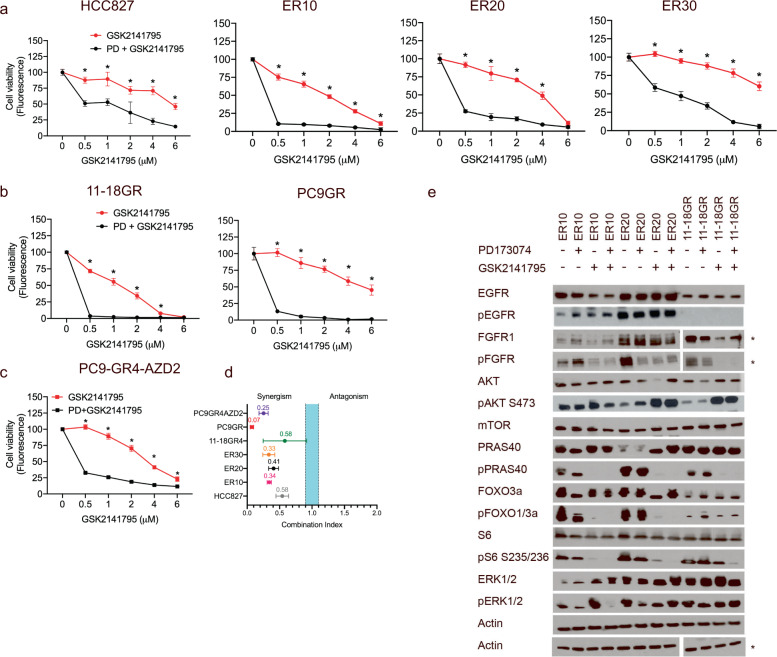
The combination of a FGFR1 inhibitor and an Akt inhibitor elicits significant growth inhibition in FGFR1-overexpressing EGFR-TKI-resistant cell lines and leads to inhibition of the Akt downstream pathway in EGFR-TKI-resistant NSCLC cells. **a** Viability assays performed with CellTiterBlue after 5 days for EGFR-TKI-resistant ER10, ER20, ER30, and parental HCC827 cell lines following exposure to FGFR inhibitor (FGFRi, PD173074 (PD), 10 µM) in combination with a range of different concentration of the Akt inhibitor (Akti, GSK2141795 (GSK)). **b-c** Viability assays performed with CellTiterBlue after 5 days for EGFR-TKI-resistant PC9GR, 11-18GR4, and PC9-GR4-AZD2 cell lines following exposure to PD173074 (10 µM) in combination with a range of different concentrations of GSK2141795. **d** Synergisms between FGFRi and Akti in erlotinib-, gefitinib-, or osimertinib-resistant cell lines as determined by a range of combination indexes from CellTiterBlue viability assays after 5 days of incubation. The blue band indicates the area corresponding to additive interaction, while the area to the left of the blue band indicates synergisms. **e** ER10, ER20, and 11-18GR cell lines were incubated in the absence of an EGFR-TKI with the FGFRi (PD173074, 10 µM) and the Akti (GSK2141795, 2 µM), either alone, in combination, or as vehicle for 4 h before harvest and assessed for protein expression and phosphorylation by western blotting. Asterisk indicates that the cell lines were analyzed separately on two gels, using the lower actin band as loading control. For CellTiterBlue assays: data are presented as mean of seven replicates ±SD. Asterisks indicate significant difference in two-tailed *t*-test (*P* < 0.05) for the drug combination-treated cells (FGFRi and Akti) compared to cells treated with Akti alone at the same concentration.

The correlation between the FGFR1-Akt pathway and EGFR-TKI resistance prompted us to combine the FGFRi with two different Akti’s, GSK2141795 and AZD5363. In contrast to the effect in HCC827, combining Akti and FGFRi very significantly decreased cell viability and proliferation in ER10 and ER20 while the effect in ER30 was less pronounced (Fig. 3a and Supplementary Fig. 2a). Similarly, significant growth inhibition was also observed for 11-18GR, PC9GR, and PC9-GR4-AZD2 when combining the FGFRi and Akti (Fig. 3b, c). Almost invariably, the FGFRi was found to be moderately to strongly synergistic with the Akti’s in the resistant cell lines tested (Fig. 3d). Similar results were observed using the Akti (AZD5363) (Supplementary Fig. 2b–d). Collectively, this demonstrates that targeting FGFR1 and the Akt pathway is a very effective strategy to control proliferation of FGFR1-overexpressing EGFR-TKI-resistant NSCLC cells.

### Co-treatment with a FGFR1 inhibitor and an Akt inhibitor abrogates Akt downstream signaling

The effect of the combined treatment of FGFRi and Akti on protein expression and phosphorylation in ER10, ER20, and 11-18GR cells was investigated by western blotting (Fig. 3e). Importantly, phosphorylation of the Akt downstream targets PRAS40 and was completely abrogated by the FGFRi and AKTi combination in all three EGFR-TKI-resistant cell lines while phosphorylation of the Akt target FOXO and S6 ribosomal protein was completely blocked by the drug combination in ER10 and ER20, but not in 11-18GR. In contrast, AKTi alone was not able to completely block the phosphorylation of FOXO and S6 in all three cell lines. Furthermore, in ER20 and 11-18GR the phosphorylation of PRAS40 was also not completely blocked following Akti alone (Fig. 3e and Supplementary Fig. 2e–g). Taken together, these observations indicate that the combination of FGFRi and Akti is necessary to completely abolish the growth-stimulatory pathways in FGFR1-overexpressing EGFR-TKI-resistant NSCLC cells leading to the observed decreased cell growth and proliferation.

### Combined FGFi and Akti is superior to combined FGFRi and EGFR-TKI in FGFR1-overexpressing EGFR-TKI-resistant cell lines

**Fig. 4:**
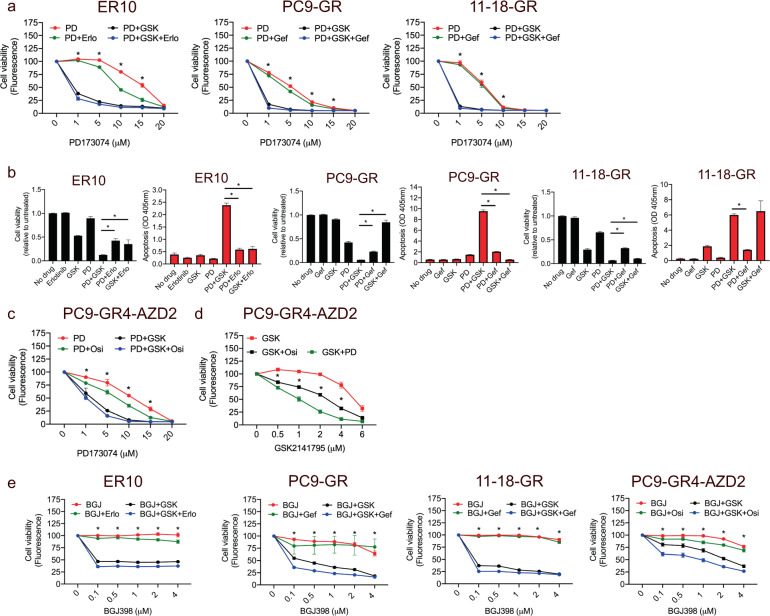
Combined FGFR inhibitor and Akt inhibitor elicits superior growth inhibition compared to combined FGFR inhibitor and EGFR-TKI in FGFR1-overexpressing EGFR-TKI-resistant NSCLC cell lines. **a** Viability assays for erlotinib-resistant ER10, and gefitinib-resistant PC9-GR and 11-18GR cell lines following exposure to the combined FGFR inhibitor (FGFRi, PD173074 (PD, 0–20 µM), and Akt inhibitor (Akti, GSK2141795 (GSK) 2 µM) and/or EGFR-TKI (erlotinib (Erlo, 5 µM) or gefitinib (Gef, 5 µM) performed using CellTiterBlue after 5 days. Data are presented as mean of five replicates ±SD. Asterisks indicate significant differences between cells treated with combined FGFRi and Akti, and those treated with combined FGFRi and EGFR-TKI at the same concentrations using two-tailed *t*-test (*P* < 0.05). **b** CellTiterBlue viability assay and apoptosis assay of ER10, PC9-GR, and 11-18GR following treatment with different combinations of Akti (GSK, 2 µM), EGFR-TKI, and FGFRi (PD, 5 µM) as well as treatment with each inhibitor alone. Data are mean of three replicates ±SD. Asterisks indicate significant differences in ANOVA one-way test (*P* < 0.05). **c** Viability assays for the osimertinib-resistant cell line PC9-GR4-AZD2 following exposure to FGFRi (PD) in a range of different concentrations alone or in combination with Akti (GSK, 2 µM) and/or osimertinib (Osi, 1.25 µM) performed using CellTiterBlue after 5 days. Data are presented as mean of five replicates ±SD. Asterisks indicate significant differences between cells treated with combined FGFRi and Akti, and those treated with combined FGFRi and EGFR-TKI at the same concentrations using two-tailed *t*-test (*P* < 0.05). **d** Viability assays for the PC9-GR4-AZD2 following exposure to Akti (GSK, 0–6 µM) alone or in combination with FGFRi (PD, 10 µM) or osimertinib (Osi, 1.25 µM) performed using CellTiterBlue after 5 days. **e** Viability assays for ER10, PC9GR, 11-18GR, and PC9-GR4-AZD2 following exposure to FGFRi (BGJ398 (BGJ)) in a range of different concentrations alone or in combination with Akti (GSK, 2 µM) and/or EGFR-TKI (Erlo, 5 µM; Gef, 5 µM; Osi, 1.25 µM) performed using CellTiterBlue after 5 days. Data are presented as mean of five replicates ±SD. Asterisks indicate significant differences between cells treated with combined FGFRi and Akti, and those treated with combined FGFRi and EGFR-TKI at the same concentrations using two-tailed *t*-test (*P* < 0.05). Data are presented as mean of five replicates ±SD. Asterisks indicate significant differences between cells treated with combined FGFRi and Akti, and those treated with Akti and EGFR-TKI at the same concentrations using two-tailed *t*-test (*P* < 0.05).

Significantly stronger synergism for co-treatment with a FGFRi and an Akti compared to co-treatment with a FGFRi and an EGFR-TKI was found by comparing the average combination index across ER10, ER20, ER30, PC9GR, and 11-18GR (FGFRi and Akti: 0.34 ± 0.18 (SD) vs. FGFRi and EGFR-TKI: 0.69 ± 0.23 (SD), *P* = 0.019). Thus, our data suggested that, in our cell line models, the combination of FGFRi and Akti was more efficient to overcome EGFR-TKI resistance compared to FGFRi combined with EGFR-TKI. When directly compared, a significantly stronger inhibitory effect and increased apoptosis was observed following combined FGFRi and Aki vs. combined FGFRi and EGFR-TKI in ER10, PC9GR, and 11-18GR (Fig. 4a, b). Furthermore, combined FGFRi and Akti was also superior to combined Akti and EGFR-TKI in ER10 and PC9GR (Fig. 4b). Importantly, combined FGFRi and Akti was superior to both the combinations of FGFRi and EGFR-TKI and Akti and EGFR-TKI in osimertinib-resistant PC9-GR4-AZD2 cells (Fig. 4c, d). We also studied whether co-treatment with a FGFRi and Akti should be combined with an EGFR-TKI. However, the effect of FGFRi, Akti, and EGFR-TKI compared to FGFRi and Akti was very modest, suggesting a minor additional benefit from including an EGFR-TKI (Fig. 4a, c). Finally, we tested another FGFR inhibitor (BGJ398). Similar to FGFRi (PD173074), significantly stronger inhibition was observed following treatment with combined FGFRi (BGJ398) and Aki compared to combined FGFRi (BGJ398) and EGFR-TKI in ER10, PC9GR, and 11-18GR (Fig. 4e).

### Co-treatment with FGFRi and PI3K-mTORi also elicits synergistic growth inhibition of EGFR-TKI-resistant cell lines

**Fig. 5:**
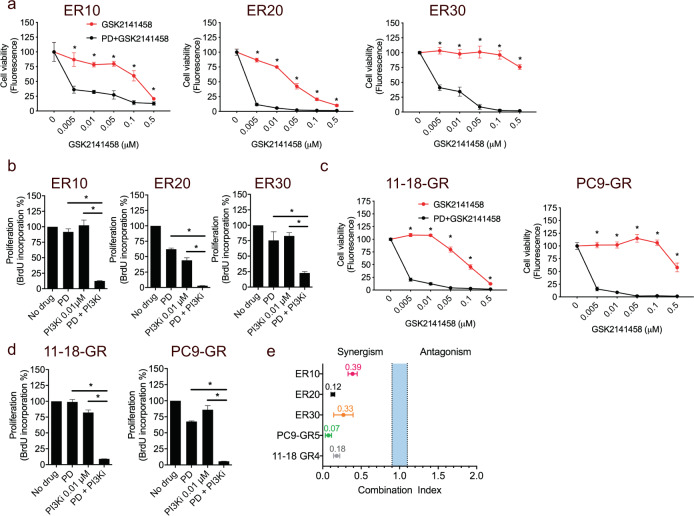
Combined FGFR inhibitor and dual PI3K-mTOR inhibitor elicits significant growth inhibition and anti-proliferative effects in EGFR-TKI-resistant NSCLC cell lines. **a** Viability assays of ER10, ER20, and ER30 performed with CellTiterBlue after 5 days of incubation with PI3K-mTOR inhibitor (GSK2141458) alone or in combination with the FGFR1 inhibitor (FGFRi, PD173074 (PD), 10 μM) **b** BrdU incorporation assays assessing the anti-proliferative effect of FGFRi (PD) combined with GSK2141458 after 5 days of incubation. **c** Viability assays of 11-18-GR and PC9-GR performed with CellTiterBlue after 5 days of incubation with PI3K-mTOR inhibitor (PI3Ki) alone or in combination with FGFRi (PD, 10 μM). **d** BrdU incorporation assays assessing the anti-proliferative effect of FGFRi (PD) combined with GSK2141458 (GSK) after 5 days of incubation. **e** Range of combination indexes in gefitinib-resistant cell lines of CellTiterBlue viability assays after 5 days of incubation. The blue band indicates the area corresponding to additive interaction, while the area to the left of the blue band indicates synergisms. For CellTiterBlue assays: data are presented as mean of seven replicates ±SD. Asterisks indicate significant difference in a two-tailed *t*-test (*P* < 0.05) for the drug combination-treated cells (PD173074 and GSK2141458) compared to cells treated with GSK2141458 alone at the same concentration. For BrdU assays: data are presented as mean of triplicates ±SD. Asterisks indicate significant difference in ANOVA one-way test (*P* < 0.05) for the drug combination-treated cells (PD173074 and GSK2141458) compared to cells treated with GSK2141458 or PD173074 alone at the same concentration.

We also evaluated the combination of FGFR inhibition with the dual PI3K-mTOR inhibitor (PI3K-mTORi, GSK2141458). Compared to FGFRi alone, this combination significantly reduced the cell viability and proliferation of ER10, ER20, ER30, PC9GR, and 11-118GR even at very low concentrations (Fig. 5a, e). The PI3K-mTOR inhibitor also induced a stronger synergism with FGFRi compared to that between FGFRi and an EGFR-TKI when comparing the average combination index across ER10, ER20, ER30, PC9GR, and 11-18GR (FGFRi and PI3Ki: 0.21 ± 0.13 (SD) vs. FGFRi and EGFR-TKI: 0.69 ± 0.20 (SD), *P* = 0.0026). These data further support the therapeutic benefit of combining Akt-pathway inhibitors with the FGFRi.

### Co-treatment with a FGFR1 inhibitor and an Akt inhibitor elicits significant tumor inhibition in two EGFR-TKI-resistant NSCLC xenograft models

**Fig. 6:**
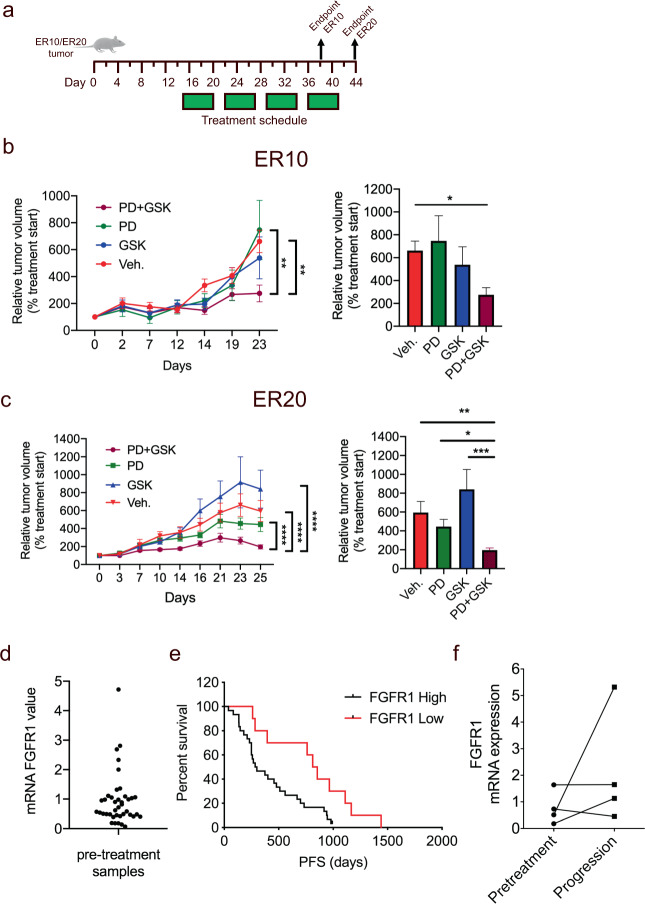
In vivo growth inhibition of FGFR1^high^ EGFR-TKI-resistant NSCLC tumors and assessment of FGFR1 expression in *EGFR*-mutant NSCLC tumors of EGFR-TKI-treated patients. **a** Schema showing the treatment schedule for tumor-bearing mice. Tumor cells were inoculated on day 1 and treatment started on day 15 with FGFRi alone (PD173074, PD, 50 mg/kg), Akti alone (GSK2141795, GSK, 10 mg/kg), combined FGFRi and Akti (PD + GSK), or vehicle (Control) given by daily oral gavage 5 days a week for 4 weeks. **b**, **c** Each mouse harbored two tumors, one on each flank, derived from the EGFR-TKI-resistant NSCLC cell lines ER10 or ER20. Relative tumor volumes, calculated as the ratio between tumor size at randomization and at endpoint, of the individual tumors were used for statistical comparison. Left: The group-specific time slope is referred to as growth rates (GR) and the reported *P*-values are calculated using mixed effects model on log-transformed data and represent the difference in GR of the combined FGFRi and Akti compared to control or either treatment alone. Right: Results are shown as mean ± SEM. Asterisks indicate significant differences at endpoint using non-parametric Kruskal Wallis test with Dunn’s multiple comparison (^*^P < 0.05, ^**^P < 0.01, ^***^P < 0.001, ^****^P < 0.0001). **d** FGFR1 mRNA expression levels in clinical *EGFR*-mutant NSCLC tumors of a cohort of 40 patients treated with first-line EGFR-TKIs using qPCR. **e** Kaplan-Meier curves illustrate correlations between FGFR1 mRNA expression progression-free survival (PFS) for the 40 patients treated with first-line EGFR-TKIs. **f** FGFR1 mRNA expression level in paired biopsies of *EGFR*-mutant NSCLC tumors taken prior to treatment with first-line EGFR-TKIs and upon progression on the first-line EGFR-TKI treatment using qPCR.

Next, we evaluated the in vivo anti-tumor activity of the combination of FGFRi (PD173074) and Akti (GSK2141795) in the ER10 model as described in “Methods” (Fig. 6). Using mixed linear effect models, we showed that tumor growth rates (GR) were significantly slower in mice treated with combined FGFRi and Akti (GR = 0.025) than vehicle (GR = 0.061, CI (0.044–0.077), *P* < 0.005) and FGFRi alone (GR = 0.066, CI (0.042–0.089) *P* < 0.009), while no difference was observed when compared to AKTi alone (GR = 0.041, CI (0.023–0.060), *P* < 0.237). At endpoint, a significant difference in the relative tumor volume between those treated with the drug combination (*n* = 10 tumors) compared to vehicle (*n* = 14 tumors) (*P* = 0.044) was observed (Fig. 6b). In contrast, the difference in the relative tumor volume between those treated with either drug alone (Akti alone; *n* = 11 and FGFRi alone; *n* = 7 tumors) and the vehicle (*n* = 14 tumors) was not significant. The average tumor sizes at start-point and endpoint in the groups treated with combined FGFRi and Akti, FGFRi alone, Akti alone, and vehicle groups were 8.4 mm^3^ and 18.6 mm^3^, 9.7 mm^3^ and 59.9 mm^3^, 7.3 mm^3^ and 28.5 mm^3^, and 7.0 mm^3^ and 39.5 mm^3^, respectively. To evaluate whether Akt signaling was downregulated following the different treatments, tumors from the different groups were stained for phosphorylated PRAS40 (pPRAS40), which is downstream of Akt. As expected, the levels of pPRAS40 were lower in the tumors treated with ether FGFRi and Akti alone compared to vehicle, while the lowest level of pPRAS40 observed in the tumors treated with combined FGFRi and Akti (Supplementary Fig. 1g). In a similar setup we used the ER20 model to test the combination of FGFRi and Akti (Fig. 6c, left). Using mixed linear effect models, we showed that tumor growth rates (GR) were significantly slower in mice treated with combined FGFRi and Akti (GR = 0.034) than vehicle (GR = 0.068, CI (0.059–0.077), *P* < 0.0001), Akti alone (GR = 0.080, CI (0.071–0.089) *P* < 0.0001), and FGFRi alone (GR = 0.055, CI (0.071–0.089), *P* < 0.0001), respectively. Comparing tumor sizes at endpoint also showed a significant difference in relative tumor volume between tumors treated with the FGFRi and Akti combination (*n* = 14 tumors) compared to FGFRi alone (*n* = 20 tumors, *P* = 0.033), Akti alone (*n* = 12 tumors, *P* = 0.0006), and vehicle (*n* = 13 tumors, *P* = 0.0067) (Fig. 6c, right). The average tumor sizes at start-point and endpoint in the FGFRi and Akti, FGFRi alone, Akti alone, and vehicle groups were 51.1 mm^3^ and 86.6 mm^3^, 45.7 mm^3^ and 141.4 mm^3^, 23.4 mm^3^ and 118.0 mm^3^, and 44.6 mm^3^ and 189.3 mm^3^, respectively.

### Clinical evaluation of FGFR1 mRNA expression levels in EGFR-mutant NSCLC tumors of EGFR-TKI-treated patients

We first determined baseline FGFR1 mRNA expression by Q-PCR in 40 EGFR-mutant NSCLC tumors (Supplementary Table 3) treated with first-line EGFR-TKIs and found widely different baseline levels of FGFR1 mRNA (Fig. 6d). The patients were then stratified into FGFR1^low^ (the lower quartile of the ΔΔCt FGFR1 values) and FGFR1^high^ (the three higher quartiles of the ΔΔCt FGFR1 values) expression groups. PFS was significantly longer in FGFR1^low^ patients (813 days, 95% CI, 261–1110; *n* = 10) compared with FGFR1^high^ patients (284, 95% CI, 2495–511 days; *n* = 30) (*P* = 0.011) (Fig. 6e). Univariate and multivariate Cox’s proportional hazards regression analysis showed that FGFR1 expression and stage were prognostic factors for PFS and significantly associated with PFS (Supplementary Table 4). Next, we analyzed the level of pAkt by immunohistochemistry and further stratified the patients into FGFR1^high^/pAkt^high^ (*n* = 3) and FGFR^high/low^/pAkt^low^ subgroups (*n* = 37). PFS was significantly shorter in FGFR1^high^/pAkt^high^ patients than in FGFR^high/low^/pAkt^low^ patients (*P* < 0.0001) (Supplementary Fig. 2h) and supports the presented evidence of combined FGFR1 overexpression and Akt activation in EGFR-TKI resistance. In a random subgroup of the 40 EGFR-mutant NSCLC tumors, including both FGFR1^high^ and FGFR1^low^ expressing tumors, we analyzed the co-occurrence of other reported EGFR-TKI resistance mechanisms such as T790M, MET amp, HER2 amp, and AXL expression (Supplementary Table 5). However, none of these resistance mechanisms were found to be associated with FGFR1 expression.

To evaluate whether FGFR1 upregulation occurs in clinical NSCLC tumors that progressed on EGFR-TKI treatment, we examined the FGFR1 mRNA expression in paired tumor samples from EGFR-mutant NSCLC patients at baseline and after progression to EGFR-TKI treatment (Fig. 6f). Although sample pairs from only four patients could be obtained, two of these exhibited increased FGFR1 mRNA expression in the post-EGFR-TKI-treatment tumor sample compared to the corresponding pre-treatment tumor sample, suggesting that FGFR1 upregulation occurs in some clinical tumor samples with acquired resistance to EGFR-TKI treatment.

## Discussion

NSCLC patients who initially respond to EGFR-TKI therapy ultimately develop resistance by many different mechanisms. Here, we showed that upregulation of proteins in the FGFR1-Akt pathway is associated with acquired EGFR-TKI resistance in NSCLC using a proteomic approach. Previously, we have shown that Akt activation is a targetable convergent feature of acquired EGFR-TKI resistance^8^. In the present study, we showed that while the effect of Akt inhibition seems to be only modest in cancer cells overexpressing FGFR1, targeting FGFR1 in combination with Akti or PI3K-mTORi resulted in a strong and synergistic growth inhibition and induction of apoptosis in FGFR1-overexpressing EGFR-TKI-resistant NSCLC cells. FGFR1 has previously been associated with erlotinib resistance in NSCLC^15,17^ and combined FGFRi and EGFR-TKI has been shown to be therapeutically beneficial in EGFR-TKI-resistant NSCLC models^12,23,25^. Consistent with these reports^12^, we showed that FGFR1 overexpression was associated with erlotinib-, gefitinib-, and osimertinib-resistant NSCLC cells. However, we showed in this study that the beneficial effect of targeting FGFR1 in combination with erlotinib, gefitinib, or osimertinib was not consistently effective across the FGFR1-overexpressing EGFR-TKI-resistant models, suggesting that this combination strategy might not be optimal to overcome EGFR-TKI resistance in all FGFR1-overexpressing tumors. Instead, we found that combined FGFRi and Akti or PI3K-mTORi exhibited much stronger and superior inhibition of cancer cell growth compared to combined FGFRi and EGFR-TKI across all the erlotinib, gefitinib-, and osimertinib-resistant models. Interestingly, no additional effect of adding an EGFR-TKI to the FGFRi and Akti combination was observed. This suggests that ERK signaling is no longer necessary when combining FGFRi and Akti. Despite inhibition of the Akt pathway following Akti alone, it does not inhibit proliferation of the tumor cells, which might be due to residual Akt activity. However, when Akti and FGFRi are combined, the Akt pathway is completely blocked, and we demonstrate maximal suppression of cell viability. Collectively, this indicates that when the FGFR1 is overexpressed, the ERK pathway becomes dispensable, and the tumor cells rely solely on the FGFR1/Akt pathway. We also observed gene amplification of HER2 and MET in ER10 and ER30, both of which have been shown to be involved in EGFR-TKI resistance^26,27^. To this end, combined Akt and FGFR inhibition could be superior because it blocks multiple RTKs. However, the absence of HER2 and MET amplification in PC9GR, PC9GR4-AZD2 and 11-18GR, and the low effect of combined METi (foretinib) and EGFR-TKI suggest that the benefit of co-targeting Akt is not due to suppression of downstream signaling from MET and HER2 and points to a specific relationship between FGFR1 and Akt in our models.

We also demonstrated the superior effect of combined FGFR and Akt inhibition compared to single-drug treatment in ER20 tumor-bearing xenograft mice. Previous studies reporting synergistic growth inhibition when combining a FGFR and mTOR or Akt inhibitor in FGFR1-dependent lung, head-and-neck, and hepatocellular carcinoma cell lines^28,29^ support our findings; however, none of these studies addressed the effectiveness of the drug combinations in relation to EGFR-TKI resistance in NSCLC. Collectively, these data clearly demonstrate the strong potential of co-targeting FGFR1 and the PI3K/Akt/mTOR pathway in FGFR1-overexpressing EGFR-TKI-resistant tumors. Mechanistically, we showed that the combined FGFRi and Akti completely prevented phosphorylation of the Akt downstream proteins PRAS40, FOXO, and S6 ribosomal protein, which is supported by a study showing that combining mTORi and FGFRi elicited synergistic growth inhibition and pS6 levels (S235/236)^29^. This further supports the tight relationship between FGFR1 and the Akt pathway in resistance to EGFR-TKI and the need for dual targeting of Akt and FGFR1.

Despite a higher FGFR1 level in brain metastases originating from NSCLC adenocarcinomas (15.3%)^30^, FGFR1 amplification is somewhat lower in adenocarcinomas compared to squamous NSCLC (2.2-4.1%)^31,32^. Interestingly, none of the NSCLC adenocarcinoma patients in these studies were treated with EGFR-TKIs. To this end, we showed that approximately 12% of EGFR-mutant NSCLC tumors exhibited high FGFR1 expression, suggesting that EGFR-TKI are driving FGFR1 in patients with primary adenomacarcinoma NSCLC. Furthermore, our findings support two other studies that found a significant correlation between FGFR1 expression and PFS and/or OS in EGFR-TKI-naive clinical specimens^12,33^. However, multivariate analyses to investigate whether the prognostic value of FGFR1 expression was affected by underlying clinicopathological covariates were not performed in these two studies. While initially FGFR1 copy number was used to stratify SCC patients into FGFRi clinical trials, later, FGFR1 at mRNA or protein levels was found to be a better biomarker^21,22^, supporting the predictive value of FGFR1 expression for clinical outcome. Thus, our study provides additional valuable information of the prognostic value of FGFR1 expression in NSCLC progression.

In conclusion, we identified the FGFR1-Akt pathway to be an important resistance mechanism to EGFR-TKI in NSCLC. We showed that in FGFR1-overexpressing EGFR-TKI-resistant cells, dual FGFR and Akt inhibition was necessary for efficient growth inhibition, and furthermore that this combination was superior to combined FGFRi and EGFR-TKI treatment across multiple EGFR-TKI resistance models. Moreover, we showed that increased levels of FGFR1 in EGFR-TKI-naive EGFR-mutant NSCLC clinical specimens predicted worse outcome on EGFR-TKI treatment. Collectively, our preclinical and clinical data provide a strong rationale for clinical testing of dual targeting of FGFR and Akt in NSCLC patients with FGFR1-overexpressing EGFR-mutant tumors resistant to EGFR-TKI.

## Methods

### Cell lines, cell culture, inhibitors, and cell viability assays

All tissue culture materials were obtained from Gibco Life Technologies. The parental HCC827 cells were purchased from the ATCC. Parental PC9 cells were kindly provided by F. Hoffman-La Roche Ltd with the authorization of Dr. Mayumi Ono (Kyushu University, Fukuoka, Japan). Parental 11-18 cells were kindly provided by Dr. Mayumi Ono. Cells were cultured in RPMI-1640 Glutamax medium supplemented with 10% fetal bovine serum (FBS) and 50 µg/mL penicillin–streptomycin, and maintained in a humidified atmosphere with 5% CO_2_ at 37 °C. The isogenic erlotinib-resistant clones ER10, ER20, and ER30 were generated from the sensitive HCC827 cell line (CRL-2868, ATCC) by growing the cells in increasing concentrations of erlotinib over the course of 7 months. ER10, ER20, and ER30 were routinely grown in the presence of 10, 20, and 30 µM erlotinib, respectively. The gefitinib-resistant adenocarcinoma NSCLC cell line PC9GR was derived from PC9 and harbors an exon19del in *EGFR*. The gefitinib-resistant cell line 11-18GR was generated from the adenocarcinoma NSCLC cell line 11-18, which carries the sensitizing L858R mutation in EGFR and a NRAS mutation (Q61L). The osimertinib-resistant cell line PC9-GR4-AZD2 is derived from PC9GR. Neither PC9GR, 11-18GR, nor PC9-GR4-AZD2 acquired the T790M mutation. For more information see^8,34^. The EGFR-TKIs erlotinib and osimertinib (AZD9291), the FGFRi (PD173074), the FGFRi (BGJ398), the Akti capivasertib (AZD5363), the c-METi foretinib (GSK1363089), the ERK1/2i FR180204, the MEKi GSK2110212, and the PI3K-mTORi omipalisib (GSK2126458) were purchased from Selleck Chemicals and the Akti uprosertib (GSK2141795) was from Medchemexpress LLC. All drugs were dissolved in DMSO, aliquoted, and kept at −20 °C. PD173074 is a selective and potent ATP-competitive inhibitor of FGFR3 and FGFR1 (IC_50_ = 5 and 21.5 nM, respectively), and also inhibits FGFR2 and FGFR4^35–37^.

### STR analysis

To authenticate cell line identity, short tandem repeat (STR) analysis was performed using the Cell ID™ System (Promega, #G9500) as described by the manufacturer. In brief, ten specific loci of the human genome were PCR amplified and analyzed by capillary electrophoresis. We found that ER10, ER20, and ER30 had the same allelic sizes at all ten loci as the parental HCC827 clone. We also found the allelic loci sizes to be identical to those published by ATCC.

### Cell harvest for mass spectrometry

Cells were harvested at 80–90% confluency. Cells were washed once in ice-cold Tris-buffered saline (TBS, Sigma-Aldrich) including complete Mini EDTA-free protease inhibitors (Roche, #1183670001), carefully scraped from the cell culture flask to avoid damaging extracellular membrane proteins and spun at 250*g* for 2 min, and then the cells were washed twice in ice-cold TBS with protease inhibitor. Finally, cells were lysed in ice-cold 0.1 M Na_2_CO_3_ with protease inhibitor, pH 11. The lysate was adjusted to 1 mM MgCl_2_ and 5 µL benzonase (Sigma-Aldrich, #8263) was added; samples were then left on ice for 15 min to degrade RNA and DNA.

To increase the number of proteins identified, the samples were divided into soluble and membrane-associated proteins. Lysates were homogenized using a Branson sonifier 250, 2 × 30 s, output 10, output control 2.5, and subsequently ultra-centrifuged at 100,000*g* for 45 min at 4 °C in a Sorvall RC M150 GX centrifuge to separate the soluble proteins (supernatant) from membrane proteins (pellet). After removal of the supernatant, pellets were washed with 0.5 M triethylammonium bicarbonate (TEAB) (Thermo Fischer Scientific (#90114) followed by 0.05 M TEAB to remove soluble protein contamination. Of the total of 4049 proteins identified, 2006 were found in the soluble fraction and 3025 in the membrane-associated fraction (24.3% were present in both fractions).

### Protein purification and digestion

The supernatant proteins were precipitated by adding five volumes of ice-cold acetone, vortexed, and stored ON at −20 °C followed by centrifugation at 6000*g* for 15 min. After removal of acetone, the pellets containing soluble proteins were re-dissolved in 8 M urea and incubated ON to fully dissolve proteins. Protein concentrations were determined by the Bio-Rad Protein Assay (Bio-Rad #500-0113, USA) before proteins were reduced by 20 mM DTT at 56 °C for 45 min and subsequently alkylated by 40 mM iodoacetamide for 45 min in the dark. Samples were then diluted eight times with 0.05 M TEAB and digested with 1 µg trypsin/50 µg protein (Promega, #V5280) at 37 °C ON. Samples were acidified to 0.1% trifluoroacetic acid (TFA) and centrifuged at 15,000*g* for 10 min to pellet insoluble materials such as lipids. The membrane proteins were re-dissolved in 8 M urea, reduced, and alkylated as stated above before addition of 0.5 µL Sialidase A (Europa Bioproducts) and 1 µL PNGase F (Sigma-Aldrich, #P7367) at 37 °C ON to remove extracellular glycan structures. Samples were then digested with trypsin as described above.

### iTRAQ labeling

Peptide mixtures were desalted using in-house-packed stage tip columns composed of two C18 membrane disks (Empore 3 M, Bellefonte, USA) and porous R2/R3 reversed-phase resins (Thermo Fischer Scientific). In brief, samples were acidified to pH ~ 2 before peptides were applied to 0.1% TFA pre-equilibrated columns, washed with 0.1% TFA, and eluted using 70% ACN, 0.1% TFA. The eluted peptides were vacuum centrifuge dried before being reconstituted in 0.05 M TEAB prior to amino acid analysis (AAA) to measure peptide concentration. AAA was performed by lyophilizing a small aliquot of peptide sample and adding 200 µL hydrolysis buffer (6 M HCl, 0.1% phenol, 0.1% thioglycolic acid), filling with argon and subsequently evaporating under vacuum. Samples were then incubated at 110 °C ON. After hydrolysis, the amino acids were analyzed on a Biochrom30 amino acid composition analyzer (Cambridge, UK) as described in^38^, and 20 µg of peptides from each sample were then transferred to new Eppendorf tubes, vacuum dried, and re-dissolved in 0.5 M TEAB prior to iTRAQ labeling (AB Sciex), as described by the manufacturer. After iTRAQ labeling and pooling, samples were vacuum centrifuge dried, re-dissolved in 0.1% TFA, and desalted on in-house-packed R2/R3 stage tip columns, as previously described.

### HILIC fractionation

Samples were fractionated using hydrophilic interaction chromatography (HILIC). Briefly, samples were re-dissolved in 90% ACN/0.1% TFA, and 15 µL aliquots corresponding to approximately 25 µg peptides were injected onto an in-house-packed TSKgel Amide-80 HILIC 300 μm × 300 mm capillary HPLC column and fractionated into 22 fractions by a Dionex UltiMate 3000 nano high-performance liquid chromatography. The fractions were automatically collected in a microwell plate at 1 min intervals after UV detection at 210 nm, and the fractions were dried by vacuum centrifugation and re-dissolved in 10 µL 0.1% TFA and analyzed by nanoLC–MS/MS, as described below.

### NanoLC-MS/MS

NanoLC-MS/MS analysis was conducted on a Q-Exactive mass spectrometer (Thermo Fisher Scientific, Bremen, Germany) equipped with a nanoHPLC interface (Dionex UltiMate 3000 nano HPLC). The samples (5 µL) were loaded onto a customized fused capillary pre-column (2 cm length, 360 µm OD, 75 µm ID packed with ReproSil Pur C18 3 µm resin) (Dr Maish, GmbH, #rs13.9e) with a flow of 5 µL/min for 7 min. Trapped peptides were separated on a customized fused capillary column (20 cm length, 360 µm OD,100 µm ID, packed with ReproSil Pur C13 3 µm resin) using a linear gradient from 95% solution A (0.1% formic acid) to 30% solution B (100% acetonitrile in 0.1% formic acid) over 51 min, followed by 5 min at 90% solution B and 5 min at 98% solution A at a flow rate of 250 nL per minute. Ammonia vapor was used to decrease the charge states of the iTRAQ-labeled peptides, thereby increasing the number of protein identifications^39^. Mass spectra were acquired in positive-ion mode applying automatic data-dependent switch between an Orbitrap survey MS scan in the mass range of 400–1200 *m*/*z* followed by high-energy collisional dissociation fragmentation (HCD) and Orbitrap detection of the 12 most intense ions observed in the MS scan. Target value in the Orbitrap for MS scan was 1,000,000 ions at a resolution of 70,000 at *m*/*z* 200 and 50,000 ions at a resolution of 17,500 at *m*/*z* 200 for MS/MS scans. Fragmentation in the HCD cell was performed at normalized collision energy of peptides and 31 eV for iTRAQ-labeled peptides. Ion selection threshold was set to 33,000 counts. Selected sequenced ions were dynamically excluded for 60 s. Each of the soluble fractions was analyzed in biological triplicates, while the membrane-associated fractions were analyzed in biological duplicates.

### Analysis of proteomic data

All Q-Exactive raw data files were processed and quantified using Proteome Discoverer version 1.4.0.288 (Thermo Scientific). The SEQUEST search engine and Mascot search engine (v. 2.2.3) integrated with Proteome Discoverer were used to search the data with the following criteria: protein database used was Uniprot/Swissprot (downloaded on 7 November 2012; 452,768 entries) and restricted to humans. Fixed search parameters included trypsin, one missed cleavage allowed, carbamidomethylation at cysteines, and iTRAQ labeling at lysine and N-terminal amines, while methionine oxidation and deamidation were set as dynamic. Precursor mass tolerance was set to 8 ppm and fragment mass tolerance was set to 0.05 Da. Peptide data were extracted using Mascot significance threshold 0.05 and minimum peptide length 6 amino acids. Minimum two peptides were used for protein identification, and minimum two unique peptides were used for protein quantitation. FDR was calculated using a decoy database search and only high-confidence peptide identifications (FDR < 1%) were included. The mass spectrometry proteomics data have been deposited to the ProteomeXchange Consortium via the PRIDE partner repository with the dataset identifier PXD011803.

### Statistical analysis of proteomic data

For the proteomic analysis, only statistically significant regulated proteins were considered for bioinformatics analysis. These proteins were identified by calculations based on the log2-transformed ratio and the intensity of the iTRAQ reporter ions from all quantified peptides by using the Perseus software (version 1.304)^40^. Statistical significance was defined as *P* < 0.05 after correction for multiple tests by Benjamini-Hochberg.

### IC_50_ determination

In all, 2500 cells/well were seeded in 96-well plates and left for 6–8 h to settle before adding the appropriate drug, and were then incubated at 37 °C and 5% CO_2_ for 72 h and quantified by crystal violet assay.

### DNA purification, EGFR/KRAS mutation testing, and next-generation sequencing

DNA was extracted from the cells using the QIAamp DNA Mini Kit (Qiagen, #51304), and EGFR and KRAS mutation status examined using the TheraScreen EGFR RGQ PCR kit (Qiagen #87411) and the TheraScreen KRAS RGQ PCR kits (Qiagen #870001) as described by the manufacturer. Next-generation sequencing of DNA purified from the cell lines was performed by the GeneReader Platform (Qiagen). Purified DNA (16.75 μL, ~10–70 ng) was used as a template to generate libraries for sequencing with the GeneRead^TM^ QIAact Lung DNA UMI Panel, according to manufacturer’s instructions. Libraries were quantified using a QIAxcel Advanced System and Qubit dsDNA HS Assay kit, diluted to 100 pg/μL, and pooled in batches of six (liquid biopsies). Clonal amplification was performed on 625 pg of pooled libraries by the GeneRead Clonal Amp Q Kit using the GeneRead QIAcube and an automated protocol. Following bead enrichment, pooled libraries were sequenced using the GeneRead UMI Advanced Sequencing Q kit in a GeneReader instrument. QIAGEN Clinical Insight Analyze software was employed for the secondary analysis of FASTQ reads, align the read data to the hg19 reference genome sequence, call sequence variants, and generate a report for visualization of the sequencing results. Variants were imported into the QIAGEN Clinical Insight Interpret web interface for data interpretation and generation of final custom report.

### Western blotting

When EGFR-TKI-resistant cells were evaluated in the absence of EGFR-TKI, the drug was removed 24 h prior to analysis, and when parental cell lines were evaluated in the presence of EGFR-TKI- or EGFR-TKI-resistant cells exposed to drugs other than an EGFR-TKI, the drug was added 4 h prior to analysis. Cells were washed in ice-cold TBS, spun down, and lysed in RIPA buffer (10 mM Tris HCl pH 8, 5 mM Na_2_EDTA pH 8, 1% NP-40, 0.5% sodium dioxycholate, 0.1% SDS), both containing protease and complete Mini PhosphoSTOP phosphatase inhibitors (Roche, #116995001). Protein concentrations were determined by Pierce BCA Protein Assay (Thermo Scientific, #23225) according to the manufacturer’s protocol. In all, 5–40 μg protein was resolved on 4–12% RunBlue SDS-PAGE gels (Expedeon, #NXG00812), transferred onto PVDF membrane (GE Healthcare Life Sciences), blocked and then incubated with primary antibodies anti-EGFR (#HPA001200, dilution 1:1000), pEGFR (#3777, dilution 1:1000), FGFR1 (#9740, dilution 1:1000), pFGFR1 (#3476, dilution 1:1000), anti-MET (#3148, dilution 1:1000), anti-pMET (#3077, dilution 1:1000), anti-AXL (#AB154,, dilution 1:1000), anti-Akt (#4685, dilution 1:1000), anti-pAkt (#4060, dilution 1:2000), anti-PRAS40 (#2691, dilution 1:1000), anti-pPRAS40 (#2997, dilution 1:1000), anti-PTEN (#9556, dilution 1:1000), anti-EKR1/2 (#9102, dilution 1:2000), anti-pERK1/2 (#4370, dilution 1:1000), anti-mTOR (#2983, dilution 1:1000), anti-FOXO3a (#2497, dilution 1:500), anti-pFOXO3a (#2599, dilution 1:1000), anti-S6 (#2317, dilution 1:1000), anti-pS6 (#4858, dilution 1:2000), and anti-β-actin (#ab6276, dilution 1:100,000) ON at 4 °C. All antibodies were purchased from Cell Signaling Technology except the anti-EGFR (Sigma Aldrich), anti-AXL (R&D Systems), and anti- β-actin (Abcam). Following incubation with primary antibodies, the membranes were incubated with goat anti-rabbit (Dako P0448), goat anti-mouse (Dako, P0447), or donkey anti-goat (Santa Cruz, F1515) HRP-conjugated secondary antibodies in 1:5000 dilution for 1 h at room temperature. The immunoreactive bands were visualized by Amersham ECL Prime Western Blotting Detecting Reagent (GE Healthcare Life Sciences, Buckinghamshire, UK) and exposed to CL-Xposure film (Thermo Fischer Scientific, #34089). All blots were derived from the same experiment and were processed in parallel.

### Quantitative real-time PCR

Total RNA was purified using Isol-Lysis Reagent, TRIzol (Life Technologies). cDNA synthesis was performed using RevertAid Premium Reverse Transcriptase Kit (Fermentas). The relative quantification of gene expression was performed using SYBR Green PCR Mastermix (Applied Biosystems) according to manufacturer’s instructions. FGFR1 primers (QT00102837) and PUM1 (QT00029421) (reference gene) were purchased from Qiagen. The relative expression levels were calculated using the comparative threshold method^41^.

### Analysis of FGFR1 mRNA levels

RNA was isolated from formalin-fixed paraffin-embedded (FFPE) tissue specimens as previously described^42^. Quantification of gene expression was performed using the ABI Prism 7900HT Sequence Detection System (Applied Biosystems) and calculated according to the comparative Ct method. The primer and probe sets for FGFR1 were designed using Primer Express 3.0 Software (Applied Biosystems) according to their Ref Seq: (http://www.ncbi.nlm.nih.gov/LocusLink). mRNA from the parental cell line PC9, and the two FGFR1-high cell lines PC9GR and PC9GR4-AZD2 were included as controls.

### Cell proliferation, viability, and apoptosis assays

In all, 2500 cells/well in 96-well plates or 10,000 cells/well in 24-well plates were seeded and left to attach for 6 h before drugs or vehicle were added, and then incubated at 37 °C. Cell proliferation and viability was quantified by crystal violet staining, by CellTiterBlue (Promega, #G8080) or by BrdU incorporation using the BrdU Cell Proliferation Assay Kit (Cell Signaling Technology, #6813), the two latter according to the manufacturer’s instructions. Crystal violet assay was performed by incubation with 0.5% crystal violet in 25% V/V Methanol (Sigma Aldrich, # V5265) 25% V/V Methanol for 5 min. Cells were washed twice in H_2_O and the stained cells were then dissolved in citrate buffer (0.1 mM sodium citrate in 50% EtOH) while shaking for 30 min at RT and the absorbance was measured at 570 nm. Apoptosis was assessed using the Cell Death Detection ELISA Plus kit (Roché, #11774425001), according to the manufacturer’s instructions.

### Immunohistochemistry

Immunohistochemistry was performed on 5 μm sections using an automated immunostainer (Ventana BenchMark ULTRA, Ventana Medical Systems, Oro Valley, AZ, USA) and protein expression was quantified using the histoscore method as previously described^43^. The following antibodies were used: pAkt (Cell Signaling Technology, #4060, dilution 1:50) and phospho-PRAS40 (Cell Signaling Technology, #2997, dilution 1:100).

### Mice xenograft study

All animal experiments were approved by The Experimental Animal Committee of The Danish Ministry of Justice and were performed at the animal core facility at University of Southern Denmark. The mice were housed under specific pathogen-free conditions with ad libitum food and water.

Subconfluent ER10 and ER20 cells were harvested by accutase treatment and resuspended in a 1:1 mixture of extracellular matrix from Engelbreth-Holm-Swarm sarcoma (Sigma-Aldrich) and RPMI-1640 media, and injected subcutaneously into 8-week-old female CB17 SCID mice (Taconic). Tumor size was measured weekly by calipers, and after 15 days the mice were randomized to administration of the FGFRi (PD173074, 50 mg/kg), the Akti (GSK2141795, 10 mg/kg), the combination thereof or vehicle by oral gavage 5 days a week for 4 weeks #CB17SC-F).

Tumors were excluded from the final analysis if volumes at randomization were not evaluable. Tumor volumes at endpoint were calculated according to: tumor volume (mm^3^) = (length × width^2^)/2.

Animals were euthanized if they showed any adverse signs or symptoms of disease, including weight loss, paralysis, thymus dysfunction, or general discomfort. Accordingly, five mice were censored during the course of the study. PD173074 was formulated at 70 µg/g bodyweight and GSK2141795 was formulated at 10 µg/g bodyweight. Both drugs were kept in DMSO, but diluted into 15% Captisol (Captisol) upon administration; 15% Captisol was used as vehicle, and the concentration of DMSO did not exceed 10% when administered. Drugs were administered 5 days a week for 4 weeks by oral gavage. Maximum volume per mouse was 200 µL. Mouse bodyweight was surveyed throughout the study.

### Patient samples

The patient cohort consisted of EGFR-mutated NSCLC patients diagnosed in the Dexeus Quirón University Hospital (Barcelona, Spain), Germans Trias i Pujol Hospital (Badalona, Spain) and Fundación Santa Fe de Bogotá (Colombia). Studies were conducted in accordance with the Declaration of Helsinki and all relevant ethical regulations for work with human participants, under an approved protocol of the Institutional Review Board of Dexeus Quirón University Hospital and Germans Trias i Pujol Hospital. Samples were de-identified for patient confidentiality and informed written consent, also approved by the Institutional Review Boards, was obtained from all subjects.

### Statistics

For cell viability and BrdU incorporation assays, either Student’s *t*-test or ANOVA testing was employed, and statistical significance was defined as *P* < 0.05. For xenograft analysis, non-parametric Kruskal Wallis test with Dunn’s multiple comparison was employed, and statistical significance was defined as *P* < 0.05. The reported tumor size was calculated relative to the tumor size at treatment initiation. Tumor sizes were compared using the linear mixed effects model with categorical treatment groups and continuous time as fixed effects including the interaction. The model contains a random effect for the individual tumors to take repeated measurements within each mouse into account. The group-specific time slope is referred to as growth rates (GR) and the reported *P*-values are calculated on log-transformed data and represent the difference in GR of the combined FGFRi and Akti compared to control or either treatment alone. The combination index (CI) was calculated using the Bliss method.

PFS were estimated by the Kaplan-Meier method, and the non-parametric log-rank test was applied to compare the different groups. The selection of cutoff was empiric. First, the patient population was divided into four quartiles and Kaplan-Meier plots of each were analyzed and showed that the plots of Q1–Q3 were coincident (and therefore subsequently combined), while the Q0 patients had a longer PFS. Cox’s multivariate regression model was applied with FGFR1 levels as covariate, obtaining HR and 95% CI. Significance levels of less than 0.05 in the univariate model were used to select variables for the Cox multivariate regression model.

### Reporting summary

Further information on research design is available in the Nature Research Reporting Summary linked to this article.

## Supplementary information

Supplementary information.

Reporting summary.

## Data Availability

The mass spectrometry proteomics data generated during the study are publicly available in the PRIDE repository under the accession number https://identifiers.org/pride.project: PXD011803. Survival analyses and immunohistochemistry data are not publicly available to protect patient privacy, but will be made available to authorized researchers who have an approved Institutional Review Board application and have obtained approval from Dexeus Quirón University Hospital and Germans Trias i Pujol Hospital. Please contact the corresponding author with data access requests. The NGS data generated during the study are publicly available in the NCBI repository under the accession number https://www.ncbi.nlm.nih.gov/bioproject/?term=PRJNA524804 and sample accession numbers: SAMN11035315 (PC9-GR4); SAMN11035323 (11-18GR5), and https://www.ncbi.nlm.nih.gov/bioproject/?term=PRJNA734250 and sample accession numbers: SAMN19487316 (PC9GR4AZD2); SAMN19487317 (HCC827); SAMN19487318 (ER10); SAMN19487319 (ER20); SAMN19487320 (ER30). All other datasets generated during the study will be made available upon reasonable request to the corresponding author. Uncropped western blots are part of the supplementary information.
