# Supplementary material for: Combined FGFR and Akt pathway inhibition abrogates growth of FGFR1 overexpressing EGFR-TKI-resistant NSCLC cells
Source: NPJ Precis Oncol. 2021 Jul 15;5:65. doi: 10.1038/s41698-021-00208-w (PMC8282882; doi:10.1038/s41698-021-00208-w)
Supplement: Supplementary file 1 — Supplementary information. [file 41698_2021_208_MOESM1_ESM.pdf]

Supplementary Figures and Tables

SUPPLEMENTARY FIGURE 1

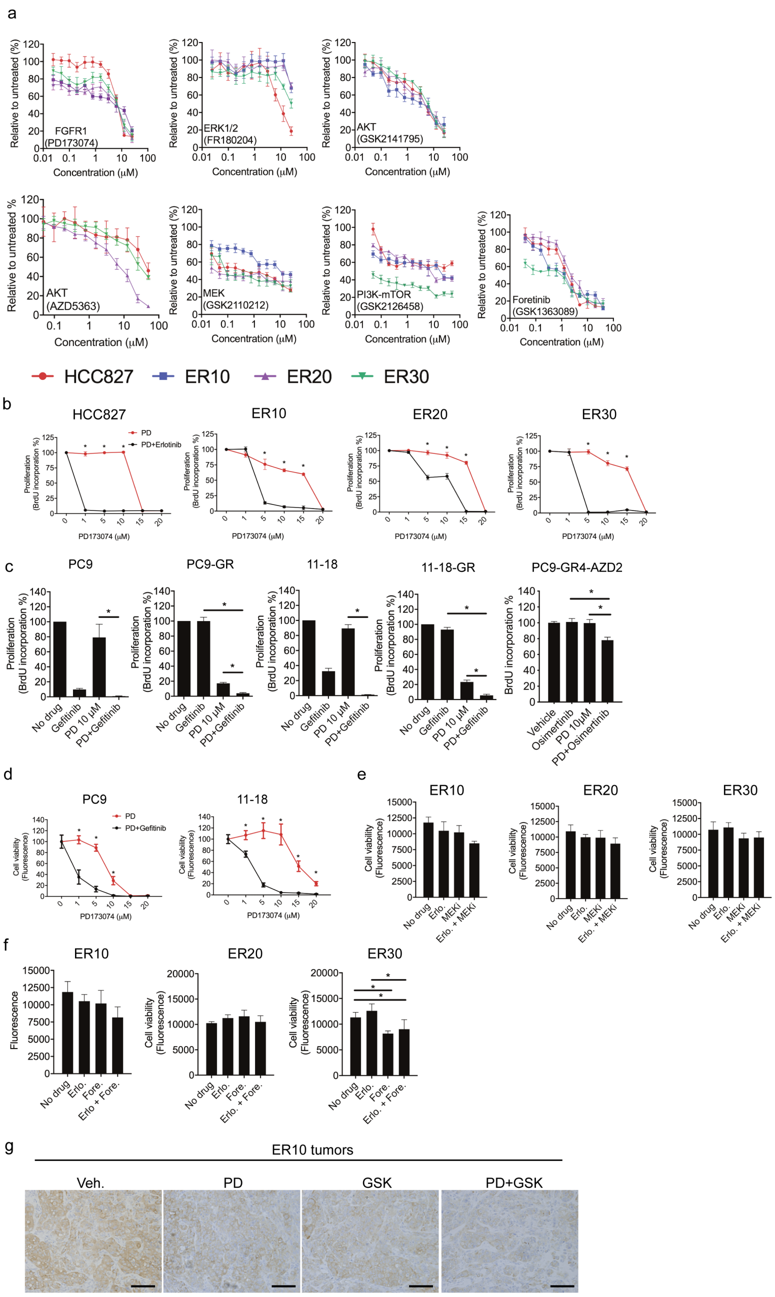

Supplementary figure 1. a Dose-response relationships on growth of the parental HCC827 and the three EGFR-TKI-resistant cell lines ER10, ER20 and ER30 evaluated by crystal violet assay after 72h incubation and using the following small molecule-specific inhibitors: FGFR1 inhibitor PD173074, ERK1/2 inhibitor FR180204, Akt inhibitor GSK2141795, Akt inhibitor AZD5363, MEK inhibitor GSK2110212, PI3K-mTOR inhibitor GSK2126458, and foretinib (GSK1363089). **b** BrdU incorporation assays assessing the anti-proliferative effect of erlotinib* combined with PD173074 (PD) after 5 days of incubation. Data are presented as mean of triplicates ± SD. *Asterisks* indicate significant difference in two tailed t-test ($p < 0.05$) for the drug combination-treated cells (PD173074 and erlotinib) compared to cells treated with PD173074 alone at same concentration. The zero-point of FGFR1 concentration represents untreated cells. **c** Proliferation assay performed with BrdU incorporation assessing the combinatorial effect of PD173074 (PD) and erlotinib in PC9 and PC9GR, 11-18 and 11-18GR as well as PC9-GR4-AZD2 after 5 days of incubation. Data in the BrdU assays are presented as mean of triplicates ± SD. *Asterisks* indicate significant difference in ANOVA one-way test ($p < 0.05$) for the drug combination-treated cells (PD173074 and erlotinib/osimertinib) compared to cells treated with the

gefitinib/osimertinib or PD173074 alone at same concentration. **d** Viability assay performed with CellTiterBlue assessing the combinatorial effect of PD173074 (10 μM) and gefitinib in PC9 and 11-18 over a range of PD173074 concentrations. Data are mean of seven replicates ± SD. *Asterisks* indicate significant difference in two tailed t-test ($p < 0.05$) for the drug combination-treated cells compared to cells treated with PD173074 alone at the same time point. **e** Viability assay performed with CellTiterBlue assessing the effect of EGFR-TKI (erlotinib, Erlo.) combined with MEK inhibitor GSK2110212 (5μM, MEKi). **f** Viability assay performed with CellTiterBlue assessing the effect of EGFR-TKI (erlotinib, Erlo.) combined with MET inhibitor foretinib (GSK1363089, 0.5μM, Fore). **g** Immunohistochemical stainings for pPRAS40 in ER10 tumors from mice treated with either Vehicle (Veh.), FGFRi alone (PD), Akti alone (GSK) and combined FGFRi and Akti (PD+GSK). Viability assays were performed after six days incubation. Data are mean of seven replicates ± SD. *Asterisks* indicate significant difference in ANOVA one-way test ($p < 0.05$) for the drug combination-treated cells compared to cells treated with either drug alone at the same time point. The zero-point of FGFR1 concentration represents untreated cells.

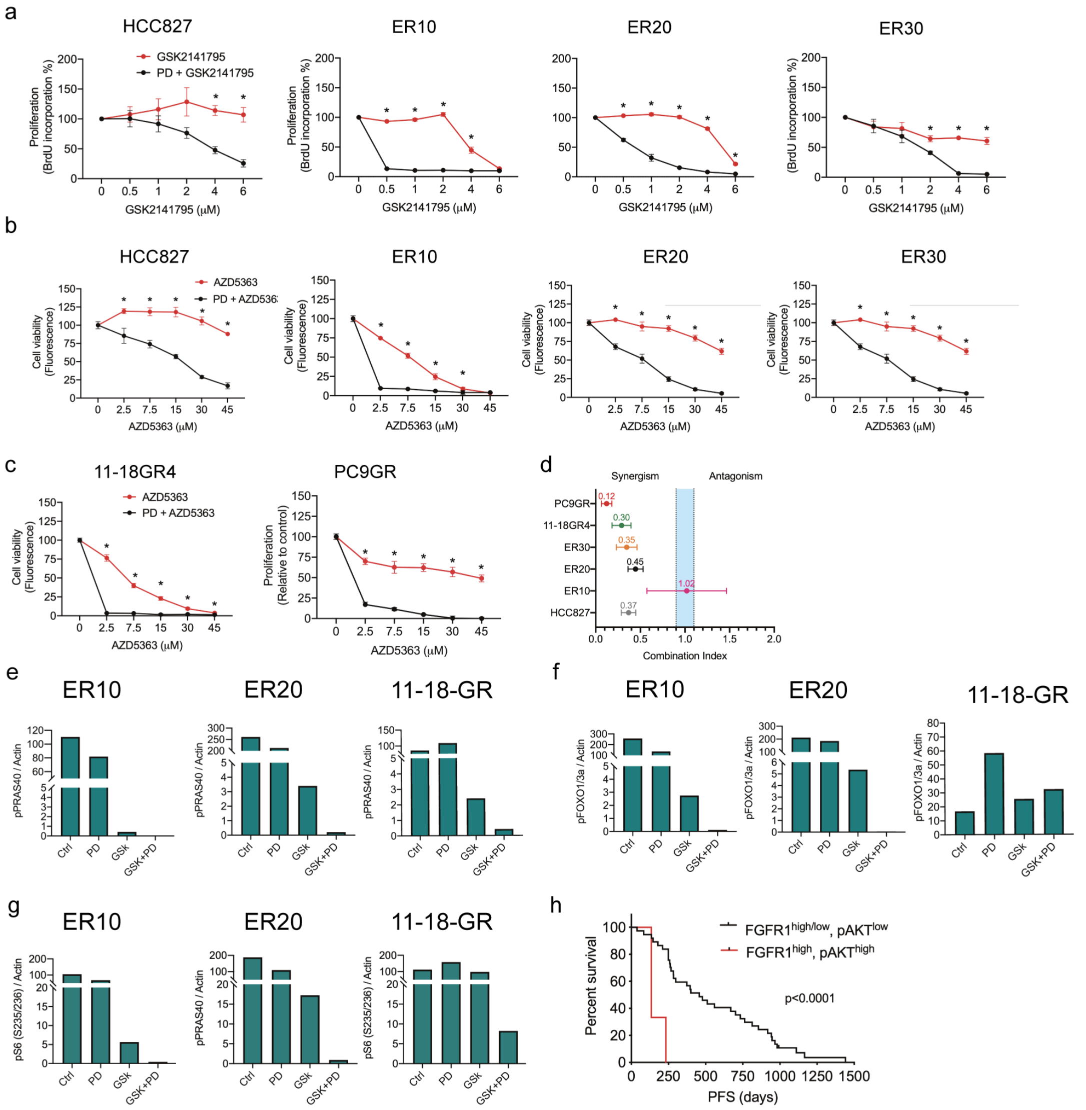

Supplementary Figure 2 a BrdU incorporation assays assessing the anti-proliferative effect of PD173074 combined with GSK2141795 after 5 days of incubation for ER10, ER20, ER30 and parental HCC827 cell lines. Data are presented as mean of six replicates ± SD. *Asterisks* indicate significant difference in two-tailed t-test ($p < 0.05$) for the drug combination-treated cells (PD173074 and GSK2141795) compared to cells treated with GSK2141795 alone at the same concentration **b** Viability assays performed with CellTiterBlue after 5 days for EGFR-TKI-resistant ER10, ER20, ER30 and parental HCC827 cell lines following exposure to the FGFR inhibitor (PD173074 (PD), 10 µM) in combination with a range of different concentrations of the Akt inhibitor AZD5363 (AZD)#. **c** Viability assays performed with CellTiterBlue after 5 days for EGFR-TKI-resistant PC9GR and 11-18GR4 cell lines following exposure to PD173074 (10 µM) in combination with a range of different concentrations of AZD5363. Data are presented as mean of seven replicates ± SD. *Asterisks* indicate significant difference in two-tailed t-test ($p < 0.05$) for the drug combination-treated cells (PD173074 and GSK2141795) compared to cells treated with GSK2141795 alone at the same concentration. **d** Synergisms between PD173074 (PD) and AZD5363 (AZD) in erlotinib and gefitinib-resistant cell lines as determined by range of combination indexes (CIs) from CellTiterBlue viability assays after 5 days of incubation. **e-g** The difference in band intensity between combined Akti and FGFR1 and Akti alone was confirmed using densitometry quantification of pPRAS40 (**e**), pFOXO1/3a (**f**)and pS6 (S235/236) (**g**) from the Western Blot in **figure 5**. The band intensity values of the target proteins were normalized to the band intensity of the corresponding actin band. **h** Kaplan-Meier curves illustrate correlations between FGFR1 mRNA expression and pAKT and progression-free survival (PFS) for the 40 patients treated with first-line EGFR-TKIs. #**For CellTiterBlue assays**: Data are presented as mean of seven replicates ± SD. *Asterisks* indicate significant difference in two-tailed t-test ($p < 0.05$) for the drug combination-treated cells (PD173074 and AZD5363) compared to cells treated with AZD5363 alone at the same concentration.

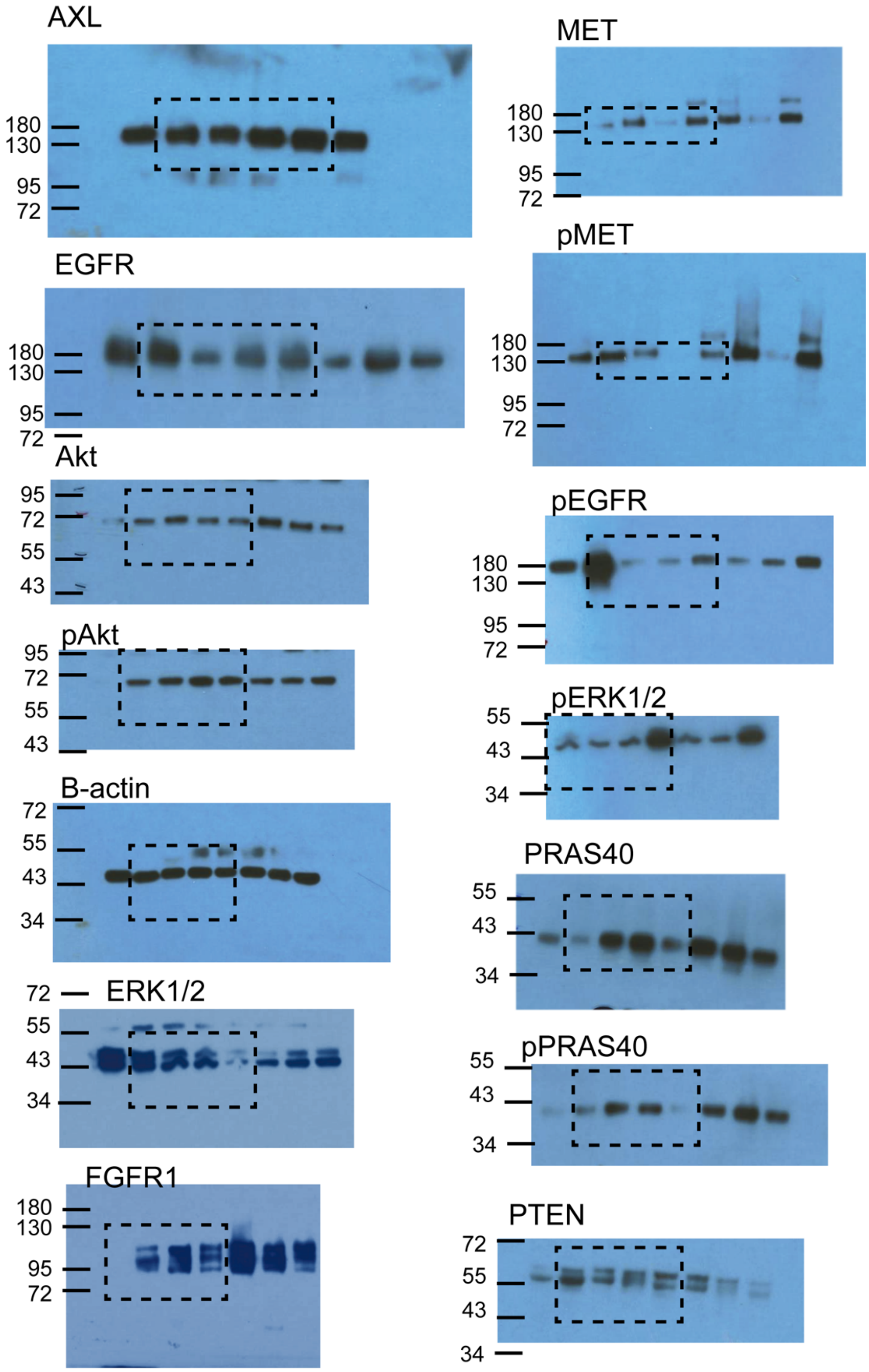

Supplementary Figure 3. Uncropped western blots from figure 1

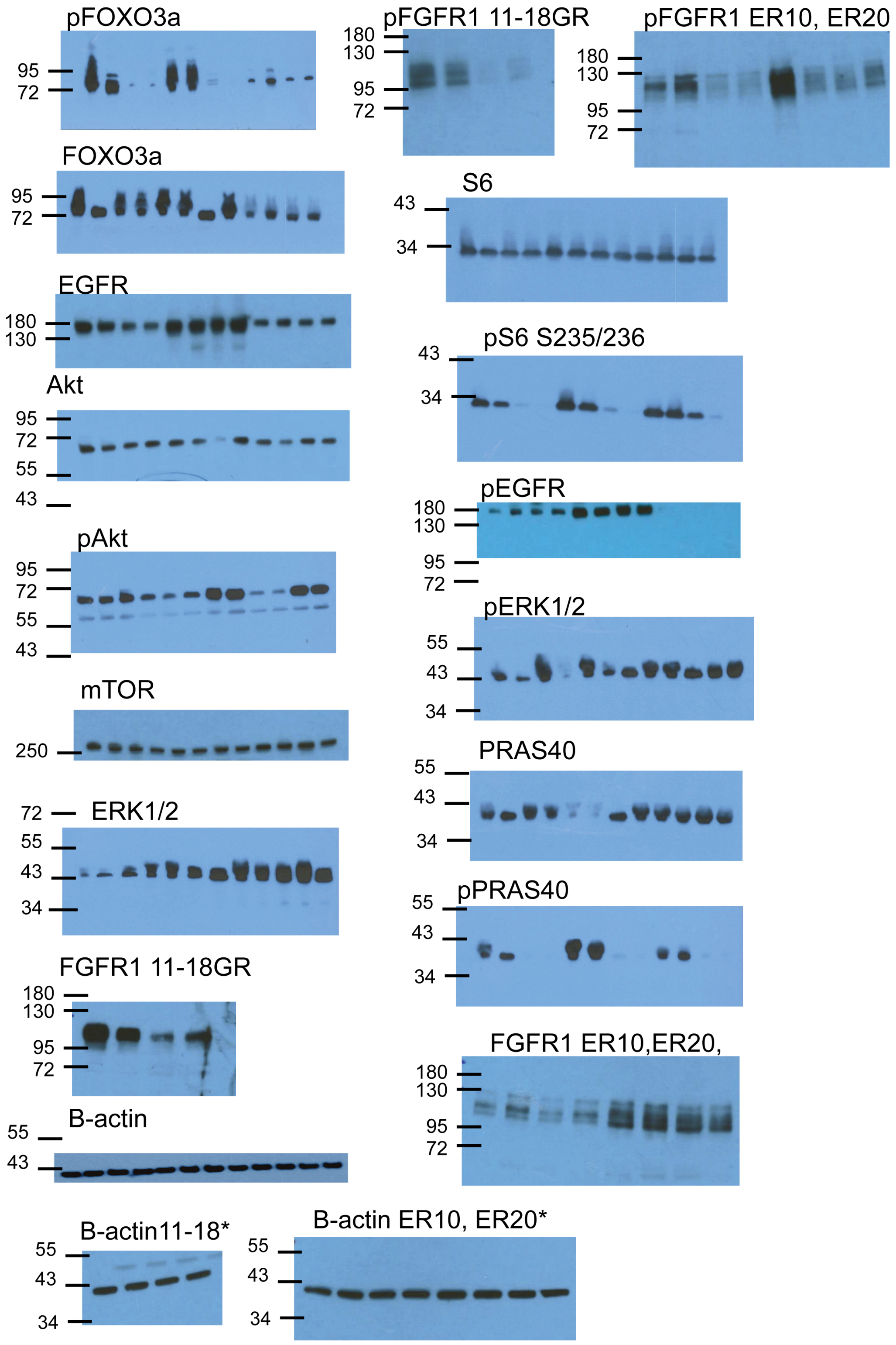

Supplementary Figure 4. Uncropped western blots from figure 3

Supplementary Table 1. Characteristics of the parental HCC827 cell line and the derived erlotinib-resistant cell lines.

	**Parental HCC827**	**ER10**	**ER20**	**ER30**
Phenotype	Sensitive	Resistant	Resistant	Resistant
IC50	0.005 µM	>50 µM	>50 µM	>50 µM
Exposed to conc. of erlotinib	0 µM	10 µM	20 µM	30 µM
EGFR status	Exon19del only	Exon19del only	Exon19del only	Exon19del only
KRAS status	wt	wt	wt	wt

Supplementary Table 2a and b:

See below at the end of the document

Supplementary Table 3. Patient characteristics of 40 patients with *EGFR*-mutant NSCLC treated with first line EGFR-TKIs (pre-treatment samples).

Patient Characteristics	**Value**
**Sex - no. (%)**	
Male	11 (27.5)
Female	29 (72.5)
**Age - years**	
Median	65
Range	42−88
**ECOG*performance status - no. (%)**	
0	9 (22.5)
1	21 (52.5)
2	9 (22.5)
3	1 (2.5)
**Smoking status - no. (%)**	
Never	27 (66.5)
Former	10 (25)
Current	3 (7.5)
**Disease stage - no. (%)**	
III	7 (17.5)
IV	33 (82.5)
**Brain metastasis - no. (%)**	
No	25 (62.5)
Yes	15 (37.5)
**Bone metastasis - no. (%)**	
No	25 (62.5)
Yes	15 (37.5)
**Type of EGFR mutation - no. (%)**	
Exon 19 deletion	29 (72.5)
L858R	10 (25)
Other**	1 (2.5)
**Type of EGFR-TKI - no. (%)**	
Erlotinib	22 (55.0)
Gefitinib	17 (42.5)
Afatinib	1 (2.5)

* ECOG, Eastern Cooperative Oncology Group
**Other, exon 20 insertion

Supplementary Table 4 Univariate and multivariate analysis of various parameters associated with PFS of NSCLC patients.

	Univariate			Multivariate		
	Hazard Ratio	95% CI	P	Hazard Ratio	95% CI	P
FGFR1 expression	2.82	1.21-6.56	0.015	2.60	1.05-6.41	0.037
Age (≤65 vs. >65)	0.98	0.51-1.85	0.953			
Smoking Yes/no	2.17	0.60-2.41	0.592			
TNM stage Stage III vs. Stage IV	2.81	1.14-6.87	0.024	2.63	1.13-6.14	0.024
Sex	0.89	0.44-1.81	0.761			

Supplementary Table 5. Evaluation of common mechanisms of resistance to EGFR TKI in a selection of the pre-treatment clinical samples. The expression of AXL, MET and HER2 as well as T790M mutations were evaluated in pre-treatment tumor samples where additional tissue was available and correlated with the FGFR1 mRNA expression.

ID	FGFR1 expression (high/low)	AXL expression (pos/neg)	Met expression (pos/neg)	HER2 expression (pos/neg)	T790M mutation
1	High	NEG	NEG	NEG	NEG
2	High	NEG	NEG	NEG	NEG
3	Low	NEG	NEG	NEG	NEG
4	Low	NEG	NEG	NEG	NEG
5	Low	NEG	NEG	NEG	NEG
6	High	NEG	NEG	NEG	NEG
7	Low	NEG	NEG	NEG	NEG
8	Low	NEG	NEG	NEG	NEG
9	High	NEG	NEG	NEG	NEG
10	Low	NEG	NEG	NEG	NEG
11	Low	NEG	NEG	NEG	NEG
12	Low	NEG	NEG	NEG	NEG
13	High	NEG	NEG	NEG	NEG
14	High	NEG	POS	NEG	NEG
15	Low	NEG	NEG	NEG	NEG
16	Low	NEG	POS	-	NEG
17	High	NEG	NEG	NEG	NEG
18	Low	NEG	NEG	NEG	NEG
19	Low	NEG	NEG	NEG	NEG
20	Low	NEG	NEG	NEG	NEG
21	Low	NEG	NEG	NEG	NEG

Supplementary Table 2a

Supplementary table 2a summarizes protein accesion numbers, protein descriptions, Mascot and Sequest scores, MW, calculated pI, number of unique peptides identified for each of the identified proteins in the membrane fractions (MEMB) and soluble fractions of erlotinib-resistant HCC827-cells resistant to 10, 20 or 30 uM erlotinib (ER10, ER20 or ER30) relative to the erlotinib-sensitive HCC827 cell line. Relative protein expression for proteins quantified with at least two unique peptides were log2 transformed and used for calculation of significantly regulated proteins in the erlotinib-resistant cell lines relative to the HCC827 control cell line as determined by the Perseus software as described in the methods section.

				10 uM erlotinib					20 uM erlotinib					30 uM erlotinib						
				ER10 MEMB/HCC827 (115/114)	ER10 MEMB/HCC827 (115/114)	ER10 SOL/HCC827 (115/114)	ER10 SOL/HCC827 (115/114)	ER10 SOL/HCC827 (115/114)	ER20 MEMB/HCC827 (116/114)	ER20 MEMB/HCC827 (116/114)	ER20 SOL/HCC827 (116/114)	ER20 SOL/HCC827 (116/114)	ER20 SOL/HCC827 (116/114)	ER30 MEMB/HCC827 (117/114)	ER30 MEMB/HCC827 (117/114)	ER30 SOL/HCC827 (117/114)	ER30 SOL/HCC827 (117/114)	ER30 SOL/HCC827 (117/114)		
Accession	Description	ΣCoverage	Σ# Unique Peptides	A5: 115/114	B5: 115/114	C5: 115/114	D5: 115/114	E5: 115/114	A5: 116/114	B5: 116/114	C5: 116/114	D5: 116/114	E5: 116/114	A5: 117/114	B5: 117/114	C5: 117/114	D5: 117/114	E5: 117/114	Score A2	Coverage A2
Q09666	Neuroblast differentiation-associated protein AHNAK OS=Homo sapiens GN=AHNAK PE=1 SV=2 - [AHNK_HUMAN]	77,88	293	1,816	1,920	1,287	1,305	1,398	1,871	**2,088**	1,578	1,660	1,836	1,489	1,575	1,914	**2,164**	**2,746**	25487,89	63,80
Q9NZM1	Myoferlin OS=Homo sapiens GN=MYOF PE=1 SV=1 - [MYOF_HUMAN]	57,16	108	1,462	1,454	1,188	1,103	1,269	0,913	0,916	0,998	0,798	0,852	0,963	0,966	0,667	0,625	0,647	14361,27	54,54
Q14315	Filamin-C OS=Homo sapiens GN=FLNC PE=1 SV=3 - [FLNC_HUMAN]	43,38	92	1,671	1,876	2,111	1,998	1,768	2,227	2,480	2,159	1,981	1,852	1,374	1,461	1,241	1,331	1,374	4923,32	23,71
P35579	Myosin-9 OS=Homo sapiens GN=MYH9 PE=1 SV=4 - [MYH9_HUMAN]	51,68	90	1,251	1,179	1,418	1,548	1,548	1,497	1,518	1,639	1,727	1,586	0,932	0,911	0,888	0,858	1,050	4910,76	37,55
P21333	Filamin-A OS=Homo sapiens GN=FLNA PE=1 SV=4 - [FLNA_HUMAN]	44,77	86	0,801	0,757	0,898	0,906	0,915	1,049	1,020	1,077	1,072	1,076	1,170	1,192	1,393	1,514	1,620	3612,49	24,59
O75369	Filamin-B OS=Homo sapiens GN=FLNB PE=1 SV=2 - [FLNB_HUMAN]	43,54	81	1,581	1,675	1,761	1,743	1,547	1,680	1,766	1,608	1,574	1,487	1,264	1,268	1,278	1,510	1,487	3419,37	25,17
Q13813	Spectrin alpha chain, brain OS=Homo sapiens GN=SPTAN1 PE=1 SV=3 - [SPTA2_HUMAN]	38,55	79	1,041	1,095	1,209	1,095	1,120	0,912	0,978	1,080	0,902	0,983	1,118	1,133	0,820	0,865	1,140	1812,14	21,24
Q86UP2	Kinectin OS=Homo sapiens GN=KTN1 PE=1 SV=1 - [KTN1_HUMAN]	52,76	68	1,164	1,102	1,245	1,327	1,112	0,980	0,997	0,981	1,067	0,932	0,936	0,938	0,889	1,018	1,118	4285,96	44,88
P19338	Nucleolin OS=Homo sapiens GN=NCL PE=1 SV=3 - [NUCL_HUMAN]	56,20	65	0,887	0,932	0,944	0,989	0,943	0,840	0,869	0,914	0,964	0,968	1,215	1,255	3,323	3,204	3,144	874,82	17,89
P78527	DNA-dependent protein kinase catalytic subunit OS=Homo sapiens GN=PRKDC PE=1 SV=3 - [PRKDC_HUMAN]	17,64	64	0,759	0,837	1,199	1,134	1,102	0,863	0,903	[illegible]	[illegible]	[illegible]	[illegible]	[illegible]	[illegible]	[illegible]	[illegible]	[illegible]	[illegible]
P11717	Cation-independent mannose-6-phosphate receptor OS=Homo sapiens GN=IGF2R PE=1 SV=3 - [MPRI_HUMAN]	30,31	63	1,973	1,968	1,295	1,599	1,509	1,325	1,317	[illegible]	[illegible]	[illegible]	[illegible]	[illegible]	[illegible]	[illegible]	[illegible]	[illegible]	[illegible]
P46013	Antigen KI-67 OS=Homo sapiens GN=MKI67 PE=1 SV=2 - [KI67_HUMAN]	24,36	60	0,791	0,701	1,003	0,926	0,873	0,927	1,050	[illegible]	[illegible]	[illegible]	[illegible]	[illegible]	[illegible]	[illegible]	[illegible]	[illegible]	[illegible]
Q14204	Cytoplasmic dynein 1 heavy chain 1 OS=Homo sapiens GN=DYNC1H1 PE=1 SV=5 - [DYHC1_HUMAN]	16,25	60	0,772	0,746	1,030	0,926	1,006	0,900	0,888	[illegible]	[illegible]	[illegible]	[illegible]	[illegible]	[illegible]	[illegible]	[illegible]	[illegible]	[illegible]
Q9P2E9	Ribosome-binding protein 1 OS=Homo sapiens GN=RRBP1 PE=1 SV=4 - [RRBP1_HUMAN]	58,51	56	0,763	0,739	0,803	0,909	0,916	0,782	0,802	[illegible]	[illegible]	[illegible]	[illegible]	[illegible]	[illegible]	[illegible]	[illegible]	[illegible]	[illegible]
[illegible]	[illegible]	[illegible]	[illegible]	[illegible]	[illegible]	[illegible]	[illegible]	[illegible]	[illegible]	[illegible]	[illegible]	[illegible]	[illegible]	[illegible]	[illegible]	[illegible]	[illegible]	[illegible]	[illegible]	[illegible]

P27797	Calreticulin OS=Homo sapiens GN=CALR PE=1 SV=1 - [CALR_HUMAN]	42,93	22	1,694	1,407	1,021	0,994	1,063
O76021	Ribosomal L1 domain-containing protein 1 OS=Homo sapiens GN=RSL1D1 PE=1 SV=3 - [RL1D1_HUMAN]	39,59	22	0,864	0,842	1,119	1,053	1,170
P16435	NADPH--cytochrome P450 reductase OS=Homo sapiens GN=POR PE=1 SV=2 - [NCPR_HUMAN]	37,96	22	0,987	1,015	1,004	0,847	
P28288	ATP-binding cassette sub-family D member 3 OS=Homo sapiens GN=ABCD3 PE=1 SV=1 - [ABCD3_HUMAN]	34,45	22	1,138	1,166		0,943	1,002
P26006	Integrin alpha-3 OS=Homo sapiens GN=ITGA3 PE=1 SV=5 - [ITA3_HUMAN]	25,12	22	1,263	1,333	0,721	1,085	1,142
O15438	Canalicular multispecific organic anion transporter 2 OS=Homo sapiens GN=ABCC3 PE=1 SV=3 - [MRP3_HUMAN]	19,38	22	1,062	1,090		0,770	1,138
P35658	Nuclear pore complex protein Nup214 OS=Homo sapiens GN=NUP214 PE=1 SV=2 - [NU214_HUMAN]	15,60	22	1,130	1,118	1,172	1,134	1,119
J3KN01	Afadin OS=Homo sapiens GN=MLLT4 PE=4 SV=1 - [J3KN01_HUMAN]	15,51	22	1,045	1,049			0,995
P40926	Malate dehydrogenase, mitochondrial OS=Homo sapiens GN=MDH2 PE=1 SV=3 - [MDHM_HUMAN]	65,98	21	0,861	0,790	1,591	1,481	1,438
P16401	Histone H1.5 OS=Homo sapiens GN=HIST1H1B PE=1 SV=3 - [H15_HUMAN]	63,27	21	0,955	1,017	0,921	0,921	0,895
P04075	Fructose-bisphosphate aldolase A OS=Homo sapiens GN=ALDOA PE=1 SV=2 - [ALDOA_HUMAN]	62,91	21	1,173	1,047	1,257	1,245	1,283
P06748	Nucleophosmin OS=Homo sapiens GN=NPM1 PE=1 SV=2 - [NPM_HUMAN]	61,56	21	1,095	1,170	1,459	1,318	1,202
P78371	T-complex protein 1 subunit beta OS=Homo sapiens GN=CCT2 PE=1 SV=4 - [TCPB_HUMAN]	47,29	21	0,845	0,884	0,831	0,794	0,846
P07910	Heterogeneous nuclear ribonucleoproteins C1/C2 OS=Homo sapiens GN=HNRNPC PE=1 SV=4 - [HNRPC_HUMAN]	45,75	21	0,772	0,862	1,029	0,975	1,047
Q9Y6N5	Sulfide:quinone oxidoreductase, mitochondrial OS=Homo sapiens GN=SQRDL PE=1 SV=1 - [SQRD_HUMAN]	42,44	21	0,690	0,611	0,672	0,643	0,602
Q96AE4-2	Isoform 2 of Far upstream element-binding protein 1 OS=Homo sapiens GN=FUBP1 - [FUBP1_HUMAN]	41,96	21	1,102	0,999	0,966	1,009	1,002
O95573	Long-chain-fatty-acid--CoA ligase 3 OS=Homo sapiens GN=ACSL3 PE=1 SV=3 - [ACSL3_HUMAN]	39,17	21	0,776	0,733	1,139		0,881
P42892	Endothelin-converting enzyme 1 OS=Homo sapiens GN=ECE1 PE=1 SV=2 - [ECE1_HUMAN]	30,65	21	0,668	0,656			
P29323	Ephrin type-B receptor 2 OS=Homo sapiens GN=EPHB2 PE=1 SV=5 - [EPHB2_HUMAN]	28,72	21	1,505	1,231			
P46087-2	Isoform 2 of Putative ribosomal RNA methyltransferase NOP2 OS=Homo sapiens GN=NOP2 - [NOP2_HUMAN]	27,72	21	1,002		0,886	0,938	0,881
Q02218	2-oxoglutarate dehydrogenase, mitochondrial OS=Homo sapiens GN=OGDH PE=1 SV=3 - [ODO1_HUMAN]	26,98	21	0,777	0,741	1,033	1,042	1,146
O75533	Splicing factor 3B subunit 1 OS=Homo sapiens GN=SF3B1 PE=1 SV=3 - [SF3B1_HUMAN]	20,55	21	0,841	0,727	1,167	1,133	0,898
Q86VP6	Cullin-associated NEDD8-dissociated protein 1 OS=Homo sapiens GN=CAND1 PE=1 SV=2 - [CAND1_HUMAN]	20,08	21	0,803	0,729	0,687	0,893	0,935
Q9C0C2	182 kDa tankyrase-1-binding protein OS=Homo sapiens GN=TNKS1BP1 PE=1 SV=4 - [TB182_HUMAN]	16,66	21	0,705	0,761	1,051	0,996	1,000
Q14571	Inositol 1,4,5-trisphosphate receptor type 2 OS=Homo sapiens GN=ITPR2 PE=1 SV=2 - [ITPR2_HUMAN]	11,66	21	1,222	1,252			
Q6P2Q9	Pre-mRNA-processing-splicing factor 8 OS=Homo sapiens GN=PRPF8 PE=1 SV=2 - [PRP8_HUMAN]	11,65	21	0,907	0,845		1,125	0,977
P08727	Keratin, type I cytoskeletal 19 OS=Homo sapiens GN=KRT19 PE=1 SV=4 - [K1C19_HUMAN]	73,00	20	0,520	0,472	0,393	0,383	0,616
Q99623	Prohibitin-2 OS=Homo sapiens GN=PHB2 PE=1 SV=2 - [PHB2_HUMAN]	62,21	20	1,182	1,176	1,104	0,959	0,872
P11142	Heat shock cognate 71 kDa protein OS=Homo sapiens GN=HSPA8 PE=1 SV=1 - [HSP7C_HUMAN]	51,24	20	1,022	0,968	0,931	1,007	1,006
P51572-2	Isoform 2 of B-cell receptor-associated protein 31 OS=Homo sapiens GN=BCAP31 - [BAP31_HUMAN]	48,24	20	0,834	1,038	0,802		
Q99832	T-complex protein 1 subunit eta OS=Homo sapiens GN=CCT7 PE=1 SV=2 - [TCPH_HUMAN]	46,41	20	0,916	0,768	0,779	0,963	0,953
P49257	Protein ERGIC-53 OS=Homo sapiens GN=LMAN1 PE=1 SV=2 - [LMAN1_HUMAN]	44,12	20	1,414	1,587			1,287
Q92945	Far upstream element-binding protein 2 OS=Homo sapiens GN=KHSRP PE=1 SV=4 - [FUBP2_HUMAN]	41,35	20	0,945	0,864	0,923	0,937	0,982
P25705	ATP synthase subunit alpha, mitochondrial OS=Homo sapiens GN=ATP5A1 PE=1 SV=1 - [ATPA_HUMAN]	39,96	20	0,804	0,784	0,992	1,072	1,059
P05362	Intercellular adhesion molecule 1 OS=Homo sapiens GN=ICAM1 PE=1 SV=2 - [ICAM1_HUMAN]	37,59	20	1,519	1,541	1,456	1,324	1,310
Q9H845	Acyl-CoA dehydrogenase family member 9, mitochondrial OS=Homo sapiens GN=ACAD9 PE=1 SV=1 - [ACAD9_HUMAN]	37,52	20	1,065	1,000	1,200	1,024	
Q8NF37	Lysophosphatidylcholine acyltransferase 1 OS=Homo sapiens GN=LPCAT1 PE=1 SV=2 - [PCAT1_HUMAN]	36,89	20	0,699	0,714	0,533	0,850	0,716
P29401	Transketolase OS=Homo sapiens GN=TKT PE=1 SV=3 - [TKT_HUMAN]	32,58	20	0,708	0,550	0,724	0,758	0,894
Q12931	Heat shock protein 75 kDa, mitochondrial OS=Homo sapiens GN=TRAP1 PE=1 SV=3 - [TRAP1_HUMAN]	32,24	20	0,772	0,755	0,945	0,959	0,994
Q13263	Transcription intermediary factor 1-beta OS=Homo sapiens GN=TRIM28 PE=1 SV=5 - [TIF1B_HUMAN]	31,86	20	0,797	0,772	0,811	0,836	0,860
Q8TCS8	Polyribonucleotide nucleotidyltransferase 1, mitochondrial OS=Homo sapiens GN=PNPT1 PE=1 SV=2 - [PNPT1_HUMAN]	31,16	20	0,721	0,502	0,817	0,818	0,797
Q92598	Heat shock protein 105 kDa OS=Homo sapiens GN=HSPH1 PE=1 SV=1 - [HS105_HUMAN]	29,02	20	0,789	0,704	1,065	1,056	0,859
Q9NSE4	Isoleucine--tRNA ligase, mitochondrial OS=Homo sapiens GN=IARS2 PE=1 SV=2 - [SYIM_HUMAN]	25,20	20	0,701	0,718	0,676	0,966	1,063
Q9HD20	Probable cation-transporting ATPase 13A1 OS=Homo sapiens GN=ATP13A1 PE=1 SV=2 - [AT131_HUMAN]	24,92	20	0,951	0,951	1,036		
Q13308	Inactive tyrosine-protein kinase 7 OS=Homo sapiens GN=PTK7 PE=1 SV=2 - [PTK7_HUMAN]	24,86	20	1,582	1,489			1,297
P55786	Puromycin-sensitive aminopeptidase OS=Homo sapiens GN=NPEPPS PE=1 SV=2 - [PSA_HUMAN]	24,59	20	0,705	0,658	0,820	1,004	0,864
Q27J81	Inverted formin-2 OS=Homo sapiens GN=INF2 PE=1 SV=2 - [INF2_HUMAN]	22,66	20	0,677	0,625	0,897	0,920	0,661
O15118	Niemann-Pick C1 protein OS=Homo sapiens GN=NPC1 PE=1 SV=2 - [NPC1_HUMAN]	19,80	20	1,874	2,005			
Q14152	Eukaryotic translation initiation factor 3 subunit A OS=Homo sapiens GN=EIF3A PE=1 SV=1 - [EIF3A_HUMAN]	15,56	20	0,871	0,818	0,989	1,034	0,989
P22626	Heterogeneous nuclear ribonucleoproteins A2/B1 OS=Homo sapiens GN=HNRNPA2B1 PE=1 SV=2 - [ROA2_HUMAN]	62,04	19	1,043	1,100	1,092	1,097	1,162
Q96AG4	Leucine-rich repeat-containing protein 59 OS=Homo sapiens GN=LRRC59 PE=1 SV=1 - [LRC59_HUMAN]	61,24	19	1,341	1,280	1,004	1,065	1,294
P78310	Coxsackievirus and adenovirus receptor OS=Homo sapiens GN=CXADR PE=1 SV=1 - [CXAR_HUMAN]	57,81	19	1,453	1,484	0,975	1,189	1,521
P56181-2	Isoform 2 of NADH dehydrogenase [ubiquinone] flavoprotein 3, mitochondrial OS=Homo sapiens GN=NDUFV3 - [NDUV3_HUMAN]	52,85	19	1,113	1,083	0,918	1,147	1,004
P30101	Protein disulfide-isomerase A3 OS=Homo sapiens GN=PDIA3 PE=1 SV=4 - [PDIA3_HUMAN]	49,70	19	1,284	1,243	0,830	0,890	0,866
Q14254	Flotillin-2 OS=Homo sapiens GN=FLOT2 PE=1 SV=2 - [FLOT2_HUMAN]	43,93	19	1,292	1,256			
P22695	Cytochrome b-c1 complex subunit 2, mitochondrial OS=Homo sapiens GN=UQCRC2 PE=1 SV=3 - [QCR2_HUMAN]	43,93	19	0,728	0,695	0,606	0,494	0,700
P36578	60S ribosomal protein L4 OS=Homo sapiens GN=RPL4 PE=1 SV=5 - [RL4_HUMAN]	41,45	19	0,837	0,843	0,898	0,988	0,971
P26038	Moesin OS=Homo sapiens GN=MSN PE=1 SV=3 - [MOES_HUMAN]	40,38	19	1,215	1,155	1,164	1,071	1,014
Q6NUK1	Calcium-binding mitochondrial carrier protein SCaMC-1 OS=Homo sapiens GN=SLC25A24 PE=1 SV=2 - [SCMC1_HUMAN]	38,57	19	1,037	1,073		0,690	1,073
P50990	T-complex protein 1 subunit theta OS=Homo sapiens GN=CCT8 PE=1 SV=4 - [TCPQ_HUMAN]	37,59	19	0,849	0,844	0,875	0,891	0,916
P12956	X-ray repair cross-complementing protein 6 OS=Homo sapiens GN=XRCC6 PE=1 SV=2 - [XRCC6_HUMAN]	36,62	19	0,820	0,900	1,011	1,003	1,005
O60488	Long-chain-fatty-acid--CoA ligase 4 OS=Homo sapiens GN=ACSL4 PE=1 SV=2 - [ACSL4_HUMAN]	36,15	19	0,473	0,534	0,776	0,731	0,788
Q8TBA6	Golgin subfamily A member 5 OS=Homo sapiens GN=GOLGA5 PE=1 SV=3 - [GOGA5_HUMAN]	35,43	19	0,976	0,898			
P04844	Dolichyl-diphosphooligosaccharide--protein glycosyltransferase subunit 2 OS=Homo sapiens GN=RPN2 PE=1 SV=3 - [RPN2_HUMAN]	35,18	19	0,955	1,027	0,892	1,279	1,144
P13010	X-ray repair cross-complementing protein 5 OS=Homo sapiens GN=XRCC5 PE=1 SV=3 - [XRCC5_HUMAN]	34,02	19	0,783	0,752	0,934	0,858	0,930
P35222	Catenin beta-1 OS=Homo sapiens GN=CTNNB1 PE=1 SV=1 - [CTNB1_HUMAN]	32,78	19	0,595	0,516	1,003	0,586	0,790
Q96TA2	ATP-dependent zinc metalloprotease YME1L1 OS=Homo sapiens GN=YME1L1 PE=1 SV=2 - [YMEL1_HUMAN]	30,53	19	1,216	1,207	1,129	0,925	1,168
O00461	Golgi integral membrane protein 4 OS=Homo sapiens GN=GOLIM4 PE=1 SV=1 - [GOLI4_HUMAN]	29,45	19	1,914	1,901			1,107
Q9BZF1	Oxysterol-binding protein-related protein 8 OS=Homo sapiens GN=OSBPL8 PE=1 SV=3 - [OSBL8_HUMAN]	24,18	19	1,265	1,328	0,824	1,024	
Q4KMQ2	Anoctamin-6 OS=Homo sapiens GN=ANO6 PE=1 SV=2 - [ANO6_HUMAN]	22,20	19	1,584	1,548			
Q16706	Alpha-mannosidase 2 OS=Homo sapiens GN=MAN2A1 PE=1 SV=2 - [MA2A1_HUMAN]	21,85	19	1,098	1,125		1,105	
P41252	Isoleucine--tRNA ligase, cytoplasmic OS=Homo sapiens GN=IARS PE=1 SV=2 - [SYIC_HUMAN]	17,91	19	0,972	0,961	1,148	1,062	1,120
Q15075	Early endosome antigen 1 OS=Homo sapiens GN=EEA1 PE=1 SV=2 - [EEA1_HUMAN]	15,31	19	1,411	1,799	1,244	1,150	1,327
Q14676	Mediator of DNA damage checkpoint protein 1 OS=Homo sapiens GN=MDC1 PE=1 SV=3 - [MDC1_HUMAN]	14,65	19	1,014	1,077	1,355	1,226	1,167
P07942	Laminin subunit beta-1 OS=Homo sapiens GN=LAMB1 PE=1 SV=2 - [LAMB1_HUMAN]	13,66	19	1,171	1,186	0,795	1,255	1,239
P80723	Brain acid soluble protein 1 OS=Homo sapiens GN=BASP1 PE=1 SV=2 - [BASP1_HUMAN]	89,43	18	2,127	2,262	1,739	1,692	2,091
P21796	Voltage-dependent anion-selective channel protein 1 OS=Homo sapiens GN=VDAC1 PE=1 SV=2 - [VDAC1_HUMAN]	81,63	18	1,414	1,587	1,043	1,149	1,327
Q9UJZ1	Stomatin-like protein 2 OS=Homo sapiens GN=STOML2 PE=1 SV=1 - [STML2_HUMAN]	59,55	18	1,130	1,089	0,889	0,944	1,035
P00338	L-lactate dehydrogenase A chain OS=Homo sapiens GN=LDHA PE=1 SV=2 - [LDHA_HUMAN]	53,61	18	1,007	0,901	0,810	0,912	0,986
Q02878	60S ribosomal protein L6 OS=Homo sapiens GN=RPL6 PE=1 SV=3 - [RL6_HUMAN]	48,26	18	0,895	0,882	0,927	0,980	1,281
P18031	Tyrosine-protein phosphatase non-receptor type 1 OS=Homo sapiens GN=PTPN1 PE=1 SV=1 - [PTN1_HUMAN]	46,90	18	1,291	1,424	1,625	1,118	1,020
Q9H4M9	EH domain-containing protein 1 OS=Homo sapiens GN=EHD1 PE=1 SV=2 - [EHD1_HUMAN]	43,07	18	0,850	0,810	0,867	0,891	0,861
P15586	N-acetylglucosamine-6-sulfatase OS=Homo sapiens GN=GNS PE=1 SV=3 - [GNS_HUMAN]	37,32	18	1,449	1,480	1,331	1,396	1,423
O00116	Alkyldihydroxyacetonephosphate synthase, peroxisomal OS=Homo sapiens GN=AGPS PE=1 SV=1 - [ADAS_HUMAN]	35,41	18	1,236	1,106	1,022	0,941	0,948
Q969V3	Nicalin OS=Homo sapiens GN=NCLN PE=1 SV=2 - [NCLN_HUMAN]	34,99	18	1,033	0,990			0,765
P14314	Glucosidase 2 subunit beta OS=Homo sapiens GN=PRKCSH PE=1 SV=2 - [GLU2B_HUMAN]	34,66	18	1,567	1,519	1,089	1,087	1,040
P07900-2	Isoform 2 of Heat shock protein HSP 90-alpha OS=Homo sapiens GN=HSP90AA1 - [HS90A_HUMAN]	34,66	18	0,757	1,034	0,988	0,897	0,749
P26641	Elongation factor 1-gamma OS=Homo sapiens GN=EEF1G PE=1 SV=3 - [EF1G_HUMAN]	33,18	18	0,934	0,833	1,086	1,016	1,075
P23246	Splicing factor, proline- and glutamine-rich OS=Homo sapiens GN=SFPQ PE=1 SV=2 - [SFPQ_HUMAN]	32,53	18	1,101	0,878	1,005	1,101	0,998
Q9Y5K6	CD2-associated protein OS=Homo sapiens GN=CD2AP PE=1 SV=1 - [CD2AP_HUMAN]	30,99	18	1,192	1,053	1,032	0,913	1,050
P05067	Amyloid beta A4 protein OS=Homo sapiens GN=APP PE=1 SV=3 - [A4_HUMAN]	30,91	18	1,214	1,324		1,451	
O94874	E3 UFM1-protein ligase 1 OS=Homo sapiens GN=UFL1 PE=1 SV=2 - [UFL1_HUMAN]	28,84	18	0,972	0,996			
Q6ZNB6	NF-X1-type zinc finger protein NFXL1 OS=Homo sapiens GN=NFXL1 PE=1 SV=2 - [NFXL1_HUMAN]	28,54	18	1,369	1,264	1,263		
Q14764	Major vault protein OS=Homo sapiens GN=MVP PE=1 SV=4 - [MVP_HUMAN]	28,44	18	0,872	0,728	0,804	0,891	0,857
Q9UGP8	Translocation protein SEC63 homolog OS=Homo sapiens GN=SEC63 PE=1 SV=2 - [SEC63_HUMAN]	28,42	18	1,429	1,378	1,307		1,530
Q06481	Amyloid-like protein 2 OS=Homo sapiens GN=APLP2 PE=1 SV=2 - [APLP2_HUMAN]	28,05	18	1,027	1,024			
Q96PD2	Discoidin, CUB and LCCL domain-containing protein 2 OS=Homo sapiens GN=DCBLD2 PE=1 SV=1 - [DCBD2_HUMAN]	26,97	18	1,524	1,588			
P10253	Lysosomal alpha-glucosidase OS=Homo sapiens GN=GAA PE=1 SV=4 - [LYAG_HUMAN]	25,42	18	1,017	1,182		0,971	0,901
P41250	Glycine--tRNA ligase OS=Homo sapiens GN=GARS PE=1 SV=3 - [SYG_HUMAN]	25,03	18	0,831	1,050	1,069	0,970	1,101
Q7KZF4	Staphylococcal nuclease domain-containing protein 1 OS=Homo sapiens GN=SND1 PE=1 SV=1 - [SND1_HUMAN]	24,95	18	0,933	0,925	0,970	1,033	1,089
Q9P206-2	Isoform 2 of Uncharacterized protein KIAA1522 OS=Homo sapiens GN=KIAA1522 - [K1522_HUMAN]	23,67	18	1,230	1,124		0,960	0,816
P22314	Ubiquitin-like modifier-activating enzyme 1 OS=Homo sapiens GN=UBA1 PE=1 SV=3 - [UBA1_HUMAN]	23,06	18	0,910	0,830	1,074	1,009	0,938
O75534	Cold shock domain-containing protein E1 OS=Homo sapiens GN=CSDE1 PE=1 SV=2 - [CSDE1_HUMAN]	22,93	18	1,000	0,956	1,138	1,187	1,001
Q5M775	Cytospin-B OS=Homo sapiens GN=SPECC1 PE=1 SV=1 - [CYTSB_HUMAN]	22,57	18	1,332	1,270	1,504		1,246
P78347	General transcription factor II-I OS=Homo sapiens GN=GTF2I PE=1 SV=2 - [GTF2I_HUMAN]	21,64	18	0,885	0,838	1,111	1,013	1,026
P26640	Valine--tRNA ligase OS=Homo sapiens GN=VARS PE=1 SV=4 - [SYVC_HUMAN]	19,46	18	0,911	0,718	1,268	0,976	1,151
Q9Y666	Solute carrier family 12 member 7 OS=Homo sapiens GN=SLC12A7 PE=1 SV=3 - [S12A7_HUMAN]	19,30	18	0,941	0,975	0,994		
O60841	Eukaryotic translation initiation factor 5B OS=Homo sapiens GN=EIF5B PE=1 SV=4 - [IF2P_HUMAN]	16,72	18	1,073	0,890	0,908	0,894	1,054
P53621	Coatomer subunit alpha OS=Homo sapiens GN=COPA PE=1 SV=2 - [COPA_HUMAN]	16,01	18	0,686	0,685	0,913	0,906	0,858
Q9ULH0-2	Isoform 2 of Kinase D-interacting substrate of 220 kDa OS=Homo sapiens GN=KIDINS220 - [KDIS_HUMAN]	13,70	18	1,155	1,101			
Q14008	Cytoskeleton-associated protein 5 OS=Homo sapiens GN=CKAP5 PE=1 SV=3 - [CKAP5_HUMAN]	9,60	18	0,936	0,839	1,251	1,089	0,960
Q9UQ35	Serine/arginine repetitive matrix protein 2 OS=Homo sapiens GN=SRRM2 PE=1 SV=2 - [SRRM2_HUMAN]	9,23	18		0,857	1,058	0,992	0,996
Q14789	Golgin subfamily B member 1 OS=Homo sapiens GN=GOLGB1 PE=1 SV=2 - [GOGB1_HUMAN]	7,67	18	0,789	0,600	0,991	0,941	0,911
P35232	Prohibitin OS=Homo sapiens GN=PHB PE=1 SV=1 - [PHB_HUMAN]	80,15	17	1,200	1,204	0,955	1,059	1,001
P60174	Triosephosphate isomerase OS=Homo sapiens GN=TPI1 PE=1 SV=3 - [TPIS_HUMAN]	71,33	17	0,741	0,691	0,784	0,785	0,808
Q14847	LIM and SH3 domain protein 1 OS=Homo sapiens GN=LASP1 PE=1 SV=2 - [LASP1_HUMAN]	53,64	17	1,674	1,851	1,714	1,922	1,846
O75955	Flotillin-1 OS=Homo sapiens GN=FLOT1 PE=1 SV=3 - [FLOT1_HUMAN]	50,59	17	1,336	1,317		0,940	1,407
Q8NBJ4	Golgi membrane protein 1 OS=Homo sapiens GN=GOLM1 PE=1 SV=1 - [GOLM1_HUMAN]	49,13	17	0,885	0,943	0,841		0,853
Q96S66	Chloride channel CLIC-like protein 1 OS=Homo sapiens GN=CLCC1 PE=1 SV=1 - [CLCC1_HUMAN]	41,20	17	1,516	1,472		1,323	
P31939	Bifunctional purine biosynthesis protein PURH OS=Homo sapiens GN=ATIC PE=1 SV=3 - [PUR9_HUMAN]	40,71	17	0,760	0,679	0,862	0,850	0,859
Q8IXI1	Mitochondrial Rho GTPase 2 OS=Homo sapiens GN=RHOT2 PE=1 SV=2 - [MIRO2_HUMAN]	39,64	17	1,172	1,219		1,007	1,020
Q9Y4P3	Transducin beta-like protein 2 OS=Homo sapiens GN=TBL2 PE=1 SV=1 - [TBL2_HUMAN]	39,60	17	1,025	1,150			
P55084	Trifunctional enzyme subunit beta, mitochondrial OS=Homo sapiens GN=HADHB PE=1 SV=3 - [ECHB_HUMAN]	38,19	17	0,815	0,766	1,057	1,049	1,107
P54577	Tyrosine--tRNA ligase, cytoplasmic OS=Homo sapiens GN=YARS PE=1 SV=4 - [SYYC_HUMAN]	37,88	17	0,604	0,611	1,199	1,026	1,049
P14923	Junction plakoglobin OS=Homo sapiens GN=JUP PE=1 SV=3 - [PLAK_HUMAN]	33,69	17	0,322	0,387	1,024	0,303	0,357
Q9UHD8	Septin-9 OS=Homo sapiens GN=SEPT9 PE=1 SV=2 - [SEPT9_HUMAN]	32,08	17	1,089	0,910	1,103	1,000	1,011
Q01813	6-phosphofructokinase type C OS=Homo sapiens GN=PFKP PE=1 SV=2 - [K6PP_HUMAN]	31,25	17	0,880	0,850	1,200	1,027	1,053
P17844	Probable ATP-dependent RNA helicase DDX5 OS=Homo sapiens GN=DDX5 PE=1 SV=1 - [DDX5_HUMAN]	31,11	17	0,642	0,783	1,012	1,217	0,923
B7Z468	Calpastatin OS=Homo sapiens GN=CAST PE=2 SV=1 - [B7Z468_HUMAN]	30,64	17	1,444	0,661	0,630	0,715	1,079
P49915	GMP synthase [glutamine-hydrolyzing] OS=Homo sapiens GN=GMPS PE=1 SV=1 - [GUAA_HUMAN]	30,01	17	1,004	0,912	0,693	0,912	0,978
P23634	Plasma membrane calcium-transporting ATPase 4 OS=Homo sapiens GN=ATP2B4 PE=1 SV=2 - [AT2B4_HUMAN]	29,41	17	1,701	1,869			0,943
O00571	ATP-dependent RNA helicase DDX3X OS=Homo sapiens GN=DDX3X PE=1 SV=3 - [DDX3X_HUMAN]	29,00	17	0,869	0,972	1,148	1,132	1,126
O95140	Mitofusin-2 OS=Homo sapiens GN=MFN2 PE=1 SV=3 - [MFN2_HUMAN]	27,61	17	1,699	1,605			
O00410	Importin-5 OS=Homo sapiens GN=IPO5 PE=1 SV=4 - [IPO5_HUMAN]	26,16	17	0,759	0,644	1,014		0,881
Q8WUM4	Programmed cell death 6-interacting protein OS=Homo sapiens GN=PDCD6IP PE=1 SV=1 - [PDC6I_HUMAN]	26,15	17	1,066	1,062	0,984	0,954	1,061
Q13724	Mannosyl-oligosaccharide glucosidase OS=Homo sapiens GN=MOGS PE=1 SV=5 - [MOGS_HUMAN]	25,93	17	0,974	1,010		0,945	
Q86XL3	Ankyrin repeat and LEM domain-containing protein 2 OS=Homo sapiens GN=ANKLE2 PE=1 SV=4 - [ANKL2_HUMAN]	25,16	17	1,242	1,300	1,583		
P48960	CD97 antigen OS=Homo sapiens GN=CD97 PE=1 SV=4 - [CD97_HUMAN]	25,03	17	1,004	1,021			
Q15029	116 kDa U5 small nuclear ribonucleoprotein component OS=Homo sapiens GN=EFTUD2 PE=1 SV=1 - [U5S1_HUMAN]	24,69	17	0,870	0,876	0,935	0,907	0,964
Q14974	Importin subunit beta-1 OS=Homo sapiens GN=KPNB1 PE=1 SV=2 - [IMB1_HUMAN]	24,20	17	0,789	0,807	1,114	1,078	0,873
P27487	Dipeptidyl peptidase 4 OS=Homo sapiens GN=DPP4 PE=1 SV=2 - [DPP4_HUMAN]	24,02	17	1,425	1,560		1,213	1,061
P54886	Delta-1-pyrroline-5-carboxylate synthase OS=Homo sapiens GN=ALDH18A1 PE=1 SV=2 - [P5CS_HUMAN]	23,77	17	0,840	0,796	0,954	0,805	1,020
Q6NUQ4	Transmembrane protein 214 OS=Homo sapiens GN=TMEM214 PE=1 SV=2 - [TM214_HUMAN]	23,22	17	1,029	0,950		0,980	
A8MXP9	Matrin-3 OS=Homo sapiens GN=MATR3 PE=4 SV=1 - [A8MXP9_HUMAN]	23,13	17		1,101	1,036	1,218	0,911
A4D2Q0	SUN domain-containing protein 1 OS=Homo sapiens GN=UNC84A PE=4 SV=1 - [A4D2Q0_HUMAN]	22,90	17	0,798	0,721			
B2CI53	Sodium bicarbonate cotransporter 3 OS=Homo sapiens GN=SLC4A7 PE=2 SV=1 - [B2CI53_HUMAN]	21,66	17	1,187	1,033			
Q9P2J5	Leucine--tRNA ligase, cytoplasmic OS=Homo sapiens GN=LARS PE=1 SV=2 - [SYLC_HUMAN]	18,88	17	0,910	1,100	0,927	1,174	1,481
Q13753	Laminin subunit gamma-2 OS=Homo sapiens GN=LAMC2 PE=1 SV=2 - [LAMC2_HUMAN]	18,69	17	0,756	0,642	0,242		0,607
Q9H7F0	Probable cation-transporting ATPase 13A3 OS=Homo sapiens GN=ATP13A3 PE=1 SV=4 - [AT133_HUMAN]	18,60	17	1,385	1,348			
Q6WKZ4	Rab11 family-interacting protein 1 OS=Homo sapiens GN=RAB11FIP1 PE=1 SV=2 - [RFIP1_HUMAN]	16,52	17	1,249	1,343		1,183	1,090
P11388	DNA topoisomerase 2-alpha OS=Homo sapiens GN=TOP2A PE=1 SV=3 - [TOP2A_HUMAN]	15,48	17	0,692	0,763	0,907	0,937	0,886
Q9UKV3	Apoptotic chromatin condensation inducer in the nucleus OS=Homo sapiens GN=ACIN1 PE=1 SV=2 - [ACINU_HUMAN]	12,23	17	0,861	0,929	0,931	1,154	1,149
Q9UIW2	Plexin-A1 OS=Homo sapiens GN=PLXNA1 PE=1 SV=3 - [PLXA1_HUMAN]	11,81	17	1,153	1,114			
A6NEM2	HCF N-terminal chain 2 OS=Homo sapiens GN=HCFC1 PE=4 SV=2 - [A6NEM2_HUMAN]	10,48	17			1,046	0,878	0,892
Q5T4S7	E3 ubiquitin-protein ligase UBR4 OS=Homo sapiens GN=UBR4 PE=1 SV=1 - [UBR4_HUMAN]	4,77	17	0,959	0,801	0,936	0,927	0,971
P51149	Ras-related protein Rab-7a OS=Homo sapiens GN=RAB7A PE=1 SV=1 - [RAB7A_HUMAN]	69,57	16	1,125	1,134		0,756	0,771
P62258	14-3-3 protein epsilon OS=Homo sapiens GN=YWHAE PE=1 SV=1 - [1433E_HUMAN]	65,49	16	0,858	0,869	1,003	1,016	1,017
P13804	Electron transfer flavoprotein subunit alpha, mitochondrial OS=Homo sapiens GN=ETFA PE=1 SV=1 - [ETFA_HUMAN]	58,56	16	0,607	0,494	0,889	0,901	0,944
Q9P0L0	Vesicle-associated membrane protein-associated protein A OS=Homo sapiens GN=VAPA PE=1 SV=3 - [VAPA_HUMAN]	58,23	16	1,302	1,235	1,533	1,304	1,174
P15880	40S ribosomal protein S2 OS=Homo sapiens GN=RPS2 PE=1 SV=2 - [RS2_HUMAN]	51,88	16	0,839	0,889	1,009	1,002	1,269
P07195	L-lactate dehydrogenase B chain OS=Homo sapiens GN=LDHB PE=1 SV=2 - [LDHB_HUMAN]	46,71	16	0,728	0,703	0,721	0,733	0,715
P09543	2',3'-cyclic-nucleotide 3'-phosphodiesterase OS=Homo sapiens GN=CNP PE=1 SV=2 - [CN37_HUMAN]	44,66	16	0,898	0,829	0,946	1,014	0,883
Q01518	Adenylyl cyclase-associated protein 1 OS=Homo sapiens GN=CAP1 PE=1 SV=5 - [CAP1_HUMAN]	44,00	16	0,966	1,096	0,968	1,056	1,118
Q9BTU6	Phosphatidylinositol 4-kinase type 2-alpha OS=Homo sapiens GN=PI4K2A PE=1 SV=1 - [P4K2A_HUMAN]	42,80	16	1,186	1,056		1,250	
P06576	ATP synthase subunit beta, mitochondrial OS=Homo sapiens GN=ATP5B PE=1 SV=3 - [ATPB_HUMAN]	39,32	16	0,584	0,648	0,957	0,952	1,024
P00505	Aspartate aminotransferase, mitochondrial OS=Homo sapiens GN=GOT2 PE=1 SV=3 - [AATM_HUMAN]	39,30	16	0,722	0,853	1,193	1,069	1,134
Q5SWX8	Protein odr-4 homolog OS=Homo sapiens GN=ODR4 PE=2 SV=1 - [ODR4_HUMAN]	38,55	16	1,154	1,200			
Q9UJS0	Calcium-binding mitochondrial carrier protein Aralar2 OS=Homo sapiens GN=SLC25A13 PE=1 SV=2 - [CMC2_HUMAN]	37,48	16	1,356	1,394		1,112	1,099
Q9Y512	Sorting and assembly machinery component 50 homolog OS=Homo sapiens GN=SAMM50 PE=1 SV=3 - [SAM50_HUMAN]	36,25	16	0,989	1,028			0,911
P15311	Ezrin OS=Homo sapiens GN=EZR PE=1 SV=4 - [EZRI_HUMAN]	36,01	16	0,683	0,708	0,892	0,873	0,793

Q15046	Lysine--tRNA ligase OS=Homo sapiens GN=KARS PE=1 SV=3 - [SYK_HUMAN]	33,17	16	[illegible]
Q6DD88	Atlastin-3 OS=Homo sapiens GN=ATL3 PE=1 SV=1 - [ATLA3_HUMAN]	31,24	16	[illegible]
P22307	Non-specific lipid-transfer protein OS=Homo sapiens GN=SCP2 PE=1 SV=2 - [NLTP_HUMAN]	30,35	16	[illegible]
Q9H0H5	Rac GTPase-activating protein 1 OS=Homo sapiens GN=RACGAP1 PE=1 SV=1 - [RGAP1_HUMAN]	28,80	16	[illegible]
P20020	Plasma membrane calcium-transporting ATPase 1 OS=Homo sapiens GN=ATP2B1 PE=1 SV=3 - [AT2B1_HUMAN]	28,62	16	[illegible]
P11216	Glycogen phosphorylase, brain form OS=Homo sapiens GN=PYGB PE=1 SV=5 - [PYGB_HUMAN]	27,40	16	[illegible]
Q9NXE4-2	Isoform 2 of Sphingomyelin phosphodiesterase 4 OS=Homo sapiens GN=SMPD4 - [NSMA3_HUMAN]	27,19	16	[illegible]
P98155	Very low-density lipoprotein receptor OS=Homo sapiens GN=VLDLR PE=1 SV=1 - [VLDLR_HUMAN]	25,09	16	[illegible]
Q8IWT6	Leucine-rich repeat-containing protein 8A OS=Homo sapiens GN=LRRC8A PE=1 SV=1 - [LRC8A_HUMAN]	25,06	16	[illegible]
Q13443	Disintegrin and metalloproteinase domain-containing protein 9 OS=Homo sapiens GN=ADAM9 PE=1 SV=1 - [ADAM9_HUMAN]	24,91	16	[illegible]
Q16513	Serine/threonine-protein kinase N2 OS=Homo sapiens GN=PKN2 PE=1 SV=1 - [PKN2_HUMAN]	22,97	16	[illegible]
Q9Y678	Coatomer subunit gamma OS=Homo sapiens GN=COPG PE=1 SV=1 - [COPG_HUMAN]	22,31	16	[illegible]
Q9P2B2	Prostaglandin F2 receptor negative regulator OS=Homo sapiens GN=PTGFRN PE=1 SV=2 - [FPRP_HUMAN]	21,50	16	[illegible]
P01130	Low-density lipoprotein receptor OS=Homo sapiens GN=LDLR PE=1 SV=1 - [LDLR_HUMAN]	21,05	16	[illegible]
P55060	Exportin-2 OS=Homo sapiens GN=CSE1L PE=1 SV=3 - [XPO2_HUMAN]	19,26	16	[illegible]
Q53EP0	Fibronectin type III domain-containing protein 3B OS=Homo sapiens GN=FNDC3B PE=1 SV=2 - [FND3B_HUMAN]	19,02	16	[illegible]
O14936	Peripheral plasma membrane protein CASK OS=Homo sapiens GN=CASK PE=1 SV=3 - [CSKP_HUMAN]	18,68	16	[illegible]
P55265	Double-stranded RNA-specific adenosine deaminase OS=Homo sapiens GN=ADAR PE=1 SV=4 - [DSRAD_HUMAN]	15,25	16	[illegible]
Q04721	Neurogenic locus notch homolog protein 2 OS=Homo sapiens GN=NOTCH2 PE=1 SV=3 - [NOTC2_HUMAN]	9,59	16	[illegible]
Q92508	Piezo-type mechanosensitive ion channel component 1 OS=Homo sapiens GN=PIEZO1 PE=1 SV=4 - [PIEZ1_HUMAN]	8,33	16	[illegible]
P07355	Annexin A2 OS=Homo sapiens GN=ANXA2 PE=1 SV=2 - [ANXA2_HUMAN]	62,83	15	[illegible]
O95292	Vesicle-associated membrane protein-associated protein B/C OS=Homo sapiens GN=VAPB PE=1 SV=3 - [VAPB_HUMAN]	60,08	15	[illegible]
P62917	60S ribosomal protein L8 OS=Homo sapiens GN=RPL8 PE=1 SV=2 - [RL8_HUMAN]	56,03	15	[illegible]
P61247	40S ribosomal protein S3a OS=Homo sapiens GN=RPS3A PE=1 SV=2 - [RS3A_HUMAN]	55,68	15	[illegible]
P62701	40S ribosomal protein S4, X isoform OS=Homo sapiens GN=RPS4X PE=1 SV=2 - [RS4X_HUMAN]	55,51	15	[illegible]
Q14344	Guanine nucleotide-binding protein subunit alpha-13 OS=Homo sapiens GN=GNA13 PE=1 SV=2 - [GNA13_HUMAN]	52,52	15	[illegible]
Q6PIU2	Neutral cholesterol ester hydrolase 1 OS=Homo sapiens GN=NCEH1 PE=1 SV=3 - [NCEH1_HUMAN]	52,21	15	[illegible]
Q9Y266	Nuclear migration protein nudC OS=Homo sapiens GN=NUDC PE=1 SV=1 - [NUDC_HUMAN]	46,53	15	[illegible]
Q16795	NADH dehydrogenase [ubiquinone] 1 alpha subcomplex subunit 9, mitochondrial OS=Homo sapiens GN=NDUFA9 PE=1 SV=2 - [NDUA9_H	45,62	15	[illegible]
P08107	Heat shock 70 kDa protein 1A/1B OS=Homo sapiens GN=HSPA1A PE=1 SV=5 - [HSP71_HUMAN]	45,24	15	[illegible]
Q9HDC9	Adipocyte plasma membrane-associated protein OS=Homo sapiens GN=APMAP PE=1 SV=2 - [APMAP_HUMAN]	41,83	15	[illegible]
P43490	Nicotinamide phosphoribosyltransferase OS=Homo sapiens GN=NAMPT PE=1 SV=1 - [NAMPT_HUMAN]	41,75	15	[illegible]
P35998	26S protease regulatory subunit 7 OS=Homo sapiens GN=PSMC2 PE=1 SV=3 - [PRS7_HUMAN]	37,88	15	[illegible]
P23526	Adenosylhomocysteinase OS=Homo sapiens GN=AHCY PE=1 SV=4 - [SAHH_HUMAN]	37,50	15	[illegible]
P13645	Keratin, type I cytoskeletal 10 OS=Homo sapiens GN=KRT10 PE=1 SV=6 - [K1C10_HUMAN]	37,16	15	[illegible]
P09622	Dihydrolipoyl dehydrogenase, mitochondrial OS=Homo sapiens GN=DLD PE=1 SV=2 - [DLDH_HUMAN]	35,76	15	[illegible]
P05455	Lupus La protein OS=Homo sapiens GN=SSB PE=1 SV=2 - [LA_HUMAN]	34,56	15	[illegible]
P21589	5'-nucleotidase OS=Homo sapiens GN=NT5E PE=1 SV=1 - [5NTD_HUMAN]	34,15	15	[illegible]
O00515	Ladinin-1 OS=Homo sapiens GN=LAD1 PE=1 SV=2 - [LAD1_HUMAN]	34,04	15	[illegible]
O15269	Serine palmitoyltransferase 1 OS=Homo sapiens GN=SPTLC1 PE=1 SV=1 - [SPTC1_HUMAN]	33,40	15	[illegible]
P04040	Catalase OS=Homo sapiens GN=CAT PE=1 SV=3 - [CATA_HUMAN]	32,83	15	[illegible]
Q96A33	Coiled-coil domain-containing protein 47 OS=Homo sapiens GN=CCDC47 PE=1 SV=1 - [CCD47_HUMAN]	31,88	15	[illegible]
O43175	D-3-phosphoglycerate dehydrogenase OS=Homo sapiens GN=PHGDH PE=1 SV=4 - [SERA_HUMAN]	30,21	15	[illegible]
P10515	Dihydrolipoyllysine-residue acetyltransferase component of pyruvate dehydrogenase complex, mitochondrial OS=Homo sapiens GN=DLAT	28,59	15	[illegible]
P49321	Nuclear autoantigenic sperm protein OS=Homo sapiens GN=NASP PE=1 SV=2 - [NASP_HUMAN]	28,30	15	[illegible]
Q6P1M0	Long-chain fatty acid transport protein 4 OS=Homo sapiens GN=SLC27A4 PE=1 SV=1 - [S27A4_HUMAN]	27,22	15	[illegible]
Q15459	Splicing factor 3A subunit 1 OS=Homo sapiens GN=SF3A1 PE=1 SV=1 - [SF3A1_HUMAN]	26,73	15	[illegible]
Q08J23	tRNA (cytosine(34)-C(5))-methyltransferase OS=Homo sapiens GN=NSUN2 PE=1 SV=2 - [NSUN2_HUMAN]	25,68	15	[illegible]
Q8IWA4	Mitofusin-1 OS=Homo sapiens GN=MFN1 PE=1 SV=2 - [MFN1_HUMAN]	25,64	15	[illegible]
Q5T8D3	Acyl-CoA-binding domain-containing protein 5 OS=Homo sapiens GN=ACBD5 PE=1 SV=1 - [ACBD5_HUMAN]	25,28	15	[illegible]
Q9H4G0-2	Isoform 2 of Band 4.1-like protein 1 OS=Homo sapiens GN=EPB41L1 - [E41L1_HUMAN]	25,03	15	[illegible]
O43290	U4/U6.U5 tri-snRNP-associated protein 1 OS=Homo sapiens GN=SART1 PE=1 SV=1 - [SNUT1_HUMAN]	23,63	15	[illegible]
Q92542	Nicastrin OS=Homo sapiens GN=NCSTN PE=1 SV=2 - [NICA_HUMAN]	22,14	15	[illegible]
O14786	Neuropilin-1 OS=Homo sapiens GN=NRP1 PE=1 SV=3 - [NRP1_HUMAN]	22,10	15	[illegible]
P78536	Disintegrin and metalloproteinase domain-containing protein 17 OS=Homo sapiens GN=ADAM17 PE=1 SV=1 - [ADA17_HUMAN]	21,97	15	[illegible]
Q9H5V8	CUB domain-containing protein 1 OS=Homo sapiens GN=CDCP1 PE=1 SV=3 - [CDCP1_HUMAN]	20,81	15	[illegible]
P12830	Cadherin-1 OS=Homo sapiens GN=CDH1 PE=1 SV=3 - [CADH1_HUMAN]	20,63	15	[illegible]
Q13435	Splicing factor 3B subunit 2 OS=Homo sapiens GN=SF3B2 PE=1 SV=2 - [SF3B2_HUMAN]	19,33	15	[illegible]
Q14BN4	Sarcolemmal membrane-associated protein OS=Homo sapiens GN=SLMAP PE=1 SV=1 - [SLMAP_HUMAN]	19,20	15	[illegible]
Q14739	Lamin-B receptor OS=Homo sapiens GN=LBR PE=1 SV=2 - [LBR_HUMAN]	19,19	15	[illegible]
Q05682	Caldesmon OS=Homo sapiens GN=CALD1 PE=1 SV=3 - [CALD1_HUMAN]	19,04	15	[illegible]
Q7Z2K6	Endoplasmic reticulum metallopeptidase 1 OS=Homo sapiens GN=ERMP1 PE=1 SV=2 - [ERMP1_HUMAN]	18,25	15	[illegible]
A6NJZ6	Immunoglobulin superfamily member 3 OS=Homo sapiens GN=IGSF3 PE=4 SV=1 - [A6NJZ6_HUMAN]	17,87	15	[illegible]
P07996	Thrombospondin-1 OS=Homo sapiens GN=THBS1 PE=1 SV=2 - [TSP1_HUMAN]	17,52	15	[illegible]
Q86VP1	Tax1-binding protein 1 OS=Homo sapiens GN=TAX1BP1 PE=1 SV=2 - [TAXB1_HUMAN]	17,36	15	[illegible]
P55011	Solute carrier family 12 member 2 OS=Homo sapiens GN=SLC12A2 PE=1 SV=1 - [S12A2_HUMAN]	17,08	15	[illegible]
O95347	Structural maintenance of chromosomes protein 2 OS=Homo sapiens GN=SMC2 PE=1 SV=2 - [SMC2_HUMAN]	14,29	15	[illegible]
O00468	Agrin OS=Homo sapiens GN=AGRN PE=1 SV=4 - [AGRIN_HUMAN]	10,46	15	[illegible]
P24821	Tenascin OS=Homo sapiens GN=TNC PE=1 SV=3 - [TENA_HUMAN]	8,86	15	[illegible]
O15230	Laminin subunit alpha-5 OS=Homo sapiens GN=LAMA5 PE=1 SV=8 - [LAMA5_HUMAN]	6,50	15	[illegible]
Q92520	Protein FAM3C OS=Homo sapiens GN=FAM3C PE=1 SV=1 - [FAM3C_HUMAN]	61,23	14	[illegible]
B1AK87	Capping protein (Actin filament) muscle Z-line, beta OS=Homo sapiens GN=CAPZB PE=4 SV=1 - [B1AK87_HUMAN]	59,62	14	[illegible]
P45880	Voltage-dependent anion-selective channel protein 2 OS=Homo sapiens GN=VDAC2 PE=1 SV=2 - [VDAC2_HUMAN]	58,50	14	[illegible]
Q9NX63	Coiled-coil-helix-coiled-coil-helix domain-containing protein 3, mitochondrial OS=Homo sapiens GN=CHCHD3 PE=1 SV=1 - [CHCH3_HUM	49,34	14	[illegible]
Q15738	Sterol-4-alpha-carboxylate 3-dehydrogenase, decarboxylating OS=Homo sapiens GN=NSDHL PE=1 SV=2 - [NSDHL_HUMAN]	47,72	14	[illegible]
P62191	26S protease regulatory subunit 4 OS=Homo sapiens GN=PSMC1 PE=1 SV=1 - [PRS4_HUMAN]	47,50	14	[illegible]
P04083	Annexin A1 OS=Homo sapiens GN=ANXA1 PE=1 SV=2 - [ANXA1_HUMAN]	47,40	14	[illegible]
P24752	Acetyl-CoA acetyltransferase, mitochondrial OS=Homo sapiens GN=ACAT1 PE=1 SV=1 - [THIL_HUMAN]	45,90	14	[illegible]
P63244	Guanine nucleotide-binding protein subunit beta-2-like 1 OS=Homo sapiens GN=GNB2L1 PE=1 SV=3 - [GBLP_HUMAN]	43,85	14	[illegible]
Q02127	Dihydroorotate dehydrogenase (quinone), mitochondrial OS=Homo sapiens GN=DHODH PE=1 SV=3 - [PYRD_HUMAN]	41,77	14	[illegible]
Q15084	Protein disulfide-isomerase A6 OS=Homo sapiens GN=PDIA6 PE=1 SV=1 - [PDIA6_HUMAN]	41,59	14	[illegible]
P18124	60S ribosomal protein L7 OS=Homo sapiens GN=RPL7 PE=1 SV=1 - [RL7_HUMAN]	41,13	14	[illegible]
Q9BS26	Endoplasmic reticulum resident protein 44 OS=Homo sapiens GN=ERP44 PE=1 SV=1 - [ERP44_HUMAN]	40,64	14	[illegible]
P49821	NADH dehydrogenase [ubiquinone] flavoprotein 1, mitochondrial OS=Homo sapiens GN=NDUFV1 PE=1 SV=4 - [NDUV1_HUMAN]	40,52	14	[illegible]
Q15050	Ribosome biogenesis regulatory protein homolog OS=Homo sapiens GN=RRS1 PE=1 SV=2 - [RRS1_HUMAN]	40,00	14	[illegible]
P35908	Keratin, type II cytoskeletal 2 epidermal OS=Homo sapiens GN=KRT2 PE=1 SV=2 - [K22E_HUMAN]	36,31	14	[illegible]
P00352	Retinal dehydrogenase 1 OS=Homo sapiens GN=ALDH1A1 PE=1 SV=2 - [AL1A1_HUMAN]	36,13	14	[illegible]
P11279	Lysosome-associated membrane glycoprotein 1 OS=Homo sapiens GN=LAMP1 PE=1 SV=3 - [LAMP1_HUMAN]	35,97	14	[illegible]
O75439	Mitochondrial-processing peptidase subunit beta OS=Homo sapiens GN=PMPCB PE=1 SV=2 - [MPPB_HUMAN]	34,15	14	[illegible]
Q53H12	Acylglycerol kinase, mitochondrial OS=Homo sapiens GN=AGK PE=1 SV=2 - [AGK_HUMAN]	33,41	14	[illegible]
Q9UGI8	Testin OS=Homo sapiens GN=TES PE=1 SV=1 - [TES_HUMAN]	32,78	14	[illegible]
A1L0T0	Acetolactate synthase-like protein OS=Homo sapiens GN=ILVBL PE=1 SV=2 - [ILVBL_HUMAN]	30,54	14	[illegible]
Q08380	Galectin-3-binding protein OS=Homo sapiens GN=LGALS3BP PE=1 SV=1 - [LG3BP_HUMAN]	29,23	14	[illegible]
Q9C0B5	Palmitoyltransferase ZDHHC5 OS=Homo sapiens GN=ZDHHC5 PE=1 SV=2 - [ZDHC5_HUMAN]	28,53	14	[illegible]
O00567	Nucleolar protein 56 OS=Homo sapiens GN=NOP56 PE=1 SV=4 - [NOP56_HUMAN]	25,59	14	[illegible]
Q9NTJ5	Phosphatidylinositide phosphatase SAC1 OS=Homo sapiens GN=SACM1L PE=1 SV=2 - [SAC1_HUMAN]	25,04	14	[illegible]
Q8N163	Protein KIAA1967 OS=Homo sapiens GN=KIAA1967 PE=1 SV=2 - [K1967_HUMAN]	23,51	14	[illegible]
Q7L1W4	Leucine-rich repeat-containing protein 8D OS=Homo sapiens GN=LRRC8D PE=1 SV=1 - [LRC8D_HUMAN]	23,43	14	[illegible]
Q13488	V-type proton ATPase 116 kDa subunit a isoform 3 OS=Homo sapiens GN=TCIRG1 PE=1 SV=3 - [VPP3_HUMAN]	23,13	14	[illegible]
P19440	Gamma-glutamyltranspeptidase 1 OS=Homo sapiens GN=GGT1 PE=1 SV=2 - [GGT1_HUMAN]	22,67	14	[illegible]
Q02241	Kinesin-like protein KIF23 OS=Homo sapiens GN=KIF23 PE=1 SV=3 - [KIF23_HUMAN]	21,88	14	[illegible]
Q9H0D6	5'-3' exoribonuclease 2 OS=Homo sapiens GN=XRN2 PE=1 SV=1 - [XRN2_HUMAN]	20,95	14	[illegible]
O43143	Putative pre-mRNA-splicing factor ATP-dependent RNA helicase DHX15 OS=Homo sapiens GN=DHX15 PE=1 SV=2 - [DHX15_HUMAN]	20,38	14	[illegible]
P46977	Dolichyl-diphosphooligosaccharide--protein glycosyltransferase subunit STT3A OS=Homo sapiens GN=STT3A PE=1 SV=2 - [STT3A_HUM	20,14	14	[illegible]
Q14118	Dystroglycan OS=Homo sapiens GN=DAG1 PE=1 SV=2 - [DAG1_HUMAN]	20,11	14	[illegible]
P78344	Eukaryotic translation initiation factor 4 gamma 2 OS=Homo sapiens GN=EIF4G2 PE=1 SV=1 - [IF4G2_HUMAN]	19,63	14	[illegible]
Q13200	26S proteasome non-ATPase regulatory subunit 2 OS=Homo sapiens GN=PSMD2 PE=1 SV=3 - [PSMD2_HUMAN]	18,17	14	[illegible]
Q9NW13	RNA-binding protein 28 OS=Homo sapiens GN=RBM28 PE=1 SV=3 - [RBM28_HUMAN]	17,92	14	[illegible]
Q1KMD3	Heterogeneous nuclear ribonucleoprotein U-like protein 2 OS=Homo sapiens GN=HNRNPUL2 PE=1 SV=1 - [HNRL2_HUMAN]	17,40	14	[illegible]
Q9NQW6	Actin-binding protein anillin OS=Homo sapiens GN=ANLN PE=1 SV=2 - [ANLN_HUMAN]	14,86	14	[illegible]
Q6YHK3	CD109 antigen OS=Homo sapiens GN=CD109 PE=1 SV=2 - [CD109_HUMAN]	12,87	14	[illegible]
Q5JTH9	RRP12-like protein OS=Homo sapiens GN=RRP12 PE=1 SV=2 - [RRP12_HUMAN]	12,64	14	[illegible]
P49790	Nuclear pore complex protein Nup153 OS=Homo sapiens GN=NUP153 PE=1 SV=2 - [NU153_HUMAN]	10,17	14	[illegible]
Q9NYU2	UDP-glucose:glycoprotein glucosyltransferase 1 OS=Homo sapiens GN=UGGT1 PE=1 SV=3 - [UGGG1_HUMAN]	10,16	14	[illegible]
J3KNL6	Protein transport protein Sec16A OS=Homo sapiens GN=SEC16A PE=4 SV=1 - [J3KNL6_HUMAN]	9,29	14	[illegible]
Q7Z6Z7	E3 ubiquitin-protein ligase HUWE1 OS=Homo sapiens GN=HUWE1 PE=1 SV=3 - [HUWE1_HUMAN]	4,14	14	[illegible]
P62937	Peptidyl-prolyl cis-trans isomerase A OS=Homo sapiens GN=PPIA PE=1 SV=2 - [PPIA_HUMAN]	65,45	13	[illegible]
P23396	40S ribosomal protein S3 OS=Homo sapiens GN=RPS3 PE=1 SV=2 - [RS3_HUMAN]	52,67	13	[illegible]
P29966	Myristoylated alanine-rich C-kinase substrate OS=Homo sapiens GN=MARCKS PE=1 SV=4 - [MARCS_HUMAN]	51,51	13	[illegible]
P62753	40S ribosomal protein S6 OS=Homo sapiens GN=RPS6 PE=1 SV=1 - [RS6_HUMAN]	47,39	13	[illegible]
P05198	Eukaryotic translation initiation factor 2 subunit 1 OS=Homo sapiens GN=EIF2S1 PE=1 SV=3 - [IF2A_HUMAN]	43,81	13	[illegible]
P31930	Cytochrome b-c1 complex subunit 1, mitochondrial OS=Homo sapiens GN=UQCRC1 PE=1 SV=3 - [QCR1_HUMAN]	43,33	13	[illegible]
P43686	26S protease regulatory subunit 6B OS=Homo sapiens GN=PSMC4 PE=1 SV=2 - [PRS6B_HUMAN]	43,06	13	[illegible]
P31689	DnaJ homolog subfamily A member 1 OS=Homo sapiens GN=DNAJA1 PE=1 SV=2 - [DNJA1_HUMAN]	42,82	13	[illegible]
O60664	Perilipin-3 OS=Homo sapiens GN=PLIN3 PE=1 SV=3 - [PLIN3_HUMAN]	40,78	13	[illegible]
Q3ZCQ8	Mitochondrial import inner membrane translocase subunit TIM50 OS=Homo sapiens GN=TIMM50 PE=1 SV=2 - [TIM50_HUMAN]	40,51	13	[illegible]
O00231	26S proteasome non-ATPase regulatory subunit 11 OS=Homo sapiens GN=PSMD11 PE=1 SV=3 - [PSD11_HUMAN]	39,57	13	[illegible]
Q02818	Nucleobindin-1 OS=Homo sapiens GN=NUCB1 PE=1 SV=4 - [NUCB1_HUMAN]	39,48	13	[illegible]
P62424	60S ribosomal protein L7a OS=Homo sapiens GN=RPL7A PE=1 SV=2 - [RL7A_HUMAN]	39,10	13	[illegible]
O00622	Protein CYR61 OS=Homo sapiens GN=CYR61 PE=1 SV=1 - [CYR61_HUMAN]	37,01	13	[illegible]
Q00325-2	Isoform B of Phosphate carrier protein, mitochondrial OS=Homo sapiens GN=SLC25A3 - [MPCP_HUMAN]	36,84	13	[illegible]
Q6UW02	Cytochrome P450 20A1 OS=Homo sapiens GN=CYP20A1 PE=1 SV=1 - [CP20A_HUMAN]	36,80	13	[illegible]
Q15233	Non-POU domain-containing octamer-binding protein OS=Homo sapiens GN=NONO PE=1 SV=4 - [NONO_HUMAN]	36,73	13	[illegible]
Q9UHQ9	NADH-cytochrome b5 reductase 1 OS=Homo sapiens GN=CYB5R1 PE=1 SV=1 - [NB5R1_HUMAN]	36,39	13	[illegible]
O75367	Core histone macro-H2A.1 OS=Homo sapiens GN=H2AFY PE=1 SV=4 - [H2AY_HUMAN]	35,48	13	[illegible]
P46777	60S ribosomal protein L5 OS=Homo sapiens GN=RPL5 PE=1 SV=3 - [RL5_HUMAN]	35,02	13	[illegible]
P07954	Fumarate hydratase, mitochondrial OS=Homo sapiens GN=FH PE=1 SV=3 - [FUMH_HUMAN]	34,51	13	[illegible]
Q9Y265	RuvB-like 1 OS=Homo sapiens GN=RUVBL1 PE=1 SV=1 - [RUVB1_HUMAN]	34,21	13	[illegible]
Q9UHQ4	B-cell receptor-associated protein 29 OS=Homo sapiens GN=BCAP29 PE=1 SV=2 - [BAP29_HUMAN]	33,20	13	[illegible]
Q96I99	Succinyl-CoA ligase [GDP-forming] subunit beta, mitochondrial OS=Homo sapiens GN=SUCLG2 PE=1 SV=2 - [SUCB2_HUMAN]	31,71	13	[illegible]
Q5VV42	CDK5 regulatory subunit-associated protein 1-like 1 OS=Homo sapiens GN=CDKAL1 PE=1 SV=1 - [CDKAL_HUMAN]	29,36	13	[illegible]
P48444	Coatomer subunit delta OS=Homo sapiens GN=ARCN1 PE=1 SV=1 - [COPD_HUMAN]	29,16	13	[illegible]
P31040	Succinate dehydrogenase [ubiquinone] flavoprotein subunit, mitochondrial OS=Homo sapiens GN=SDHA PE=1 SV=2 - [DHSA_HUMAN]	28,77	13	[illegible]
O75844	CAAX prenyl protease 1 homolog OS=Homo sapiens GN=ZMPSTE24 PE=1 SV=2 - [FACE1_HUMAN]	28,42	13	[illegible]
Q14195-2	Isoform LCRMP-4 of Dihydropyrimidinase-related protein 3 OS=Homo sapiens GN=DPYSL3 - [DPYL3_HUMAN]	28,22	13	[illegible]
P08243	Asparagine synthetase [glutamine-hydrolyzing] OS=Homo sapiens GN=ASNS PE=1 SV=4 - [ASNS_HUMAN]	27,81	13	[illegible]
Q16134	Electron transfer flavoprotein-ubiquinone oxidoreductase, mitochondrial OS=Homo sapiens GN=ETFDH PE=1 SV=2 - [ETFD_HUMAN]	27,23	13	[illegible]
Q15582	Transforming growth factor-beta-induced protein ig-h3 OS=Homo sapiens GN=TGFBI PE=1 SV=1 - [BGH3_HUMAN]	26,06	13	[illegible]
Q14247	Src substrate cortactin OS=Homo sapiens GN=CTTN PE=1 SV=2 - [SRC8_HUMAN]	25,64	13	[illegible]
P00367	Glutamate dehydrogenase 1, mitochondrial OS=Homo sapiens GN=GLUD1 PE=1 SV=2 - [DHE3_HUMAN]	25,09	13	[illegible]
Q14258	E3 ubiquitin/ISG15 ligase TRIM25 OS=Homo sapiens GN=TRIM25 PE=1 SV=2 - [TRI25_HUMAN]	25,08	13	[illegible]
Q15637-5	Isoform 5 of Splicing factor 1 OS=Homo sapiens GN=SF1 - [SF01_HUMAN]	25,00	13	[illegible]
Q96J84	Kin of IRRE-like protein 1 OS=Homo sapiens GN=KIRREL PE=1 SV=2 - [KIRR1_HUMAN]	24,97	13	[illegible]
P11940	Polyadenylate-binding protein 1 OS=Homo sapiens GN=PABPC1 PE=1 SV=2 - [PABP1_HUMAN]	24,53	13	[illegible]
P33176	Kinesin-1 heavy chain OS=Homo sapiens GN=KIF5B PE=1 SV=1 - [KINH_HUMAN]	24,09	13	[illegible]
Q13586	Stromal interaction molecule 1 OS=Homo sapiens GN=STIM1 PE=1 SV=3 - [STIM1_HUMAN]	23,65	13	[illegible]
Q6NSJ5	Leucine-rich repeat-containing protein 8E OS=Homo sapiens GN=LRRC8E PE=2 SV=2 - [LRC8E_HUMAN]	22,61	13	[illegible]
P23588	Eukaryotic translation initiation factor 4B OS=Homo sapiens GN=EIF4B PE=1 SV=2 - [IF4B_HUMAN]	22,42	13	[illegible]
P54136	Arginine--tRNA ligase, cytoplasmic OS=Homo sapiens GN=RARS PE=1 SV=2 - [SYRC_HUMAN]	22,27	13	[illegible]
Q9UBC2	Epidermal growth factor receptor substrate 15-like 1 OS=Homo sapiens GN=EPS15L1 PE=1 SV=1 - [EP15R_HUMAN]	21,53	13	[illegible]
Q9NRK6	ATP-binding cassette sub-family B member 10, mitochondrial OS=Homo sapiens GN=ABCB10 PE=1 SV=2 - [ABCBA_HUMAN]	21,27	13	[illegible]
Q99459	Cell division cycle 5-like protein OS=Homo sapiens GN=CDC5L PE=1 SV=2 - [CDC5L_HUMAN]	21,20	13	[illegible]
Q92544	Transmembrane 9 superfamily member 4 OS=Homo sapiens GN=TM9SF4 PE=1 SV=2 - [TM9S4_HUMAN]	20,72	13	[illegible]
Q96QK1	Vacuolar protein sorting-associated protein 35 OS=Homo sapiens GN=VPS35 PE=1 SV=2 - [VPS35_HUMAN]	19,85	13	[illegible]
P56192	Methionine--tRNA ligase, cytoplasmic OS=Homo sapiens GN=MARS PE=1 SV=2 - [SYMC_HUMAN]	19,56	13	[illegible]
P42224	Signal transducer and activator of transcription 1-alpha/beta OS=Homo sapiens GN=STAT1 PE=1 SV=2 - [STAT1_HUMAN]	19,33	13	[illegible]
P54760	Ephrin type-B receptor 4 OS=Homo sapiens GN=EPHB4 PE=1 SV=2 - [EPHB4_HUMAN]	18,95	13	[illegible]
Q93050	V-type proton ATPase 116 kDa subunit a isoform 1 OS=Homo sapiens GN=ATP6V0A1 PE=1 SV=3 - [VPP1_HUMAN]	18,16	13	[illegible]
Q9ULW0	Targeting protein for Xklp2 OS=Homo sapiens GN=TPX2 PE=1 SV=2 - [TPX2_HUMAN]	17,40	13	[illegible]
Q9NZ08	Endoplasmic reticulum aminopeptidase 1 OS=Homo sapiens GN=ERAP1 PE=1 SV=3 - [ERAP1_HUMAN]	16,58	13	[illegible]

P08648	Integrin alpha-5 OS=Homo sapiens GN=ITGA5 PE=1 SV=2 - [ITA5_HUMAN]	15,82	13	[illegible]
Q9Y2H6	Fibronectin type-III domain-containing protein 3A OS=Homo sapiens GN=FNDC3A PE=1 SV=4 - [FND3A_HUMAN]	14,52	13	[illegible]
O60271	C-Jun-amino-terminal kinase-interacting protein 4 OS=Homo sapiens GN=SPAG9 PE=1 SV=4 - [JIP4_HUMAN]	14,00	13	[illegible]
P06213	Insulin receptor OS=Homo sapiens GN=INSR PE=1 SV=4 - [INSR_HUMAN]	13,46	13	[illegible]
Q9Y4D1	Disheveled-associated activator of morphogenesis 1 OS=Homo sapiens GN=DAAM1 PE=1 SV=2 - [DAAM1_HUMAN]	13,45	13	[illegible]
Q9UQE7	Structural maintenance of chromosomes protein 3 OS=Homo sapiens GN=SMC3 PE=1 SV=2 - [SMC3_HUMAN]	12,82	13	[illegible]
Q9P2M7	Cingulin OS=Homo sapiens GN=CGN PE=1 SV=2 - [CING_HUMAN]	12,61	13	[illegible]
Q9P266	Junctional protein associated with coronary artery disease OS=Homo sapiens GN=KIAA1462 PE=1 SV=3 - [JCAD_HUMAN]	12,58	13	[illegible]
Q99758	ATP-binding cassette sub-family A member 3 OS=Homo sapiens GN=ABCA3 PE=1 SV=2 - [ABCA3_HUMAN]	10,04	13	[illegible]
Q16787	Laminin subunit alpha-3 OS=Homo sapiens GN=LAMA3 PE=1 SV=2 - [LAMA3_HUMAN]	4,74	13	[illegible]
Q14517	Protocadherin Fat 1 OS=Homo sapiens GN=FAT1 PE=1 SV=2 - [FAT1_HUMAN]	3,38	13	[illegible]
P61604	10 kDa heat shock protein, mitochondrial OS=Homo sapiens GN=HSPE1 PE=1 SV=2 - [CH10_HUMAN]	73,53	12	[illegible]
P61106	Ras-related protein Rab-14 OS=Homo sapiens GN=RAB14 PE=1 SV=4 - [RAB14_HUMAN]	72,56	12	[illegible]
P37802	Transgelin-2 OS=Homo sapiens GN=TAGLN2 PE=1 SV=3 - [TAGL2_HUMAN]	71,86	12	[illegible]
P23528	Cofilin-1 OS=Homo sapiens GN=CFL1 PE=1 SV=3 - [COF1_HUMAN]	71,69	12	[illegible]
P16402	Histone H1.3 OS=Homo sapiens GN=HIST1H1D PE=1 SV=2 - [H13_HUMAN]	66,06	12	[illegible]
P54819	Adenylate kinase 2, mitochondrial OS=Homo sapiens GN=AK2 PE=1 SV=2 - [KAD2_HUMAN]	62,34	12	[illegible]
O75947	ATP synthase subunit d, mitochondrial OS=Homo sapiens GN=ATP5H PE=1 SV=3 - [ATP5H_HUMAN]	62,11	12	[illegible]
Q9GZY8-2	Isoform 2 of Mitochondrial fission factor OS=Homo sapiens GN=MFF - [MFF_HUMAN]	61,17	12	[illegible]
O43399	Tumor protein D54 OS=Homo sapiens GN=TPD52L2 PE=1 SV=2 - [TPD54_HUMAN]	59,71	12	[illegible]
P68104	Elongation factor 1-alpha 1 OS=Homo sapiens GN=EEF1A1 PE=1 SV=1 - [EF1A1_HUMAN]	56,71	12	[illegible]
P05388	60S acidic ribosomal protein P0 OS=Homo sapiens GN=RPLP0 PE=1 SV=1 - [RLA0_HUMAN]	53,94	12	[illegible]
P27105	Erythrocyte band 7 integral membrane protein OS=Homo sapiens GN=STOM PE=1 SV=3 - [STOM_HUMAN]	53,47	12	[illegible]
P54709	Sodium/potassium-transporting ATPase subunit beta-3 OS=Homo sapiens GN=ATP1B3 PE=1 SV=1 - [AT1B3_HUMAN]	51,97	12	[illegible]
P38117	Electron transfer flavoprotein subunit beta OS=Homo sapiens GN=ETFB PE=1 SV=3 - [ETFB_HUMAN]	48,63	12	[illegible]
P21964	Catechol O-methyltransferase OS=Homo sapiens GN=COMT PE=1 SV=2 - [COMT_HUMAN]	41,70	12	[illegible]
Q9BRK5	45 kDa calcium-binding protein OS=Homo sapiens GN=SDF4 PE=1 SV=1 - [CAB45_HUMAN]	41,44	12	[illegible]
Q99729-3	Isoform 3 of Heterogeneous nuclear ribonucleoprotein A/B OS=Homo sapiens GN=HNRNPAB - [ROAA_HUMAN]	41,40	12	[illegible]
Q12904	Aminoacyl tRNA synthase complex-interacting multifunctional protein 1 OS=Homo sapiens GN=AIMP1 PE=1 SV=2 - [AIMP1_HUMAN]	41,35	12	[illegible]
P61421	V-type proton ATPase subunit d 1 OS=Homo sapiens GN=ATP6V0D1 PE=1 SV=1 - [VA0D1_HUMAN]	41,31	12	[illegible]
P07339	Cathepsin D OS=Homo sapiens GN=CTSD PE=1 SV=1 - [CATD_HUMAN]	39,81	12	[illegible]
Q9BTV4	Transmembrane protein 43 OS=Homo sapiens GN=TMEM43 PE=1 SV=1 - [TMM43_HUMAN]	39,75	12	[illegible]
Q9UQ80	Proliferation-associated protein 2G4 OS=Homo sapiens GN=PA2G4 PE=1 SV=3 - [PA2G4_HUMAN]	39,09	12	[illegible]
P05026	Sodium/potassium-transporting ATPase subunit beta-1 OS=Homo sapiens GN=ATP1B1 PE=1 SV=1 - [AT1B1_HUMAN]	38,94	12	[illegible]
Q14103-3	Isoform 3 of Heterogeneous nuclear ribonucleoprotein D0 OS=Homo sapiens GN=HNRNPD - [HNRPD_HUMAN]	35,95	12	[illegible]
Q02790	Peptidyl-prolyl cis-trans isomerase FKBP4 OS=Homo sapiens GN=FKBP4 PE=1 SV=3 - [FKBP4_HUMAN]	33,99	12	[illegible]
Q9UBS4	DnaJ homolog subfamily B member 11 OS=Homo sapiens GN=DNAJB11 PE=1 SV=1 - [DJB11_HUMAN]	33,80	12	[illegible]
P38919	Eukaryotic initiation factor 4A-III OS=Homo sapiens GN=EIF4A3 PE=1 SV=4 - [IF4A3_HUMAN]	33,09	12	[illegible]
P61158	Actin-related protein 3 OS=Homo sapiens GN=ACTR3 PE=1 SV=3 - [ARP3_HUMAN]	32,54	12	[illegible]
Q14165	Malectin OS=Homo sapiens GN=MLEC PE=1 SV=1 - [MLEC_HUMAN]	31,85	12	[illegible]
O75306	NADH dehydrogenase [ubiquinone] iron-sulfur protein 2, mitochondrial OS=Homo sapiens GN=NDUFS2 PE=1 SV=2 - [NDUS2_HUMAN]	31,75	12	[illegible]
O15355	Protein phosphatase 1G OS=Homo sapiens GN=PPM1G PE=1 SV=1 - [PPM1G_HUMAN]	31,68	12	[illegible]
P39656	Dolichyl-diphosphooligosaccharide--protein glycosyltransferase 48 kDa subunit OS=Homo sapiens GN=DDOST PE=1 SV=4 - [OST48_HUM	31,36	12	[illegible]
Q96CS3	FAS-associated factor 2 OS=Homo sapiens GN=FAF2 PE=1 SV=2 - [FAF2_HUMAN]	31,24	12	[illegible]
P28838	Cytosol aminopeptidase OS=Homo sapiens GN=LAP3 PE=1 SV=3 - [AMPL_HUMAN]	30,83	12	[illegible]
P41091	Eukaryotic translation initiation factor 2 subunit 3 OS=Homo sapiens GN=EIF2S3 PE=1 SV=3 - [IF2G_HUMAN]	30,08	12	[illegible]
Q9BQE5	Apolipoprotein L2 OS=Homo sapiens GN=APOL2 PE=1 SV=1 - [APOL2_HUMAN]	29,67	12	[illegible]
O95831	Apoptosis-inducing factor 1, mitochondrial OS=Homo sapiens GN=AIFM1 PE=1 SV=1 - [AIFM1_HUMAN]	29,20	12	[illegible]
Q96TC7	Regulator of microtubule dynamics protein 3 OS=Homo sapiens GN=FAM82A2 PE=1 SV=2 - [RMD3_HUMAN]	29,15	12	[illegible]
Q8IZ21	Phosphatase and actin regulator 4 OS=Homo sapiens GN=PHACTR4 PE=1 SV=1 - [PHAR4_HUMAN]	28,92	12	[illegible]
O43242	26S proteasome non-ATPase regulatory subunit 3 OS=Homo sapiens GN=PSMD3 PE=1 SV=2 - [PSMD3_HUMAN]	28,28	12	[illegible]
Q8TBX8	Phosphatidylinositol-5-phosphate 4-kinase type-2 gamma OS=Homo sapiens GN=PIP4K2C PE=1 SV=3 - [PI42C_HUMAN]	28,03	12	[illegible]
P50895	Basal cell adhesion molecule OS=Homo sapiens GN=BCAM PE=1 SV=2 - [BCAM_HUMAN]	27,87	12	[illegible]
O15270	Serine palmitoyltransferase 2 OS=Homo sapiens GN=SPTLC2 PE=1 SV=1 - [SPTC2_HUMAN]	27,22	12	[illegible]
P07099	Epoxide hydrolase 1 OS=Homo sapiens GN=EPHX1 PE=1 SV=1 - [HYEP_HUMAN]	26,59	12	[illegible]
O43615	Mitochondrial import inner membrane translocase subunit TIM44 OS=Homo sapiens GN=TIMM44 PE=1 SV=2 - [TIM44_HUMAN]	25,44	12	[illegible]
Q9BVP2	Guanine nucleotide-binding protein-like 3 OS=Homo sapiens GN=GNL3 PE=1 SV=2 - [GNL3_HUMAN]	24,77	12	[illegible]
Q14435	Polypeptide N-acetylgalactosaminyltransferase 3 OS=Homo sapiens GN=GALNT3 PE=2 SV=2 - [GALT3_HUMAN]	24,01	12	[illegible]
P06737	Glycogen phosphorylase, liver form OS=Homo sapiens GN=PYGL PE=1 SV=4 - [PYGL_HUMAN]	23,97	12	[illegible]
O43278	Kunitz-type protease inhibitor 1 OS=Homo sapiens GN=SPINT1 PE=1 SV=2 - [SPIT1_HUMAN]	23,44	12	[illegible]
Q9NZW5	MAGUK p55 subfamily member 6 OS=Homo sapiens GN=MPP6 PE=1 SV=2 - [MPP6_HUMAN]	23,33	12	[illegible]
P08582	Melanotransferrin OS=Homo sapiens GN=MFI2 PE=1 SV=2 - [TRFM_HUMAN]	21,68	12	[illegible]
Q16881	Thioredoxin reductase 1, cytoplasmic OS=Homo sapiens GN=TXNRD1 PE=1 SV=3 - [TRXR1_HUMAN]	21,42	12	[illegible]
O75083	WD repeat-containing protein 1 OS=Homo sapiens GN=WDR1 PE=1 SV=4 - [WDR1_HUMAN]	21,29	12	[illegible]
Q12929	Epidermal growth factor receptor kinase substrate 8 OS=Homo sapiens GN=EPS8 PE=1 SV=1 - [EPS8_HUMAN]	20,80	12	[illegible]
Q6NUM9	All-trans-retinol 13,14-reductase OS=Homo sapiens GN=RETSAT PE=2 SV=2 - [RETST_HUMAN]	20,33	12	[illegible]
Q96RP9	Elongation factor G, mitochondrial OS=Homo sapiens GN=GFM1 PE=1 SV=2 - [EFGM_HUMAN]	20,11	12	[illegible]
P16070	CD44 antigen OS=Homo sapiens GN=CD44 PE=1 SV=3 - [CD44_HUMAN]	19,81	12	[illegible]
P22102	Trifunctional purine biosynthetic protein adenosine-3 OS=Homo sapiens GN=GART PE=1 SV=1 - [PUR2_HUMAN]	19,70	12	[illegible]
P30530	Tyrosine-protein kinase receptor UFO OS=Homo sapiens GN=AXL PE=1 SV=3 - [UFO_HUMAN]	19,24	12	[illegible]
Q96PK6	RNA-binding protein 14 OS=Homo sapiens GN=RBM14 PE=1 SV=2 - [RBM14_HUMAN]	19,13	12	[illegible]
O75326	Semaphorin-7A OS=Homo sapiens GN=SEMA7A PE=1 SV=1 - [SEM7A_HUMAN]	19,07	12	[illegible]
Q9Y446	Plakophilin-3 OS=Homo sapiens GN=PKP3 PE=1 SV=1 - [PKP3_HUMAN]	18,95	12	[illegible]
Q92499	ATP-dependent RNA helicase DDX1 OS=Homo sapiens GN=DDX1 PE=1 SV=2 - [DDX1_HUMAN]	18,38	12	[illegible]
Q8TCJ2	Dolichyl-diphosphooligosaccharide--protein glycosyltransferase subunit STT3B OS=Homo sapiens GN=STT3B PE=1 SV=1 - [STT3B_HUM	18,28	12	[illegible]
Q15424	Scaffold attachment factor B1 OS=Homo sapiens GN=SAFB PE=1 SV=4 - [SAFB1_HUMAN]	18,25	12	[illegible]
Q12959	Disks large homolog 1 OS=Homo sapiens GN=DLG1 PE=1 SV=2 - [DLG1_HUMAN]	18,03	12	[illegible]
P46459	Vesicle-fusing ATPase OS=Homo sapiens GN=NSF PE=1 SV=3 - [NSF_HUMAN]	17,88	12	[illegible]
Q9NZB2	Constitutive coactivator of PPAR-gamma-like protein 1 OS=Homo sapiens GN=FAM120A PE=1 SV=2 - [F120A_HUMAN]	14,40	12	[illegible]
P42285	Superkiller viralicidic activity 2-like 2 OS=Homo sapiens GN=SKIV2L2 PE=1 SV=3 - [SK2L2_HUMAN]	13,82	12	[illegible]
Q12913	Receptor-type tyrosine-protein phosphatase eta OS=Homo sapiens GN=PTPRJ PE=1 SV=3 - [PTPRJ_HUMAN]	12,12	12	[illegible]
Q7L014	Probable ATP-dependent RNA helicase DDX46 OS=Homo sapiens GN=DDX46 PE=1 SV=2 - [DDX46_HUMAN]	10,96	12	[illegible]
Q92878	DNA repair protein RAD50 OS=Homo sapiens GN=RAD50 PE=1 SV=1 - [RAD50_HUMAN]	10,90	12	[illegible]
Q15393	Splicing factor 3B subunit 3 OS=Homo sapiens GN=SF3B3 PE=1 SV=4 - [SF3B3_HUMAN]	10,68	12	[illegible]
Q8TEW0	Partitioning defective 3 homolog OS=Homo sapiens GN=PARD3 PE=1 SV=2 - [PARD3_HUMAN]	10,55	12	[illegible]
Q14683	Structural maintenance of chromosomes protein 1A OS=Homo sapiens GN=SMC1A PE=1 SV=2 - [SMC1A_HUMAN]	10,54	12	[illegible]
O75970	Multiple PDZ domain protein OS=Homo sapiens GN=MPDZ PE=1 SV=2 - [MPDZ_HUMAN]	8,65	12	[illegible]
Q14690	Protein RRP5 homolog OS=Homo sapiens GN=PDCD11 PE=1 SV=3 - [RRP5_HUMAN]	8,28	12	[illegible]
O95425	Supervillin OS=Homo sapiens GN=SVIL PE=1 SV=2 - [SVIL_HUMAN]	7,09	12	[illegible]
Q9H583	HEAT repeat-containing protein 1 OS=Homo sapiens GN=HEATR1 PE=1 SV=3 - [HEAT1_HUMAN]	6,30	12	[illegible]
P55327-2	Isoform 2 of Tumor protein D52 OS=Homo sapiens GN=TPD52 - [TPD52_HUMAN]	76,09	11	[illegible]
Q99653	Calcium-binding protein p22 OS=Homo sapiens GN=CHP PE=1 SV=3 - [CHP1_HUMAN]	72,31	11	[illegible]
Q99497	Protein DJ-1 OS=Homo sapiens GN=PARK7 PE=1 SV=2 - [PARK7_HUMAN]	71,96	11	[illegible]
P61026	Ras-related protein Rab-10 OS=Homo sapiens GN=RAB10 PE=1 SV=1 - [RAB10_HUMAN]	67,00	11	[illegible]
P29692	Elongation factor 1-delta OS=Homo sapiens GN=EEF1D PE=1 SV=5 - [EF1D_HUMAN]	57,65	11	[illegible]
P13073	Cytochrome c oxidase subunit 4 isoform 1, mitochondrial OS=Homo sapiens GN=COX4I1 PE=1 SV=1 - [COX41_HUMAN]	56,21	11	[illegible]
Q9UL25	Ras-related protein Rab-21 OS=Homo sapiens GN=RAB21 PE=1 SV=3 - [RAB21_HUMAN]	55,56	11	[illegible]
P42167	Lamina-associated polypeptide 2, isoforms beta/gamma OS=Homo sapiens GN=TMPO PE=1 SV=2 - [LAP2B_HUMAN]	54,63	11	[illegible]
P51858	Hepatoma-derived growth factor OS=Homo sapiens GN=HDGF PE=1 SV=1 - [HDGF_HUMAN]	51,25	11	[illegible]
O96008	Mitochondrial import receptor subunit TOM40 homolog OS=Homo sapiens GN=TOMM40 PE=1 SV=1 - [TOM40_HUMAN]	50,42	11	[illegible]
P04792	Heat shock protein beta-1 OS=Homo sapiens GN=HSPB1 PE=1 SV=2 - [HSPB1_HUMAN]	49,27	11	[illegible]
P25786	Proteasome subunit alpha type-1 OS=Homo sapiens GN=PSMA1 PE=1 SV=1 - [PSA1_HUMAN]	48,67	11	[illegible]
Q9H936	Mitochondrial glutamate carrier 1 OS=Homo sapiens GN=SLC25A22 PE=1 SV=1 - [GHC1_HUMAN]	48,30	11	[illegible]
P62269	40S ribosomal protein S18 OS=Homo sapiens GN=RPS18 PE=1 SV=3 - [RS18_HUMAN]	48,03	11	[illegible]
O75489	NADH dehydrogenase [ubiquinone] iron-sulfur protein 3, mitochondrial OS=Homo sapiens GN=NDUFS3 PE=1 SV=1 - [NDUS3_HUMAN]	47,73	11	[illegible]
P20645	Cation-dependent mannose-6-phosphate receptor OS=Homo sapiens GN=M6PR PE=1 SV=1 - [MPRD_HUMAN]	45,49	11	[illegible]
P47985	Cytochrome b-c1 complex subunit Rieske, mitochondrial OS=Homo sapiens GN=UQCRFS1 PE=1 SV=2 - [UCRI_HUMAN]	44,89	11	[illegible]
P09110	3-ketoacyl-CoA thiolase, peroxisomal OS=Homo sapiens GN=ACAA1 PE=1 SV=2 - [THIK_HUMAN]	44,34	11	[illegible]
P26373	60S ribosomal protein L13 OS=Homo sapiens GN=RPL13 PE=1 SV=4 - [RL13_HUMAN]	43,13	11	[illegible]
Q9BWM7	Sideroflexin-3 OS=Homo sapiens GN=SFXN3 PE=2 SV=2 - [SFXN3_HUMAN]	42,15	11	[illegible]
P09651	Heterogeneous nuclear ribonucleoprotein A1 OS=Homo sapiens GN=HNRNPA1 PE=1 SV=5 - [ROA1_HUMAN]	41,94	11	[illegible]
P22087	rRNA 2'-O-methyltransferase fibrillarin OS=Homo sapiens GN=FBL PE=1 SV=2 - [FBRL_HUMAN]	41,43	11	[illegible]
Q9Y394	Dehydrogenase/reductase SDR family member 7 OS=Homo sapiens GN=DHRS7 PE=1 SV=1 - [DHRS7_HUMAN]	41,00	11	[illegible]
Q02978	Mitochondrial 2-oxoglutarate/malate carrier protein OS=Homo sapiens GN=SLC25A11 PE=1 SV=3 - [M2OM_HUMAN]	40,76	11	[illegible]
P46781	40S ribosomal protein S9 OS=Homo sapiens GN=RPS9 PE=1 SV=3 - [RS9_HUMAN]	40,72	11	[illegible]
O95210	Starch-binding domain-containing protein 1 OS=Homo sapiens GN=STBD1 PE=1 SV=1 - [STBD1_HUMAN]	40,50	11	[illegible]
Q7Z434	Mitochondrial antiviral-signaling protein OS=Homo sapiens GN=MAVS PE=1 SV=2 - [MAVS_HUMAN]	40,37	11	[illegible]
Q9BXK5	Bcl-2-like protein 13 OS=Homo sapiens GN=BCL2L13 PE=1 SV=1 - [B2L13_HUMAN]	39,18	11	[illegible]
P11802	Cyclin-dependent kinase 4 OS=Homo sapiens GN=CDK4 PE=1 SV=2 - [CDK4_HUMAN]	38,61	11	[illegible]
P30519	Heme oxygenase 2 OS=Homo sapiens GN=HMOX2 PE=1 SV=2 - [HMOX2_HUMAN]	38,61	11	[illegible]
P62333	26S protease regulatory subunit 10B OS=Homo sapiens GN=PSMC6 PE=1 SV=1 - [PRS10_HUMAN]	36,25	11	[illegible]
Q12907	Vesicular integral-membrane protein VIP36 OS=Homo sapiens GN=LMAN2 PE=1 SV=1 - [LMAN2_HUMAN]	35,67	11	[illegible]
Q15293	Reticulocalbin-1 OS=Homo sapiens GN=RCN1 PE=1 SV=1 - [RCN1_HUMAN]	34,44	11	[illegible]
Q9Y276	Mitochondrial chaperone BCS1 OS=Homo sapiens GN=BCS1L PE=1 SV=1 - [BCS1_HUMAN]	33,17	11	[illegible]
Q8TF74	WAS/WASL-interacting protein family member 2 OS=Homo sapiens GN=WIPF2 PE=1 SV=1 - [WIPF2_HUMAN]	32,50	11	[illegible]
P50454	Serpin H1 OS=Homo sapiens GN=SERPINH1 PE=1 SV=2 - [SERPH_HUMAN]	32,06	11	[illegible]
P13797	Plastin-3 OS=Homo sapiens GN=PLS3 PE=1 SV=4 - [PLST_HUMAN]	31,27	11	[illegible]
P36542	ATP synthase subunit gamma, mitochondrial OS=Homo sapiens GN=ATP5C1 PE=1 SV=1 - [ATPG_HUMAN]	31,21	11	[illegible]
Q96ER9	Coiled-coil domain-containing protein 51 OS=Homo sapiens GN=CCDC51 PE=1 SV=2 - [CCD51_HUMAN]	31,14	11	[illegible]
Q9UNM6	26S proteasome non-ATPase regulatory subunit 13 OS=Homo sapiens GN=PSMD13 PE=1 SV=2 - [PSD13_HUMAN]	30,59	11	[illegible]
P51991	Heterogeneous nuclear ribonucleoprotein A3 OS=Homo sapiens GN=HNRNPA3 PE=1 SV=2 - [ROA3_HUMAN]	29,63	11	[illegible]
Q8NBS9	Thioredoxin domain-containing protein 5 OS=Homo sapiens GN=TXNDC5 PE=1 SV=2 - [TXND5_HUMAN]	29,40	11	[illegible]
P14866	Heterogeneous nuclear ribonucleoprotein L OS=Homo sapiens GN=HNRNPL PE=1 SV=2 - [HNRPL_HUMAN]	28,69	11	[illegible]
Q13409-6	Isoform 2F of Cytoplasmic dynein 1 intermediate chain 2 OS=Homo sapiens GN=DYNC1I2 - [DC1I2_HUMAN]	28,64	11	[illegible]
Q9Y2W6	Tudor and KH domain-containing protein OS=Homo sapiens GN=TDRKH PE=1 SV=2 - [TDRKH_HUMAN]	28,34	11	[illegible]
P04181	Ornithine aminotransferase, mitochondrial OS=Homo sapiens GN=OAT PE=1 SV=1 - [OAT_HUMAN]	28,25	11	[illegible]
Q15942	Zyxin OS=Homo sapiens GN=ZYX PE=1 SV=1 - [ZYX_HUMAN]	27,27	11	[illegible]
P62495	Eukaryotic peptide chain release factor subunit 1 OS=Homo sapiens GN=ETF1 PE=1 SV=3 - [ERF1_HUMAN]	27,00	11	[illegible]
Q8TD30	Alanine aminotransferase 2 OS=Homo sapiens GN=GPT2 PE=1 SV=1 - [ALAT2_HUMAN]	26,96	11	[illegible]
Q9Y6M5	Zinc transporter 1 OS=Homo sapiens GN=SLC30A1 PE=1 SV=3 - [ZNT1_HUMAN]	26,43	11	[illegible]
Q8WVX9	Fatty acyl-CoA reductase 1 OS=Homo sapiens GN=FAR1 PE=1 SV=1 - [FACR1_HUMAN]	25,63	11	[illegible]
Q86Y07	Serine/threonine-protein kinase VRK2 OS=Homo sapiens GN=VRK2 PE=1 SV=3 - [VRK2_HUMAN]	25,00	11	[illegible]
Q9NZJ7	Mitochondrial carrier homolog 1 OS=Homo sapiens GN=MTCH1 PE=1 SV=1 - [MTCH1_HUMAN]	24,94	11	[illegible]
O75390	Citrate synthase, mitochondrial OS=Homo sapiens GN=CS PE=1 SV=2 - [CISY_HUMAN]	24,89	11	[illegible]
Q96DX4	RING finger and SPRY domain-containing protein 1 OS=Homo sapiens GN=RSPRY1 PE=2 SV=1 - [RSPRY_HUMAN]	24,83	11	[illegible]
P06744	Glucose-6-phosphate isomerase OS=Homo sapiens GN=GPI PE=1 SV=4 - [G6PI_HUMAN]	24,01	11	[illegible]
E9PAU2	Ribonucleoprotein PTB-binding 1 OS=Homo sapiens GN=RAVER1 PE=4 SV=1 - [E9PAU2_HUMAN]	23,81	11	[illegible]
Q96GQ5	UPF0420 protein C16orf58 OS=Homo sapiens GN=C16orf58 PE=1 SV=2 - [CP058_HUMAN]	23,72	11	[illegible]
Q9ULC5	Long-chain-fatty-acid--CoA ligase 5 OS=Homo sapiens GN=ACSL5 PE=1 SV=1 - [ACSL5_HUMAN]	23,57	11	[illegible]
Q9Y639	Neuroplastin OS=Homo sapiens GN=NPTN PE=1 SV=2 - [NPTN_HUMAN]	22,61	11	[illegible]
Q8N8S7	Protein enabled homolog OS=Homo sapiens GN=ENAH PE=1 SV=2 - [ENAH_HUMAN]	21,83	11	[illegible]
Q15833	Syntaxin-binding protein 2 OS=Homo sapiens GN=STXBP2 PE=1 SV=2 - [STXB2_HUMAN]	21,25	11	[illegible]
O75027	ATP-binding cassette sub-family B member 7, mitochondrial OS=Homo sapiens GN=ABCB7 PE=1 SV=2 - [ABCB7_HUMAN]	21,01	11	[illegible]
O75475	PC4 and SFRS1-interacting protein OS=Homo sapiens GN=PSIP1 PE=1 SV=1 - [PSIP1_HUMAN]	20,75	11	[illegible]
Q32MZ4-3	Isoform 3 of Leucine-rich repeat flightless-interacting protein 1 OS=Homo sapiens GN=LRRFIP1 - [LRRF1_HUMAN]	20,35	11	[illegible]
Q9UHD2	Serine/threonine-protein kinase TBK1 OS=Homo sapiens GN=TBK1 PE=1 SV=1 - [TBK1_HUMAN]	19,89	11	[illegible]
Q96PZ0	Pseudouridylate synthase 7 homolog OS=Homo sapiens GN=PUS7 PE=1 SV=2 - [PUS7_HUMAN]	19,82	11	[illegible]
P26639	Threonine--tRNA ligase, cytoplasmic OS=Homo sapiens GN=TARS PE=1 SV=3 - [SYTC_HUMAN]	19,50	11	[illegible]
Q99615	DnaJ homolog subfamily C member 7 OS=Homo sapiens GN=DNAJC7 PE=1 SV=2 - [DNJC7_HUMAN]	19,03	11	[illegible]
Q8NCG7	Sn1-specific diacylglycerol lipase beta OS=Homo sapiens GN=DAGLB PE=1 SV=2 - [DGLB_HUMAN]	18,60	11	[illegible]
Q15392	Delta(24)-sterol reductase OS=Homo sapiens GN=DHCR24 PE=1 SV=2 - [DHC24_HUMAN]	18,41	11	[illegible]
O60462	Neuropilin-2 OS=Homo sapiens GN=NRP2 PE=1 SV=2 - [NRP2_HUMAN]	18,15	11	[illegible]
P48634	Protein PRRC2A OS=Homo sapiens GN=PRRC2A PE=1 SV=3 - [PRC2A_HUMAN]	18,03	11	[illegible]
Q9BVJ6	U3 small nucleolar RNA-associated protein 14 homolog A OS=Homo sapiens GN=UTP14A PE=1 SV=1 - [UT14A_HUMAN]	17,64	11	[illegible]
Q6ZRP7	Sulfhydryl oxidase 2 OS=Homo sapiens GN=QSOX2 PE=1 SV=3 - [QSOX2_HUMAN]	16,76	11	[illegible]
P35606	Coatomer subunit beta' OS=Homo sapiens GN=COPB2 PE=1 SV=2 - [COPB2_HUMAN]	14,13	11	[illegible]
Q9BTX1	Nucleoporin NDC1 OS=Homo sapiens GN=TMEM48 PE=1 SV=2 - [NDC1_HUMAN]	13,80	11	[illegible]
P36776	Lon protease homolog, mitochondrial OS=Homo sapiens GN=LONP1 PE=1 SV=2 - [LONM_HUMAN]	13,14	11	[illegible]
Q8IY17	Neuropathy target esterase OS=Homo sapiens GN=PNPLA6 PE=1 SV=2 - [PLPL6_HUMAN]	12,30	11	[illegible]
P98196	Probable phospholipid-transporting ATPase IH OS=Homo sapiens GN=ATP11A PE=2 SV=3 - [AT11A_HUMAN]	11,11	11	[illegible]

P29144	Tripeptidyl-peptidase 2 OS=Homo sapiens GN=TPP2 PE=1 SV=4 - [TPP2_HUMAN]	10,25	11	0,964	[illegible]
Q92621	Nuclear pore complex protein Nup205 OS=Homo sapiens GN=NUP205 PE=1 SV=3 - [NU205_HUMAN]	8,70	11	0,905	[illegible]
P49750	YLP motif-containing protein 1 OS=Homo sapiens GN=YLPM1 PE=1 SV=3 - [YLPM1_HUMAN]	8,35	11	1,203	[illegible]
P18583-5	Isoform D of Protein SON OS=Homo sapiens GN=SON - [SON_HUMAN]	5,08	11		[illegible]
Q63HN8-4	Isoform 2 of E3 ubiquitin-protein ligase RNF213 OS=Homo sapiens GN=RNF213 - [RN213_HUMAN]	2,45	11	0,831	[illegible]
P30050	60S ribosomal protein L12 OS=Homo sapiens GN=RPL12 PE=1 SV=1 - [RL12_HUMAN]	72,12	10	0,893	[illegible]
P27348	14-3-3 protein theta OS=Homo sapiens GN=YWHAQ PE=1 SV=1 - [1433T_HUMAN]	65,31	10	0,763	[illegible]
Q06830	Peroxiredoxin-1 OS=Homo sapiens GN=PRDX1 PE=1 SV=1 - [PRDX1_HUMAN]	64,32	10	1,073	[illegible]
P07737	Profilin-1 OS=Homo sapiens GN=PFN1 PE=1 SV=2 - [PROF1_HUMAN]	62,86	10	0,615	[illegible]
P63104	14-3-3 protein zeta/delta OS=Homo sapiens GN=YWHAZ PE=1 SV=1 - [1433Z_HUMAN]	60,00	10	0,760	[illegible]
P30041	Peroxiredoxin-6 OS=Homo sapiens GN=PRDX6 PE=1 SV=3 - [PRDX6_HUMAN]	57,59	10	0,722	[illegible]
Q8N183	Mimitin, mitochondrial OS=Homo sapiens GN=NDUFAF2 PE=1 SV=1 - [MIMIT_HUMAN]	56,80	10	1,261	[illegible]
Q99714	3-hydroxyacyl-CoA dehydrogenase type-2 OS=Homo sapiens GN=HSD17B10 PE=1 SV=3 - [HCD2_HUMAN]	52,87	10	0,580	[illegible]
O95816	BAG family molecular chaperone regulator 2 OS=Homo sapiens GN=BAG2 PE=1 SV=1 - [BAG2_HUMAN]	47,39	10	1,820	[illegible]
P09493-3	Isoform 3 of Tropomyosin alpha-1 chain OS=Homo sapiens GN=TPM1 - [TPM1_HUMAN]	47,18	10	1,727	[illegible]
Q9P032	NADH dehydrogenase [ubiquinone] 1 alpha subcomplex assembly factor 4 OS=Homo sapiens GN=NDUFAF4 PE=1 SV=1 - [NDUF4_HUMA	46,29	10	1,194	[illegible]
P23284	Peptidyl-prolyl cis-trans isomerase B OS=Homo sapiens GN=PPIB PE=1 SV=2 - [PPIB_HUMAN]	44,91	10	0,991	[illegible]
P60842	Eukaryotic initiation factor 4A-I OS=Homo sapiens GN=EIF4A1 PE=1 SV=1 - [IF4A1_HUMAN]	44,83	10	0,895	[illegible]
Q15181	Inorganic pyrophosphatase OS=Homo sapiens GN=PPA1 PE=1 SV=2 - [IPYR_HUMAN]	44,64	10	0,493	[illegible]
P83731	60S ribosomal protein L24 OS=Homo sapiens GN=RPL24 PE=1 SV=1 - [RL24_HUMAN]	43,95	10	1,259	[illegible]
Q70UQ0	Inhibitor of nuclear factor kappa-B kinase-interacting protein OS=Homo sapiens GN=IKBIP PE=1 SV=1 - [IKIP_HUMAN]	42,86	10	1,724	[illegible]
O15173	Membrane-associated progesterone receptor component 2 OS=Homo sapiens GN=PGRMC2 PE=1 SV=1 - [PGRC2_HUMAN]	42,60	10	1,383	[illegible]
P19404	NADH dehydrogenase [ubiquinone] flavoprotein 2, mitochondrial OS=Homo sapiens GN=NDUFV2 PE=1 SV=2 - [NDUV2_HUMAN]	40,96	10	0,990	[illegible]
Q15005	Signal peptidase complex subunit 2 OS=Homo sapiens GN=SPCS2 PE=1 SV=3 - [SPCS2_HUMAN]	40,27	10	1,133	[illegible]
Q13190	Syntaxin-5 OS=Homo sapiens GN=STX5 PE=1 SV=2 - [STX5_HUMAN]	39,44	10	1,143	[illegible]
Q96C36	Pyrroline-5-carboxylate reductase 2 OS=Homo sapiens GN=PYCR2 PE=1 SV=1 - [P5CR2_HUMAN]	37,81	10	0,829	[illegible]
P14384	Carboxypeptidase M OS=Homo sapiens GN=CPM PE=1 SV=2 - [CBPM_HUMAN]	37,70	10	0,758	[illegible]
Q9BZG1	Ras-related protein Rab-34 OS=Homo sapiens GN=RAB34 PE=1 SV=1 - [RAB34_HUMAN]	37,45	10	0,988	[illegible]
Q9NX40	OCIA domain-containing protein 1 OS=Homo sapiens GN=OCIAD1 PE=1 SV=1 - [OCAD1_HUMAN]	37,14	10	1,207	[illegible]
P53701	Cytochrome c-type heme lyase OS=Homo sapiens GN=HCCS PE=1 SV=1 - [CCHL_HUMAN]	36,94	10	0,371	[illegible]
Q70UQ0-4	Isoform 4 of Inhibitor of nuclear factor kappa-B kinase-interacting protein OS=Homo sapiens GN=IKBIP - [IKIP_HUMAN]	35,28	10	**2,114**	[illegible]
Q9BRJ2	39S ribosomal protein L45, mitochondrial OS=Homo sapiens GN=MRPL45 PE=1 SV=2 - [RM45_HUMAN]	34,31	10	1,183	[illegible]
Q8NFQ8	Torsin-1A-interacting protein 2 OS=Homo sapiens GN=TOR1AIP2 PE=1 SV=1 - [TOIP2_HUMAN]	33,62	10	1,121	[illegible]
Q99848	Probable rRNA-processing protein EBP2 OS=Homo sapiens GN=EBNA1BP2 PE=1 SV=2 - [EBP2_HUMAN]	33,01	10	1,214	[illegible]
P80303	Nucleobindin-2 OS=Homo sapiens GN=NUCB2 PE=1 SV=2 - [NUCB2_HUMAN]	32,14	10	1,247	[illegible]
Q9HCM4-2	Isoform 2 of Band 4.1-like protein 5 OS=Homo sapiens GN=EPB41L5 - [E41L5_HUMAN]	32,08	10	0,893	[illegible]
P23381	Tryptophan--tRNA ligase, cytoplasmic OS=Homo sapiens GN=WARS PE=1 SV=2 - [SYWC_HUMAN]	31,63	10	0,544	[illegible]
P16422	Epithelial cell adhesion molecule OS=Homo sapiens GN=EPCAM PE=1 SV=2 - [EPCAM_HUMAN]	31,53	10	0,422	[illegible]
Q14108	Lysosome membrane protein 2 OS=Homo sapiens GN=SCARB2 PE=1 SV=2 - [SCRB2_HUMAN]	31,38	10	1,198	[illegible]
P20591	Interferon-induced GTP-binding protein Mx1 OS=Homo sapiens GN=MX1 PE=1 SV=4 - [MX1_HUMAN]	31,12	10	0,184	[illegible]
P08621	U1 small nuclear ribonucleoprotein 70 kDa OS=Homo sapiens GN=SNRNP70 PE=1 SV=2 - [RU17_HUMAN]	30,66	10	0,895	[illegible]
Q7L1Q6	Basic leucine zipper and W2 domain-containing protein 1 OS=Homo sapiens GN=BZW1 PE=1 SV=1 - [BZW1_HUMAN]	30,31	10	0,628	[illegible]
P82650	28S ribosomal protein S22, mitochondrial OS=Homo sapiens GN=MRPS22 PE=1 SV=1 - [RT22_HUMAN]	30,28	10	1,108	[illegible]
P37268	Squalene synthase OS=Homo sapiens GN=FDFT1 PE=1 SV=1 - [FDFT_HUMAN]	29,98	10	0,503	[illegible]
Q9NUL3	Double-stranded RNA-binding protein Staufen homolog 2 OS=Homo sapiens GN=STAU2 PE=1 SV=1 - [STAU2_HUMAN]	29,12	10	1,262	[illegible]
Q9BY67	Cell adhesion molecule 1 OS=Homo sapiens GN=CADM1 PE=1 SV=2 - [CADM1_HUMAN]	28,51	10	1,657	[illegible]
O60506	Heterogeneous nuclear ribonucleoprotein Q OS=Homo sapiens GN=SYNCRIP PE=1 SV=2 - [HNRPQ_HUMAN]	27,77	10	0,900	[illegible]
O75746	Calcium-binding mitochondrial carrier protein Aralar1 OS=Homo sapiens GN=SLC25A12 PE=1 SV=2 - [CMC1_HUMAN]	27,14	10	0,889	[illegible]
Q9Y617	Phosphoserine aminotransferase OS=Homo sapiens GN=PSAT1 PE=1 SV=2 - [SERC_HUMAN]	27,03	10	0,984	[illegible]
P12268	Inosine-5'-monophosphate dehydrogenase 2 OS=Homo sapiens GN=IMPDH2 PE=1 SV=2 - [IMDH2_HUMAN]	26,85	10	0,891	[illegible]
P13861	cAMP-dependent protein kinase type II-alpha regulatory subunit OS=Homo sapiens GN=PRKAR2A PE=1 SV=2 - [KAP2_HUMAN]	26,49	10	1,029	[illegible]
O00425	Insulin-like growth factor 2 mRNA-binding protein 3 OS=Homo sapiens GN=IGF2BP3 PE=1 SV=2 - [IF2B3_HUMAN]	26,42	10	0,833	[illegible]
Q08722	Leukocyte surface antigen CD47 OS=Homo sapiens GN=CD47 PE=1 SV=1 - [CD47_HUMAN]	26,32	10	1,117	[illegible]
P22234	Multifunctional protein ADE2 OS=Homo sapiens GN=PAICS PE=1 SV=3 - [PUR6_HUMAN]	26,12	10	0,891	[illegible]
O60884	DnaJ homolog subfamily A member 2 OS=Homo sapiens GN=DNAJA2 PE=1 SV=1 - [DNJA2_HUMAN]	25,97	10	1,044	[illegible]
Q9NZN4	EH domain-containing protein 2 OS=Homo sapiens GN=EHD2 PE=1 SV=2 - [EHD2_HUMAN]	25,60	10	1,550	[illegible]
P52209	6-phosphogluconate dehydrogenase, decarboxylating OS=Homo sapiens GN=PGD PE=1 SV=3 - [6PGD_HUMAN]	25,47	10	1,045	[illegible]
Q13177	Serine/threonine-protein kinase PAK 2 OS=Homo sapiens GN=PAK2 PE=1 SV=3 - [PAK2_HUMAN]	25,38	10	0,903	[illegible]
O43390	Heterogeneous nuclear ribonucleoprotein R OS=Homo sapiens GN=HNRNPR PE=1 SV=1 - [HNRPR_HUMAN]	25,12	10	0,904	[illegible]
P17812	CTP synthase 1 OS=Homo sapiens GN=CTPS PE=1 SV=2 - [PYRG1_HUMAN]	25,04	10	0,872	[illegible]
Q9HCC0	Methylcrotonoyl-CoA carboxylase beta chain, mitochondrial OS=Homo sapiens GN=MCCC2 PE=1 SV=1 - [MCCB_HUMAN]	24,87	10	0,942	[illegible]
Q9Y3I0	tRNA-splicing ligase RtcB homolog OS=Homo sapiens GN=C22orf28 PE=1 SV=1 - [RTCB_HUMAN]	24,75	10	0,881	[illegible]
Q16850	Lanosterol 14-alpha demethylase OS=Homo sapiens GN=CYP51A1 PE=1 SV=3 - [CP51A_HUMAN]	24,45	10	0,860	[illegible]
Q9NVH0;Q9NVH0-2	Exonuclease 3'-5' domain-containing protein 2 OS=Homo sapiens GN=EXD2 PE=1 SV=1 - [EXD2_HUMAN]	23,39	10	1,063	[illegible]
P83111	Serine beta-lactamase-like protein LACTB, mitochondrial OS=Homo sapiens GN=LACTB PE=1 SV=2 - [LACTB_HUMAN]	23,03	10	1,795	[illegible]
O95817	BAG family molecular chaperone regulator 3 OS=Homo sapiens GN=BAG3 PE=1 SV=3 - [BAG3_HUMAN]	22,96	10		[illegible]
O43493	Trans-Golgi network integral membrane protein 2 OS=Homo sapiens GN=TGOLN2 PE=1 SV=2 - [TGON2_HUMAN]	22,92	10	1,547	[illegible]
O14979	Heterogeneous nuclear ribonucleoprotein D-like OS=Homo sapiens GN=HNRPDL PE=1 SV=3 - [HNRDL_HUMAN]	22,86	10	1,117	[illegible]
Q9BRZ2	E3 ubiquitin-protein ligase TRIM56 OS=Homo sapiens GN=TRIM56 PE=1 SV=3 - [TRI56_HUMAN]	22,38	10	1,101	[illegible]
P14868	Aspartate--tRNA ligase, cytoplasmic OS=Homo sapiens GN=DARS PE=1 SV=2 - [SYDC_HUMAN]	22,36	10	0,719	[illegible]
Q9H3N1	Thioredoxin-related transmembrane protein 1 OS=Homo sapiens GN=TMX1 PE=1 SV=1 - [TMX1_HUMAN]	22,14	10	1,034	[illegible]
Q16625	Occludin OS=Homo sapiens GN=OCLN PE=1 SV=1 - [OCLN_HUMAN]	22,03	10	1,029	[illegible]
Q92841	Probable ATP-dependent RNA helicase DDX17 OS=Homo sapiens GN=DDX17 PE=1 SV=2 - [DDX17_HUMAN]	21,95	10	0,992	[illegible]
Q96TA1	Niban-like protein 1 OS=Homo sapiens GN=FAM129B PE=1 SV=3 - [NIBL1_HUMAN]	21,85	10	1,041	[illegible]
Q06124	Tyrosine-protein phosphatase non-receptor type 11 OS=Homo sapiens GN=PTPN11 PE=1 SV=2 - [PTN11_HUMAN]	21,78	10	0,758	[illegible]
P07686	Beta-hexosaminidase subunit beta OS=Homo sapiens GN=HEXB PE=1 SV=3 - [HEXB_HUMAN]	21,40	10	0,990	[illegible]
O95470	Sphingosine-1-phosphate lyase 1 OS=Homo sapiens GN=SGPL1 PE=1 SV=3 - [SGPL1_HUMAN]	21,30	10	0,753	[illegible]
P18084	Integrin beta-5 OS=Homo sapiens GN=ITGB5 PE=1 SV=1 - [ITB5_HUMAN]	20,90	10	1,508	[illegible]
O96005	Cleft lip and palate transmembrane protein 1 OS=Homo sapiens GN=CLPTM1 PE=1 SV=1 - [CLPT1_HUMAN]	20,63	10	1,147	[illegible]
Q15070	Mitochondrial inner membrane protein OXA1L OS=Homo sapiens GN=OXA1L PE=1 SV=3 - [OXA1L_HUMAN]	20,00	10	1,314	[illegible]
Q93052	Lipoma-preferred partner OS=Homo sapiens GN=LPP PE=1 SV=1 - [LPP_HUMAN]	19,77	10		[illegible]
Q03169	Tumor necrosis factor alpha-induced protein 2 OS=Homo sapiens GN=TNFAIP2 PE=1 SV=2 - [TNAP2_HUMAN]	19,72	10	1,284	[illegible]
P46060	Ran GTPase-activating protein 1 OS=Homo sapiens GN=RANGAP1 PE=1 SV=1 - [RAGP1_HUMAN]	19,59	10	0,821	[illegible]
P53985	Monocarboxylate transporter 1 OS=Homo sapiens GN=SLC16A1 PE=1 SV=3 - [MOT1_HUMAN]	19,20	10	1,731	[illegible]
Q9HDC5	Junctophilin-1 OS=Homo sapiens GN=JPH1 PE=1 SV=2 - [JPH1_HUMAN]	19,06	10	1,100	[illegible]
P22223	Cadherin-3 OS=Homo sapiens GN=CDH3 PE=1 SV=2 - [CADH3_HUMAN]	18,82	10	0,421	[illegible]
Q96KC8	DnaJ homolog subfamily C member 1 OS=Homo sapiens GN=DNAJC1 PE=1 SV=1 - [DNJC1_HUMAN]	18,59	10	0,894	[illegible]
Q9UBV2	Protein sel-1 homolog 1 OS=Homo sapiens GN=SEL1L PE=1 SV=3 - [SE1L1_HUMAN]	18,51	10	0,932	[illegible]
Q99460	26S proteasome non-ATPase regulatory subunit 1 OS=Homo sapiens GN=PSMD1 PE=1 SV=2 - [PSMD1_HUMAN]	17,52	10	0,825	[illegible]
Q9NP58	ATP-binding cassette sub-family B member 6, mitochondrial OS=Homo sapiens GN=ABCB6 PE=1 SV=1 - [ABCB6_HUMAN]	16,98	10	1,188	[illegible]
Q9NSD9	Phenylalanine--tRNA ligase beta subunit OS=Homo sapiens GN=FARSB PE=1 SV=3 - [SYFB_HUMAN]	16,64	10	0,688	[illegible]
P35610	Sterol O-acyltransferase 1 OS=Homo sapiens GN=SOAT1 PE=1 SV=3 - [SOAT1_HUMAN]	16,18	10	1,702	[illegible]
Q96PY5	Formin-like protein 2 OS=Homo sapiens GN=FMNL2 PE=1 SV=3 - [FMNL2_HUMAN]	16,02	10	1,380	[illegible]
Q6PJF5-2	Isoform 2 of Inactive rhomboid protein 2 OS=Homo sapiens GN=RHBDF2 - [RHDF2_HUMAN]	15,96	10	1,293	[illegible]
Q6UB35	Monofunctional C1-tetrahydrofolate synthase, mitochondrial OS=Homo sapiens GN=MTHFD1L PE=1 SV=1 - [C1TM_HUMAN]	15,64	10	0,877	[illegible]
Q96QC0	Serine/threonine-protein phosphatase 1 regulatory subunit 10 OS=Homo sapiens GN=PPP1R10 PE=1 SV=1 - [PP1RA_HUMAN]	15,53	10		[illegible]
Q9H307	Pinin OS=Homo sapiens GN=PNN PE=1 SV=4 - [PININ_HUMAN]	15,06	10		[illegible]
O94973	AP-2 complex subunit alpha-2 OS=Homo sapiens GN=AP2A2 PE=1 SV=2 - [AP2A2_HUMAN]	15,02	10	0,928	[illegible]
Q9ULF5	Zinc transporter ZIP10 OS=Homo sapiens GN=SLC39A10 PE=1 SV=2 - [S39AA_HUMAN]	14,56	10	1,435	[illegible]
P53618	Coatomer subunit beta OS=Homo sapiens GN=COPB1 PE=1 SV=3 - [COPB_HUMAN]	14,48	10	0,804	[illegible]
Q16666	Gamma-interferon-inducible protein 16 OS=Homo sapiens GN=IFI16 PE=1 SV=3 - [IF16_HUMAN]	14,01	10	0,434	[illegible]
O95373	Importin-7 OS=Homo sapiens GN=IPO7 PE=1 SV=1 - [IPO7_HUMAN]	13,97	10	0,758	[illegible]
P42566	Epidermal growth factor receptor substrate 15 OS=Homo sapiens GN=EPS15 PE=1 SV=2 - [EPS15_HUMAN]	13,95	10	0,887	[illegible]
Q9BZE4	Nucleolar GTP-binding protein 1 OS=Homo sapiens GN=GTPBP4 PE=1 SV=3 - [NOG1_HUMAN]	13,88	10	1,014	[illegible]
Q7Z2W4	Zinc finger CCCH-type antiviral protein 1 OS=Homo sapiens GN=ZC3HAV1 PE=1 SV=3 - [ZCCHV_HUMAN]	13,75	10	0,856	[illegible]
P40189	Interleukin-6 receptor subunit beta OS=Homo sapiens GN=IL6ST PE=1 SV=2 - [IL6RB_HUMAN]	13,18	10	1,437	[illegible]
Q01780	Exosome component 10 OS=Homo sapiens GN=EXOSC10 PE=1 SV=2 - [EXOSX_HUMAN]	12,32	10		[illegible]
Q9BUJ2	Heterogeneous nuclear ribonucleoprotein U-like protein 1 OS=Homo sapiens GN=HNRNPUL1 PE=1 SV=2 - [HNRL1_HUMAN]	12,27	10	1,092	[illegible]
O75146	Huntingtin-interacting protein 1-related protein OS=Homo sapiens GN=HIP1R PE=1 SV=2 - [HIP1R_HUMAN]	12,27	10	0,878	[illegible]
O14980	Exportin-1 OS=Homo sapiens GN=XPO1 PE=1 SV=1 - [XPO1_HUMAN]	12,14	10	0,706	[illegible]
Q9UP95	Solute carrier family 12 member 4 OS=Homo sapiens GN=SLC12A4 PE=1 SV=2 - [S12A4_HUMAN]	11,98	10	1,237	[illegible]
Q9BXP5	Serrate RNA effector molecule homolog OS=Homo sapiens GN=SRRT PE=1 SV=1 - [SRRT_HUMAN]	11,87	10		[illegible]
Q16531	DNA damage-binding protein 1 OS=Homo sapiens GN=DDB1 PE=1 SV=1 - [DDB1_HUMAN]	11,75	10	0,895	[illegible]
Q6PKG0	La-related protein 1 OS=Homo sapiens GN=LARP1 PE=1 SV=2 - [LARP1_HUMAN]	10,77	10		[illegible]
E7ERS3	Zinc finger CCCH domain-containing protein 18 OS=Homo sapiens GN=ZC3H18 PE=4 SV=1 - [E7ERS3_HUMAN]	10,64	10		[illegible]
P35251	Replication factor C subunit 1 OS=Homo sapiens GN=RFC1 PE=1 SV=4 - [RFC1_HUMAN]	9,93	10	0,735	[illegible]
Q9Y5B9	FACT complex subunit SPT16 OS=Homo sapiens GN=SUPT16H PE=1 SV=1 - [SP16H_HUMAN]	9,93	10		[illegible]
Q29RF7	Sister chromatid cohesion protein PDS5 homolog A OS=Homo sapiens GN=PDS5A PE=1 SV=1 - [PDS5A_HUMAN]	9,87	10	0,785	[illegible]
O60610	Protein diaphanous homolog 1 OS=Homo sapiens GN=DIAPH1 PE=1 SV=2 - [DIAP1_HUMAN]	9,36	10	0,753	[illegible]
Q8IX12	Cell division cycle and apoptosis regulator protein 1 OS=Homo sapiens GN=CCAR1 PE=1 SV=2 - [CCAR1_HUMAN]	9,30	10		[illegible]
P24928	DNA-directed RNA polymerase II subunit RPB1 OS=Homo sapiens GN=POLR2A PE=1 SV=2 - [RPB1_HUMAN]	7,66	10		[illegible]
P26358-2	Isoform 2 of DNA (cytosine-5)-methyltransferase 1 OS=Homo sapiens GN=DNMT1 - [DNMT1_HUMAN]	7,54	10	1,090	[illegible]
Q5VYK3	Proteasome-associated protein ECM29 homolog OS=Homo sapiens GN=ECM29 PE=1 SV=2 - [ECM29_HUMAN]	7,05	10	0,845	[illegible]
Q8NF91	Nesprin-1 OS=Homo sapiens GN=SYNE1 PE=1 SV=3 - [SYNE1_HUMAN]	1,61	10	0,994	[illegible]
P09382	Galectin-1 OS=Homo sapiens GN=LGALS1 PE=1 SV=2 - [LEG1_HUMAN]	77,78	9	1,327	[illegible]
P16403	Histone H1.2 OS=Homo sapiens GN=HIST1H1C PE=1 SV=2 - [H12_HUMAN]	64,79	9	1,221	[illegible]
O00161	Synaptosomal-associated protein 23 OS=Homo sapiens GN=SNAP23 PE=1 SV=1 - [SNP23_HUMAN]	56,87	9	1,359	[illegible]
Q9Y2B0	Protein canopy homolog 2 OS=Homo sapiens GN=CNPY2 PE=1 SV=1 - [CNPY2_HUMAN]	56,59	9	1,157	[illegible]
P62266	40S ribosomal protein S23 OS=Homo sapiens GN=RPS23 PE=1 SV=3 - [RS23_HUMAN]	55,24	9	0,977	[illegible]
Q5T9A4	ATPase family AAA domain-containing protein 3B OS=Homo sapiens GN=ATAD3B PE=1 SV=1 - [ATD3B_HUMAN]	53,40	9	0,704	[illegible]
P63241	Eukaryotic translation initiation factor 5A-1 OS=Homo sapiens GN=EIF5A PE=1 SV=2 - [IF5A1_HUMAN]	53,25	9	1,101	[illegible]
P46783	40S ribosomal protein S10 OS=Homo sapiens GN=RPS10 PE=1 SV=1 - [RS10_HUMAN]	50,91	9	0,947	[illegible]
Q04917	14-3-3 protein eta OS=Homo sapiens GN=YWHAH PE=1 SV=4 - [1433F_HUMAN]	49,19	9	1,214	[illegible]
O60869	Endothelial differentiation-related factor 1 OS=Homo sapiens GN=EDF1 PE=1 SV=1 - [EDF1_HUMAN]	46,62	9	1,072	[illegible]
O15400	Syntaxin-7 OS=Homo sapiens GN=STX7 PE=1 SV=4 - [STX7_HUMAN]	45,59	9	1,494	[illegible]
P62280	40S ribosomal protein S11 OS=Homo sapiens GN=RPS11 PE=1 SV=3 - [RS11_HUMAN]	45,57	9	0,842	[illegible]
P20340	Ras-related protein Rab-6A OS=Homo sapiens GN=RAB6A PE=1 SV=3 - [RAB6A_HUMAN]	44,23	9	0,958	[illegible]
Q96A35	39S ribosomal protein L24, mitochondrial OS=Homo sapiens GN=MRPL24 PE=1 SV=1 - [RM24_HUMAN]	43,06	9	1,098	[illegible]
P27144	Adenylate kinase isoenzyme 4, mitochondrial OS=Homo sapiens GN=AK4 PE=1 SV=1 - [KAD4_HUMAN]	41,26	9	0,955	[illegible]
Q15165	Serum paraoxonase/arylesterase 2 OS=Homo sapiens GN=PON2 PE=1 SV=3 - [PON2_HUMAN]	41,24	9	1,185	[illegible]
P62158	Calmodulin OS=Homo sapiens GN=CALM1 PE=1 SV=2 - [CALM_HUMAN]	40,27	9	1,384	[illegible]
P09758	Tumor-associated calcium signal transducer 2 OS=Homo sapiens GN=TACSTD2 PE=1 SV=3 - [TACD2_HUMAN]	39,32	9	0,577	[illegible]
P39019	40S ribosomal protein S19 OS=Homo sapiens GN=RPS19 PE=1 SV=2 - [RS19_HUMAN]	39,31	9	0,662	[illegible]
Q9UL46	Proteasome activator complex subunit 2 OS=Homo sapiens GN=PSME2 PE=1 SV=4 - [PSME2_HUMAN]	38,91	9	0,558	[illegible]
Q9NUQ9	Protein FAM49B OS=Homo sapiens GN=FAM49B PE=1 SV=1 - [FA49B_HUMAN]	38,27	9	1,366	[illegible]
Q9HAV7	GrpE protein homolog 1, mitochondrial OS=Homo sapiens GN=GRPEL1 PE=1 SV=2 - [GRPE1_HUMAN]	37,79	9	0,819	[illegible]
Q9UNK0	Syntaxin-8 OS=Homo sapiens GN=STX8 PE=1 SV=2 - [STX8_HUMAN]	37,71	9	1,026	[illegible]
P24539	ATP synthase subunit b, mitochondrial OS=Homo sapiens GN=ATP5F1 PE=1 SV=2 - [AT5F1_HUMAN]	37,50	9	1,500	[illegible]
Q9GZT6	Coiled-coil domain-containing protein 90B, mitochondrial OS=Homo sapiens GN=CCDC90B PE=1 SV=2 - [CC90B_HUMAN]	37,01	9	1,514	[illegible]
Q9Y673	Dolichyl-phosphate beta-glucosyltransferase OS=Homo sapiens GN=ALG5 PE=1 SV=1 - [ALG5_HUMAN]	36,42	9	1,018	[illegible]
Q9Y5M8	Signal recognition particle receptor subunit beta OS=Homo sapiens GN=SRPRB PE=1 SV=3 - [SRPRB_HUMAN]	35,42	9	0,935	[illegible]
Q96HY6	DDRGK domain-containing protein 1 OS=Homo sapiens GN=DDRGK1 PE=1 SV=2 - [DDRGK_HUMAN]	35,35	9	1,199	[illegible]
Q15599	Na(+)/H(+) exchange regulatory cofactor NHE-RF2 OS=Homo sapiens GN=SLC9A3R2 PE=1 SV=2 - [NHRF2_HUMAN]	34,72	9	0,597	[illegible]
Q15717	ELAV-like protein 1 OS=Homo sapiens GN=ELAVL1 PE=1 SV=2 - [ELAV1_HUMAN]	34,05	9	0,988	[illegible]
Q99536	Synaptic vesicle membrane protein VAT-1 homolog OS=Homo sapiens GN=VAT1 PE=1 SV=2 - [VAT1_HUMAN]	33,33	9	1,533	[illegible]
Q07666	KH domain-containing, RNA-binding, signal transduction-associated protein 1 OS=Homo sapiens GN=KHDRBS1 PE=1 SV=1 - [KHDR1_HU	33,18	9		[illegible]
Q15019	Septin-2 OS=Homo sapiens GN=SEPT2 PE=1 SV=1 - [SEPT2_HUMAN]	32,96	9	0,899	[illegible]
Q13347	Eukaryotic translation initiation factor 3 subunit I OS=Homo sapiens GN=EIF3I PE=1 SV=1 - [EIF3I_HUMAN]	32,92	9	0,884	[illegible]
Q53GQ0	Estradiol 17-beta-dehydrogenase 12 OS=Homo sapiens GN=HSD17B12 PE=1 SV=2 - [DHB12_HUMAN]	32,69	9	0,743	[illegible]
Q12905	Interleukin enhancer-binding factor 2 OS=Homo sapiens GN=ILF2 PE=1 SV=2 - [ILF2_HUMAN]	32,31	9	0,828	[illegible]
P53007	Tricarboxylate transport protein, mitochondrial OS=Homo sapiens GN=SLC25A1 PE=1 SV=2 - [TXTP_HUMAN]	32,15	9	1,048	[illegible]
Q9HBH5	Retinol dehydrogenase 14 OS=Homo sapiens GN=RDH14 PE=1 SV=1 - [RDH14_HUMAN]	32,14	9	0,962	[illegible]
Q13423	NAD(P) transhydrogenase, mitochondrial OS=Homo sapiens GN=NNT PE=1 SV=3 - [NNTM_HUMAN]	32,14	9	1,396	[illegible]
P18754	Regulator of chromosome condensation OS=Homo sapiens GN=RCC1 PE=1 SV=1 - [RCC1_HUMAN]	31,83	9	0,928	[illegible]
Q13501	Sequestosome-1 OS=Homo sapiens GN=SQSTM1 PE=1 SV=1 - [SQSTM_HUMAN]	31,82	9	1,593	[illegible]
Q9Y6I9	Testis-expressed sequence 264 protein OS=Homo sapiens GN=TEX264 PE=1 SV=1 - [TX264_HUMAN]	31,63	9	1,208	[illegible]
Q9P2W9	Syntaxin-18 OS=Homo sapiens GN=STX18 PE=1 SV=1 - [STX18_HUMAN]	31,04	9	1,175	[illegible]
Q6IAN0	Dehydrogenase/reductase SDR family member 7B OS=Homo sapiens GN=DHRS7B PE=1 SV=2 - [DRS7B_HUMAN]	30,77	9	0,840	[illegible]
Q9Y3F4	Serine-threonine kinase receptor-associated protein OS=Homo sapiens GN=STRAP PE=1 SV=1 - [STRAP_HUMAN]	30,57	9	0,827	[illegible]
Q8NBU5	ATPase family AAA domain-containing protein 1 OS=Homo sapiens GN=ATAD1 PE=1 SV=1 - [ATAD1_HUMAN]	30,47	9	1,052	[illegible]

P62195	26S protease regulatory subunit 8 OS=Homo sapiens GN=PSMC5 PE=1 SV=1 - [PRS8_HUMAN]	30,05
P17931	Galectin-3 OS=Homo sapiens GN=LGALS3 PE=1 SV=5 - [LEG3_HUMAN]	30,00
Q9BY77	Polymerase delta-interacting protein 3 OS=Homo sapiens GN=POLDIP3 PE=1 SV=2 - [PDIP3_HUMAN]	29,69
Q92665	28S ribosomal protein S31, mitochondrial OS=Homo sapiens GN=MRPS31 PE=1 SV=3 - [RT31_HUMAN]	29,62
P35241	Radixin OS=Homo sapiens GN=RDX PE=1 SV=1 - [RADI_HUMAN]	29,50
Q9Y320	Thioredoxin-related transmembrane protein 2 OS=Homo sapiens GN=TMX2 PE=1 SV=1 - [TMX2_HUMAN]	29,39
Q9P035	3-hydroxyacyl-CoA dehydratase 3 OS=Homo sapiens GN=PTPLAD1 PE=1 SV=2 - [HACD3_HUMAN]	29,28
F8W9Y0	Syntaxin-3 OS=Homo sapiens GN=STX3 PE=3 SV=1 - [F8W9Y0_HUMAN]	29,07
P05091	Aldehyde dehydrogenase, mitochondrial OS=Homo sapiens GN=ALDH2 PE=1 SV=2 - [ALDH2_HUMAN]	29,01
P52799	Ephrin-B2 OS=Homo sapiens GN=EFNB2 PE=1 SV=1 - [EFNB2_HUMAN]	27,93
P17980	26S protease regulatory subunit 6A OS=Homo sapiens GN=PSMC3 PE=1 SV=3 - [PRS6A_HUMAN]	27,79
Q07960	Rho GTPase-activating protein 1 OS=Homo sapiens GN=ARHGAP1 PE=1 SV=1 - [RHG01_HUMAN]	27,56
P48509	CD151 antigen OS=Homo sapiens GN=CD151 PE=1 SV=3 - [CD151_HUMAN]	26,88
P30153	Serine/threonine-protein phosphatase 2A 65 kDa regulatory subunit A alpha isoform OS=Homo sapiens GN=PPP2R1A PE=1 SV=4 - [2AA	26,83
Q6YN16	Hydroxysteroid dehydrogenase-like protein 2 OS=Homo sapiens GN=HSDL2 PE=1 SV=1 - [HSDL2_HUMAN]	26,56
P30837	Aldehyde dehydrogenase X, mitochondrial OS=Homo sapiens GN=ALDH1B1 PE=1 SV=3 - [AL1B1_HUMAN]	26,11
P07947	Tyrosine-protein kinase Yes OS=Homo sapiens GN=YES1 PE=1 SV=3 - [YES_HUMAN]	25,78
Q96I24	Far upstream element-binding protein 3 OS=Homo sapiens GN=FUBP3 PE=1 SV=2 - [FUBP3_HUMAN]	25,17
Q9BT22	Chitobiosyldiphosphodolichol beta-mannosyltransferase OS=Homo sapiens GN=ALG1 PE=1 SV=2 - [ALG1_HUMAN]	24,78
P15529	Membrane cofactor protein OS=Homo sapiens GN=CD46 PE=1 SV=3 - [MCP_HUMAN]	24,74
Q9BWF3	RNA-binding protein 4 OS=Homo sapiens GN=RBM4 PE=1 SV=1 - [RBM4_HUMAN]	24,73
Q92688	Acidic leucine-rich nuclear phosphoprotein 32 family member B OS=Homo sapiens GN=ANP32B PE=1 SV=1 - [AN32B_HUMAN]	24,70
Q9GZS1-2	Isoform 2 of DNA-directed RNA polymerase I subunit RPA49 OS=Homo sapiens GN=POLR1E - [RPA49_HUMAN]	23,87
Q9NZ01	Trans-2,3-enoyl-CoA reductase OS=Homo sapiens GN=TECR PE=1 SV=1 - [TECR_HUMAN]	23,38
P13489	Ribonuclease inhibitor OS=Homo sapiens GN=RNH1 PE=1 SV=2 - [RINI_HUMAN]	23,21
Q96JJ7	Protein disulfide-isomerase TMX3 OS=Homo sapiens GN=TMX3 PE=1 SV=2 - [TMX3_HUMAN]	23,13
P54727	UV excision repair protein RAD23 homolog B OS=Homo sapiens GN=RAD23B PE=1 SV=1 - [RD23B_HUMAN]	22,98
P36957	Dihydrolipoyllysine-residue succinyltransferase component of 2-oxoglutarate dehydrogenase complex, mitochondrial OS=Homo sapiens G	22,96
Q9H223	EH domain-containing protein 4 OS=Homo sapiens GN=EHD4 PE=1 SV=1 - [EHD4_HUMAN]	22,74
O14763	Tumor necrosis factor receptor superfamily member 10B OS=Homo sapiens GN=TNFRSF10B PE=1 SV=2 - [TR10B_HUMAN]	22,05
Q8WVM8	Sec1 family domain-containing protein 1 OS=Homo sapiens GN=SCFD1 PE=1 SV=4 - [SCFD1_HUMAN]	21,50
Q9NRW7	Vacuolar protein sorting-associated protein 45 OS=Homo sapiens GN=VPS45 PE=1 SV=1 - [VPS45_HUMAN]	20,70
Q9UH99	SUN domain-containing protein 2 OS=Homo sapiens GN=SUN2 PE=1 SV=3 - [SUN2_HUMAN]	20,64
Q6IC98	GRAM domain-containing protein 4 OS=Homo sapiens GN=GRAMD4 PE=1 SV=1 - [GRAM4_HUMAN]	20,07
P28799	Granulins OS=Homo sapiens GN=GRN PE=1 SV=2 - [GRN_HUMAN]	20,07
Q86X29	Lipolysis-stimulated lipoprotein receptor OS=Homo sapiens GN=LSR PE=1 SV=4 - [LSR_HUMAN]	19,88
Q13425	Beta-2-syntrophin OS=Homo sapiens GN=SNTB2 PE=1 SV=1 - [SNTB2_HUMAN]	19,81
Q8WZA1	Protein O-linked-mannose beta-1,2-N-acetylglucosaminyltransferase 1 OS=Homo sapiens GN=POMGNT1 PE=1 SV=2 - [PMGT1_HUMAN]	19,70
Q96S52	GPI transamidase component PIG-S OS=Homo sapiens GN=PIGS PE=1 SV=3 - [PIGS_HUMAN]	18,92
Q9P258	Protein RCC2 OS=Homo sapiens GN=RCC2 PE=1 SV=2 - [RCC2_HUMAN]	18,77
O00429	Dynamin-1-like protein OS=Homo sapiens GN=DNM1L PE=1 SV=2 - [DNM1L_HUMAN]	18,34
O43776	Asparagine--tRNA ligase, cytoplasmic OS=Homo sapiens GN=NARS PE=1 SV=1 - [SYNC_HUMAN]	18,07
P61619	Protein transport protein Sec61 subunit alpha isoform 1 OS=Homo sapiens GN=SEC61A1 PE=1 SV=2 - [S61A1_HUMAN]	17,86
Q13573	SNW domain-containing protein 1 OS=Homo sapiens GN=SNW1 PE=1 SV=1 - [SNW1_HUMAN]	17,72
O76031	ATP-dependent Clp protease ATP-binding subunit clpX-like, mitochondrial OS=Homo sapiens GN=CLPX PE=1 SV=2 - [CLPX_HUMAN]	17,22
Q16822	Phosphoenolpyruvate carboxykinase [GTP], mitochondrial OS=Homo sapiens GN=PCK2 PE=1 SV=3 - [PCKGM_HUMAN]	17,19
Q16643	Drebrin OS=Homo sapiens GN=DBN1 PE=1 SV=4 - [DREB_HUMAN]	17,10
Q9Y5Y6	Suppressor of tumorigenicity 14 protein OS=Homo sapiens GN=ST14 PE=1 SV=2 - [ST14_HUMAN]	17,08
Q8NCL4	Polypeptide N-acetylgalactosaminyltransferase 6 OS=Homo sapiens GN=GALNT6 PE=2 SV=2 - [GALT6_HUMAN]	16,88
Q6NZY4	Zinc finger CCHC domain-containing protein 8 OS=Homo sapiens GN=ZCCHC8 PE=1 SV=2 - [ZCHC8_HUMAN]	16,69
Q14151	Scaffold attachment factor B2 OS=Homo sapiens GN=SAFB2 PE=1 SV=1 - [SAFB2_HUMAN]	16,58
O60476	Mannosyl-oligosaccharide 1,2-alpha-mannosidase IB OS=Homo sapiens GN=MAN1A2 PE=2 SV=1 - [MA1A2_HUMAN]	16,54
P30038	Delta-1-pyrroline-5-carboxylate dehydrogenase, mitochondrial OS=Homo sapiens GN=ALDH4A1 PE=1 SV=3 - [AL4A1_HUMAN]	16,52
Q14156	Protein EFR3 homolog A OS=Homo sapiens GN=EFR3A PE=1 SV=2 - [EFR3A_HUMAN]	15,83
Q969N2	GPI transamidase component PIG-T OS=Homo sapiens GN=PIGT PE=1 SV=1 - [PIGT_HUMAN]	15,57
Q8N4S9	MARVEL domain-containing protein 2 OS=Homo sapiens GN=MARVELD2 PE=1 SV=2 - [MALD2_HUMAN]	15,41
P55884	Eukaryotic translation initiation factor 3 subunit B OS=Homo sapiens GN=EIF3B PE=1 SV=3 - [EIF3B_HUMAN]	14,74
Q9UQ90	Paraplegin OS=Homo sapiens GN=SPG7 PE=1 SV=2 - [SPG7_HUMAN]	14,34
P17480	Nucleolar transcription factor 1 OS=Homo sapiens GN=UBTF PE=1 SV=1 - [UBF1_HUMAN]	13,74
Q8IY81	Putative rRNA methyltransferase 3 OS=Homo sapiens GN=FTSJ3 PE=1 SV=2 - [RRMJ3_HUMAN]	13,70
O14939	Phospholipase D2 OS=Homo sapiens GN=PLD2 PE=1 SV=2 - [PLD2_HUMAN]	13,40
Q86XP3	ATP-dependent RNA helicase DDX42 OS=Homo sapiens GN=DDX42 PE=1 SV=1 - [DDX42_HUMAN]	12,47
O75152	Zinc finger CCCH domain-containing protein 11A OS=Homo sapiens GN=ZC3H11A PE=1 SV=3 - [ZC11A_HUMAN]	12,47
P46379	Large proline-rich protein BAG6 OS=Homo sapiens GN=BAG6 PE=1 SV=2 - [BAG6_HUMAN]	12,10
Q8N1F7	Nuclear pore complex protein Nup93 OS=Homo sapiens GN=NUP93 PE=1 SV=2 - [NUP93_HUMAN]	11,97
O75400	Pre-mRNA-processing factor 40 homolog A OS=Homo sapiens GN=PRPF40A PE=1 SV=2 - [PR40A_HUMAN]	11,81
Q5T6F2	Ubiquitin-associated protein 2 OS=Homo sapiens GN=UBAP2 PE=1 SV=1 - [UBAP2_HUMAN]	11,62
Q9NYF8	Bcl-2-associated transcription factor 1 OS=Homo sapiens GN=BCLAF1 PE=1 SV=2 - [BCLF1_HUMAN]	11,52
O95782	AP-2 complex subunit alpha-1 OS=Homo sapiens GN=AP2A1 PE=1 SV=3 - [AP2A1_HUMAN]	11,46
Q9HC35	Echinoderm microtubule-associated protein-like 4 OS=Homo sapiens GN=EML4 PE=1 SV=3 - [EMAL4_HUMAN]	11,42
Q8IWA5	Choline transporter-like protein 2 OS=Homo sapiens GN=SLC44A2 PE=1 SV=3 - [CTL2_HUMAN]	11,19
Q99613	Eukaryotic translation initiation factor 3 subunit C OS=Homo sapiens GN=EIF3C PE=1 SV=1 - [EIF3C_HUMAN]	11,17
Q12965	Unconventional myosin-Ie OS=Homo sapiens GN=MYO1E PE=1 SV=2 - [MYO1E_HUMAN]	10,92
Q8WUM0	Nuclear pore complex protein Nup133 OS=Homo sapiens GN=NUP133 PE=1 SV=2 - [NU133_HUMAN]	10,21
Q15648	Mediator of RNA polymerase II transcription subunit 1 OS=Homo sapiens GN=MED1 PE=1 SV=4 - [MED1_HUMAN]	10,12
Q6ZXV5	Transmembrane and TPR repeat-containing protein 3 OS=Homo sapiens GN=TMTC3 PE=1 SV=2 - [TMTC3_HUMAN]	9,95
Q96JH7	Deubiquitinating protein VCIP135 OS=Homo sapiens GN=VCPIP1 PE=1 SV=2 - [VCIP1_HUMAN]	9,74
Q9BXJ9	N-alpha-acetyltransferase 15, NatA auxiliary subunit OS=Homo sapiens GN=NAA15 PE=1 SV=1 - [NAA15_HUMAN]	9,70
Q8NEN9	PDZ domain-containing protein 8 OS=Homo sapiens GN=PDZD8 PE=1 SV=1 - [PDZD8_HUMAN]	9,45
Q9H0A0	N-acetyltransferase 10 OS=Homo sapiens GN=NAT10 PE=1 SV=2 - [NAT10_HUMAN]	9,37
O60264	SWI/SNF-related matrix-associated actin-dependent regulator of chromatin subfamily A member 5 OS=Homo sapiens GN=SMARCA5 PE=	8,94
Q14139	Ubiquitin conjugation factor E4 A OS=Homo sapiens GN=UBE4A PE=1 SV=2 - [UBE4A_HUMAN]	8,72
Q14839	Chromodomain-helicase-DNA-binding protein 4 OS=Homo sapiens GN=CHD4 PE=1 SV=2 - [CHD4_HUMAN]	8,53
O75694	Nuclear pore complex protein Nup155 OS=Homo sapiens GN=NUP155 PE=1 SV=1 - [NU155_HUMAN]	8,34
Q8NI35	InaD-like protein OS=Homo sapiens GN=INADL PE=1 SV=3 - [INADL_HUMAN]	8,27
Q8WX93	Palladin OS=Homo sapiens GN=PALLD PE=1 SV=3 - [PALLD_HUMAN]	8,03
Q9Y2A7	Nck-associated protein 1 OS=Homo sapiens GN=NCKAP1 PE=1 SV=1 - [NCKP1_HUMAN]	7,80
Q9P265	Disco-interacting protein 2 homolog B OS=Homo sapiens GN=DIP2B PE=1 SV=3 - [DIP2B_HUMAN]	7,42
O95785	Protein Wiz OS=Homo sapiens GN=WIZ PE=1 SV=2 - [WIZ_HUMAN]	7,33
Q7L576	Cytoplasmic FMR1-interacting protein 1 OS=Homo sapiens GN=CYFIP1 PE=1 SV=1 - [CYFP1_HUMAN]	7,18
Q96T23	Remodeling and spacing factor 1 OS=Homo sapiens GN=RSF1 PE=1 SV=2 - [RSF1_HUMAN]	6,66
Q7Z6E9	E3 ubiquitin-protein ligase RBBP6 OS=Homo sapiens GN=RBBP6 PE=1 SV=1 - [RBBP6_HUMAN]	6,64
Q9BZF9	Uveal autoantigen with coiled-coil domains and ankyrin repeats OS=Homo sapiens GN=UACA PE=1 SV=2 - [UACA_HUMAN]	6,29
Q12923	Tyrosine-protein phosphatase non-receptor type 13 OS=Homo sapiens GN=PTPN13 PE=1 SV=2 - [PTN13_HUMAN]	4,67
A6NHR9	Structural maintenance of chromosomes flexible hinge domain-containing protein 1 OS=Homo sapiens GN=SMCHD1 PE=1 SV=2 - [SMHD	4,39
P05387	60S acidic ribosomal protein P2 OS=Homo sapiens GN=RPLP2 PE=1 SV=1 - [RLA2_HUMAN]	77,39
P60660-2	Isoform Smooth muscle of Myosin light polypeptide 6 OS=Homo sapiens GN=MYL6 - [MYL6_HUMAN]	75,50
P60709	Actin, cytoplasmic 1 OS=Homo sapiens GN=ACTB PE=1 SV=1 - [ACTB_HUMAN]	62,67
P63208	S-phase kinase-associated protein 1 OS=Homo sapiens GN=SKP1 PE=1 SV=2 - [SKP1_HUMAN]	58,90
Q96DA6	Mitochondrial import inner membrane translocase subunit TIM14 OS=Homo sapiens GN=DNAJC19 PE=1 SV=3 - [TIM14_HUMAN]	54,31
P68366	Tubulin alpha-4A chain OS=Homo sapiens GN=TUBA4A PE=1 SV=1 - [TBA4A_HUMAN]	53,57
P67936	Tropomyosin alpha-4 chain OS=Homo sapiens GN=TPM4 PE=1 SV=3 - [TPM4_HUMAN]	52,42
Q9NVI7-2	Isoform 2 of ATPase family AAA domain-containing protein 3A OS=Homo sapiens GN=ATAD3A - [ATD3A_HUMAN]	52,22
P31947	14-3-3 protein sigma OS=Homo sapiens GN=SFN PE=1 SV=1 - [1433S_HUMAN]	51,61
Q9NP72	Ras-related protein Rab-18 OS=Homo sapiens GN=RAB18 PE=1 SV=1 - [RAB18_HUMAN]	50,00
P67809	Nuclease-sensitive element-binding protein 1 OS=Homo sapiens GN=YBX1 PE=1 SV=3 - [YBOX1_HUMAN]	49,07
O95298	NADH dehydrogenase [ubiquinone] 1 subunit C2 OS=Homo sapiens GN=NDUFC2 PE=1 SV=1 - [NDUC2_HUMAN]	48,74
Q56VL3	OCIA domain-containing protein 2 OS=Homo sapiens GN=OCIAD2 PE=1 SV=1 - [OCAD2_HUMAN]	48,70
O95881	Thioredoxin domain-containing protein 12 OS=Homo sapiens GN=TXNDC12 PE=1 SV=1 - [TXD12_HUMAN]	48,26
Q8WW12	PEST proteolytic signal-containing nuclear protein OS=Homo sapiens GN=PCNP PE=1 SV=2 - [PCNP_HUMAN]	46,07
P09211	Glutathione S-transferase P OS=Homo sapiens GN=GSTP1 PE=1 SV=2 - [GSTP1_HUMAN]	45,71
Q86Y82	Syntaxin-12 OS=Homo sapiens GN=STX12 PE=1 SV=1 - [STX12_HUMAN]	45,29
P62910	60S ribosomal protein L32 OS=Homo sapiens GN=RPL32 PE=1 SV=2 - [RL32_HUMAN]	45,19
Q03135	Caveolin-1 OS=Homo sapiens GN=CAV1 PE=1 SV=4 - [CAV1_HUMAN]	43,82
Q96E11	Ribosome-recycling factor, mitochondrial OS=Homo sapiens GN=MRRF PE=1 SV=1 - [RRFM_HUMAN]	43,13
P62241	40S ribosomal protein S8 OS=Homo sapiens GN=RPS8 PE=1 SV=2 - [RS8_HUMAN]	42,31
O00299	Chloride intracellular channel protein 1 OS=Homo sapiens GN=CLIC1 PE=1 SV=4 - [CLIC1_HUMAN]	41,49
P62081	40S ribosomal protein S7 OS=Homo sapiens GN=RPS7 PE=1 SV=1 - [RS7_HUMAN]	40,72
P18669	Phosphoglycerate mutase 1 OS=Homo sapiens GN=PGAM1 PE=1 SV=2 - [PGAM1_HUMAN]	39,76
Q9Y3A6	Transmembrane emp24 domain-containing protein 5 OS=Homo sapiens GN=TMED5 PE=1 SV=1 - [TMED5_HUMAN]	39,74
P61313	60S ribosomal protein L15 OS=Homo sapiens GN=RPL15 PE=1 SV=2 - [RL15_HUMAN]	39,71
Q9UBX3	Mitochondrial dicarboxylate carrier OS=Homo sapiens GN=SLC25A10 PE=1 SV=2 - [DIC_HUMAN]	39,37
O75396	Vesicle-trafficking protein SEC22b OS=Homo sapiens GN=SEC22B PE=1 SV=4 - [SC22B_HUMAN]	39,07
Q06323	Proteasome activator complex subunit 1 OS=Homo sapiens GN=PSME1 PE=1 SV=1 - [PSME1_HUMAN]	38,96
P16989	DNA-binding protein A OS=Homo sapiens GN=CSDA PE=1 SV=4 - [DBPA_HUMAN]	38,71
P30040	Endoplasmic reticulum resident protein 29 OS=Homo sapiens GN=ERP29 PE=1 SV=4 - [ERP29_HUMAN]	37,93
Q96KN1	Protein FAM84B OS=Homo sapiens GN=FAM84B PE=1 SV=1 - [FA84B_HUMAN]	37,42
P30084	Enoyl-CoA hydratase, mitochondrial OS=Homo sapiens GN=ECHS1 PE=1 SV=4 - [ECHM_HUMAN]	36,90
P29992	Guanine nucleotide-binding protein subunit alpha-11 OS=Homo sapiens GN=GNA11 PE=1 SV=2 - [GNA11_HUMAN]	36,21
O15126	Secretory carrier-associated membrane protein 1 OS=Homo sapiens GN=SCAMP1 PE=1 SV=2 - [SCAM1_HUMAN]	36,09
P13995	Bifunctional methylenetetrahydrofolate dehydrogenase/cyclohydrolase, mitochondrial OS=Homo sapiens GN=MTHFD2 PE=1 SV=2 - [MTD	36,00
P62906	60S ribosomal protein L10a OS=Homo sapiens GN=RPL10A PE=1 SV=2 - [RL10A_HUMAN]	35,48
P62750	60S ribosomal protein L23a OS=Homo sapiens GN=RPL23A PE=1 SV=1 - [RL23A_HUMAN]	35,26
Q15417	Calponin-3 OS=Homo sapiens GN=CNN3 PE=1 SV=1 - [CNN3_HUMAN]	34,65
P09429	High mobility group protein B1 OS=Homo sapiens GN=HMGB1 PE=1 SV=3 - [HMGB1_HUMAN]	33,95
P31937	3-hydroxyisobutyrate dehydrogenase, mitochondrial OS=Homo sapiens GN=HIBADH PE=1 SV=2 - [3HIDH_HUMAN]	33,93
P00387	NADH-cytochrome b5 reductase 3 OS=Homo sapiens GN=CYB5R3 PE=1 SV=3 - [NB5R3_HUMAN]	33,89
P40429	60S ribosomal protein L13a OS=Homo sapiens GN=RPL13A PE=1 SV=2 - [RL13A_HUMAN]	33,50
Q9NX62	Inositol monophosphatase 3 OS=Homo sapiens GN=IMPAD1 PE=1 SV=1 - [IMPA3_HUMAN]	33,15
P15328	Folate receptor alpha OS=Homo sapiens GN=FOLR1 PE=1 SV=3 - [FOLR1_HUMAN]	33,07
P46778	60S ribosomal protein L21 OS=Homo sapiens GN=RPL21 PE=1 SV=2 - [RL21_HUMAN]	32,50
O94905	Erlin-2 OS=Homo sapiens GN=ERLIN2 PE=1 SV=1 - [ERLN2_HUMAN]	32,45
Q5T653	39S ribosomal protein L2, mitochondrial OS=Homo sapiens GN=MRPL2 PE=1 SV=2 - [RM02_HUMAN]	32,13
O95249	Golgi SNAP receptor complex member 1 OS=Homo sapiens GN=GOSR1 PE=1 SV=1 - [GOSR1_HUMAN]	32,00
Q15006	Tetratricopeptide repeat protein 35 OS=Homo sapiens GN=TTC35 PE=1 SV=1 - [TTC35_HUMAN]	31,99
P84103	Serine/arginine-rich splicing factor 3 OS=Homo sapiens GN=SRSF3 PE=1 SV=1 - [SRSF3_HUMAN]	31,71
P50914	60S ribosomal protein L14 OS=Homo sapiens GN=RPL14 PE=1 SV=4 - [RL14_HUMAN]	31,63
Q9UH62	Armadillo repeat-containing X-linked protein 3 OS=Homo sapiens GN=ARMCX3 PE=1 SV=1 - [ARMX3_HUMAN]	30,34
P84098	60S ribosomal protein L19 OS=Homo sapiens GN=RPL19 PE=1 SV=1 - [RL19_HUMAN]	30,10
O75381	Peroxisomal membrane protein PEX14 OS=Homo sapiens GN=PEX14 PE=1 SV=1 - [PEX14_HUMAN]	29,44
O15372	Eukaryotic translation initiation factor 3 subunit H OS=Homo sapiens GN=EIF3H PE=1 SV=1 - [EIF3H_HUMAN]	28,13
Q9UKM9	RNA-binding protein Raly OS=Homo sapiens GN=RALY PE=1 SV=1 - [RALY_HUMAN]	28,10
Q9H2F3	3 beta-hydroxysteroid dehydrogenase type 7 OS=Homo sapiens GN=HSD3B7 PE=1 SV=2 - [3BHS7_HUMAN]	27,64
P17301	Integrin alpha-2 OS=Homo sapiens GN=ITGA2 PE=1 SV=1 - [ITA2_HUMAN]	27,43
Q96HS1	Serine/threonine-protein phosphatase PGAM5, mitochondrial OS=Homo sapiens GN=PGAM5 PE=1 SV=2 - [PGAM5_HUMAN]	26,30
O75821	Eukaryotic translation initiation factor 3 subunit G OS=Homo sapiens GN=EIF3G PE=1 SV=2 - [EIF3G_HUMAN]	26,25
O14828	Secretory carrier-associated membrane protein 3 OS=Homo sapiens GN=SCAMP3 PE=1 SV=3 - [SCAM3_HUMAN]	26,22
Q9Y375	Complex I intermediate-associated protein 30, mitochondrial OS=Homo sapiens GN=NDUFAF1 PE=1 SV=2 - [CIA30_HUMAN]	25,99
P40925	Malate dehydrogenase, cytoplasmic OS=Homo sapiens GN=MDH1 PE=1 SV=4 - [MDHC_HUMAN]	25,75
P21281	V-type proton ATPase subunit B, brain isoform OS=Homo sapiens GN=ATP6V1B2 PE=1 SV=3 - [VATB2_HUMAN]	25,44
P30520	Adenylosuccinate synthetase isozyme 2 OS=Homo sapiens GN=ADSS PE=1 SV=3 - [PURA2_HUMAN]	24,56
Q13283	Ras GTPase-activating protein-binding protein 1 OS=Homo sapiens GN=G3BP1 PE=1 SV=1 - [G3BP1_HUMAN]	24,46
Q6NZI2	Polymerase I and transcript release factor OS=Homo sapiens GN=PTRF PE=1 SV=1 - [PTRF_HUMAN]	24,36
Q16543	Hsp90 co-chaperone Cdc37 OS=Homo sapiens GN=CDC37 PE=1 SV=1 - [CDC37_HUMAN]	24,34
Q9HC21	Mitochondrial thiamine pyrophosphate carrier OS=Homo sapiens GN=SLC25A19 PE=1 SV=1 - [TPC_HUMAN]	24,06
Q658P3	Metalloreductase STEAP3 OS=Homo sapiens GN=STEAP3 PE=1 SV=2 - [STEA3_HUMAN]	23,98
P53634	Dipeptidyl peptidase 1 OS=Homo sapiens GN=CTSC PE=1 SV=2 - [CATC_HUMAN]	23,97
O75874	Isocitrate dehydrogenase [NADP] cytoplasmic OS=Homo sapiens GN=IDH1 PE=1 SV=2 - [IDHC_HUMAN]	23,19
Q9NTK5	Obg-like ATPase 1 OS=Homo sapiens GN=OLA1 PE=1 SV=2 - [OLA1_HUMAN]	22,98
O43272	Proline dehydrogenase 1, mitochondrial OS=Homo sapiens GN=PRODH PE=1 SV=3 - [PROD_HUMAN]	22,83

Q8NHP6	Motile sperm domain-containing protein 2 OS=Homo sapiens GN=MOSPD2 PE=1 SV=1 - [MSPD2_HUMAN]	22,78	8
P16219	Short-chain specific acyl-CoA dehydrogenase, mitochondrial OS=Homo sapiens GN=ACADS PE=1 SV=1 - [ACADS_HUMAN]	22,57	8
P11310	Medium-chain specific acyl-CoA dehydrogenase, mitochondrial OS=Homo sapiens GN=ACADM PE=1 SV=1 - [ACADM_HUMAN]	22,57	8
Q96CW1	AP-2 complex subunit mu OS=Homo sapiens GN=AP2M1 PE=1 SV=2 - [AP2M1_HUMAN]	22,53	8
P26368	Splicing factor U2AF 65 kDa subunit OS=Homo sapiens GN=U2AF2 PE=1 SV=4 - [U2AF2_HUMAN]	22,53	8
P08174	Complement decay-accelerating factor OS=Homo sapiens GN=CD55 PE=1 SV=4 - [DAF_HUMAN]	22,31	8
Q10713	Mitochondrial-processing peptidase subunit alpha OS=Homo sapiens GN=PMPCA PE=1 SV=2 - [MPPA_HUMAN]	22,29	8
P20592	Interferon-induced GTP-binding protein Mx2 OS=Homo sapiens GN=MX2 PE=1 SV=1 - [MX2_HUMAN]	22,24	8
P26599	Polypyrimidine tract-binding protein 1 OS=Homo sapiens GN=PTBP1 PE=1 SV=1 - [PTBP1_HUMAN]	22,22	8
Q9NUQ6-2	Isoform 2 of SPATS2-like protein OS=Homo sapiens GN=SPATS2L - [SPS2L_HUMAN]	22,09	8
P50995	Annexin A11 OS=Homo sapiens GN=ANXA11 PE=1 SV=1 - [ANX11_HUMAN]	21,78	8
P00390	Glutathione reductase, mitochondrial OS=Homo sapiens GN=GSR PE=1 SV=2 - [GSHR_HUMAN]	21,65	8
Q14699	Raftlin OS=Homo sapiens GN=RFTN1 PE=1 SV=4 - [RFTN1_HUMAN]	21,28	8
P61221	ATP-binding cassette sub-family E member 1 OS=Homo sapiens GN=ABCE1 PE=1 SV=1 - [ABCE1_HUMAN]	21,04	8
Q9UHG3	Prenylcysteine oxidase 1 OS=Homo sapiens GN=PCYOX1 PE=1 SV=3 - [PCYOX_HUMAN]	20,99	8
Q9UHR4	Brain-specific angiogenesis inhibitor 1-associated protein 2-like protein 1 OS=Homo sapiens GN=BAIAP2L1 PE=1 SV=2 - [BI2L1_HUMAN	20,35	8
Q99873	Protein arginine N-methyltransferase 1 OS=Homo sapiens GN=PRMT1 PE=1 SV=2 - [ANM1_HUMAN]	20,22	8
Q10472	Polypeptide N-acetylgalactosaminyltransferase 1 OS=Homo sapiens GN=GALNT1 PE=1 SV=1 - [GALT1_HUMAN]	20,21	8
Q15758	Neutral amino acid transporter B(0) OS=Homo sapiens GN=SLC1A5 PE=1 SV=2 - [AAAT_HUMAN]	20,15	8
O15371	Eukaryotic translation initiation factor 3 subunit D OS=Homo sapiens GN=EIF3D PE=1 SV=1 - [EIF3D_HUMAN]	19,71	8
P13674	Prolyl 4-hydroxylase subunit alpha-1 OS=Homo sapiens GN=P4HA1 PE=1 SV=2 - [P4HA1_HUMAN]	19,66	8
P11166	Solute carrier family 2, facilitated glucose transporter member 1 OS=Homo sapiens GN=SLC2A1 PE=1 SV=2 - [GTR1_HUMAN]	19,51	8
P63010	AP-2 complex subunit beta OS=Homo sapiens GN=AP2B1 PE=1 SV=1 - [AP2B1_HUMAN]	19,42	8
Q01650	Large neutral amino acids transporter small subunit 1 OS=Homo sapiens GN=SLC7A5 PE=1 SV=2 - [LAT1_HUMAN]	19,13	8
P09960	Leukotriene A-4 hydrolase OS=Homo sapiens GN=LTA4H PE=1 SV=2 - [LKHA4_HUMAN]	18,82	8
P00749	Urokinase-type plasminogen activator OS=Homo sapiens GN=PLAU PE=1 SV=2 - [UROK_HUMAN]	18,79	8
Q8TAA9	Vang-like protein 1 OS=Homo sapiens GN=VANGL1 PE=1 SV=1 - [VANG1_HUMAN]	18,70	8
Q8IVT2	Uncharacterized protein C19orf21 OS=Homo sapiens GN=C19orf21 PE=1 SV=1 - [CS021_HUMAN]	18,56	8
P52292	Importin subunit alpha-2 OS=Homo sapiens GN=KPNA2 PE=1 SV=1 - [IMA2_HUMAN]	18,53	8
Q9BXF6	Rab11 family-interacting protein 5 OS=Homo sapiens GN=RAB11FIP5 PE=1 SV=1 - [RFIP5_HUMAN]	18,22	8
Q86YP4	Transcriptional repressor p66-alpha OS=Homo sapiens GN=GATAD2A PE=1 SV=1 - [P66A_HUMAN]	18,17	8
O75427	Leucine-rich repeat and calponin homology domain-containing protein 4 OS=Homo sapiens GN=LRCH4 PE=1 SV=2 - [LRCH4_HUMAN]	18,01	8
O75695	Protein XRP2 OS=Homo sapiens GN=RP2 PE=1 SV=4 - [XRP2_HUMAN]	18,00	8
Q5T3I0	G patch domain-containing protein 4 OS=Homo sapiens GN=GPATCH4 PE=1 SV=2 - [GPTC4_HUMAN]	17,94	8
Q9UHB9	Signal recognition particle 68 kDa protein OS=Homo sapiens GN=SRP68 PE=1 SV=2 - [SRP68_HUMAN]	17,70	8
Q9Y230	RuvB-like 2 OS=Homo sapiens GN=RUVBL2 PE=1 SV=3 - [RUVB2_HUMAN]	17,49	8
Q9NVP1	ATP-dependent RNA helicase DDX18 OS=Homo sapiens GN=DDX18 PE=1 SV=2 - [DDX18_HUMAN]	17,31	8
Q96AY3	Peptidyl-prolyl cis-trans isomerase FKBP10 OS=Homo sapiens GN=FKBP10 PE=1 SV=1 - [FKB10_HUMAN]	17,18	8
O60832	H/ACA ribonucleoprotein complex subunit 4 OS=Homo sapiens GN=DKC1 PE=1 SV=3 - [DKC1_HUMAN]	17,12	8
Q9UBT2	SUMO-activating enzyme subunit 2 OS=Homo sapiens GN=UBA2 PE=1 SV=2 - [SAE2_HUMAN]	16,72	8
Q8IYS2	Uncharacterized protein KIAA2013 OS=Homo sapiens GN=KIAA2013 PE=2 SV=1 - [K2013_HUMAN]	16,56	8
Q03519	Antigen peptide transporter 2 OS=Homo sapiens GN=TAP2 PE=1 SV=1 - [TAP2_HUMAN]	16,18	8
O43172	U4/U6 small nuclear ribonucleoprotein Prp4 OS=Homo sapiens GN=PRPF4 PE=1 SV=2 - [PRP4_HUMAN]	16,09	8
Q9NRC1	Suppressor of tumorigenicity 7 protein OS=Homo sapiens GN=ST7 PE=1 SV=1 - [ST7_HUMAN]	16,07	8
Q8WXF1	Paraspeckle component 1 OS=Homo sapiens GN=PSPC1 PE=1 SV=1 - [PSPC1_HUMAN]	16,06	8
Q14534	Squalene monooxygenase OS=Homo sapiens GN=SQLE PE=2 SV=3 - [ERG1_HUMAN]	15,85	8
O60568	Procollagen-lysine,2-oxoglutarate 5-dioxygenase 3 OS=Homo sapiens GN=PLOD3 PE=1 SV=1 - [PLOD3_HUMAN]	15,85	8
P30825	High affinity cationic amino acid transporter 1 OS=Homo sapiens GN=SLC7A1 PE=1 SV=1 - [CTR1_HUMAN]	15,42	8
Q5SSJ5	Heterochromatin protein 1-binding protein 3 OS=Homo sapiens GN=HP1BP3 PE=1 SV=1 - [HP1B3_HUMAN]	15,37	8
P27694	Replication protein A 70 kDa DNA-binding subunit OS=Homo sapiens GN=RPA1 PE=1 SV=2 - [RFA1_HUMAN]	15,26	8
Q03518	Antigen peptide transporter 1 OS=Homo sapiens GN=TAP1 PE=1 SV=2 - [TAP1_HUMAN]	15,22	8
P22033	Methylmalonyl-CoA mutase, mitochondrial OS=Homo sapiens GN=MUT PE=1 SV=4 - [MUTA_HUMAN]	14,93	8
P15170	Eukaryotic peptide chain release factor GTP-binding subunit ERF3A OS=Homo sapiens GN=GSPT1 PE=1 SV=1 - [ERF3A_HUMAN]	14,83	8
Q92575	UBX domain-containing protein 4 OS=Homo sapiens GN=UBXN4 PE=1 SV=2 - [UBXN4_HUMAN]	14,76	8
Q969X6	Cirhin OS=Homo sapiens GN=CIRH1A PE=1 SV=1 - [CIR1A_HUMAN]	14,72	8
Q9NW15	Anoctamin-10 OS=Homo sapiens GN=ANO10 PE=1 SV=2 - [ANO10_HUMAN]	14,55	8
Q9HD45	Transmembrane 9 superfamily member 3 OS=Homo sapiens GN=TM9SF3 PE=1 SV=2 - [TM9S3_HUMAN]	14,43	8
Q99805	Transmembrane 9 superfamily member 2 OS=Homo sapiens GN=TM9SF2 PE=1 SV=1 - [TM9S2_HUMAN]	14,33	8
O00592	Podocalyxin OS=Homo sapiens GN=PODXL PE=1 SV=2 - [PODXL_HUMAN]	14,16	8
Q96AQ8	Coiled-coil domain-containing protein 90A, mitochondrial OS=Homo sapiens GN=CCDC90A PE=2 SV=1 - [CC90A_HUMAN]	13,93	8
O15228	Dihydroxyacetone phosphate acyltransferase OS=Homo sapiens GN=GNPAT PE=1 SV=1 - [GNPAT_HUMAN]	13,68	8
P33991	DNA replication licensing factor MCM4 OS=Homo sapiens GN=MCM4 PE=1 SV=5 - [MCM4_HUMAN]	13,33	8
Q9UG63	ATP-binding cassette sub-family F member 2 OS=Homo sapiens GN=ABCF2 PE=1 SV=2 - [ABCF2_HUMAN]	13,32	8
Q14444	Caprin-1 OS=Homo sapiens GN=CAPRIN1 PE=1 SV=2 - [CAPR1_HUMAN]	13,26	8
P17655	Calpain-2 catalytic subunit OS=Homo sapiens GN=CAPN2 PE=1 SV=6 - [CAN2_HUMAN]	13,14	8
P50416	Carnitine O-palmitoyltransferase 1, liver isoform OS=Homo sapiens GN=CPT1A PE=1 SV=2 - [CPT1A_HUMAN]	13,07	8
Q9Y2W2	WW domain-binding protein 11 OS=Homo sapiens GN=WBP11 PE=1 SV=1 - [WBP11_HUMAN]	12,95	8
O75410	Transforming acidic coiled-coil-containing protein 1 OS=Homo sapiens GN=TACC1 PE=1 SV=2 - [TACC1_HUMAN]	12,80	8
Q9H330-2	Isoform 2 of Transmembrane protein 245 OS=Homo sapiens GN=TMEM245 - [TM245_HUMAN]	12,63	8
P49589	Cysteine--tRNA ligase, cytoplasmic OS=Homo sapiens GN=CARS PE=1 SV=3 - [SYCC_HUMAN]	12,57	8
Q14566	DNA replication licensing factor MCM6 OS=Homo sapiens GN=MCM6 PE=1 SV=1 - [MCM6_HUMAN]	12,30	8
O95757	Heat shock 70 kDa protein 4L OS=Homo sapiens GN=HSPA4L PE=1 SV=3 - [HS74L_HUMAN]	12,04	8
P49736	DNA replication licensing factor MCM2 OS=Homo sapiens GN=MCM2 PE=1 SV=4 - [MCM2_HUMAN]	11,84	8
Q08345	Epithelial discoidin domain-containing receptor 1 OS=Homo sapiens GN=DDR1 PE=1 SV=1 - [DDR1_HUMAN]	11,83	8
Q92973	Transportin-1 OS=Homo sapiens GN=TNPO1 PE=1 SV=2 - [TNPO1_HUMAN]	11,80	8
Q9Y2G8	DnaJ homolog subfamily C member 16 OS=Homo sapiens GN=DNAJC16 PE=2 SV=3 - [DJC16_HUMAN]	11,76	8
P50570	Dynamin-2 OS=Homo sapiens GN=DNM2 PE=1 SV=2 - [DYN2_HUMAN]	10,69	8
Q14157	Ubiquitin-associated protein 2-like OS=Homo sapiens GN=UBAP2L PE=1 SV=2 - [UBP2L_HUMAN]	10,67	8
Q8IZA0	Dyslexia-associated protein KIAA0319-like protein OS=Homo sapiens GN=KIAA0319L PE=1 SV=2 - [K319L_HUMAN]	10,49	8
Q9Y2D5	A-kinase anchor protein 2 OS=Homo sapiens GN=AKAP2 PE=1 SV=3 - [AKAP2_HUMAN]	10,36	8
Q7L2E3	Putative ATP-dependent RNA helicase DHX30 OS=Homo sapiens GN=DHX30 PE=1 SV=1 - [DHX30_HUMAN]	9,88	8
Q9NTZ6	RNA-binding protein 12 OS=Homo sapiens GN=RBM12 PE=1 SV=1 - [RBM12_HUMAN]	9,76	8
Q9Y4F1	FERM, RhoGEF and pleckstrin domain-containing protein 1 OS=Homo sapiens GN=FARP1 PE=1 SV=1 - [FARP1_HUMAN]	9,47	8
P08069	Insulin-like growth factor 1 receptor OS=Homo sapiens GN=IGF1R PE=1 SV=1 - [IGF1R_HUMAN]	9,29	8
Q8NB49	Probable phospholipid-transporting ATPase IG OS=Homo sapiens GN=ATP11C PE=1 SV=3 - [AT11C_HUMAN]	9,10	8
P49916	DNA ligase 3 OS=Homo sapiens GN=LIG3 PE=1 SV=2 - [DNLI3_HUMAN]	8,52	8
Q76FK4	Nucleolar protein 8 OS=Homo sapiens GN=NOL8 PE=1 SV=1 - [NOL8_HUMAN]	8,31	8
Q9NWH9	SAFB-like transcription modulator OS=Homo sapiens GN=SLTM PE=1 SV=2 - [SLTM_HUMAN]	8,22	8
Q86SQ0	Pleckstrin homology-like domain family B member 2 OS=Homo sapiens GN=PHLDB2 PE=1 SV=2 - [PHLB2_HUMAN]	7,82	8
Q92887	Canalicular multispecific organic anion transporter 1 OS=Homo sapiens GN=ABCC2 PE=1 SV=3 - [MRP2_HUMAN]	7,64	8
P30876	DNA-directed RNA polymerase II subunit RPB2 OS=Homo sapiens GN=POLR2B PE=1 SV=1 - [RPB2_HUMAN]	7,50	8
Q92859	Neogenin OS=Homo sapiens GN=NEO1 PE=1 SV=2 - [NEO1_HUMAN]	7,46	8
Q02880	DNA topoisomerase 2-beta OS=Homo sapiens GN=TOP2B PE=1 SV=3 - [TOP2B_HUMAN]	7,13	8
P08572	Collagen alpha-2(IV) chain OS=Homo sapiens GN=COL4A2 PE=1 SV=4 - [CO4A2_HUMAN]	6,95	8
O15067	Phosphoribosylformylglycinamidine synthase OS=Homo sapiens GN=PFAS PE=1 SV=4 - [PUR4_HUMAN]	6,73	8
Q9UIG0	Tyrosine-protein kinase BAZ1B OS=Homo sapiens GN=BAZ1B PE=1 SV=2 - [BAZ1B_HUMAN]	6,14	8
Q9NTI5	Sister chromatid cohesion protein PDS5 homolog B OS=Homo sapiens GN=PDS5B PE=1 SV=1 - [PDS5B_HUMAN]	5,94	8
A5YKK6	CCR4-NOT transcription complex subunit 1 OS=Homo sapiens GN=CNOT1 PE=1 SV=2 - [CNOT1_HUMAN]	5,05	8
P52948	Nuclear pore complex protein Nup98-Nup96 OS=Homo sapiens GN=NUP98 PE=1 SV=4 - [NUP98_HUMAN]	4,84	8
Q9BTC0	Death-inducer obliterator 1 OS=Homo sapiens GN=DIDO1 PE=1 SV=5 - [DIDO1_HUMAN]	4,38	8
Q9BYK8	Peroxisomal proliferator-activated receptor A-interacting complex 285 kDa protein OS=Homo sapiens GN=PRIC285 PE=1 SV=6 - [PR285	4,23	8
P50851-2	Isoform 2 of Lipopolysaccharide-responsive and beige-like anchor protein OS=Homo sapiens GN=LRBA - [LRBA_HUMAN]	3,51	8
Q9NU22	Midasin OS=Homo sapiens GN=MDN1 PE=1 SV=2 - [MDN1_HUMAN]	1,66	8
Q16718	NADH dehydrogenase [ubiquinone] 1 alpha subcomplex subunit 5 OS=Homo sapiens GN=NDUFA5 PE=1 SV=3 - [NDUA5_HUMAN]	66,38	7
P61019	Ras-related protein Rab-2A OS=Homo sapiens GN=RAB2A PE=1 SV=1 - [RAB2A_HUMAN]	65,57	7
P10412	Histone H1.4 OS=Homo sapiens GN=HIST1H1E PE=1 SV=2 - [H14_HUMAN]	63,01	7
P99999	Cytochrome c OS=Homo sapiens GN=CYCS PE=1 SV=2 - [CYC_HUMAN]	61,90	7
P62316	Small nuclear ribonucleoprotein Sm D2 OS=Homo sapiens GN=SNRPD2 PE=1 SV=1 - [SMD2_HUMAN]	61,02	7
Q9GZT3	SRA stem-loop-interacting RNA-binding protein, mitochondrial OS=Homo sapiens GN=SLIRP PE=1 SV=1 - [SLIRP_HUMAN]	60,55	7
Q9BQE3	Tubulin alpha-1C chain OS=Homo sapiens GN=TUBA1C PE=1 SV=1 - [TBA1C_HUMAN]	56,57	7
P62805	Histone H4 OS=Homo sapiens GN=HIST1H4A PE=1 SV=2 - [H4_HUMAN]	56,31	7
F5H2A4	High mobility group protein HMGI-C OS=Homo sapiens GN=HMGA2 PE=4 SV=1 - [F5H2A4_HUMAN]	55,08	7
Q13232	Nucleoside diphosphate kinase 3 OS=Homo sapiens GN=NME3 PE=1 SV=2 - [NDK3_HUMAN]	54,44	7
P32969	60S ribosomal protein L9 OS=Homo sapiens GN=RPL9 PE=1 SV=1 - [RL9_HUMAN]	52,60	7
P14927	Cytochrome b-c1 complex subunit 7 OS=Homo sapiens GN=UQCRB PE=1 SV=2 - [QCR7_HUMAN]	52,25	7
P00441	Superoxide dismutase [Cu-Zn] OS=Homo sapiens GN=SOD1 PE=1 SV=2 - [SODC_HUMAN]	51,95	7
Q15286	Ras-related protein Rab-35 OS=Homo sapiens GN=RAB35 PE=1 SV=1 - [RAB35_HUMAN]	50,75	7
Q8N5K1	CDGSH iron-sulfur domain-containing protein 2 OS=Homo sapiens GN=CISD2 PE=1 SV=1 - [CISD2_HUMAN]	50,37	7
Q9H3Z4	DnaJ homolog subfamily C member 5 OS=Homo sapiens GN=DNAJC5 PE=1 SV=1 - [DNJC5_HUMAN]	50,00	7
Q15365	Poly(rC)-binding protein 1 OS=Homo sapiens GN=PCBP1 PE=1 SV=2 - [PCBP1_HUMAN]	46,91	7
P62070	Ras-related protein R-Ras2 OS=Homo sapiens GN=RRAS2 PE=1 SV=1 - [RRAS2_HUMAN]	46,57	7
P10606	Cytochrome c oxidase subunit 5B, mitochondrial OS=Homo sapiens GN=COX5B PE=1 SV=2 - [COX5B_HUMAN]	46,51	7
Q9H444	Charged multivesicular body protein 4b OS=Homo sapiens GN=CHMP4B PE=1 SV=1 - [CHM4B_HUMAN]	44,64	7
P60953	Cell division control protein 42 homolog OS=Homo sapiens GN=CDC42 PE=1 SV=2 - [CDC42_HUMAN]	43,98	7
P10301	Ras-related protein R-Ras OS=Homo sapiens GN=RRAS PE=1 SV=1 - [RRAS_HUMAN]	43,12	7
Q13126	S-methyl-5'-thioadenosine phosphorylase OS=Homo sapiens GN=MTAP PE=1 SV=2 - [MTAP_HUMAN]	42,05	7
P16949	Stathmin OS=Homo sapiens GN=STMN1 PE=1 SV=3 - [STMN1_HUMAN]	41,61	7
P62913	60S ribosomal protein L11 OS=Homo sapiens GN=RPL11 PE=1 SV=2 - [RL11_HUMAN]	41,01	7
P84095	Rho-related GTP-binding protein RhoG OS=Homo sapiens GN=RHOG PE=1 SV=1 - [RHOG_HUMAN]	40,84	7
O43752	Syntaxin-6 OS=Homo sapiens GN=STX6 PE=1 SV=1 - [STX6_HUMAN]	40,39	7
Q15691	Microtubule-associated protein RP/EB family member 1 OS=Homo sapiens GN=MAPRE1 PE=1 SV=3 - [MARE1_HUMAN]	39,93	7
P52907	F-actin-capping protein subunit alpha-1 OS=Homo sapiens GN=CAPZA1 PE=1 SV=3 - [CAZA1_HUMAN]	39,86	7
Q9Y277	Voltage-dependent anion-selective channel protein 3 OS=Homo sapiens GN=VDAC3 PE=1 SV=1 - [VDAC3_HUMAN]	39,58	7
P24534	Elongation factor 1-beta OS=Homo sapiens GN=EEF1B2 PE=1 SV=3 - [EF1B_HUMAN]	39,56	7
Q16270	Insulin-like growth factor-binding protein 7 OS=Homo sapiens GN=IGFBP7 PE=1 SV=1 - [IBP7_HUMAN]	39,36	7
P55145	Mesencephalic astrocyte-derived neurotrophic factor OS=Homo sapiens GN=MANF PE=1 SV=3 - [MANF_HUMAN]	39,01	7
Q9H910	Hematological and neurological expressed 1-like protein OS=Homo sapiens GN=HN1L PE=1 SV=1 - [HN1L_HUMAN]	37,89	7
Q9Y3B7	39S ribosomal protein L11, mitochondrial OS=Homo sapiens GN=MRPL11 PE=1 SV=1 - [RM11_HUMAN]	37,50	7
P26583	High mobility group protein B2 OS=Homo sapiens GN=HMGB2 PE=1 SV=2 - [HMGB2_HUMAN]	36,84	7
P61353	60S ribosomal protein L27 OS=Homo sapiens GN=RPL27 PE=1 SV=2 - [RL27_HUMAN]	36,76	7
Q9NVS2	28S ribosomal protein S18a, mitochondrial OS=Homo sapiens GN=MRPS18A PE=1 SV=1 - [RT18A_HUMAN]	36,73	7
Q13442	28 kDa heat- and acid-stable phosphoprotein OS=Homo sapiens GN=PDAP1 PE=1 SV=1 - [HAP28_HUMAN]	36,46	7
Q9HB71	Calcyclin-binding protein OS=Homo sapiens GN=CACYBP PE=1 SV=2 - [CYBP_HUMAN]	36,40	7
P32322	Pyrroline-5-carboxylate reductase 1, mitochondrial OS=Homo sapiens GN=PYCR1 PE=1 SV=2 - [P5CR1_HUMAN]	36,36	7
P08865	40S ribosomal protein SA OS=Homo sapiens GN=RPSA PE=1 SV=4 - [RSSA_HUMAN]	35,93	7
Q02543	60S ribosomal protein L18a OS=Homo sapiens GN=RPL18A PE=1 SV=2 - [RL18A_HUMAN]	35,80	7
Q969X5	Endoplasmic reticulum-Golgi intermediate compartment protein 1 OS=Homo sapiens GN=ERGIC1 PE=1 SV=1 - [ERGI1_HUMAN]	35,52	7
Q9Y2R9	28S ribosomal protein S7, mitochondrial OS=Homo sapiens GN=MRPS7 PE=1 SV=2 - [RT07_HUMAN]	35,12	7
O00584	Ribonuclease T2 OS=Homo sapiens GN=RNASET2 PE=1 SV=2 - [RNT2_HUMAN]	33,20	7
P62277	40S ribosomal protein S13 OS=Homo sapiens GN=RPS13 PE=1 SV=2 - [RS13_HUMAN]	32,45	7
P45973	Chromobox protein homolog 5 OS=Homo sapiens GN=CBX5 PE=1 SV=1 - [CBX5_HUMAN]	31,94	7
Q07955	Serine/arginine-rich splicing factor 1 OS=Homo sapiens GN=SRSF1 PE=1 SV=2 - [SRSF1_HUMAN]	31,85	7
Q9BRQ6	Coiled-coil-helix-coiled-coil-helix domain-containing protein 6 OS=Homo sapiens GN=CHCHD6 PE=1 SV=1 - [CHCH6_HUMAN]	31,49	7
Q13185	Chromobox protein homolog 3 OS=Homo sapiens GN=CBX3 PE=1 SV=4 - [CBX3_HUMAN]	31,15	7
O00217	NADH dehydrogenase [ubiquinone] iron-sulfur protein 8, mitochondrial OS=Homo sapiens GN=NDUFS8 PE=1 SV=1 - [NDUS8_HUMAN]	30,95	7
P38159-2	Isoform 2 of RNA-binding motif protein, X chromosome OS=Homo sapiens GN=RBMX - [RBMX_HUMAN]	30,69	7
Q9P0I2	Transmembrane protein 111 OS=Homo sapiens GN=TMEM111 PE=1 SV=3 - [TM111_HUMAN]	30,65	7
Q9H2U2	Inorganic pyrophosphatase 2, mitochondrial OS=Homo sapiens GN=PPA2 PE=1 SV=2 - [IPYR2_HUMAN]	30,54	7
Q7Z7K6-3	Isoform 3 of Centromere protein V OS=Homo sapiens GN=CENPV - [CENPV_HUMAN]	30,51	7
P40261	Nicotinamide N-methyltransferase OS=Homo sapiens GN=NNMT PE=1 SV=1 - [NNMT_HUMAN]	30,30	7
P09661	U2 small nuclear ribonucleoprotein A' OS=Homo sapiens GN=SNRPA1 PE=1 SV=2 - [RU2A_HUMAN]	29,80	7
P60900	Proteasome subunit alpha type-6 OS=Homo sapiens GN=PSMA6 PE=1 SV=1 - [PSA6_HUMAN]	29,67	7
Q9BRX8	Redox-regulatory protein FAM213A OS=Homo sapiens GN=FAM213A PE=1 SV=3 - [F213A_HUMAN]	29,26	7
O00151	PDZ and LIM domain protein 1 OS=Homo sapiens GN=PDLIM1 PE=1 SV=4 - [PDLI1_HUMAN]	27,96	7
Q9H0V9	VIP36-like protein OS=Homo sapiens GN=LMAN2L PE=1 SV=1 - [LMA2L_HUMAN]	27,87	7
O95299	NADH dehydrogenase [ubiquinone] 1 alpha subcomplex subunit 10, mitochondrial OS=Homo sapiens GN=NDUFA10 PE=1 SV=1 - [NDUAA	27,61	7
P12004	Proliferating cell nuclear antigen OS=Homo sapiens GN=PCNA PE=1 SV=1 - [PCNA_HUMAN]	27,59	7
Q8IZV5	Retinol dehydrogenase 10 OS=Homo sapiens GN=RDH10 PE=1 SV=1 - [RDH10_HUMAN]	27,57	7
Q16698	2,4-dienoyl-CoA reductase, mitochondrial OS=Homo sapiens GN=DECR1 PE=1 SV=1 - [DECR_HUMAN]	27,46	7
Q96RQ1	Endoplasmic reticulum-Golgi intermediate compartment protein 2 OS=Homo sapiens GN=ERGIC2 PE=1 SV=2 - [ERGI2_HUMAN]	27,32	7

O43684	Mitotic checkpoint protein BUB3 OS=Homo sapiens GN=BUB3 PE=1 SV=1 - [BUB3_HUMAN]	27,13	7	1,044	1,093	[illegible]
P12277	Creatine kinase B-type OS=Homo sapiens GN=CKB PE=1 SV=1 - [KCRB_HUMAN]	27,03	7	1,287	1,329	[illegible]
Q13011	Delta(3,5)-Delta(2,4)-dienoyl-CoA isomerase, mitochondrial OS=Homo sapiens GN=ECH1 PE=1 SV=2 - [ECH1_HUMAN]	26,83	7	0,590	0,647	[illegible]
P48507	Glutamate--cysteine ligase regulatory subunit OS=Homo sapiens GN=GCLM PE=1 SV=1 - [GSH0_HUMAN]	26,64	7	1,195	1,234	[illegible]
Q99436	Proteasome subunit beta type-7 OS=Homo sapiens GN=PSMB7 PE=1 SV=1 - [PSB7_HUMAN]	26,35	7	0,947	1,311	[illegible]
Q16739	Ceramide glucosyltransferase OS=Homo sapiens GN=UGCG PE=1 SV=1 - [CEGT_HUMAN]	25,63	7	1,486	1,613	[illegible]
Q9H0P0	Cytosolic 5'-nucleotidase 3 OS=Homo sapiens GN=NT5C3 PE=1 SV=3 - [5NT3_HUMAN]	25,60	7	0,600	0,696	[illegible]
Q15785	Mitochondrial import receptor subunit TOM34 OS=Homo sapiens GN=TOMM34 PE=1 SV=2 - [TOM34_HUMAN]	25,57	7	0,932	0,937	[illegible]
Q9UKX7	Nuclear pore complex protein Nup50 OS=Homo sapiens GN=NUP50 PE=1 SV=2 - [NUP50_HUMAN]	25,43	7		1,011	[illegible]
Q15654	Thyroid receptor-interacting protein 6 OS=Homo sapiens GN=TRIP6 PE=1 SV=3 - [TRIP6_HUMAN]	25,42	7	1,052	1,175	[illegible]
Q9UNZ2-4	Isoform 2 of NSFL1 cofactor p47 OS=Homo sapiens GN=NSFL1C - [NSF1C_HUMAN]	25,37	7			[illegible]
P23919	Thymidylate kinase OS=Homo sapiens GN=DTYMK PE=1 SV=4 - [KTHY_HUMAN]	25,00	7	0,880	0,781	[illegible]
P46734	Dual specificity mitogen-activated protein kinase kinase 3 OS=Homo sapiens GN=MAP2K3 PE=1 SV=2 - [MP2K3_HUMAN]	24,50	7	0,777	0,830	[illegible]
Q9BV23	Monoacylglycerol lipase ABHD6 OS=Homo sapiens GN=ABHD6 PE=2 SV=1 - [ABHD6_HUMAN]	24,33	7	0,835	0,814	[illegible]
Q3SXY8	ADP-ribosylation factor-like protein 13B OS=Homo sapiens GN=ARL13B PE=1 SV=1 - [AR13B_HUMAN]	24,30	7	1,253	1,206	[illegible]
Q14318	Peptidyl-prolyl cis-trans isomerase FKBP8 OS=Homo sapiens GN=FKBP8 PE=1 SV=2 - [FKBP8_HUMAN]	24,27	7	1,316	1,432	[illegible]
P13473	Lysosome-associated membrane glycoprotein 2 OS=Homo sapiens GN=LAMP2 PE=1 SV=2 - [LAMP2_HUMAN]	24,15	7	0,985	1,060	[illegible]
P20042	Eukaryotic translation initiation factor 2 subunit 2 OS=Homo sapiens GN=EIF2S2 PE=1 SV=2 - [IF2B_HUMAN]	24,02	7	1,069	0,854	[illegible]
O00214	Galectin-8 OS=Homo sapiens GN=LGALS8 PE=1 SV=4 - [LEG8_HUMAN]	23,97	7	2,190	1,656	[illegible]
P08758	Annexin A5 OS=Homo sapiens GN=ANXA5 PE=1 SV=2 - [ANXA5_HUMAN]	23,44	7	1,064	1,352	[illegible]
P23193	Transcription elongation factor A protein 1 OS=Homo sapiens GN=TCEA1 PE=1 SV=2 - [TCEA1_HUMAN]	23,26	7			[illegible]
Q9NPL8	Translocase of inner mitochondrial membrane domain-containing protein 1 OS=Homo sapiens GN=TIMMDC1 PE=1 SV=2 - [TIDC1_HUM	23,16	7	1,213	1,089	[illegible]
P50213	Isocitrate dehydrogenase [NAD] subunit alpha, mitochondrial OS=Homo sapiens GN=IDH3A PE=1 SV=1 - [IDH3A_HUMAN]	22,68	7	0,795	0,821	[illegible]
O60701	UDP-glucose 6-dehydrogenase OS=Homo sapiens GN=UGDH PE=1 SV=1 - [UGDH_HUMAN]	22,27	7	0,528	0,596	[illegible]
Q9NV96	Cell cycle control protein 50A OS=Homo sapiens GN=TMEM30A PE=1 SV=1 - [CC50A_HUMAN]	22,16	7	1,097	1,069	[illegible]
Q6RFH5	WD repeat-containing protein 74 OS=Homo sapiens GN=WDR74 PE=1 SV=1 - [WDR74_HUMAN]	22,08	7	0,840		[illegible]
Q9UKS6	Protein kinase C and casein kinase substrate in neurons protein 3 OS=Homo sapiens GN=PACSIN3 PE=1 SV=2 - [PACN3_HUMAN]	21,93	7	1,100	1,195	[illegible]
Q9C0E8	Protein lunapark OS=Homo sapiens GN=LNP PE=1 SV=2 - [LNP_HUMAN]	21,26	7	0,882	0,855	[illegible]
P61160	Actin-related protein 2 OS=Homo sapiens GN=ACTR2 PE=1 SV=1 - [ARP2_HUMAN]	21,07	7	0,765	0,671	[illegible]
P39748	Flap endonuclease 1 OS=Homo sapiens GN=FEN1 PE=1 SV=1 - [FEN1_HUMAN]	21,05	7	0,667	0,757	[illegible]
Q9NUL7	Probable ATP-dependent RNA helicase DDX28 OS=Homo sapiens GN=DDX28 PE=2 SV=2 - [DDX28_HUMAN]	20,93	7	0,769	1,054	[illegible]
P51398	28S ribosomal protein S29, mitochondrial OS=Homo sapiens GN=DAP3 PE=1 SV=1 - [RT29_HUMAN]	20,85	7	0,738	0,938	[illegible]
Q9P2R7	Succinyl-CoA ligase [ADP-forming] subunit beta, mitochondrial OS=Homo sapiens GN=SUCLA2 PE=1 SV=3 - [SUCB1_HUMAN]	20,73	7	0,828	0,731	[illegible]
Q00587	Cdc42 effector protein 1 OS=Homo sapiens GN=CDC42EP1 PE=1 SV=1 - [BORG5_HUMAN]	20,72	7	1,653	1,253	[illegible]
P55010	Eukaryotic translation initiation factor 5 OS=Homo sapiens GN=EIF5 PE=1 SV=2 - [IF5_HUMAN]	20,42	7	0,681	0,831	[illegible]
Q9Y383	Putative RNA-binding protein Luc7-like 2 OS=Homo sapiens GN=LUC7L2 PE=1 SV=2 - [LC7L2_HUMAN]	20,41	7	0,965		[illegible]
Q9NXW2	DnaJ homolog subfamily B member 12 OS=Homo sapiens GN=DNAJB12 PE=1 SV=4 - [DJB12_HUMAN]	20,27	7	1,361	1,083	[illegible]
P49591	Serine--tRNA ligase, cytoplasmic OS=Homo sapiens GN=SARS PE=1 SV=3 - [SYSC_HUMAN]	20,04	7	0,782	0,942	[illegible]
Q8TB36	Ganglioside-induced differentiation-associated protein 1 OS=Homo sapiens GN=GDAP1 PE=1 SV=3 - [GDAP1_HUMAN]	19,55	7	1,031	0,859	[illegible]
Q16630	Cleavage and polyadenylation specificity factor subunit 6 OS=Homo sapiens GN=CPSF6 PE=1 SV=2 - [CPSF6_HUMAN]	19,42	7		1,074	[illegible]
Q9NYK5	39S ribosomal protein L39, mitochondrial OS=Homo sapiens GN=MRPL39 PE=1 SV=3 - [RM39_HUMAN]	19,23	7	0,884	0,834	[illegible]
Q9Y6M1	Insulin-like growth factor 2 mRNA-binding protein 2 OS=Homo sapiens GN=IGF2BP2 PE=1 SV=2 - [IF2B2_HUMAN]	19,03	7	0,501	0,731	[illegible]
Q9Y2X3	Nucleolar protein 58 OS=Homo sapiens GN=NOP58 PE=1 SV=1 - [NOP58_HUMAN]	18,71	7	0,780	0,762	[illegible]
Q8N7H5	RNA polymerase II-associated factor 1 homolog OS=Homo sapiens GN=PAF1 PE=1 SV=2 - [PAF1_HUMAN]	18,64	7			[illegible]
P78324	Tyrosine-protein phosphatase non-receptor type substrate 1 OS=Homo sapiens GN=SIRPA PE=1 SV=2 - [SHPS1_HUMAN]	18,45	7	1,618	1,777	[illegible]
Q96N66	Lysophospholipid acyltransferase 7 OS=Homo sapiens GN=MBOAT7 PE=1 SV=2 - [MBOA7_HUMAN]	18,43	7	1,118	0,954	[illegible]
Q9NR12	PDZ and LIM domain protein 7 OS=Homo sapiens GN=PDLIM7 PE=1 SV=1 - [PDLI7_HUMAN]	18,38	7		1,620	[illegible]
Q7L0Y3	Mitochondrial ribonuclease P protein 1 OS=Homo sapiens GN=RG9MTD1 PE=1 SV=2 - [MRRP1_HUMAN]	18,11	7	0,645	0,603	[illegible]
O95232	Luc7-like protein 3 OS=Homo sapiens GN=LUC7L3 PE=1 SV=2 - [LC7L3_HUMAN]	18,06	7	0,979		[illegible]
P37198	Nuclear pore glycoprotein p62 OS=Homo sapiens GN=NUP62 PE=1 SV=3 - [NUP62_HUMAN]	18,01	7	0,759	1,048	[illegible]
Q96SK2	Transmembrane protein 209 OS=Homo sapiens GN=TMEM209 PE=1 SV=2 - [TM209_HUMAN]	18,00	7	1,453	1,149	[illegible]
Q6KB66	Keratin, type II cytoskeletal 80 OS=Homo sapiens GN=KRT80 PE=1 SV=2 - [K2C80_HUMAN]	17,92	7	0,781	0,813	[illegible]
Q9NUQ7	Ufm1-specific protease 2 OS=Homo sapiens GN=UFSP2 PE=2 SV=3 - [UFSP2_HUMAN]	17,91	7	0,869	0,861	[illegible]
Q9UHX1	Poly(U)-binding-splicing factor PUF60 OS=Homo sapiens GN=PUF60 PE=1 SV=1 - [PUF60_HUMAN]	17,89	7	0,921		[illegible]
Q14651	Plastin-1 OS=Homo sapiens GN=PLS1 PE=1 SV=2 - [PLSI_HUMAN]	17,81	7	0,856	0,962	[illegible]
Q99808	Equilibrative nucleoside transporter 1 OS=Homo sapiens GN=SLC29A1 PE=1 SV=3 - [S29A1_HUMAN]	17,76	7	2,181	2,007	[illegible]
Q9NWS8	Required for meiotic nuclear division protein 1 homolog OS=Homo sapiens GN=RMND1 PE=1 SV=2 - [RMND1_HUMAN]	17,59	7	1,611	1,433	[illegible]
Q9Y697	Cysteine desulfurase, mitochondrial OS=Homo sapiens GN=NFS1 PE=1 SV=3 - [NFS1_HUMAN]	17,51	7	0,959		[illegible]
O95793	Double-stranded RNA-binding protein Staufen homolog 1 OS=Homo sapiens GN=STAU1 PE=1 SV=2 - [STAU1_HUMAN]	17,33	7			[illegible]
Q13310	Polyadenylate-binding protein 4 OS=Homo sapiens GN=PABPC4 PE=1 SV=1 - [PABP4_HUMAN]	17,24	7	1,101	0,931	[illegible]
Q9UKM7	Endoplasmic reticulum mannosyl-oligosaccharide 1,2-alpha-mannosidase OS=Homo sapiens GN=MAN1B1 PE=1 SV=2 - [MA1B1_HUMAN]	16,88	7	1,485	1,560	[illegible]
P17858	6-phosphofructokinase, liver type OS=Homo sapiens GN=PFKL PE=1 SV=6 - [K6PL_HUMAN]	16,67	7	0,574		[illegible]
Q16181	Septin-7 OS=Homo sapiens GN=SEPT7 PE=1 SV=2 - [SEPT7_HUMAN]	16,48	7	0,996	0,919	[illegible]
Q8IXI2	Mitochondrial Rho GTPase 1 OS=Homo sapiens GN=RHOT1 PE=1 SV=2 - [MIRO1_HUMAN]	16,34	7	1,232	1,444	[illegible]
Q8N2K0	Monoacylglycerol lipase ABHD12 OS=Homo sapiens GN=ABHD12 PE=2 SV=2 - [ABD12_HUMAN]	15,83	7	1,812	1,595	[illegible]
P51114	Fragile X mental retardation syndrome-related protein 1 OS=Homo sapiens GN=FXR1 PE=1 SV=3 - [FXR1_HUMAN]	15,78	7	0,976	0,957	[illegible]
Q8NC56	LEM domain-containing protein 2 OS=Homo sapiens GN=LEMD2 PE=1 SV=1 - [LEMD2_HUMAN]	15,71	7	1,135	1,075	[illegible]
O94842	TOX high mobility group box family member 4 OS=Homo sapiens GN=TOX4 PE=1 SV=1 - [TOX4_HUMAN]	15,62	7			[illegible]
P49189	4-trimethylaminobutyraldehyde dehydrogenase OS=Homo sapiens GN=ALDH9A1 PE=1 SV=3 - [AL9A1_HUMAN]	15,59	7	0,928	0,820	[illegible]
P48723	Heat shock 70 kDa protein 13 OS=Homo sapiens GN=HSPA13 PE=1 SV=1 - [HSP13_HUMAN]	15,50	7	1,463	1,461	[illegible]
Q9NZU0	Leucine-rich repeat transmembrane protein FLRT3 OS=Homo sapiens GN=FLRT3 PE=1 SV=1 - [FLRT3_HUMAN]	15,41	7	0,613	0,477	[illegible]
Q7Z4V5	Hepatoma-derived growth factor-related protein 2 OS=Homo sapiens GN=HDGFRP2 PE=1 SV=1 - [HDGR2_HUMAN]	15,35	7			[illegible]
O15427	Monocarboxylate transporter 4 OS=Homo sapiens GN=SLC16A3 PE=1 SV=1 - [MOT4_HUMAN]	15,27	7	1,311	1,349	[illegible]
Q13505	Metaxin-1 OS=Homo sapiens GN=MTX1 PE=1 SV=2 - [MTX1_HUMAN]	15,02	7	1,083	1,089	[illegible]
Q7LGA3	Heparan sulfate 2-O-sulfotransferase 1 OS=Homo sapiens GN=HS2ST1 PE=1 SV=1 - [HS2ST_HUMAN]	14,89	7	1,457	1,286	[illegible]
O43237	Cytoplasmic dynein 1 light intermediate chain 2 OS=Homo sapiens GN=DYNC1LI2 PE=1 SV=1 - [DC1L2_HUMAN]	14,84	7	0,754	1,039	[illegible]
Q15043	Zinc transporter ZIP14 OS=Homo sapiens GN=SLC39A14 PE=1 SV=3 - [S39AE_HUMAN]	14,84	7	1,387	1,783	[illegible]
O00330	Pyruvate dehydrogenase protein X component, mitochondrial OS=Homo sapiens GN=PDHX PE=1 SV=3 - [ODPX_HUMAN]	14,57	7	1,022	0,945	[illegible]
O43264	Centromere/kinetochore protein zw10 homolog OS=Homo sapiens GN=ZW10 PE=1 SV=3 - [ZW10_HUMAN]	14,51	7	0,801	0,927	[illegible]
Q96RQ3	Methylcrotonoyl-CoA carboxylase subunit alpha, mitochondrial OS=Homo sapiens GN=MCCC1 PE=1 SV=3 - [MCCA_HUMAN]	14,21	7	0,844	0,773	[illegible]
H0YHG0	Uncharacterized protein (Fragment) OS=Homo sapiens PE=4 SV=1 - [H0YHG0_HUMAN]	14,15	7			[illegible]
H0Y4R5	Transmembrane protein 201 (Fragment) OS=Homo sapiens GN=TMEM201 PE=4 SV=1 - [H0Y4R5_HUMAN]	14,13	7	1,041	1,221	[illegible]
Q9Y679	Ancient ubiquitous protein 1 OS=Homo sapiens GN=AUP1 PE=1 SV=1 - [AUP1_HUMAN]	14,08	7	1,079	1,007	[illegible]
O94776	Metastasis-associated protein MTA2 OS=Homo sapiens GN=MTA2 PE=1 SV=1 - [MTA2_HUMAN]	14,07	7	0,990	1,105	[illegible]
P33993	DNA replication licensing factor MCM7 OS=Homo sapiens GN=MCM7 PE=1 SV=4 - [MCM7_HUMAN]	14,05	7	0,784	0,500	[illegible]
Q06210	Glucosamine--fructose-6-phosphate aminotransferase [isomerizing] 1 OS=Homo sapiens GN=GFPT1 PE=1 SV=3 - [GFPT1_HUMAN]	14,02	7	0,501	0,898	[illegible]
Q9NV06	DDB1- and CUL4-associated factor 13 OS=Homo sapiens GN=DCAF13 PE=1 SV=2 - [DCA13_HUMAN]	13,93	7	1,179		[illegible]
P38606	V-type proton ATPase catalytic subunit A OS=Homo sapiens GN=ATP6V1A PE=1 SV=2 - [VATA_HUMAN]	13,78	7	0,808	0,924	[illegible]
P47897	Glutamine--tRNA ligase OS=Homo sapiens GN=QARS PE=1 SV=1 - [SYQ_HUMAN]	13,55	7	0,669	0,804	[illegible]
Q8NBN3	Transmembrane protein 87A OS=Homo sapiens GN=TMEM87A PE=1 SV=3 - [TM87A_HUMAN]	13,51	7	1,042	0,988	[illegible]
P18564	Integrin beta-6 OS=Homo sapiens GN=ITGB6 PE=1 SV=2 - [ITB6_HUMAN]	13,45	7	0,521	0,386	[illegible]
Q96HC4	PDZ and LIM domain protein 5 OS=Homo sapiens GN=PDLIM5 PE=1 SV=5 - [PDLI5_HUMAN]	13,26	7	0,717	0,800	[illegible]
O00391	Sulfhydryl oxidase 1 OS=Homo sapiens GN=QSOX1 PE=1 SV=3 - [QSOX1_HUMAN]	13,25	7	1,015	1,077	[illegible]
Q9BQ52	Zinc phosphodiesterase ELAC protein 2 OS=Homo sapiens GN=ELAC2 PE=1 SV=2 - [RNZ2_HUMAN]	13,20	7	0,870	0,874	[illegible]
Q13772	Nuclear receptor coactivator 4 OS=Homo sapiens GN=NCOA4 PE=1 SV=1 - [NCOA4_HUMAN]	12,87	7	1,311	1,169	[illegible]
Q5QJE6	Deoxynucleotidyltransferase terminal-interacting protein 2 OS=Homo sapiens GN=DNTTIP2 PE=1 SV=2 - [TDIF2_HUMAN]	12,70	7			[illegible]
P45974	Ubiquitin carboxyl-terminal hydrolase 5 OS=Homo sapiens GN=USP5 PE=1 SV=2 - [UBP5_HUMAN]	12,59	7	0,813		[illegible]
Q96AQ6	Pre-B-cell leukemia transcription factor-interacting protein 1 OS=Homo sapiens GN=PBXIP1 PE=1 SV=1 - [PBIP1_HUMAN]	12,59	7	1,196	1,042	[illegible]
Q9BY44	Eukaryotic translation initiation factor 2A OS=Homo sapiens GN=EIF2A PE=1 SV=3 - [EIF2A_HUMAN]	12,48	7		1,115	[illegible]
P40763	Signal transducer and activator of transcription 3 OS=Homo sapiens GN=STAT3 PE=1 SV=2 - [STAT3_HUMAN]	12,34	7	0,705	0,533	[illegible]
Q8NE71	ATP-binding cassette sub-family F member 1 OS=Homo sapiens GN=ABCF1 PE=1 SV=2 - [ABCF1_HUMAN]	12,07	7	1,076	0,936	[illegible]
Q13123	Protein Red OS=Homo sapiens GN=IK PE=1 SV=3 - [RED_HUMAN]	12,03	7			[illegible]
Q8N3R9	MAGUK p55 subfamily member 5 OS=Homo sapiens GN=MPP5 PE=1 SV=3 - [MPP5_HUMAN]	12,00	7	0,986	0,991	[illegible]
Q9UBH6	Xenotropic and polytropic retrovirus receptor 1 OS=Homo sapiens GN=XPR1 PE=1 SV=1 - [XPR1_HUMAN]	11,93	7	0,938	0,821	[illegible]
Q9HBM0	Vezatin OS=Homo sapiens GN=VEZT PE=1 SV=3 - [VEZA_HUMAN]	11,81	7	1,123	1,024	[illegible]
O76094	Signal recognition particle 72 kDa protein OS=Homo sapiens GN=SRP72 PE=1 SV=3 - [SRP72_HUMAN]	11,77	7	0,792	0,797	[illegible]
P06396	Gelsolin OS=Homo sapiens GN=GSN PE=1 SV=1 - [GELS_HUMAN]	11,51	7	0,541	0,712	[illegible]
P19634	Sodium/hydrogen exchanger 1 OS=Homo sapiens GN=SLC9A1 PE=1 SV=2 - [SL9A1_HUMAN]	11,41	7	0,689	1,137	[illegible]
Q969Z0	Protein TBRG4 OS=Homo sapiens GN=TBRG4 PE=1 SV=1 - [TBRG4_HUMAN]	11,41	7	0,848	0,834	[illegible]
Q9ULQ1	Two pore calcium channel protein 1 OS=Homo sapiens GN=TPCN1 PE=1 SV=3 - [TPC1_HUMAN]	11,40	7	1,221	1,362	[illegible]
O76024	Wolframin OS=Homo sapiens GN=WFS1 PE=1 SV=2 - [WFS1_HUMAN]	11,24	7	1,803	1,019	[illegible]
Q9Y487	V-type proton ATPase 116 kDa subunit a isoform 2 OS=Homo sapiens GN=ATP6V0A2 PE=1 SV=2 - [VPP2_HUMAN]	10,98	7	1,094	1,039	[illegible]
P38435	Vitamin K-dependent gamma-carboxylase OS=Homo sapiens GN=GGCX PE=1 SV=2 - [VKGC_HUMAN]	10,95	7	1,165	1,299	[illegible]
P19022	Cadherin-2 OS=Homo sapiens GN=CDH2 PE=1 SV=4 - [CADH2_HUMAN]	10,93	7	**3,490**	**3,876**	[illegible]
A6NDB9	Paralemmin-3 OS=Homo sapiens GN=PALM3 PE=1 SV=2 - [PALM3_HUMAN]	10,85	7	0,784	0,622	[illegible]
O60266	Adenylate cyclase type 3 OS=Homo sapiens GN=ADCY3 PE=1 SV=3 - [ADCY3_HUMAN]	10,75	7	1,075	1,263	[illegible]
Q0ZGT2	Nexilin OS=Homo sapiens GN=NEXN PE=1 SV=1 - [NEXN_HUMAN]	10,52	7	1,435	1,982	[illegible]
P58107	Epiplakin OS=Homo sapiens GN=EPPK1 PE=1 SV=2 - [EPIPL_HUMAN]	10,33	7	1,273		[illegible]
Q99650	Oncostatin-M-specific receptor subunit beta OS=Homo sapiens GN=OSMR PE=1 SV=1 - [OSMR_HUMAN]	10,11	7	1,635	1,450	[illegible]
Q15042	Rab3 GTPase-activating protein catalytic subunit OS=Homo sapiens GN=RAB3GAP1 PE=1 SV=3 - [RB3GP_HUMAN]	9,68	7	0,708	0,831	[illegible]
O00267	Transcription elongation factor SPT5 OS=Homo sapiens GN=SUPT5H PE=1 SV=1 - [SPT5H_HUMAN]	9,29	7	0,743		[illegible]
O14974	Protein phosphatase 1 regulatory subunit 12A OS=Homo sapiens GN=PPP1R12A PE=1 SV=1 - [MYPT1_HUMAN]	8,93	7	0,951	1,022	[illegible]
Q68CZ2	Tensin-3 OS=Homo sapiens GN=TNS3 PE=1 SV=2 - [TENS3_HUMAN]	8,86	7	0,837	0,659	[illegible]
Q9Y2H5	Pleckstrin homology domain-containing family A member 6 OS=Homo sapiens GN=PLEKHA6 PE=1 SV=4 - [PKHA6_HUMAN]	8,68	7	0,731	0,591	[illegible]
Q9P2R3	Ankyrin repeat and FYVE domain-containing protein 1 OS=Homo sapiens GN=ANKFY1 PE=1 SV=2 - [ANFY1_HUMAN]	8,64	7	0,621		[illegible]
Q9BUQ8	Probable ATP-dependent RNA helicase DDX23 OS=Homo sapiens GN=DDX23 PE=1 SV=3 - [DDX23_HUMAN]	8,54	7	0,838	0,826	[illegible]
O94906	Pre-mRNA-processing factor 6 OS=Homo sapiens GN=PRPF6 PE=1 SV=1 - [PRP6_HUMAN]	8,50	7	0,866	0,894	[illegible]
P11498	Pyruvate carboxylase, mitochondrial OS=Homo sapiens GN=PC PE=1 SV=2 - [PYC_HUMAN]	8,49	7	0,917	0,983	[illegible]
Q9Y2W1	Thyroid hormone receptor-associated protein 3 OS=Homo sapiens GN=THRAP3 PE=1 SV=2 - [TR150_HUMAN]	8,48	7	0,827	0,811	[illegible]
O75150	E3 ubiquitin-protein ligase BRE1B OS=Homo sapiens GN=RNF40 PE=1 SV=4 - [BRE1B_HUMAN]	8,09	7			[illegible]
Q9NPR2	Semaphorin-4B OS=Homo sapiens GN=SEMA4B PE=1 SV=3 - [SEM4B_HUMAN]	8,05	7	0,458	0,405	[illegible]
Q14137	Ribosome biogenesis protein BOP1 OS=Homo sapiens GN=BOP1 PE=1 SV=2 - [BOP1_HUMAN]	8,04	7	0,780		[illegible]
Q9H6R4	Nucleolar protein 6 OS=Homo sapiens GN=NOL6 PE=1 SV=2 - [NOL6_HUMAN]	8,03	7		0,923	[illegible]
P49756	RNA-binding protein 25 OS=Homo sapiens GN=RBM25 PE=1 SV=3 - [RBM25_HUMAN]	7,95	7	0,809	0,753	[illegible]
Q5VT52	Regulation of nuclear pre-mRNA domain-containing protein 2 OS=Homo sapiens GN=RPRD2 PE=1 SV=1 - [RPRD2_HUMAN]	7,80	7			[illegible]
O95239	Chromosome-associated kinesin KIF4A OS=Homo sapiens GN=KIF4A PE=1 SV=3 - [KIF4A_HUMAN]	7,79	7	0,476		[illegible]
Q99700	Ataxin-2 OS=Homo sapiens GN=ATXN2 PE=1 SV=2 - [ATX2_HUMAN]	7,69	7		1,099	[illegible]
Q9HBR0	Putative sodium-coupled neutral amino acid transporter 10 OS=Homo sapiens GN=SLC38A10 PE=1 SV=2 - [S38AA_HUMAN]	7,60	7	0,849	0,957	[illegible]
Q96KR1	Zinc finger RNA-binding protein OS=Homo sapiens GN=ZFR PE=1 SV=2 - [ZFR_HUMAN]	7,54	7	1,134		[illegible]
Q92900	Regulator of nonsense transcripts 1 OS=Homo sapiens GN=UPF1 PE=1 SV=2 - [RENT1_HUMAN]	7,53	7	0,630	0,923	[illegible]
Q6P2E9	Enhancer of mRNA-decapping protein 4 OS=Homo sapiens GN=EDC4 PE=1 SV=1 - [EDC4_HUMAN]	7,35	7	0,868	0,652	[illegible]
Q9C0J8	pre-mRNA 3' end processing protein WDR33 OS=Homo sapiens GN=WDR33 PE=1 SV=2 - [WDR33_HUMAN]	7,26	7		1,263	[illegible]
Q01973	Tyrosine-protein kinase transmembrane receptor ROR1 OS=Homo sapiens GN=ROR1 PE=2 SV=2 - [ROR1_HUMAN]	6,94	7	1,106	1,250	[illegible]
P78357	Contactin-associated protein 1 OS=Homo sapiens GN=CNTNAP1 PE=1 SV=1 - [CNTP1_HUMAN]	6,86	7	1,991	2,026	[illegible]
P30622	CAP-Gly domain-containing linker protein 1 OS=Homo sapiens GN=CLIP1 PE=1 SV=2 - [CLIP1_HUMAN]	6,68	7	1,129		[illegible]
Q9NTJ3	Structural maintenance of chromosomes protein 4 OS=Homo sapiens GN=SMC4 PE=1 SV=2 - [SMC4_HUMAN]	5,98	7	0,883	0,895	[illegible]
Q9BY89	Uncharacterized protein KIAA1671 OS=Homo sapiens GN=KIAA1671 PE=1 SV=2 - [K1671_HUMAN]	5,70	7			[illegible]
Q7LBC6	Lysine-specific demethylase 3B OS=Homo sapiens GN=KDM3B PE=1 SV=2 - [KDM3B_HUMAN]	5,45	7	0,693	0,974	[illegible]
Q5VZK9	Leucine-rich repeat-containing protein 16A OS=Homo sapiens GN=LRRC16A PE=1 SV=1 - [LR16A_HUMAN]	5,32	7	1,077	0,924	[illegible]
Q7KZ85	Transcription elongation factor SPT6 OS=Homo sapiens GN=SUPT6H PE=1 SV=2 - [SPT6H_HUMAN]	4,75	7	1,147	1,134	[illegible]
O14578	Citron Rho-interacting kinase OS=Homo sapiens GN=CIT PE=1 SV=2 - [CTRO_HUMAN]	4,49	7	0,872	0,813	[illegible]
P51532	Transcription activator BRG1 OS=Homo sapiens GN=SMARCA4 PE=1 SV=2 - [SMCA4_HUMAN]	4,37	7		1,060	[illegible]
P42345	Serine/threonine-protein kinase mTOR OS=Homo sapiens GN=MTOR PE=1 SV=1 - [MTOR_HUMAN]	3,65	7	1,089	1,292	[illegible]
Q9NYQ6	Cadherin EGF LAG seven-pass G-type receptor 1 OS=Homo sapiens GN=CELSR1 PE=1 SV=1 - [CELR1_HUMAN]	3,45	7	1,170	1,282	[illegible]
P62820	Ras-related protein Rab-1A OS=Homo sapiens GN=RAB1A PE=1 SV=3 - [RAB1A_HUMAN]	74,15	6	1,052	1,037	[illegible]
Q15388	Mitochondrial import receptor subunit TOM20 homolog OS=Homo sapiens GN=TOMM20 PE=1 SV=1 - [TOM20_HUMAN]	64,83	6	1,117	**2,140**	[illegible]
P49006	MARCKS-related protein OS=Homo sapiens GN=MARCKSL1 PE=1 SV=2 - [MRP_HUMAN]	64,10	6	1,521	1,432	[illegible]
P14854	Cytochrome c oxidase subunit 6B1 OS=Homo sapiens GN=COX6B1 PE=1 SV=2 - [CX6B1_HUMAN]	62,79	6	1,634	1,424	[illegible]
Q16891	Mitochondrial inner membrane protein OS=Homo sapiens GN=IMMT PE=1 SV=1 - [IMMT_HUMAN]	61,21	6	1,421	1,309	[illegible]
P05204	Non-histone chromosomal protein HMG-17 OS=Homo sapiens GN=HMGN2 PE=1 SV=3 - [HMGN2_HUMAN]	61,11	6			[illegible]
P0CW22;P08708	40S ribosomal protein S17-like OS=Homo sapiens GN=RPS17L PE=3 SV=1 - [RS17L_HUMAN]	60,00	6	0,994	0,993	[illegible]
P51148	Ras-related protein Rab-5C OS=Homo sapiens GN=RAB5C PE=1 SV=2 - [RAB5C_HUMAN]	56,94	6	1,264	1,331	[illegible]
P31946	14-3-3 protein beta/alpha OS=Homo sapiens GN=YWHAB PE=1 SV=3 - [1433B_HUMAN]	56,50	6	0,964	0,905	[illegible]
P20339	Ras-related protein Rab-5A OS=Homo sapiens GN=RAB5A PE=1 SV=2 - [RAB5A_HUMAN]	53,95	6	1,288	1,358	[illegible]
P61020	Ras-related protein Rab-5B OS=Homo sapiens GN=RAB5B PE=1 SV=1 - [RAB5B_HUMAN]	53,95	6	1,763	1,576	[illegible]
P11234	Ras-related protein Ral-B OS=Homo sapiens GN=RALB PE=1 SV=1 - [RALB_HUMAN]	53,88	6	0,764	0,664	[illegible]
P00167	Cytochrome b5 OS=Homo sapiens GN=CYB5A PE=1 SV=2 - [CYB5_HUMAN]	53,73	6	0,555	0,522	[illegible]
P56385	ATP synthase subunit e, mitochondrial OS=Homo sapiens GN=ATP5I PE=1 SV=2 - [ATP5I_HUMAN]	53,62	6	1,162	1,132	[illegible]
P20337	Ras-related protein Rab-3B OS=Homo sapiens GN=RAB3B PE=1 SV=2 - [RAB3B_HUMAN]	52,97	6	1,089	1,161	[illegible]
Q5TEC6	Histone H3 OS=Homo sapiens GN=HIST2H3PS2 PE=3 SV=1 - [Q5TEC6_HUMAN]	52,94	6		1,259	[illegible]

P17568	NADH dehydrogenase [ubiquinone] 1 beta subcomplex subunit 7 OS=Homo sapiens GN=NDUFB7 PE=1 SV=4 - [NDUB7_HUMAN]	51,09	6
P11233	Ras-related protein Ral-A OS=Homo sapiens GN=RALA PE=1 SV=1 - [RALA_HUMAN]	49,51	6
Q9Y3E5	Peptidyl-tRNA hydrolase 2, mitochondrial OS=Homo sapiens GN=PTRH2 PE=1 SV=1 - [PTH2_HUMAN]	48,60	6
Q6IAA8	Ragulator complex protein LAMTOR1 OS=Homo sapiens GN=LAMTOR1 PE=1 SV=2 - [LTOR1_HUMAN]	47,83	6
P61981	14-3-3 protein gamma OS=Homo sapiens GN=YWHAG PE=1 SV=2 - [1433G_HUMAN]	46,15	6
P53999	Activated RNA polymerase II transcriptional coactivator p15 OS=Homo sapiens GN=SUB1 PE=1 SV=3 - [TCP4_HUMAN]	44,88	6
P82912	28S ribosomal protein S11, mitochondrial OS=Homo sapiens GN=MRPS11 PE=1 SV=2 - [RT11_HUMAN]	44,85	6
Q7Z7H5	Transmembrane emp24 domain-containing protein 4 OS=Homo sapiens GN=TMED4 PE=1 SV=1 - [TMED4_HUMAN]	44,49	6
P62829	60S ribosomal protein L23 OS=Homo sapiens GN=RPL23 PE=1 SV=1 - [RL23_HUMAN]	44,29	6
P57105	Synaptojanin-2-binding protein OS=Homo sapiens GN=SYNJ2BP PE=1 SV=2 - [SYJ2B_HUMAN]	42,76	6
Q9Y3C1	Nucleolar protein 16 OS=Homo sapiens GN=NOP16 PE=1 SV=2 - [NOP16_HUMAN]	42,70	6
Q5W111	SPRY domain-containing protein 7 OS=Homo sapiens GN=SPRYD7 PE=1 SV=2 - [SPRY7_HUMAN]	41,84	6
Q9H9B4	Sideroflexin-1 OS=Homo sapiens GN=SFXN1 PE=1 SV=4 - [SFXN1_HUMAN]	40,99	6
Q13637	Ras-related protein Rab-32 OS=Homo sapiens GN=RAB32 PE=1 SV=3 - [RAB32_HUMAN]	39,56	6
Q05639	Elongation factor 1-alpha 2 OS=Homo sapiens GN=EEF1A2 PE=1 SV=1 - [EF1A2_HUMAN]	39,52	6
Q9BVK6	Transmembrane emp24 domain-containing protein 9 OS=Homo sapiens GN=TMED9 PE=1 SV=2 - [TMED9_HUMAN]	38,30	6
P28066	Proteasome subunit alpha type-5 OS=Homo sapiens GN=PSMA5 PE=1 SV=3 - [PSA5_HUMAN]	37,76	6
P31943	Heterogeneous nuclear ribonucleoprotein H OS=Homo sapiens GN=HNRNPH1 PE=1 SV=4 - [HNRH1_HUMAN]	37,42	6
P61081	NEDD8-conjugating enzyme Ubc12 OS=Homo sapiens GN=UBE2M PE=1 SV=1 - [UBC12_HUMAN]	37,16	6
P61927	60S ribosomal protein L37 OS=Homo sapiens GN=RPL37 PE=1 SV=2 - [RL37_HUMAN]	37,11	6
P56537	Eukaryotic translation initiation factor 6 OS=Homo sapiens GN=EIF6 PE=1 SV=1 - [IF6_HUMAN]	36,73	6
P07741	Adenine phosphoribosyltransferase OS=Homo sapiens GN=APRT PE=1 SV=2 - [APT_HUMAN]	36,67	6
P08238	Heat shock protein HSP 90-beta OS=Homo sapiens GN=HSP90AB1 PE=1 SV=4 - [HS90B_HUMAN]	36,46	6
P62249	40S ribosomal protein S16 OS=Homo sapiens GN=RPS16 PE=1 SV=2 - [RS16_HUMAN]	36,30	6
P02794	Ferritin heavy chain OS=Homo sapiens GN=FTH1 PE=1 SV=2 - [FRIH_HUMAN]	36,07	6
Q9P0J0	NADH dehydrogenase [ubiquinone] 1 alpha subcomplex subunit 13 OS=Homo sapiens GN=NDUFA13 PE=1 SV=3 - [NDUAD_HUMAN]	35,42	6
Q9Y2Q3	Glutathione S-transferase kappa 1 OS=Homo sapiens GN=GSTK1 PE=1 SV=3 - [GSTK1_HUMAN]	35,40	6
O43402	Neighbor of COX4 OS=Homo sapiens GN=COX4NB PE=1 SV=1 - [CX4NB_HUMAN]	35,24	6
P62899	60S ribosomal protein L31 OS=Homo sapiens GN=RPL31 PE=1 SV=1 - [RL31_HUMAN]	35,20	6
Q9GZZ1	N-alpha-acetyltransferase 50 OS=Homo sapiens GN=NAA50 PE=1 SV=1 - [NAA50_HUMAN]	34,91	6
Q14019	Coactosin-like protein OS=Homo sapiens GN=COTL1 PE=1 SV=3 - [COTL1_HUMAN]	34,51	6
Q9UEU0	Vesicle transport through interaction with t-SNAREs homolog 1B OS=Homo sapiens GN=VTI1B PE=1 SV=3 - [VTI1B_HUMAN]	34,48	6
O43819	Protein SCO2 homolog, mitochondrial OS=Homo sapiens GN=SCO2 PE=1 SV=3 - [SCO2_HUMAN]	33,83	6
P46782	40S ribosomal protein S5 OS=Homo sapiens GN=RPS5 PE=1 SV=4 - [RS5_HUMAN]	33,82	6
P32119	Peroxiredoxin-2 OS=Homo sapiens GN=PRDX2 PE=1 SV=5 - [PRDX2_HUMAN]	32,83	6
Q6IQ22	Ras-related protein Rab-12 OS=Homo sapiens GN=RAB12 PE=1 SV=3 - [RAB12_HUMAN]	32,79	6
Q96A26	Protein FAM162A OS=Homo sapiens GN=FAM162A PE=1 SV=2 - [F162A_HUMAN]	32,47	6
O96011	Peroxisomal membrane protein 11B OS=Homo sapiens GN=PEX11B PE=1 SV=1 - [PX11B_HUMAN]	32,43	6
P33316	Deoxyuridine 5'-triphosphate nucleotidohydrolase, mitochondrial OS=Homo sapiens GN=DUT PE=1 SV=4 - [DUT_HUMAN]	32,14	6
Q99618	Cell division cycle-associated protein 3 OS=Homo sapiens GN=CDCA3 PE=1 SV=1 - [CDCA3_HUMAN]	32,09	6
Q8WVC6	Dephospho-CoA kinase domain-containing protein OS=Homo sapiens GN=DCAKD PE=1 SV=1 - [DCAKD_HUMAN]	31,17	6
P00403	Cytochrome c oxidase subunit 2 OS=Homo sapiens GN=MT-CO2 PE=1 SV=1 - [COX2_HUMAN]	30,84	6
P46779	60S ribosomal protein L28 OS=Homo sapiens GN=RPL28 PE=1 SV=3 - [RL28_HUMAN]	30,66	6
P30085	UMP-CMP kinase OS=Homo sapiens GN=CMPK1 PE=1 SV=3 - [KCY_HUMAN]	30,61	6
P51809	Vesicle-associated membrane protein 7 OS=Homo sapiens GN=VAMP7 PE=1 SV=3 - [VAMP7_HUMAN]	30,45	6
P43307	Translocon-associated protein subunit alpha OS=Homo sapiens GN=SSR1 PE=1 SV=3 - [SSRA_HUMAN]	30,42	6
P62851	40S ribosomal protein S25 OS=Homo sapiens GN=RPS25 PE=1 SV=1 - [RS25_HUMAN]	30,40	6
Q9UMS0	NFU1 iron-sulfur cluster scaffold homolog, mitochondrial OS=Homo sapiens GN=NFU1 PE=1 SV=2 - [NFU1_HUMAN]	30,31	6
Q04323	UBX domain-containing protein 1 OS=Homo sapiens GN=UBXN1 PE=1 SV=2 - [UBXN1_HUMAN]	29,97	6
P04179	Superoxide dismutase [Mn], mitochondrial OS=Homo sapiens GN=SOD2 PE=1 SV=2 - [SODM_HUMAN]	29,73	6
P49755	Transmembrane emp24 domain-containing protein 10 OS=Homo sapiens GN=TMED10 PE=1 SV=2 - [TMEDA_HUMAN]	29,68	6
Q15526	Surfeit locus protein 1 OS=Homo sapiens GN=SURF1 PE=1 SV=1 - [SURF1_HUMAN]	28,33	6
Q12846	Syntaxin-4 OS=Homo sapiens GN=STX4 PE=1 SV=2 - [STX4_HUMAN]	28,28	6
P42166	Lamina-associated polypeptide 2, isoform alpha OS=Homo sapiens GN=TMPO PE=1 SV=2 - [LAP2A_HUMAN]	27,95	6
O14745	Na(+)/H(+) exchange regulatory cofactor NHE-RF1 OS=Homo sapiens GN=SLC9A3R1 PE=1 SV=4 - [NHRF1_HUMAN]	27,93	6
Q5ZPR3	CD276 antigen OS=Homo sapiens GN=CD276 PE=1 SV=1 - [CD276_HUMAN]	27,53	6
O15260	Surfeit locus protein 4 OS=Homo sapiens GN=SURF4 PE=1 SV=3 - [SURF4_HUMAN]	27,51	6
Q8N9F7	Glycerophosphodiester phosphodiesterase domain-containing protein 1 OS=Homo sapiens GN=GDPD1 PE=1 SV=2 - [GDPD1_HUMAN]	27,07	6
P12931	Proto-oncogene tyrosine-protein kinase Src OS=Homo sapiens GN=SRC PE=1 SV=3 - [SRC_HUMAN]	27,05	6
E7EVA0	Microtubule-associated protein OS=Homo sapiens GN=MAP4 PE=4 SV=1 - [E7EVA0_HUMAN]	26,86	6
Q92522	Histone H1x OS=Homo sapiens GN=H1FX PE=1 SV=1 - [H1X_HUMAN]	26,29	6
Q9NUM4	Transmembrane protein 106B OS=Homo sapiens GN=TMEM106B PE=1 SV=2 - [T106B_HUMAN]	26,28	6
Q9NVV0	Trimeric intracellular cation channel type B OS=Homo sapiens GN=TMEM38B PE=1 SV=1 - [TM38B_HUMAN]	26,12	6
P30048	Thioredoxin-dependent peroxide reductase, mitochondrial OS=Homo sapiens GN=PRDX3 PE=1 SV=3 - [PRDX3_HUMAN]	25,78	6
Q8IY95	Transmembrane protein 192 OS=Homo sapiens GN=TMEM192 PE=1 SV=1 - [TM192_HUMAN]	25,46	6
Q96C19	EF-hand domain-containing protein D2 OS=Homo sapiens GN=EFHD2 PE=1 SV=1 - [EFHD2_HUMAN]	25,42	6
P31431	Syndecan-4 OS=Homo sapiens GN=SDC4 PE=1 SV=2 - [SDC4_HUMAN]	25,25	6
P35249	Replication factor C subunit 4 OS=Homo sapiens GN=RFC4 PE=1 SV=2 - [RFC4_HUMAN]	25,07	6
Q96EP5	DAZ-associated protein 1 OS=Homo sapiens GN=DAZAP1 PE=1 SV=1 - [DAZP1_HUMAN]	25,06	6
Q8NBJ7	Sulfatase-modifying factor 2 OS=Homo sapiens GN=SUMF2 PE=1 SV=2 - [SUMF2_HUMAN]	24,92	6
P53597	Succinyl-CoA ligase [ADP/GDP-forming] subunit alpha, mitochondrial OS=Homo sapiens GN=SUCLG1 PE=1 SV=4 - [SUCA_HUMAN]	24,86	6
O75477	Erlin-1 OS=Homo sapiens GN=ERLIN1 PE=1 SV=1 - [ERLN1_HUMAN]	24,86	6
O00560	Syntenin-1 OS=Homo sapiens GN=SDCBP PE=1 SV=1 - [SDCB1_HUMAN]	24,83	6
Q9Y3A5	Ribosome maturation protein SBDS OS=Homo sapiens GN=SBDS PE=1 SV=4 - [SBDS_HUMAN]	24,80	6
P06493	Cyclin-dependent kinase 1 OS=Homo sapiens GN=CDK1 PE=1 SV=3 - [CDK1_HUMAN]	24,58	6
Q07021	Complement component 1 Q subcomponent-binding protein, mitochondrial OS=Homo sapiens GN=C1QBP PE=1 SV=1 - [C1QBP_HUMAN	24,11	6
Q9BYG5	Partitioning defective 6 homolog beta OS=Homo sapiens GN=PARD6B PE=1 SV=1 - [PAR6B_HUMAN]	23,92	6
P08962	CD63 antigen OS=Homo sapiens GN=CD63 PE=1 SV=2 - [CD63_HUMAN]	23,53	6
Q96ND0	Protein FAM210A OS=Homo sapiens GN=FAM210A PE=2 SV=2 - [F210A_HUMAN]	23,16	6
Q96BZ9	TBC1 domain family member 20 OS=Homo sapiens GN=TBC1D20 PE=1 SV=1 - [TBC20_HUMAN]	23,08	6
P31153	S-adenosylmethionine synthase isoform type-2 OS=Homo sapiens GN=MAT2A PE=1 SV=1 - [METK2_HUMAN]	23,04	6
Q8TC12	Retinol dehydrogenase 11 OS=Homo sapiens GN=RDH11 PE=1 SV=2 - [RDH11_HUMAN]	22,96	6
O96013	Serine/threonine-protein kinase PAK 4 OS=Homo sapiens GN=PAK4 PE=1 SV=1 - [PAK4_HUMAN]	22,67	6
O76003	Glutaredoxin-3 OS=Homo sapiens GN=GLRX3 PE=1 SV=2 - [GLRX3_HUMAN]	22,39	6
P16260	Graves disease carrier protein OS=Homo sapiens GN=SLC25A16 PE=2 SV=3 - [GDC_HUMAN]	21,99	6
Q9NYL9	Tropomodulin-3 OS=Homo sapiens GN=TMOD3 PE=1 SV=1 - [TMOD3_HUMAN]	21,88	6
Q9HC07	Transmembrane protein 165 OS=Homo sapiens GN=TMEM165 PE=1 SV=1 - [TM165_HUMAN]	21,60	6
P43007	Neutral amino acid transporter A OS=Homo sapiens GN=SLC1A4 PE=1 SV=1 - [SATT_HUMAN]	21,43	6
O94760	N(G),N(G)-dimethylarginine dimethylaminohydrolase 1 OS=Homo sapiens GN=DDAH1 PE=1 SV=3 - [DDAH1_HUMAN]	21,40	6
P27695	DNA-(apurinic or apyrimidinic site) lyase OS=Homo sapiens GN=APEX1 PE=1 SV=2 - [APEX1_HUMAN]	21,38	6
Q9UM00	Transmembrane and coiled-coil domain-containing protein 1 OS=Homo sapiens GN=TMCO1 PE=1 SV=1 - [TMCO1_HUMAN]	21,28	6
O15446	DNA-directed RNA polymerase I subunit RPA34 OS=Homo sapiens GN=CD3EAP PE=1 SV=1 - [RPA34_HUMAN]	21,18	6
Q6P1Q0	LETM1 domain-containing protein 1 OS=Homo sapiens GN=LETMD1 PE=1 SV=1 - [LTMD1_HUMAN]	21,11	6
P15291	Beta-1,4-galactosyltransferase 1 OS=Homo sapiens GN=B4GALT1 PE=1 SV=5 - [B4GT1_HUMAN]	21,11	6
Q96G23	Ceramide synthase 2 OS=Homo sapiens GN=CERS2 PE=1 SV=1 - [CERS2_HUMAN]	21,05	6
Q9Y295	Developmentally-regulated GTP-binding protein 1 OS=Homo sapiens GN=DRG1 PE=1 SV=1 - [DRG1_HUMAN]	20,98	6
Q92890	Ubiquitin fusion degradation protein 1 homolog OS=Homo sapiens GN=UFD1L PE=1 SV=3 - [UFD1_HUMAN]	20,85	6
Q6P4A7	Sideroflexin-4 OS=Homo sapiens GN=SFXN4 PE=1 SV=1 - [SFXN4_HUMAN]	20,77	6
Q9UQ53	Alpha-1,3-mannosyl-glycoprotein 4-beta-N-acetylglucosaminyltransferase B OS=Homo sapiens GN=MGAT4B PE=1 SV=1 - [MGT4B_HUM	20,62	6
P11177	Pyruvate dehydrogenase E1 component subunit beta, mitochondrial OS=Homo sapiens GN=PDHB PE=1 SV=3 - [ODPB_HUMAN]	20,33	6
P25685	DnaJ homolog subfamily B member 1 OS=Homo sapiens GN=DNAJB1 PE=1 SV=4 - [DNJB1_HUMAN]	20,29	6
Q92552	28S ribosomal protein S27, mitochondrial OS=Homo sapiens GN=MRPS27 PE=1 SV=3 - [RT27_HUMAN]	20,05	6
O75071	EF-hand domain-containing protein KIAA0494 OS=Homo sapiens GN=KIAA0494 PE=1 SV=1 - [K0494_HUMAN]	20,00	6
Q9UJM3	ERBB receptor feedback inhibitor 1 OS=Homo sapiens GN=ERRFI1 PE=1 SV=1 - [ERRFI_HUMAN]	19,91	6
P56962	Syntaxin-17 OS=Homo sapiens GN=STX17 PE=1 SV=2 - [STX17_HUMAN]	19,87	6
P08559	Pyruvate dehydrogenase E1 component subunit alpha, somatic form, mitochondrial OS=Homo sapiens GN=PDHA1 PE=1 SV=3 - [ODPA_	19,23	6
Q9BQ95	Evolutionarily conserved signaling intermediate in Toll pathway, mitochondrial OS=Homo sapiens GN=ECSIT PE=1 SV=1 - [ECSIT_HUMA	19,03	6
O75937	DnaJ homolog subfamily C member 8 OS=Homo sapiens GN=DNAJC8 PE=1 SV=2 - [DNJC8_HUMAN]	18,97	6
Q13418	Integrin-linked protein kinase OS=Homo sapiens GN=ILK PE=1 SV=2 - [ILK_HUMAN]	18,81	6
Q9Y3B9	RRP15-like protein OS=Homo sapiens GN=RRP15 PE=1 SV=2 - [RRP15_HUMAN]	18,79	6
P09496	Clathrin light chain A OS=Homo sapiens GN=CLTA PE=1 SV=1 - [CLCA_HUMAN]	18,55	6
Q9UMS4	Pre-mRNA-processing factor 19 OS=Homo sapiens GN=PRPF19 PE=1 SV=1 - [PRP19_HUMAN]	18,45	6
Q9NX58	Cell growth-regulating nucleolar protein OS=Homo sapiens GN=LYAR PE=1 SV=2 - [LYAR_HUMAN]	18,21	6
O15533	Tapasin OS=Homo sapiens GN=TAPBP PE=1 SV=1 - [TPSN_HUMAN]	17,86	6
P50402	Emerin OS=Homo sapiens GN=EMD PE=1 SV=1 - [EMD_HUMAN]	17,72	6
Q8NE86	Calcium uniporter protein, mitochondrial OS=Homo sapiens GN=MCU PE=1 SV=1 - [MCU_HUMAN]	17,66	6
P17706	Tyrosine-protein phosphatase non-receptor type 2 OS=Homo sapiens GN=PTPN2 PE=1 SV=2 - [PTN2_HUMAN]	17,59	6
Q8TDN6	Ribosome biogenesis protein BRX1 homolog OS=Homo sapiens GN=BRIX1 PE=1 SV=2 - [BRX1_HUMAN]	17,56	6
Q7Z5L9	Interferon regulatory factor 2-binding protein 2 OS=Homo sapiens GN=IRF2BP2 PE=1 SV=2 - [I2BP2_HUMAN]	17,38	6
Q9H6F5	Coiled-coil domain-containing protein 86 OS=Homo sapiens GN=CCDC86 PE=1 SV=1 - [CCD86_HUMAN]	17,22	6
Q9Y2R4	Probable ATP-dependent RNA helicase DDX52 OS=Homo sapiens GN=DDX52 PE=1 SV=3 - [DDX52_HUMAN]	17,20	6
Q7L5A8	Fatty acid 2-hydroxylase OS=Homo sapiens GN=FA2H PE=1 SV=1 - [FA2H_HUMAN]	17,20	6
Q8N684	Cleavage and polyadenylation specificity factor subunit 7 OS=Homo sapiens GN=CPSF7 PE=1 SV=1 - [CPSF7_HUMAN]	17,20	6
Q9UN86-2	Isoform B of Ras GTPase-activating protein-binding protein 2 OS=Homo sapiens GN=G3BP2 - [G3BP2_HUMAN]	17,15	6
Q8N8A6	ATP-dependent RNA helicase DDX51 OS=Homo sapiens GN=DDX51 PE=1 SV=3 - [DDX51_HUMAN]	17,12	6
P43034	Platelet-activating factor acetylhydrolase IB subunit alpha OS=Homo sapiens GN=PAFAH1B1 PE=1 SV=2 - [LIS1_HUMAN]	16,83	6
Q96EY1	DnaJ homolog subfamily A member 3, mitochondrial OS=Homo sapiens GN=DNAJA3 PE=1 SV=2 - [DNJA3_HUMAN]	16,67	6
Q96HE7	ERO1-like protein alpha OS=Homo sapiens GN=ERO1L PE=1 SV=2 - [ERO1A_HUMAN]	16,67	6
Q7KZN9	Cytochrome c oxidase assembly protein COX15 homolog OS=Homo sapiens GN=COX15 PE=1 SV=1 - [COX15_HUMAN]	16,59	6
Q9BQP7	Uncharacterized protein C20orf72 OS=Homo sapiens GN=C20orf72 PE=1 SV=1 - [CT072_HUMAN]	16,57	6
O60906	Sphingomyelin phosphodiesterase 2 OS=Homo sapiens GN=SMPD2 PE=1 SV=2 - [NSMA_HUMAN]	16,55	6
P35659	Protein DEK OS=Homo sapiens GN=DEK PE=1 SV=1 - [DEK_HUMAN]	16,27	6
P56182	Ribosomal RNA processing protein 1 homolog A OS=Homo sapiens GN=RRP1 PE=1 SV=1 - [RRP1_HUMAN]	16,05	6
P50395	Rab GDP dissociation inhibitor beta OS=Homo sapiens GN=GDI2 PE=1 SV=2 - [GDIB_HUMAN]	15,96	6
Q9NRZ7	1-acyl-sn-glycerol-3-phosphate acyltransferase gamma OS=Homo sapiens GN=AGPAT3 PE=1 SV=1 - [PLCC_HUMAN]	15,96	6
Q15067	Peroxisomal acyl-coenzyme A oxidase 1 OS=Homo sapiens GN=ACOX1 PE=1 SV=3 - [ACOX1_HUMAN]	15,91	6
Q96E52	Metalloendopeptidase OMA1, mitochondrial OS=Homo sapiens GN=OMA1 PE=2 SV=1 - [OMA1_HUMAN]	15,84	6
Q96AC1	Fermitin family homolog 2 OS=Homo sapiens GN=FERMT2 PE=1 SV=1 - [FERM2_HUMAN]	15,74	6
P33240	Cleavage stimulation factor subunit 2 OS=Homo sapiens GN=CSTF2 PE=1 SV=1 - [CSTF2_HUMAN]	15,42	6
P49419	Alpha-aminoadipic semialdehyde dehydrogenase OS=Homo sapiens GN=ALDH7A1 PE=1 SV=5 - [AL7A1_HUMAN]	15,40	6
P26440	Isovaleryl-CoA dehydrogenase, mitochondrial OS=Homo sapiens GN=IVD PE=1 SV=1 - [IVD_HUMAN]	15,37	6
Q16401	26S proteasome non-ATPase regulatory subunit 5 OS=Homo sapiens GN=PSMD5 PE=1 SV=3 - [PSMD5_HUMAN]	15,28	6
Q9Y282	Endoplasmic reticulum-Golgi intermediate compartment protein 3 OS=Homo sapiens GN=ERGIC3 PE=1 SV=1 - [ERGI3_HUMAN]	15,14	6
P82675	28S ribosomal protein S5, mitochondrial OS=Homo sapiens GN=MRPS5 PE=1 SV=2 - [RT05_HUMAN]	15,12	6
Q9NS86	LanC-like protein 2 OS=Homo sapiens GN=LANCL2 PE=1 SV=1 - [LANC2_HUMAN]	15,11	6
Q16851	UTP--glucose-1-phosphate uridylyltransferase OS=Homo sapiens GN=UGP2 PE=1 SV=5 - [UGPA_HUMAN]	14,96	6
Q9BWS9	Chitinase domain-containing protein 1 OS=Homo sapiens GN=CHID1 PE=1 SV=1 - [CHID1_HUMAN]	14,76	6
P11182	Lipoamide acyltransferase component of branched-chain alpha-keto acid dehydrogenase complex, mitochondrial OS=Homo sapiens GN=[	14,52	6
Q9BZE1	39S ribosomal protein L37, mitochondrial OS=Homo sapiens GN=MRPL37 PE=1 SV=2 - [RM37_HUMAN]	14,42	6
O96019	Actin-like protein 6A OS=Homo sapiens GN=ACTL6A PE=1 SV=1 - [ACL6A_HUMAN]	13,99	6
Q13137	Calcium-binding and coiled-coil domain-containing protein 2 OS=Homo sapiens GN=CALCOCO2 PE=1 SV=1 - [CACO2_HUMAN]	13,90	6
Q9NP81	Serine--tRNA ligase, mitochondrial OS=Homo sapiens GN=SARS2 PE=1 SV=1 - [SYSM_HUMAN]	13,90	6
Q8TB61	Adenosine 3'-phospho 5'-phosphosulfate transporter 1 OS=Homo sapiens GN=SLC35B2 PE=1 SV=1 - [S35B2_HUMAN]	13,89	6
Q13601	KRR1 small subunit processome component homolog OS=Homo sapiens GN=KRR1 PE=1 SV=4 - [KRR1_HUMAN]	13,65	6
Q02809	Procollagen-lysine,2-oxoglutarate 5-dioxygenase 1 OS=Homo sapiens GN=PLOD1 PE=1 SV=2 - [PLOD1_HUMAN]	13,62	6
O75131	Copine-3 OS=Homo sapiens GN=CPNE3 PE=1 SV=1 - [CPNE3_HUMAN]	13,59	6
Q9Y426	C2 domain-containing protein 2 OS=Homo sapiens GN=C2CD2 PE=1 SV=2 - [CU025_HUMAN]	13,51	6
Q9H8Y8	Golgi reassembly-stacking protein 2 OS=Homo sapiens GN=GORASP2 PE=1 SV=3 - [GORS2_HUMAN]	13,50	6
P58335	Anthrax toxin receptor 2 OS=Homo sapiens GN=ANTXR2 PE=1 SV=5 - [ANTR2_HUMAN]	13,29	6
P51798	H(+)/Cl(-) exchange transporter 7 OS=Homo sapiens GN=CLCN7 PE=1 SV=2 - [CLCN7_HUMAN]	13,29	6
P60228	Eukaryotic translation initiation factor 3 subunit E OS=Homo sapiens GN=EIF3E PE=1 SV=1 - [EIF3E_HUMAN]	13,26	6
Q9BTT6	Leucine-rich repeat-containing protein 1 OS=Homo sapiens GN=LRRC1 PE=1 SV=1 - [LRRC1_HUMAN]	13,17	6
Q9UBM7	7-dehydrocholesterol reductase OS=Homo sapiens GN=DHCR7 PE=1 SV=1 - [DHCR7_HUMAN]	13,05	6
P25490	Transcriptional repressor protein YY1 OS=Homo sapiens GN=YY1 PE=1 SV=2 - [TYY1_HUMAN]	13,04	6
Q86SJ2	Amphoterin-induced protein 2 OS=Homo sapiens GN=AMIGO2 PE=1 SV=1 - [AMGO2_HUMAN]	13,03	6
Q9NY61	Protein AATF OS=Homo sapiens GN=AATF PE=1 SV=1 - [AATF_HUMAN]	12,86	6
Q9H0B6	Kinesin light chain 2 OS=Homo sapiens GN=KLC2 PE=1 SV=1 - [KLC2_HUMAN]	12,54	6
Q9NY33	Dipeptidyl peptidase 3 OS=Homo sapiens GN=DPP3 PE=1 SV=2 - [DPP3_HUMAN]	12,35	6
O95340	Bifunctional 3'-phosphoadenosine 5'-phosphosulfate synthase 2 OS=Homo sapiens GN=PAPSS2 PE=1 SV=2 - [PAPS2_HUMAN]	12,21	6
Q96B97	SH3 domain-containing kinase-binding protein 1 OS=Homo sapiens GN=SH3KBP1 PE=1 SV=2 - [SH3K1_HUMAN]	11,88	6

P19525	Interferon-induced, double-stranded RNA-activated protein kinase OS=Homo sapiens GN=EIF2AK2 PE=1 SV=2 - [E2AK2_HUMAN]	11,80	6	0,377	0,393	[illegible]
Q01844	RNA-binding protein EWS OS=Homo sapiens GN=EWSR1 PE=1 SV=1 - [EWS_HUMAN]	11,59	6	1,172	1,227	[illegible]
Q9NYY8	FAST kinase domain-containing protein 2 OS=Homo sapiens GN=FASTKD2 PE=1 SV=1 - [FAKD2_HUMAN]	11,55	6	0,777	0,748	[illegible]
P56945	Breast cancer anti-estrogen resistance protein 1 OS=Homo sapiens GN=BCAR1 PE=1 SV=2 - [BCAR1_HUMAN]	11,49	6	0,979	0,681	[illegible]
Q5T9L3	Protein wntless homolog OS=Homo sapiens GN=WLS PE=1 SV=2 - [WLS_HUMAN]	11,46	6	0,874	0,790	[illegible]
Q99442	Translocation protein SEC62 OS=Homo sapiens GN=SEC62 PE=1 SV=1 - [SEC62_HUMAN]	11,28	6	1,385	1,419	[illegible]
Q96BD0	Solute carrier organic anion transporter family member 4A1 OS=Homo sapiens GN=SLCO4A1 PE=1 SV=2 - [SO4A1_HUMAN]	11,22	6	1,074	0,986	[illegible]
O15381	Nuclear valosin-containing protein-like OS=Homo sapiens GN=NVL PE=1 SV=1 - [NVL_HUMAN]	11,21	6	0,974		[illegible]
Q9BYC5	Alpha-(1,6)-fucosyltransferase OS=Homo sapiens GN=FUT8 PE=1 SV=2 - [FUT8_HUMAN]	11,13	6	0,587	0,632	[illegible]
Q8IZ41	Ras and EF-hand domain-containing protein OS=Homo sapiens GN=RASEF PE=1 SV=1 - [RASEF_HUMAN]	11,08	6	1,322	1,329	[illegible]
Q9H078	Caseinolytic peptidase B protein homolog OS=Homo sapiens GN=CLPB PE=1 SV=1 - [CLPB_HUMAN]	11,03	6		0,975	[illegible]
P17813	Endoglin OS=Homo sapiens GN=ENG PE=1 SV=2 - [EGLN_HUMAN]	10,94	6	1,685	1,523	[illegible]
P33121	Long-chain-fatty-acid--CoA ligase 1 OS=Homo sapiens GN=ACSL1 PE=1 SV=1 - [ACSL1_HUMAN]	10,89	6	1,687	1,374	[illegible]
P07384	Calpain-1 catalytic subunit OS=Homo sapiens GN=CAPN1 PE=1 SV=1 - [CAN1_HUMAN]	10,78	6	0,676	0,831	[illegible]
Q8IW92	Beta-galactosidase-1-like protein 2 OS=Homo sapiens GN=GLB1L2 PE=2 SV=1 - [GLBL2_HUMAN]	10,69	6	0,869	1,166	[illegible]
P30419	Glycylpeptide N-tetradecanoyltransferase 1 OS=Homo sapiens GN=NMT1 PE=1 SV=2 - [NMT1_HUMAN]	10,69	6	0,671		[illegible]
Q8N271	Prominin-2 OS=Homo sapiens GN=PROM2 PE=1 SV=1 - [PROM2_HUMAN]	10,31	6	0,467	0,710	[illegible]
P29728	2'-5'-oligoadenylate synthase 2 OS=Homo sapiens GN=OAS2 PE=1 SV=3 - [OAS2_HUMAN]	10,29	6	0,262	0,438	[illegible]
Q96AA3	Protein RFT1 homolog OS=Homo sapiens GN=RFT1 PE=1 SV=1 - [RFT1_HUMAN]	10,17	6	1,179	1,159	[illegible]
Q96CM8	Acyl-CoA synthetase family member 2, mitochondrial OS=Homo sapiens GN=ACSF2 PE=1 SV=2 - [ACSF2_HUMAN]	9,92	6	0,761	0,756	[illegible]
P21980	Protein-glutamine gamma-glutamyltransferase 2 OS=Homo sapiens GN=TGM2 PE=1 SV=2 - [TGM2_HUMAN]	9,90	6	0,499	0,716	[illegible]
Q9NY93	Probable ATP-dependent RNA helicase DDX56 OS=Homo sapiens GN=DDX56 PE=1 SV=1 - [DDX56_HUMAN]	9,87	6	0,812	0,887	[illegible]
Q2NL82	Pre-rRNA-processing protein TSR1 homolog OS=Homo sapiens GN=TSR1 PE=1 SV=1 - [TSR1_HUMAN]	9,70	6	0,868		[illegible]
P25205	DNA replication licensing factor MCM3 OS=Homo sapiens GN=MCM3 PE=1 SV=3 - [MCM3_HUMAN]	9,53	6		0,788	[illegible]
Q7L8L6	FAST kinase domain-containing protein 5 OS=Homo sapiens GN=FASTKD5 PE=1 SV=1 - [FAKD5_HUMAN]	9,42	6	0,862	0,688	[illegible]
O94854-2	Isoform 2 of Uncharacterized protein KIAA0754 OS=Homo sapiens GN=KIAA0754 - [K0754_HUMAN]	9,39	6		1,593	[illegible]
Q86W92-4	Isoform 4 of Liprin-beta-1 OS=Homo sapiens GN=PPFIBP1 - [LIPB1_HUMAN]	9,39	6	1,278	1,006	[illegible]
Q8IUC4	Rhophilin-2 OS=Homo sapiens GN=RHPN2 PE=1 SV=1 - [RHPN2_HUMAN]	9,33	6	0,851	0,977	[illegible]
Q5JRX3	Presequence protease, mitochondrial OS=Homo sapiens GN=PITRM1 PE=1 SV=2 - [PREP_HUMAN]	9,26	6	0,977	1,151	[illegible]
Q6NUM6	tRNA wybutosine-synthesizing protein 1 homolog B OS=Homo sapiens GN=TYW1B PE=2 SV=2 - [TYW1B_HUMAN]	9,13	6	1,198	0,992	[illegible]
Q7Z2K8	G protein-regulated inducer of neurite outgrowth 1 OS=Homo sapiens GN=GPRIN1 PE=1 SV=2 - [GRIN1_HUMAN]	9,03	6	1,740	1,233	[illegible]
Q9H8H2	Probable ATP-dependent RNA helicase DDX31 OS=Homo sapiens GN=DDX31 PE=1 SV=2 - [DDX31_HUMAN]	8,93	6	1,287		[illegible]
P04626	Receptor tyrosine-protein kinase erbB-2 OS=Homo sapiens GN=ERBB2 PE=1 SV=1 - [ERBB2_HUMAN]	8,84	6	0,930	0,765	[illegible]
Q08945	FACT complex subunit SSRP1 OS=Homo sapiens GN=SSRP1 PE=1 SV=1 - [SSRP1_HUMAN]	8,74	6	1,111	0,808	[illegible]
Q15334	Lethal(2) giant larvae protein homolog 1 OS=Homo sapiens GN=LLGL1 PE=1 SV=3 - [L2GL1_HUMAN]	8,74	6	1,291	1,106	[illegible]
P51790	H(+)/Cl(-) exchange transporter 3 OS=Homo sapiens GN=CLCN3 PE=1 SV=2 - [CLCN3_HUMAN]	8,68	6	1,327	1,137	[illegible]
Q9Y6R1	Electrogenic sodium bicarbonate cotransporter 1 OS=Homo sapiens GN=SLC4A4 PE=1 SV=1 - [S4A4_HUMAN]	8,16	6	1,961	1,702	[illegible]
Q12866	Tyrosine-protein kinase Mer OS=Homo sapiens GN=MERTK PE=1 SV=2 - [MERTK_HUMAN]	8,11	6	0,473	0,572	[illegible]
I3L416	Coronin OS=Homo sapiens GN=CORO7 PE=3 SV=1 - [I3L416_HUMAN]	7,92	6	1,412	1,231	[illegible]
Q5JTZ9	Alanine--tRNA ligase, mitochondrial OS=Homo sapiens GN=AARS2 PE=1 SV=1 - [SYAM_HUMAN]	7,82	6	0,810	0,963	[illegible]
Q8IZL8	Proline-, glutamic acid- and leucine-rich protein 1 OS=Homo sapiens GN=PELP1 PE=1 SV=2 - [PELP1_HUMAN]	7,79	6		0,714	[illegible]
O00203	AP-3 complex subunit beta-1 OS=Homo sapiens GN=AP3B1 PE=1 SV=3 - [AP3B1_HUMAN]	7,77	6	0,721	1,150	[illegible]
Q8NI36	WD repeat-containing protein 36 OS=Homo sapiens GN=WDR36 PE=1 SV=1 - [WDR36_HUMAN]	7,68	6	1,012	0,928	[illegible]
Q02487	Desmocollin-2 OS=Homo sapiens GN=DSC2 PE=1 SV=1 - [DSC2_HUMAN]	7,55	6	0,679	0,654	[illegible]
Q99569	Plakophilin-4 OS=Homo sapiens GN=PKP4 PE=1 SV=2 - [PKP4_HUMAN]	7,38	6	0,813	0,829	[illegible]
Q8IY37	Probable ATP-dependent RNA helicase DHX37 OS=Homo sapiens GN=DHX37 PE=1 SV=1 - [DHX37_HUMAN]	7,35	6	0,768	0,331	[illegible]
Q5T8P6	RNA-binding protein 26 OS=Homo sapiens GN=RBM26 PE=1 SV=3 - [RBM26_HUMAN]	7,35	6	0,646		[illegible]
Q7Z3K3	Pogo transposable element with ZNF domain OS=Homo sapiens GN=POGZ PE=1 SV=2 - [POGZ_HUMAN]	7,30	6			[illegible]
Q9BWU0	Kanadaptin OS=Homo sapiens GN=SLC4A1AP PE=1 SV=1 - [NADAP_HUMAN]	7,29	6			[illegible]
O95819-5	Isoform 5 of Mitogen-activated protein kinase kinase kinase kinase 4 OS=Homo sapiens GN=MAP4K4 - [M4K4_HUMAN]	6,84	6			[illegible]
Q15021	Condensin complex subunit 1 OS=Homo sapiens GN=NCAPD2 PE=1 SV=3 - [CND1_HUMAN]	6,71	6	0,763	0,615	[illegible]
Q04912	Macrophage-stimulating protein receptor OS=Homo sapiens GN=MST1R PE=1 SV=2 - [RON_HUMAN]	6,71	6	0,374	1,020	[illegible]
O15357	Phosphatidylinositol 3,4,5-trisphosphate 5-phosphatase 2 OS=Homo sapiens GN=INPPL1 PE=1 SV=2 - [SHIP2_HUMAN]	6,60	6	0,814		[illegible]
A0AVT1	Ubiquitin-like modifier-activating enzyme 6 OS=Homo sapiens GN=UBA6 PE=1 SV=1 - [UBA6_HUMAN]	6,37	6	0,664	0,709	[illegible]
Q9Y6K5	2'-5'-oligoadenylate synthase 3 OS=Homo sapiens GN=OAS3 PE=1 SV=3 - [OAS3_HUMAN]	6,07	6	0,151	0,618	[illegible]
Q9UBS9	Protein osteopotentia homolog OS=Homo sapiens GN=C1orf9 PE=2 SV=1 - [OSPT_HUMAN]	5,90	6	0,884	0,846	[illegible]
O15294	UDP-N-acetylglucosamine--peptide N-acetylglucosaminyltransferase 110 kDa subunit OS=Homo sapiens GN=OGT PE=1 SV=3 - [OGT1_H	5,26	6	0,939	1,039	[illegible]
Q92545	Transmembrane protein 131 OS=Homo sapiens GN=TMEM131 PE=1 SV=3 - [TM131_HUMAN]	5,20	6	0,826	1,180	[illegible]
Q460N5	Poly [ADP-ribose] polymerase 14 OS=Homo sapiens GN=PARP14 PE=1 SV=3 - [PAR14_HUMAN]	5,16	6	0,672	0,639	[illegible]
Q9UM54	Unconventional myosin-VI OS=Homo sapiens GN=MYO6 PE=1 SV=4 - [MYO6_HUMAN]	5,10	6	0,439	0,274	[illegible]
O95602	DNA-directed RNA polymerase I subunit RPA1 OS=Homo sapiens GN=POLR1A PE=1 SV=2 - [RPA1_HUMAN]	5,00	6			[illegible]
Q8TD43	Transient receptor potential cation channel subfamily M member 4 OS=Homo sapiens GN=TRPM4 PE=1 SV=1 - [TRPM4_HUMAN]	4,94	6	1,006	0,911	[illegible]
Q9Y4K1	Absent in melanoma 1 protein OS=Homo sapiens GN=AIM1 PE=1 SV=3 - [AIM1_HUMAN]	4,88	6	0,583		[illegible]
P23470	Receptor-type tyrosine-protein phosphatase gamma OS=Homo sapiens GN=PTPRG PE=1 SV=4 - [PTPRG_HUMAN]	4,71	6	1,585	1,604	[illegible]
O14776	Transcription elongation regulator 1 OS=Homo sapiens GN=TCERG1 PE=1 SV=2 - [TCRG1_HUMAN]	4,55	6	1,287	1,309	[illegible]
Q5SW79	Centrosomal protein of 170 kDa OS=Homo sapiens GN=CEP170 PE=1 SV=1 - [CE170_HUMAN]	4,55	6	1,303		[illegible]
Q12888	Tumor suppressor p53-binding protein 1 OS=Homo sapiens GN=TP53BP1 PE=1 SV=2 - [TP53B_HUMAN]	4,36	6			[illegible]
Q9BZ29-5	Isoform 2 of Dedicator of cytokinesis protein 9 OS=Homo sapiens GN=DOCK9 - [DOCK9_HUMAN]	4,26	6	2,164		[illegible]
Q14966	Zinc finger protein 638 OS=Homo sapiens GN=ZNF638 PE=1 SV=2 - [ZN638_HUMAN]	4,20	6			[illegible]
A3KMH1	Uncharacterized protein KIAA0564 OS=Homo sapiens GN=KIAA0564 PE=1 SV=2 - [K0564_HUMAN]	3,52	6	0,792	1,232	[illegible]
Q96L91	E1A-binding protein p400 OS=Homo sapiens GN=EP400 PE=1 SV=4 - [EP400_HUMAN]	3,39	6			[illegible]
Q96N67	Dedicator of cytokinesis protein 7 OS=Homo sapiens GN=DOCK7 PE=1 SV=4 - [DOCK7_HUMAN]	3,36	6	0,984	1,009	[illegible]
Q5UIP0	Telomere-associated protein RIF1 OS=Homo sapiens GN=RIF1 PE=1 SV=2 - [RIF1_HUMAN]	3,07	6	0,828		[illegible]
O95071	E3 ubiquitin-protein ligase UBR5 OS=Homo sapiens GN=UBR5 PE=1 SV=2 - [UBR5_HUMAN]	2,97	6	0,801		[illegible]
Q709C8	Vacuolar protein sorting-associated protein 13C OS=Homo sapiens GN=VPS13C PE=1 SV=1 - [VP13C_HUMAN]	2,77	6	1,061	0,825	[illegible]
Q03164	Histone-lysine N-methyltransferase MLL OS=Homo sapiens GN=MLL PE=1 SV=5 - [MLL1_HUMAN]	1,89	6			[illegible]
P04114	Apolipoprotein B-100 OS=Homo sapiens GN=APOB PE=1 SV=2 - [APOB_HUMAN]	1,45	6	0,978	1,063	[illegible]
Q99417	C-Myc-binding protein OS=Homo sapiens GN=MYCBP PE=1 SV=3 - [MYCBP_HUMAN]	65,05	5	0,548	0,875	[illegible]
Q9HCY8	Protein S100-A14 OS=Homo sapiens GN=S100A14 PE=1 SV=1 - [S10AE_HUMAN]	63,46	5	0,536	0,415	[illegible]
P84085	ADP-ribosylation factor 5 OS=Homo sapiens GN=ARF5 PE=1 SV=2 - [ARF5_HUMAN]	61,11	5	1,034		[illegible]
Q16864	V-type proton ATPase subunit F OS=Homo sapiens GN=ATP6V1F PE=1 SV=2 - [VATF_HUMAN]	60,50	5	1,072	0,952	[illegible]
Q15369	Transcription elongation factor B polypeptide 1 OS=Homo sapiens GN=TCEB1 PE=1 SV=1 - [ELOC_HUMAN]	58,04	5	0,917	1,053	[illegible]
P05141	ADP/ATP translocase 2 OS=Homo sapiens GN=SLC25A5 PE=1 SV=7 - [ADT2_HUMAN]	55,70	5	0,549	0,496	[illegible]
P13746	HLA class I histocompatibility antigen, A-11 alpha chain OS=Homo sapiens GN=HLA-A PE=1 SV=1 - [1A11_HUMAN]	55,62	5	1,121	1,118	[illegible]
O00483	NADH dehydrogenase [ubiquinone] 1 alpha subcomplex subunit 4 OS=Homo sapiens GN=NDUFA4 PE=1 SV=1 - [NDUA4_HUMAN]	55,56	5	1,851	1,649	[illegible]
Q9Y5L4	Mitochondrial import inner membrane translocase subunit Tim13 OS=Homo sapiens GN=TIMM13 PE=1 SV=1 - [TIM13_HUMAN]	53,68	5	0,657	0,715	[illegible]
P82909	28S ribosomal protein S36, mitochondrial OS=Homo sapiens GN=MRPS36 PE=1 SV=2 - [RT36_HUMAN]	52,43	5	1,116	1,168	[illegible]
Q9NS69	Mitochondrial import receptor subunit TOM22 homolog OS=Homo sapiens GN=TOMM22 PE=1 SV=3 - [TOM22_HUMAN]	51,41	5	1,230	1,159	[illegible]
Q9Y281	Cofilin-2 OS=Homo sapiens GN=CFL2 PE=1 SV=1 - [COF2_HUMAN]	51,20	5			[illegible]
P18859	ATP synthase-coupling factor 6, mitochondrial OS=Homo sapiens GN=ATP5J PE=1 SV=1 - [ATP5J_HUMAN]	50,93	5	1,212	0,896	[illegible]
P63220	40S ribosomal protein S21 OS=Homo sapiens GN=RPS21 PE=1 SV=1 - [RS21_HUMAN]	49,40	5	0,777	0,891	[illegible]
P61006	Ras-related protein Rab-8A OS=Homo sapiens GN=RAB8A PE=1 SV=1 - [RAB8A_HUMAN]	47,34	5	1,575	1,646	[illegible]
A6NIZ1	Ras-related protein Rap-1b-like protein OS=Homo sapiens PE=2 SV=1 - [RP1BL_HUMAN]	46,74	5	0,977	0,915	[illegible]
Q13636	Ras-related protein Rab-31 OS=Homo sapiens GN=RAB31 PE=1 SV=1 - [RAB31_HUMAN]	46,39	5	1,045	0,955	[illegible]
O43852	Calumenin OS=Homo sapiens GN=CALU PE=1 SV=2 - [CALU_HUMAN]	44,76	5	1,554	1,931	[illegible]
Q9NZ45	CDGSH iron-sulfur domain-containing protein 1 OS=Homo sapiens GN=CISD1 PE=1 SV=1 - [CISD1_HUMAN]	44,44	5	1,424	1,447	[illegible]
P61769	Beta-2-microglobulin OS=Homo sapiens GN=B2M PE=1 SV=1 - [B2MG_HUMAN]	43,70	5	0,897	0,795	[illegible]
P61916	Epididymal secretory protein E1 OS=Homo sapiens GN=NPC2 PE=1 SV=1 - [NPC2_HUMAN]	43,05	5	1,113	0,999	[illegible]
O43491	Band 4.1-like protein 2 OS=Homo sapiens GN=EPB41L2 PE=1 SV=1 - [E41L2_HUMAN]	41,89	5	2,222	1,464	[illegible]
P08754	Guanine nucleotide-binding protein G(k) subunit alpha OS=Homo sapiens GN=GNAI3 PE=1 SV=3 - [GNAI3_HUMAN]	41,24	5	1,541	1,330	[illegible]
Q9BV40	Vesicle-associated membrane protein 8 OS=Homo sapiens GN=VAMP8 PE=1 SV=1 - [VAMP8_HUMAN]	41,00	5	0,682	0,689	[illegible]
P51636	Caveolin-2 OS=Homo sapiens GN=CAV2 PE=1 SV=2 - [CAV2_HUMAN]	40,74	5	1,828	1,920	[illegible]
P04899	Guanine nucleotide-binding protein G(i) subunit alpha-2 OS=Homo sapiens GN=GNAI2 PE=1 SV=3 - [GNAI2_HUMAN]	40,56	5	1,289	1,322	[illegible]
P84243	Histone H3.3 OS=Homo sapiens GN=H3F3A PE=1 SV=2 - [H33_HUMAN]	40,44	5	1,158	1,124	[illegible]
Q8N4Q1	Mitochondrial intermembrane space import and assembly protein 40 OS=Homo sapiens GN=CHCHD4 PE=1 SV=1 - [MIA40_HUMAN]	40,14	5		0,622	[illegible]
P62072	Mitochondrial import inner membrane translocase subunit Tim10 OS=Homo sapiens GN=TIMM10 PE=1 SV=1 - [TIM10_HUMAN]	40,00	5	1,070	1,117	[illegible]
P60981	Destrin OS=Homo sapiens GN=DSTN PE=1 SV=3 - [DEST_HUMAN]	40,00	5	0,614	0,712	[illegible]
P05114	Non-histone chromosomal protein HMG-14 OS=Homo sapiens GN=HMGN1 PE=1 SV=3 - [HMGN1_HUMAN]	39,00	5			[illegible]
Q9UL26	Ras-related protein Rab-22A OS=Homo sapiens GN=RAB22A PE=1 SV=2 - [RB22A_HUMAN]	38,66	5	1,132	1,456	[illegible]
P22061	Protein-L-isoaspartate(D-aspartate) O-methyltransferase OS=Homo sapiens GN=PCMT1 PE=1 SV=4 - [PIMT_HUMAN]	38,33	5	0,903	1,017	[illegible]
P35080	Profilin-2 OS=Homo sapiens GN=PFN2 PE=1 SV=3 - [PROF2_HUMAN]	37,86	5		0,920	[illegible]
O60499	Syntaxin-10 OS=Homo sapiens GN=STX10 PE=1 SV=1 - [STX10_HUMAN]	37,35	5	0,985	1,000	[illegible]
P21291	Cysteine and glycine-rich protein 1 OS=Homo sapiens GN=CSRP1 PE=1 SV=3 - [CSRP1_HUMAN]	36,79	5	**2,016**		[illegible]
Q13405	39S ribosomal protein L49, mitochondrial OS=Homo sapiens GN=MRPL49 PE=1 SV=1 - [RM49_HUMAN]	36,75	5	1,025	0,778	[illegible]
P20674	Cytochrome c oxidase subunit 5A, mitochondrial OS=Homo sapiens GN=COX5A PE=1 SV=2 - [COX5A_HUMAN]	36,00	5	1,462	1,451	[illegible]
P51153	Ras-related protein Rab-13 OS=Homo sapiens GN=RAB13 PE=1 SV=1 - [RAB13_HUMAN]	35,96	5	1,391	1,112	[illegible]
P62841	40S ribosomal protein S15 OS=Homo sapiens GN=RPS15 PE=1 SV=2 - [RS15_HUMAN]	35,86	5	1,009	0,908	[illegible]
O95182	NADH dehydrogenase [ubiquinone] 1 alpha subcomplex subunit 7 OS=Homo sapiens GN=NDUFA7 PE=1 SV=3 - [NDUA7_HUMAN]	35,40	5	1,121	1,082	[illegible]
O60493	Sorting nexin-3 OS=Homo sapiens GN=SNX3 PE=1 SV=3 - [SNX3_HUMAN]	35,19	5	1,317	1,255	[illegible]
P06454	Prothymosin alpha OS=Homo sapiens GN=PTMA PE=1 SV=2 - [PTMA_HUMAN]	35,14	5	0,964	1,012	[illegible]
P25398	40S ribosomal protein S12 OS=Homo sapiens GN=RPS12 PE=1 SV=3 - [RS12_HUMAN]	34,85	5	0,764	0,758	[illegible]
P18621	60S ribosomal protein L17 OS=Homo sapiens GN=RPL17 PE=1 SV=3 - [RL17_HUMAN]	34,78	5	0,904	0,861	[illegible]
G3V203	60S ribosomal protein L18 OS=Homo sapiens GN=RPL18 PE=3 SV=1 - [G3V203_HUMAN]	34,76	5			[illegible]
P62873	Guanine nucleotide-binding protein G(I)/G(S)/G(T) subunit beta-1 OS=Homo sapiens GN=GNB1 PE=1 SV=3 - [GBB1_HUMAN]	34,71	5	1,478	1,747	[illegible]
P69905	Hemoglobin subunit alpha OS=Homo sapiens GN=HBA1 PE=1 SV=2 - [HBA_HUMAN]	34,51	5	1,897	1,873	[illegible]
Q8WUD4	Coiled-coil domain-containing protein 12 OS=Homo sapiens GN=CCDC12 PE=1 SV=1 - [CCD12_HUMAN]	34,34	5	0,883		[illegible]
O96000	NADH dehydrogenase [ubiquinone] 1 beta subcomplex subunit 10 OS=Homo sapiens GN=NDUFB10 PE=1 SV=3 - [NDUBA_HUMAN]	34,30	5	1,256	1,262	[illegible]
P67812	Signal peptidase complex catalytic subunit SEC11A OS=Homo sapiens GN=SEC11A PE=1 SV=1 - [SC11A_HUMAN]	33,52	5	1,065	0,900	[illegible]
P13693	Translationally-controlled tumor protein OS=Homo sapiens GN=TPT1 PE=1 SV=1 - [TCTP_HUMAN]	33,14	5	0,553	0,783	[illegible]
Q8TAE8	Growth arrest and DNA damage-inducible proteins-interacting protein 1 OS=Homo sapiens GN=GADD45GIP1 PE=1 SV=1 - [G45IP_HUMA	32,88	5	0,809	0,843	[illegible]
P30044	Peroxiredoxin-5, mitochondrial OS=Homo sapiens GN=PRDX5 PE=1 SV=4 - [PRDX5_HUMAN]	32,71	5	0,646	0,669	[illegible]
O14653	Golgi SNAP receptor complex member 2 OS=Homo sapiens GN=GOSR2 PE=1 SV=2 - [GOSR2_HUMAN]	32,55	5	0,977	1,007	[illegible]
P55795	Heterogeneous nuclear ribonucleoprotein H2 OS=Homo sapiens GN=HNRNPH2 PE=1 SV=1 - [HNRH2_HUMAN]	32,52	5	0,615	0,567	[illegible]
P18077	60S ribosomal protein L35a OS=Homo sapiens GN=RPL35A PE=1 SV=2 - [RL35A_HUMAN]	31,82	5	1,191	1,080	[illegible]
Q00839	Heterogeneous nuclear ribonucleoprotein U OS=Homo sapiens GN=HNRNPU PE=1 SV=6 - [HNRPU_HUMAN]	31,52	5	1,975	1,967	[illegible]
P62847	40S ribosomal protein S24 OS=Homo sapiens GN=RPS24 PE=1 SV=1 - [RS24_HUMAN]	30,83	5	0,876	0,750	[illegible]
P49207	60S ribosomal protein L34 OS=Homo sapiens GN=RPL34 PE=1 SV=3 - [RL34_HUMAN]	30,77	5	1,323	1,343	[illegible]
Q9BPX5	Actin-related protein 2/3 complex subunit 5-like protein OS=Homo sapiens GN=ARPC5L PE=1 SV=1 - [ARP5L_HUMAN]	30,72	5	0,635	0,642	[illegible]
P0C7P0	CDGSH iron-sulfur domain-containing protein 3, mitochondrial OS=Homo sapiens GN=CISD3 PE=1 SV=1 - [CISD3_HUMAN]	30,71	5	**2,275**	1,585	[illegible]
Q13162	Peroxiredoxin-4 OS=Homo sapiens GN=PRDX4 PE=1 SV=1 - [PRDX4_HUMAN]	30,26	5	1,538	1,400	[illegible]
P30042	ES1 protein homolog, mitochondrial OS=Homo sapiens GN=C21orf33 PE=1 SV=3 - [ES1_HUMAN]	30,22	5	0,471	0,853	[illegible]
O95336	6-phosphogluconolactonase OS=Homo sapiens GN=PGLS PE=1 SV=2 - [6PGL_HUMAN]	29,84	5		0,831	[illegible]
O00264	Membrane-associated progesterone receptor component 1 OS=Homo sapiens GN=PGRMC1 PE=1 SV=3 - [PGRC1_HUMAN]	29,74	5	0,638	0,591	[illegible]
P42766	60S ribosomal protein L35 OS=Homo sapiens GN=RPL35 PE=1 SV=2 - [RL35_HUMAN]	29,27	5	1,059	0,778	[illegible]
E7EVD0	Casein kinase I isoform gamma-3 OS=Homo sapiens GN=CSNK1G3 PE=4 SV=1 - [E7EVD0_HUMAN]	28,62	5	1,066		[illegible]
Q13084	39S ribosomal protein L28, mitochondrial OS=Homo sapiens GN=MRPL28 PE=1 SV=4 - [RM28_HUMAN]	28,52	5	0,891	0,633	[illegible]
P46776	60S ribosomal protein L27a OS=Homo sapiens GN=RPL27A PE=1 SV=2 - [RL27A_HUMAN]	28,38	5	0,969	0,992	[illegible]
P29373	Cellular retinoic acid-binding protein 2 OS=Homo sapiens GN=CRABP2 PE=1 SV=2 - [RABP2_HUMAN]	28,26	5	0,313	0,319	[illegible]
Q15185	Prostaglandin E synthase 3 OS=Homo sapiens GN=PTGES3 PE=1 SV=1 - [TEBP_HUMAN]	28,13	5	0,761	0,665	[illegible]
P19388	DNA-directed RNA polymerases I, II, and III subunit RPABC1 OS=Homo sapiens GN=POLR2E PE=1 SV=4 - [RPAB1_HUMAN]	28,10	5	0,853	1,037	[illegible]
Q15363	Transmembrane emp24 domain-containing protein 2 OS=Homo sapiens GN=TMED2 PE=1 SV=1 - [TMED2_HUMAN]	27,86	5	0,724	0,748	[illegible]
O43447	Peptidyl-prolyl cis-trans isomerase H OS=Homo sapiens GN=PPIH PE=1 SV=1 - [PPIH_HUMAN]	27,68	5	0,925	0,840	[illegible]
O00233	26S proteasome non-ATPase regulatory subunit 9 OS=Homo sapiens GN=PSMD9 PE=1 SV=3 - [PSMD9_HUMAN]	27,35	5	0,981		[illegible]
O95372	Acyl-protein thioesterase 2 OS=Homo sapiens GN=LYPLA2 PE=1 SV=1 - [LYPA2_HUMAN]	27,27	5	1,016	0,928	[illegible]
Q9HBH1	Peptide deformylase, mitochondrial OS=Homo sapiens GN=PDF PE=1 SV=1 - [DEFM_HUMAN]	27,16	5	1,443	1,678	[illegible]
Q9NQX7	Integral membrane protein 2C OS=Homo sapiens GN=ITM2C PE=1 SV=1 - [ITM2C_HUMAN]	26,97	5	0,947	0,812	[illegible]
P48047	ATP synthase subunit O, mitochondrial OS=Homo sapiens GN=ATP5O PE=1 SV=1 - [ATPO_HUMAN]	26,76	5	0,499	0,646	[illegible]
Q9UHA4	Ragulator complex protein LAMTOR3 OS=Homo sapiens GN=LAMTOR3 PE=1 SV=1 - [LTOR3_HUMAN]	26,61	5	1,183	1,348	[illegible]
Q16740	Putative ATP-dependent Clp protease proteolytic subunit, mitochondrial OS=Homo sapiens GN=CLPP PE=1 SV=1 - [CLPP_HUMAN]	26,35	5	0,840	0,805	[illegible]
C9JXG8	Ran-specific GTPase-activating protein (Fragment) OS=Homo sapiens GN=RANBP1 PE=4 SV=2 - [C9JXG8_HUMAN]	26,21	5			[illegible]
Q9HBH0	Rho-related GTP-binding protein RhoF OS=Homo sapiens GN=RHOF PE=2 SV=1 - [RHOF_HUMAN]	25,12	5	1,130	1,288	[illegible]
P52597	Heterogeneous nuclear ribonucleoprotein F OS=Homo sapiens GN=HNRNPF PE=1 SV=3 - [HNRPF_HUMAN]	25,06	5	0,682	0,731	[illegible]
Q9BPW8	Protein NipSnap homolog 1 OS=Homo sapiens GN=NIPSNAP1 PE=1 SV=1 - [NIPS1_HUMAN]	25,00	5	0,776	0,980	[illegible]
Q9HCN8	Stromal cell-derived factor 2-like protein 1 OS=Homo sapiens GN=SDF2L1 PE=1 SV=2 - [SDF2L_HUMAN]	24,89	5	0,904	1,128	[illegible]
Q9Y676	28S ribosomal protein S18b, mitochondrial OS=Homo sapiens GN=MRPS18B PE=1 SV=1 - [RT18B_HUMAN]	24,81	5	1,029	1,204	[illegible]
Q13838	Spliceosome RNA helicase DDX39B OS=Homo sapiens GN=DDX39B PE=1 SV=1 - [DX39B_HUMAN]	24,53	5	0,662	1,109	[illegible]
Q9Y287	Integral membrane protein 2B OS=Homo sapiens GN=ITM2B PE=1 SV=1 - [ITM2B_HUMAN]	24,44	5	0,979	0,971	[illegible]
P04632	Calpain small subunit 1 OS=Homo sapiens GN=CAPNS1 PE=1 SV=1 - [CPNS1_HUMAN]	24,25	5	1,090	0,988	[illegible]
Q9ULC3	Ras-related protein Rab-23 OS=Homo sapiens GN=RAB23 PE=1 SV=1 - [RAB23_HUMAN]	24,05	5	1,285	1,197	[illegible]
Q8NCW5	NAD(P)H-hydrate epimerase OS=Homo sapiens GN=APOA1BP PE=1 SV=2 - [NNRE_HUMAN]	23,96	5	0,665	0,535	[illegible]

O75915	PRA1 family protein 3 OS=Homo sapiens GN=ARL6IP5 PE=1 SV=1 - [PRAF3_HUMAN]	23,94	5	1,113
Q71RG4	Transmembrane and ubiquitin-like domain-containing protein 2 OS=Homo sapiens GN=TMUB2 PE=2 SV=2 - [TMUB2_HUMAN]	23,68	5	1,640
P09012	U1 small nuclear ribonucleoprotein A OS=Homo sapiens GN=SNRPA PE=1 SV=3 - [SNRPA_HUMAN]	23,40	5	
Q12981	Vesicle transport protein SEC20 OS=Homo sapiens GN=BNIP1 PE=1 SV=3 - [SEC20_HUMAN]	23,25	5	1,148
Q9UBE0	SUMO-activating enzyme subunit 1 OS=Homo sapiens GN=SAE1 PE=1 SV=1 - [SAE1_HUMAN]	23,12	5	1,115
Q9NPA0	UPF0480 protein C15orf24 OS=Homo sapiens GN=C15orf24 PE=1 SV=1 - [CO024_HUMAN]	22,73	5	0,951
P52815	39S ribosomal protein L12, mitochondrial OS=Homo sapiens GN=MRPL12 PE=1 SV=2 - [RM12_HUMAN]	22,73	5	0,829
P57088	Transmembrane protein 33 OS=Homo sapiens GN=TMEM33 PE=1 SV=2 - [TMM33_HUMAN]	22,27	5	1,003
O14908	PDZ domain-containing protein GIPC1 OS=Homo sapiens GN=GIPC1 PE=1 SV=2 - [GIPC1_HUMAN]	22,22	5	1,279
Q9GZT9	Egl nine homolog 1 OS=Homo sapiens GN=EGLN1 PE=1 SV=1 - [EGLN1_HUMAN]	22,07	5	1,140
P61289	Proteasome activator complex subunit 3 OS=Homo sapiens GN=PSME3 PE=1 SV=1 - [PSME3_HUMAN]	22,05	5	0,843
Q13151	Heterogeneous nuclear ribonucleoprotein A0 OS=Homo sapiens GN=HNRNPA0 PE=1 SV=1 - [ROA0_HUMAN]	21,97	5	1,147
P09601	Heme oxygenase 1 OS=Homo sapiens GN=HMOX1 PE=1 SV=1 - [HMOX1_HUMAN]	21,88	5	**2,162**
Q86V81	THO complex subunit 4 OS=Homo sapiens GN=ALYREF PE=1 SV=3 - [THOC4_HUMAN]	21,79	5	0,964
Q9Y624	Junctional adhesion molecule A OS=Homo sapiens GN=F11R PE=1 SV=1 - [JAM1_HUMAN]	21,74	5	0,491
Q9UNQ2	Probable dimethyladenosine transferase OS=Homo sapiens GN=DIMT1 PE=1 SV=1 - [DIM1_HUMAN]	21,73	5	0,999
Q9BYD2	39S ribosomal protein L9, mitochondrial OS=Homo sapiens GN=MRPL9 PE=1 SV=2 - [RM09_HUMAN]	21,72	5	0,693
O75911	Short-chain dehydrogenase/reductase 3 OS=Homo sapiens GN=DHRS3 PE=1 SV=2 - [DHRS3_HUMAN]	21,52	5	0,519
Q9BVV7	Mitochondrial import inner membrane translocase subunit Tim21 OS=Homo sapiens GN=TIMM21 PE=1 SV=1 - [TIM21_HUMAN]	21,37	5	1,512
Q08257	Quinone oxidoreductase OS=Homo sapiens GN=CRYZ PE=1 SV=1 - [QOR_HUMAN]	21,28	5	1,077
Q9BYG3	MKI67 FHA domain-interacting nucleolar phosphoprotein OS=Homo sapiens GN=MKI67IP PE=1 SV=1 - [MK67I_HUMAN]	21,16	5	
O15162	Phospholipid scramblase 1 OS=Homo sapiens GN=PLSCR1 PE=1 SV=1 - [PLS1_HUMAN]	21,07	5	0,587
P46937	Yorkie homolog OS=Homo sapiens GN=YAP1 PE=1 SV=2 - [YAP1_HUMAN]	20,83	5	
O43809	Cleavage and polyadenylation specificity factor subunit 5 OS=Homo sapiens GN=NUDT21 PE=1 SV=1 - [CPSF5_HUMAN]	20,70	5	0,705
O75822	Eukaryotic translation initiation factor 3 subunit J OS=Homo sapiens GN=EIF3J PE=1 SV=2 - [EIF3J_HUMAN]	20,54	5	0,874
Q9BTT0	Acidic leucine-rich nuclear phosphoprotein 32 family member E OS=Homo sapiens GN=ANP32E PE=1 SV=1 - [AN32E_HUMAN]	20,52	5	0,966
Q8NBX0	Saccharopine dehydrogenase-like oxidoreductase OS=Homo sapiens GN=SCCPDH PE=1 SV=1 - [SCPDL_HUMAN]	20,51	5	1,819
P15559	NAD(P)H dehydrogenase [quinone] 1 OS=Homo sapiens GN=NQO1 PE=1 SV=1 - [NQO1_HUMAN]	20,44	5	0,725
Q13641	Trophoblast glycoprotein OS=Homo sapiens GN=TPBG PE=1 SV=1 - [TPBG_HUMAN]	20,24	5	0,625
P31942	Heterogeneous nuclear ribonucleoprotein H3 OS=Homo sapiens GN=HNRNPH3 PE=1 SV=2 - [HNRH3_HUMAN]	20,23	5	1,116
P55036	26S proteasome non-ATPase regulatory subunit 4 OS=Homo sapiens GN=PSMD4 PE=1 SV=1 - [PSMD4_HUMAN]	20,16	5	0,709
Q00765	Receptor expression-enhancing protein 5 OS=Homo sapiens GN=REEP5 PE=1 SV=3 - [REEP5_HUMAN]	20,11	5	1,317
Q9Y2Z0	Suppressor of G2 allele of SKP1 homolog OS=Homo sapiens GN=SUGT1 PE=1 SV=3 - [SUGT1_HUMAN]	20,00	5	
P26885	Peptidyl-prolyl cis-trans isomerase FKBP2 OS=Homo sapiens GN=FKBP2 PE=1 SV=2 - [FKBP2_HUMAN]	19,72	5	1,040
Q9H061	Transmembrane protein 126A OS=Homo sapiens GN=TMEM126A PE=1 SV=1 - [T126A_HUMAN]	19,49	5	1,296
O43464	Serine protease HTRA2, mitochondrial OS=Homo sapiens GN=HTRA2 PE=1 SV=2 - [HTRA2_HUMAN]	19,43	5	
O43633	Charged multivesicular body protein 2a OS=Homo sapiens GN=CHMP2A PE=1 SV=1 - [CHM2A_HUMAN]	19,37	5	1,040
Q92820	Gamma-glutamyl hydrolase OS=Homo sapiens GN=GGH PE=1 SV=2 - [GGH_HUMAN]	19,18	5	0,900
Q96HE9	Proline-rich protein 11 OS=Homo sapiens GN=PRR11 PE=1 SV=1 - [PRR11_HUMAN]	19,17	5	0,853
Q00796	Sorbitol dehydrogenase OS=Homo sapiens GN=SORD PE=1 SV=4 - [DHSO_HUMAN]	19,05	5	0,897
Q92643	GPI-anchor transamidase OS=Homo sapiens GN=PIGK PE=1 SV=2 - [GPI8_HUMAN]	18,99	5	1,108
P07858	Cathepsin B OS=Homo sapiens GN=CTSB PE=1 SV=3 - [CATB_HUMAN]	18,88	5	0,910
Q9BUB7	Transmembrane protein 70, mitochondrial OS=Homo sapiens GN=TMEM70 PE=1 SV=2 - [TMM70_HUMAN]	18,85	5	1,214
P48739	Phosphatidylinositol transfer protein beta isoform OS=Homo sapiens GN=PITPNB PE=1 SV=2 - [PIPNB_HUMAN]	18,82	5	1,003
Q9H9J2	39S ribosomal protein L44, mitochondrial OS=Homo sapiens GN=MRPL44 PE=1 SV=1 - [RM44_HUMAN]	18,67	5	0,790
P10644	cAMP-dependent protein kinase type I-alpha regulatory subunit OS=Homo sapiens GN=PRKAR1A PE=1 SV=1 - [KAP0_HUMAN]	18,64	5	1,442
Q8NBZ7	UDP-glucuronic acid decarboxylase 1 OS=Homo sapiens GN=UXS1 PE=1 SV=1 - [UXS1_HUMAN]	18,57	5	1,347
O75683	Surfeit locus protein 6 OS=Homo sapiens GN=SURF6 PE=1 SV=3 - [SURF6_HUMAN]	18,56	5	1,291
O60762	Dolichol-phosphate mannosyltransferase OS=Homo sapiens GN=DPM1 PE=1 SV=1 - [DPM1_HUMAN]	18,46	5	0,917
Q13492	Phosphatidylinositol-binding clathrin assembly protein OS=Homo sapiens GN=PICALM PE=1 SV=2 - [PICAL_HUMAN]	18,40	5	0,785
Q9NUE0	Palmitoyltransferase ZDHHC18 OS=Homo sapiens GN=ZDHHC18 PE=2 SV=2 - [ZDH18_HUMAN]	18,30	5	1,076
Q6PCB7	Long-chain fatty acid transport protein 1 OS=Homo sapiens GN=SLC27A1 PE=2 SV=1 - [S27A1_HUMAN]	18,27	5	0,816
O43681	ATPase ASNA1 OS=Homo sapiens GN=ASNA1 PE=1 SV=2 - [ASNA_HUMAN]	18,10	5	1,394
Q92733	Proline-rich protein PRCC OS=Homo sapiens GN=PRCC PE=1 SV=1 - [PRCC_HUMAN]	17,92	5	
Q92783-2	Isoform 2 of Signal transducing adapter molecule 1 OS=Homo sapiens GN=STAM - [STAM1_HUMAN]	17,87	5	
Q96DI7	U5 small nuclear ribonucleoprotein 40 kDa protein OS=Homo sapiens GN=SNRNP40 PE=1 SV=1 - [SNR40_HUMAN]	17,65	5	0,738
Q9BXS4	Transmembrane protein 59 OS=Homo sapiens GN=TMEM59 PE=1 SV=1 - [TMM59_HUMAN]	17,65	5	1,199
Q96J42	Thioredoxin domain-containing protein 15 OS=Homo sapiens GN=TXNDC15 PE=1 SV=1 - [TXD15_HUMAN]	17,50	5	0,918
Q96CQ1	Solute carrier family 25 member 36 OS=Homo sapiens GN=SLC25A36 PE=2 SV=1 - [S2536_HUMAN]	17,36	5	1,334
A4D1E9	GTP-binding protein 10 OS=Homo sapiens GN=GTPBP10 PE=1 SV=1 - [GTPBA_HUMAN]	17,31	5	0,959
P11766	Alcohol dehydrogenase class-3 OS=Homo sapiens GN=ADH5 PE=1 SV=4 - [ADHX_HUMAN]	17,11	5	0,911
Q9BWH2	FUN14 domain-containing protein 2 OS=Homo sapiens GN=FUNDC2 PE=1 SV=2 - [FUND2_HUMAN]	16,93	5	0,852
Q96Q45	Transmembrane protein 237 OS=Homo sapiens GN=TMEM237 PE=1 SV=2 - [TM237_HUMAN]	16,91	5	1,606
Q9HB09	Bcl-2-like protein 12 OS=Homo sapiens GN=BCL2L12 PE=1 SV=1 - [B2L12_HUMAN]	16,77	5	1,403
Q96EU7	C1GALT1-specific chaperone 1 OS=Homo sapiens GN=C1GALT1C1 PE=1 SV=1 - [C1GLC_HUMAN]	16,67	5	0,954
Q6P9B6	TLD domain-containing protein KIAA1609 OS=Homo sapiens GN=KIAA1609 PE=1 SV=2 - [K1609_HUMAN]	16,67	5	0,641
Q13287	N-myc-interactor OS=Homo sapiens GN=NMI PE=1 SV=2 - [NMI_HUMAN]	16,61	5	0,608
Q9H7Z7	Prostaglandin E synthase 2 OS=Homo sapiens GN=PTGES2 PE=1 SV=1 - [PGES2_HUMAN]	16,45	5	1,093
Q9HC38	Glyoxalase domain-containing protein 4 OS=Homo sapiens GN=GLOD4 PE=1 SV=1 - [GLOD4_HUMAN]	16,29	5	0,738
Q8WVQ1	Soluble calcium-activated nucleotidase 1 OS=Homo sapiens GN=CANT1 PE=1 SV=1 - [CANT1_HUMAN]	16,21	5	0,937
Q9BRR6	ADP-dependent glucokinase OS=Homo sapiens GN=ADPGK PE=1 SV=1 - [ADPGK_HUMAN]	16,10	5	1,586
P13196	5-aminolevulinate synthase, nonspecific, mitochondrial OS=Homo sapiens GN=ALAS1 PE=1 SV=2 - [HEM1_HUMAN]	16,09	5	0,832
F5GZL4	Zinc finger protein 185 OS=Homo sapiens GN=ZNF185 PE=4 SV=1 - [F5GZL4_HUMAN]	16,04	5	
P45954	Short/branched chain specific acyl-CoA dehydrogenase, mitochondrial OS=Homo sapiens GN=ACADSB PE=1 SV=1 - [ACDSB_HUMAN]	15,97	5	0,741
Q13561	Dynactin subunit 2 OS=Homo sapiens GN=DCTN2 PE=1 SV=4 - [DCTN2_HUMAN]	15,96	5	0,879
Q86YQ8	Copine-8 OS=Homo sapiens GN=CPNE8 PE=1 SV=2 - [CPNE8_HUMAN]	15,96	5	1,142
Q00059	Transcription factor A, mitochondrial OS=Homo sapiens GN=TFAM PE=1 SV=1 - [TFAM_HUMAN]	15,85	5	0,625
Q9UHL4	Dipeptidyl peptidase 2 OS=Homo sapiens GN=DPP7 PE=1 SV=3 - [DPP2_HUMAN]	15,85	5	1,178
Q92485	Acid sphingomyelinase-like phosphodiesterase 3b OS=Homo sapiens GN=SMPDL3B PE=2 SV=2 - [ASM3B_HUMAN]	15,82	5	0,480
Q06136	3-ketodihydrosphingosine reductase OS=Homo sapiens GN=KDSR PE=1 SV=1 - [KDSR_HUMAN]	15,66	5	1,403
Q9BX95	Sphingosine-1-phosphate phosphatase 1 OS=Homo sapiens GN=SGPP1 PE=1 SV=2 - [SGPP1_HUMAN]	15,65	5	1,634
Q9GZR2	RNA exonuclease 4 OS=Homo sapiens GN=REXO4 PE=1 SV=2 - [REXO4_HUMAN]	15,64	5	0,954
P49768	Presenilin-1 OS=Homo sapiens GN=PSEN1 PE=1 SV=1 - [PSN1_HUMAN]	15,63	5	1,064
Q8TBR7	Protein FAM57A OS=Homo sapiens GN=FAM57A PE=1 SV=2 - [FA57A_HUMAN]	15,56	5	1,444
Q9Y285	Phenylalanine--tRNA ligase alpha subunit OS=Homo sapiens GN=FARSA PE=1 SV=3 - [SYFA_HUMAN]	15,35	5	0,773
P50552	Vasodilator-stimulated phosphoprotein OS=Homo sapiens GN=VASP PE=1 SV=3 - [VASP_HUMAN]	15,26	5	1,244
P37837	Transaldolase OS=Homo sapiens GN=TALDO1 PE=1 SV=2 - [TALDO_HUMAN]	15,13	5	0,802
P68400	Casein kinase II subunit alpha OS=Homo sapiens GN=CSNK2A1 PE=1 SV=1 - [CSK21_HUMAN]	15,09	5	1,055
P08574	Cytochrome c1, heme protein, mitochondrial OS=Homo sapiens GN=CYC1 PE=1 SV=3 - [CY1_HUMAN]	15,08	5	0,659
Q9H3K2	Growth hormone-inducible transmembrane protein OS=Homo sapiens GN=GHITM PE=1 SV=2 - [GHITM_HUMAN]	15,07	5	1,726
Q92947	Glutaryl-CoA dehydrogenase, mitochondrial OS=Homo sapiens GN=GCDH PE=1 SV=1 - [GCDH_HUMAN]	15,07	5	0,860
O15144	Actin-related protein 2/3 complex subunit 2 OS=Homo sapiens GN=ARPC2 PE=1 SV=1 - [ARPC2_HUMAN]	15,00	5	0,825
P43353	Aldehyde dehydrogenase family 3 member B1 OS=Homo sapiens GN=ALDH3B1 PE=1 SV=1 - [AL3B1_HUMAN]	14,96	5	0,814
Q96I25	Splicing factor 45 OS=Homo sapiens GN=RBM17 PE=1 SV=1 - [SPF45_HUMAN]	14,96	5	
Q92917	G patch domain and KOW motifs-containing protein OS=Homo sapiens GN=GPKOW PE=1 SV=2 - [GPKOW_HUMAN]	14,92	5	
Q9HD23	Magnesium transporter MRS2 homolog, mitochondrial OS=Homo sapiens GN=MRS2 PE=2 SV=1 - [MRS2_HUMAN]	14,90	5	0,979
O95084	Serine protease 23 OS=Homo sapiens GN=PRSS23 PE=1 SV=1 - [PRS23_HUMAN]	14,88	5	0,930
P13796	Plastin-2 OS=Homo sapiens GN=LCP1 PE=1 SV=6 - [PLSL_HUMAN]	14,83	5	0,271
Q9Y5Y0	Feline leukemia virus subgroup C receptor-related protein 1 OS=Homo sapiens GN=FLVCR1 PE=1 SV=1 - [FLVC1_HUMAN]	14,77	5	1,029
Q9C0H2	Protein tweety homolog 3 OS=Homo sapiens GN=TTYH3 PE=1 SV=3 - [TTYH3_HUMAN]	14,72	5	1,127
P36941	Tumor necrosis factor receptor superfamily member 3 OS=Homo sapiens GN=LTBR PE=1 SV=1 - [TNR3_HUMAN]	14,71	5	1,169
Q9Y5A9	YTH domain family protein 2 OS=Homo sapiens GN=YTHDF2 PE=1 SV=2 - [YTHD2_HUMAN]	14,68	5	1,087
P13726	Tissue factor OS=Homo sapiens GN=F3 PE=1 SV=1 - [TF_HUMAN]	14,58	5	0,514
Q3SXM5	Inactive hydroxysteroid dehydrogenase-like protein 1 OS=Homo sapiens GN=HSDL1 PE=1 SV=3 - [HSDL1_HUMAN]	14,55	5	1,163
Q10469	Alpha-1,6-mannosyl-glycoprotein 2-beta-N-acetylglucosaminyltransferase OS=Homo sapiens GN=MGAT2 PE=1 SV=1 - [MGAT2_HUMAN]	14,54	5	0,894
P17174	Aspartate aminotransferase, cytoplasmic OS=Homo sapiens GN=GOT1 PE=1 SV=3 - [AATC_HUMAN]	14,53	5	0,993
P52788	Spermine synthase OS=Homo sapiens GN=SMS PE=1 SV=2 - [SPSY_HUMAN]	14,48	5	0,665
O43252	Bifunctional 3'-phosphoadenosine 5'-phosphosulfate synthase 1 OS=Homo sapiens GN=PAPSS1 PE=1 SV=2 - [PAPS1_HUMAN]	14,26	5	0,652
Q9UJU6	Drebrin-like protein OS=Homo sapiens GN=DBNL PE=1 SV=1 - [DBNL_HUMAN]	14,19	5	0,964
Q96QD8	Sodium-coupled neutral amino acid transporter 2 OS=Homo sapiens GN=SLC38A2 PE=1 SV=2 - [S38A2_HUMAN]	14,03	5	1,011
Q6UWP7	Lysocardiolipin acyltransferase 1 OS=Homo sapiens GN=LCLAT1 PE=1 SV=1 - [LCLT1_HUMAN]	14,01	5	0,781
O00220	Tumor necrosis factor receptor superfamily member 10A OS=Homo sapiens GN=TNFRSF10A PE=1 SV=3 - [TR10A_HUMAN]	13,89	5	1,378
Q9NVN8	Guanine nucleotide-binding protein-like 3-like protein OS=Homo sapiens GN=GNL3L PE=1 SV=1 - [GNL3L_HUMAN]	13,75	5	1,173
Q8NFJ5	Retinoic acid-induced protein 3 OS=Homo sapiens GN=GPRC5A PE=1 SV=2 - [RAI3_HUMAN]	13,45	5	2,208
Q9Y305	Acyl-coenzyme A thioesterase 9, mitochondrial OS=Homo sapiens GN=ACOT9 PE=1 SV=2 - [ACOT9_HUMAN]	13,44	5	0,912
P15151	Poliovirus receptor OS=Homo sapiens GN=PVR PE=1 SV=2 - [PVR_HUMAN]	13,43	5	2,055
Q4G148	Glucoside xylosyltransferase 1 OS=Homo sapiens GN=GXYLT1 PE=1 SV=2 - [GXLT1_HUMAN]	13,41	5	0,980
O94919	Endonuclease domain-containing 1 protein OS=Homo sapiens GN=ENDOD1 PE=1 SV=2 - [ENDD1_HUMAN]	13,40	5	0,728
Q53EU6	Glycerol-3-phosphate acyltransferase 3 OS=Homo sapiens GN=AGPAT9 PE=1 SV=2 - [GPAT3_HUMAN]	13,36	5	0,824
Q9ULX3	RNA-binding protein NOB1 OS=Homo sapiens GN=NOB1 PE=1 SV=1 - [NOB1_HUMAN]	13,35	5	0,704
Q29983	MHC class I polypeptide-related sequence A OS=Homo sapiens GN=MICA PE=1 SV=1 - [MICA_HUMAN]	13,32	5	1,033
P06731	Carcinoembryonic antigen-related cell adhesion molecule 5 OS=Homo sapiens GN=CEACAM5 PE=1 SV=3 - [CEAM5_HUMAN]	13,25	5	0,110
Q9NUQ2	1-acyl-sn-glycerol-3-phosphate acyltransferase epsilon OS=Homo sapiens GN=AGPAT5 PE=1 SV=3 - [PLCE_HUMAN]	13,19	5	1,087
Q9BVC6	Transmembrane protein 109 OS=Homo sapiens GN=TMEM109 PE=1 SV=1 - [TM109_HUMAN]	13,17	5	1,427
P35637	RNA-binding protein FUS OS=Homo sapiens GN=FUS PE=1 SV=1 - [FUS_HUMAN]	13,12	5	1,025
Q16880	2-hydroxyacylsphingosine 1-beta-galactosyltransferase OS=Homo sapiens GN=UGT8 PE=2 SV=2 - [CGT_HUMAN]	13,12	5	1,031
P20073	Annexin A7 OS=Homo sapiens GN=ANXA7 PE=1 SV=3 - [ANXA7_HUMAN]	13,11	5	1,357
O60749	Sorting nexin-2 OS=Homo sapiens GN=SNX2 PE=1 SV=2 - [SNX2_HUMAN]	13,10	5	
Q86SR1	Polypeptide N-acetylgalactosaminyltransferase 10 OS=Homo sapiens GN=GALNT10 PE=1 SV=2 - [GLT10_HUMAN]	13,10	5	1,142
O14744	Protein arginine N-methyltransferase 5 OS=Homo sapiens GN=PRMT5 PE=1 SV=4 - [ANM5_HUMAN]	13,03	5	0,943
Q16222	UDP-N-acetylhexosamine pyrophosphorylase OS=Homo sapiens GN=UAP1 PE=1 SV=3 - [UAP1_HUMAN]	13,03	5	1,131
Q4KMP7	TBC1 domain family member 10B OS=Homo sapiens GN=TBC1D10B PE=1 SV=3 - [TB10B_HUMAN]	13,00	5	1,451
P36871	Phosphoglucomutase-1 OS=Homo sapiens GN=PGM1 PE=1 SV=3 - [PGM1_HUMAN]	12,99	5	0,862
P19438	Tumor necrosis factor receptor superfamily member 1A OS=Homo sapiens GN=TNFRSF1A PE=1 SV=1 - [TNR1A_HUMAN]	12,97	5	1,002
Q6NVY1	3-hydroxyisobutyryl-CoA hydrolase, mitochondrial OS=Homo sapiens GN=HIBCH PE=1 SV=2 - [HIBCH_HUMAN]	12,95	5	0,795
P05166	Propionyl-CoA carboxylase beta chain, mitochondrial OS=Homo sapiens GN=PCCB PE=1 SV=3 - [PCCB_HUMAN]	12,62	5	1,000
P26572	Alpha-1,3-mannosyl-glycoprotein 2-beta-N-acetylglucosaminyltransferase OS=Homo sapiens GN=MGAT1 PE=2 SV=2 - [MGAT1_HUMAN]	12,58	5	0,939
Q8IVS2	Malonyl-CoA-acyl carrier protein transacylase, mitochondrial OS=Homo sapiens GN=MCAT PE=1 SV=2 - [FABD_HUMAN]	12,56	5	0,749
O15460	Prolyl 4-hydroxylase subunit alpha-2 OS=Homo sapiens GN=P4HA2 PE=1 SV=1 - [P4HA2_HUMAN]	12,34	5	1,311
Q14728	Major facilitator superfamily domain-containing protein 10 OS=Homo sapiens GN=MFSD10 PE=2 SV=1 - [MFS10_HUMAN]	12,31	5	1,450
P42685	Tyrosine-protein kinase FRK OS=Homo sapiens GN=FRK PE=1 SV=1 - [FRK_HUMAN]	12,28	5	0,593
P41743	Protein kinase C iota type OS=Homo sapiens GN=PRKCI PE=1 SV=2 - [KPCI_HUMAN]	12,25	5	1,041
P52429	Diacylglycerol kinase epsilon OS=Homo sapiens GN=DGKE PE=2 SV=1 - [DGKE_HUMAN]	12,17	5	0,761
P11172	Uridine 5'-monophosphate synthase OS=Homo sapiens GN=UMPS PE=1 SV=1 - [UMPS_HUMAN]	11,88	5	0,725
P16278	Beta-galactosidase OS=Homo sapiens GN=GLB1 PE=1 SV=2 - [BGAL_HUMAN]	11,82	5	0,856
P61764	Syntaxin-binding protein 1 OS=Homo sapiens GN=STXBP1 PE=1 SV=1 - [STXB1_HUMAN]	11,78	5	1,806
Q8WXI9	Transcriptional repressor p66-beta OS=Homo sapiens GN=GATAD2B PE=1 SV=1 - [P66B_HUMAN]	11,47	5	
Q13510	Acid ceramidase OS=Homo sapiens GN=ASAH1 PE=1 SV=5 - [ASAH1_HUMAN]	11,39	5	0,703
Q7Z739	YTH domain family protein 3 OS=Homo sapiens GN=YTHDF3 PE=1 SV=1 - [YTHD3_HUMAN]	11,28	5	
Q969S9	Ribosome-releasing factor 2, mitochondrial OS=Homo sapiens GN=GFM2 PE=1 SV=1 - [RRF2M_HUMAN]	11,04	5	0,732
Q9BW04	Specifically androgen-regulated gene protein OS=Homo sapiens GN=SARG PE=1 SV=2 - [SARG_HUMAN]	10,98	5	
Q96GA3	Protein LTV1 homolog OS=Homo sapiens GN=LTV1 PE=1 SV=1 - [LTV1_HUMAN]	10,95	5	
Q6P1J9	Parafibromin OS=Homo sapiens GN=CDC73 PE=1 SV=1 - [CDC73_HUMAN]	10,92	5	
P61011	Signal recognition particle 54 kDa protein OS=Homo sapiens GN=SRP54 PE=1 SV=1 - [SRP54_HUMAN]	10,91	5	0,934
O15254	Peroxisomal acyl-coenzyme A oxidase 3 OS=Homo sapiens GN=ACOX3 PE=1 SV=2 - [ACOX3_HUMAN]	10,86	5	1,114
P52594	Arf-GAP domain and FG repeat-containing protein 1 OS=Homo sapiens GN=AGFG1 PE=1 SV=2 - [AGFG1_HUMAN]	10,85	5	
Q8IUH4	Palmitoyltransferase ZDHHC13 OS=Homo sapiens GN=ZDHHC13 PE=2 SV=3 - [ZDH13_HUMAN]	10,77	5	1,039
Q96CU9	FAD-dependent oxidoreductase domain-containing protein 1 OS=Homo sapiens GN=FOXRED1 PE=1 SV=2 - [FXRD1_HUMAN]	10,70	5	1,367
P23786	Carnitine O-palmitoyltransferase 2, mitochondrial OS=Homo sapiens GN=CPT2 PE=1 SV=2 - [CPT2_HUMAN]	10,64	5	0,430
O00499	Myc box-dependent-interacting protein 1 OS=Homo sapiens GN=BIN1 PE=1 SV=1 - [BIN1_HUMAN]	10,62	5	0,649
Q9NUT2	ATP-binding cassette sub-family B member 8, mitochondrial OS=Homo sapiens GN=ABCB8 PE=1 SV=3 - [ABCB8_HUMAN]	10,48	5	1,182
Q6P1A2	Lysophospholipid acyltransferase 5 OS=Homo sapiens GN=LPCAT3 PE=1 SV=1 - [MBOA5_HUMAN]	10,47	5	1,341
A0AV96	RNA-binding protein 47 OS=Homo sapiens GN=RBM47 PE=1 SV=2 - [RBM47_HUMAN]	10,46	5	0,726
Q9NS93	Transmembrane 7 superfamily member 3 OS=Homo sapiens GN=TM7SF3 PE=2 SV=1 - [TM7S3_HUMAN]	10,35	5	1,000
Q6PML9	Zinc transporter 9 OS=Homo sapiens GN=SLC30A9 PE=1 SV=1 - [ZNT9_HUMAN]	10,04	5	1,204
P23469	Receptor-type tyrosine-protein phosphatase epsilon OS=Homo sapiens GN=PTPRE PE=1 SV=1 - [PTPRE_HUMAN]	10,00	5	1,911
Q8WTT2	Nucleolar complex protein 3 homolog OS=Homo sapiens GN=NOC3L PE=1 SV=1 - [NOC3L_HUMAN]	10,00	5	0,985
Q9UPY5	Cystine/glutamate transporter OS=Homo sapiens GN=SLC7A11 PE=1 SV=1 - [XCT_HUMAN]	9,78	5	1,594
Q9H4A4	Aminopeptidase B OS=Homo sapiens GN=RNPEP PE=1 SV=2 - [AMPB_HUMAN]	9,69	5	0,678
P35269	General transcription factor IIF subunit 1 OS=Homo sapiens GN=GTF2F1 PE=1 SV=2 - [T2FA_HUMAN]	9,67	5	

Q99661	Kinesin-like protein KIF2C OS=Homo sapiens GN=KIF2C PE=1 SV=2 - [KIF2C_HUMAN]	9,66	5	1,817	0,731			0,978	2,004	1,216	[illegible]
P08237	6-phosphofructokinase, muscle type OS=Homo sapiens GN=PFKM PE=1 SV=2 - [K6PF_HUMAN]	9,49	5	0,822	0,856		1,055	0,905	0,912	0,886	[illegible]
O95302	Peptidyl-prolyl cis-trans isomerase FKBP9 OS=Homo sapiens GN=FKBP9 PE=1 SV=2 - [FKBP9_HUMAN]	9,47	5	1,147	0,798		1,202	1,190	1,138	0,980	[illegible]
Q14677	Clathrin interactor 1 OS=Homo sapiens GN=CLINT1 PE=1 SV=1 - [EPN4_HUMAN]	9,44	5	0,843			0,852	1,031	0,711		[illegible]
Q99640	Membrane-associated tyrosine- and threonine-specific cdc2-inhibitory kinase OS=Homo sapiens GN=PKMYT1 PE=1 SV=1 - [PMYT1_HUM	9,42	5	1,057	1,269				1,230	1,218	[illegible]
P33992	DNA replication licensing factor MCM5 OS=Homo sapiens GN=MCM5 PE=1 SV=5 - [MCM5_HUMAN]	9,40	5	0,531	0,501		0,709		0,718	0,612	[illegible]
P43246	DNA mismatch repair protein Msh2 OS=Homo sapiens GN=MSH2 PE=1 SV=1 - [MSH2_HUMAN]	9,31	5	0,578	0,843	0,831			0,930	1,155	[illegible]
Q14684	Ribosomal RNA processing protein 1 homolog B OS=Homo sapiens GN=RRP1B PE=1 SV=3 - [RRP1B_HUMAN]	9,23	5	0,999	1,399	1,178	1,067	1,126	1,302	1,123	[illegible]
Q9P2B4	CTTNBP2 N-terminal-like protein OS=Homo sapiens GN=CTTNBP2NL PE=1 SV=2 - [CT2NL_HUMAN]	9,23	5			1,068	0,982	0,945			[illegible]
Q9NPR9	Protein GPR108 OS=Homo sapiens GN=GPR108 PE=2 SV=3 - [GP108_HUMAN]	9,21	5	1,190	1,171				1,011	0,901	[illegible]
Q9BXP2	Solute carrier family 12 member 9 OS=Homo sapiens GN=SLC12A9 PE=1 SV=1 - [S12A9_HUMAN]	9,19	5	1,102	1,161			1,184	0,897	1,022	[illegible]
Q96D71	RalBP1-associated Eps domain-containing protein 1 OS=Homo sapiens GN=REPS1 PE=1 SV=3 - [REPS1_HUMAN]	9,05	5	1,076			0,977	1,050	0,966		[illegible]
Q9BW92	Threonine--tRNA ligase, mitochondrial OS=Homo sapiens GN=TARS2 PE=1 SV=1 - [SYTM_HUMAN]	9,05	5	1,166	0,588	1,441	0,775	0,989	0,728	0,426	[illegible]
Q9H2H9	Sodium-coupled neutral amino acid transporter 1 OS=Homo sapiens GN=SLC38A1 PE=1 SV=1 - [S38A1_HUMAN]	9,03	5	1,366	1,373				1,219	1,346	[illegible]
Q8IYB8	ATP-dependent RNA helicase SUPV3L1, mitochondrial OS=Homo sapiens GN=SUPV3L1 PE=1 SV=1 - [SUV3_HUMAN]	9,03	5	0,914	0,886	1,147	0,884	1,162	0,730	0,843	[illegible]
Q53F19	Uncharacterized protein C17orf85 OS=Homo sapiens GN=C17orf85 PE=1 SV=2 - [CQ085_HUMAN]	9,03	5				0,854	0,948			[illegible]
Q9H6T3	RNA polymerase II-associated protein 3 OS=Homo sapiens GN=RPAP3 PE=1 SV=2 - [RPAP3_HUMAN]	9,02	5	1,139		0,951		0,843	1,124		[illegible]
Q9NQW7	Xaa-Pro aminopeptidase 1 OS=Homo sapiens GN=XPNPEP1 PE=1 SV=3 - [XPP1_HUMAN]	8,99	5	0,793		1,012		0,853	0,838		[illegible]
O00469	Procollagen-lysine,2-oxoglutarate 5-dioxygenase 2 OS=Homo sapiens GN=PLOD2 PE=1 SV=2 - [PLOD2_HUMAN]	8,96	5	0,897	0,858			0,749	0,747	0,584	[illegible]
O43395	U4/U6 small nuclear ribonucleoprotein Prp3 OS=Homo sapiens GN=PRPF3 PE=1 SV=2 - [PRPF3_HUMAN]	8,93	5		1,053	0,736	0,839	0,774		1,290	[illegible]
Q9BU23	Lipase maturation factor 2 OS=Homo sapiens GN=LMF2 PE=1 SV=2 - [LMF2_HUMAN]	8,91	5	1,297	1,398		1,392		1,112	1,343	[illegible]
Q71RC2	La-related protein 4 OS=Homo sapiens GN=LARP4 PE=1 SV=3 - [LARP4_HUMAN]	8,84	5	0,782		1,196	1,026	1,080	1,163		[illegible]
Q6NYC8	Phostensin OS=Homo sapiens GN=PPP1R18 PE=1 SV=1 - [PPR18_HUMAN]	8,81	5			1,244	0,993	1,066			[illegible]
Q8TDB6	E3 ubiquitin-protein ligase DTX3L OS=Homo sapiens GN=DTX3L PE=1 SV=1 - [DTX3L_HUMAN]	8,78	5	0,446	0,527		0,792		0,468	0,609	[illegible]
Q9H6S3	Epidermal growth factor receptor kinase substrate 8-like protein 2 OS=Homo sapiens GN=EPS8L2 PE=1 SV=2 - [ES8L2_HUMAN]	8,53	5	1,583		0,596	0,632	0,860	1,202		[illegible]
O00186	Syntaxin-binding protein 3 OS=Homo sapiens GN=STXBP3 PE=1 SV=2 - [STXB3_HUMAN]	8,45	5	1,016	1,416			0,614	0,789	0,997	[illegible]
Q6UN15	Pre-mRNA 3'-end-processing factor FIP1 OS=Homo sapiens GN=FIP1L1 PE=1 SV=1 - [FIP1_HUMAN]	8,42	5			0,853	0,855	0,829			[illegible]
Q9P246	Stromal interaction molecule 2 OS=Homo sapiens GN=STIM2 PE=1 SV=2 - [STIM2_HUMAN]	8,31	5	1,139	0,925				1,049	1,076	[illegible]
Q12979	Active breakpoint cluster region-related protein OS=Homo sapiens GN=ABR PE=2 SV=2 - [ABR_HUMAN]	8,27	5	0,714	0,644	0,813	0,674		0,698	0,722	[illegible]
P49711	Transcriptional repressor CTCF OS=Homo sapiens GN=CTCF PE=1 SV=1 - [CTCF_HUMAN]	8,25	5	1,045		0,986	0,982	1,124	0,628		[illegible]
G5E9H4	Dynactin 1 (P150, glued homolog, Drosophila), isoform CRA_a OS=Homo sapiens GN=DCTN1 PE=4 SV=1 - [G5E9H4_HUMAN]	8,25	5	0,922		1,042		0,928	0,932		[illegible]
Q9NXH9	tRNA (guanine(26)-N(2))-dimethyltransferase OS=Homo sapiens GN=TRMT1 PE=1 SV=1 - [TRM1_HUMAN]	8,19	5		0,753	1,183	0,854	0,604		0,842	[illegible]
Q6PI48	Aspartate--tRNA ligase, mitochondrial OS=Homo sapiens GN=DARS2 PE=1 SV=1 - [SYDM_HUMAN]	8,06	5	0,633		1,176		1,241	0,738		[illegible]
P57740	Nuclear pore complex protein Nup107 OS=Homo sapiens GN=NUP107 PE=1 SV=1 - [NU107_HUMAN]	7,78	5	0,803	0,756	0,917	1,237	0,930	1,005	0,862	[illegible]
Q8IWX8	Calcium homeostasis endoplasmic reticulum protein OS=Homo sapiens GN=CHERP PE=1 SV=3 - [CHERP_HUMAN]	7,64	5	1,140		0,653	0,848	0,944	1,531		[illegible]
Q7L2J0	7SK snRNA methylphosphate capping enzyme OS=Homo sapiens GN=MEPCE PE=1 SV=1 - [MEPCE_HUMAN]	7,55	5			1,397	1,083	0,957			[illegible]
P29590-3	Isoform PML-8 of Protein PML OS=Homo sapiens GN=PML - [PML_HUMAN]	7,52	5		0,851		0,915			1,593	[illegible]
P53992	Protein transport protein Sec24C OS=Homo sapiens GN=SEC24C PE=1 SV=3 - [SC24C_HUMAN]	7,50	5	0,990		1,069	1,040	0,963	1,101		[illegible]
Q96T37	Putative RNA-binding protein 15 OS=Homo sapiens GN=RBM15 PE=1 SV=2 - [RBM15_HUMAN]	7,47	5			1,140	1,378	1,062			[illegible]
Q6JQN1	Acyl-CoA dehydrogenase family member 10 OS=Homo sapiens GN=ACAD10 PE=2 SV=1 - [ACD10_HUMAN]	7,46	5	0,608	0,863	1,273		0,874	0,649	0,697	[illegible]
Q92541	RNA polymerase-associated protein RTF1 homolog OS=Homo sapiens GN=RTF1 PE=1 SV=4 - [RTF1_HUMAN]	7,46	5			0,972	1,090	0,778			[illegible]
Q13433	Zinc transporter ZIP6 OS=Homo sapiens GN=SLC39A6 PE=1 SV=3 - [S39A6_HUMAN]	7,42	5	1,983	1,976				0,966	1,065	[illegible]
O43292	Glycosylphosphatidylinositol anchor attachment 1 protein OS=Homo sapiens GN=GPAA1 PE=1 SV=3 - [GPAA1_HUMAN]	7,41	5	0,979	1,140		1,006	1,037	1,045	1,043	[illegible]
Q86WA8	Lon protease homolog 2, peroxisomal OS=Homo sapiens GN=LONP2 PE=1 SV=1 - [LONP2_HUMAN]	7,28	5	1,031	1,111		0,769		1,009	1,419	[illegible]
P49959	Double-strand break repair protein MRE11A OS=Homo sapiens GN=MRE11A PE=1 SV=3 - [MRE11_HUMAN]	7,20	5	0,792	0,900	1,406	1,344	1,126	0,868	1,097	[illegible]
Q5R3F8	Protein phosphatase 1 regulatory subunit 29 OS=Homo sapiens GN=ELFN2 PE=1 SV=1 - [PPR29_HUMAN]	7,20	5	1,226	1,169				1,093	1,022	[illegible]
Q6PJT7	Zinc finger CCCH domain-containing protein 14 OS=Homo sapiens GN=ZC3H14 PE=1 SV=1 - [ZC3HE_HUMAN]	7,20	5			1,002	0,807	0,992			[illegible]
Q13618	Cullin-3 OS=Homo sapiens GN=CUL3 PE=1 SV=2 - [CUL3_HUMAN]	7,16	5	0,723	0,941		0,739		0,957	1,064	[illegible]
Q9P275	Ubiquitin carboxyl-terminal hydrolase 36 OS=Homo sapiens GN=USP36 PE=1 SV=3 - [UBP36_HUMAN]	6,96	5		1,039	1,205	1,265	0,962		1,055	[illegible]
P04035	3-hydroxy-3-methylglutaryl-coenzyme A reductase OS=Homo sapiens GN=HMGCR PE=1 SV=1 - [HMDH_HUMAN]	6,87	5	0,969	0,825				1,335	1,376	[illegible]
Q9HCS7	Pre-mRNA-splicing factor SYF1 OS=Homo sapiens GN=XAB2 PE=1 SV=2 - [SYF1_HUMAN]	6,78	5	0,989			1,008	0,960	0,898		[illegible]
Q8IYB3	Serine/arginine repetitive matrix protein 1 OS=Homo sapiens GN=SRRM1 PE=1 SV=2 - [SRRM1_HUMAN]	6,75	5		1,372	0,804	0,919	0,942		1,053	[illegible]
Q14114	Low-density lipoprotein receptor-related protein 8 OS=Homo sapiens GN=LRP8 PE=1 SV=4 - [LRP8_HUMAN]	6,65	5	1,053	1,433				1,070	1,523	[illegible]
Q9H2P0	Activity-dependent neuroprotector homeobox protein OS=Homo sapiens GN=ADNP PE=1 SV=1 - [ADNP_HUMAN]	6,62	5			1,197		0,926			[illegible]
O60885	Bromodomain-containing protein 4 OS=Homo sapiens GN=BRD4 PE=1 SV=2 - [BRD4_HUMAN]	6,61	5			0,953	1,038	1,382			[illegible]
Q9Y2H0	Disks large-associated protein 4 OS=Homo sapiens GN=DLGAP4 PE=1 SV=3 - [DLGP4_HUMAN]	6,55	5	1,365	1,221	1,286	1,311		1,055	0,981	[illegible]
Q3T906	N-acetylglucosamine-1-phosphotransferase subunits alpha/beta OS=Homo sapiens GN=GNPTAB PE=1 SV=1 - [GNPTA_HUMAN]	6,53	5	0,694	0,858				0,810	0,824	[illegible]
Q9H3U1	Protein unc-45 homolog A OS=Homo sapiens GN=UNC45A PE=1 SV=1 - [UN45A_HUMAN]	6,46	5	0,787	0,697	0,783			1,092	0,944	[illegible]
Q96EV2	RNA-binding protein 33 OS=Homo sapiens GN=RBM33 PE=1 SV=3 - [RBM33_HUMAN]	6,41	5	1,377		1,233		0,809	1,303		[illegible]
Q8IWB9	Testis-expressed sequence 2 protein OS=Homo sapiens GN=TEX2 PE=1 SV=2 - [TEX2_HUMAN]	6,39	5	1,500	0,915				1,652	0,880	[illegible]
Q6ZRV2	Protein FAM83H OS=Homo sapiens GN=FAM83H PE=1 SV=3 - [FA83H_HUMAN]	6,36	5	0,650		0,835		0,775	0,770		[illegible]
Q9P0K7	Ankycorbin OS=Homo sapiens GN=RAI14 PE=1 SV=2 - [RAI14_HUMAN]	6,33	5	0,849		2,064		1,176	0,880		[illegible]
Q9H2G2	STE20-like serine/threonine-protein kinase OS=Homo sapiens GN=SLK PE=1 SV=1 - [SLK_HUMAN]	6,32	5	1,092	0,946	1,001	0,928		1,229	1,060	[illegible]
Q9GZR7	ATP-dependent RNA helicase DDX24 OS=Homo sapiens GN=DDX24 PE=1 SV=1 - [DDX24_HUMAN]	6,29	5			0,709	0,686				[illegible]
Q86Y56	HEAT repeat-containing protein 2 OS=Homo sapiens GN=HEATR2 PE=1 SV=4 - [HEAT2_HUMAN]	6,20	5		0,841	0,907	1,099	1,126		1,194	[illegible]
Q96A65	Exocyst complex component 4 OS=Homo sapiens GN=EXOC4 PE=1 SV=1 - [EXOC4_HUMAN]	6,16	5	0,892	0,829				0,707	0,763	[illegible]
Q5H8A4	GPI ethanolamine phosphate transferase 2 OS=Homo sapiens GN=PIGG PE=1 SV=1 - [PIGG_HUMAN]	6,10	5	0,734	0,646				0,573	0,413	[illegible]
Q9NQS7	Inner centromere protein OS=Homo sapiens GN=INCENP PE=1 SV=3 - [INCE_HUMAN]	6,10	5	1,115		1,058	0,712	0,880	0,662		[illegible]
O43747	AP-1 complex subunit gamma-1 OS=Homo sapiens GN=AP1G1 PE=1 SV=5 - [AP1G1_HUMAN]	5,84	5	0,499	0,827	1,055		1,061	0,737	0,842	[illegible]
Q9HAV4	Exportin-5 OS=Homo sapiens GN=XPO5 PE=1 SV=1 - [XPO5_HUMAN]	5,81	5	0,796	1,100	1,166	1,275	1,362	1,038	1,047	[illegible]
Q9UPT8	Zinc finger CCCH domain-containing protein 4 OS=Homo sapiens GN=ZC3H4 PE=1 SV=3 - [ZC3H4_HUMAN]	5,76	5			1,075	0,960	0,911			[illegible]
Q9HAU0	Pleckstrin homology domain-containing family A member 5 OS=Homo sapiens GN=PLEKHA5 PE=1 SV=1 - [PKHA5_HUMAN]	5,65	5			0,762	0,715	0,762			[illegible]
O75882	Attractin OS=Homo sapiens GN=ATRN PE=1 SV=2 - [ATRN_HUMAN]	5,60	5	1,023	0,946				1,063	0,790	[illegible]
P14735	Insulin-degrading enzyme OS=Homo sapiens GN=IDE PE=1 SV=4 - [IDE_HUMAN]	5,59	5	0,857	0,770	0,875	1,206	1,104	1,056	0,907	[illegible]
O94887	FERM, RhoGEF and pleckstrin domain-containing protein 2 OS=Homo sapiens GN=FARP2 PE=1 SV=3 - [FARP2_HUMAN]	5,50	5	0,905	0,868			0,923	0,746	0,852	[illegible]
O43847	Nardilysin OS=Homo sapiens GN=NRD1 PE=1 SV=2 - [NRDC_HUMAN]	5,48	5	0,915	0,897	0,775	0,707	0,989	1,097	1,047	[illegible]
Q86VI3	Ras GTPase-activating-like protein IQGAP3 OS=Homo sapiens GN=IQGAP3 PE=1 SV=2 - [IQGA3_HUMAN]	5,40	5	0,730			0,815	0,853	0,792		[illegible]
Q86UU1	Pleckstrin homology-like domain family B member 1 OS=Homo sapiens GN=PHLDB1 PE=1 SV=1 - [PHLB1_HUMAN]	5,23	5	0,760		1,399		1,613	1,277		[illegible]
P51828	Adenylate cyclase type 7 OS=Homo sapiens GN=ADCY7 PE=2 SV=1 - [ADCY7_HUMAN]	5,19	5	1,096	0,704				1,106	0,819	[illegible]
Q02952	A-kinase anchor protein 12 OS=Homo sapiens GN=AKAP12 PE=1 SV=4 - [AKA12_HUMAN]	4,94	5	**2,365**	1,945				1,614	1,528	[illegible]
Q14C86	GTPase-activating protein and VPS9 domain-containing protein 1 OS=Homo sapiens GN=GAPVD1 PE=1 SV=2 - [GAPD1_HUMAN]	4,94	5	0,733	0,786	0,763		1,062	0,776	0,966	[illegible]
O75179	Ankyrin repeat domain-containing protein 17 OS=Homo sapiens GN=ANKRD17 PE=1 SV=3 - [ANR17_HUMAN]	4,84	5	0,998	0,735	0,958	0,894		0,916	0,943	[illegible]
P78316	Nucleolar protein 14 OS=Homo sapiens GN=NOP14 PE=1 SV=3 - [NOP14_HUMAN]	4,78	5		1,102		1,337	1,347		1,178	[illegible]
Q8TEQ6	Gem-associated protein 5 OS=Homo sapiens GN=GEMIN5 PE=1 SV=3 - [GEMI5_HUMAN]	4,64	5	1,285			1,236	1,144	0,894		[illegible]
O75962	Triple functional domain protein OS=Homo sapiens GN=TRIO PE=1 SV=2 - [TRIO_HUMAN]	4,59	5	**2,335**	1,652				1,334	1,078	[illegible]
Q9Y5S2	Serine/threonine-protein kinase MRCK beta OS=Homo sapiens GN=CDC42BPB PE=1 SV=2 - [MRCKB_HUMAN]	4,56	5	0,952	0,831	0,704		0,887	1,056	0,874	[illegible]
Q7Z3T8	Zinc finger FYVE domain-containing protein 16 OS=Homo sapiens GN=ZFYVE16 PE=1 SV=3 - [ZFY16_HUMAN]	4,55	5			1,235	1,074	0,831			[illegible]
Q13523	Serine/threonine-protein kinase PRP4 homolog OS=Homo sapiens GN=PRPF4B PE=1 SV=3 - [PRP4B_HUMAN]	4,37	5			1,006	0,946	0,939			[illegible]
Q15477	Helicase SKI2W OS=Homo sapiens GN=SKIV2L PE=1 SV=3 - [SKIV2_HUMAN]	4,25	5			0,953	0,906	1,154			[illegible]
Q9H2M9	Rab3 GTPase-activating protein non-catalytic subunit OS=Homo sapiens GN=RAB3GAP2 PE=1 SV=1 - [RBGPR_HUMAN]	4,16	5		0,953	0,797		0,840		0,775	[illegible]
Q9Y6D6	Brefeldin A-inhibited guanine nucleotide-exchange protein 1 OS=Homo sapiens GN=ARFGEF1 PE=1 SV=2 - [BIG1_HUMAN]	3,95	5	0,717			0,957	0,828	0,797		[illegible]
Q8WWI1	LIM domain only protein 7 OS=Homo sapiens GN=LMO7 PE=1 SV=3 - [LMO7_HUMAN]	3,68	5	0,411	0,517	0,415	0,699	0,623	0,419	0,556	[illegible]
Q13459	Unconventional myosin-IXb OS=Homo sapiens GN=MYO9B PE=1 SV=3 - [MYO9B_HUMAN]	3,43	5	0,775		0,973	1,087		0,869		[illegible]
O14497	AT-rich interactive domain-containing protein 1A OS=Homo sapiens GN=ARID1A PE=1 SV=3 - [ARI1A_HUMAN]	3,37	5			0,900	0,892				[illegible]
Q14643	Inositol 1,4,5-trisphosphate receptor type 1 OS=Homo sapiens GN=ITPR1 PE=1 SV=2 - [ITPR1_HUMAN]	3,34	5	1,367	1,483				1,091	1,398	[illegible]
Q7Z5J4	Retinoic acid-induced protein 1 OS=Homo sapiens GN=RAI1 PE=1 SV=2 - [RAI1_HUMAN]	3,04	5			0,913	0,836	0,743			[illegible]
Q9HC84	Mucin-5B OS=Homo sapiens GN=MUC5B PE=1 SV=3 - [MUC5B_HUMAN]	2,55	5	0,223	0,284				0,229	0,413	[illegible]
O75691	Small subunit processome component 20 homolog OS=Homo sapiens GN=UTP20 PE=1 SV=3 - [UTP20_HUMAN]	1,90	5	0,671	1,060	1,058	1,061	1,254	0,755	0,939	[illegible]
O43504	Hepatitis B virus X-interacting protein OS=Homo sapiens GN=HBXIP PE=1 SV=1 - [HBXIP_HUMAN]	81,32	4	1,117	1,389	0,657			1,137	1,046	[illegible]
Q9H0U4	Ras-related protein Rab-1B OS=Homo sapiens GN=RAB1B PE=1 SV=1 - [RAB1B_HUMAN]	63,18	4	1,209	1,152	0,912		0,949	1,019	0,982	[illegible]
P61586	Transforming protein RhoA OS=Homo sapiens GN=RHOA PE=1 SV=1 - [RHOA_HUMAN]	54,92	4	1,344	1,284	1,525	1,451	0,920	1,462	1,460	[illegible]
Q96FQ6	Protein S100-A16 OS=Homo sapiens GN=S100A16 PE=1 SV=1 - [S10AG_HUMAN]	52,43	4	0,564	0,671				0,617	0,698	[illegible]
P63313	Thymosin beta-10 OS=Homo sapiens GN=TMSB10 PE=1 SV=2 - [TYB10_HUMAN]	52,27	4		1,229	0,872	0,966	0,928		1,276	[illegible]
P35268	60S ribosomal protein L22 OS=Homo sapiens GN=RPL22 PE=1 SV=2 - [RL22_HUMAN]	50,78	4	0,575	0,661	0,929	0,871	0,910	0,663	0,740	[illegible]
P62328	Thymosin beta-4 OS=Homo sapiens GN=TMSB4X PE=1 SV=2 - [TYB4_HUMAN]	50,00	4			0,767	0,790	0,836			[illegible]
P62888	60S ribosomal protein L30 OS=Homo sapiens GN=RPL30 PE=1 SV=2 - [RL30_HUMAN]	48,70	4	0,560	0,662	1,225	0,995	0,929	0,712	0,764	[illegible]
Q9Y5J7	Mitochondrial import inner membrane translocase subunit Tim9 OS=Homo sapiens GN=TIMM9 PE=1 SV=1 - [TIM9_HUMAN]	48,31	4	1,250	1,283	1,484			0,972	1,080	[illegible]
Q69YU5	Uncharacterized protein C12orf73 OS=Homo sapiens GN=C12orf73 PE=2 SV=2 - [CL073_HUMAN]	47,89	4	1,182	1,331				0,979	0,996	[illegible]
Q8N4H5	Mitochondrial import receptor subunit TOM5 homolog OS=Homo sapiens GN=TOMM5 PE=1 SV=1 - [TOM5_HUMAN]	47,06	4	1,396	1,176			0,965	1,474	1,223	[illegible]
O75380	NADH dehydrogenase [ubiquinone] iron-sulfur protein 6, mitochondrial OS=Homo sapiens GN=NDUFS6 PE=1 SV=1 - [NDUS6_HUMAN]	46,77	4	0,937	1,111			0,858	1,320	1,586	[illegible]
Q9NX55	Huntingtin-interacting protein K OS=Homo sapiens GN=HYPK PE=1 SV=2 - [HYPK_HUMAN]	44,96	4	1,251			2,236	1,195	1,248		[illegible]
O14737	Programmed cell death protein 5 OS=Homo sapiens GN=PDCD5 PE=1 SV=3 - [PDCD5_HUMAN]	44,80	4	1,104	1,042	1,447	1,650	1,143	2,055	1,555	[illegible]
Q9C002	Normal mucosa of esophagus-specific gene 1 protein OS=Homo sapiens GN=NMES1 PE=2 SV=1 - [NMES1_HUMAN]	43,37	4	0,522	0,545	0,652			0,367	0,358	[illegible]
P63000-2	Isoform B of Ras-related C3 botulinum toxin substrate 1 OS=Homo sapiens GN=RAC1 - [RAC1_HUMAN]	43,13	4	1,175	1,140	1,017	0,821	1,134	1,434	1,264	[illegible]
P62834	Ras-related protein Rap-1A OS=Homo sapiens GN=RAP1A PE=1 SV=1 - [RAP1A_HUMAN]	42,93	4	1,439	1,502	1,083	1,187	0,959	1,340	1,343	[illegible]
P68036	Ubiquitin-conjugating enzyme E2 L3 OS=Homo sapiens GN=UBE2L3 PE=1 SV=1 - [UB2L3_HUMAN]	41,56	4	0,681	1,066	0,844	0,798	0,765	1,142	1,576	[illegible]
Q99584	Protein S100-A13 OS=Homo sapiens GN=S100A13 PE=1 SV=1 - [S10AD_HUMAN]	39,80	4	0,899	0,770		0,676	0,617	0,837	0,845	[illegible]
Q9Y2Q5	Ragulator complex protein LAMTOR2 OS=Homo sapiens GN=LAMTOR2 PE=1 SV=1 - [LTOR2_HUMAN]	39,20	4	0,958	1,038				1,073	0,932	[illegible]
P15153	Ras-related C3 botulinum toxin substrate 2 OS=Homo sapiens GN=RAC2 PE=1 SV=1 - [RAC2_HUMAN]	39,06	4	0,882	0,880			0,931	1,102	0,988	[illegible]
O14949	Cytochrome b-c1 complex subunit 8 OS=Homo sapiens GN=UQCRQ PE=1 SV=4 - [QCR8_HUMAN]	39,02	4	0,746	0,566		0,690		1,212	1,237	[illegible]
O00762	Ubiquitin-conjugating enzyme E2 C OS=Homo sapiens GN=UBE2C PE=1 SV=1 - [UBE2C_HUMAN]	38,55	4	0,717			0,870	0,742	0,947		[illegible]
Q9Y6H1	Coiled-coil-helix-coiled-coil-helix domain-containing protein 2, mitochondrial OS=Homo sapiens GN=CHCHD2 PE=1 SV=1 - [CHCH2_HUM	38,41	4	0,477	0,603	0,801	0,636	0,792	0,730	0,733	[illegible]
Q9BRA2	Thioredoxin domain-containing protein 17 OS=Homo sapiens GN=TXNDC17 PE=1 SV=1 - [TXD17_HUMAN]	38,21	4	0,628	0,687		0,685	0,810	0,973	0,865	[illegible]
P62854	40S ribosomal protein S26 OS=Homo sapiens GN=RPS26 PE=1 SV=3 - [RS26_HUMAN]	37,39	4	0,984	0,951	0,982	0,994	1,001	1,041	1,041	[illegible]
Q8IXM2	Chromatin complexes subunit BAP18 OS=Homo sapiens GN=BAP18 PE=1 SV=1 - [BAP18_HUMAN]	37,21	4			0,987	0,858				[illegible]
Q01469	Fatty acid-binding protein, epidermal OS=Homo sapiens GN=FABP5 PE=1 SV=3 - [FABP5_HUMAN]	37,04	4	0,715	0,715	0,940	0,848	1,081	1,639	1,569	[illegible]
Q9GZY4	Uncharacterized protein C7orf44 OS=Homo sapiens GN=C7orf44 PE=1 SV=1 - [CG044_HUMAN]	36,99	4	1,368	1,548				1,003	1,290	[illegible]
P61225	Ras-related protein Rap-2b OS=Homo sapiens GN=RAP2B PE=1 SV=1 - [RAP2B_HUMAN]	35,52	4	0,861	1,074				1,000	0,988	[illegible]
P61803	Dolichyl-diphosphooligosaccharide--protein glycosyltransferase subunit DAD1 OS=Homo sapiens GN=DAD1 PE=1 SV=3 - [DAD1_HUMAN]	35,40	4	1,008	1,097				0,883	0,917	[illegible]
P62879	Guanine nucleotide-binding protein G(I)/G(S)/G(T) subunit beta-2 OS=Homo sapiens GN=GNB2 PE=1 SV=3 - [GBB2_HUMAN]	34,71	4	1,526	1,354	0,868	1,531	1,036	0,928	0,919	[illegible]
P31949	Protein S100-A11 OS=Homo sapiens GN=S100A11 PE=1 SV=2 - [S10AB_HUMAN]	34,29	4	0,405	1,224	1,249	1,151	1,043	0,486	0,909	[illegible]
P55769	NHP2-like protein 1 OS=Homo sapiens GN=NHP2L1 PE=1 SV=3 - [NH2L1_HUMAN]	33,59	4	0,744	1,238	1,080	0,929	1,083	1,048	1,278	[illegible]
Q01105	Protein SET OS=Homo sapiens GN=SET PE=1 SV=3 - [SET_HUMAN]	33,10	4		0,714	0,778	0,940			0,775	[illegible]
O43920	NADH dehydrogenase [ubiquinone] iron-sulfur protein 5 OS=Homo sapiens GN=NDUFS5 PE=1 SV=3 - [NDUS5_HUMAN]	33,02	4	1,352	1,380		1,443		1,435	1,443	[illegible]
Q86YS6	Ras-related protein Rab-43 OS=Homo sapiens GN=RAB43 PE=1 SV=1 - [RAB43_HUMAN]	33,02	4	0,621	0,682				0,632	0,861	[illegible]
Q7Z5G4	Golgin subfamily A member 7 OS=Homo sapiens GN=GOLGA7 PE=1 SV=2 - [GOGA7_HUMAN]	32,12	4	1,425	1,321				1,166	1,224	[illegible]
O95168	NADH dehydrogenase [ubiquinone] 1 beta subcomplex subunit 4 OS=Homo sapiens GN=NDUFB4 PE=1 SV=3 - [NDUB4_HUMAN]	31,78	4	1,125	1,242				1,175	1,387	[illegible]
Q9H6K4	Optic atrophy 3 protein OS=Homo sapiens GN=OPA3 PE=1 SV=1 - [OPA3_HUMAN]	31,28	4	1,549	1,149				1,306	1,359	[illegible]
O95139	NADH dehydrogenase [ubiquinone] 1 beta subcomplex subunit 6 OS=Homo sapiens GN=NDUFB6 PE=1 SV=3 - [NDUB6_HUMAN]	31,25	4	1,194	1,216		0,859	1,177	1,246	1,196	[illegible]
O00194	Ras-related protein Rab-27B OS=Homo sapiens GN=RAB27B PE=1 SV=4 - [RB27B_HUMAN]	31,19	4	0,281	0,289				0,284	0,259	[illegible]
Q99471	Prefoldin subunit 5 OS=Homo sapiens GN=PFDN5 PE=1 SV=2 - [PFD5_HUMAN]	31,17	4	0,641	0,900	0,946	1,069	0,885	0,800	1,678	[illegible]
Q9NP84	Tumor necrosis factor receptor superfamily member 12A OS=Homo sapiens GN=TNFRSF12A PE=1 SV=1 - [TNR12_HUMAN]	31,01	4	1,697	1,301			1,112	1,791	1,740	[illegible]
P35613	Basigin OS=Homo sapiens GN=BSG PE=1 SV=2 - [BASI_HUMAN]	30,91	4	1,855	2,030		1,403		1,507	1,674	[illegible]
P05161	Ubiquitin-like protein ISG15 OS=Homo sapiens GN=ISG15 PE=1 SV=5 - [ISG15_HUMAN]	30,91	4	0,384	0,186	0,543	0,289		0,324	0,276	[illegible]
P63096	Guanine nucleotide-binding protein G(i) subunit alpha-1 OS=Homo sapiens GN=GNAI1 PE=1 SV=2 - [GNAI1_HUMAN]	30,79	4	**2,717**	**2,124**				1,234	1,079	[illegible]
Q9Y6C9	Mitochondrial carrier homolog 2 OS=Homo sapiens GN=MTCH2 PE=1 SV=1 - [MTCH2_HUMAN]	30,69	4	1,629	1,401				1,246	1,142	[illegible]
P61513	60S ribosomal protein L37a OS=Homo sapiens GN=RPL37A PE=1 SV=2 - [RL37A_HUMAN]	30,43	4	1,053	0,973	1,192	1,096		1,004	1,065	[illegible]
Q96C01	Protein FAM136A OS=Homo sapiens GN=FAM136A PE=1 SV=1 - [F136A_HUMAN]	29,71	4		0,584	0,879		0,848		0,535	[illegible]
O15212	Prefoldin subunit 6 OS=Homo sapiens GN=PFDN6 PE=1 SV=1 - [PFD6_HUMAN]	29,46	4			0,923	0,806	0,926			[illegible]
Q9Y2Q9	28S ribosomal protein S28, mitochondrial OS=Homo sapiens GN=MRPS28 PE=1 SV=1 - [RT28_HUMAN]	29,41	4		0,968		1,236	1,080		0,908	[illegible]
P83881	60S ribosomal protein L36a OS=Homo sapiens GN=RPL36A PE=1 SV=2 - [RL36A_HUMAN]	29,25	4	0,956	0,961	1,146	1,389	1,178	0,814	0,871	[illegible]
Q9Y3D3	28S ribosomal protein S16, mitochondrial OS=Homo sapiens GN=MRPS16 PE=1 SV=1 - [RT16_HUMAN]	29,20	4	1,092	1,013		1,088		0,928	0,885	[illegible]
P82930	28S ribosomal protein S34, mitochondrial OS=Homo sapiens GN=MRPS34 PE=1 SV=2 - [RT34_HUMAN]	28,90	4	1,168	1,177	1,090	1,069	1,125	0,944	0,832	[illegible]
P13498	Cytochrome b-245 light chain OS=Homo sapiens GN=CYBA PE=1 SV=3 - [CY24A_HUMAN]	28,72	4	0,879	0,786				0,999	0,927	[illegible]
P62341	Selenoprotein T OS=Homo sapiens GN=SELT PE=2 SV=2 - [SELT_HUMAN]	28,72	4	1,113	1,022				0,892	0,812	[illegible]
P20338	Ras-related protein Rab-4A OS=Homo sapiens GN=RAB4A PE=1 SV=2 - [RAB4A_HUMAN]	28,64	4	1,414	1,169				1,177	1,184	[illegible]
O60739	Eukaryotic translation initiation factor 1b OS=Homo sapiens GN=EIF1B PE=1 SV=2 - [EIF1B_HUMAN]	28,32	4		1,012	1,315		1,169		1,304	[illegible]
Q96C57	Uncharacterized protein C12orf43 OS=Homo sapiens GN=C12orf43 PE=1 SV=2 - [CL043_HUMAN]	28,24	4			1,004	0,780				[illegible]
Q9BY32	Inosine triphosphate pyrophosphatase OS=Homo sapiens GN=ITPA PE=1 SV=2 - [ITPA_HUMAN]	27,84	4	1,088	1,056		1,196	1,228	1,172	1,114	[illegible]
P80188	Neutrophil gelatinase-associated lipocalin OS=Homo sapiens GN=LCN2 PE=1 SV=2 - [NGAL_HUMAN]	27,78	4	0,198	0,339				0,260	0,573	[illegible]
Q13795	ADP-ribosylation factor-related protein 1 OS=Homo sapiens GN=ARFRP1 PE=1 SV=1 - [ARFRP_HUMAN]	27,36	4	0,852	0,936				1,098	0,988	[illegible]
Q9BQE4	Selenoprotein S OS=Homo sapiens GN=SELS PE=1 SV=3 - [SELS_HUMAN]	26,98	4	1,198	1,502				1,134	1,397	[illegible]
Q9BSD7	Cancer-related nucleoside-triphosphatase OS=Homo sapiens GN=NTPCR PE=1 SV=1 - [NTPCR_HUMAN]	26,32	4	0,731			1,135	0,999	1,067		[illegible]
P39210	Protein Mpv17 OS=Homo sapiens GN=MPV17 PE=1 SV=1 - [MPV17_HUMAN]	26,14	4	1,168	0,982	0,742			0,957	0,807	[illegible]
Q9BUF5	Tubulin beta-6 chain OS=Homo sapiens GN=TUBB6 PE=1 SV=1 - [TBB6_HUMAN]	25,78	4	1,169	0,684			1,257	1,617	1,445	[illegible]
O00422	Histone deacetylase complex subunit SAP18 OS=Homo sapiens GN=SAP18 PE=1 SV=1 - [SAP18_HUMAN]	25,49	4	1,174		1,270	1,302	1,056	0,945		[illegible]
Q9NQ50	39S ribosomal protein L40, mitochondrial OS=Homo sapiens GN=MRPL40 PE=1 SV=1 - [RM40_HUMAN]	25,24	4	0,735	0,565	1,014	0,817	1,131	0,681	0,616	[illegible]
O95563	Brain protein 44 OS=Homo sapiens GN=BRP44 PE=1 SV=1 - [BR44_HUMAN]	25,20	4	1,325	1,433				1,001	0,966	[illegible]
O15511	Actin-related protein 2/3 complex subunit 5 OS=Homo sapiens GN=ARPC5 PE=1 SV=3 - [ARPC5_HUMAN]	25,17	4	0,894	0,742	0,819	0,736	0,796	0,971	0,722	[illegible]
P62745	Rho-related GTP-binding protein RhoB OS=Homo sapiens GN=RHOB PE=1 SV=1 - [RHOB_HUMAN]	25,00	4	**2,412**	**2,968**		0,823		1,604	2,077	[illegible]

Q16563	Synaptophysin-like protein 1 OS=Homo sapiens GN=SYPL1 PE=1 SV=1 - [SYPL1_HUMAN]	24,71	4	1,214	1,247	[illegible]
Q9UII2	ATPase inhibitor, mitochondrial OS=Homo sapiens GN=ATPIF1 PE=1 SV=1 - [ATIF1_HUMAN]	24,53	4	0,906	0,887	[illegible]
P50148	Guanine nucleotide-binding protein G(q) subunit alpha OS=Homo sapiens GN=GNAQ PE=1 SV=4 - [GNAQ_HUMAN]	24,51	4	2,089	1,591	[illegible]
Q15382	GTP-binding protein Rheb OS=Homo sapiens GN=RHEB PE=1 SV=1 - [RHEB_HUMAN]	24,46	4	0,926	1,025	[illegible]
Q15631	Translin OS=Homo sapiens GN=TSN PE=1 SV=1 - [TSN_HUMAN]	24,12	4	0,854		[illegible]
Q9NRX2	39S ribosomal protein L17, mitochondrial OS=Homo sapiens GN=MRPL17 PE=1 SV=1 - [RM17_HUMAN]	24,00	4	0,757	0,915	[illegible]
Q8TED1	Probable glutathione peroxidase 8 OS=Homo sapiens GN=GPX8 PE=1 SV=2 - [GPX8_HUMAN]	23,92	4	0,846	0,925	[illegible]
P52566	Rho GDP-dissociation inhibitor 2 OS=Homo sapiens GN=ARHGDIB PE=1 SV=3 - [GDIR2_HUMAN]	23,88	4	0,481	0,395	[illegible]
P62263	40S ribosomal protein S14 OS=Homo sapiens GN=RPS14 PE=1 SV=3 - [RS14_HUMAN]	23,84	4	0,866	0,753	[illegible]
P61758	Prefoldin subunit 3 OS=Homo sapiens GN=VBP1 PE=1 SV=3 - [PFD3_HUMAN]	23,35	4	0,558		[illegible]
Q9NWU5	39S ribosomal protein L22, mitochondrial OS=Homo sapiens GN=MRPL22 PE=1 SV=1 - [RM22_HUMAN]	23,30	4	0,744	0,741	[illegible]
P47914	60S ribosomal protein L29 OS=Homo sapiens GN=RPL29 PE=1 SV=2 - [RL29_HUMAN]	23,27	4	1,120	1,099	[illegible]
P30626	Sorcin OS=Homo sapiens GN=SRI PE=1 SV=1 - [SORCN_HUMAN]	23,23	4	1,137	1,732	[illegible]
P62166	Neuronal calcium sensor 1 OS=Homo sapiens GN=NCS1 PE=1 SV=2 - [NCS1_HUMAN]	23,16	4	1,593	1,558	[illegible]
Q8IUX1	Transmembrane protein 126B OS=Homo sapiens GN=TMEM126B PE=2 SV=2 - [T126B_HUMAN]	23,04	4	1,141	1,196	[illegible]
P17152	Transmembrane protein 11, mitochondrial OS=Homo sapiens GN=TMEM11 PE=1 SV=1 - [TMM11_HUMAN]	22,92	4	0,780	0,678	[illegible]
Q96HR9	Receptor expression-enhancing protein 6 OS=Homo sapiens GN=REEP6 PE=1 SV=1 - [REEP6_HUMAN]	22,83	4	0,505	0,671	[illegible]
O14548	Cytochrome c oxidase subunit 7A-related protein, mitochondrial OS=Homo sapiens GN=COX7A2L PE=1 SV=2 - [COX7R_HUMAN]	22,81	4	0,761	0,738	[illegible]
P50479	PDZ and LIM domain protein 4 OS=Homo sapiens GN=PDLIM4 PE=1 SV=2 - [PDLI4_HUMAN]	22,73	4	1,693		[illegible]
P51970	NADH dehydrogenase [ubiquinone] 1 alpha subcomplex subunit 8 OS=Homo sapiens GN=NDUFA8 PE=1 SV=3 - [NDUA8_HUMAN]	22,67	4	1,262	1,310	[illegible]
P40616	ADP-ribosylation factor-like protein 1 OS=Homo sapiens GN=ARL1 PE=1 SV=1 - [ARL1_HUMAN]	22,65	4	0,670	0,741	[illegible]
P02792	Ferritin light chain OS=Homo sapiens GN=FTL PE=1 SV=2 - [FRIL_HUMAN]	22,29	4	0,973	1,302	[illegible]
P37235	Hippocalcin-like protein 1 OS=Homo sapiens GN=HPCAL1 PE=1 SV=3 - [HPCL1_HUMAN]	22,28	4	0,667	0,818	[illegible]
Q9NX57	Ras-related protein Rab-20 OS=Homo sapiens GN=RAB20 PE=1 SV=1 - [RAB20_HUMAN]	22,22	4	1,135	1,218	[illegible]
Q9UNZ5	Leydig cell tumor 10 kDa protein homolog OS=Homo sapiens GN=C19orf53 PE=1 SV=1 - [L10K_HUMAN]	22,22	4			[illegible]
P98173	Protein FAM3A OS=Homo sapiens GN=FAM3A PE=1 SV=2 - [FAM3A_HUMAN]	22,17	4	0,793	0,626	[illegible]
Q01081	Splicing factor U2AF 35 kDa subunit OS=Homo sapiens GN=U2AF1 PE=1 SV=3 - [U2AF1_HUMAN]	22,08	4			[illegible]
Q9NX47	E3 ubiquitin-protein ligase MARCH5 OS=Homo sapiens GN=MARCH5 PE=1 SV=1 - [MARH5_HUMAN]	21,94	4	1,194	1,688	[illegible]
Q9HBL7	Plasminogen receptor (KT) OS=Homo sapiens GN=C9orf46 PE=1 SV=1 - [PLRKT_HUMAN]	21,77	4	0,744	0,789	[illegible]
O14579	Coatomer subunit epsilon OS=Homo sapiens GN=COPE PE=1 SV=3 - [COPE_HUMAN]	21,75	4	0,482	0,837	[illegible]
P61009	Signal peptidase complex subunit 3 OS=Homo sapiens GN=SPCS3 PE=1 SV=1 - [SPCS3_HUMAN]	21,67	4	0,971	0,848	[illegible]
P21926	CD9 antigen OS=Homo sapiens GN=CD9 PE=1 SV=4 - [CD9_HUMAN]	21,49	4	0,949	1,208	[illegible]
Q9Y3A4	Ribosomal RNA-processing protein 7 homolog A OS=Homo sapiens GN=RRP7A PE=1 SV=2 - [RRP7A_HUMAN]	21,43	4	0,796	0,906	[illegible]
Q96G21	U3 small nucleolar ribonucleoprotein protein IMP4 OS=Homo sapiens GN=IMP4 PE=1 SV=1 - [IMP4_HUMAN]	21,31	4	0,861		[illegible]
P51159	Ras-related protein Rab-27A OS=Homo sapiens GN=RAB27A PE=1 SV=3 - [RB27A_HUMAN]	21,27	4	0,593	0,625	[illegible]
P36543	V-type proton ATPase subunit E 1 OS=Homo sapiens GN=ATP6V1E1 PE=1 SV=1 - [VATE1_HUMAN]	21,24	4	0,986	1,009	[illegible]
Q9BYD3	39S ribosomal protein L4, mitochondrial OS=Homo sapiens GN=MRPL4 PE=1 SV=1 - [RM04_HUMAN]	21,22	4	1,045	1,019	[illegible]
Q8N0U8	Vitamin K epoxide reductase complex subunit 1-like protein 1 OS=Homo sapiens GN=VKORC1L1 PE=1 SV=2 - [VKORL_HUMAN]	21,02	4	0,886	1,021	[illegible]
Q9Y3B3	Transmembrane emp24 domain-containing protein 7 OS=Homo sapiens GN=TMED7 PE=1 SV=2 - [TMED7_HUMAN]	20,98	4	0,736	0,653	[illegible]
Q9GZU8	Protein FAM192A OS=Homo sapiens GN=FAM192A PE=1 SV=1 - [F192A_HUMAN]	20,87	4			[illegible]
Q09028	Histone-binding protein RBBP4 OS=Homo sapiens GN=RBBP4 PE=1 SV=3 - [RBBP4_HUMAN]	20,71	4	0,728		[illegible]
P25789	Proteasome subunit alpha type-4 OS=Homo sapiens GN=PSMA4 PE=1 SV=1 - [PSA4_HUMAN]	20,69	4	0,989	1,156	[illegible]
Q92506	Estradiol 17-beta-dehydrogenase 8 OS=Homo sapiens GN=HSD17B8 PE=1 SV=2 - [DHB8_HUMAN]	20,69	4	0,457	0,549	[illegible]
P43897	Elongation factor Ts, mitochondrial OS=Homo sapiens GN=TSFM PE=1 SV=2 - [EFTS_HUMAN]	20,62	4	0,860	0,993	[illegible]
P49069	Calcium signal-modulating cyclophilin ligand OS=Homo sapiens GN=CAMLG PE=1 SV=1 - [CAMLG_HUMAN]	20,61	4	1,169	1,227	[illegible]
Q9Y619	Mitochondrial ornithine transporter 1 OS=Homo sapiens GN=SLC25A15 PE=1 SV=1 - [ORNT1_HUMAN]	20,60	4	0,842	1,013	[illegible]
P37108	Signal recognition particle 14 kDa protein OS=Homo sapiens GN=SRP14 PE=1 SV=2 - [SRP14_HUMAN]	20,59	4	1,052	0,913	[illegible]
O95721	Synaptosomal-associated protein 29 OS=Homo sapiens GN=SNAP29 PE=1 SV=1 - [SNP29_HUMAN]	20,54	4	1,148	1,126	[illegible]
O00399	Dynactin subunit 6 OS=Homo sapiens GN=DCTN6 PE=1 SV=1 - [DCTN6_HUMAN]	20,53	4			[illegible]
O15258	Protein RER1 OS=Homo sapiens GN=RER1 PE=1 SV=1 - [RER1_HUMAN]	20,41	4	0,853	0,876	[illegible]
Q9UBP6	tRNA (guanine-N(7)-)-methyltransferase OS=Homo sapiens GN=METTL1 PE=1 SV=1 - [TRMB_HUMAN]	20,29	4			[illegible]
Q14257	Reticulocalbin-2 OS=Homo sapiens GN=RCN2 PE=1 SV=1 - [RCN2_HUMAN]	20,19	4	0,958		[illegible]
O95433	Activator of 90 kDa heat shock protein ATPase homolog 1 OS=Homo sapiens GN=AHSA1 PE=1 SV=1 - [AHSA1_HUMAN]	19,82	4	0,828	0,769	[illegible]
Q6PI78	Transmembrane protein 65 OS=Homo sapiens GN=TMEM65 PE=1 SV=2 - [TMM65_HUMAN]	19,58	4	1,743	1,363	[illegible]
P78330	Phosphoserine phosphatase OS=Homo sapiens GN=PSPH PE=1 SV=2 - [SERB_HUMAN]	19,56	4			[illegible]
Q01130	Serine/arginine-rich splicing factor 2 OS=Homo sapiens GN=SRSF2 PE=1 SV=4 - [SRSF2_HUMAN]	19,46	4			[illegible]
Q03405	Urokinase plasminogen activator surface receptor OS=Homo sapiens GN=PLAUR PE=1 SV=1 - [UPAR_HUMAN]	19,40	4	0,655	0,468	[illegible]
Q9NY12	H/ACA ribonucleoprotein complex subunit 1 OS=Homo sapiens GN=GAR1 PE=1 SV=1 - [GAR1_HUMAN]	19,35	4		1,323	[illegible]
P09525	Annexin A4 OS=Homo sapiens GN=ANXA4 PE=1 SV=4 - [ANXA4_HUMAN]	19,12	4	0,709	0,791	[illegible]
Q16576	Histone-binding protein RBBP7 OS=Homo sapiens GN=RBBP7 PE=1 SV=1 - [RBBP7_HUMAN]	19,06	4		1,060	[illegible]
O95772	MLN64 N-terminal domain homolog OS=Homo sapiens GN=STARD3NL PE=1 SV=1 - [MENTO_HUMAN]	18,80	4	1,413	1,175	[illegible]
P16152	Carbonyl reductase [NADPH] 1 OS=Homo sapiens GN=CBR1 PE=1 SV=3 - [CBR1_HUMAN]	18,77	4	1,018	0,778	[illegible]
O14657	Torsin-1B OS=Homo sapiens GN=TOR1B PE=1 SV=2 - [TOR1B_HUMAN]	18,75	4	1,283	1,245	[illegible]
Q53H82	Beta-lactamase-like protein 2 OS=Homo sapiens GN=LACTB2 PE=1 SV=2 - [LACB2_HUMAN]	18,75	4	0,677	1,056	[illegible]
Q9H2D1	Mitochondrial folate transporter/carrier OS=Homo sapiens GN=SLC25A32 PE=1 SV=2 - [MFTC_HUMAN]	18,73	4	1,251	1,190	[illegible]
O95297	Myelin protein zero-like protein 1 OS=Homo sapiens GN=MPZL1 PE=1 SV=1 - [MPZL1_HUMAN]	18,59	4	1,924	1,729	[illegible]
O14561	Acyl carrier protein, mitochondrial OS=Homo sapiens GN=NDUFAB1 PE=1 SV=3 - [ACPM_HUMAN]	18,59	4	1,014	0,873	[illegible]
P49795	Regulator of G-protein signaling 19 OS=Homo sapiens GN=RGS19 PE=1 SV=1 - [RGS19_HUMAN]	18,43	4	2,008	1,930	[illegible]
Q86T03	Transmembrane protein 55B OS=Homo sapiens GN=TMEM55B PE=1 SV=1 - [TM55B_HUMAN]	18,41	4	1,042	1,019	[illegible]
P02647	Apolipoprotein A-I OS=Homo sapiens GN=APOA1 PE=1 SV=1 - [APOA1_HUMAN]	18,35	4	1,041	1,189	[illegible]
Q9H4I3	TraB domain-containing protein OS=Homo sapiens GN=TRABD PE=2 SV=1 - [TRABD_HUMAN]	18,35	4	1,321	0,877	[illegible]
P29084	Transcription initiation factor IIE subunit beta OS=Homo sapiens GN=GTF2E2 PE=1 SV=1 - [T2EB_HUMAN]	18,21	4	0,787		[illegible]
Q9Y696	Chloride intracellular channel protein 4 OS=Homo sapiens GN=CLIC4 PE=1 SV=4 - [CLIC4_HUMAN]	18,18	4	1,089		[illegible]
Q6PK18	PKHD domain-containing transmembrane protein C17orf101 OS=Homo sapiens GN=C17orf101 PE=2 SV=2 - [CQ101_HUMAN]	18,18	4	1,810	1,343	[illegible]
Q9NZ43	Vesicle transport protein USE1 OS=Homo sapiens GN=USE1 PE=1 SV=2 - [USE1_HUMAN]	18,15	4	1,110	1,211	[illegible]
P25788	Proteasome subunit alpha type-3 OS=Homo sapiens GN=PSMA3 PE=1 SV=2 - [PSA3_HUMAN]	18,04	4	0,926	0,897	[illegible]
Q12792	Twinfilin-1 OS=Homo sapiens GN=TWF1 PE=1 SV=3 - [TWF1_HUMAN]	18,00	4	0,865	0,704	[illegible]
Q86WC4	Osteopetrosis-associated transmembrane protein 1 OS=Homo sapiens GN=OSTM1 PE=1 SV=1 - [OSTM1_HUMAN]	17,96	4	2,068	1,881	[illegible]
Q8TCZ2	CD99 antigen-like protein 2 OS=Homo sapiens GN=CD99L2 PE=2 SV=1 - [C99L2_HUMAN]	17,94	4	1,728	2,007	[illegible]
P14678	Small nuclear ribonucleoprotein-associated proteins B and B' OS=Homo sapiens GN=SNRPB PE=1 SV=2 - [RSMB_HUMAN]	17,92	4	1,028	0,860	[illegible]
Q96BJ3	Axin interactor, dorsalization-associated protein OS=Homo sapiens GN=AIDA PE=1 SV=1 - [AIDA_HUMAN]	17,65	4	1,340	1,404	[illegible]
Q96IZ0	PRKC apoptosis WT1 regulator protein OS=Homo sapiens GN=PAWR PE=1 SV=1 - [PAWR_HUMAN]	17,65	4	1,246	1,305	[illegible]
Q8N9N7	Leucine-rich repeat-containing protein 57 OS=Homo sapiens GN=LRRC57 PE=1 SV=1 - [LRC57_HUMAN]	17,57	4	0,742	0,919	[illegible]
Q9P287	BRCA2 and CDKN1A-interacting protein OS=Homo sapiens GN=BCCIP PE=1 SV=1 - [BCCIP_HUMAN]	17,52	4	1,053		[illegible]
P41227	N-alpha-acetyltransferase 10 OS=Homo sapiens GN=NAA10 PE=1 SV=1 - [NAA10_HUMAN]	17,45	4		1,236	[illegible]
O00401	Neural Wiskott-Aldrich syndrome protein OS=Homo sapiens GN=WASL PE=1 SV=2 - [WASL_HUMAN]	17,43	4	1,007	0,941	[illegible]
Q9Y5Y5	Peroxisomal membrane protein PEX16 OS=Homo sapiens GN=PEX16 PE=1 SV=2 - [PEX16_HUMAN]	17,26	4	0,856	0,777	[illegible]
O95861	3'(2'),5'-bisphosphate nucleotidase 1 OS=Homo sapiens GN=BPNT1 PE=1 SV=1 - [BPNT1_HUMAN]	17,21	4	0,624	0,625	[illegible]
P62826	GTP-binding nuclear protein Ran OS=Homo sapiens GN=RAN PE=1 SV=3 - [RAN_HUMAN]	17,13	4	0,810	0,980	[illegible]
Q13868	Exosome complex component RRP4 OS=Homo sapiens GN=EXOSC2 PE=1 SV=2 - [EXOS2_HUMAN]	17,06	4			[illegible]
P78417	Glutathione S-transferase omega-1 OS=Homo sapiens GN=GSTO1 PE=1 SV=2 - [GSTO1_HUMAN]	17,01	4	1,185	1,070	[illegible]
Q8NBQ5	Estradiol 17-beta-dehydrogenase 11 OS=Homo sapiens GN=HSD17B11 PE=1 SV=3 - [DHB11_HUMAN]	17,00	4	0,904	1,014	[illegible]
O14818	Proteasome subunit alpha type-7 OS=Homo sapiens GN=PSMA7 PE=1 SV=1 - [PSA7_HUMAN]	16,94	4	1,248	1,095	[illegible]
Q96FZ7	Charged multivesicular body protein 6 OS=Homo sapiens GN=CHMP6 PE=1 SV=3 - [CHMP6_HUMAN]	16,92	4	1,296	1,232	[illegible]
P14550	Alcohol dehydrogenase [NADP(+)] OS=Homo sapiens GN=AKR1A1 PE=1 SV=3 - [AK1A1_HUMAN]	16,92	4			[illegible]
Q96RD7	Pannexin-1 OS=Homo sapiens GN=PANX1 PE=1 SV=4 - [PANX1_HUMAN]	16,90	4	1,151	1,252	[illegible]
Q8ND76	Cyclin-Y OS=Homo sapiens GN=CCNY PE=1 SV=2 - [CCNY_HUMAN]	16,72	4	1,111	0,977	[illegible]
P15514	Amphiregulin OS=Homo sapiens GN=AREG PE=1 SV=2 - [AREG_HUMAN]	16,67	4	1,123	0,829	[illegible]
Q96B26	Exosome complex component RRP43 OS=Homo sapiens GN=EXOSC8 PE=1 SV=1 - [EXOS8_HUMAN]	16,67	4			[illegible]
O00273	DNA fragmentation factor subunit alpha OS=Homo sapiens GN=DFFA PE=1 SV=1 - [DFFA_HUMAN]	16,62	4	0,836		[illegible]
P30533	Alpha-2-macroglobulin receptor-associated protein OS=Homo sapiens GN=LRPAP1 PE=1 SV=1 - [AMRP_HUMAN]	16,53	4	0,954		[illegible]
Q12893	Transmembrane protein 115 OS=Homo sapiens GN=TMEM115 PE=1 SV=1 - [TM115_HUMAN]	16,52	4	1,447	1,267	[illegible]
Q8NFV4	Abhydrolase domain-containing protein 11 OS=Homo sapiens GN=ABHD11 PE=1 SV=1 - [ABHDB_HUMAN]	16,51	4	0,452	0,345	[illegible]
Q5W0Z9	Probable palmitoyltransferase ZDHHC20 OS=Homo sapiens GN=ZDHHC20 PE=1 SV=1 - [ZDH20_HUMAN]	16,44	4	0,904	1,166	[illegible]
Q9HD33	39S ribosomal protein L47, mitochondrial OS=Homo sapiens GN=MRPL47 PE=1 SV=2 - [RM47_HUMAN]	16,40	4	1,138	1,218	[illegible]
O43808	Peroxisomal membrane protein PMP34 OS=Homo sapiens GN=SLC25A17 PE=1 SV=1 - [PM34_HUMAN]	16,29	4	0,801	0,797	[illegible]
Q9H813	Transmembrane protein 206 OS=Homo sapiens GN=TMEM206 PE=1 SV=1 - [TM206_HUMAN]	16,29	4	1,552	1,528	[illegible]
Q99439	Calponin-2 OS=Homo sapiens GN=CNN2 PE=1 SV=4 - [CNN2_HUMAN]	16,18	4	0,859	0,745	[illegible]
O95674	Phosphatidate cytidylyltransferase 2 OS=Homo sapiens GN=CDS2 PE=1 SV=1 - [CDS2_HUMAN]	16,18	4	1,311	1,330	[illegible]
Q9NR45	Sialic acid synthase OS=Homo sapiens GN=NANS PE=1 SV=2 - [SIAS_HUMAN]	16,16	4			[illegible]
O95159	Zinc finger protein-like 1 OS=Homo sapiens GN=ZFPL1 PE=1 SV=2 - [ZFPL1_HUMAN]	16,13	4	1,438	1,844	[illegible]
P55209	Nucleosome assembly protein 1-like 1 OS=Homo sapiens GN=NAP1L1 PE=1 SV=1 - [NP1L1_HUMAN]	16,11	4	0,996	1,060	[illegible]
P12429	Annexin A3 OS=Homo sapiens GN=ANXA3 PE=1 SV=3 - [ANXA3_HUMAN]	16,10	4	0,681	0,675	[illegible]
Q8N1S5	Zinc transporter ZIP11 OS=Homo sapiens GN=SLC39A11 PE=2 SV=3 - [S39AB_HUMAN]	16,08	4	0,764	0,968	[illegible]
Q6FI81	Anamorsin OS=Homo sapiens GN=CIAPIN1 PE=1 SV=2 - [CPIN1_HUMAN]	16,03	4			[illegible]
Q9UN37	Vacuolar protein sorting-associated protein 4A OS=Homo sapiens GN=VPS4A PE=1 SV=1 - [VPS4A_HUMAN]	16,02	4		0,799	[illegible]
Q8NC42	E3 ubiquitin-protein ligase RNF149 OS=Homo sapiens GN=RNF149 PE=2 SV=2 - [RN149_HUMAN]	16,00	4	0,834	0,894	[illegible]
O00303	Eukaryotic translation initiation factor 3 subunit F OS=Homo sapiens GN=EIF3F PE=1 SV=1 - [EIF3F_HUMAN]	15,97	4	0,697	0,869	[illegible]
Q9BUN8	Derlin-1 OS=Homo sapiens GN=DERL1 PE=1 SV=1 - [DERL1_HUMAN]	15,94	4	0,903	0,933	[illegible]
P10451	Osteopontin OS=Homo sapiens GN=SPP1 PE=1 SV=1 - [OSTP_HUMAN]	15,92	4	**2,062**	**2,523**	[illegible]
P29218	Inositol monophosphatase 1 OS=Homo sapiens GN=IMPA1 PE=1 SV=1 - [IMPA1_HUMAN]	15,88	4	1,342	1,127	[illegible]
Q14192	Four and a half LIM domains protein 2 OS=Homo sapiens GN=FHL2 PE=1 SV=3 - [FHL2_HUMAN]	15,77	4			[illegible]
Q9BYN8	28S ribosomal protein S26, mitochondrial OS=Homo sapiens GN=MRPS26 PE=1 SV=1 - [RT26_HUMAN]	15,61	4	0,931	1,086	[illegible]
Q03113	Guanine nucleotide-binding protein subunit alpha-12 OS=Homo sapiens GN=GNA12 PE=1 SV=4 - [GNA12_HUMAN]	15,49	4	0,893	1,022	[illegible]
Q96K37	Solute carrier family 35 member E1 OS=Homo sapiens GN=SLC35E1 PE=1 SV=2 - [S35E1_HUMAN]	15,37	4	1,070	1,108	[illegible]
Q9UHD1	Cysteine and histidine-rich domain-containing protein 1 OS=Homo sapiens GN=CHORDC1 PE=1 SV=2 - [CHRD1_HUMAN]	15,36	4	0,691	1,010	[illegible]
O95858	Tetraspanin-15 OS=Homo sapiens GN=TSPAN15 PE=2 SV=1 - [TSN15_HUMAN]	15,31	4	1,026	1,113	[illegible]
Q16637	Survival motor neuron protein OS=Homo sapiens GN=SMN1 PE=1 SV=1 - [SMN_HUMAN]	15,31	4		0,813	[illegible]
P13647	Keratin, type II cytoskeletal 5 OS=Homo sapiens GN=KRT5 PE=1 SV=3 - [K2C5_HUMAN]	15,25	4	1,175	2,075	[illegible]
P35250	Replication factor C subunit 2 OS=Homo sapiens GN=RFC2 PE=1 SV=3 - [RFC2_HUMAN]	15,25	4	0,873		[illegible]
Q8NBM4	Ubiquitin-associated domain-containing protein 2 OS=Homo sapiens GN=UBAC2 PE=2 SV=1 - [UBAC2_HUMAN]	15,12	4	0,988	1,012	[illegible]
O15160	DNA-directed RNA polymerases I and III subunit RPAC1 OS=Homo sapiens GN=POLR1C PE=1 SV=1 - [RPAC1_HUMAN]	15,03	4	0,730		[illegible]
Q9NP79	Vacuolar protein sorting-associated protein VTA1 homolog OS=Homo sapiens GN=VTA1 PE=1 SV=1 - [VTA1_HUMAN]	14,98	4	0,806		[illegible]
Q6P6C2	Probable alpha-ketoglutarate-dependent dioxygenase ABH5 OS=Homo sapiens GN=ALKBH5 PE=1 SV=2 - [ALKB5_HUMAN]	14,97	4			[illegible]
Q10567	AP-1 complex subunit beta-1 OS=Homo sapiens GN=AP1B1 PE=1 SV=2 - [AP1B1_HUMAN]	14,96	4	0,706	0,956	[illegible]
Q9H553	Alpha-1,3/1,6-mannosyltransferase ALG2 OS=Homo sapiens GN=ALG2 PE=2 SV=1 - [ALG2_HUMAN]	14,90	4	0,765	0,766	[illegible]
P46976	Glycogenin-1 OS=Homo sapiens GN=GYG1 PE=1 SV=4 - [GLYG_HUMAN]	14,86	4	1,432	1,525	[illegible]
Q6ZMG9	Ceramide synthase 6 OS=Homo sapiens GN=CERS6 PE=1 SV=1 - [CERS6_HUMAN]	14,84	4	0,568	0,702	[illegible]
Q9Y6E2	Basic leucine zipper and W2 domain-containing protein 2 OS=Homo sapiens GN=BZW2 PE=1 SV=1 - [BZW2_HUMAN]	14,80	4	0,764	0,973	[illegible]
Q8TBP6	Solute carrier family 25 member 40 OS=Homo sapiens GN=SLC25A40 PE=2 SV=1 - [S2540_HUMAN]	14,79	4	1,435	1,361	[illegible]
Q9HCU5	Prolactin regulatory element-binding protein OS=Homo sapiens GN=PREB PE=1 SV=2 - [PREB_HUMAN]	14,63	4	0,847	0,944	[illegible]
Q9Y5Q0	Fatty acid desaturase 3 OS=Homo sapiens GN=FADS3 PE=2 SV=1 - [FADS3_HUMAN]	14,61	4	1,978	1,651	[illegible]
Q96NC0	Zinc finger matrin-type protein 2 OS=Homo sapiens GN=ZMAT2 PE=1 SV=1 - [ZMAT2_HUMAN]	14,57	4			[illegible]
Q13445	Transmembrane emp24 domain-containing protein 1 OS=Homo sapiens GN=TMED1 PE=1 SV=1 - [TMED1_HUMAN]	14,54	4	0,942	0,866	[illegible]
Q643R3	Lysophospholipid acyltransferase LPCAT4 OS=Homo sapiens GN=LPCAT4 PE=1 SV=1 - [LPCT4_HUMAN]	14,50	4	0,803	0,852	[illegible]
Q9BXW7	Cat eye syndrome critical region protein 5 OS=Homo sapiens GN=CECR5 PE=1 SV=1 - [CECR5_HUMAN]	14,42	4		1,050	[illegible]
O00629	Importin subunit alpha-4 OS=Homo sapiens GN=KPNA4 PE=1 SV=1 - [IMA4_HUMAN]	14,40	4	0,758	0,818	[illegible]
Q9ULA0	Aspartyl aminopeptidase OS=Homo sapiens GN=DNPEP PE=1 SV=1 - [DNPEP_HUMAN]	14,32	4	0,702		[illegible]
O43657	Tetraspanin-6 OS=Homo sapiens GN=TSPAN6 PE=1 SV=1 - [TSN6_HUMAN]	14,29	4	0,643	0,613	[illegible]
P35914	Hydroxymethylglutaryl-CoA lyase, mitochondrial OS=Homo sapiens GN=HMGCL PE=1 SV=2 - [HMGCL_HUMAN]	14,15	4	0,626	0,909	[illegible]
Q6ZT21	Transmembrane protein with metallophosphoesterase domain OS=Homo sapiens GN=TMPPE PE=2 SV=2 - [TMPPE_HUMAN]	14,13	4	1,290	1,373	[illegible]
P26196	Probable ATP-dependent RNA helicase DDX6 OS=Homo sapiens GN=DDX6 PE=1 SV=2 - [DDX6_HUMAN]	14,08	4	0,940	0,906	[illegible]
Q7KYR7	Butyrophilin subfamily 2 member A1 OS=Homo sapiens GN=BTN2A1 PE=1 SV=3 - [BT2A1_HUMAN]	14,04	4	1,369	1,285	[illegible]
Q13045	Protein flightless-1 homolog OS=Homo sapiens GN=FLII PE=1 SV=2 - [FLII_HUMAN]	14,03	4	0,871	1,012	[illegible]
P11117	Lysosomal acid phosphatase OS=Homo sapiens GN=ACP2 PE=1 SV=3 - [PPAL_HUMAN]	13,95	4	1,400	1,288	[illegible]
O43765	Small glutamine-rich tetratricopeptide repeat-containing protein alpha OS=Homo sapiens GN=SGTA PE=1 SV=1 - [SGTA_HUMAN]	13,74	4	0,928		[illegible]
O75487	Glypican-4 OS=Homo sapiens GN=GPC4 PE=1 SV=4 - [GPC4_HUMAN]	13,67	4	1,935	1,796	[illegible]
Q9BXR0	Queuine tRNA-ribosyltransferase OS=Homo sapiens GN=QTRT1 PE=1 SV=3 - [TGT_HUMAN]	13,65	4			[illegible]
Q96A49	Synapse-associated protein 1 OS=Homo sapiens GN=SYAP1 PE=1 SV=1 - [SYAP1_HUMAN]	13,64	4	1,307		[illegible]
Q08209	Serine/threonine-protein phosphatase 2B catalytic subunit alpha isoform OS=Homo sapiens GN=PPP3CA PE=1 SV=1 - [PP2BA_HUMAN]	13,63	4	1,046	0,872	[illegible]
Q9H0S4	Probable ATP-dependent RNA helicase DDX47 OS=Homo sapiens GN=DDX47 PE=1 SV=1 - [DDX47_HUMAN]	13,63	4	0,885		[illegible]
O60684	Importin subunit alpha-7 OS=Homo sapiens GN=KPNA6 PE=1 SV=1 - [IMA7_HUMAN]	13,62	4	0,939		[illegible]
P61163	Alpha-centractin OS=Homo sapiens GN=ACTR1A PE=1 SV=1 - [ACTZ_HUMAN]	13,56	4	0,843		[illegible]
P40937	Replication factor C subunit 5 OS=Homo sapiens GN=RFC5 PE=1 SV=1 - [RFC5_HUMAN]	13,53	4	0,472	0,435	[illegible]
Q8WZ73	E3 ubiquitin-protein ligase rififylin OS=Homo sapiens GN=RFFL PE=1 SV=1 - [RFFL_HUMAN]	13,50	4	0,902	1,144	[illegible]
Q8TBM8	DnaJ homolog subfamily B member 14 OS=Homo sapiens GN=DNAJB14 PE=2 SV=1 - [DJB14_HUMAN]	13,46	4	1,392	1,107	[illegible]
Q969V5	Mitochondrial ubiquitin ligase activator of NFKB 1 OS=Homo sapiens GN=MUL1 PE=1 SV=1 - [MUL1_HUMAN]	13,35	4	1,173	1,295	[illegible]
Q9BTZ2	Dehydrogenase/reductase SDR family member 4 OS=Homo sapiens GN=DHRS4 PE=1 SV=3 - [DHRS4_HUMAN]	13,31	4		0,851	[illegible]
Q8WWY3	U4/U6 small nuclear ribonucleoprotein Prp31 OS=Homo sapiens GN=PRPF31 PE=1 SV=2 - [PRP31_HUMAN]	13,23	4	0,688	0,804	[illegible]
Q9P289	Serine/threonine-protein kinase MST4 OS=Homo sapiens GN=MST4 PE=1 SV=2 - [MST4_HUMAN]	13,22	4	0,771	0,948	[illegible]

Q15904	V-type proton ATPase subunit S1 OS=Homo sapiens GN=ATP6AP1 PE=1 SV=2 - [VAS1_HUMAN]	13,19
Q96GK7	Fumarylacetoacetate hydrolase domain-containing protein 2A OS=Homo sapiens GN=FAHD2A PE=1 SV=1 - [FAH2A_HUMAN]	13,06
P82673	28S ribosomal protein S35, mitochondrial OS=Homo sapiens GN=MRPS35 PE=1 SV=1 - [RT35_HUMAN]	13,00
Q8NBN7	Retinol dehydrogenase 13 OS=Homo sapiens GN=RDH13 PE=1 SV=2 - [RDH13_HUMAN]	12,99
P54578	Ubiquitin carboxyl-terminal hydrolase 14 OS=Homo sapiens GN=USP14 PE=1 SV=3 - [UBP14_HUMAN]	12,96
Q96GY0	Protein FAM164A OS=Homo sapiens GN=FAM164A PE=1 SV=2 - [F164A_HUMAN]	12,92
O15143	Actin-related protein 2/3 complex subunit 1B OS=Homo sapiens GN=ARPC1B PE=1 SV=3 - [ARC1B_HUMAN]	12,90
Q9H3P7	Golgi resident protein GCP60 OS=Homo sapiens GN=ACBD3 PE=1 SV=4 - [GCP60_HUMAN]	12,88
P46926	Glucosamine-6-phosphate isomerase 1 OS=Homo sapiens GN=GNPDA1 PE=1 SV=1 - [GNPI1_HUMAN]	12,80
Q9BXS5	AP-1 complex subunit mu-1 OS=Homo sapiens GN=AP1M1 PE=1 SV=3 - [AP1M1_HUMAN]	12,77
Q5VT66	MOSC domain-containing protein 1, mitochondrial OS=Homo sapiens GN=MARC1 PE=1 SV=1 - [MOSC1_HUMAN]	12,76
Q3LXA3	Bifunctional ATP-dependent dihydroxyacetone kinase/FAD-AMP lyase (cyclizing) OS=Homo sapiens GN=DAK PE=1 SV=2 - [DHAK_HUMA	12,70
Q8NFH4	Nucleoporin Nup37 OS=Homo sapiens GN=NUP37 PE=1 SV=1 - [NUP37_HUMAN]	12,58
Q8N6T3	ADP-ribosylation factor GTPase-activating protein 1 OS=Homo sapiens GN=ARFGAP1 PE=1 SV=2 - [ARFG1_HUMAN]	12,56
Q9BZE9	Tether containing UBX domain for GLUT4 OS=Homo sapiens GN=ASPSCR1 PE=1 SV=1 - [ASPC1_HUMAN]	12,48
Q9NZC3	Glycerophosphodiester phosphodiesterase 1 OS=Homo sapiens GN=GDE1 PE=1 SV=1 - [GDE1_HUMAN]	12,39
Q9Y2Z4	Tyrosine--tRNA ligase, mitochondrial OS=Homo sapiens GN=YARS2 PE=1 SV=2 - [SYYM_HUMAN]	12,37
P36551	Coproporphyrinogen-III oxidase, mitochondrial OS=Homo sapiens GN=CPOX PE=1 SV=3 - [HEM6_HUMAN]	12,33
Q13148	TAR DNA-binding protein 43 OS=Homo sapiens GN=TARDBP PE=1 SV=1 - [TADBP_HUMAN]	12,32
Q9BVG9	Phosphatidylserine synthase 2 OS=Homo sapiens GN=PTDSS2 PE=1 SV=1 - [PTSS2_HUMAN]	12,32
Q9BZI7	Regulator of nonsense transcripts 3B OS=Homo sapiens GN=UPF3B PE=1 SV=1 - [REN3B_HUMAN]	12,22
P51648	Fatty aldehyde dehydrogenase OS=Homo sapiens GN=ALDH3A2 PE=1 SV=1 - [AL3A2_HUMAN]	12,16
Q969S3	Zinc finger protein 622 OS=Homo sapiens GN=ZNF622 PE=1 SV=1 - [ZN622_HUMAN]	12,16
P53990	IST1 homolog OS=Homo sapiens GN=IST1 PE=1 SV=1 - [IST1_HUMAN]	12,09
Q9UMY1	Nucleolar protein 7 OS=Homo sapiens GN=NOL7 PE=1 SV=2 - [NOL7_HUMAN]	12,06
P07093	Glia-derived nexin OS=Homo sapiens GN=SERPINE2 PE=1 SV=1 - [GDN_HUMAN]	12,06
Q96D42	Hepatitis A virus cellular receptor 1 OS=Homo sapiens GN=HAVCR1 PE=1 SV=2 - [HAVR1_HUMAN]	11,98
Q5T280	Uncharacterized protein C9orf114 OS=Homo sapiens GN=C9orf114 PE=1 SV=3 - [CI114_HUMAN]	11,97
Q9NV64	Transmembrane protein 39A OS=Homo sapiens GN=TMEM39A PE=2 SV=1 - [TM39A_HUMAN]	11,89
Q6P4E1	Protein CASC4 OS=Homo sapiens GN=CASC4 PE=2 SV=1 - [CASC4_HUMAN]	11,78
Q8WU90	Zinc finger CCCH domain-containing protein 15 OS=Homo sapiens GN=ZC3H15 PE=1 SV=1 - [ZC3HF_HUMAN]	11,74
O60512	Beta-1,4-galactosyltransferase 3 OS=Homo sapiens GN=B4GALT3 PE=1 SV=2 - [B4GT3_HUMAN]	11,70
Q9H9P8	L-2-hydroxyglutarate dehydrogenase, mitochondrial OS=Homo sapiens GN=L2HGDH PE=1 SV=3 - [L2HDH_HUMAN]	11,66
O94766	Galactosylgalactosylxylosylprotein 3-beta-glucuronosyltransferase 3 OS=Homo sapiens GN=B3GAT3 PE=1 SV=2 - [B3GA3_HUMAN]	11,64
P56589	Peroxisomal biogenesis factor 3 OS=Homo sapiens GN=PEX3 PE=1 SV=1 - [PEX3_HUMAN]	11,53
Q9UNF0	Protein kinase C and casein kinase substrate in neurons protein 2 OS=Homo sapiens GN=PACSIN2 PE=1 SV=2 - [PACN2_HUMAN]	11,32
P18615	Negative elongation factor E OS=Homo sapiens GN=RDBP PE=1 SV=3 - [NELFE_HUMAN]	11,32
P35052	Glypican-1 OS=Homo sapiens GN=GPC1 PE=1 SV=2 - [GPC1_HUMAN]	11,29
O60291	E3 ubiquitin-protein ligase MGRN1 OS=Homo sapiens GN=MGRN1 PE=1 SV=2 - [MGRN1_HUMAN]	11,23
O60669	Monocarboxylate transporter 2 OS=Homo sapiens GN=SLC16A7 PE=2 SV=2 - [MOT2_HUMAN]	11,09
P10619	Lysosomal protective protein OS=Homo sapiens GN=CTSA PE=1 SV=2 - [PPGB_HUMAN]	11,04
Q9H490	Phosphatidylinositol glycan anchor biosynthesis class U protein OS=Homo sapiens GN=PIGU PE=1 SV=3 - [PIGU_HUMAN]	11,03
Q9Y606	tRNA pseudouridine synthase A, mitochondrial OS=Homo sapiens GN=PUS1 PE=1 SV=3 - [TRUA_HUMAN]	11,01
Q9NPQ8	Synembryn-A OS=Homo sapiens GN=RIC8A PE=1 SV=3 - [RIC8A_HUMAN]	10,92
Q5K4L6	Long-chain fatty acid transport protein 3 OS=Homo sapiens GN=SLC27A3 PE=2 SV=3 - [S27A3_HUMAN]	10,82
Q8N357	Transmembrane protein C2orf18 OS=Homo sapiens GN=C2orf18 PE=1 SV=1 - [CB018_HUMAN]	10,78
Q9UG56	Phosphatidylserine decarboxylase proenzyme OS=Homo sapiens GN=PISD PE=2 SV=4 - [PISD_HUMAN]	10,76
Q14498	RNA-binding protein 39 OS=Homo sapiens GN=RBM39 PE=1 SV=2 - [RBM39_HUMAN]	10,75
Q9HC36	RNA methyltransferase-like protein 1 OS=Homo sapiens GN=RNMTL1 PE=1 SV=2 - [RMTL1_HUMAN]	10,71
P00966	Argininosuccinate synthase OS=Homo sapiens GN=ASS1 PE=1 SV=2 - [ASSY_HUMAN]	10,68
O75880	Protein SCO1 homolog, mitochondrial OS=Homo sapiens GN=SCO1 PE=1 SV=1 - [SCO1_HUMAN]	10,63
Q8TCT8	Signal peptide peptidase-like 2A OS=Homo sapiens GN=SPPL2A PE=1 SV=2 - [PSL2_HUMAN]	10,58
Q96KP4	Cytosolic non-specific dipeptidase OS=Homo sapiens GN=CNDP2 PE=1 SV=2 - [CNDP2_HUMAN]	10,53
P51570	Galactokinase OS=Homo sapiens GN=GALK1 PE=1 SV=1 - [GALK1_HUMAN]	10,46
O75312	Zinc finger protein ZPR1 OS=Homo sapiens GN=ZNF259 PE=1 SV=1 - [ZPR1_HUMAN]	10,46
Q969P0	Immunoglobulin superfamily member 8 OS=Homo sapiens GN=IGSF8 PE=1 SV=1 - [IGSF8_HUMAN]	10,44
Q9UNF1	Melanoma-associated antigen D2 OS=Homo sapiens GN=MAGED2 PE=1 SV=2 - [MAGD2_HUMAN]	10,40
Q96L92	Sorting nexin-27 OS=Homo sapiens GN=SNX27 PE=1 SV=2 - [SNX27_HUMAN]	10,35
O00232	26S proteasome non-ATPase regulatory subunit 12 OS=Homo sapiens GN=PSMD12 PE=1 SV=3 - [PSD12_HUMAN]	10,31
Q9H300	Presenilins-associated rhomboid-like protein, mitochondrial OS=Homo sapiens GN=PARL PE=1 SV=2 - [PARL_HUMAN]	10,29
Q9H1E3	Nuclear ubiquitous casein and cyclin-dependent kinases substrate OS=Homo sapiens GN=NUCKS1 PE=1 SV=1 - [NUCKS_HUMAN]	10,29
P51690	Arylsulfatase E OS=Homo sapiens GN=ARSE PE=1 SV=2 - [ARSE_HUMAN]	10,19
Q9H0U3	Magnesium transporter protein 1 OS=Homo sapiens GN=MAGT1 PE=1 SV=1 - [MAGT1_HUMAN]	10,15
Q6WCQ1	Myosin phosphatase Rho-interacting protein OS=Homo sapiens GN=MPRIP PE=1 SV=3 - [MPRIP_HUMAN]	10,15
P51649	Succinate-semialdehyde dehydrogenase, mitochondrial OS=Homo sapiens GN=ALDH5A1 PE=1 SV=2 - [SSDH_HUMAN]	10,09
O75153	Protein KIAA0664 OS=Homo sapiens GN=KIAA0664 PE=1 SV=2 - [K0664_HUMAN]	10,08
Q04206	Transcription factor p65 OS=Homo sapiens GN=RELA PE=1 SV=2 - [TF65_HUMAN]	9,98
Q9BXS6	Nucleolar and spindle-associated protein 1 OS=Homo sapiens GN=NUSAP1 PE=1 SV=1 - [NUSAP_HUMAN]	9,98
Q9H9A6	Leucine-rich repeat-containing protein 40 OS=Homo sapiens GN=LRRC40 PE=1 SV=1 - [LRC40_HUMAN]	9,97
Q2TAA5	GDP-Man:Man(3)GlcNAc(2)-PP-Dol alpha-1,2-mannosyltransferase OS=Homo sapiens GN=ALG11 PE=1 SV=2 - [ALG11_HUMAN]	9,96
P23368	NAD-dependent malic enzyme, mitochondrial OS=Homo sapiens GN=ME2 PE=1 SV=1 - [MAOM_HUMAN]	9,93
Q00013	55 kDa erythrocyte membrane protein OS=Homo sapiens GN=MPP1 PE=1 SV=2 - [EM55_HUMAN]	9,87
Q7Z4H8	KDEL motif-containing protein 2 OS=Homo sapiens GN=KDELC2 PE=1 SV=2 - [KDEL2_HUMAN]	9,86
Q6P4Q7	Metal transporter CNNM4 OS=Homo sapiens GN=CNNM4 PE=1 SV=3 - [CNNM4_HUMAN]	9,81
P0CG12	Chromosome transmission fidelity protein 8 homolog isoform 2 OS=Homo sapiens GN=CHTF8 PE=2 SV=1 - [CTF8A_HUMAN]	9,73
O60427	Fatty acid desaturase 1 OS=Homo sapiens GN=FADS1 PE=1 SV=1 - [FADS1_HUMAN]	9,68
Q6EMK4	Vasorin OS=Homo sapiens GN=VASN PE=1 SV=1 - [VASN_HUMAN]	9,66
Q9H6U8	Alpha-1,2-mannosyltransferase ALG9 OS=Homo sapiens GN=ALG9 PE=1 SV=2 - [ALG9_HUMAN]	9,66
Q6ZWT7	Lysophospholipid acyltransferase 2 OS=Homo sapiens GN=MBOAT2 PE=2 SV=2 - [MBOA2_HUMAN]	9,42
Q8TAD4	Zinc transporter 5 OS=Homo sapiens GN=SLC30A5 PE=1 SV=1 - [ZNT5_HUMAN]	9,41
P05107	Integrin beta-2 OS=Homo sapiens GN=ITGB2 PE=1 SV=2 - [ITB2_HUMAN]	9,36
Q8NEF9	Serum response factor-binding protein 1 OS=Homo sapiens GN=SRFBP1 PE=1 SV=1 - [SRFB1_HUMAN]	9,32
O75063	Glycosaminoglycan xylosylkinase OS=Homo sapiens GN=FAM20B PE=1 SV=1 - [XYLK_HUMAN]	9,29
P06865	Beta-hexosaminidase subunit alpha OS=Homo sapiens GN=HEXA PE=1 SV=2 - [HEXA_HUMAN]	9,26
Q15008	26S proteasome non-ATPase regulatory subunit 6 OS=Homo sapiens GN=PSMD6 PE=1 SV=1 - [PSMD6_HUMAN]	9,25
Q8NDZ6	Transmembrane protein 161B OS=Homo sapiens GN=TMEM161B PE=2 SV=1 - [T161B_HUMAN]	9,24
P31641	Sodium- and chloride-dependent taurine transporter OS=Homo sapiens GN=SLC6A6 PE=1 SV=2 - [SC6A6_HUMAN]	9,19
Q4ZIN3	Membralin OS=Homo sapiens GN=C19orf6 PE=1 SV=1 - [MBRL_HUMAN]	9,19
Q9BZZ5	Apoptosis inhibitor 5 OS=Homo sapiens GN=API5 PE=1 SV=3 - [API5_HUMAN]	8,97
Q9UK23	N-acetylglucosamine-1-phosphodiester alpha-N-acetylglucosaminidase OS=Homo sapiens GN=NAGPA PE=2 SV=2 - [NAGPA_HUMAN]	8,93
Q9UQB8	Brain-specific angiogenesis inhibitor 1-associated protein 2 OS=Homo sapiens GN=BAIAP2 PE=1 SV=1 - [BAIP2_HUMAN]	8,88
Q9H0X4	Protein ITFG3 OS=Homo sapiens GN=ITFG3 PE=1 SV=1 - [ITFG3_HUMAN]	8,88
Q8N697	Solute carrier family 15 member 4 OS=Homo sapiens GN=SLC15A4 PE=2 SV=1 - [S15A4_HUMAN]	8,84
Q86U44	N6-adenosine-methyltransferase 70 kDa subunit OS=Homo sapiens GN=METTL3 PE=1 SV=2 - [MTA70_HUMAN]	8,79
Q8N442	Translation factor GUF1, mitochondrial OS=Homo sapiens GN=GUF1 PE=1 SV=1 - [GUF1_HUMAN]	8,67
Q53GS9	U4/U6.U5 tri-snRNP-associated protein 2 OS=Homo sapiens GN=USP39 PE=1 SV=2 - [SNUT2_HUMAN]	8,67
O14524	Transmembrane protein 194A OS=Homo sapiens GN=TMEM194A PE=1 SV=2 - [T194A_HUMAN]	8,56
Q3KQU3	MAP7 domain-containing protein 1 OS=Homo sapiens GN=MAP7D1 PE=1 SV=1 - [MA7D1_HUMAN]	8,56
Q9UJV9	Probable ATP-dependent RNA helicase DDX41 OS=Homo sapiens GN=DDX41 PE=1 SV=2 - [DDX41_HUMAN]	8,52
Q96IG2	F-box/LRR-repeat protein 20 OS=Homo sapiens GN=FBXL20 PE=1 SV=2 - [FXL20_HUMAN]	8,49
Q8N9T8	Protein KRI1 homolog OS=Homo sapiens GN=KRI1 PE=1 SV=2 - [KRI1_HUMAN]	8,46
Q7L9B9	Endonuclease/exonuclease/phosphatase family domain-containing protein 1 OS=Homo sapiens GN=EEPD1 PE=1 SV=2 - [EEPD1_HUMAN]	8,44
Q86TM6	E3 ubiquitin-protein ligase synoviolin OS=Homo sapiens GN=SYVN1 PE=1 SV=2 - [SYVN1_HUMAN]	8,43
Q99797	Mitochondrial intermediate peptidase OS=Homo sapiens GN=MIPEP PE=1 SV=2 - [MIPEP_HUMAN]	8,42
Q9H3G5	Probable serine carboxypeptidase CPVL OS=Homo sapiens GN=CPVL PE=1 SV=2 - [CPVL_HUMAN]	8,40
Q99755	Phosphatidylinositol-4-phosphate 5-kinase type-1 alpha OS=Homo sapiens GN=PIP5K1A PE=1 SV=1 - [PI51A_HUMAN]	8,36
Q15397	Pumilio domain-containing protein KIAA0020 OS=Homo sapiens GN=KIAA0020 PE=1 SV=3 - [K0020_HUMAN]	8,33
Q14166	Tubulin--tyrosine ligase-like protein 12 OS=Homo sapiens GN=TTLL12 PE=1 SV=2 - [TTL12_HUMAN]	8,07
P48147	Prolyl endopeptidase OS=Homo sapiens GN=PREP PE=1 SV=2 - [PPCE_HUMAN]	8,03
Q9Y289	Sodium-dependent multivitamin transporter OS=Homo sapiens GN=SLC5A6 PE=2 SV=2 - [SC5A6_HUMAN]	8,03
P22455	Fibroblast growth factor receptor 4 OS=Homo sapiens GN=FGFR4 PE=1 SV=2 - [FGFR4_HUMAN]	7,98
Q12874	Splicing factor 3A subunit 3 OS=Homo sapiens GN=SF3A3 PE=1 SV=1 - [SF3A3_HUMAN]	7,98
Q9BX10	GTP-binding protein 2 OS=Homo sapiens GN=GTPBP2 PE=2 SV=1 - [GTPB2_HUMAN]	7,97
Q96D46	60S ribosomal export protein NMD3 OS=Homo sapiens GN=NMD3 PE=1 SV=1 - [NMD3_HUMAN]	7,95
P18433	Receptor-type tyrosine-protein phosphatase alpha OS=Homo sapiens GN=PTPRA PE=1 SV=2 - [PTPRA_HUMAN]	7,86
Q13330	Metastasis-associated protein MTA1 OS=Homo sapiens GN=MTA1 PE=1 SV=2 - [MTA1_HUMAN]	7,83
P17252	Protein kinase C alpha type OS=Homo sapiens GN=PRKCA PE=1 SV=4 - [KPCA_HUMAN]	7,74
Q9NVV4	Poly(A) RNA polymerase, mitochondrial OS=Homo sapiens GN=MTPAP PE=1 SV=1 - [PAPD1_HUMAN]	7,73
Q12849	G-rich sequence factor 1 OS=Homo sapiens GN=GRSF1 PE=1 SV=3 - [GRSF1_HUMAN]	7,71
Q9H8H0	Nucleolar protein 11 OS=Homo sapiens GN=NOL11 PE=1 SV=1 - [NOL11_HUMAN]	7,65
Q8NHP8	Putative phospholipase B-like 2 OS=Homo sapiens GN=PLBD2 PE=1 SV=2 - [PLBL2_HUMAN]	7,64
Q9Y450	HBS1-like protein OS=Homo sapiens GN=HBS1L PE=1 SV=1 - [HBS1L_HUMAN]	7,60
P52888	Thimet oligopeptidase OS=Homo sapiens GN=THOP1 PE=1 SV=2 - [THOP1_HUMAN]	7,55
P00734	Prothrombin OS=Homo sapiens GN=F2 PE=1 SV=2 - [THRB_HUMAN]	7,40
Q15061	WD repeat-containing protein 43 OS=Homo sapiens GN=WDR43 PE=1 SV=3 - [WDR43_HUMAN]	7,39
P49848	Transcription initiation factor TFIID subunit 6 OS=Homo sapiens GN=TAF6 PE=1 SV=1 - [TAF6_HUMAN]	7,39
Q9H3H5	UDP-N-acetylglucosamine--dolichyl-phosphate N-acetylglucosaminephosphotransferase OS=Homo sapiens GN=DPAGT1 PE=1 SV=2 - [GP	7,35
Q9NQU5	Serine/threonine-protein kinase PAK 6 OS=Homo sapiens GN=PAK6 PE=1 SV=1 - [PAK6_HUMAN]	7,34
Q6PJW8	Consortin OS=Homo sapiens GN=CNST PE=1 SV=3 - [CNST_HUMAN]	7,31
P11362	Fibroblast growth factor receptor 1 OS=Homo sapiens GN=FGFR1 PE=1 SV=3 - [FGFR1_HUMAN]	7,30
Q9Y5W7	Sorting nexin-14 OS=Homo sapiens GN=SNX14 PE=1 SV=3 - [SNX14_HUMAN]	7,29
P04062	Glucosylceramidase OS=Homo sapiens GN=GBA PE=1 SV=3 - [GLCM_HUMAN]	7,28
Q9BYT8	Neurolysin, mitochondrial OS=Homo sapiens GN=NLN PE=1 SV=1 - [NEUL_HUMAN]	7,24
Q8IZ83	Aldehyde dehydrogenase family 16 member A1 OS=Homo sapiens GN=ALDH16A1 PE=1 SV=2 - [A16A1_HUMAN]	7,23
P22059	Oxysterol-binding protein 1 OS=Homo sapiens GN=OSBP PE=1 SV=1 - [OSBP1_HUMAN]	7,19
P49590	Probable histidine--tRNA ligase, mitochondrial OS=Homo sapiens GN=HARS2 PE=1 SV=1 - [SYHM_HUMAN]	7,11
Q9NQS3	Poliovirus receptor-related protein 3 OS=Homo sapiens GN=PVRL3 PE=1 SV=1 - [PVRL3_HUMAN]	7,10
Q8N5G2	Macoilin OS=Homo sapiens GN=TMEM57 PE=1 SV=1 - [MACOI_HUMAN]	7,08
Q9UNN5	FAS-associated factor 1 OS=Homo sapiens GN=FAF1 PE=1 SV=2 - [FAF1_HUMAN]	7,08
Q70J99	Protein unc-13 homolog D OS=Homo sapiens GN=UNC13D PE=1 SV=1 - [UN13D_HUMAN]	7,06
Q8N370	Large neutral amino acids transporter small subunit 4 OS=Homo sapiens GN=SLC43A2 PE=1 SV=1 - [LAT4_HUMAN]	7,03
Q14694	Ubiquitin carboxyl-terminal hydrolase 10 OS=Homo sapiens GN=USP10 PE=1 SV=2 - [UBP10_HUMAN]	7,02
O14639	Actin-binding LIM protein 1 OS=Homo sapiens GN=ABLIM1 PE=1 SV=3 - [ABLM1_HUMAN]	6,94
Q6UXD5	Seizure 6-like protein 2 OS=Homo sapiens GN=SEZ6L2 PE=1 SV=2 - [SE6L2_HUMAN]	6,92
Q96JM3	Chromosome alignment-maintaining phosphoprotein 1 OS=Homo sapiens GN=CHAMP1 PE=1 SV=2 - [CHAP1_HUMAN]	6,90
Q9Y6X5	Ectonucleotide pyrophosphatase/phosphodiesterase family member 4 OS=Homo sapiens GN=ENPP4 PE=1 SV=3 - [ENPP4_HUMAN]	6,84
P04150	Glucocorticoid receptor OS=Homo sapiens GN=NR3C1 PE=1 SV=1 - [GCR_HUMAN]	6,82
Q6P1M3	Lethal(2) giant larvae protein homolog 2 OS=Homo sapiens GN=LLGL2 PE=1 SV=2 - [L2GL2_HUMAN]	6,76
P05165	Propionyl-CoA carboxylase alpha chain, mitochondrial OS=Homo sapiens GN=PCCA PE=1 SV=4 - [PCCA_HUMAN]	6,73
Q6P1N0	Coiled-coil and C2 domain-containing protein 1A OS=Homo sapiens GN=CC2D1A PE=1 SV=1 - [C2D1A_HUMAN]	6,73
O14964	Hepatocyte growth factor-regulated tyrosine kinase substrate OS=Homo sapiens GN=HGS PE=1 SV=1 - [HGS_HUMAN]	6,69
O95171	Sciellin OS=Homo sapiens GN=SCEL PE=1 SV=2 - [SCEL_HUMAN]	6,69
Q96EY7	Pentatricopeptide repeat-containing protein 3, mitochondrial OS=Homo sapiens GN=PTCD3 PE=1 SV=3 - [PTCD3_HUMAN]	6,68
O43592	Exportin-T OS=Homo sapiens GN=XPOT PE=1 SV=2 - [XPOT_HUMAN]	6,65
Q99567	Nuclear pore complex protein Nup88 OS=Homo sapiens GN=NUP88 PE=1 SV=2 - [NUP88_HUMAN]	6,61
O60645	Exocyst complex component 3 OS=Homo sapiens GN=EXOC3 PE=1 SV=2 - [EXOC3_HUMAN]	6,61
Q6PJG2	Uncharacterized protein C14orf43 OS=Homo sapiens GN=C14orf43 PE=1 SV=2 - [CN043_HUMAN]	6,60
Q12788	Transducin beta-like protein 3 OS=Homo sapiens GN=TBL3 PE=1 SV=2 - [TBL3_HUMAN]	6,44
P25440	Bromodomain-containing protein 2 OS=Homo sapiens GN=BRD2 PE=1 SV=2 - [BRD2_HUMAN]	6,37
Q96J02	E3 ubiquitin-protein ligase Itchy homolog OS=Homo sapiens GN=ITCH PE=1 SV=2 - [ITCH_HUMAN]	6,20
Q96DT7	Zinc finger and BTB domain-containing protein 10 OS=Homo sapiens GN=ZBTB10 PE=1 SV=2 - [ZBT10_HUMAN]	6,20
P48449	Lanosterol synthase OS=Homo sapiens GN=LSS PE=1 SV=1 - [ERG7_HUMAN]	6,15
O00411	DNA-directed RNA polymerase, mitochondrial OS=Homo sapiens GN=POLRMT PE=1 SV=2 - [RPOM_HUMAN]	6,10
O60502	Bifunctional protein NCOAT OS=Homo sapiens GN=MGEA5 PE=1 SV=2 - [NCOAT_HUMAN]	6,00
Q5SY16	Polynucleotide 5'-hydroxyl-kinase NOL9 OS=Homo sapiens GN=NOL9 PE=1 SV=1 - [NOL9_HUMAN]	5,98
Q70EL4	Ubiquitin carboxyl-terminal hydrolase 43 OS=Homo sapiens GN=USP43 PE=1 SV=2 - [UBP43_HUMAN]	5,97
Q14554	Protein disulfide-isomerase A5 OS=Homo sapiens GN=PDIA5 PE=1 SV=1 - [PDIA5_HUMAN]	5,97
O60763	General vesicular transport factor p115 OS=Homo sapiens GN=USO1 PE=1 SV=2 - [USO1_HUMAN]	5,93
Q8IUD2	ELKS/Rab6-interacting/CAST family member 1 OS=Homo sapiens GN=ERC1 PE=1 SV=1 - [RB6I2_HUMAN]	5,91
Q86UK7	Zinc finger protein 598 OS=Homo sapiens GN=ZNF598 PE=1 SV=1 - [ZN598_HUMAN]	5,86

Q6Y7W6	PERQ amino acid-rich with GYF domain-containing protein 2 OS=Homo sapiens GN=GIGYF2 PE=1 SV=1 - [PERQ2_HUMAN]	5,85	4		
Q8IVL5	Prolyl 3-hydroxylase 2 OS=Homo sapiens GN=LEPREL1 PE=1 SV=1 - [P3H2_HUMAN]	5,79	4	**2,097**	**2,615**
Q8TAQ2	SWI/SNF complex subunit SMARCC2 OS=Homo sapiens GN=SMARCC2 PE=1 SV=1 - [SMRC2_HUMAN]	5,77	4	0,742	0,657
Q5C9Z4	Nucleolar MIF4G domain-containing protein 1 OS=Homo sapiens GN=NOM1 PE=1 SV=1 - [NOM1_HUMAN]	5,70	4	0,988	0,867
Q9NQ11	Probable cation-transporting ATPase 13A2 OS=Homo sapiens GN=ATP13A2 PE=1 SV=2 - [AT132_HUMAN]	5,68	4	0,929	0,854
Q9BPX6	Calcium uptake protein 1, mitochondrial OS=Homo sapiens GN=MICU1 PE=1 SV=1 - [MICU1_HUMAN]	5,67	4	1,535	1,022
Q709F0	Acyl-CoA dehydrogenase family member 11 OS=Homo sapiens GN=ACAD11 PE=1 SV=2 - [ACD11_HUMAN]	5,64	4	1,393	1,464
Q9Y3T9	Nucleolar complex protein 2 homolog OS=Homo sapiens GN=NOC2L PE=1 SV=4 - [NOC2L_HUMAN]	5,61	4	0,985	0,885
Q96KM6	Zinc finger protein 512B OS=Homo sapiens GN=ZNF512B PE=1 SV=1 - [Z512B_HUMAN]	5,61	4		
Q9UL01	Dermatan-sulfate epimerase OS=Homo sapiens GN=DSE PE=1 SV=1 - [DSE_HUMAN]	5,53	4	1,610	1,251
Q99575	Ribonucleases P/MRP protein subunit POP1 OS=Homo sapiens GN=POP1 PE=1 SV=2 - [POP1_HUMAN]	5,47	4	0,511	
Q96T76	MMS19 nucleotide excision repair protein homolog OS=Homo sapiens GN=MMS19 PE=1 SV=2 - [MMS19_HUMAN]	5,44	4	0,838	1,103
Q9NQX3	Gephyrin OS=Homo sapiens GN=GPHN PE=1 SV=1 - [GEPH_HUMAN]	5,43	4		0,528
Q14596	Next to BRCA1 gene 1 protein OS=Homo sapiens GN=NBR1 PE=1 SV=3 - [NBR1_HUMAN]	5,28	4	1,419	1,210
Q9C0C4	Semaphorin-4C OS=Homo sapiens GN=SEMA4C PE=1 SV=2 - [SEM4C_HUMAN]	5,28	4	1,048	1,009
Q93009	Ubiquitin carboxyl-terminal hydrolase 7 OS=Homo sapiens GN=USP7 PE=1 SV=2 - [UBP7_HUMAN]	5,26	4		1,119
Q2M389	WASH complex subunit 7 OS=Homo sapiens GN=KIAA1033 PE=1 SV=2 - [WASH7_HUMAN]	5,20	4	0,864	0,962
P13798	Acylamino-acid-releasing enzyme OS=Homo sapiens GN=APEH PE=1 SV=4 - [ACPH_HUMAN]	5,19	4		0,708
Q7Z417	Nuclear fragile X mental retardation-interacting protein 2 OS=Homo sapiens GN=NUFIP2 PE=1 SV=1 - [NUFP2_HUMAN]	5,18	4		
Q9H501	ESF1 homolog OS=Homo sapiens GN=ESF1 PE=1 SV=1 - [ESF1_HUMAN]	5,17	4		
Q7Z3C6	Autophagy-related protein 9A OS=Homo sapiens GN=ATG9A PE=1 SV=3 - [ATG9A_HUMAN]	5,13	4	1,037	
Q86V48	Leucine zipper protein 1 OS=Homo sapiens GN=LUZP1 PE=1 SV=2 - [LUZP1_HUMAN]	5,11	4		
Q69YQ0	Cytospin-A OS=Homo sapiens GN=SPECC1L PE=1 SV=2 - [CYTSA_HUMAN]	5,10	4		
Q5T3F8	Transmembrane protein 63B OS=Homo sapiens GN=TMEM63B PE=1 SV=1 - [TM63B_HUMAN]	5,05	4	1,816	1,621
Q96GQ7	Probable ATP-dependent RNA helicase DDX27 OS=Homo sapiens GN=DDX27 PE=1 SV=2 - [DDX27_HUMAN]	5,03	4		
Q13111	Chromatin assembly factor 1 subunit A OS=Homo sapiens GN=CHAF1A PE=1 SV=2 - [CAF1A_HUMAN]	4,92	4		
Q9Y2K7	Lysine-specific demethylase 2A OS=Homo sapiens GN=KDM2A PE=1 SV=3 - [KDM2A_HUMAN]	4,91	4	0,571	0,820
Q9P2I0	Cleavage and polyadenylation specificity factor subunit 2 OS=Homo sapiens GN=CPSF2 PE=1 SV=2 - [CPSF2_HUMAN]	4,86	4	0,759	0,853
O00754	Lysosomal alpha-mannosidase OS=Homo sapiens GN=MAN2B1 PE=1 SV=3 - [MA2B1_HUMAN]	4,85	4	0,830	0,837
P57678	Gem-associated protein 4 OS=Homo sapiens GN=GEMIN4 PE=1 SV=2 - [GEMI4_HUMAN]	4,82	4	0,654	0,766
Q5VTR2	E3 ubiquitin-protein ligase BRE1A OS=Homo sapiens GN=RNF20 PE=1 SV=2 - [BRE1A_HUMAN]	4,82	4		
P23921	Ribonucleoside-diphosphate reductase large subunit OS=Homo sapiens GN=RRM1 PE=1 SV=1 - [RIR1_HUMAN]	4,80	4	1,399	0,674
Q9ULU4-6	Isoform 6 of Protein kinase C-binding protein 1 OS=Homo sapiens GN=ZMYND8 - [PKCB1_HUMAN]	4,79	4		
Q96JQ2	Calmin OS=Homo sapiens GN=CLMN PE=1 SV=1 - [CLMN_HUMAN]	4,69	4	0,854	0,846
Q12872	Splicing factor, suppressor of white-apricot homolog OS=Homo sapiens GN=SFSWAP PE=1 SV=3 - [SFSWA_HUMAN]	4,63	4		
Q8TEQ8	GPI ethanolamine phosphate transferase 3 OS=Homo sapiens GN=PIGO PE=2 SV=3 - [PIGO_HUMAN]	4,59	4	1,203	0,593
Q8NBJ5	Procollagen galactosyltransferase 1 OS=Homo sapiens GN=GLT25D1 PE=1 SV=1 - [GT251_HUMAN]	4,34	4	1,112	
Q96ST3	Paired amphipathic helix protein Sin3a OS=Homo sapiens GN=SIN3A PE=1 SV=2 - [SIN3A_HUMAN]	4,32	4		
O75110	Probable phospholipid-transporting ATPase IIA OS=Homo sapiens GN=ATP9A PE=1 SV=3 - [ATP9A_HUMAN]	4,30	4	1,050	0,891
Q96AE7	Tetratricopeptide repeat protein 17 OS=Homo sapiens GN=TTC17 PE=1 SV=1 - [TTC17_HUMAN]	4,29	4	1,125	1,069
Q66K74	Microtubule-associated protein 1S OS=Homo sapiens GN=MAP1S PE=1 SV=2 - [MAP1S_HUMAN]	4,25	4	0,841	
Q92974	Rho guanine nucleotide exchange factor 2 OS=Homo sapiens GN=ARHGEF2 PE=1 SV=4 - [ARHG2_HUMAN]	4,16	4		0,912
P46199	Translation initiation factor IF-2, mitochondrial OS=Homo sapiens GN=MTIF2 PE=1 SV=2 - [IF2M_HUMAN]	4,13	4	1,078	0,952
Q9UKK3	Poly [ADP-ribose] polymerase 4 OS=Homo sapiens GN=PARP4 PE=1 SV=3 - [PARP4_HUMAN]	3,94	4		0,583
Q9H2U1	Probable ATP-dependent RNA helicase DHX36 OS=Homo sapiens GN=DHX36 PE=1 SV=2 - [DHX36_HUMAN]	3,87	4	0,735	0,880
Q12873	Chromodomain-helicase-DNA-binding protein 3 OS=Homo sapiens GN=CHD3 PE=1 SV=3 - [CHD3_HUMAN]	3,85	4	0,683	
Q92538	Golgi-specific brefeldin A-resistance guanine nucleotide exchange factor 1 OS=Homo sapiens GN=GBF1 PE=1 SV=2 - [GBF1_HUMAN]	3,71	4	1,048	
Q14692	Ribosome biogenesis protein BMS1 homolog OS=Homo sapiens GN=BMS1 PE=1 SV=1 - [BMS1_HUMAN]	3,67	4	0,587	
P36956	Sterol regulatory element-binding protein 1 OS=Homo sapiens GN=SREBF1 PE=1 SV=2 - [SRBP1_HUMAN]	3,66	4	0,687	0,772
Q8IWU2	Serine/threonine-protein kinase LMTK2 OS=Homo sapiens GN=LMTK2 PE=1 SV=2 - [LMTK2_HUMAN]	3,59	4	1,288	1,146
Q5SNT6	WASH complex subunit FAM21B OS=Homo sapiens GN=FAM21B PE=1 SV=2 - [FA21B_HUMAN]	3,59	4		
Q9ULL5-3	Isoform 3 of Proline-rich protein 12 OS=Homo sapiens GN=PRR12 - [PRR12_HUMAN]	3,44	4		
Q92620	Pre-mRNA-splicing factor ATP-dependent RNA helicase PRP16 OS=Homo sapiens GN=DHX38 PE=1 SV=2 - [PRP16_HUMAN]	3,34	4		0,723
O94979	Protein transport protein Sec31A OS=Homo sapiens GN=SEC31A PE=1 SV=3 - [SC31A_HUMAN]	3,28	4	1,172	1,225
P01023	Alpha-2-macroglobulin OS=Homo sapiens GN=A2M PE=1 SV=3 - [A2MG_HUMAN]	3,26	4	1,465	1,363
Q7RTP6	Protein-methionine sulfoxide oxidase MICAL3 OS=Homo sapiens GN=MICAL3 PE=1 SV=2 - [MICA3_HUMAN]	3,25	4		
O60292	Signal-induced proliferation-associated 1-like protein 3 OS=Homo sapiens GN=SIPA1L3 PE=1 SV=3 - [SI1L3_HUMAN]	3,20	4		
Q9NRL2	Bromodomain adjacent to zinc finger domain protein 1A OS=Homo sapiens GN=BAZ1A PE=1 SV=2 - [BAZ1A_HUMAN]	3,15	4	1,062	
P42356	Phosphatidylinositol 4-kinase alpha OS=Homo sapiens GN=PI4KA PE=1 SV=3 - [PI4KA_HUMAN]	3,08	4	0,797	0,649
Q8WYP5	Protein ELYS OS=Homo sapiens GN=AHCTF1 PE=1 SV=3 - [ELYS_HUMAN]	3,00	4		0,605
Q562E7	WD repeat-containing protein 81 OS=Homo sapiens GN=WDR81 PE=1 SV=2 - [WDR81_HUMAN]	2,99	4	1,324	
Q5SRE5	Nucleoporin NUP188 homolog OS=Homo sapiens GN=NUP188 PE=1 SV=1 - [NU188_HUMAN]	2,92	4	1,124	1,194
O43157	Plexin-B1 OS=Homo sapiens GN=PLXNB1 PE=1 SV=3 - [PLXB1_HUMAN]	2,90	4	1,363	1,204
Q15746	Myosin light chain kinase, smooth muscle OS=Homo sapiens GN=MYLK PE=1 SV=4 - [MYLK_HUMAN]	2,72	4		
Q9C0C9	Ubiquitin-conjugating enzyme E2 O OS=Homo sapiens GN=UBE2O PE=1 SV=3 - [UBE2O_HUMAN]	2,55	4		
Q92673	Sortilin-related receptor OS=Homo sapiens GN=SORL1 PE=1 SV=2 - [SORL_HUMAN]	2,30	4	0,628	0,632
O94915	Protein furry homolog-like OS=Homo sapiens GN=FRYL PE=1 SV=2 - [FRYL_HUMAN]	2,26	4	0,700	
Q9ULT8	E3 ubiquitin-protein ligase HECTD1 OS=Homo sapiens GN=HECTD1 PE=1 SV=3 - [HECD1_HUMAN]	2,26	4	0,546	0,936
P42858	Huntingtin OS=Homo sapiens GN=HTT PE=1 SV=2 - [HD_HUMAN]	2,23	4	0,663	0,763
Q9H4A3	Serine/threonine-protein kinase WNK1 OS=Homo sapiens GN=WNK1 PE=1 SV=2 - [WNK1_HUMAN]	1,72	4		0,951
P49454	Centromere protein F OS=Homo sapiens GN=CENPF PE=1 SV=2 - [CENPF_HUMAN]	1,68	4		
Q96T58	Msx2-interacting protein OS=Homo sapiens GN=SPEN PE=1 SV=1 - [MINT_HUMAN]	1,56	4		
Q96RL7	Vacuolar protein sorting-associated protein 13A OS=Homo sapiens GN=VPS13A PE=1 SV=2 - [VP13A_HUMAN]	1,54	4	1,232	1,006
Q2LD37	Uncharacterized protein KIAA1109 OS=Homo sapiens GN=KIAA1109 PE=1 SV=2 - [K1109_HUMAN]	1,02	4	1,635	1,222
C9JI06	Mitochondrial inner membrane protein OS=Homo sapiens GN=IMMT PE=4 SV=1 - [C9JI06_HUMAN]	64,64	3	1,369	1,316
P07437	Tubulin beta chain OS=Homo sapiens GN=TUBB PE=1 SV=2 - [TBB5_HUMAN]	63,74	3	0,587	
P68371	Tubulin beta-4B chain OS=Homo sapiens GN=TUBB4B PE=1 SV=1 - [TBB4B_HUMAN]	60,22	3	0,657	0,591
Q14061	Cytochrome c oxidase copper chaperone OS=Homo sapiens GN=COX17 PE=1 SV=2 - [COX17_HUMAN]	57,14	3	1,004	
P05386	60S acidic ribosomal protein P1 OS=Homo sapiens GN=RPLP1 PE=1 SV=1 - [RLA1_HUMAN]	57,02	3	0,733	1,303
Q9UBI6	Guanine nucleotide-binding protein G(I)/G(S)/G(O) subunit gamma-12 OS=Homo sapiens GN=GNG12 PE=1 SV=3 - [GBG12_HUMAN]	56,94	3	1,584	1,814
P12235	ADP/ATP translocase 1 OS=Homo sapiens GN=SLC25A4 PE=1 SV=4 - [ADT1_HUMAN]	49,66	3	1,277	1,254
P17096	High mobility group protein HMG-I/HMG-Y OS=Homo sapiens GN=HMGA1 PE=1 SV=3 - [HMGA1_HUMAN]	49,53	3	0,862	0,666
O95716	Ras-related protein Rab-3D OS=Homo sapiens GN=RAB3D PE=1 SV=1 - [RAB3D_HUMAN]	48,86	3	0,246	
J3KN67	Tropomyosin alpha-3 chain OS=Homo sapiens GN=TPM3 PE=3 SV=1 - [J3KN67_HUMAN]	48,77	3	0,745	
P62857	40S ribosomal protein S28 OS=Homo sapiens GN=RPS28 PE=1 SV=1 - [RS28_HUMAN]	46,38	3	1,380	0,744
Q9BWJ5	Splicing factor 3B subunit 5 OS=Homo sapiens GN=SF3B5 PE=1 SV=1 - [SF3B5_HUMAN]	45,35	3	0,912	
P62314	Small nuclear ribonucleoprotein Sm D1 OS=Homo sapiens GN=SNRPD1 PE=1 SV=1 - [SMD1_HUMAN]	43,70	3	0,894	1,163
P18085	ADP-ribosylation factor 4 OS=Homo sapiens GN=ARF4 PE=1 SV=3 - [ARF4_HUMAN]	41,67	3	0,735	0,851
O43169	Cytochrome b5 type B OS=Homo sapiens GN=CYB5B PE=1 SV=2 - [CYB5B_HUMAN]	40,41	3	0,838	0,938
P69849	Nodal modulator 3 OS=Homo sapiens GN=NOMO3 PE=2 SV=2 - [NOMO3_HUMAN]	40,18	3	1,022	0,987
Q15155	Nodal modulator 1 OS=Homo sapiens GN=NOMO1 PE=1 SV=5 - [NOMO1_HUMAN]	39,77	3	1,465	1,544
P05534	HLA class I histocompatibility antigen, A-24 alpha chain OS=Homo sapiens GN=HLA-A PE=1 SV=2 - [1A24_HUMAN]	39,73	3	0,905	
P63172	Dynein light chain Tctex-type 1 OS=Homo sapiens GN=DYNLT1 PE=1 SV=1 - [DYLT1_HUMAN]	38,94	3	0,721	
P61970	Nuclear transport factor 2 OS=Homo sapiens GN=NUTF2 PE=1 SV=1 - [NTF2_HUMAN]	38,58	3	1,128	1,044
P68032	Actin, alpha cardiac muscle 1 OS=Homo sapiens GN=ACTC1 PE=1 SV=1 - [ACTC_HUMAN]	38,20	3	0,936	0,958
P15954	Cytochrome c oxidase subunit 7C, mitochondrial OS=Homo sapiens GN=COX7C PE=1 SV=1 - [COX7C_HUMAN]	38,10	3	1,377	0,908
P60520	Gamma-aminobutyric acid receptor-associated protein-like 2 OS=Homo sapiens GN=GABARAPL2 PE=1 SV=1 - [GBRL2_HUMAN]	37,61	3	1,547	1,120
P60468	Protein transport protein Sec61 subunit beta OS=Homo sapiens GN=SEC61B PE=1 SV=2 - [SC61B_HUMAN]	37,50	3	1,327	1,283
Q9H3K6	BolA-like protein 2 OS=Homo sapiens GN=BOLA2 PE=1 SV=1 - [BOLA2_HUMAN]	37,21	3	0,713	
O75531	Barrier-to-autointegration factor OS=Homo sapiens GN=BANF1 PE=1 SV=1 - [BAF_HUMAN]	37,08	3	0,836	0,923
Q7Z422	UPF0485 protein C1orf144 OS=Homo sapiens GN=C1orf144 PE=1 SV=1 - [CA144_HUMAN]	36,18	3		
Q9H299	SH3 domain-binding glutamic acid-rich-like protein 3 OS=Homo sapiens GN=SH3BGRL3 PE=1 SV=1 - [SH3L3_HUMAN]	35,48	3	0,684	1,085
P10599	Thioredoxin OS=Homo sapiens GN=TXN PE=1 SV=3 - [THIO_HUMAN]	35,24	3	0,790	0,737
O95777	N-alpha-acetyltransferase 38, NatC auxiliary subunit OS=Homo sapiens GN=NAA38 PE=1 SV=3 - [NAA38_HUMAN]	34,38	3		
O75348	V-type proton ATPase subunit G 1 OS=Homo sapiens GN=ATP6V1G1 PE=1 SV=3 - [VATG1_HUMAN]	33,90	3	1,000	
P09669	Cytochrome c oxidase subunit 6C OS=Homo sapiens GN=COX6C PE=1 SV=2 - [COX6C_HUMAN]	33,33	3	0,847	0,973
Q9NX14	NADH dehydrogenase [ubiquinone] 1 beta subcomplex subunit 11, mitochondrial OS=Homo sapiens GN=NDUFB11 PE=1 SV=1 - [NDUBB_	33,33	3	1,527	1,560
H3BN98	Uncharacterized protein (Fragment) OS=Homo sapiens PE=4 SV=2 - [H3BN98_HUMAN]	32,49	3		
Q13113	PDZK1-interacting protein 1 OS=Homo sapiens GN=PDZK1IP1 PE=1 SV=1 - [PDZI1_HUMAN]	32,46	3	1,234	1,120
P62861	40S ribosomal protein S30 OS=Homo sapiens GN=FAU PE=1 SV=1 - [RS30_HUMAN]	32,20	3		0,796
P01034	Cystatin-C OS=Homo sapiens GN=CST3 PE=1 SV=1 - [CYTC_HUMAN]	30,14	3	0,791	0,816
Q9BQ61	Uncharacterized protein C19orf43 OS=Homo sapiens GN=C19orf43 PE=1 SV=1 - [CS043_HUMAN]	30,11	3	0,903	
Q9UHV9	Prefoldin subunit 2 OS=Homo sapiens GN=PFDN2 PE=1 SV=1 - [PFD2_HUMAN]	29,87	3		
Q9BRP8	Partner of Y14 and mago OS=Homo sapiens GN=WIBG PE=1 SV=1 - [WIBG_HUMAN]	29,41	3		0,884
Q96EX1	UPF0767 protein C1orf212 OS=Homo sapiens GN=C1orf212 PE=2 SV=3 - [CA212_HUMAN]	29,35	3	0,885	0,952
P60604	Ubiquitin-conjugating enzyme E2 G2 OS=Homo sapiens GN=UBE2G2 PE=1 SV=1 - [UB2G2_HUMAN]	29,09	3	0,932	0,819
Q13438	Protein OS-9 OS=Homo sapiens GN=OS9 PE=1 SV=1 - [OS9_HUMAN]	28,79	3	0,540	0,553
O43676	NADH dehydrogenase [ubiquinone] 1 beta subcomplex subunit 3 OS=Homo sapiens GN=NDUFB3 PE=1 SV=3 - [NDUB3_HUMAN]	28,57	3	1,218	1,238
O94925-3	Isoform 3 of Glutaminase kidney isoform, mitochondrial OS=Homo sapiens GN=GLS - [GLSK_HUMAN]	28,43	3	1,047	1,030
Q15843	NEDD8 OS=Homo sapiens GN=NEDD8 PE=1 SV=1 - [NEDD8_HUMAN]	28,40	3		0,847
P13987	CD59 glycoprotein OS=Homo sapiens GN=CD59 PE=1 SV=1 - [CD59_HUMAN]	28,13	3	1,477	1,749
O75347	Tubulin-specific chaperone A OS=Homo sapiens GN=TBCA PE=1 SV=3 - [TBCA_HUMAN]	27,78	3		
Q04837	Single-stranded DNA-binding protein, mitochondrial OS=Homo sapiens GN=SSBP1 PE=1 SV=1 - [SSBP_HUMAN]	27,70	3		1,021
Q9Y2R0	Coiled-coil domain-containing protein 56 OS=Homo sapiens GN=CCDC56 PE=1 SV=1 - [CCD56_HUMAN]	27,36	3	0,848	1,031
Q8N5N7	39S ribosomal protein L50, mitochondrial OS=Homo sapiens GN=MRPL50 PE=1 SV=2 - [RM50_HUMAN]	27,22	3	1,100	1,112
Q9H082	Ras-related protein Rab-33B OS=Homo sapiens GN=RAB33B PE=1 SV=1 - [RB33B_HUMAN]	27,07	3	1,049	0,993
P60866	40S ribosomal protein S20 OS=Homo sapiens GN=RPS20 PE=1 SV=1 - [RS20_HUMAN]	26,89	3	0,907	0,809
P06703	Protein S100-A6 OS=Homo sapiens GN=S100A6 PE=1 SV=1 - [S10A6_HUMAN]	26,67	3	0,798	0,707
P82932	28S ribosomal protein S6, mitochondrial OS=Homo sapiens GN=MRPS6 PE=1 SV=3 - [RT06_HUMAN]	26,40	3	1,609	0,912
P51151	Ras-related protein Rab-9A OS=Homo sapiens GN=RAB9A PE=1 SV=1 - [RAB9A_HUMAN]	25,87	3	2,133	1,603
O14613	Cdc42 effector protein 2 OS=Homo sapiens GN=CDC42EP2 PE=1 SV=1 - [BORG1_HUMAN]	25,71	3		
Q9UI09	NADH dehydrogenase [ubiquinone] 1 alpha subcomplex subunit 12 OS=Homo sapiens GN=NDUFA12 PE=1 SV=1 - [NDUAC_HUMAN]	25,52	3	0,833	0,965
P61018	Ras-related protein Rab-4B OS=Homo sapiens GN=RAB4B PE=1 SV=1 - [RAB4B_HUMAN]	25,35	3	0,971	1,828
O43324	Eukaryotic translation elongation factor 1 epsilon-1 OS=Homo sapiens GN=EEF1E1 PE=1 SV=1 - [MCA3_HUMAN]	25,29	3	0,865	0,654
Q9P0T7	Transmembrane protein 9 OS=Homo sapiens GN=TMEM9 PE=1 SV=1 - [TMEM9_HUMAN]	25,14	3	1,133	1,241
P82663	28S ribosomal protein S25, mitochondrial OS=Homo sapiens GN=MRPS25 PE=1 SV=1 - [RT25_HUMAN]	24,86	3	1,042	1,229
Q9Y3C6	Peptidyl-prolyl cis-trans isomerase-like 1 OS=Homo sapiens GN=PPIL1 PE=1 SV=1 - [PPIL1_HUMAN]	24,70	3		
P13747	HLA class I histocompatibility antigen, alpha chain E OS=Homo sapiens GN=HLA-E PE=1 SV=3 - [HLAE_HUMAN]	24,58	3	0,754	0,880
P62330	ADP-ribosylation factor 6 OS=Homo sapiens GN=ARF6 PE=1 SV=2 - [ARF6_HUMAN]	24,57	3	0,892	0,973
Q9BU61	NADH dehydrogenase [ubiquinone] 1 alpha subcomplex assembly factor 3 OS=Homo sapiens GN=NDUFAF3 PE=1 SV=1 - [NDUF3_HUMA	24,46	3	1,088	1,345
Q49B96	Cytochrome c oxidase assembly protein COX19 OS=Homo sapiens GN=COX19 PE=1 SV=1 - [COX19_HUMAN]	24,44	3		
O75964	ATP synthase subunit g, mitochondrial OS=Homo sapiens GN=ATP5L PE=1 SV=3 - [ATP5L_HUMAN]	24,27	3	1,421	1,317
P07919	Cytochrome b-c1 complex subunit 6, mitochondrial OS=Homo sapiens GN=UQCRH PE=1 SV=2 - [QCR6_HUMAN]	24,18	3	0,813	1,015
Q9Y3C5	RING finger protein 11 OS=Homo sapiens GN=RNF11 PE=1 SV=1 - [RNF11_HUMAN]	24,03	3	1,211	
P13637	Sodium/potassium-transporting ATPase subunit alpha-3 OS=Homo sapiens GN=ATP1A3 PE=1 SV=3 - [AT1A3_HUMAN]	23,99	3	1,390	1,216
Q16527	Cysteine and glycine-rich protein 2 OS=Homo sapiens GN=CSRP2 PE=1 SV=3 - [CSRP2_HUMAN]	23,83	3	1,664	
Q8IZQ5	Selenoprotein H OS=Homo sapiens GN=SELH PE=1 SV=2 - [SELH_HUMAN]	23,77	3		
P61088	Ubiquitin-conjugating enzyme E2 N OS=Homo sapiens GN=UBE2N PE=1 SV=1 - [UBE2N_HUMAN]	23,68	3	0,672	0,683
Q7RTV0	PHD finger-like domain-containing protein 5A OS=Homo sapiens GN=PHF5A PE=1 SV=1 - [PHF5A_HUMAN]	23,64	3	0,970	0,847
P50502	Hsc70-interacting protein OS=Homo sapiens GN=ST13 PE=1 SV=2 - [F10A1_HUMAN]	23,04	3		0,826
O14925	Mitochondrial import inner membrane translocase subunit Tim23 OS=Homo sapiens GN=TIMM23 PE=1 SV=1 - [TIM23_HUMAN]	22,97	3	1,414	1,382
Q9P0M9	39S ribosomal protein L27, mitochondrial OS=Homo sapiens GN=MRPL27 PE=1 SV=1 - [RM27_HUMAN]	22,97	3	0,639	0,769
Q16763	Ubiquitin-conjugating enzyme E2 S OS=Homo sapiens GN=UBE2S PE=1 SV=2 - [UBE2S_HUMAN]	22,97	3		1,212
P49720	Proteasome subunit beta type-3 OS=Homo sapiens GN=PSMB3 PE=1 SV=2 - [PSB3_HUMAN]	22,93	3	0,793	0,818
Q5RI15	Protein FAM36A OS=Homo sapiens GN=FAM36A PE=1 SV=2 - [FA36A_HUMAN]	22,88	3	1,492	1,293
Q9NZZ3	Charged multivesicular body protein 5 OS=Homo sapiens GN=CHMP5 PE=1 SV=1 - [CHMP5_HUMAN]	22,83	3		1,045
P07203	Glutathione peroxidase 1 OS=Homo sapiens GN=GPX1 PE=1 SV=4 - [GPX1_HUMAN]	22,66	3	1,324	1,085
Q5J8M3	Transmembrane protein 85 OS=Homo sapiens GN=TMEM85 PE=1 SV=2 - [TMM85_HUMAN]	22,40	3	0,907	1,085
Q07812	Apoptosis regulator BAX OS=Homo sapiens GN=BAX PE=1 SV=1 - [BAX_HUMAN]	22,40	3	0,856	1,121
O95994	Anterior gradient protein 2 homolog OS=Homo sapiens GN=AGR2 PE=1 SV=1 - [AGR2_HUMAN]	22,29	3	0,014	0,166
Q8TAD7	Overexpressed in colon carcinoma 1 protein OS=Homo sapiens GN=OCC1 PE=1 SV=2 - [OCC1_HUMAN]	22,22	3	**2,267**	**2,149**
P30405	Peptidyl-prolyl cis-trans isomerase F, mitochondrial OS=Homo sapiens GN=PPIF PE=1 SV=1 - [PPIF_HUMAN]	21,74	3		1,255
Q8IXM6-2	Isoform 2 of Nurim OS=Homo sapiens GN=NRM - [NRM_HUMAN]	21,67	3	0,959	
Q9Y3B2	Exosome complex component CSL4 OS=Homo sapiens GN=EXOSC1 PE=1 SV=1 - [EXOS1_HUMAN]	21,54	3		
O60830	Mitochondrial import inner membrane translocase subunit Tim17-B OS=Homo sapiens GN=TIMM17B PE=1 SV=1 - [TI17B_HUMAN]	21,51	3	1,290	1,344
Q99828	Calcium and integrin-binding protein 1 OS=Homo sapiens GN=CIB1 PE=1 SV=4 - [CIB1_HUMAN]	21,47	3	0,234	0,151
Q9BRT2	Uncharacterized protein C6orf125 OS=Homo sapiens GN=C6orf125 PE=1 SV=1 - [CF125_HUMAN]	21,43	3	1,575	1,778
P08779	Keratin, type I cytoskeletal 16 OS=Homo sapiens GN=KRT16 PE=1 SV=4 - [K1C16_HUMAN]	21,14	3	1,717	1,454
P51452	Dual specificity protein phosphatase 3 OS=Homo sapiens GN=DUSP3 PE=1 SV=1 - [DUS3_HUMAN]	21,08	3	0,996	
Q9ULC4	Malignant T-cell-amplified sequence 1 OS=Homo sapiens GN=MCTS1 PE=1 SV=1 - [MCTS1_HUMAN]	20,99	3		

Q96K17	Transcription factor BTF3 homolog 4 OS=Homo sapiens GN=BTF3L4 PE=1 SV=1 - [BT3L4_HUMAN]	20,89	3	
Q9BYC9	39S ribosomal protein L20, mitochondrial OS=Homo sapiens GN=MRPL20 PE=1 SV=1 - [RM20_HUMAN]	20,81	3	1,057
P47755	F-actin-capping protein subunit alpha-2 OS=Homo sapiens GN=CAPZA2 PE=1 SV=3 - [CAZA2_HUMAN]	20,63	3	1,056
O15347	High mobility group protein B3 OS=Homo sapiens GN=HMGB3 PE=1 SV=4 - [HMGB3_HUMAN]	20,50	3	0,151
Q9BSF4	Uncharacterized protein C19orf52 OS=Homo sapiens GN=C19orf52 PE=1 SV=2 - [CS052_HUMAN]	20,38	3	1,216
P32970	CD70 antigen OS=Homo sapiens GN=CD70 PE=1 SV=2 - [CD70_HUMAN]	20,21	3	1,668
P49914	5-formyltetrahydrofolate cyclo-ligase OS=Homo sapiens GN=MTHFS PE=1 SV=2 - [MTHFS_HUMAN]	20,20	3	0,578
O43678	NADH dehydrogenase [ubiquinone] 1 alpha subcomplex subunit 2 OS=Homo sapiens GN=NDUFA2 PE=1 SV=3 - [NDUA2_HUMAN]	20,20	3	1,235
Q6UW68	Transmembrane protein 205 OS=Homo sapiens GN=TMEM205 PE=1 SV=1 - [TM205_HUMAN]	20,11	3	0,631
Q9Y584	Mitochondrial import inner membrane translocase subunit Tim22 OS=Homo sapiens GN=TIMM22 PE=1 SV=2 - [TIM22_HUMAN]	20,10	3	1,010
Q6P1S2	Uncharacterized protein C3orf33 OS=Homo sapiens GN=C3orf33 PE=1 SV=2 - [CC033_HUMAN]	20,07	3	1,068
P55789-2	Isoform 2 of FAD-linked sulfhydryl oxidase ALR OS=Homo sapiens GN=GFER - [ALR_HUMAN]	20,00	3	0,990
Q8N511	Transmembrane protein 199 OS=Homo sapiens GN=TMEM199 PE=1 SV=1 - [TM199_HUMAN]	19,71	3	0,977
O15498	Synaptobrevin homolog YKT6 OS=Homo sapiens GN=YKT6 PE=1 SV=1 - [YKT6_HUMAN]	19,70	3	1,007
Q6P587	Acylpyruvase FAHD1, mitochondrial OS=Homo sapiens GN=FAHD1 PE=1 SV=2 - [FAHD1_HUMAN]	19,64	3	0,761
P21741	Midkine OS=Homo sapiens GN=MDK PE=1 SV=1 - [MK_HUMAN]	19,58	3	1,295
Q96B36	Proline-rich AKT1 substrate 1 OS=Homo sapiens GN=AKT1S1 PE=1 SV=1 - [AKTS1_HUMAN]	19,53	3	
P02533	Keratin, type I cytoskeletal 14 OS=Homo sapiens GN=KRT14 PE=1 SV=4 - [K1C14_HUMAN]	19,49	3	0,964
Q9BVC5	Ashwin OS=Homo sapiens GN=C2orf49 PE=1 SV=1 - [ASHWN_HUMAN]	19,40	3	
O95639	Cleavage and polyadenylation specificity factor subunit 4 OS=Homo sapiens GN=CPSF4 PE=1 SV=1 - [CPSF4_HUMAN]	19,33	3	
O00124	UBX domain-containing protein 8 OS=Homo sapiens GN=UBXN8 PE=1 SV=2 - [UBXN8_HUMAN]	19,26	3	1,790
P61086	Ubiquitin-conjugating enzyme E2 K OS=Homo sapiens GN=UBE2K PE=1 SV=3 - [UBE2K_HUMAN]	19,00	3	0,918
P56556	NADH dehydrogenase [ubiquinone] 1 alpha subcomplex subunit 6 OS=Homo sapiens GN=NDUFA6 PE=1 SV=3 - [NDUA6_HUMAN]	18,83	3	0,929
P47813	Eukaryotic translation initiation factor 1A, X-chromosomal OS=Homo sapiens GN=EIF1AX PE=1 SV=2 - [IF1AX_HUMAN]	18,75	3	0,942
Q5BJF2	Transmembrane protein 97 OS=Homo sapiens GN=TMEM97 PE=1 SV=1 - [TMM97_HUMAN]	18,75	3	1,292
Q8TEA8	D-tyrosyl-tRNA(Tyr) deacylase 1 OS=Homo sapiens GN=DTD1 PE=1 SV=2 - [DTD1_HUMAN]	18,66	3	
P52565	Rho GDP-dissociation inhibitor 1 OS=Homo sapiens GN=ARHGDIA PE=1 SV=3 - [GDIR1_HUMAN]	18,63	3	0,761
F6R3U9	HLA class I histocompatibility antigen, alpha chain F (Fragment) OS=Homo sapiens GN=HLA-F PE=3 SV=1 - [F6R3U9_HUMAN]	18,56	3	
P59998	Actin-related protein 2/3 complex subunit 4 OS=Homo sapiens GN=ARPC4 PE=1 SV=3 - [ARPC4_HUMAN]	18,45	3	0,716
Q9NVS9	Pyridoxine-5'-phosphate oxidase OS=Homo sapiens GN=PNPO PE=1 SV=1 - [PNPO_HUMAN]	18,39	3	
O43181	NADH dehydrogenase [ubiquinone] iron-sulfur protein 4, mitochondrial OS=Homo sapiens GN=NDUFS4 PE=1 SV=1 - [NDUS4_HUMAN]	18,29	3	0,843
Q8TCC3	39S ribosomal protein L30, mitochondrial OS=Homo sapiens GN=MRPL30 PE=1 SV=1 - [RM30_HUMAN]	18,01	3	0,782
P06730	Eukaryotic translation initiation factor 4E OS=Homo sapiens GN=EIF4E PE=1 SV=2 - [IF4E_HUMAN]	17,97	3	0,732
Q9UFW8	CGG triplet repeat-binding protein 1 OS=Homo sapiens GN=CGGBP1 PE=1 SV=2 - [CGBP1_HUMAN]	17,96	3	
Q9Y689	ADP-ribosylation factor-like protein 5A OS=Homo sapiens GN=ARL5A PE=1 SV=1 - [ARL5A_HUMAN]	17,88	3	1,017
Q9BSH4	Translational activator of cytochrome c oxidase 1 OS=Homo sapiens GN=TACO1 PE=1 SV=1 - [TACO1_HUMAN]	17,85	3	0,704
P36969	Phospholipid hydroperoxide glutathione peroxidase, mitochondrial OS=Homo sapiens GN=GPX4 PE=1 SV=3 - [GPX4_HUMAN]	17,77	3	0,717
Q96KR6	Protein FAM210B OS=Homo sapiens GN=FAM210B PE=1 SV=2 - [F210B_HUMAN]	17,71	3	1,249
P00491	Purine nucleoside phosphorylase OS=Homo sapiens GN=PNP PE=1 SV=2 - [PNPH_HUMAN]	17,65	3	0,649
P60903	Protein S100-A10 OS=Homo sapiens GN=S100A10 PE=1 SV=2 - [S10AA_HUMAN]	17,53	3	0,650
P02686	Myelin basic protein OS=Homo sapiens GN=MBP PE=1 SV=3 - [MBP_HUMAN]	17,43	3	0,876
P52298	Nuclear cap-binding protein subunit 2 OS=Homo sapiens GN=NCBP2 PE=1 SV=1 - [NCBP2_HUMAN]	17,31	3	
Q8N565	Melanoregulin OS=Homo sapiens GN=MREG PE=1 SV=1 - [MREG_HUMAN]	17,29	3	0,697
O75431	Metaxin-2 OS=Homo sapiens GN=MTX2 PE=1 SV=1 - [MTX2_HUMAN]	17,11	3	0,968
P68402	Platelet-activating factor acetylhydrolase IB subunit beta OS=Homo sapiens GN=PAFAH1B2 PE=1 SV=1 - [PA1B2_HUMAN]	17,03	3	0,762
O95400	CD2 antigen cytoplasmic tail-binding protein 2 OS=Homo sapiens GN=CD2BP2 PE=1 SV=1 - [CD2B2_HUMAN]	17,01	3	
O43396	Thioredoxin-like protein 1 OS=Homo sapiens GN=TXNL1 PE=1 SV=3 - [TXNL1_HUMAN]	16,96	3	0,751
Q6UXV4	Apolipoprotein O-like OS=Homo sapiens GN=APOOL PE=1 SV=1 - [APOOL_HUMAN]	16,79	3	0,177
Q9NVZ3	Adaptin ear-binding coat-associated protein 2 OS=Homo sapiens GN=NECAP2 PE=1 SV=1 - [NECP2_HUMAN]	16,73	3	0,821
Q9NPF0	CD320 antigen OS=Homo sapiens GN=CD320 PE=1 SV=1 - [CD320_HUMAN]	16,67	3	1,828
O95169	NADH dehydrogenase [ubiquinone] 1 beta subcomplex subunit 8, mitochondrial OS=Homo sapiens GN=NDUFB8 PE=1 SV=1 - [NDUB8_HU	16,67	3	1,114
O75223	Gamma-glutamylcyclotransferase OS=Homo sapiens GN=GGCT PE=1 SV=1 - [GGCT_HUMAN]	16,49	3	0,528
Q9H2W6	39S ribosomal protein L46, mitochondrial OS=Homo sapiens GN=MRPL46 PE=1 SV=1 - [RM46_HUMAN]	16,49	3	0,817
Q9Y3D6	Mitochondrial fission 1 protein OS=Homo sapiens GN=FIS1 PE=1 SV=2 - [FIS1_HUMAN]	16,45	3	1,044
O15431	High affinity copper uptake protein 1 OS=Homo sapiens GN=SLC31A1 PE=1 SV=1 - [COPT1_HUMAN]	16,32	3	1,398
O60828	Polyglutamine-binding protein 1 OS=Homo sapiens GN=PQBP1 PE=1 SV=1 - [PQBP1_HUMAN]	16,23	3	
O43583	Density-regulated protein OS=Homo sapiens GN=DENR PE=1 SV=2 - [DENR_HUMAN]	16,16	3	
P62993	Growth factor receptor-bound protein 2 OS=Homo sapiens GN=GRB2 PE=1 SV=1 - [GRB2_HUMAN]	16,13	3	0,544
Q9Y224	UPF0568 protein C14orf166 OS=Homo sapiens GN=C14orf166 PE=1 SV=1 - [CN166_HUMAN]	15,98	3	0,778
Q99426	Tubulin-folding cofactor B OS=Homo sapiens GN=TBCB PE=1 SV=2 - [TBCB_HUMAN]	15,98	3	0,850
Q9UNP9	Peptidyl-prolyl cis-trans isomerase E OS=Homo sapiens GN=PPIE PE=1 SV=1 - [PPIE_HUMAN]	15,95	3	1,088
O75663	TIP41-like protein OS=Homo sapiens GN=TIPRL PE=1 SV=2 - [TIPRL_HUMAN]	15,81	3	0,864
Q68D91	Metallo-beta-lactamase domain-containing protein 2 OS=Homo sapiens GN=MBLAC2 PE=1 SV=3 - [MBLC2_HUMAN]	15,77	3	1,950
Q8WXX5	DnaJ homolog subfamily C member 9 OS=Homo sapiens GN=DNAJC9 PE=1 SV=1 - [DNJC9_HUMAN]	15,77	3	
Q9Y3Y2	Chromatin target of PRMT1 protein OS=Homo sapiens GN=CHTOP PE=1 SV=2 - [CHTOP_HUMAN]	15,73	3	1,216
Q92599	Septin-8 OS=Homo sapiens GN=SEPT8 PE=1 SV=4 - [SEPT8_HUMAN]	15,73	3	1,178
Q9BUR5	Apolipoprotein O OS=Homo sapiens GN=APOO PE=1 SV=1 - [APOO_HUMAN]	15,66	3	1,509
Q5UCC4	UPF0510 protein INM02 OS=Homo sapiens GN=C19orf63 PE=1 SV=1 - [INM02_HUMAN]	15,65	3	0,963
A6NKF9	Putative Golgi pH regulator C OS=Homo sapiens GN=GPR89C PE=5 SV=2 - [GPHRC_HUMAN]	15,63	3	1,198
Q9NX20	39S ribosomal protein L16, mitochondrial OS=Homo sapiens GN=MRPL16 PE=1 SV=1 - [RM16_HUMAN]	15,54	3	0,989
P30043	Flavin reductase (NADPH) OS=Homo sapiens GN=BLVRB PE=1 SV=3 - [BLVRB_HUMAN]	15,53	3	0,522
P18827	Syndecan-1 OS=Homo sapiens GN=SDC1 PE=1 SV=3 - [SDC1_HUMAN]	15,48	3	0,517
Q12983	BCL2/adenovirus E1B 19 kDa protein-interacting protein 3 OS=Homo sapiens GN=BNIP3 PE=1 SV=2 - [BNIP3_HUMAN]	15,46	3	1,066
Q9Y315	Putative deoxyribose-phosphate aldolase OS=Homo sapiens GN=DERA PE=1 SV=2 - [DEOC_HUMAN]	15,41	3	0,800
P04183	Thymidine kinase, cytosolic OS=Homo sapiens GN=TK1 PE=1 SV=2 - [KITH_HUMAN]	15,38	3	0,764
P07477	Trypsin-1 OS=Homo sapiens GN=PRSS1 PE=1 SV=1 - [TRY1_HUMAN]	15,38	3	1,506
Q7Z4W1	L-xylulose reductase OS=Homo sapiens GN=DCXR PE=1 SV=2 - [DCXR_HUMAN]	15,16	3	0,639
O14734	Acyl-coenzyme A thioesterase 8 OS=Homo sapiens GN=ACOT8 PE=1 SV=1 - [ACOT8_HUMAN]	15,05	3	1,686
Q96BK5	PIN2/TERF1-interacting telomerase inhibitor 1 OS=Homo sapiens GN=PINX1 PE=1 SV=2 - [PINX1_HUMAN]	14,94	3	
P10768	S-formylglutathione hydrolase OS=Homo sapiens GN=ESD PE=1 SV=2 - [ESTD_HUMAN]	14,89	3	1,180
O75351	Vacuolar protein sorting-associated protein 4B OS=Homo sapiens GN=VPS4B PE=1 SV=2 - [VPS4B_HUMAN]	14,86	3	0,685
Q96K19	RING finger protein 170 OS=Homo sapiens GN=RNF170 PE=2 SV=2 - [RN170_HUMAN]	14,73	3	0,953
Q9NV92	NEDD4 family-interacting protein 2 OS=Homo sapiens GN=NDFIP2 PE=1 SV=2 - [NFIP2_HUMAN]	14,58	3	1,203
P63279	SUMO-conjugating enzyme UBC9 OS=Homo sapiens GN=UBE2I PE=1 SV=1 - [UBC9_HUMAN]	14,56	3	
P20618	Proteasome subunit beta type-1 OS=Homo sapiens GN=PSMB1 PE=1 SV=2 - [PSB1_HUMAN]	14,52	3	
O15235	28S ribosomal protein S12, mitochondrial OS=Homo sapiens GN=MRPS12 PE=1 SV=1 - [RT12_HUMAN]	14,49	3	0,913
O75190	DnaJ homolog subfamily B member 6 OS=Homo sapiens GN=DNAJB6 PE=1 SV=2 - [DNJB6_HUMAN]	14,42	3	1,027
Q96HV5	Transmembrane protein 41A OS=Homo sapiens GN=TMEM41A PE=2 SV=1 - [TM41A_HUMAN]	14,39	3	0,846
P15407	Fos-related antigen 1 OS=Homo sapiens GN=FOSL1 PE=1 SV=1 - [FOSL1_HUMAN]	14,39	3	0,802
Q96DH6	RNA-binding protein Musashi homolog 2 OS=Homo sapiens GN=MSI2 PE=1 SV=1 - [MSI2H_HUMAN]	14,33	3	
O43674	NADH dehydrogenase [ubiquinone] 1 beta subcomplex subunit 5, mitochondrial OS=Homo sapiens GN=NDUFB5 PE=1 SV=1 - [NDUB5_HU	14,29	3	1,486
O43291	Kunitz-type protease inhibitor 2 OS=Homo sapiens GN=SPINT2 PE=1 SV=2 - [SPIT2_HUMAN]	14,29	3	0,992
Q9Y2C3	Beta-1,3-galactosyltransferase 5 OS=Homo sapiens GN=B3GALT5 PE=1 SV=1 - [B3GT5_HUMAN]	14,19	3	0,925
Q969G5	Protein kinase C delta-binding protein OS=Homo sapiens GN=PRKCDBP PE=1 SV=3 - [PRDBP_HUMAN]	14,18	3	1,110
Q14919	Dr1-associated corepressor OS=Homo sapiens GN=DRAP1 PE=1 SV=3 - [NC2A_HUMAN]	14,15	3	
Q15056	Eukaryotic translation initiation factor 4H OS=Homo sapiens GN=EIF4H PE=1 SV=5 - [IF4H_HUMAN]	14,11	3	
O75569	Interferon-inducible double stranded RNA-dependent protein kinase activator A OS=Homo sapiens GN=PRKRA PE=1 SV=1 - [PRKRA_HU	14,06	3	
P00813	Adenosine deaminase OS=Homo sapiens GN=ADA PE=1 SV=3 - [ADA_HUMAN]	14,05	3	0,720
O43768	Alpha-endosulfine OS=Homo sapiens GN=ENSA PE=1 SV=1 - [ENSA_HUMAN]	14,05	3	
Q13555	Calcium/calmodulin-dependent protein kinase type II subunit gamma OS=Homo sapiens GN=CAMK2G PE=1 SV=3 - [KCC2G_HUMAN]	13,98	3	0,505
P22830	Ferrochelatase, mitochondrial OS=Homo sapiens GN=FECH PE=1 SV=2 - [HEMH_HUMAN]	13,95	3	1,119
P15121	Aldose reductase OS=Homo sapiens GN=AKR1B1 PE=1 SV=3 - [ALDR_HUMAN]	13,92	3	1,604
O75608	Acyl-protein thioesterase 1 OS=Homo sapiens GN=LYPLA1 PE=1 SV=1 - [LYPA1_HUMAN]	13,91	3	0,862
A0PJW6	Transmembrane protein 223 OS=Homo sapiens GN=TMEM223 PE=1 SV=1 - [TM223_HUMAN]	13,86	3	1,182
O60637	Tetraspanin-3 OS=Homo sapiens GN=TSPAN3 PE=2 SV=1 - [TSN3_HUMAN]	13,83	3	1,170
Q6DKI1	60S ribosomal protein L7-like 1 OS=Homo sapiens GN=RPL7L1 PE=1 SV=1 - [RL7L_HUMAN]	13,82	3	0,916
Q9NS00	Glycoprotein-N-acetylgalactosamine 3-beta-galactosyltransferase 1 OS=Homo sapiens GN=C1GALT1 PE=1 SV=1 - [C1GLT_HUMAN]	13,77	3	0,753
P81605	Dermcidin OS=Homo sapiens GN=DCD PE=1 SV=2 - [DCD_HUMAN]	13,64	3	2,225
Q9NQS1	Cell death regulator Aven OS=Homo sapiens GN=AVEN PE=1 SV=1 - [AVEN_HUMAN]	13,54	3	1,429
Q6UX53	Methyltransferase-like protein 7B OS=Homo sapiens GN=METTL7B PE=2 SV=2 - [MET7B_HUMAN]	13,52	3	0,527
Q9P015	39S ribosomal protein L15, mitochondrial OS=Homo sapiens GN=MRPL15 PE=1 SV=1 - [RM15_HUMAN]	13,51	3	1,116
Q9H9Y2	Ribosome production factor 1 OS=Homo sapiens GN=RPF1 PE=1 SV=2 - [RPF1_HUMAN]	13,47	3	1,102
Q9NWQ8	Phosphoprotein associated with glycosphingolipid-enriched microdomains 1 OS=Homo sapiens GN=PAG1 PE=1 SV=2 - [PAG1_HUMAN]	13,43	3	1,563
P49721	Proteasome subunit beta type-2 OS=Homo sapiens GN=PSMB2 PE=1 SV=1 - [PSB2_HUMAN]	13,43	3	0,836
Q96CN7	Isochorismatase domain-containing protein 1 OS=Homo sapiens GN=ISOC1 PE=1 SV=3 - [ISOC1_HUMAN]	13,42	3	
Q9UBQ7	Glyoxylate reductase/hydroxypyruvate reductase OS=Homo sapiens GN=GRHPR PE=1 SV=1 - [GRHPR_HUMAN]	13,41	3	0,801
P51665	26S proteasome non-ATPase regulatory subunit 7 OS=Homo sapiens GN=PSMD7 PE=1 SV=2 - [PSD7_HUMAN]	13,27	3	0,772
O14562	Ubiquitin domain-containing protein UBFD1 OS=Homo sapiens GN=UBFD1 PE=1 SV=2 - [UBFD1_HUMAN]	13,27	3	
Q6NXT4	Zinc transporter 6 OS=Homo sapiens GN=SLC30A6 PE=1 SV=2 - [ZNT6_HUMAN]	13,23	3	0,936
Q9P013	Protein CWC15 homolog OS=Homo sapiens GN=CWC15 PE=1 SV=2 - [CWC15_HUMAN]	13,10	3	
Q9Y3B8	Oligoribonuclease, mitochondrial OS=Homo sapiens GN=REXO2 PE=1 SV=3 - [ORN_HUMAN]	13,08	3	0,924
Q66PJ3	ADP-ribosylation factor-like protein 6-interacting protein 4 OS=Homo sapiens GN=ARL6IP4 PE=1 SV=1 - [AR6P4_HUMAN]	13,06	3	
Q9NSI2	Protein FAM207A OS=Homo sapiens GN=FAM207A PE=1 SV=2 - [F207A_HUMAN]	13,04	3	0,848
P62714	Serine/threonine-protein phosphatase 2A catalytic subunit beta isoform OS=Homo sapiens GN=PPP2CB PE=1 SV=1 - [PP2AB_HUMAN]	12,94	3	0,702
Q11201	CMP-N-acetylneuraminate-beta-galactosamide-alpha-2,3-sialyltransferase 1 OS=Homo sapiens GN=ST3GAL1 PE=2 SV=1 - [SIA4A_HUMA	12,94	3	0,952
P28070	Proteasome subunit beta type-4 OS=Homo sapiens GN=PSMB4 PE=1 SV=4 - [PSB4_HUMAN]	12,88	3	1,151
Q9BQB6	Vitamin K epoxide reductase complex subunit 1 OS=Homo sapiens GN=VKORC1 PE=1 SV=1 - [VKOR1_HUMAN]	12,88	3	1,781
P32856	Syntaxin-2 OS=Homo sapiens GN=STX2 PE=1 SV=3 - [STX2_HUMAN]	12,85	3	1,307
Q8IWE4	DCN1-like protein 3 OS=Homo sapiens GN=DCUN1D3 PE=2 SV=1 - [DCNL3_HUMAN]	12,83	3	1,050
Q9Y6M0	Testisin OS=Homo sapiens GN=PRSS21 PE=2 SV=1 - [TEST_HUMAN]	12,74	3	1,271
P78346	Ribonuclease P protein subunit p30 OS=Homo sapiens GN=RPP30 PE=1 SV=1 - [RPP30_HUMAN]	12,69	3	0,815
F5HFY4	Nucleosome assembly protein 1-like 4 OS=Homo sapiens GN=NAP1L4b PE=2 SV=1 - [F5HFY4_HUMAN]	12,69	3	
Q9BYD6	39S ribosomal protein L1, mitochondrial OS=Homo sapiens GN=MRPL1 PE=1 SV=2 - [RM01_HUMAN]	12,62	3	0,908
Q9BZM5	NKG2D ligand 2 OS=Homo sapiens GN=ULBP2 PE=1 SV=1 - [N2DL2_HUMAN]	12,60	3	1,515
Q9NXA8	NAD-dependent lysine demalonylase and desuccinylase sirtuin-5, mitochondrial OS=Homo sapiens GN=SIRT5 PE=1 SV=2 - [SIRT5_HUM	12,58	3	
Q8IUW5	RELT-like protein 1 OS=Homo sapiens GN=RELL1 PE=1 SV=1 - [RELL1_HUMAN]	12,55	3	1,937
Q9BU76	Multiple myeloma tumor-associated protein 2 OS=Homo sapiens GN=MMTAG2 PE=1 SV=1 - [MMTA2_HUMAN]	12,55	3	
O14684	Prostaglandin E synthase OS=Homo sapiens GN=PTGES PE=1 SV=2 - [PTGES_HUMAN]	12,50	3	1,799
P78406	mRNA export factor OS=Homo sapiens GN=RAE1 PE=1 SV=1 - [RAE1L_HUMAN]	12,50	3	1,202
Q9UBP9	PTB domain-containing engulfment adapter protein 1 OS=Homo sapiens GN=GULP1 PE=1 SV=1 - [GULP1_HUMAN]	12,50	3	
Q9Y316	Protein MEMO1 OS=Homo sapiens GN=MEMO1 PE=1 SV=1 - [MEMO1_HUMAN]	12,46	3	0,821
Q9H6H4	Receptor expression-enhancing protein 4 OS=Homo sapiens GN=REEP4 PE=1 SV=1 - [REEP4_HUMAN]	12,45	3	1,147
Q6IA17	Single Ig IL-1-related receptor OS=Homo sapiens GN=SIGIRR PE=1 SV=3 - [SIGIR_HUMAN]	12,44	3	0,223
Q9P016	Thymocyte nuclear protein 1 OS=Homo sapiens GN=THYN1 PE=1 SV=1 - [THYN1_HUMAN]	12,44	3	
Q00403	Transcription initiation factor IIB OS=Homo sapiens GN=GTF2B PE=1 SV=1 - [TF2B_HUMAN]	12,34	3	0,722
P04156	Major prion protein OS=Homo sapiens GN=PRNP PE=1 SV=1 - [PRIO_HUMAN]	12,25	3	1,239
Q969E2	Secretory carrier-associated membrane protein 4 OS=Homo sapiens GN=SCAMP4 PE=2 SV=1 - [SCAM4_HUMAN]	12,23	3	1,169
Q9Y570	Protein phosphatase methylesterase 1 OS=Homo sapiens GN=PPME1 PE=1 SV=3 - [PPME1_HUMAN]	12,18	3	0,870
Q9NVA2	Septin-11 OS=Homo sapiens GN=SEPT11 PE=1 SV=3 - [SEP11_HUMAN]	12,12	3	
P55039	Developmentally-regulated GTP-binding protein 2 OS=Homo sapiens GN=DRG2 PE=1 SV=1 - [DRG2_HUMAN]	12,09	3	0,827
O60784	Target of Myb protein 1 OS=Homo sapiens GN=TOM1 PE=1 SV=2 - [TOM1_HUMAN]	11,99	3	
O75521	Enoyl-CoA delta isomerase 2, mitochondrial OS=Homo sapiens GN=ECI2 PE=1 SV=4 - [ECI2_HUMAN]	11,93	3	1,079
Q9Y371	Endophilin-B1 OS=Homo sapiens GN=SH3GLB1 PE=1 SV=1 - [SHLB1_HUMAN]	11,78	3	0,911
Q9UNI6	Dual specificity protein phosphatase 12 OS=Homo sapiens GN=DUSP12 PE=1 SV=1 - [DUS12_HUMAN]	11,76	3	
P28072	Proteasome subunit beta type-6 OS=Homo sapiens GN=PSMB6 PE=1 SV=4 - [PSB6_HUMAN]	11,72	3	1,410
Q9BUV8	Uncharacterized protein C20orf24 OS=Homo sapiens GN=C20orf24 PE=2 SV=1 - [CT024_HUMAN]	11,68	3	1,020
Q5BJD5	Transmembrane protein 41B OS=Homo sapiens GN=TMEM41B PE=1 SV=1 - [TM41B_HUMAN]	11,68	3	1,218
Q96CT7	Coiled-coil domain-containing protein 124 OS=Homo sapiens GN=CCDC124 PE=1 SV=1 - [CC124_HUMAN]	11,66	3	
O75436	Vacuolar protein sorting-associated protein 26A OS=Homo sapiens GN=VPS26A PE=1 SV=2 - [VP26A_HUMAN]	11,62	3	0,897
Q9GZL7	Ribosome biogenesis protein WDR12 OS=Homo sapiens GN=WDR12 PE=1 SV=2 - [WDR12_HUMAN]	11,58	3	0,993
Q5T2W1	Na(+)/H(+) exchange regulatory cofactor NHE-RF3 OS=Homo sapiens GN=PDZK1 PE=1 SV=2 - [NHRF3_HUMAN]	11,56	3	**2,245**
Q99685	Monoglyceride lipase OS=Homo sapiens GN=MGLL PE=1 SV=2 - [MGLL_HUMAN]	11,55	3	0,738
Q16555	Dihydropyrimidinase-related protein 2 OS=Homo sapiens GN=DPYSL2 PE=1 SV=1 - [DPYL2_HUMAN]	11,54	3	0,774
O00764	Pyridoxal kinase OS=Homo sapiens GN=PDXK PE=1 SV=1 - [PDXK_HUMAN]	11,54	3	0,656
Q6SPF0	Atherin OS=Homo sapiens GN=SAMD1 PE=1 SV=1 - [SAMD1_HUMAN]	11,52	3	1,186
Q9H1E5	Thioredoxin-related transmembrane protein 4 OS=Homo sapiens GN=TMX4 PE=1 SV=1 - [TMX4_HUMAN]	11,46	3	1,091
P20749	B-cell lymphoma 3 protein OS=Homo sapiens GN=BCL3 PE=1 SV=2 - [BCL3_HUMAN]	11,45	3	
Q9BY42	UPF0549 protein C20orf43 OS=Homo sapiens GN=C20orf43 PE=1 SV=3 - [CT043_HUMAN]	11,44	3	
Q8NFZ8	Cell adhesion molecule 4 OS=Homo sapiens GN=CADM4 PE=1 SV=1 - [CADM4_HUMAN]	11,34	3	1,303
Q9Y385	Ubiquitin-conjugating enzyme E2 J1 OS=Homo sapiens GN=UBE2J1 PE=1 SV=2 - [UB2J1_HUMAN]	11,32	3	1,140

P48735	Isocitrate dehydrogenase [NADP], mitochondrial OS=Homo sapiens GN=IDH2 PE=1 SV=2 - [IDHP_HUMAN]	11,28
Q9NZI8	Insulin-like growth factor 2 mRNA-binding protein 1 OS=Homo sapiens GN=IGF2BP1 PE=1 SV=2 - [IF2B1_HUMAN]	11,09
O75072	Fukutin OS=Homo sapiens GN=FKTN PE=1 SV=2 - [FKTN_HUMAN]	11,06
Q6P6B1	Uncharacterized protein C8orf47 OS=Homo sapiens GN=C8orf47 PE=2 SV=1 - [CH047_HUMAN]	10,96
P83916	Chromobox protein homolog 1 OS=Homo sapiens GN=CBX1 PE=1 SV=1 - [CBX1_HUMAN]	10,81
Q9H7B2	Ribosome production factor 2 homolog OS=Homo sapiens GN=RPF2 PE=1 SV=2 - [RPF2_HUMAN]	10,78
Q8IUS5	Epoxide hydrolase 4 OS=Homo sapiens GN=EPHX4 PE=2 SV=2 - [EPHX4_HUMAN]	10,77
Q92734	Protein TFG OS=Homo sapiens GN=TFG PE=1 SV=2 - [TFG_HUMAN]	10,75
Q8WWC4	Uncharacterized protein C2orf47, mitochondrial OS=Homo sapiens GN=C2orf47 PE=1 SV=1 - [CB047_HUMAN]	10,65
Q9UBU6	Protein FAM8A1 OS=Homo sapiens GN=FAM8A1 PE=1 SV=1 - [FA8A1_HUMAN]	10,65
Q14320	Protein FAM50A OS=Homo sapiens GN=FAM50A PE=1 SV=2 - [FA50A_HUMAN]	10,62
P06241	Tyrosine-protein kinase Fyn OS=Homo sapiens GN=FYN PE=1 SV=3 - [FYN_HUMAN]	10,43
Q9NZ09	Ubiquitin-associated protein 1 OS=Homo sapiens GN=UBAP1 PE=1 SV=1 - [UBAP1_HUMAN]	10,36
Q86UL3	Glycerol-3-phosphate acyltransferase 4 OS=Homo sapiens GN=AGPAT6 PE=1 SV=1 - [GPAT4_HUMAN]	10,31
Q9NNW7	Thioredoxin reductase 2, mitochondrial OS=Homo sapiens GN=TXNRD2 PE=1 SV=3 - [TRXR2_HUMAN]	10,31
O60256	Phosphoribosyl pyrophosphate synthase-associated protein 2 OS=Homo sapiens GN=PRPSAP2 PE=1 SV=1 - [KPRB_HUMAN]	10,30
Q6I9Y2	THO complex subunit 7 homolog OS=Homo sapiens GN=THOC7 PE=1 SV=3 - [THOC7_HUMAN]	10,29
P35030	Trypsin-3 OS=Homo sapiens GN=PRSS3 PE=1 SV=2 - [TRY3_HUMAN]	10,20
Q6NUK4	Receptor expression-enhancing protein 3 OS=Homo sapiens GN=REEP3 PE=2 SV=1 - [REEP3_HUMAN]	10,20
Q9NWR8	Coiled-coil domain-containing protein 109B OS=Homo sapiens GN=CCDC109B PE=1 SV=2 - [C109B_HUMAN]	10,12
Q96SQ9	Cytochrome P450 2S1 OS=Homo sapiens GN=CYP2S1 PE=2 SV=2 - [CP2S1_HUMAN]	10,12
Q9NRF8	CTP synthase 2 OS=Homo sapiens GN=CTPS2 PE=1 SV=1 - [PYRG2_HUMAN]	10,07
O43688	Lipid phosphate phosphohydrolase 2 OS=Homo sapiens GN=PPAP2C PE=1 SV=1 - [LPP2_HUMAN]	10,07
Q8NG11	Tetraspanin-14 OS=Homo sapiens GN=TSPAN14 PE=2 SV=1 - [TSN14_HUMAN]	10,00
Q96LZ7	Regulator of microtubule dynamics protein 2 OS=Homo sapiens GN=FAM82A1 PE=1 SV=2 - [RMD2_HUMAN]	10,00
Q68EM7	Rho GTPase-activating protein 17 OS=Homo sapiens GN=ARHGAP17 PE=1 SV=1 - [RHG17_HUMAN]	9,99
Q29980	MHC class I polypeptide-related sequence B OS=Homo sapiens GN=MICB PE=1 SV=1 - [MICB_HUMAN]	9,92
Q9UNE7	E3 ubiquitin-protein ligase CHIP OS=Homo sapiens GN=STUB1 PE=1 SV=2 - [CHIP_HUMAN]	9,90
P10646	Tissue factor pathway inhibitor OS=Homo sapiens GN=TFPI PE=1 SV=1 - [TFPI1_HUMAN]	9,87
Q5JTD0	Tight junction-associated protein 1 OS=Homo sapiens GN=TJAP1 PE=1 SV=1 - [TJAP1_HUMAN]	9,87
O60704	Protein-tyrosine sulfotransferase 2 OS=Homo sapiens GN=TPST2 PE=1 SV=1 - [TPST2_HUMAN]	9,81
Q8WWH5	Probable tRNA pseudouridine synthase 1 OS=Homo sapiens GN=TRUB1 PE=1 SV=1 - [TRUB1_HUMAN]	9,74
Q99816	Tumor susceptibility gene 101 protein OS=Homo sapiens GN=TSG101 PE=1 SV=2 - [TS101_HUMAN]	9,74
O60507	Protein-tyrosine sulfotransferase 1 OS=Homo sapiens GN=TPST1 PE=2 SV=1 - [TPST1_HUMAN]	9,73
Q8WUM9	Sodium-dependent phosphate transporter 1 OS=Homo sapiens GN=SLC20A1 PE=1 SV=1 - [S20A1_HUMAN]	9,72
O75616	GTPase Era, mitochondrial OS=Homo sapiens GN=ERAL1 PE=1 SV=2 - [ERAL1_HUMAN]	9,61
P19256	Lymphocyte function-associated antigen 3 OS=Homo sapiens GN=CD58 PE=1 SV=1 - [LFA3_HUMAN]	9,60
Q9BZK7	F-box-like/WD repeat-containing protein TBL1XR1 OS=Homo sapiens GN=TBL1XR1 PE=1 SV=1 - [TBL1R_HUMAN]	9,53
Q8N4A0	Polypeptide N-acetylgalactosaminyltransferase 4 OS=Homo sapiens GN=GALNT4 PE=1 SV=2 - [GALT4_HUMAN]	9,52
Q96DV4	39S ribosomal protein L38, mitochondrial OS=Homo sapiens GN=MRPL38 PE=1 SV=2 - [RM38_HUMAN]	9,47
Q92604	Acyl-CoA:lysophosphatidylglycerol acyltransferase 1 OS=Homo sapiens GN=LPGAT1 PE=2 SV=1 - [LGAT1_HUMAN]	9,46
Q9Y376	Calcium-binding protein 39 OS=Homo sapiens GN=CAB39 PE=1 SV=1 - [CAB39_HUMAN]	9,38
Q9UK59	Lariat debranching enzyme OS=Homo sapiens GN=DBR1 PE=1 SV=2 - [DBR1_HUMAN]	9,38
P61962	DDB1- and CUL4-associated factor 7 OS=Homo sapiens GN=DCAF7 PE=1 SV=1 - [DCAF7_HUMAN]	9,36
O00487	26S proteasome non-ATPase regulatory subunit 14 OS=Homo sapiens GN=PSMD14 PE=1 SV=1 - [PSDE_HUMAN]	9,35
Q8N6N3	UPF0690 protein C1orf52 OS=Homo sapiens GN=C1orf52 PE=1 SV=1 - [CA052_HUMAN]	9,34
P07711	Cathepsin L1 OS=Homo sapiens GN=CTSL1 PE=1 SV=2 - [CATL1_HUMAN]	9,31
P55061	Bax inhibitor 1 OS=Homo sapiens GN=TMBIM6 PE=1 SV=2 - [BI1_HUMAN]	9,28
P17275	Transcription factor jun-B OS=Homo sapiens GN=JUNB PE=1 SV=1 - [JUNB_HUMAN]	9,22
Q6DKK2	Tetratricopeptide repeat protein 19, mitochondrial OS=Homo sapiens GN=TTC19 PE=1 SV=4 - [TTC19_HUMAN]	9,21
Q7L4E1	Protein FAM73B OS=Homo sapiens GN=FAM73B PE=1 SV=1 - [FA73B_HUMAN]	9,11
Q9H497	Torsin-3A OS=Homo sapiens GN=TOR3A PE=1 SV=1 - [TOR3A_HUMAN]	9,07
Q08357	Sodium-dependent phosphate transporter 2 OS=Homo sapiens GN=SLC20A2 PE=1 SV=1 - [S20A2_HUMAN]	9,05
A4D1S0	Killer cell lectin-like receptor subfamily G member 2 OS=Homo sapiens GN=KLRG2 PE=1 SV=3 - [KLRG2_HUMAN]	9,05
Q15428	Splicing factor 3A subunit 2 OS=Homo sapiens GN=SF3A2 PE=1 SV=2 - [SF3A2_HUMAN]	9,05
Q14142	Tripartite motif-containing protein 14 OS=Homo sapiens GN=TRIM14 PE=2 SV=2 - [TRI14_HUMAN]	9,05
Q8NCH0	Carbohydrate sulfotransferase 14 OS=Homo sapiens GN=CHST14 PE=1 SV=2 - [CHSTE_HUMAN]	9,04
P16333	Cytoplasmic protein NCK1 OS=Homo sapiens GN=NCK1 PE=1 SV=1 - [NCK1_HUMAN]	9,02
Q9BY78	RING finger protein 26 OS=Homo sapiens GN=RNF26 PE=2 SV=1 - [RNF26_HUMAN]	9,01
Q96SL1	Disrupted in renal carcinoma protein 2 OS=Homo sapiens GN=DIRC2 PE=2 SV=1 - [DIRC2_HUMAN]	9,00
P30740	Leukocyte elastase inhibitor OS=Homo sapiens GN=SERPINB1 PE=1 SV=1 - [ILEU_HUMAN]	8,97
P19623	Spermidine synthase OS=Homo sapiens GN=SRM PE=1 SV=1 - [SPEE_HUMAN]	8,94
P21912	Succinate dehydrogenase [ubiquinone] iron-sulfur subunit, mitochondrial OS=Homo sapiens GN=SDHB PE=1 SV=3 - [DHSB_HUMAN]	8,93
Q8NBI6	Xyloside xylosyltransferase 1 OS=Homo sapiens GN=XXYLT1 PE=1 SV=1 - [XXLT1_HUMAN]	8,91
Q9UJW0	Dynactin subunit 4 OS=Homo sapiens GN=DCTN4 PE=1 SV=1 - [DCTN4_HUMAN]	8,91
Q96EC8	Protein YIPF6 OS=Homo sapiens GN=YIPF6 PE=2 SV=2 - [YIPF6_HUMAN]	8,90
Q96C86	m7GpppX diphosphatase OS=Homo sapiens GN=DCPS PE=1 SV=2 - [DCPS_HUMAN]	8,90
O94876	Transmembrane and coiled-coil domains protein 1 OS=Homo sapiens GN=TMCC1 PE=1 SV=3 - [TMCC1_HUMAN]	8,88
Q07352	Zinc finger protein 36, C3H1 type-like 1 OS=Homo sapiens GN=ZFP36L1 PE=1 SV=1 - [TISB_HUMAN]	8,88
Q9BT78	COP9 signalosome complex subunit 4 OS=Homo sapiens GN=COPS4 PE=1 SV=1 - [CSN4_HUMAN]	8,87
Q53G44	Interferon-induced protein 44-like OS=Homo sapiens GN=IFI44L PE=2 SV=3 - [IF44L_HUMAN]	8,85
P51608	Methyl-CpG-binding protein 2 OS=Homo sapiens GN=MECP2 PE=1 SV=1 - [MECP2_HUMAN]	8,85
P02649	Apolipoprotein E OS=Homo sapiens GN=APOE PE=1 SV=1 - [APOE_HUMAN]	8,83
O96009	Napsin-A OS=Homo sapiens GN=NAPSA PE=1 SV=1 - [NAPSA_HUMAN]	8,81
Q9UI10	Translation initiation factor eIF-2B subunit delta OS=Homo sapiens GN=EIF2B4 PE=1 SV=2 - [EI2BD_HUMAN]	8,80
Q9NPF5	DNA methyltransferase 1-associated protein 1 OS=Homo sapiens GN=DMAP1 PE=1 SV=1 - [DMAP1_HUMAN]	8,78
Q13155	Aminoacyl tRNA synthase complex-interacting multifunctional protein 2 OS=Homo sapiens GN=AIMP2 PE=1 SV=2 - [AIMP2_HUMAN]	8,75
O43865	Putative adenosylhomocysteinase 2 OS=Homo sapiens GN=AHCYL1 PE=1 SV=2 - [SAHH2_HUMAN]	8,68
A2RU67	Uncharacterized protein KIAA1467 OS=Homo sapiens GN=KIAA1467 PE=1 SV=1 - [K1467_HUMAN]	8,68
Q9NZH0	G-protein coupled receptor family C group 5 member B OS=Homo sapiens GN=GPRC5B PE=2 SV=2 - [GPC5B_HUMAN]	8,68
Q9Y6K0	Choline/ethanolaminephosphotransferase 1 OS=Homo sapiens GN=CEPT1 PE=1 SV=1 - [CEPT1_HUMAN]	8,65
Q9Y5J1	U3 small nucleolar RNA-associated protein 18 homolog OS=Homo sapiens GN=UTP18 PE=1 SV=3 - [UTP18_HUMAN]	8,63
P48059	LIM and senescent cell antigen-like-containing domain protein 1 OS=Homo sapiens GN=LIMS1 PE=1 SV=4 - [LIMS1_HUMAN]	8,62
P48729	Casein kinase I isoform alpha OS=Homo sapiens GN=CSNK1A1 PE=1 SV=2 - [KC1A_HUMAN]	8,61
Q9P270	SLAIN motif-containing protein 2 OS=Homo sapiens GN=SLAIN2 PE=1 SV=2 - [SLAI2_HUMAN]	8,61
P51689	Arylsulfatase D OS=Homo sapiens GN=ARSD PE=1 SV=2 - [ARSD_HUMAN]	8,60
Q9Y6G9	Cytoplasmic dynein 1 light intermediate chain 1 OS=Homo sapiens GN=DYNC1LI1 PE=1 SV=3 - [DC1L1_HUMAN]	8,60
P48556	26S proteasome non-ATPase regulatory subunit 8 OS=Homo sapiens GN=PSMD8 PE=1 SV=2 - [PSMD8_HUMAN]	8,57
Q9BV38	WD repeat-containing protein 18 OS=Homo sapiens GN=WDR18 PE=1 SV=2 - [WDR18_HUMAN]	8,56
Q9BQ67	Glutamate-rich WD repeat-containing protein 1 OS=Homo sapiens GN=GRWD1 PE=1 SV=1 - [GRWD1_HUMAN]	8,52
Q96PU8	Protein quaking OS=Homo sapiens GN=QKI PE=1 SV=1 - [QKI_HUMAN]	8,50
Q96F86	Enhancer of mRNA-decapping protein 3 OS=Homo sapiens GN=EDC3 PE=1 SV=1 - [EDC3_HUMAN]	8,46
Q9UJ14	Gamma-glutamyltransferase 7 OS=Homo sapiens GN=GGT7 PE=1 SV=2 - [GGT7_HUMAN]	8,46
Q5SQN1	Synaptosomal-associated protein 47 OS=Homo sapiens GN=SNAP47 PE=2 SV=3 - [SNP47_HUMAN]	8,41
Q96MC6	Hippocampus abundant transcript 1 protein OS=Homo sapiens GN=HIAT1 PE=2 SV=2 - [HIAT1_HUMAN]	8,37
Q4G0N4	NAD kinase domain-containing protein 1 OS=Homo sapiens GN=NADKD1 PE=1 SV=2 - [NAKD1_HUMAN]	8,37
O60353	Frizzled-6 OS=Homo sapiens GN=FZD6 PE=1 SV=2 - [FZD6_HUMAN]	8,36
Q06203	Amidophosphoribosyltransferase OS=Homo sapiens GN=PPAT PE=1 SV=1 - [PUR1_HUMAN]	8,32
Q9BW19	Kinesin-like protein KIFC1 OS=Homo sapiens GN=KIFC1 PE=1 SV=2 - [KIFC1_HUMAN]	8,32
Q15629	Translocating chain-associated membrane protein 1 OS=Homo sapiens GN=TRAM1 PE=1 SV=3 - [TRAM1_HUMAN]	8,29
P53582	Methionine aminopeptidase 1 OS=Homo sapiens GN=METAP1 PE=1 SV=2 - [AMPM1_HUMAN]	8,29
Q96K49	Transmembrane protein 87B OS=Homo sapiens GN=TMEM87B PE=1 SV=1 - [TM87B_HUMAN]	8,29
Q13530	Serine incorporator 3 OS=Homo sapiens GN=SERINC3 PE=2 SV=2 - [SERC3_HUMAN]	8,25
Q9BQA1	Methylosome protein 50 OS=Homo sapiens GN=WDR77 PE=1 SV=1 - [MEP50_HUMAN]	8,19
P50579	Methionine aminopeptidase 2 OS=Homo sapiens GN=METAP2 PE=1 SV=1 - [AMPM2_HUMAN]	8,16
Q9NX74	tRNA-dihydrouridine(20) synthase [NAD(P)+]-like OS=Homo sapiens GN=DUS2L PE=1 SV=1 - [DUS2L_HUMAN]	8,11
Q96IJ6	Mannose-1-phosphate guanyltransferase alpha OS=Homo sapiens GN=GMPPA PE=1 SV=1 - [GMPPA_HUMAN]	8,10
Q9Y3X0	Coiled-coil domain-containing protein 9 OS=Homo sapiens GN=CCDC9 PE=1 SV=1 - [CCDC9_HUMAN]	8,10
Q9Y5Q8	General transcription factor 3C polypeptide 5 OS=Homo sapiens GN=GTF3C5 PE=1 SV=2 - [TF3C5_HUMAN]	8,09
Q9BW27	Nuclear pore complex protein Nup85 OS=Homo sapiens GN=NUP85 PE=1 SV=1 - [NUP85_HUMAN]	8,08
Q96DZ1	Endoplasmic reticulum lectin 1 OS=Homo sapiens GN=ERLEC1 PE=1 SV=1 - [ERLEC_HUMAN]	8,07
Q8NAN2	Protein FAM73A OS=Homo sapiens GN=FAM73A PE=2 SV=1 - [FA73A_HUMAN]	8,07
Q15059	Bromodomain-containing protein 3 OS=Homo sapiens GN=BRD3 PE=1 SV=1 - [BRD3_HUMAN]	7,99
Q12996	Cleavage stimulation factor subunit 3 OS=Homo sapiens GN=CSTF3 PE=1 SV=1 - [CSTF3_HUMAN]	7,95
O95870	Abhydrolase domain-containing protein 16A OS=Homo sapiens GN=ABHD16A PE=1 SV=3 - [ABHGA_HUMAN]	7,89
Q6Y1H2	3-hydroxyacyl-CoA dehydratase 2 OS=Homo sapiens GN=PTPLB PE=1 SV=1 - [HACD2_HUMAN]	7,87
P82933	28S ribosomal protein S9, mitochondrial OS=Homo sapiens GN=MRPS9 PE=1 SV=2 - [RT09_HUMAN]	7,83
Q15291	Retinoblastoma-binding protein 5 OS=Homo sapiens GN=RBBP5 PE=1 SV=2 - [RBBP5_HUMAN]	7,81
Q765P7	MTSS1-like protein OS=Homo sapiens GN=MTSS1L PE=1 SV=1 - [MTSSL_HUMAN]	7,76
P84022	Mothers against decapentaplegic homolog 3 OS=Homo sapiens GN=SMAD3 PE=1 SV=1 - [SMAD3_HUMAN]	7,76
Q969Y2	tRNA modification GTPase GTPBP3, mitochondrial OS=Homo sapiens GN=GTPBP3 PE=2 SV=2 - [GTPB3_HUMAN]	7,72
Q9GZM5	Protein YIPF3 OS=Homo sapiens GN=YIPF3 PE=1 SV=1 - [YIPF3_HUMAN]	7,71
Q8NEW0	Zinc transporter 7 OS=Homo sapiens GN=SLC30A7 PE=1 SV=1 - [ZNT7_HUMAN]	7,71
Q92504	Zinc transporter SLC39A7 OS=Homo sapiens GN=SLC39A7 PE=1 SV=2 - [S39A7_HUMAN]	7,68
Q8WU20	Fibroblast growth factor receptor substrate 2 OS=Homo sapiens GN=FRS2 PE=1 SV=4 - [FRS2_HUMAN]	7,68
O95747	Serine/threonine-protein kinase OSR1 OS=Homo sapiens GN=OXSR1 PE=1 SV=1 - [OXSR1_HUMAN]	7,59
P48067-2	Isoform GlyT-1A of Sodium- and chloride-dependent glycine transporter 1 OS=Homo sapiens GN=SLC6A9 - [SC6A9_HUMAN]	7,58
Q9Y303	Putative N-acetylglucosamine-6-phosphate deacetylase OS=Homo sapiens GN=AMDHD2 PE=2 SV=2 - [NAGA_HUMAN]	7,58
Q9HBU6	Ethanolamine kinase 1 OS=Homo sapiens GN=ETNK1 PE=1 SV=1 - [EKI1_HUMAN]	7,52
Q9NRG9	Aladin OS=Homo sapiens GN=AAAS PE=1 SV=1 - [AAAS_HUMAN]	7,51
Q9Y2P8	RNA 3'-terminal phosphate cyclase-like protein OS=Homo sapiens GN=RCL1 PE=1 SV=3 - [RCL1_HUMAN]	7,51
Q8NE01	Metal transporter CNNM3 OS=Homo sapiens GN=CNNM3 PE=1 SV=1 - [CNNM3_HUMAN]	7,50
Q9H4L4	Sentrin-specific protease 3 OS=Homo sapiens GN=SENP3 PE=1 SV=2 - [SENP3_HUMAN]	7,49
Q04771	Activin receptor type-1 OS=Homo sapiens GN=ACVR1 PE=1 SV=1 - [ACVR1_HUMAN]	7,47
P43005	Excitatory amino acid transporter 3 OS=Homo sapiens GN=SLC1A1 PE=1 SV=2 - [EAA3_HUMAN]	7,44
O75354	Ectonucleoside triphosphate diphosphohydrolase 6 OS=Homo sapiens GN=ENTPD6 PE=1 SV=3 - [ENTP6_HUMAN]	7,44
P51116	Fragile X mental retardation syndrome-related protein 2 OS=Homo sapiens GN=FXR2 PE=1 SV=2 - [FXR2_HUMAN]	7,43
O14495	Lipid phosphate phosphohydrolase 3 OS=Homo sapiens GN=PPAP2B PE=1 SV=1 - [LPP3_HUMAN]	7,40
Q9UGH3	Solute carrier family 23 member 2 OS=Homo sapiens GN=SLC23A2 PE=1 SV=1 - [S23A2_HUMAN]	7,38
Q7KZI7	Serine/threonine-protein kinase MARK2 OS=Homo sapiens GN=MARK2 PE=1 SV=2 - [MARK2_HUMAN]	7,36
O43824	Putative GTP-binding protein 6 OS=Homo sapiens GN=GTPBP6 PE=2 SV=3 - [GTPB6_HUMAN]	7,36
Q8NHS3	Major facilitator superfamily domain-containing protein 8 OS=Homo sapiens GN=MFSD8 PE=1 SV=1 - [MFSD8_HUMAN]	7,34
Q9NZM5	Glioma tumor suppressor candidate region gene 2 protein OS=Homo sapiens GN=GLTSCR2 PE=1 SV=2 - [GSCR2_HUMAN]	7,32
F8W1P7	Natural resistance-associated macrophage protein 2 OS=Homo sapiens GN=SLC11A2 PE=4 SV=1 - [F8W1P7_HUMAN]	7,26
P29350	Tyrosine-protein phosphatase non-receptor type 6 OS=Homo sapiens GN=PTPN6 PE=1 SV=1 - [PTN6_HUMAN]	7,23
Q9NUQ8	ATP-binding cassette sub-family F member 3 OS=Homo sapiens GN=ABCF3 PE=1 SV=2 - [ABCF3_HUMAN]	7,19
Q9UHR5	SAP30-binding protein OS=Homo sapiens GN=SAP30BP PE=1 SV=1 - [S30BP_HUMAN]	7,14
Q9ULS5	Transmembrane and coiled-coil domains protein 3 OS=Homo sapiens GN=TMCC3 PE=2 SV=3 - [TMCC3_HUMAN]	7,13
Q9H1C4	Protein unc-93 homolog B1 OS=Homo sapiens GN=UNC93B1 PE=1 SV=2 - [UN93B_HUMAN]	7,04
Q96G03	Phosphoglucomutase-2 OS=Homo sapiens GN=PGM2 PE=1 SV=4 - [PGM2_HUMAN]	7,03
P43155	Carnitine O-acetyltransferase OS=Homo sapiens GN=CRAT PE=1 SV=5 - [CACP_HUMAN]	7,03
Q9NZT2	Opioid growth factor receptor OS=Homo sapiens GN=OGFR PE=1 SV=3 - [OGFR_HUMAN]	6,94
Q96CM3	RNA pseudouridylate synthase domain-containing protein 4 OS=Homo sapiens GN=RPUSD4 PE=2 SV=1 - [RUSD4_HUMAN]	6,90
Q9H0V1	Transmembrane protein 168 OS=Homo sapiens GN=TMEM168 PE=1 SV=2 - [TM168_HUMAN]	6,89
Q9Y5X1	Sorting nexin-9 OS=Homo sapiens GN=SNX9 PE=1 SV=1 - [SNX9_HUMAN]	6,89
Q8N3F8	MICAL-like protein 1 OS=Homo sapiens GN=MICALL1 PE=1 SV=2 - [MILK1_HUMAN]	6,84
Q6ZSS7	Major facilitator superfamily domain-containing protein 6 OS=Homo sapiens GN=MFSD6 PE=1 SV=2 - [MFSD6_HUMAN]	6,83
Q9HB19	Pleckstrin homology domain-containing family A member 2 OS=Homo sapiens GN=PLEKHA2 PE=1 SV=2 - [PKHA2_HUMAN]	6,82
Q9NPH2	Inositol-3-phosphate synthase 1 OS=Homo sapiens GN=ISYNA1 PE=1 SV=1 - [INO1_HUMAN]	6,81
P53350	Serine/threonine-protein kinase PLK1 OS=Homo sapiens GN=PLK1 PE=1 SV=1 - [PLK1_HUMAN]	6,80
Q5T2T1	MAGUK p55 subfamily member 7 OS=Homo sapiens GN=MPP7 PE=1 SV=1 - [MPP7_HUMAN]	6,77
O43837	Isocitrate dehydrogenase [NAD] subunit beta, mitochondrial OS=Homo sapiens GN=IDH3B PE=1 SV=2 - [IDH3B_HUMAN]	6,75
Q13564	NEDD8-activating enzyme E1 regulatory subunit OS=Homo sapiens GN=NAE1 PE=1 SV=1 - [ULA1_HUMAN]	6,74
O60508	Pre-mRNA-processing factor 17 OS=Homo sapiens GN=CDC40 PE=1 SV=1 - [PRP17_HUMAN]	6,74
P78385	Keratin, type II cuticular Hb3 OS=Homo sapiens GN=KRT83 PE=1 SV=2 - [KRT83_HUMAN]	6,69
O43854	EGF-like repeat and discoidin I-like domain-containing protein 3 OS=Homo sapiens GN=EDIL3 PE=1 SV=1 - [EDIL3_HUMAN]	6,67
P23468	Receptor-type tyrosine-protein phosphatase delta OS=Homo sapiens GN=PTPRD PE=1 SV=2 - [PTPRD_HUMAN]	6,64
Q9UBU9	Nuclear RNA export factor 1 OS=Homo sapiens GN=NXF1 PE=1 SV=1 - [NXF1_HUMAN]	6,62
Q96LD4	Tripartite motif-containing protein 47 OS=Homo sapiens GN=TRIM47 PE=1 SV=2 - [TRI47_HUMAN]	6,58
Q9BW91	ADP-ribose pyrophosphatase, mitochondrial OS=Homo sapiens GN=NUDT9 PE=1 SV=1 - [NUDT9_HUMAN]	6,57

O15484	Calpain-5 OS=Homo sapiens GN=CAPN5 PE=1 SV=2 - [CAN5_HUMAN]	6,56	3	[illegible]
P48651	Phosphatidylserine synthase 1 OS=Homo sapiens GN=PTDSS1 PE=1 SV=1 - [PTSS1_HUMAN]	6,55	3	[illegible]
O15234	Protein CASC3 OS=Homo sapiens GN=CASC3 PE=1 SV=2 - [CASC3_HUMAN]	6,54	3	[illegible]
P22570	NADPH:adrenodoxin oxidoreductase, mitochondrial OS=Homo sapiens GN=FDXR PE=1 SV=3 - [ADRO_HUMAN]	6,52	3	[illegible]
Q14738	Serine/threonine-protein phosphatase 2A 56 kDa regulatory subunit delta isoform OS=Homo sapiens GN=PPP2R5D PE=1 SV=1 - [2A5D_H	6,48	3	[illegible]
O00566	U3 small nucleolar ribonucleoprotein protein MPP10 OS=Homo sapiens GN=MPHOSPH10 PE=1 SV=2 - [MPP10_HUMAN]	6,46	3	[illegible]
Q5T0N5	Formin-binding protein 1-like OS=Homo sapiens GN=FNBP1L PE=1 SV=2 - [FBP1L_HUMAN]	6,45	3	[illegible]
O00178	GTP-binding protein 1 OS=Homo sapiens GN=GTPBP1 PE=1 SV=3 - [GTPB1_HUMAN]	6,43	3	[illegible]
Q92581	Sodium/hydrogen exchanger 6 OS=Homo sapiens GN=SLC9A6 PE=1 SV=2 - [SL9A6_HUMAN]	6,43	3	[illegible]
P50542	Peroxisomal targeting signal 1 receptor OS=Homo sapiens GN=PEX5 PE=1 SV=3 - [PEX5_HUMAN]	6,42	3	[illegible]
Q9Y6D9	Mitotic spindle assembly checkpoint protein MAD1 OS=Homo sapiens GN=MAD1L1 PE=1 SV=2 - [MD1L1_HUMAN]	6,41	3	[illegible]
O43194	G-protein coupled receptor 39 OS=Homo sapiens GN=GPR39 PE=1 SV=1 - [GPR39_HUMAN]	6,40	3	[illegible]
Q8N1G4	Leucine-rich repeat-containing protein 47 OS=Homo sapiens GN=LRRC47 PE=1 SV=1 - [LRC47_HUMAN]	6,35	3	[illegible]
Q01581	Hydroxymethylglutaryl-CoA synthase, cytoplasmic OS=Homo sapiens GN=HMGCS1 PE=1 SV=2 - [HMCS1_HUMAN]	6,35	3	[illegible]
Q9NPH3	Interleukin-1 receptor accessory protein OS=Homo sapiens GN=IL1RAP PE=1 SV=2 - [IL1AP_HUMAN]	6,32	3	[illegible]
Q16658	Fascin OS=Homo sapiens GN=FSCN1 PE=1 SV=3 - [FSCN1_HUMAN]	6,29	3	[illegible]
O00541	Pescadillo homolog OS=Homo sapiens GN=PES1 PE=1 SV=1 - [PESC_HUMAN]	6,29	3	[illegible]
Q9BXB5	Oxysterol-binding protein-related protein 10 OS=Homo sapiens GN=OSBPL10 PE=1 SV=2 - [OSB10_HUMAN]	6,28	3	[illegible]
P35611	Alpha-adducin OS=Homo sapiens GN=ADD1 PE=1 SV=2 - [ADDA_HUMAN]	6,24	3	[illegible]
Q99959	Plakophilin-2 OS=Homo sapiens GN=PKP2 PE=1 SV=2 - [PKP2_HUMAN]	6,24	3	[illegible]
Q9BWE0	Replication initiator 1 OS=Homo sapiens GN=REPIN1 PE=1 SV=1 - [REPI1_HUMAN]	6,17	3	[illegible]
P03915	NADH-ubiquinone oxidoreductase chain 5 OS=Homo sapiens GN=MT-ND5 PE=1 SV=2 - [NU5M_HUMAN]	6,14	3	[illegible]
Q8IZ81	ELMO domain-containing protein 2 OS=Homo sapiens GN=ELMOD2 PE=1 SV=1 - [ELMD2_HUMAN]	6,14	3	[illegible]
Q13049	E3 ubiquitin-protein ligase TRIM32 OS=Homo sapiens GN=TRIM32 PE=1 SV=2 - [TRI32_HUMAN]	6,13	3	[illegible]
P04004	Vitronectin OS=Homo sapiens GN=VTN PE=1 SV=1 - [VTNC_HUMAN]	6,07	3	[illegible]
Q2PZI1	Protein dpy-19 homolog 1 OS=Homo sapiens GN=DPY19L1 PE=2 SV=1 - [D19L1_HUMAN]	6,07	3	[illegible]
Q8NAT1	Uncharacterized glycosyltransferase AGO61 OS=Homo sapiens GN=AGO61 PE=2 SV=1 - [AGO61_HUMAN]	6,03	3	[illegible]
Q8NC60	Nitric oxide-associated protein 1 OS=Homo sapiens GN=NOA1 PE=1 SV=2 - [NOA1_HUMAN]	6,02	3	[illegible]
P49757	Protein numb homolog OS=Homo sapiens GN=NUMB PE=1 SV=2 - [NUMB_HUMAN]	5,99	3	[illegible]
P18850	Cyclic AMP-dependent transcription factor ATF-6 alpha OS=Homo sapiens GN=ATF6 PE=1 SV=3 - [ATF6A_HUMAN]	5,97	3	[illegible]
Q14746	Conserved oligomeric Golgi complex subunit 2 OS=Homo sapiens GN=COG2 PE=1 SV=1 - [COG2_HUMAN]	5,96	3	[illegible]
Q9Y5L0	Transportin-3 OS=Homo sapiens GN=TNPO3 PE=1 SV=3 - [TNPO3_HUMAN]	5,96	3	[illegible]
Q8IYQ7	Threonine synthase-like 1 OS=Homo sapiens GN=THNSL1 PE=1 SV=2 - [THNS1_HUMAN]	5,92	3	[illegible]
P02675	Fibrinogen beta chain OS=Homo sapiens GN=FGB PE=1 SV=2 - [FIBB_HUMAN]	5,91	3	[illegible]
P11171	Protein 4.1 OS=Homo sapiens GN=EPB41 PE=1 SV=4 - [41_HUMAN]	5,90	3	[illegible]
Q9Y4W2	Ribosomal biogenesis protein LAS1L OS=Homo sapiens GN=LAS1L PE=1 SV=2 - [LAS1L_HUMAN]	5,86	3	[illegible]
O94923	D-glucuronyl C5-epimerase OS=Homo sapiens GN=GLCE PE=1 SV=3 - [GLCE_HUMAN]	5,83	3	[illegible]
Q09161	Nuclear cap-binding protein subunit 1 OS=Homo sapiens GN=NCBP1 PE=1 SV=1 - [NCBP1_HUMAN]	5,82	3	[illegible]
O15226	NF-kappa-B-repressing factor OS=Homo sapiens GN=NKRF PE=1 SV=2 - [NKRF_HUMAN]	5,80	3	[illegible]
Q8WWI5	Choline transporter-like protein 1 OS=Homo sapiens GN=SLC44A1 PE=1 SV=1 - [CTL1_HUMAN]	5,78	3	[illegible]
Q9UJX3-2	Isoform 2 of Anaphase-promoting complex subunit 7 OS=Homo sapiens GN=ANAPC7 - [APC7_HUMAN]	5,77	3	[illegible]
Q07866	Kinesin light chain 1 OS=Homo sapiens GN=KLC1 PE=1 SV=2 - [KLC1_HUMAN]	5,76	3	[illegible]
Q96PU5	E3 ubiquitin-protein ligase NEDD4-like OS=Homo sapiens GN=NEDD4L PE=1 SV=2 - [NED4L_HUMAN]	5,74	3	[illegible]
Q9UBB9	Tuftelin-interacting protein 11 OS=Homo sapiens GN=TFIP11 PE=1 SV=1 - [TFP11_HUMAN]	5,73	3	[illegible]
Q9UJ83	2-hydroxyacyl-CoA lyase 1 OS=Homo sapiens GN=HACL1 PE=1 SV=2 - [HACL1_HUMAN]	5,71	3	[illegible]
P18887	DNA repair protein XRCC1 OS=Homo sapiens GN=XRCC1 PE=1 SV=2 - [XRCC1_HUMAN]	5,69	3	[illegible]
P03905	NADH-ubiquinone oxidoreductase chain 4 OS=Homo sapiens GN=MT-ND4 PE=1 SV=1 - [NU4M_HUMAN]	5,66	3	[illegible]
Q96CP6	GRAM domain-containing protein 1A OS=Homo sapiens GN=GRAMD1A PE=1 SV=2 - [GRM1A_HUMAN]	5,66	3	[illegible]
P11413	Glucose-6-phosphate 1-dehydrogenase OS=Homo sapiens GN=G6PD PE=1 SV=4 - [G6PD_HUMAN]	5,63	3	[illegible]
Q9Y5P4	Collagen type IV alpha-3-binding protein OS=Homo sapiens GN=COL4A3BP PE=1 SV=1 - [C43BP_HUMAN]	5,61	3	[illegible]
Q6P9F7	Leucine-rich repeat-containing protein 8B OS=Homo sapiens GN=LRRC8B PE=2 SV=2 - [LRC8B_HUMAN]	5,60	3	[illegible]
Q86U42	Polyadenylate-binding protein 2 OS=Homo sapiens GN=PABPN1 PE=1 SV=3 - [PABP2_HUMAN]	5,56	3	[illegible]
Q5LJA0	60 kDa SS-A/Ro ribonucleoprotein OS=Homo sapiens GN=TROVE2 PE=2 SV=1 - [Q5LJA0_HUMAN]	5,52	3	[illegible]
O75509	Tumor necrosis factor receptor superfamily member 21 OS=Homo sapiens GN=TNFRSF21 PE=1 SV=1 - [TNR21_HUMAN]	5,50	3	[illegible]
Q4G0J3	La-related protein 7 OS=Homo sapiens GN=LARP7 PE=1 SV=1 - [LARP7_HUMAN]	5,50	3	[illegible]
Q9Y6A5	Transforming acidic coiled-coil-containing protein 3 OS=Homo sapiens GN=TACC3 PE=1 SV=1 - [TACC3_HUMAN]	5,49	3	[illegible]
Q9BQL6	Fermitin family homolog 1 OS=Homo sapiens GN=FERMT1 PE=1 SV=1 - [FERM1_HUMAN]	5,47	3	[illegible]
Q8WUI4	Histone deacetylase 7 OS=Homo sapiens GN=HDAC7 PE=1 SV=2 - [HDAC7_HUMAN]	5,46	3	[illegible]
Q9NQZ2	Something about silencing protein 10 OS=Homo sapiens GN=UTP3 PE=1 SV=1 - [SAS10_HUMAN]	5,43	3	[illegible]
P51511	Matrix metalloproteinase-15 OS=Homo sapiens GN=MMP15 PE=1 SV=1 - [MMP15_HUMAN]	5,38	3	[illegible]
Q05209	Tyrosine-protein phosphatase non-receptor type 12 OS=Homo sapiens GN=PTPN12 PE=1 SV=3 - [PTN12_HUMAN]	5,38	3	[illegible]
Q5VSL9	Protein FAM40A OS=Homo sapiens GN=FAM40A PE=1 SV=1 - [FA40A_HUMAN]	5,38	3	[illegible]
Q8WX92	Negative elongation factor B OS=Homo sapiens GN=COBRA1 PE=1 SV=1 - [NELFB_HUMAN]	5,34	3	[illegible]
Q9H089	Large subunit GTPase 1 homolog OS=Homo sapiens GN=LSG1 PE=1 SV=2 - [LSG1_HUMAN]	5,32	3	[illegible]
O14975	Very long-chain acyl-CoA synthetase OS=Homo sapiens GN=SLC27A2 PE=1 SV=2 - [S27A2_HUMAN]	5,32	3	[illegible]
Q12765	Secernin-1 OS=Homo sapiens GN=SCRN1 PE=1 SV=2 - [SCRN1_HUMAN]	5,31	3	[illegible]
Q6P3W7	SCY1-like protein 2 OS=Homo sapiens GN=SCYL2 PE=1 SV=1 - [SCYL2_HUMAN]	5,27	3	[illegible]
Q6L8Q7	2',5'-phosphodiesterase 12 OS=Homo sapiens GN=PDE12 PE=1 SV=2 - [PDE12_HUMAN]	5,25	3	[illegible]
Q92922	SWI/SNF complex subunit SMARCC1 OS=Homo sapiens GN=SMARCC1 PE=1 SV=3 - [SMRC1_HUMAN]	5,25	3	[illegible]
O60216	Double-strand-break repair protein rad21 homolog OS=Homo sapiens GN=RAD21 PE=1 SV=2 - [RAD21_HUMAN]	5,23	3	[illegible]
Q2M2I8	AP2-associated protein kinase 1 OS=Homo sapiens GN=AAK1 PE=1 SV=3 - [AAK1_HUMAN]	5,20	3	[illegible]
Q96SB3	Neurabin-2 OS=Homo sapiens GN=PPP1R9B PE=1 SV=2 - [NEB2_HUMAN]	5,15	3	[illegible]
O14678	ATP-binding cassette sub-family D member 4 OS=Homo sapiens GN=ABCD4 PE=2 SV=1 - [ABCD4_HUMAN]	5,12	3	[illegible]
Q9ULJ8	Neurabin-1 OS=Homo sapiens GN=PPP1R9A PE=1 SV=2 - [NEB1_HUMAN]	5,10	3	[illegible]
O15327	Type II inositol-3,4-bisphosphate 4-phosphatase OS=Homo sapiens GN=INPP4B PE=2 SV=4 - [INP4B_HUMAN]	5,09	3	[illegible]
E9PFM7	Poly [ADP-ribose] polymerase 9 OS=Homo sapiens GN=PARP9 PE=4 SV=1 - [E9PFM7_HUMAN]	5,07	3	[illegible]
P21399	Cytoplasmic aconitate hydratase OS=Homo sapiens GN=ACO1 PE=1 SV=3 - [ACOC_HUMAN]	5,06	3	[illegible]
Q9Y210	Short transient receptor potential channel 6 OS=Homo sapiens GN=TRPC6 PE=1 SV=1 - [TRPC6_HUMAN]	5,05	3	[illegible]
Q96SB4	SRSF protein kinase 1 OS=Homo sapiens GN=SRPK1 PE=1 SV=2 - [SRPK1_HUMAN]	5,04	3	[illegible]
P52569	Low affinity cationic amino acid transporter 2 OS=Homo sapiens GN=SLC7A2 PE=1 SV=2 - [CTR2_HUMAN]	5,02	3	[illegible]
Q93063	Exostosin-2 OS=Homo sapiens GN=EXT2 PE=1 SV=1 - [EXT2_HUMAN]	5,01	3	[illegible]
Q9Y262	Eukaryotic translation initiation factor 3 subunit L OS=Homo sapiens GN=EIF3L PE=1 SV=1 - [EIF3L_HUMAN]	4,96	3	[illegible]
A4D1P6	WD repeat-containing protein 91 OS=Homo sapiens GN=WDR91 PE=1 SV=2 - [WDR91_HUMAN]	4,95	3	[illegible]
G3V325	Pentatricopeptide repeat-containing protein 1 OS=Homo sapiens GN=PTCD1 PE=4 SV=1 - [G3V325_HUMAN]	4,94	3	[illegible]
Q9NZV1	Cysteine-rich motor neuron 1 protein OS=Homo sapiens GN=CRIM1 PE=1 SV=1 - [CRIM1_HUMAN]	4,92	3	[illegible]
Q96FV9	THO complex subunit 1 OS=Homo sapiens GN=THOC1 PE=1 SV=1 - [THOC1_HUMAN]	4,87	3	[illegible]
Q15003	Condensin complex subunit 2 OS=Homo sapiens GN=NCAPH PE=1 SV=3 - [CND2_HUMAN]	4,86	3	[illegible]
Q01970	1-phosphatidylinositol-4,5-bisphosphate phosphodiesterase beta-3 OS=Homo sapiens GN=PLCB3 PE=1 SV=2 - [PLCB3_HUMAN]	4,86	3	[illegible]
Q5VUB5	Protein FAM171A1 OS=Homo sapiens GN=FAM171A1 PE=1 SV=1 - [F1711_HUMAN]	4,83	3	[illegible]
O00339	Matrilin-2 OS=Homo sapiens GN=MATN2 PE=1 SV=4 - [MATN2_HUMAN]	4,81	3	[illegible]
Q99523	Sortilin OS=Homo sapiens GN=SORT1 PE=1 SV=3 - [SORT_HUMAN]	4,81	3	[illegible]
Q9Y6W3	Calpain-7 OS=Homo sapiens GN=CAPN7 PE=1 SV=1 - [CAN7_HUMAN]	4,80	3	[illegible]
E9PSI1	Transmembrane 9 superfamily member 1 OS=Homo sapiens GN=TM9SF1 PE=4 SV=1 - [E9PSI1_HUMAN]	4,79	3	[illegible]
Q8IUH5	Palmitoyltransferase ZDHHC17 OS=Homo sapiens GN=ZDHHC17 PE=1 SV=2 - [ZDH17_HUMAN]	4,75	3	[illegible]
P53794	Sodium/myo-inositol cotransporter OS=Homo sapiens GN=SLC5A3 PE=2 SV=2 - [SC5A3_HUMAN]	4,74	3	[illegible]
Q8TEX9	Importin-4 OS=Homo sapiens GN=IPO4 PE=1 SV=2 - [IPO4_HUMAN]	4,72	3	[illegible]
Q96P70	Importin-9 OS=Homo sapiens GN=IPO9 PE=1 SV=3 - [IPO9_HUMAN]	4,71	3	[illegible]
Q9Y6A1	Protein O-mannosyl-transferase 1 OS=Homo sapiens GN=POMT1 PE=1 SV=3 - [POMT1_HUMAN]	4,69	3	[illegible]
Q7Z7M9	Polypeptide N-acetylgalactosaminyltransferase 5 OS=Homo sapiens GN=GALNT5 PE=1 SV=1 - [GALT5_HUMAN]	4,68	3	[illegible]
Q8IXB1	DnaJ homolog subfamily C member 10 OS=Homo sapiens GN=DNAJC10 PE=1 SV=2 - [DJC10_HUMAN]	4,67	3	[illegible]
Q5T0F9	Coiled-coil and C2 domain-containing protein 1B OS=Homo sapiens GN=CC2D1B PE=1 SV=1 - [C2D1B_HUMAN]	4,66	3	[illegible]
Q14703	Membrane-bound transcription factor site-1 protease OS=Homo sapiens GN=MBTPS1 PE=1 SV=1 - [MBTP1_HUMAN]	4,66	3	[illegible]
Q9UQR1	Zinc finger protein 148 OS=Homo sapiens GN=ZNF148 PE=1 SV=2 - [ZN148_HUMAN]	4,66	3	[illegible]
Q8TED0	U3 small nucleolar RNA-associated protein 15 homolog OS=Homo sapiens GN=UTP15 PE=1 SV=3 - [UTP15_HUMAN]	4,63	3	[illegible]
Q86X55	Histone-arginine methyltransferase CARM1 OS=Homo sapiens GN=CARM1 PE=1 SV=3 - [CARM1_HUMAN]	4,61	3	[illegible]
Q96JG6	Coiled-coil domain-containing protein 132 OS=Homo sapiens GN=CCDC132 PE=1 SV=3 - [CC132_HUMAN]	4,56	3	[illegible]
O60563	Cyclin-T1 OS=Homo sapiens GN=CCNT1 PE=1 SV=1 - [CCNT1_HUMAN]	4,55	3	[illegible]
Q9Y672	Dolichyl pyrophosphate Man9GlcNAc2 alpha-1,3-glucosyltransferase OS=Homo sapiens GN=ALG6 PE=1 SV=1 - [ALG6_HUMAN]	4,54	3	[illegible]
Q68D10	Protein SPT2 homolog OS=Homo sapiens GN=SPTY2D1 PE=1 SV=3 - [SPT2_HUMAN]	4,53	3	[illegible]
Q9BXB1	Leucine-rich repeat-containing G-protein coupled receptor 4 OS=Homo sapiens GN=LGR4 PE=2 SV=2 - [LGR4_HUMAN]	4,52	3	[illegible]
Q9BR76	Coronin-1B OS=Homo sapiens GN=CORO1B PE=1 SV=1 - [COR1B_HUMAN]	4,50	3	[illegible]
Q9P1Y5	Calmodulin-regulated spectrin-associated protein 3 OS=Homo sapiens GN=CAMSAP3 PE=1 SV=2 - [CAMP3_HUMAN]	4,48	3	[illegible]
Q9BUK6	Protein misato homolog 1 OS=Homo sapiens GN=MSTO1 PE=1 SV=1 - [MSTO1_HUMAN]	4,39	3	[illegible]
Q5JSH3	WD repeat-containing protein 44 OS=Homo sapiens GN=WDR44 PE=1 SV=1 - [WDR44_HUMAN]	4,38	3	[illegible]
Q68CQ4	Digestive organ expansion factor homolog OS=Homo sapiens GN=DIEXF PE=1 SV=2 - [DIEXF_HUMAN]	4,37	3	[illegible]
Q9P2N5	RNA-binding protein 27 OS=Homo sapiens GN=RBM27 PE=1 SV=2 - [RBM27_HUMAN]	4,25	3	[illegible]
Q8IWZ3	Ankyrin repeat and KH domain-containing protein 1 OS=Homo sapiens GN=ANKHD1 PE=1 SV=1 - [ANKH1_HUMAN]	4,21	3	[illegible]
Q7Z6B7	SLIT-ROBO Rho GTPase-activating protein 1 OS=Homo sapiens GN=SRGAP1 PE=1 SV=1 - [SRGP1_HUMAN]	4,15	3	[illegible]
Q13823	Nucleolar GTP-binding protein 2 OS=Homo sapiens GN=GNL2 PE=1 SV=1 - [NOG2_HUMAN]	4,10	3	[illegible]
Q08379	Golgin subfamily A member 2 OS=Homo sapiens GN=GOLGA2 PE=1 SV=3 - [GOGA2_HUMAN]	4,09	3	[illegible]
Q5H9R7	Serine/threonine-protein phosphatase 6 regulatory subunit 3 OS=Homo sapiens GN=PPP6R3 PE=1 SV=2 - [PP6R3_HUMAN]	4,01	3	[illegible]
Q8TD16	Protein bicaudal D homolog 2 OS=Homo sapiens GN=BICD2 PE=1 SV=1 - [BICD2_HUMAN]	4,00	3	[illegible]
O60341	Lysine-specific histone demethylase 1A OS=Homo sapiens GN=KDM1A PE=1 SV=2 - [KDM1A_HUMAN]	3,99	3	[illegible]
O14976	Cyclin-G-associated kinase OS=Homo sapiens GN=GAK PE=1 SV=2 - [GAK_HUMAN]	3,97	3	[illegible]
O60343	TBC1 domain family member 4 OS=Homo sapiens GN=TBC1D4 PE=1 SV=2 - [TBCD4_HUMAN]	3,93	3	[illegible]
Q9UKV5	E3 ubiquitin-protein ligase AMFR OS=Homo sapiens GN=AMFR PE=1 SV=2 - [AMFR2_HUMAN]	3,89	3	[illegible]
O43520	Probable phospholipid-transporting ATPase IC OS=Homo sapiens GN=ATP8B1 PE=1 SV=3 - [AT8B1_HUMAN]	3,84	3	[illegible]
Q9Y4R8	Telomere length regulation protein TEL2 homolog OS=Homo sapiens GN=TELO2 PE=1 SV=2 - [TELO2_HUMAN]	3,82	3	[illegible]
P23378	Glycine dehydrogenase [decarboxylating], mitochondrial OS=Homo sapiens GN=GLDC PE=1 SV=2 - [GCSP_HUMAN]	3,82	3	[illegible]
P40818	Ubiquitin carboxyl-terminal hydrolase 8 OS=Homo sapiens GN=USP8 PE=1 SV=1 - [UBP8_HUMAN]	3,76	3	[illegible]
Q9H694	Protein bicaudal C homolog 1 OS=Homo sapiens GN=BICC1 PE=1 SV=2 - [BICC1_HUMAN]	3,70	3	[illegible]
O75116	Rho-associated protein kinase 2 OS=Homo sapiens GN=ROCK2 PE=1 SV=4 - [ROCK2_HUMAN]	3,67	3	[illegible]
Q6P4R8	Nuclear factor related to kappa-B-binding protein OS=Homo sapiens GN=NFRKB PE=1 SV=2 - [NFRKB_HUMAN]	3,62	3	[illegible]
P35568	Insulin receptor substrate 1 OS=Homo sapiens GN=IRS1 PE=1 SV=1 - [IRS1_HUMAN]	3,62	3	[illegible]
Q9NP73	UDP-N-acetylglucosamine transferase subunit ALG13 homolog OS=Homo sapiens GN=ALG13 PE=1 SV=2 - [ALG13_HUMAN]	3,61	3	[illegible]
Q9Y2G3	Probable phospholipid-transporting ATPase IF OS=Homo sapiens GN=ATP11B PE=1 SV=2 - [AT11B_HUMAN]	3,57	3	[illegible]
Q6NUQ1	RAD50-interacting protein 1 OS=Homo sapiens GN=RINT1 PE=1 SV=1 - [RINT1_HUMAN]	3,54	3	[illegible]
O94886	Transmembrane protein 63A OS=Homo sapiens GN=TMEM63A PE=1 SV=3 - [TM63A_HUMAN]	3,47	3	[illegible]
Q7Z5K2	Wings apart-like protein homolog OS=Homo sapiens GN=WAPAL PE=1 SV=1 - [WAPL_HUMAN]	3,45	3	[illegible]
Q9P0V3	SH3 domain-binding protein 4 OS=Homo sapiens GN=SH3BP4 PE=1 SV=1 - [SH3B4_HUMAN]	3,43	3	[illegible]
Q9H7N4	Splicing factor, arginine/serine-rich 19 OS=Homo sapiens GN=SCAF1 PE=1 SV=3 - [SFR19_HUMAN]	3,43	3	[illegible]
P46934	E3 ubiquitin-protein ligase NEDD4 OS=Homo sapiens GN=NEDD4 PE=1 SV=4 - [NEDD4_HUMAN]	3,41	3	[illegible]
Q13616	Cullin-1 OS=Homo sapiens GN=CUL1 PE=1 SV=2 - [CUL1_HUMAN]	3,35	3	[illegible]
O15440	Multidrug resistance-associated protein 5 OS=Homo sapiens GN=ABCC5 PE=1 SV=2 - [MRP5_HUMAN]	3,34	3	[illegible]
O60503	Adenylate cyclase type 9 OS=Homo sapiens GN=ADCY9 PE=1 SV=4 - [ADCY9_HUMAN]	3,33	3	[illegible]
O95487	Protein transport protein Sec24B OS=Homo sapiens GN=SEC24B PE=1 SV=2 - [SC24B_HUMAN]	3,31	3	[illegible]
Q9UMZ2	Synergin gamma OS=Homo sapiens GN=SYNRG PE=1 SV=2 - [SYNRG_HUMAN]	3,27	3	[illegible]
Q86TB9	Protein PAT1 homolog 1 OS=Homo sapiens GN=PATL1 PE=1 SV=2 - [PATL1_HUMAN]	3,25	3	[illegible]
Q9NZJ5	Eukaryotic translation initiation factor 2-alpha kinase 3 OS=Homo sapiens GN=EIF2AK3 PE=1 SV=3 - [E2AK3_HUMAN]	3,23	3	[illegible]
Q14997	Proteasome activator complex subunit 4 OS=Homo sapiens GN=PSME4 PE=1 SV=2 - [PSME4_HUMAN]	3,20	3	[illegible]
Q8IX01-4	Isoform 4 of SURP and G-patch domain-containing protein 2 OS=Homo sapiens GN=SUGP2 - [SUGP2_HUMAN]	3,20	3	[illegible]
P15941	Mucin-1 OS=Homo sapiens GN=MUC1 PE=1 SV=3 - [MUC1_HUMAN]	3,19	3	[illegible]
Q9BPX3	Condensin complex subunit 3 OS=Homo sapiens GN=NCAPG PE=1 SV=1 - [CND3_HUMAN]	3,15	3	[illegible]
Q14671	Pumilio homolog 1 OS=Homo sapiens GN=PUM1 PE=1 SV=3 - [PUM1_HUMAN]	3,12	3	[illegible]
O95104	Splicing factor, arginine/serine-rich 15 OS=Homo sapiens GN=SCAF4 PE=1 SV=3 - [SFR15_HUMAN]	3,05	3	[illegible]
O60306	Intron-binding protein aquarius OS=Homo sapiens GN=AQR PE=1 SV=4 - [AQR_HUMAN]	3,03	3	[illegible]
P52701	DNA mismatch repair protein Msh6 OS=Homo sapiens GN=MSH6 PE=1 SV=2 - [MSH6_HUMAN]	3,01	3	[illegible]
Q9Y3P9	Rab GTPase-activating protein 1 OS=Homo sapiens GN=RABGAP1 PE=1 SV=3 - [RBGP1_HUMAN]	2,99	3	[illegible]
O60337	E3 ubiquitin-protein ligase MARCH6 OS=Homo sapiens GN=MARCH6 PE=1 SV=2 - [MARH6_HUMAN]	2,97	3	[illegible]
Q68CR1	Protein sel-1 homolog 3 OS=Homo sapiens GN=SEL1L3 PE=1 SV=2 - [SE1L3_HUMAN]	2,92	3	[illegible]
Q9UNS1	Protein timeless homolog OS=Homo sapiens GN=TIMELESS PE=1 SV=2 - [TIM_HUMAN]	2,90	3	[illegible]
I3L2J0	Protein capicua homolog OS=Homo sapiens GN=CIC PE=4 SV=1 - [I3L2J0_HUMAN]	2,82	3	[illegible]
Q8IWK6	Probable G-protein coupled receptor 125 OS=Homo sapiens GN=GPR125 PE=1 SV=2 - [GP125_HUMAN]	2,80	3	[illegible]
Q15020	Squamous cell carcinoma antigen recognized by T-cells 3 OS=Homo sapiens GN=SART3 PE=1 SV=1 - [SART3_HUMAN]	2,80	3	[illegible]
P39060	Collagen alpha-1(XVIII) chain OS=Homo sapiens GN=COL18A1 PE=1 SV=5 - [COIA1_HUMAN]	2,74	3	[illegible]
Q86SQ4	G-protein coupled receptor 126 OS=Homo sapiens GN=GPR126 PE=1 SV=3 - [GP126_HUMAN]	2,70	3	[illegible]
P41229	Lysine-specific demethylase 5C OS=Homo sapiens GN=KDM5C PE=1 SV=2 - [KDM5C_HUMAN]	2,69	3	[illegible]
O60437	Periplakin OS=Homo sapiens GN=PPL PE=1 SV=4 - [PEPL_HUMAN]	2,68	3	[illegible]
Q14241	Transcription elongation factor B polypeptide 3 OS=Homo sapiens GN=TCEB3 PE=1 SV=2 - [ELOA1_HUMAN]	2,63	3	[illegible]
O75376	Nuclear receptor corepressor 1 OS=Homo sapiens GN=NCOR1 PE=1 SV=2 - [NCOR1_HUMAN]	2,46	3	[illegible]

Q9H582	Zinc finger protein 644 OS=Homo sapiens GN=ZNF644 PE=1 SV=2 - [ZN644_HUMAN]	2,41	3
P46531	Neurogenic locus notch homolog protein 1 OS=Homo sapiens GN=NOTCH1 PE=1 SV=4 - [NOTC1_HUMAN]	2,39	3
Q69YN4	Protein virilizer homolog OS=Homo sapiens GN=KIAA1429 PE=1 SV=2 - [VIR_HUMAN]	2,37	3
Q9UMD9	Collagen alpha-1(XVII) chain OS=Homo sapiens GN=COL17A1 PE=1 SV=3 - [COHA1_HUMAN]	2,34	3
Q9Y4D7-2	Isoform 2 of Plexin-D1 OS=Homo sapiens GN=PLXND1 - [PLXD1_HUMAN]	2,24	3
Q8WWQ0	PH-interacting protein OS=Homo sapiens GN=PHIP PE=1 SV=2 - [PHIP_HUMAN]	2,14	3
Q8IY21	Probable ATP-dependent RNA helicase DDX60 OS=Homo sapiens GN=DDX60 PE=2 SV=3 - [DDX60_HUMAN]	2,10	3
Q8IWJ2	GRIP and coiled-coil domain-containing protein 2 OS=Homo sapiens GN=GCC2 PE=1 SV=4 - [GCC2_HUMAN]	2,08	3
Q14669	Probable E3 ubiquitin-protein ligase TRIP12 OS=Homo sapiens GN=TRIP12 PE=1 SV=1 - [TRIPC_HUMAN]	2,01	3
Q96HP0	Dedicator of cytokinesis protein 6 OS=Homo sapiens GN=DOCK6 PE=1 SV=3 - [DOCK6_HUMAN]	1,91	3
Q14146	Unhealthy ribosome biogenesis protein 2 homolog OS=Homo sapiens GN=URB2 PE=1 SV=2 - [URB2_HUMAN]	1,90	3
Q9UK61	Protein FAM208A OS=Homo sapiens GN=FAM208A PE=1 SV=3 - [F208A_HUMAN]	1,80	3
Q14667	UPF0378 protein KIAA0100 OS=Homo sapiens GN=KIAA0100 PE=1 SV=3 - [K0100_HUMAN]	1,79	3
Q9H9B1	Histone-lysine N-methyltransferase EHMT1 OS=Homo sapiens GN=EHMT1 PE=1 SV=4 - [EHMT1_HUMAN]	1,77	3
P42694	Probable helicase with zinc finger domain OS=Homo sapiens GN=HELZ PE=1 SV=2 - [HELZ_HUMAN]	1,70	3
O75165	DnaJ homolog subfamily C member 13 OS=Homo sapiens GN=DNAJC13 PE=1 SV=5 - [DJC13_HUMAN]	1,69	3
O95477	ATP-binding cassette sub-family A member 1 OS=Homo sapiens GN=ABCA1 PE=1 SV=3 - [ABCA1_HUMAN]	1,68	3
Q6ZRS2	Helicase SRCAP OS=Homo sapiens GN=SRCAP PE=1 SV=3 - [SRCAP_HUMAN]	1,55	3
Q9H6A9	Pecanex-like protein 3 OS=Homo sapiens GN=PCNXL3 PE=1 SV=2 - [PCX3_HUMAN]	1,52	3
P12259	Coagulation factor V OS=Homo sapiens GN=F5 PE=1 SV=4 - [FA5_HUMAN]	1,39	3
Q6KC79	Nipped-B-like protein OS=Homo sapiens GN=NIPBL PE=1 SV=2 - [NIPBL_HUMAN]	1,28	3
Q8IWI9	MAX gene-associated protein OS=Homo sapiens GN=MGA PE=1 SV=2 - [MGAP_HUMAN]	1,19	3
O14686	Histone-lysine N-methyltransferase MLL2 OS=Homo sapiens GN=MLL2 PE=1 SV=2 - [MLL2_HUMAN]	1,12	3
P98160	Basement membrane-specific heparan sulfate proteoglycan core protein OS=Homo sapiens GN=HSPG2 PE=1 SV=4 - [PGBM_HUMAN]	1,07	3
Q9NR09	Baculoviral IAP repeat-containing protein 6 OS=Homo sapiens GN=BIRC6 PE=1 SV=2 - [BIRC6_HUMAN]	1,07	3
Q8NFC6	Biorientation of chromosomes in cell division protein 1-like OS=Homo sapiens GN=BOD1L PE=1 SV=2 - [BOD1L_HUMAN]	0,82	3
F5H265	Ubiquitin (Fragment) OS=Homo sapiens GN=UBC PE=4 SV=1 - [F5H265_HUMAN]	89,93	2
P22392-2	Isoform 3 of Nucleoside diphosphate kinase B OS=Homo sapiens GN=NME2 - [NDKB_HUMAN]	71,54	2
E5RGW4	Nucleophosmin (Fragment) OS=Homo sapiens GN=NPM1 PE=4 SV=1 - [E5RGW4_HUMAN]	59,32	2
Q15907	Ras-related protein Rab-11B OS=Homo sapiens GN=RAB11B PE=1 SV=4 - [RB11B_HUMAN]	53,67	2
P01116	GTPase KRas OS=Homo sapiens GN=KRAS PE=1 SV=1 - [RASK_HUMAN]	52,38	2
P62491	Ras-related protein Rab-11A OS=Homo sapiens GN=RAB11A PE=1 SV=3 - [RB11A_HUMAN]	52,31	2
P61960	Ubiquitin-fold modifier 1 OS=Homo sapiens GN=UFM1 PE=1 SV=1 - [UFM1_HUMAN]	50,59	2
Q5VU66	Tropomyosin 3 OS=Homo sapiens GN=TPM3 PE=2 SV=1 - [Q5VU66_HUMAN]	49,60	2
P27816	Microtubule-associated protein 4 OS=Homo sapiens GN=MAP4 PE=1 SV=3 - [MAP4_HUMAN]	48,35	2
Q8IUE6	Histone H2A type 2-B OS=Homo sapiens GN=HIST2H2AB PE=1 SV=3 - [H2A2B_HUMAN]	44,62	2
P19105	Myosin regulatory light chain 12A OS=Homo sapiens GN=MYL12A PE=1 SV=2 - [ML12A_HUMAN]	44,44	2
P27635	60S ribosomal protein L10 OS=Homo sapiens GN=RPL10 PE=1 SV=4 - [RL10_HUMAN]	44,39	2
E5RGN3	Copper transport protein ATOX1 OS=Homo sapiens GN=ATOX1 PE=4 SV=1 - [E5RGN3_HUMAN]	44,07	2
P17096-2	Isoform HMG-Y of High mobility group protein HMG-I/HMG-Y OS=Homo sapiens GN=HMGA1 - [HMGA1_HUMAN]	43,75	2
E9PHY5	Band 4.1-like protein 2 OS=Homo sapiens GN=EPB41L2 PE=4 SV=1 - [E9PHY5_HUMAN]	42,67	2
P61254	60S ribosomal protein L26 OS=Homo sapiens GN=RPL26 PE=1 SV=1 - [RL26_HUMAN]	41,38	2
Q12906-4	Isoform 4 of Interleukin enhancer-binding factor 3 OS=Homo sapiens GN=ILF3 - [ILF3_HUMAN]	40,97	2
P16104	Histone H2A.x OS=Homo sapiens GN=H2AFX PE=1 SV=2 - [H2AX_HUMAN]	40,56	2
P01893	Putative HLA class I histocompatibility antigen, alpha chain H OS=Homo sapiens GN=HLA-H PE=5 SV=3 - [HLAH_HUMAN]	37,02	2
O95167	NADH dehydrogenase [ubiquinone] 1 alpha subcomplex subunit 3 OS=Homo sapiens GN=NDUFA3 PE=1 SV=1 - [NDUA3_HUMAN]	36,90	2
Q9NYJ1	Coiled-coil-helix-coiled-coil-helix domain-containing protein 8 OS=Homo sapiens GN=CHCHD8 PE=1 SV=2 - [CHCH8_HUMAN]	36,78	2
Q92930	Ras-related protein Rab-8B OS=Homo sapiens GN=RAB8B PE=1 SV=2 - [RAB8B_HUMAN]	36,71	2
Q13509	Tubulin beta-3 chain OS=Homo sapiens GN=TUBB3 PE=1 SV=2 - [TBB3_HUMAN]	36,44	2
P62244	40S ribosomal protein S15a OS=Homo sapiens GN=RPS15A PE=1 SV=2 - [RS15A_HUMAN]	36,15	2
H0Y9C2	Calcium/calmodulin-dependent protein kinase type II subunit delta (Fragment) OS=Homo sapiens GN=CAMK2D PE=4 SV=1 - [H0Y9C2_H	35,57	2
O43715	TP53-regulated inhibitor of apoptosis 1 OS=Homo sapiens GN=TRIAP1 PE=1 SV=1 - [TRIA1_HUMAN]	35,53	2
Q8WVX3	Uncharacterized protein C4orf3 OS=Homo sapiens GN=C4orf3 PE=2 SV=2 - [CD003_HUMAN]	34,85	2
O43852-2	Isoform 2 of Calumenin OS=Homo sapiens GN=CALU - [CALU_HUMAN]	34,60	2
O75438	NADH dehydrogenase [ubiquinone] 1 beta subcomplex subunit 1 OS=Homo sapiens GN=NDUFB1 PE=1 SV=1 - [NDUB1_HUMAN]	34,48	2
P61204	ADP-ribosylation factor 3 OS=Homo sapiens GN=ARF3 PE=1 SV=2 - [ARF3_HUMAN]	33,70	2
Q9BSJ8	Extended synaptotagmin-1 OS=Homo sapiens GN=ESYT1 PE=1 SV=1 - [ESYT1_HUMAN]	33,61	2
Q14697-2	Isoform 2 of Neutral alpha-glucosidase AB OS=Homo sapiens GN=GANAB - [GANAB_HUMAN]	32,92	2
P02795	Metallothionein-2 OS=Homo sapiens GN=MT2A PE=1 SV=1 - [MT2_HUMAN]	32,79	2
Q8NHG7	Small VCP/p97-interacting protein OS=Homo sapiens GN=SVIP PE=2 SV=1 - [SVIP_HUMAN]	32,47	2
H3BM67	Nucleolar protein 3 (Fragment) OS=Homo sapiens GN=NOL3 PE=4 SV=1 - [H3BM67_HUMAN]	32,37	2
Q13541	Eukaryotic translation initiation factor 4E-binding protein 1 OS=Homo sapiens GN=EIF4EBP1 PE=1 SV=3 - [4EBP1_HUMAN]	32,20	2
Q8WUW1	Protein BRICK1 OS=Homo sapiens GN=BRK1 PE=1 SV=1 - [BRK1_HUMAN]	32,00	2
Q12906	Interleukin enhancer-binding factor 3 OS=Homo sapiens GN=ILF3 PE=1 SV=3 - [ILF3_HUMAN]	31,77	2
P60602	Reactive oxygen species modulator 1 OS=Homo sapiens GN=ROMO1 PE=1 SV=1 - [ROMO1_HUMAN]	31,65	2
P0C0S5	Histone H2A.Z OS=Homo sapiens GN=H2AFZ PE=1 SV=2 - [H2AZ_HUMAN]	31,25	2
Q15149	Plectin OS=Homo sapiens GN=PLEC PE=1 SV=3 - [PLEC_HUMAN]	31,23	2
D6RBG4	Signal recognition particle 19 kDa protein OS=Homo sapiens GN=SRP19 PE=4 SV=1 - [D6RBG4_HUMAN]	30,23	2
Q8WUH6	UPF0444 transmembrane protein C12orf23 OS=Homo sapiens GN=C12orf23 PE=1 SV=1 - [CL023_HUMAN]	30,17	2
Q00839-2	Isoform Short of Heterogeneous nuclear ribonucleoprotein U OS=Homo sapiens GN=HNRNPU - [HNRPU_HUMAN]	30,15	2
P82921	28S ribosomal protein S21, mitochondrial OS=Homo sapiens GN=MRPS21 PE=1 SV=2 - [RT21_HUMAN]	29,89	2
Q9NP97	Dynein light chain roadblock-type 1 OS=Homo sapiens GN=DYNLRB1 PE=1 SV=3 - [DLRB1_HUMAN]	29,17	2
P53396	ATP-citrate synthase OS=Homo sapiens GN=ACLY PE=1 SV=3 - [ACLY_HUMAN]	29,16	2
P62136	Serine/threonine-protein phosphatase PP1-alpha catalytic subunit OS=Homo sapiens GN=PPP1CA PE=1 SV=1 - [PP1A_HUMAN]	29,09	2
P53396-2	Isoform 2 of ATP-citrate synthase OS=Homo sapiens GN=ACLY - [ACLY_HUMAN]	28,96	2
P14406	Cytochrome c oxidase subunit 7A2, mitochondrial OS=Homo sapiens GN=COX7A2 PE=1 SV=1 - [CX7A2_HUMAN]	27,71	2
P56378	6.8 kDa mitochondrial proteolipid OS=Homo sapiens GN=MP68 PE=1 SV=1 - [68MP_HUMAN]	27,59	2
Q5JTV8	Torsin-1A-interacting protein 1 OS=Homo sapiens GN=TOR1AIP1 PE=1 SV=2 - [TOIP1_HUMAN]	27,44	2
P36873	Serine/threonine-protein phosphatase PP1-gamma catalytic subunit OS=Homo sapiens GN=PPP1CC PE=1 SV=1 - [PP1G_HUMAN]	27,24	2
P62140	Serine/threonine-protein phosphatase PP1-beta catalytic subunit OS=Homo sapiens GN=PPP1CB PE=1 SV=3 - [PP1B_HUMAN]	27,22	2
Q6ZQX7	Uncharacterized protein C17orf97 OS=Homo sapiens GN=C17orf97 PE=2 SV=2 - [CQ097_HUMAN]	26,49	2
P63173	60S ribosomal protein L38 OS=Homo sapiens GN=RPL38 PE=1 SV=2 - [RL38_HUMAN]	25,71	2
Q9NR31	GTP-binding protein SAR1a OS=Homo sapiens GN=SAR1A PE=1 SV=1 - [SAR1A_HUMAN]	25,25	2
Q9NYM9	BET1-like protein OS=Homo sapiens GN=BET1L PE=1 SV=1 - [BET1L_HUMAN]	25,23	2
Q8N4V1	Membrane magnesium transporter 1 OS=Homo sapiens GN=MMGT1 PE=1 SV=1 - [MMGT1_HUMAN]	25,19	2
P62273	40S ribosomal protein S29 OS=Homo sapiens GN=RPS29 PE=1 SV=2 - [RS29_HUMAN]	25,00	2
Q9BZL1	Ubiquitin-like protein 5 OS=Homo sapiens GN=UBL5 PE=1 SV=1 - [UBL5_HUMAN]	24,66	2
P10114	Ras-related protein Rap-2a OS=Homo sapiens GN=RAP2A PE=1 SV=1 - [RAP2A_HUMAN]	24,59	2
O15155	BET1 homolog OS=Homo sapiens GN=BET1 PE=1 SV=1 - [BET1_HUMAN]	24,58	2
P04080	Cystatin-B OS=Homo sapiens GN=CSTB PE=1 SV=2 - [CYTB_HUMAN]	24,49	2
Q9Y6B6	GTP-binding protein SAR1b OS=Homo sapiens GN=SAR1B PE=1 SV=1 - [SAR1B_HUMAN]	24,24	2
Q9BQ48	39S ribosomal protein L34, mitochondrial OS=Homo sapiens GN=MRPL34 PE=1 SV=1 - [RM34_HUMAN]	23,91	2
Q9P2X0	Dolichol-phosphate mannosyltransferase subunit 3 OS=Homo sapiens GN=DPM3 PE=1 SV=2 - [DPM3_HUMAN]	23,91	2
P42677	40S ribosomal protein S27 OS=Homo sapiens GN=RPS27 PE=1 SV=3 - [RS27_HUMAN]	23,81	2
P62318	Small nuclear ribonucleoprotein Sm D3 OS=Homo sapiens GN=SNRPD3 PE=1 SV=1 - [SMD3_HUMAN]	23,81	2
Q9Y3U8	60S ribosomal protein L36 OS=Homo sapiens GN=RPL36 PE=1 SV=3 - [RL36_HUMAN]	23,81	2
P62312	U6 snRNA-associated Sm-like protein LSm6 OS=Homo sapiens GN=LSM6 PE=1 SV=1 - [LSM6_HUMAN]	23,75	2
Q92692-2	Isoform Alpha of Poliovirus receptor-related protein 2 OS=Homo sapiens GN=PVRL2 - [PVRL2_HUMAN]	23,59	2
Q9Y5J6	Mitochondrial import inner membrane translocase subunit Tim9 B OS=Homo sapiens GN=FXC1 PE=1 SV=1 - [TIM9B_HUMAN]	23,30	2
Q6EEV6	Small ubiquitin-related modifier 4 OS=Homo sapiens GN=SUMO4 PE=1 SV=2 - [SUMO4_HUMAN]	23,16	2
Q13948	Protein CASP OS=Homo sapiens GN=CUX1 PE=1 SV=2 - [CASP_HUMAN]	23,01	2
Q6NZ67	Mitotic-spindle organizing protein 2B OS=Homo sapiens GN=MZT2B PE=1 SV=1 - [MZT2B_HUMAN]	22,78	2
Q8N490-2	Isoform 2 of Probable hydrolase PNKD OS=Homo sapiens GN=PNKD - [PNKD_HUMAN]	22,54	2
Q9Y2R5	28S ribosomal protein S17, mitochondrial OS=Homo sapiens GN=MRPS17 PE=1 SV=1 - [RT17_HUMAN]	22,31	2
Q8TCT9	Minor histocompatibility antigen H13 OS=Homo sapiens GN=HM13 PE=1 SV=1 - [HM13_HUMAN]	22,28	2
P58546	Myotrophin OS=Homo sapiens GN=MTPN PE=1 SV=2 - [MTPN_HUMAN]	22,03	2
B7Z6R0	Phosphomannomutase 2 OS=Homo sapiens GN=PMM2 PE=4 SV=1 - [B7Z6R0_HUMAN]	21,85	2
Q9BUE6	Iron-sulfur cluster assembly 1 homolog, mitochondrial OS=Homo sapiens GN=ISCA1 PE=2 SV=1 - [ISCA1_HUMAN]	21,71	2
Q9P0B6	Coiled-coil domain-containing protein 167 OS=Homo sapiens GN=CCDC167 PE=2 SV=2 - [CC167_HUMAN]	21,65	2
Q5JTJ3	Uncharacterized protein C1orf31 OS=Homo sapiens GN=C1orf31 PE=2 SV=1 - [CA031_HUMAN]	21,60	2
Q9UKY7	Protein CDV3 homolog OS=Homo sapiens GN=CDV3 PE=1 SV=1 - [CDV3_HUMAN]	21,32	2
Q9Y6D0	Selenoprotein K OS=Homo sapiens GN=SELK PE=1 SV=3 - [SELK_HUMAN]	21,28	2
E9PLD3	Uncharacterized protein OS=Homo sapiens PE=4 SV=1 - [E9PLD3_HUMAN]	21,21	2
F8WD51	Uridine phosphorylase 1 OS=Homo sapiens GN=UPP1 PE=4 SV=1 - [F8WD51_HUMAN]	21,18	2
P14649	Myosin light chain 6B OS=Homo sapiens GN=MYL6B PE=1 SV=1 - [MYL6B_HUMAN]	21,15	2
Q96A57	UPF0414 transmembrane protein C20orf30 OS=Homo sapiens GN=C20orf30 PE=1 SV=1 - [CT030_HUMAN]	20,83	2
H0YIV9	Glutamyl-tRNA(Gln) amidotransferase subunit C, mitochondrial (Fragment) OS=Homo sapiens GN=GATC PE=3 SV=1 - [H0YIV9_HUMAN	20,83	2
P09104	Gamma-enolase OS=Homo sapiens GN=ENO2 PE=1 SV=3 - [ENOG_HUMAN]	20,74	2
Q92692	Poliovirus receptor-related protein 2 OS=Homo sapiens GN=PVRL2 PE=1 SV=1 - [PVRL2_HUMAN]	20,45	2
Q96E39	RNA binding motif protein, X-linked-like-1 OS=Homo sapiens GN=RBMXL1 PE=1 SV=1 - [RMXL1_HUMAN]	20,26	2
P61024	Cyclin-dependent kinases regulatory subunit 1 OS=Homo sapiens GN=CKS1B PE=1 SV=1 - [CKS1_HUMAN]	20,25	2
P63167	Dynein light chain 1, cytoplasmic OS=Homo sapiens GN=DYNLL1 PE=1 SV=1 - [DYL1_HUMAN]	20,22	2
O15320	Cutaneous T-cell lymphoma-associated antigen 5 OS=Homo sapiens GN=CTAGE5 PE=1 SV=4 - [CTGE5_HUMAN]	20,15	2
Q13247	Serine/arginine-rich splicing factor 6 OS=Homo sapiens GN=SRSF6 PE=1 SV=2 - [SRSF6_HUMAN]	20,06	2
Q9BV81	Transmembrane protein 93 OS=Homo sapiens GN=TMEM93 PE=1 SV=1 - [TMM93_HUMAN]	20,00	2
H3BP66	N-acetylgalactosamine-6-sulfatase (Fragment) OS=Homo sapiens GN=GALNS PE=4 SV=1 - [H3BP66_HUMAN]	20,00	2
Q86Y39	NADH dehydrogenase [ubiquinone] 1 alpha subcomplex subunit 11 OS=Homo sapiens GN=NDUFA11 PE=1 SV=3 - [NDUAB_HUMAN]	19,86	2
P63165	Small ubiquitin-related modifier 1 OS=Homo sapiens GN=SUMO1 PE=1 SV=1 - [SUMO1_HUMAN]	19,80	2
O95166	Gamma-aminobutyric acid receptor-associated protein OS=Homo sapiens GN=GABARAP PE=1 SV=1 - [GBRAP_HUMAN]	19,66	2
Q9BQC6	Ribosomal protein 63, mitochondrial OS=Homo sapiens GN=MRP63 PE=2 SV=1 - [RT63_HUMAN]	19,61	2
Q9BW61	DET1- and DDB1-associated protein 1 OS=Homo sapiens GN=DDA1 PE=1 SV=1 - [DDA1_HUMAN]	19,61	2
Q9BT67	NEDD4 family-interacting protein 1 OS=Homo sapiens GN=NDFIP1 PE=1 SV=1 - [NFIP1_HUMAN]	19,46	2
P52434	DNA-directed RNA polymerases I, II, and III subunit RPABC3 OS=Homo sapiens GN=POLR2H PE=1 SV=4 - [RPAB3_HUMAN]	19,33	2
Q9UL33	Trafficking protein particle complex subunit 2-like protein OS=Homo sapiens GN=TRAPPC2L PE=1 SV=1 - [TPC2L_HUMAN]	19,29	2
Q9UMY4	Sorting nexin-12 OS=Homo sapiens GN=SNX12 PE=1 SV=3 - [SNX12_HUMAN]	19,19	2
Q86WX3	Active regulator of SIRT1 OS=Homo sapiens GN=RPS19BP1 PE=1 SV=1 - [AROS_HUMAN]	19,12	2
Q86SX6	Glutaredoxin-related protein 5, mitochondrial OS=Homo sapiens GN=GLRX5 PE=1 SV=2 - [GLRX5_HUMAN]	19,11	2
O15551	Claudin-3 OS=Homo sapiens GN=CLDN3 PE=1 SV=1 - [CLD3_HUMAN]	19,09	2
Q8IXM3	39S ribosomal protein L41, mitochondrial OS=Homo sapiens GN=MRPL41 PE=1 SV=1 - [RM41_HUMAN]	18,98	2
Q9BRT6	Protein LLP homolog OS=Homo sapiens GN=LLPH PE=2 SV=1 - [LLPH_HUMAN]	18,60	2
C9JLW8	Protein FAM195B OS=Homo sapiens GN=FAM195B PE=3 SV=1 - [F195B_HUMAN]	18,56	2
A8MWD9	Small nuclear ribonucleoprotein G-like protein OS=Homo sapiens PE=3 SV=2 - [RUXGL_HUMAN]	18,42	2
Q14240	Eukaryotic initiation factor 4A-II OS=Homo sapiens GN=EIF4A2 PE=1 SV=2 - [IF4A2_HUMAN]	18,18	2
Q96KA5	Cleft lip and palate transmembrane protein 1-like protein OS=Homo sapiens GN=CLPTM1L PE=1 SV=1 - [CLP1L_HUMAN]	18,03	2
Q96AJ9	Vesicle transport through interaction with t-SNAREs homolog 1A OS=Homo sapiens GN=VTI1A PE=1 SV=2 - [VTI1A_HUMAN]	17,51	2
Q8N8J7	Uncharacterized protein C4orf32 OS=Homo sapiens GN=C4orf32 PE=1 SV=2 - [CD032_HUMAN]	17,42	2
P14174	Macrophage migration inhibitory factor OS=Homo sapiens GN=MIF PE=1 SV=4 - [MIF_HUMAN]	17,39	2
P23434	Glycine cleavage system H protein, mitochondrial OS=Homo sapiens GN=GCSH PE=1 SV=2 - [GCSH_HUMAN]	17,34	2
O60613	15 kDa selenoprotein OS=Homo sapiens GN=SEP15 PE=1 SV=3 - [SEP15_HUMAN]	17,28	2
P30049	ATP synthase subunit delta, mitochondrial OS=Homo sapiens GN=ATP5D PE=1 SV=2 - [ATPD_HUMAN]	17,26	2
Q96AB3	Isochorismatase domain-containing protein 2, mitochondrial OS=Homo sapiens GN=ISOC2 PE=1 SV=1 - [ISOC2_HUMAN]	17,07	2
Q16611	Bcl-2 homologous antagonist/killer OS=Homo sapiens GN=BAK1 PE=1 SV=1 - [BAK_HUMAN]	17,06	2
Q9Y478	5'-AMP-activated protein kinase subunit beta-1 OS=Homo sapiens GN=PRKAB1 PE=1 SV=4 - [AAKB1_HUMAN]	17,04	2
P61923	Coatomer subunit zeta-1 OS=Homo sapiens GN=COPZ1 PE=1 SV=1 - [COPZ1_HUMAN]	16,95	2
Q15370	Transcription elongation factor B polypeptide 2 OS=Homo sapiens GN=TCEB2 PE=1 SV=1 - [ELOB_HUMAN]	16,95	2
Q9BYD1	39S ribosomal protein L13, mitochondrial OS=Homo sapiens GN=MRPL13 PE=1 SV=1 - [RM13_HUMAN]	16,85	2
A6NCE7	Microtubule-associated proteins 1A/1B light chain 3 beta 2 OS=Homo sapiens GN=MAP1LC3B2 PE=2 SV=1 - [MP3B2_HUMAN]	16,80	2
P51571	Translocon-associated protein subunit delta OS=Homo sapiens GN=SSR4 PE=1 SV=1 - [SSRD_HUMAN]	16,76	2
Q9BQI0	Allograft inflammatory factor 1-like OS=Homo sapiens GN=AIF1L PE=1 SV=1 - [AIF1L_HUMAN]	16,67	2
Q10589	Bone marrow stromal antigen 2 OS=Homo sapiens GN=BST2 PE=1 SV=1 - [BST2_HUMAN]	16,67	2
Q13951-2	Isoform 2 of Core-binding factor subunit beta OS=Homo sapiens GN=CBFB - [PEBB_HUMAN]	16,58	2
P63098	Calcineurin subunit B type 1 OS=Homo sapiens GN=PPP3R1 PE=1 SV=2 - [CANB1_HUMAN]	16,47	2
Q9UKR5	Probable ergosterol biosynthetic protein 28 OS=Homo sapiens GN=C14orf1 PE=1 SV=1 - [ERG28_HUMAN]	16,43	2
Q5BJH2	Transmembrane protein 128 OS=Homo sapiens GN=TMEM128 PE=2 SV=1 - [TM128_HUMAN]	16,36	2
Q6ZSJ8	Uncharacterized protein C1orf122 OS=Homo sapiens GN=C1orf122 PE=2 SV=2 - [CA122_HUMAN]	16,36	2
P29034	Protein S100-A2 OS=Homo sapiens GN=S100A2 PE=1 SV=3 - [S10A2_HUMAN]	16,33	2
Q96CF2	Charged multivesicular body protein 4c OS=Homo sapiens GN=CHMP4C PE=1 SV=1 - [CHM4C_HUMAN]	16,31	2
Q9BUL9	Ribonuclease P protein subunit p25 OS=Homo sapiens GN=RPP25 PE=1 SV=1 - [RPP25_HUMAN]	16,08	2
P40855	Peroxisomal biogenesis factor 19 OS=Homo sapiens GN=PEX19 PE=1 SV=1 - [PEX19_HUMAN]	16,05	2
Q13526	Peptidyl-prolyl cis-trans isomerase NIMA-interacting 1 OS=Homo sapiens GN=PIN1 PE=1 SV=1 - [PIN1_HUMAN]	15,95	2

C9JI98	Transmembrane protein 238 OS=Homo sapiens GN=TMEM238 PE=2 SV=1 - [TM238_HUMAN]	15,91
Q9HAC8	Ubiquitin domain-containing protein 1 OS=Homo sapiens GN=UBTD1 PE=1 SV=1 - [UBTD1_HUMAN]	15,86
P62633	Cellular nucleic acid-binding protein OS=Homo sapiens GN=CNBP PE=1 SV=1 - [CNBP_HUMAN]	15,82
Q9NUP9	Protein lin-7 homolog C OS=Homo sapiens GN=LIN7C PE=1 SV=1 - [LIN7C_HUMAN]	15,74
P57729	Ras-related protein Rab-38 OS=Homo sapiens GN=RAB38 PE=1 SV=1 - [RAB38_HUMAN]	15,64
Q13444-10	Isoform 10 of Disintegrin and metalloproteinase domain-containing protein 15 OS=Homo sapiens GN=ADAM15 - [ADA15_HUMAN]	15,54
Q9H4B7	Tubulin beta-1 chain OS=Homo sapiens GN=TUBB1 PE=1 SV=1 - [TBB1_HUMAN]	15,52
Q92934	Bcl2 antagonist of cell death OS=Homo sapiens GN=BAD PE=1 SV=3 - [BAD_HUMAN]	15,48
Q7Z7H8	39S ribosomal protein L10, mitochondrial OS=Homo sapiens GN=MRPL10 PE=1 SV=3 - [RM10_HUMAN]	15,33
Q9NUU7	ATP-dependent RNA helicase DDX19A OS=Homo sapiens GN=DDX19A PE=1 SV=1 - [DD19A_HUMAN]	15,27
P60033	CD81 antigen OS=Homo sapiens GN=CD81 PE=1 SV=1 - [CD81_HUMAN]	15,25
P30046	D-dopachrome decarboxylase OS=Homo sapiens GN=DDT PE=1 SV=3 - [DOPD_HUMAN]	15,25
Q9Y3B4	Pre-mRNA branch site protein p14 OS=Homo sapiens GN=SF3B14 PE=1 SV=1 - [PM14_HUMAN]	15,20
Q96EL3	39S ribosomal protein L53, mitochondrial OS=Homo sapiens GN=MRPL53 PE=1 SV=1 - [RM53_HUMAN]	15,18
P11908	Ribose-phosphate pyrophosphokinase 2 OS=Homo sapiens GN=PRPS2 PE=1 SV=2 - [PRPS2_HUMAN]	15,09
Q9H9H4	Vacuolar protein sorting-associated protein 37B OS=Homo sapiens GN=VPS37B PE=1 SV=1 - [VP37B_HUMAN]	15,09
Q9P1T7	MyoD family inhibitor domain-containing protein OS=Homo sapiens GN=MDFIC PE=1 SV=2 - [MDFIC_HUMAN]	15,04
Q969H8	UPF0556 protein C19orf10 OS=Homo sapiens GN=C19orf10 PE=1 SV=1 - [CS010_HUMAN]	15,03
Q96NT0	Coiled-coil domain-containing protein 115 OS=Homo sapiens GN=CCDC115 PE=1 SV=1 - [CC115_HUMAN]	15,00
O14618	Copper chaperone for superoxide dismutase OS=Homo sapiens GN=CCS PE=1 SV=1 - [CCS_HUMAN]	14,96
P82664	28S ribosomal protein S10, mitochondrial OS=Homo sapiens GN=MRPS10 PE=1 SV=2 - [RT10_HUMAN]	14,93
Q14210	Lymphocyte antigen 6D OS=Homo sapiens GN=LY6D PE=1 SV=1 - [LY6D_HUMAN]	14,84
Q9BY50	Signal peptidase complex catalytic subunit SEC11C OS=Homo sapiens GN=SEC11C PE=1 SV=3 - [SC11C_HUMAN]	14,58
Q9UK45	U6 snRNA-associated Sm-like protein LSm7 OS=Homo sapiens GN=LSM7 PE=1 SV=1 - [LSM7_HUMAN]	14,56
Q9Y657	Spindlin-1 OS=Homo sapiens GN=SPIN1 PE=1 SV=3 - [SPIN1_HUMAN]	14,50
Q9UDX5	Mitochondrial fission process protein 1 OS=Homo sapiens GN=MTFP1 PE=1 SV=1 - [MTFP1_HUMAN]	14,46
Q8TCD5	5'(3')-deoxyribonucleotidase, cytosolic type OS=Homo sapiens GN=NT5C PE=1 SV=2 - [NT5C_HUMAN]	14,43
Q16540	39S ribosomal protein L23, mitochondrial OS=Homo sapiens GN=MRPL23 PE=1 SV=1 - [RM23_HUMAN]	14,38
Q8WY22	BRI3-binding protein OS=Homo sapiens GN=BRI3BP PE=1 SV=1 - [BRI3B_HUMAN]	14,34
Q9NRV9	Heme-binding protein 1 OS=Homo sapiens GN=HEBP1 PE=1 SV=1 - [HEBP1_HUMAN]	14,29
P60891	Ribose-phosphate pyrophosphokinase 1 OS=Homo sapiens GN=PRPS1 PE=1 SV=2 - [PRPS1_HUMAN]	14,15
Q15828	Cystatin-M OS=Homo sapiens GN=CST6 PE=1 SV=1 - [CYTM_HUMAN]	14,09
P57735	Ras-related protein Rab-25 OS=Homo sapiens GN=RAB25 PE=1 SV=2 - [RAB25_HUMAN]	14,08
Q07820	Induced myeloid leukemia cell differentiation protein Mcl-1 OS=Homo sapiens GN=MCL1 PE=1 SV=3 - [MCL1_HUMAN]	14,00
Q9UDY4	DnaJ homolog subfamily B member 4 OS=Homo sapiens GN=DNAJB4 PE=1 SV=1 - [DNJB4_HUMAN]	13,95
O14519	Cyclin-dependent kinase 2-associated protein 1 OS=Homo sapiens GN=CDK2AP1 PE=1 SV=1 - [CDKA1_HUMAN]	13,91
Q9NY26	Zinc transporter ZIP1 OS=Homo sapiens GN=SLC39A1 PE=1 SV=1 - [S39A1_HUMAN]	13,89
P53801	Pituitary tumor-transforming gene 1 protein-interacting protein OS=Homo sapiens GN=PTTG1IP PE=1 SV=1 - [PTTG_HUMAN]	13,89
Q9BQ15	SOSS complex subunit B1 OS=Homo sapiens GN=OBFC2B PE=1 SV=1 - [SOSB1_HUMAN]	13,74
Q9NV56	MRG-binding protein OS=Homo sapiens GN=MRGBP PE=1 SV=1 - [MRGBP_HUMAN]	13,73
Q8N9Z2	Coiled-coil domain-containing protein 71L OS=Homo sapiens GN=CCDC71L PE=2 SV=2 - [CC71L_HUMAN]	13,62
P84090	Enhancer of rudimentary homolog OS=Homo sapiens GN=ERH PE=1 SV=1 - [ERH_HUMAN]	13,46
Q8NC96	Adaptin ear-binding coat-associated protein 1 OS=Homo sapiens GN=NECAP1 PE=1 SV=2 - [NECP1_HUMAN]	13,45
Q9Y3Q3	Transmembrane emp24 domain-containing protein 3 OS=Homo sapiens GN=TMED3 PE=1 SV=1 - [TMED3_HUMAN]	13,36
Q8N9N8	Probable RNA-binding protein EIF1AD OS=Homo sapiens GN=EIF1AD PE=1 SV=1 - [EIF1A_HUMAN]	13,33
P00492	Hypoxanthine-guanine phosphoribosyltransferase OS=Homo sapiens GN=HPRT1 PE=1 SV=2 - [HPRT_HUMAN]	13,30
P09234	U1 small nuclear ribonucleoprotein C OS=Homo sapiens GN=SNRPC PE=1 SV=1 - [RU1C_HUMAN]	13,21
Q9NZQ7	Programmed cell death 1 ligand 1 OS=Homo sapiens GN=CD274 PE=1 SV=1 - [PD1L1_HUMAN]	13,10
Q15121	Astrocytic phosphoprotein PEA-15 OS=Homo sapiens GN=PEA15 PE=1 SV=2 - [PEA15_HUMAN]	13,08
Q13765	Nascent polypeptide-associated complex subunit alpha OS=Homo sapiens GN=NACA PE=1 SV=1 - [NACA_HUMAN]	13,02
Q13444	Disintegrin and metalloproteinase domain-containing protein 15 OS=Homo sapiens GN=ADAM15 PE=1 SV=4 - [ADA15_HUMAN]	12,98
Q96E22	Nogo-B receptor OS=Homo sapiens GN=NUS1 PE=2 SV=1 - [NGBR_HUMAN]	12,97
P25686	DnaJ homolog subfamily B member 2 OS=Homo sapiens GN=DNAJB2 PE=1 SV=3 - [DNJB2_HUMAN]	12,96
P02042	Hemoglobin subunit delta OS=Homo sapiens GN=HBD PE=1 SV=2 - [HBD_HUMAN]	12,93
Q8N726	Cyclin-dependent kinase inhibitor 2A, isoform 4 OS=Homo sapiens GN=CDKN2A PE=1 SV=2 - [CD2A2_HUMAN]	12,88
P21283	V-type proton ATPase subunit C 1 OS=Homo sapiens GN=ATP6V1C1 PE=1 SV=4 - [VATC1_HUMAN]	12,83
Q96EY8	Cob(I)yrinic acid a,c-diamide adenosyltransferase, mitochondrial OS=Homo sapiens GN=MMAB PE=1 SV=1 - [MMAB_HUMAN]	12,80
P25325	3-mercaptopyruvate sulfurtransferase OS=Homo sapiens GN=MPST PE=1 SV=3 - [THTM_HUMAN]	12,79
O00165	HCLS1-associated protein X-1 OS=Homo sapiens GN=HAX1 PE=1 SV=2 - [HAX1_HUMAN]	12,54
Q4VC31	Coiled-coil domain-containing protein 58 OS=Homo sapiens GN=CCDC58 PE=1 SV=1 - [CCD58_HUMAN]	12,50
Q8WUK0	Protein-tyrosine phosphatase mitochondrial 1 OS=Homo sapiens GN=PTPMT1 PE=1 SV=1 - [PTPM1_HUMAN]	12,44
Q96B96	Promethin OS=Homo sapiens GN=TMEM159 PE=2 SV=2 - [TM159_HUMAN]	12,42
P61326	Protein mago nashi homolog OS=Homo sapiens GN=MAGOH PE=1 SV=1 - [MGN_HUMAN]	12,33
Q9H019	Protein FAM54B OS=Homo sapiens GN=FAM54B PE=1 SV=2 - [FA54B_HUMAN]	12,33
Q96AT9	Ribulose-phosphate 3-epimerase OS=Homo sapiens GN=RPE PE=1 SV=1 - [RPE_HUMAN]	12,28
O00623	Peroxisome assembly protein 12 OS=Homo sapiens GN=PEX12 PE=1 SV=1 - [PEX12_HUMAN]	12,26
Q9NX00	Transmembrane protein 160 OS=Homo sapiens GN=TMEM160 PE=2 SV=1 - [TM160_HUMAN]	12,23
Q13242	Serine/arginine-rich splicing factor 9 OS=Homo sapiens GN=SRSF9 PE=1 SV=1 - [SRSF9_HUMAN]	12,22
Q9BY49	Peroxisomal trans-2-enoyl-CoA reductase OS=Homo sapiens GN=PECR PE=1 SV=2 - [PECR_HUMAN]	12,21
Q96FW1	Ubiquitin thioesterase OTUB1 OS=Homo sapiens GN=OTUB1 PE=1 SV=2 - [OTUB1_HUMAN]	12,18
Q9NPD8	Ubiquitin-conjugating enzyme E2 T OS=Homo sapiens GN=UBE2T PE=1 SV=1 - [UBE2T_HUMAN]	12,18
Q5BJH7	Protein YIF1B OS=Homo sapiens GN=YIF1B PE=1 SV=1 - [YIF1B_HUMAN]	12,10
Q9NQC3	Reticulon-4 OS=Homo sapiens GN=RTN4 PE=1 SV=2 - [RTN4_HUMAN]	12,08
Q96LW7-2	Isoform 2 of Bcl10-interacting CARD protein OS=Homo sapiens GN=C9orf89 - [BINCA_HUMAN]	12,02
P28482	Mitogen-activated protein kinase 1 OS=Homo sapiens GN=MAPK1 PE=1 SV=3 - [MK01_HUMAN]	11,94
Q9NRX1	RNA-binding protein PNO1 OS=Homo sapiens GN=PNO1 PE=1 SV=1 - [PNO1_HUMAN]	11,90
Q5XKP0	Protein QIL1 OS=Homo sapiens GN=QIL1 PE=1 SV=1 - [QIL1_HUMAN]	11,86
P07305	Histone H1.0 OS=Homo sapiens GN=H1F0 PE=1 SV=3 - [H10_HUMAN]	11,86
P00568	Adenylate kinase isoenzyme 1 OS=Homo sapiens GN=AK1 PE=1 SV=3 - [KAD1_HUMAN]	11,86
Q9NT62	Ubiquitin-like-conjugating enzyme ATG3 OS=Homo sapiens GN=ATG3 PE=1 SV=1 - [ATG3_HUMAN]	11,78
Q9BQA9	Uncharacterized protein C17orf62 OS=Homo sapiens GN=C17orf62 PE=1 SV=1 - [CQ062_HUMAN]	11,76
Q9BUH6	Uncharacterized protein C9orf142 OS=Homo sapiens GN=C9orf142 PE=1 SV=2 - [CI142_HUMAN]	11,76
Q9NW81	ATP synthase subunit s-like protein OS=Homo sapiens GN=ATP5SL PE=2 SV=3 - [AT5SL_HUMAN]	11,67
Q9H1X3	DnaJ homolog subfamily C member 25 OS=Homo sapiens GN=DNAJC25 PE=2 SV=1 - [DJC25_HUMAN]	11,67
Q14197	Peptidyl-tRNA hydrolase ICT1, mitochondrial OS=Homo sapiens GN=ICT1 PE=1 SV=1 - [ICT1_HUMAN]	11,65
P19387	DNA-directed RNA polymerase II subunit RPB3 OS=Homo sapiens GN=POLR2C PE=1 SV=2 - [RPB3_HUMAN]	11,64
Q14011	Cold-inducible RNA-binding protein OS=Homo sapiens GN=CIRBP PE=1 SV=1 - [CIRBP_HUMAN]	11,63
Q9NZL4	Hsp70-binding protein 1 OS=Homo sapiens GN=HSPBP1 PE=1 SV=1 - [HPBP1_HUMAN]	11,60
O95865	N(G),N(G)-dimethylarginine dimethylaminohydrolase 2 OS=Homo sapiens GN=DDAH2 PE=1 SV=1 - [DDAH2_HUMAN]	11,58
P08579	U2 small nuclear ribonucleoprotein B'' OS=Homo sapiens GN=SNRPB2 PE=1 SV=1 - [RU2B_HUMAN]	11,56
Q8WUY1	UPF0670 protein C8orf55 OS=Homo sapiens GN=C8orf55 PE=1 SV=2 - [CH055_HUMAN]	11,54
O95900	Probable tRNA pseudouridine synthase 2 OS=Homo sapiens GN=TRUB2 PE=2 SV=1 - [TRUB2_HUMAN]	11,48
Q9UBQ5	Eukaryotic translation initiation factor 3 subunit K OS=Homo sapiens GN=EIF3K PE=1 SV=1 - [EIF3K_HUMAN]	11,47
O95832	Claudin-1 OS=Homo sapiens GN=CLDN1 PE=1 SV=1 - [CLD1_HUMAN]	11,37
P82914	28S ribosomal protein S15, mitochondrial OS=Homo sapiens GN=MRPS15 PE=1 SV=1 - [RT15_HUMAN]	11,28
Q9NXU5	ADP-ribosylation factor-like protein 15 OS=Homo sapiens GN=ARL15 PE=1 SV=1 - [ARL15_HUMAN]	11,27
O60725	Protein-S-isoprenylcysteine O-methyltransferase OS=Homo sapiens GN=ICMT PE=1 SV=1 - [ICMT_HUMAN]	11,27
O14944	Proepiregulin OS=Homo sapiens GN=EREG PE=1 SV=1 - [EREG_HUMAN]	11,24
Q93062	RNA-binding protein with multiple splicing OS=Homo sapiens GN=RBPMS PE=1 SV=1 - [RBPMS_HUMAN]	11,22
Q567V2	Mpv17-like protein 2 OS=Homo sapiens GN=MPV17L2 PE=1 SV=2 - [M17L2_HUMAN]	11,17
Q15287	RNA-binding protein with serine-rich domain 1 OS=Homo sapiens GN=RNPS1 PE=1 SV=1 - [RNPS1_HUMAN]	11,15
Q53FV1	ORM1-like protein 2 OS=Homo sapiens GN=ORMDL2 PE=2 SV=2 - [ORML2_HUMAN]	11,11
Q8N2A8	Mitochondrial cardiolipin hydrolase OS=Homo sapiens GN=PLD6 PE=1 SV=1 - [PLD6_HUMAN]	11,11
Q9BXK1	Krueppel-like factor 16 OS=Homo sapiens GN=KLF16 PE=1 SV=1 - [KLF16_HUMAN]	11,11
Q99943	1-acyl-sn-glycerol-3-phosphate acyltransferase alpha OS=Homo sapiens GN=AGPAT1 PE=2 SV=2 - [PLCA_HUMAN]	10,95
Q01995	Transgelin OS=Homo sapiens GN=TAGLN PE=1 SV=4 - [TAGL_HUMAN]	10,95
Q15390	Mitochondrial fission regulator 1 OS=Homo sapiens GN=MTFR1 PE=1 SV=2 - [MTFR1_HUMAN]	10,81
Q16762	Thiosulfate sulfurtransferase OS=Homo sapiens GN=TST PE=1 SV=4 - [THTR_HUMAN]	10,77
Q8N4T4	Vav-like protein C9orf100 OS=Homo sapiens GN=C9orf100 PE=2 SV=1 - [CI100_HUMAN]	10,75
P41236	Protein phosphatase inhibitor 2 OS=Homo sapiens GN=PPP1R2 PE=1 SV=2 - [IPP2_HUMAN]	10,73
Q9BVK8	Transmembrane protein 147 OS=Homo sapiens GN=TMEM147 PE=1 SV=1 - [TM147_HUMAN]	10,71
Q8WUY8	N-acetyltransferase 14 OS=Homo sapiens GN=NAT14 PE=1 SV=1 - [NAT14_HUMAN]	10,68
Q9H147	Deoxynucleotidyltransferase terminal-interacting protein 1 OS=Homo sapiens GN=DNTTIP1 PE=1 SV=2 - [TDIF1_HUMAN]	10,64
Q9UBN6	Tumor necrosis factor receptor superfamily member 10D OS=Homo sapiens GN=TNFRSF10D PE=1 SV=1 - [TR10D_HUMAN]	10,62
P78381	UDP-galactose translocator OS=Homo sapiens GN=SLC35A2 PE=1 SV=1 - [S35A2_HUMAN]	10,61
P24941-2	Isoform 2 of Cyclin-dependent kinase 2 OS=Homo sapiens GN=CDK2 - [CDK2_HUMAN]	10,61
O00767	Acyl-CoA desaturase OS=Homo sapiens GN=SCD PE=1 SV=2 - [ACOD_HUMAN]	10,58
O75586	Mediator of RNA polymerase II transcription subunit 6 OS=Homo sapiens GN=MED6 PE=1 SV=2 - [MED6_HUMAN]	10,57
P54105	Methylosome subunit pICln OS=Homo sapiens GN=CLNS1A PE=1 SV=1 - [ICLN_HUMAN]	10,55
Q6UXN9	WD repeat-containing protein 82 OS=Homo sapiens GN=WDR82 PE=1 SV=1 - [WDR82_HUMAN]	10,54
Q9Y2S0	DNA-directed RNA polymerases I and III subunit RPAC2 OS=Homo sapiens GN=POLR1D PE=1 SV=1 - [RPAC2_HUMAN]	10,53
P27701	CD82 antigen OS=Homo sapiens GN=CD82 PE=1 SV=1 - [CD82_HUMAN]	10,49
P17302	Gap junction alpha-1 protein OS=Homo sapiens GN=GJA1 PE=1 SV=2 - [CXA1_HUMAN]	10,47
Q15404	Ras suppressor protein 1 OS=Homo sapiens GN=RSU1 PE=1 SV=3 - [RSU1_HUMAN]	10,47
Q99470	Stromal cell-derived factor 2 OS=Homo sapiens GN=SDF2 PE=1 SV=2 - [SDF2_HUMAN]	10,43
A8CG34	Nuclear envelope pore membrane protein POM 121C OS=Homo sapiens GN=POM121C PE=1 SV=2 - [P121C_HUMAN]	10,33
Q9BRJ6	Uncharacterized protein C7orf50 OS=Homo sapiens GN=C7orf50 PE=1 SV=1 - [CG050_HUMAN]	10,31
Q96S97	Myeloid-associated differentiation marker OS=Homo sapiens GN=MYADM PE=1 SV=2 - [MYADM_HUMAN]	10,25
P08397	Porphobilinogen deaminase OS=Homo sapiens GN=HMBS PE=1 SV=2 - [HEM3_HUMAN]	10,25
Q9BT25	HAUS augmin-like complex subunit 8 OS=Homo sapiens GN=HAUS8 PE=1 SV=3 - [HAUS8_HUMAN]	10,24
Q8N983	39S ribosomal protein L43, mitochondrial OS=Homo sapiens GN=MRPL43 PE=1 SV=1 - [RM43_HUMAN]	10,23
P67870	Casein kinase II subunit beta OS=Homo sapiens GN=CSNK2B PE=1 SV=1 - [CSK2B_HUMAN]	10,23
Q9UJY1	Heat shock protein beta-8 OS=Homo sapiens GN=HSPB8 PE=1 SV=1 - [HSPB8_HUMAN]	10,20
O15145	Actin-related protein 2/3 complex subunit 3 OS=Homo sapiens GN=ARPC3 PE=1 SV=3 - [ARPC3_HUMAN]	10,11
Q9Y508	RING finger protein 114 OS=Homo sapiens GN=RNF114 PE=1 SV=1 - [RN114_HUMAN]	10,09
Q9Y6A9	Signal peptidase complex subunit 1 OS=Homo sapiens GN=SPCS1 PE=1 SV=3 - [SPCS1_HUMAN]	10,06
Q9NR28	Diablo homolog, mitochondrial OS=Homo sapiens GN=DIABLO PE=1 SV=1 - [DBLOH_HUMAN]	10,04
P39880	Homeobox protein cut-like 1 OS=Homo sapiens GN=CUX1 PE=1 SV=3 - [CUX1_HUMAN]	10,03
Q96BQ5	Coiled-coil domain-containing protein 127 OS=Homo sapiens GN=CCDC127 PE=1 SV=1 - [CC127_HUMAN]	10,00
P24311	Cytochrome c oxidase subunit 7B, mitochondrial OS=Homo sapiens GN=COX7B PE=1 SV=2 - [COX7B_HUMAN]	10,00
O00625	Pirin OS=Homo sapiens GN=PIR PE=1 SV=1 - [PIR_HUMAN]	10,00
P35270	Sepiapterin reductase OS=Homo sapiens GN=SPR PE=1 SV=1 - [SPRE_HUMAN]	9,96
Q96PK2;Q9UPN3-5	Microtubule-actin cross-linking factor 1, isoform 4 OS=Homo sapiens GN=MACF1 PE=1 SV=2 - [MACF4_HUMAN]	9,94
Q8NHG8	E3 ubiquitin-protein ligase ZNRF2 OS=Homo sapiens GN=ZNRF2 PE=1 SV=1 - [ZNRF2_HUMAN]	9,92
Q330K2	UPF0551 protein C8orf38, mitochondrial OS=Homo sapiens GN=C8orf38 PE=1 SV=2 - [CH038_HUMAN]	9,91
P40306	Proteasome subunit beta type-10 OS=Homo sapiens GN=PSMB10 PE=1 SV=1 - [PSB10_HUMAN]	9,89
O14735	CDP-diacylglycerol--inositol 3-phosphatidyltransferase OS=Homo sapiens GN=CDIPT PE=1 SV=1 - [CDIPT_HUMAN]	9,86
P52294	Importin subunit alpha-1 OS=Homo sapiens GN=KPNA1 PE=1 SV=3 - [IMA1_HUMAN]	9,85
Q9GZS3	WD repeat-containing protein 61 OS=Homo sapiens GN=WDR61 PE=1 SV=1 - [WDR61_HUMAN]	9,84
Q9NQG5	Regulation of nuclear pre-mRNA domain-containing protein 1B OS=Homo sapiens GN=RPRD1B PE=1 SV=1 - [RPR1B_HUMAN]	9,82
Q16890	Tumor protein D53 OS=Homo sapiens GN=TPD52L1 PE=1 SV=1 - [TPD53_HUMAN]	9,80
P49366	Deoxyhypusine synthase OS=Homo sapiens GN=DHPS PE=1 SV=1 - [DHYS_HUMAN]	9,76
O75352	Mannose-P-dolichol utilization defect 1 protein OS=Homo sapiens GN=MPDU1 PE=1 SV=2 - [MPU1_HUMAN]	9,72
Q9NXF7	DDB1- and CUL4-associated factor 16 OS=Homo sapiens GN=DCAF16 PE=1 SV=1 - [DCA16_HUMAN]	9,72
P14635	G2/mitotic-specific cyclin-B1 OS=Homo sapiens GN=CCNB1 PE=1 SV=1 - [CCNB1_HUMAN]	9,70
O75712	Gap junction beta-3 protein OS=Homo sapiens GN=GJB3 PE=1 SV=1 - [CXB3_HUMAN]	9,63
P17936	Insulin-like growth factor-binding protein 3 OS=Homo sapiens GN=IGFBP3 PE=1 SV=2 - [IBP3_HUMAN]	9,62
Q15125	3-beta-hydroxysteroid-Delta(8),Delta(7)-isomerase OS=Homo sapiens GN=EBP PE=1 SV=3 - [EBP_HUMAN]	9,57
Q6NXG1-2	Isoform 2 of Epithelial splicing regulatory protein 1 OS=Homo sapiens GN=ESRP1 - [ESRP1_HUMAN]	9,54
Q8NFH5	Nucleoporin NUP53 OS=Homo sapiens GN=NUP35 PE=1 SV=1 - [NUP53_HUMAN]	9,51
Q8TA86	Retinitis pigmentosa 9 protein OS=Homo sapiens GN=RP9 PE=1 SV=2 - [RP9_HUMAN]	9,50
P30536	Translocator protein OS=Homo sapiens GN=TSPO PE=1 SV=3 - [TSPOA_HUMAN]	9,47
Q9ULR0	Pre-mRNA-splicing factor ISY1 homolog OS=Homo sapiens GN=ISY1 PE=1 SV=3 - [ISY1_HUMAN]	9,47
O95571	Protein ETHE1, mitochondrial OS=Homo sapiens GN=ETHE1 PE=1 SV=2 - [ETHE1_HUMAN]	9,45
Q9NQT5	Exosome complex component RRP40 OS=Homo sapiens GN=EXOSC3 PE=1 SV=3 - [EXOS3_HUMAN]	9,45
Q5JVS0	Intracellular hyaluronan-binding protein 4 OS=Homo sapiens GN=HABP4 PE=1 SV=1 - [HABP4_HUMAN]	9,44
P09417	Dihydropteridine reductase OS=Homo sapiens GN=QDPR PE=1 SV=2 - [DHPR_HUMAN]	9,43
Q99720	Sigma non-opioid intracellular receptor 1 OS=Homo sapiens GN=SIGMAR1 PE=1 SV=1 - [SGMR1_HUMAN]	9,42
Q9BTD8	RNA-binding protein 42 OS=Homo sapiens GN=RBM42 PE=1 SV=1 - [RBM42_HUMAN]	9,38
Q99595	Mitochondrial import inner membrane translocase subunit Tim17-A OS=Homo sapiens GN=TIMM17A PE=1 SV=1 - [TI17A_HUMAN]	9,36

Q92572	AP-3 complex subunit sigma-1 OS=Homo sapiens GN=AP3S1 PE=1 SV=1 - [AP3S1_HUMAN]	9,33	2	0,873	0,898
Q9BW60	Elongation of very long chain fatty acids protein 1 OS=Homo sapiens GN=ELOVL1 PE=1 SV=1 - [ELOV1_HUMAN]	9,32	2	1,186	1,180
Q96IZ7	Serine/Arginine-related protein 53 OS=Homo sapiens GN=RSRC1 PE=1 SV=1 - [RSRC1_HUMAN]	9,28	2		
Q96EK6	Glucosamine 6-phosphate N-acetyltransferase OS=Homo sapiens GN=GNPNAT1 PE=1 SV=1 - [GNA1_HUMAN]	9,24	2	0,648	
P10109	Adrenodoxin, mitochondrial OS=Homo sapiens GN=FDX1 PE=1 SV=1 - [ADX_HUMAN]	9,24	2		
Q9H875	PRKR-interacting protein 1 OS=Homo sapiens GN=PRKRIP1 PE=1 SV=1 - [PKRI1_HUMAN]	9,24	2		
Q9UKD2	mRNA turnover protein 4 homolog OS=Homo sapiens GN=MRTO4 PE=1 SV=2 - [MRT4_HUMAN]	9,21	2		0,772
Q9Y5S9	RNA-binding protein 8A OS=Homo sapiens GN=RBM8A PE=1 SV=1 - [RBM8A_HUMAN]	9,20	2	0,952	
Q969T9	WW domain-binding protein 2 OS=Homo sapiens GN=WBP2 PE=1 SV=1 - [WBP2_HUMAN]	9,20	2	0,988	1,438
Q969T4	Ubiquitin-conjugating enzyme E2 E3 OS=Homo sapiens GN=UBE2E3 PE=1 SV=1 - [UB2E3_HUMAN]	9,18	2		
Q9GZZ9	Ubiquitin-like modifier-activating enzyme 5 OS=Homo sapiens GN=UBA5 PE=1 SV=1 - [UBA5_HUMAN]	9,16	2	0,789	
Q9NRY6	Phospholipid scramblase 3 OS=Homo sapiens GN=PLSCR3 PE=1 SV=2 - [PLS3_HUMAN]	9,15	2	1,234	1,786
A0PK00	Transmembrane protein 120B OS=Homo sapiens GN=TMEM120B PE=2 SV=1 - [T120B_HUMAN]	9,14	2		0,880
Q9C0K1	Zinc transporter ZIP8 OS=Homo sapiens GN=SLC39A8 PE=1 SV=1 - [S39A8_HUMAN]	9,13	2	1,365	
Q9NY35	Claudin domain-containing protein 1 OS=Homo sapiens GN=CLDND1 PE=1 SV=1 - [CLDN1_HUMAN]	9,09	2	1,756	1,390
Q16775	Hydroxyacylglutathione hydrolase, mitochondrial OS=Homo sapiens GN=HAGH PE=1 SV=2 - [GLO2_HUMAN]	9,09	2		1,119
Q9NY97	UDP-GlcNAc:betaGal beta-1,3-N-acetylglucosaminyltransferase 2 OS=Homo sapiens GN=B3GNT2 PE=1 SV=2 - [B3GN2_HUMAN]	9,07	2	0,690	
P10620	Microsomal glutathione S-transferase 1 OS=Homo sapiens GN=MGST1 PE=1 SV=1 - [MGST1_HUMAN]	9,03	2	0,741	0,718
P56749	Claudin-12 OS=Homo sapiens GN=CLDN12 PE=1 SV=1 - [CLD12_HUMAN]	9,02	2	1,120	1,056
Q9BSR8	Protein YIPF4 OS=Homo sapiens GN=YIPF4 PE=1 SV=1 - [YIPF4_HUMAN]	9,02	2	0,855	0,777
Q9H488	GDP-fucose protein O-fucosyltransferase 1 OS=Homo sapiens GN=POFUT1 PE=1 SV=1 - [OFUT1_HUMAN]	9,02	2	1,006	
Q96CS7	Pleckstrin homology domain-containing family B member 2 OS=Homo sapiens GN=PLEKHB2 PE=1 SV=1 - [PKHB2_HUMAN]	9,01	2	1,226	0,994
Q9BVG4	UPF0368 protein Cxorf26 OS=Homo sapiens GN=CXorf26 PE=1 SV=1 - [CX026_HUMAN]	9,01	2		
Q14696	LDLR chaperone MESD OS=Homo sapiens GN=MESDC2 PE=1 SV=2 - [MESD_HUMAN]	8,97	2		1,249
P24390	ER lumen protein retaining receptor 1 OS=Homo sapiens GN=KDELR1 PE=1 SV=1 - [ERD21_HUMAN]	8,96	2	1,605	1,463
Q9Y3D9	28S ribosomal protein S23, mitochondrial OS=Homo sapiens GN=MRPS23 PE=1 SV=2 - [RT23_HUMAN]	8,95	2	0,615	0,806
P35354	Prostaglandin G/H synthase 2 OS=Homo sapiens GN=PTGS2 PE=1 SV=2 - [PGH2_HUMAN]	8,94	2	0,032	0,242
Q86WA6	Valacyclovir hydrolase OS=Homo sapiens GN=BPHL PE=1 SV=1 - [BPHL_HUMAN]	8,93	2	0,816	
O43760	Synaptogyrin-2 OS=Homo sapiens GN=SYNGR2 PE=1 SV=1 - [SNG2_HUMAN]	8,93	2	1,059	0,970
Q15024	Exosome complex component RRP42 OS=Homo sapiens GN=EXOSC7 PE=1 SV=3 - [EXOS7_HUMAN]	8,93	2		
Q96B77	Transmembrane protein 186 OS=Homo sapiens GN=TMEM186 PE=2 SV=1 - [TM186_HUMAN]	8,92	2	1,096	1,235
H7C3J3	THUMP domain-containing protein 3 (Fragment) OS=Homo sapiens GN=THUMPD3 PE=4 SV=1 - [H7C3J3_HUMAN]	8,92	2		0,730
Q02750	Dual specificity mitogen-activated protein kinase kinase 1 OS=Homo sapiens GN=MAP2K1 PE=1 SV=2 - [MP2K1_HUMAN]	8,91	2	0,972	0,602
Q9UBR2	Cathepsin Z OS=Homo sapiens GN=CTSZ PE=1 SV=1 - [CATZ_HUMAN]	8,91	2	2,195	
Q9Y5Z9	UbiA prenyltransferase domain-containing protein 1 OS=Homo sapiens GN=UBIAD1 PE=1 SV=1 - [UBIA1_HUMAN]	8,88	2	1,249	1,399
Q7L5N1	COP9 signalosome complex subunit 6 OS=Homo sapiens GN=COPS6 PE=1 SV=1 - [CSN6_HUMAN]	8,87	2	0,711	
Q5TBP9	LSM14 homolog B (SCD6, S. cerevisiae) (Fragment) OS=Homo sapiens GN=LSM14B PE=2 SV=1 - [Q5TBP9_HUMAN]	8,87	2		
Q96GF1	E3 ubiquitin-protein ligase RNF185 OS=Homo sapiens GN=RNF185 PE=1 SV=1 - [RN185_HUMAN]	8,85	2	1,454	
Q15434	RNA-binding motif, single-stranded-interacting protein 2 OS=Homo sapiens GN=RBMS2 PE=1 SV=1 - [RBMS2_HUMAN]	8,85	2		
O95478	Ribosome biogenesis protein NSA2 homolog OS=Homo sapiens GN=NSA2 PE=1 SV=1 - [NSA2_HUMAN]	8,85	2		
Q16629	Serine/arginine-rich splicing factor 7 OS=Homo sapiens GN=SRSF7 PE=1 SV=1 - [SRSF7_HUMAN]	8,82	2		0,868
Q96C03	Mitochondrial dynamic protein MID49 OS=Homo sapiens GN=SMCR7 PE=1 SV=1 - [MID49_HUMAN]	8,81	2	1,207	1,101
Q96JY6	PDZ and LIM domain protein 2 OS=Homo sapiens GN=PDLIM2 PE=1 SV=1 - [PDLI2_HUMAN]	8,81	2		
G3V178	Asparaginyl-tRNA synthetase 2 (Mitochondrial)(Putative), isoform CRA_a OS=Homo sapiens GN=NARS2 PE=4 SV=1 - [G3V178_HUMAN]	8,80	2		0,838
Q7Z2W9	39S ribosomal protein L21, mitochondrial OS=Homo sapiens GN=MRPL21 PE=1 SV=2 - [RM21_HUMAN]	8,78	2	1,042	0,988
Q9Y399	28S ribosomal protein S2, mitochondrial OS=Homo sapiens GN=MRPS2 PE=1 SV=1 - [RT02_HUMAN]	8,78	2	0,842	0,884
Q7Z6M3	Allergin-1 OS=Homo sapiens GN=MILR1 PE=1 SV=2 - [MILR1_HUMAN]	8,75	2	0,567	0,509
Q8NC54	Keratinocyte-associated transmembrane protein 2 OS=Homo sapiens GN=KCT2 PE=2 SV=2 - [KCT2_HUMAN]	8,68	2	1,490	1,121
P61964	WD repeat-containing protein 5 OS=Homo sapiens GN=WDR5 PE=1 SV=1 - [WDR5_HUMAN]	8,68	2		
O94992	Protein HEXIM1 OS=Homo sapiens GN=HEXIM1 PE=1 SV=1 - [HEXI1_HUMAN]	8,64	2	0,717	
Q07011	Tumor necrosis factor receptor superfamily member 9 OS=Homo sapiens GN=TNFRSF9 PE=1 SV=1 - [TNR9_HUMAN]	8,63	2	**2,411**	**2,430**
O15120	1-acyl-sn-glycerol-3-phosphate acyltransferase beta OS=Homo sapiens GN=AGPAT2 PE=1 SV=1 - [PLCB_HUMAN]	8,63	2		0,856
Q03001-13	Isoform 8 of Dystonin OS=Homo sapiens GN=DST - [DYST_HUMAN]	8,62	2	1,548	2,109
O95476	CTD nuclear envelope phosphatase 1 OS=Homo sapiens GN=CTDNEP1 PE=1 SV=2 - [CNEP1_HUMAN]	8,61	2	1,261	1,139
Q92979	Ribosomal RNA small subunit methyltransferase NEP1 OS=Homo sapiens GN=EMG1 PE=1 SV=4 - [NEP1_HUMAN]	8,61	2		
Q9BQ75	Uncharacterized protein C3orf26 OS=Homo sapiens GN=C3orf26 PE=1 SV=2 - [CC026_HUMAN]	8,60	2	0,882	1,086
P46109	Crk-like protein OS=Homo sapiens GN=CRKL PE=1 SV=1 - [CRKL_HUMAN]	8,58	2		
Q8NEJ9	Neuroguidin OS=Homo sapiens GN=NGDN PE=1 SV=1 - [NGDN_HUMAN]	8,57	2		0,669
Q9BWD1	Acetyl-CoA acetyltransferase, cytosolic OS=Homo sapiens GN=ACAT2 PE=1 SV=2 - [THIC_HUMAN]	8,56	2	0,857	0,739
P49770	Translation initiation factor eIF-2B subunit beta OS=Homo sapiens GN=EIF2B2 PE=1 SV=3 - [EI2BB_HUMAN]	8,55	2		
Q9BVT8	Transmembrane and ubiquitin-like domain-containing protein 1 OS=Homo sapiens GN=TMUB1 PE=1 SV=1 - [TMUB1_HUMAN]	8,54	2	1,809	1,525
Q14331	Protein FRG1 OS=Homo sapiens GN=FRG1 PE=1 SV=1 - [FRG1_HUMAN]	8,53	2		
O75208	Ubiquinone biosynthesis protein COQ9, mitochondrial OS=Homo sapiens GN=COQ9 PE=1 SV=1 - [COQ9_HUMAN]	8,49	2	0,796	0,896
Q92530	Proteasome inhibitor PI31 subunit OS=Homo sapiens GN=PSMF1 PE=1 SV=2 - [PSMF1_HUMAN]	8,49	2		
Q8N3Z3	GTP-binding protein 8 OS=Homo sapiens GN=GTPBP8 PE=2 SV=1 - [GTPB8_HUMAN]	8,45	2	1,227	1,211
Q99961	Endophilin-A2 OS=Homo sapiens GN=SH3GL1 PE=1 SV=1 - [SH3G1_HUMAN]	8,42	2	1,002	
Q9NZJ6	Hexaprenyldihydroxybenzoate methyltransferase, mitochondrial OS=Homo sapiens GN=COQ3 PE=1 SV=3 - [COQ3_HUMAN]	8,40	2	0,864	1,546
O75494	Serine/arginine-rich splicing factor 10 OS=Homo sapiens GN=SRSF10 PE=1 SV=1 - [SRS10_HUMAN]	8,40	2		
Q9NY27	Serine/threonine-protein phosphatase 4 regulatory subunit 2 OS=Homo sapiens GN=PPP4R2 PE=1 SV=3 - [PP4R2_HUMAN]	8,39	2		
Q9UIJ7	GTP:AMP phosphotransferase, mitochondrial OS=Homo sapiens GN=AK3 PE=1 SV=4 - [KAD3_HUMAN]	8,37	2	1,143	
P28074	Proteasome subunit beta type-5 OS=Homo sapiens GN=PSMB5 PE=1 SV=3 - [PSB5_HUMAN]	8,37	2	1,217	1,438
A6NKD9	Coiled-coil domain-containing protein 85C OS=Homo sapiens GN=CCDC85C PE=1 SV=1 - [CC85C_HUMAN]	8,35	2		
Q7L9L4	MOB kinase activator 1B OS=Homo sapiens GN=MOB1B PE=1 SV=3 - [MOB1B_HUMAN]	8,33	2	0,773	0,828
Q5HYI7	Metaxin-3 OS=Homo sapiens GN=MTX3 PE=1 SV=2 - [MTX3_HUMAN]	8,33	2	0,971	1,677
Q5TBB1	Ribonuclease H2 subunit B OS=Homo sapiens GN=RNASEH2B PE=1 SV=1 - [RNH2B_HUMAN]	8,33	2		
Q96D31	Calcium release-activated calcium channel protein 1 OS=Homo sapiens GN=ORAI1 PE=1 SV=2 - [CRCM1_HUMAN]	8,31	2	0,643	1,031
Q76EJ3	UDP-N-acetylglucosamine/UDP-glucose/GDP-mannose transporter OS=Homo sapiens GN=SLC35D2 PE=1 SV=1 - [S35D2_HUMAN]	8,31	2	1,188	
P42126	Enoyl-CoA delta isomerase 1, mitochondrial OS=Homo sapiens GN=ECI1 PE=1 SV=1 - [ECI1_HUMAN]	8,28	2	0,469	0,568
H0Y7M6	Solute carrier family 2, facilitated glucose transporter member 8 (Fragment) OS=Homo sapiens GN=SLC2A8 PE=4 SV=1 - [H0Y7M6_HU	8,28	2		1,552
Q00534	Cyclin-dependent kinase 6 OS=Homo sapiens GN=CDK6 PE=1 SV=1 - [CDK6_HUMAN]	8,28	2		
Q9H3R2	Mucin-13 OS=Homo sapiens GN=MUC13 PE=1 SV=2 - [MUC13_HUMAN]	8,22	2		0,245
O00193	Small acidic protein OS=Homo sapiens GN=SMAP PE=1 SV=1 - [SMAP_HUMAN]	8,20	2		
P78356	Phosphatidylinositol 5-phosphate 4-kinase type-2 beta OS=Homo sapiens GN=PIP4K2B PE=1 SV=1 - [PI42B_HUMAN]	8,17	2		
Q9NVT9	Armadillo repeat-containing protein 1 OS=Homo sapiens GN=ARMC1 PE=1 SV=1 - [ARMC1_HUMAN]	8,16	2	1,203	1,112
Q8NDZ4	Deleted in autism protein 1 OS=Homo sapiens GN=C3orf58 PE=1 SV=1 - [DIA1_HUMAN]	8,14	2	0,823	0,740
Q9BSA9	Transmembrane protein 175 OS=Homo sapiens GN=TMEM175 PE=1 SV=1 - [TM175_HUMAN]	8,13	2	1,326	
Q9BX59	Tapasin-related protein OS=Homo sapiens GN=TAPBPL PE=1 SV=2 - [TPSNR_HUMAN]	8,12	2	0,719	
Q9Y625	Glypican-6 OS=Homo sapiens GN=GPC6 PE=2 SV=1 - [GPC6_HUMAN]	8,11	2	1,123	
Q96NB2	Sideroflexin-2 OS=Homo sapiens GN=SFXN2 PE=1 SV=2 - [SFXN2_HUMAN]	8,07	2	0,855	1,027
Q7LBR1	Charged multivesicular body protein 1b OS=Homo sapiens GN=CHMP1B PE=1 SV=1 - [CHM1B_HUMAN]	8,04	2		1,130
P36507	Dual specificity mitogen-activated protein kinase kinase 2 OS=Homo sapiens GN=MAP2K2 PE=1 SV=1 - [MP2K2_HUMAN]	8,00	2		
O75251	NADH dehydrogenase [ubiquinone] iron-sulfur protein 7, mitochondrial OS=Homo sapiens GN=NDUFS7 PE=1 SV=3 - [NDUS7_HUMAN]	7,98	2	1,079	1,024
P49585	Choline-phosphate cytidylyltransferase A OS=Homo sapiens GN=PCYT1A PE=1 SV=2 - [PCY1A_HUMAN]	7,90	2		
P52803	Ephrin-A5 OS=Homo sapiens GN=EFNA5 PE=1 SV=1 - [EFNA5_HUMAN]	7,89	2	1,473	1,400
Q6P1X6	UPF0598 protein C8orf82 OS=Homo sapiens GN=C8orf82 PE=1 SV=2 - [CH082_HUMAN]	7,87	2	1,228	
Q9BUP3	Oxidoreductase HTATIP2 OS=Homo sapiens GN=HTATIP2 PE=1 SV=2 - [HTAI2_HUMAN]	7,85	2	0,950	
Q9NUJ1	Abhydrolase domain-containing protein 10, mitochondrial OS=Homo sapiens GN=ABHD10 PE=1 SV=1 - [ABHDA_HUMAN]	7,84	2	0,613	
Q5VW32	BRO1 domain-containing protein BROX OS=Homo sapiens GN=BROX PE=1 SV=1 - [BROX_HUMAN]	7,79	2	0,836	0,930
A6NDG6	Phosphoglycolate phosphatase OS=Homo sapiens GN=PGP PE=1 SV=1 - [PGP_HUMAN]	7,79	2	0,844	0,621
P55042	GTP-binding protein RAD OS=Homo sapiens GN=RRAD PE=1 SV=2 - [RAD_HUMAN]	7,79	2		**2,470**
Q8MH63	Putative L-type amino acid transporter 1-like protein MLAS OS=Homo sapiens GN=SLC7A5P1 PE=5 SV=1 - [LAT1N_HUMAN]	7,78	2	1,830	1,481
Q8NCC3	Group XV phospholipase A2 OS=Homo sapiens GN=PLA2G15 PE=1 SV=2 - [PAG15_HUMAN]	7,77	2		1,246
O95684	FGFR1 oncogene partner OS=Homo sapiens GN=FGFR1OP PE=1 SV=1 - [FR1OP_HUMAN]	7,77	2		
Q8WWV3-2	Isoform 2 of Reticulon-4-interacting protein 1, mitochondrial OS=Homo sapiens GN=RTN4IP1 - [RT4I1_HUMAN]	7,77	2		
P09001	39S ribosomal protein L3, mitochondrial OS=Homo sapiens GN=MRPL3 PE=1 SV=1 - [RM03_HUMAN]	7,76	2	0,511	
Q9H8G2	Conserved anti-apoptotic protein OS=Homo sapiens GN=CAAP PE=1 SV=2 - [CAAP_HUMAN]	7,76	2		
Q07817	Bcl-2-like protein 1 OS=Homo sapiens GN=BCL2L1 PE=1 SV=1 - [B2CL1_HUMAN]	7,73	2	1,086	0,970
Q8WVF1	Protein OSCP1 OS=Homo sapiens GN=OSCP1 PE=1 SV=4 - [OSCP1_HUMAN]	7,71	2		
Q9HD42	Charged multivesicular body protein 1a OS=Homo sapiens GN=CHMP1A PE=1 SV=1 - [CHM1A_HUMAN]	7,65	2		0,955
Q5VST6	Abhydrolase domain-containing protein FAM108B1 OS=Homo sapiens GN=FAM108B1 PE=2 SV=1 - [F108B_HUMAN]	7,64	2	1,245	1,546
Q5HYI8	Rab-like protein 3 OS=Homo sapiens GN=RABL3 PE=1 SV=1 - [RABL3_HUMAN]	7,63	2		1,280
Q9BQ39	ATP-dependent RNA helicase DDX50 OS=Homo sapiens GN=DDX50 PE=1 SV=1 - [DDX50_HUMAN]	7,60	2		
Q8NEG4	Protein FAM83F OS=Homo sapiens GN=FAM83F PE=2 SV=1 - [FA83F_HUMAN]	7,60	2	0,388	0,702
P20839	Inosine-5'-monophosphate dehydrogenase 1 OS=Homo sapiens GN=IMPDH1 PE=1 SV=2 - [IMDH1_HUMAN]	7,59	2	0,921	
Q6DKJ4	Nucleoredoxin OS=Homo sapiens GN=NXN PE=1 SV=2 - [NXN_HUMAN]	7,59	2	0,691	
Q9UM22	Mammalian ependymin-related protein 1 OS=Homo sapiens GN=EPDR1 PE=1 SV=2 - [EPDR1_HUMAN]	7,59	2		1,613
Q8IUF1	COBW domain-containing protein 2 OS=Homo sapiens GN=CBWD2 PE=1 SV=1 - [CBWD2_HUMAN]	7,59	2		0,820
O60513	Beta-1,4-galactosyltransferase 4 OS=Homo sapiens GN=B4GALT4 PE=1 SV=1 - [B4GT4_HUMAN]	7,56	2	0,933	1,338
P42765	3-ketoacyl-CoA thiolase, mitochondrial OS=Homo sapiens GN=ACAA2 PE=1 SV=2 - [THIM_HUMAN]	7,56	2	0,834	
O43766	Lipoyl synthase, mitochondrial OS=Homo sapiens GN=LIAS PE=1 SV=3 - [LIAS_HUMAN]	7,53	2		
Q9C0B1	Alpha-ketoglutarate-dependent dioxygenase FTO OS=Homo sapiens GN=FTO PE=1 SV=3 - [FTO_HUMAN]	7,52	2	0,927	
Q9NZL9	Methionine adenosyltransferase 2 subunit beta OS=Homo sapiens GN=MAT2B PE=1 SV=1 - [MAT2B_HUMAN]	7,49	2		
Q8NDD1	Uncharacterized protein C1orf131 OS=Homo sapiens GN=C1orf131 PE=1 SV=3 - [CA131_HUMAN]	7,48	2		
P31350	Ribonucleoside-diphosphate reductase subunit M2 OS=Homo sapiens GN=RRM2 PE=1 SV=1 - [RIR2_HUMAN]	7,46	2	0,690	
P54920	Alpha-soluble NSF attachment protein OS=Homo sapiens GN=NAPA PE=1 SV=3 - [SNAA_HUMAN]	7,46	2	0,733	
P35237	Serpin B6 OS=Homo sapiens GN=SERPINB6 PE=1 SV=3 - [SPB6_HUMAN]	7,45	2	0,632	
Q16798	NADP-dependent malic enzyme, mitochondrial OS=Homo sapiens GN=ME3 PE=2 SV=2 - [MAON_HUMAN]	7,45	2		1,125
O15213	WD repeat-containing protein 46 OS=Homo sapiens GN=WDR46 PE=1 SV=3 - [WDR46_HUMAN]	7,38	2	0,818	
C9J8M4	Ubiquitin carboxyl-terminal hydrolase 19 (Fragment) OS=Homo sapiens GN=USP19 PE=4 SV=1 - [C9J8M4_HUMAN]	7,38	2		
P04637	Cellular tumor antigen p53 OS=Homo sapiens GN=TP53 PE=1 SV=4 - [P53_HUMAN]	7,38	2		
Q96QR8	Transcriptional activator protein Pur-beta OS=Homo sapiens GN=PURB PE=1 SV=3 - [PURB_HUMAN]	7,37	2		
Q86TW2	Uncharacterized aarF domain-containing protein kinase 1 OS=Homo sapiens GN=ADCK1 PE=1 SV=2 - [ADCK1_HUMAN]	7,36	2	1,012	
Q9NVV5	Androgen-induced gene 1 protein OS=Homo sapiens GN=AIG1 PE=1 SV=2 - [AIG1_HUMAN]	7,35	2	1,074	0,588
P21583	Kit ligand OS=Homo sapiens GN=KITLG PE=1 SV=1 - [SCF_HUMAN]	7,33	2	0,987	0,954
Q9H0W8	Protein SMG9 OS=Homo sapiens GN=SMG9 PE=1 SV=1 - [SMG9_HUMAN]	7,31	2		
O00505	Importin subunit alpha-3 OS=Homo sapiens GN=KPNA3 PE=1 SV=2 - [IMA3_HUMAN]	7,29	2		0,623
Q9Y2A9	UDP-GlcNAc:betaGal beta-1,3-N-acetylglucosaminyltransferase 3 OS=Homo sapiens GN=B3GNT3 PE=1 SV=2 - [B3GN3_HUMAN]	7,26	2	0,181	0,193
Q8N8R3	Mitochondrial carnitine/acylcarnitine carrier protein CACL OS=Homo sapiens GN=SLC25A29 PE=2 SV=2 - [MCATL_HUMAN]	7,26	2	1,579	1,458
Q16254	Transcription factor E2F4 OS=Homo sapiens GN=E2F4 PE=1 SV=2 - [E2F4_HUMAN]	7,26	2		
P28062	Proteasome subunit beta type-8 OS=Homo sapiens GN=PSMB8 PE=1 SV=3 - [PSB8_HUMAN]	7,25	2	0,773	0,765
O43286	Beta-1,4-galactosyltransferase 5 OS=Homo sapiens GN=B4GALT5 PE=2 SV=1 - [B4GT5_HUMAN]	7,22	2	1,734	1,650
Q15436	Protein transport protein Sec23A OS=Homo sapiens GN=SEC23A PE=1 SV=2 - [SC23A_HUMAN]	7,19	2		
O95070	Protein YIF1A OS=Homo sapiens GN=YIF1A PE=1 SV=2 - [YIF1A_HUMAN]	7,17	2	1,651	1,009
Q9BTE7	DCN1-like protein 5 OS=Homo sapiens GN=DCUN1D5 PE=1 SV=1 - [DCNL5_HUMAN]	7,17	2		
P15260	Interferon gamma receptor 1 OS=Homo sapiens GN=IFNGR1 PE=1 SV=1 - [INGR1_HUMAN]	7,16	2	1,260	1,194
O15382	Branched-chain-amino-acid aminotransferase, mitochondrial OS=Homo sapiens GN=BCAT2 PE=1 SV=2 - [BCAT2_HUMAN]	7,14	2		
Q8NBI5	Solute carrier family 43 member 3 OS=Homo sapiens GN=SLC43A3 PE=1 SV=2 - [S43A3_HUMAN]	7,13	2	1,732	**2,558**
Q86WV6	Transmembrane protein 173 OS=Homo sapiens GN=TMEM173 PE=1 SV=1 - [TM173_HUMAN]	7,12	2	0,593	0,440
O00154	Cytosolic acyl coenzyme A thioester hydrolase OS=Homo sapiens GN=ACOT7 PE=1 SV=3 - [BACH_HUMAN]	7,11	2		0,746
Q9H9L3	Interferon-stimulated 20 kDa exonuclease-like 2 OS=Homo sapiens GN=ISG20L2 PE=1 SV=1 - [I20L2_HUMAN]	7,08	2	0,734	
Q13884	Beta-1-syntrophin OS=Homo sapiens GN=SNTB1 PE=1 SV=3 - [SNTB1_HUMAN]	7,06	2	1,274	
P12694	2-oxoisovalerate dehydrogenase subunit alpha, mitochondrial OS=Homo sapiens GN=BCKDHA PE=1 SV=2 - [ODBA_HUMAN]	6,97	2		0,723
Q70CQ3	Ubiquitin carboxyl-terminal hydrolase 30 OS=Homo sapiens GN=USP30 PE=1 SV=1 - [UBP30_HUMAN]	6,96	2	1,321	1,168
Q8N7R7	Cyclin-Y-like protein 1 OS=Homo sapiens GN=CCNYL1 PE=1 SV=2 - [CCYL1_HUMAN]	6,96	2	0,560	
Q96FN4	Copine-2 OS=Homo sapiens GN=CPNE2 PE=1 SV=3 - [CPNE2_HUMAN]	6,93	2	0,874	0,920
Q75N03	E3 ubiquitin-protein ligase Hakai OS=Homo sapiens GN=CBLL1 PE=1 SV=1 - [HAKAI_HUMAN]	6,92	2		
Q8IYU8	EF-hand domain-containing family member A1 OS=Homo sapiens GN=EFHA1 PE=1 SV=2 - [EFHA1_HUMAN]	6,91	2		1,078
O75909	Cyclin-K OS=Homo sapiens GN=CCNK PE=1 SV=2 - [CCNK_HUMAN]	6,90	2	1,394	
Q86TX2	Acyl-coenzyme A thioesterase 1 OS=Homo sapiens GN=ACOT1 PE=1 SV=1 - [ACOT1_HUMAN]	6,89	2		0,933
P55263-3	Isoform 3 of Adenosine kinase OS=Homo sapiens GN=ADK - [ADK_HUMAN]	6,89	2		0,733
Q13426-2	Isoform 2 of DNA repair protein XRCC4 OS=Homo sapiens GN=XRCC4 - [XRCC4_HUMAN]	6,89	2		
Q9Y5X2	Sorting nexin-8 OS=Homo sapiens GN=SNX8 PE=1 SV=1 - [SNX8_HUMAN]	6,88	2	0,602	
O95562	Vesicle transport protein SFT2B OS=Homo sapiens GN=SFT2D2 PE=1 SV=1 - [SFT2B_HUMAN]	6,88	2	1,372	1,257
Q9NR50	Translation initiation factor eIF-2B subunit gamma OS=Homo sapiens GN=EIF2B3 PE=1 SV=1 - [EI2BG_HUMAN]	6,86	2		1,046
Q8IVM0	Coiled-coil domain-containing protein 50 OS=Homo sapiens GN=CCDC50 PE=1 SV=1 - [CCD50_HUMAN]	6,86	2		
Q9H0R6	Glutamyl-tRNA(Gln) amidotransferase subunit A, mitochondrial OS=Homo sapiens GN=QRSL1 PE=1 SV=2 - [GATA_HUMAN]	6,82	2	0,739	
O15121	Sphingolipid delta(4)-desaturase DES1 OS=Homo sapiens GN=DEGS1 PE=1 SV=1 - [DEGS1_HUMAN]	6,81	2	0,995	2,105
P38571	Lysosomal acid lipase/cholesteryl ester hydrolase OS=Homo sapiens GN=LIPA PE=1 SV=2 - [LICH_HUMAN]	6,77	2	1,263	0,949
Q9NRB3	Carbohydrate sulfotransferase 12 OS=Homo sapiens GN=CHST12 PE=1 SV=2 - [CHSTC_HUMAN]	6,76	2	0,948	
Q96L58	Beta-1,3-galactosyltransferase 6 OS=Homo sapiens GN=B3GALT6 PE=2 SV=2 - [B3GT6_HUMAN]	6,69	2	1,136	0,847

Q96I51	Williams-Beuren syndrome chromosomal region 16 protein OS=Homo sapiens GN=WBSCR16 PE=1 SV=2 - [WBS16_HUMAN]	6,68	2	1,009
Q8IYS1	Peptidase M20 domain-containing protein 2 OS=Homo sapiens GN=PM20D2 PE=1 SV=2 - [P20D2_HUMAN]	6,65	2	
Q92785	Zinc finger protein ubi-d4 OS=Homo sapiens GN=DPF2 PE=1 SV=2 - [REQU_HUMAN]	6,65	2	
O60779	Thiamine transporter 1 OS=Homo sapiens GN=SLC19A2 PE=1 SV=2 - [S19A2_HUMAN]	6,64	2	1,486
O75832	26S proteasome non-ATPase regulatory subunit 10 OS=Homo sapiens GN=PSMD10 PE=1 SV=1 - [PSD10_HUMAN]	6,64	2	
Q16186	Proteasomal ubiquitin receptor ADRM1 OS=Homo sapiens GN=ADRM1 PE=1 SV=2 - [ADRM1_HUMAN]	6,63	2	
Q9NRX5	Serine incorporator 1 OS=Homo sapiens GN=SERINC1 PE=1 SV=1 - [SERC1_HUMAN]	6,62	2	2,184
P30566	Adenylosuccinate lyase OS=Homo sapiens GN=ADSL PE=1 SV=2 - [PUR8_HUMAN]	6,61	2	0,891
Q99986	Serine/threonine-protein kinase VRK1 OS=Homo sapiens GN=VRK1 PE=1 SV=1 - [VRK1_HUMAN]	6,57	2	
P09668	Pro-cathepsin H OS=Homo sapiens GN=CTSH PE=1 SV=4 - [CATH_HUMAN]	6,57	2	
P09497	Clathrin light chain B OS=Homo sapiens GN=CLTB PE=1 SV=1 - [CLCB_HUMAN]	6,55	2	
Q14849	StAR-related lipid transfer protein 3 OS=Homo sapiens GN=STARD3 PE=1 SV=2 - [STAR3_HUMAN]	6,52	2	1,751
Q9BV20	Methylthioribose-1-phosphate isomerase OS=Homo sapiens GN=MRI1 PE=1 SV=1 - [MTNA_HUMAN]	6,50	2	0,852
Q9UHE8	Metalloreductase STEAP1 OS=Homo sapiens GN=STEAP1 PE=1 SV=1 - [STEA1_HUMAN]	6,49	2	0,559
Q13889	General transcription factor IIH subunit 3 OS=Homo sapiens GN=GTF2H3 PE=1 SV=2 - [TF2H3_HUMAN]	6,49	2	
Q969M1	Mitochondrial import receptor subunit TOM40B OS=Homo sapiens GN=TOMM40L PE=2 SV=1 - [TM40L_HUMAN]	6,49	2	
Q8NFL0	UDP-GlcNAc:betaGal beta-1,3-N-acetylglucosaminyltransferase 7 OS=Homo sapiens GN=B3GNT7 PE=2 SV=1 - [B3GN7_HUMAN]	6,48	2	
Q9NZ32	Actin-related protein 10 OS=Homo sapiens GN=ACTR10 PE=1 SV=1 - [ARP10_HUMAN]	6,47	2	0,714
Q14244-5	Isoform 5 of Ensconsin OS=Homo sapiens GN=MAP7 - [MAP7_HUMAN]	6,47	2	
Q92968	Peroxisomal membrane protein PEX13 OS=Homo sapiens GN=PEX13 PE=1 SV=2 - [PEX13_HUMAN]	6,45	2	0,785
Q9NUQ3	Gamma-taxilin OS=Homo sapiens GN=TXLNG PE=1 SV=2 - [TXLNG_HUMAN]	6,44	2	1,004
P14324	Farnesyl pyrophosphate synthase OS=Homo sapiens GN=FDPS PE=1 SV=4 - [FPPS_HUMAN]	6,44	2	
Q9NW64	Pre-mRNA-splicing factor RBM22 OS=Homo sapiens GN=RBM22 PE=1 SV=1 - [RBM22_HUMAN]	6,43	2	
Q8IXU6	Solute carrier family 35 member F2 OS=Homo sapiens GN=SLC35F2 PE=1 SV=1 - [S35F2_HUMAN]	6,42	2	1,202
Q9UI12	V-type proton ATPase subunit H OS=Homo sapiens GN=ATP6V1H PE=1 SV=1 - [VATH_HUMAN]	6,42	2	0,798
Q99747	Gamma-soluble NSF attachment protein OS=Homo sapiens GN=NAPG PE=1 SV=1 - [SNAG_HUMAN]	6,41	2	0,903
Q8ND24	RING finger protein 214 OS=Homo sapiens GN=RNF214 PE=1 SV=2 - [RN214_HUMAN]	6,40	2	
Q9Y5U2	Protein TSSC4 OS=Homo sapiens GN=TSSC4 PE=1 SV=3 - [TSSC4_HUMAN]	6,38	2	
Q6UW63	KDEL motif-containing protein 1 OS=Homo sapiens GN=KDELC1 PE=1 SV=1 - [KDEL1_HUMAN]	6,37	2	
P20933	N(4)-(beta-N-acetylglucosaminyl)-L-asparaginase OS=Homo sapiens GN=AGA PE=1 SV=2 - [ASPG_HUMAN]	6,36	2	
O75530	Polycomb protein EED OS=Homo sapiens GN=EED PE=1 SV=2 - [EED_HUMAN]	6,35	2	0,868
P54619	5'-AMP-activated protein kinase subunit gamma-1 OS=Homo sapiens GN=PRKAG1 PE=1 SV=1 - [AAKG1_HUMAN]	6,34	2	0,948
Q6UWB1	Interleukin-27 receptor subunit alpha OS=Homo sapiens GN=IL27RA PE=2 SV=2 - [I27RA_HUMAN]	6,29	2	0,288
Q8WVV9	Heterogeneous nuclear ribonucleoprotein L-like OS=Homo sapiens GN=HNRPLL PE=1 SV=1 - [HNRLL_HUMAN]	6,27	2	
Q12972	Nuclear inhibitor of protein phosphatase 1 OS=Homo sapiens GN=PPP1R8 PE=1 SV=2 - [PP1R8_HUMAN]	6,27	2	
Q7LGC8	Carbohydrate sulfotransferase 3 OS=Homo sapiens GN=CHST3 PE=1 SV=3 - [CHST3_HUMAN]	6,26	2	1,098
Q8NC44	Protein FAM134A OS=Homo sapiens GN=FAM134A PE=1 SV=3 - [F134A_HUMAN]	6,26	2	1,556
Q9H3U5	Major facilitator superfamily domain-containing protein 1 OS=Homo sapiens GN=MFSD1 PE=2 SV=2 - [MFSD1_HUMAN]	6,24	2	1,421
O75718	Cartilage-associated protein OS=Homo sapiens GN=CRTAP PE=1 SV=1 - [CRTAP_HUMAN]	6,23	2	2,106
Q96IW7	Vesicle-trafficking protein SEC22a OS=Homo sapiens GN=SEC22A PE=1 SV=1 - [SC22A_HUMAN]	6,19	2	1,097
Q8WUX9	Charged multivesicular body protein 7 OS=Homo sapiens GN=CHMP7 PE=1 SV=1 - [CHMP7_HUMAN]	6,18	2	
Q96NB3	Zinc finger protein 830 OS=Homo sapiens GN=ZNF830 PE=1 SV=2 - [ZN830_HUMAN]	6,18	2	
Q9NRN7	L-aminoadipate-semialdehyde dehydrogenase-phosphopantetheinyl transferase OS=Homo sapiens GN=AASDHPPT PE=1 SV=2 - [ADPPT_	6,15	2	0,727
Q9NP92	28S ribosomal protein S30, mitochondrial OS=Homo sapiens GN=MRPS30 PE=1 SV=2 - [RT30_HUMAN]	6,15	2	0,779
Q01085	Nucleolysin TIAR OS=Homo sapiens GN=TIAL1 PE=1 SV=1 - [TIAR_HUMAN]	6,13	2	0,960
P01859	Ig gamma-2 chain C region OS=Homo sapiens GN=IGHG2 PE=1 SV=2 - [IGHG2_HUMAN]	6,13	2	0,754
Q8N5B7	Ceramide synthase 5 OS=Homo sapiens GN=CERS5 PE=1 SV=1 - [CERS5_HUMAN]	6,12	2	1,290
Q8NFT2	Metalloreductase STEAP2 OS=Homo sapiens GN=STEAP2 PE=1 SV=3 - [STEA2_HUMAN]	6,12	2	0,424
O00115	Deoxyribonuclease-2-alpha OS=Homo sapiens GN=DNASE2 PE=1 SV=2 - [DNS2A_HUMAN]	6,11	2	
Q53LP3	Ankyrin repeat domain-containing protein SOWAHC OS=Homo sapiens GN=SOWAHC PE=1 SV=1 - [SWAHC_HUMAN]	6,10	2	0,497
P61201	COP9 signalosome complex subunit 2 OS=Homo sapiens GN=COPS2 PE=1 SV=1 - [CSN2_HUMAN]	6,09	2	0,899
Q14CN4	Keratin, type II cytoskeletal 72 OS=Homo sapiens GN=KRT72 PE=1 SV=2 - [K2C72_HUMAN]	6,07	2	0,984
P01857	Ig gamma-1 chain C region OS=Homo sapiens GN=IGHG1 PE=1 SV=1 - [IGHG1_HUMAN]	6,06	2	1,014
O00170	AH receptor-interacting protein OS=Homo sapiens GN=AIP PE=1 SV=2 - [AIP_HUMAN]	6,06	2	0,492
O95218	Zinc finger Ran-binding domain-containing protein 2 OS=Homo sapiens GN=ZRANB2 PE=1 SV=2 - [ZRAB2_HUMAN]	6,06	2	
P47895	Aldehyde dehydrogenase family 1 member A3 OS=Homo sapiens GN=ALDH1A3 PE=1 SV=2 - [AL1A3_HUMAN]	6,05	2	0,703
Q8IWZ8	SURP and G-patch domain-containing protein 1 OS=Homo sapiens GN=SUGP1 PE=1 SV=2 - [SUGP1_HUMAN]	6,05	2	
Q9UL15	BAG family molecular chaperone regulator 5 OS=Homo sapiens GN=BAG5 PE=1 SV=1 - [BAG5_HUMAN]	6,04	2	0,875
Q8IVF7	Formin-like protein 3 OS=Homo sapiens GN=FMNL3 PE=1 SV=3 - [FMNL3_HUMAN]	6,03	2	1,249
P78362-2	Isoform 2 of SRSF protein kinase 2 OS=Homo sapiens GN=SRPK2 - [SRPK2_HUMAN]	6,01	2	
Q8N5I2	Arrestin domain-containing protein 1 OS=Homo sapiens GN=ARRDC1 PE=2 SV=1 - [ARRD1_HUMAN]	6,00	2	
Q86VY4	Testis-specific Y-encoded-like protein 5 OS=Homo sapiens GN=TSPYL5 PE=1 SV=2 - [TSYL5_HUMAN]	6,00	2	
Q8WV24	Pleckstrin homology-like domain family A member 1 OS=Homo sapiens GN=PHLDA1 PE=1 SV=4 - [PHLA1_HUMAN]	5,99	2	0,522
P17612	cAMP-dependent protein kinase catalytic subunit alpha OS=Homo sapiens GN=PRKACA PE=1 SV=2 - [KAPCA_HUMAN]	5,98	2	0,757
Q86WR7	Uncharacterized protein C10orf47 OS=Homo sapiens GN=C10orf47 PE=1 SV=2 - [CJ047_HUMAN]	5,98	2	1,652
P16930	Fumarylacetoacetase OS=Homo sapiens GN=FAH PE=1 SV=2 - [FAAA_HUMAN]	5,97	2	
Q96B01	RAD51-associated protein 1 OS=Homo sapiens GN=RAD51AP1 PE=1 SV=1 - [R51A1_HUMAN]	5,97	2	
Q9Y3P4	Rhomboid domain-containing protein 3 OS=Homo sapiens GN=RHBDD3 PE=2 SV=1 - [RHBD3_HUMAN]	5,96	2	1,015
Q13217	DnaJ homolog subfamily C member 3 OS=Homo sapiens GN=DNAJC3 PE=1 SV=1 - [DNJC3_HUMAN]	5,95	2	
Q13895	Bystin OS=Homo sapiens GN=BYSL PE=1 SV=3 - [BYST_HUMAN]	5,95	2	
P21127-12	Isoform 7 of Cyclin-dependent kinase 11B OS=Homo sapiens GN=CDK11B - [CD11B_HUMAN]	5,92	2	
O00743	Serine/threonine-protein phosphatase 6 catalytic subunit OS=Homo sapiens GN=PPP6C PE=1 SV=1 - [PPP6_HUMAN]	5,90	2	0,977
Q9H6B4	CXADR-like membrane protein OS=Homo sapiens GN=CLMP PE=1 SV=1 - [CLMP_HUMAN]	5,90	2	1,661
Q86UB9	Transmembrane protein 135 OS=Homo sapiens GN=TMEM135 PE=2 SV=2 - [TM135_HUMAN]	5,90	2	0,748
Q9Y548	Protein YIPF1 OS=Homo sapiens GN=YIPF1 PE=2 SV=1 - [YIPF1_HUMAN]	5,88	2	0,855
Q8WU67	Abhydrolase domain-containing protein 3 OS=Homo sapiens GN=ABHD3 PE=2 SV=2 - [ABHD3_HUMAN]	5,87	2	0,793
Q15257	Serine/threonine-protein phosphatase 2A activator OS=Homo sapiens GN=PPP2R4 PE=1 SV=3 - [PTPA_HUMAN]	5,87	2	0,728
Q96MH6	Transmembrane protein 68 OS=Homo sapiens GN=TMEM68 PE=2 SV=2 - [TMM68_HUMAN]	5,86	2	0,915
Q68DK7	Male-specific lethal 1 homolog OS=Homo sapiens GN=MSL1 PE=1 SV=3 - [MSL1_HUMAN]	5,86	2	
P56545	C-terminal-binding protein 2 OS=Homo sapiens GN=CTBP2 PE=1 SV=1 - [CTBP2_HUMAN]	5,84	2	1,535
Q96GM5	SWI/SNF-related matrix-associated actin-dependent regulator of chromatin subfamily D member 1 OS=Homo sapiens GN=SMARCD1 PE:	5,83	2	1,211
Q96EE3	Nucleoporin SEH1 OS=Homo sapiens GN=SEH1L PE=1 SV=3 - [SEH1_HUMAN]	5,83	2	0,730
Q8N490-3	Isoform 3 of Probable hydrolase PNKD OS=Homo sapiens GN=PNKD - [PNKD_HUMAN]	5,82	2	0,496
Q9NYG2	Palmitoyltransferase ZDHHC3 OS=Homo sapiens GN=ZDHHC3 PE=1 SV=1 - [ZDHC3_HUMAN]	5,81	2	1,022
Q15645	Pachytene checkpoint protein 2 homolog OS=Homo sapiens GN=TRIP13 PE=1 SV=2 - [PCH2_HUMAN]	5,79	2	0,998
O43505	N-acetyllactosaminide beta-1,3-N-acetylglucosaminyltransferase OS=Homo sapiens GN=B3GNT1 PE=1 SV=1 - [B3GN1_HUMAN]	5,78	2	1,172
O75934	Pre-mRNA-splicing factor SPF27 OS=Homo sapiens GN=BCAS2 PE=1 SV=1 - [SPF27_HUMAN]	5,78	2	
Q86UD5	Mitochondrial sodium/hydrogen exchanger 9B2 OS=Homo sapiens GN=SLC9B2 PE=1 SV=2 - [SL9B2_HUMAN]	5,77	2	1,869
Q9NWD8	UPF0458 protein C7orf42 OS=Homo sapiens GN=C7orf42 PE=2 SV=1 - [CG042_HUMAN]	5,73	2	1,284
Q96CB9	Putative methyltransferase NSUN4 OS=Homo sapiens GN=NSUN4 PE=1 SV=2 - [NSUN4_HUMAN]	5,73	2	0,825
Q9H0U9	Testis-specific Y-encoded-like protein 1 OS=Homo sapiens GN=TSPYL1 PE=1 SV=3 - [TSYL1_HUMAN]	5,72	2	
O75845	Lathosterol oxidase OS=Homo sapiens GN=SC5DL PE=1 SV=2 - [SC5D_HUMAN]	5,69	2	0,847
Q96IY1	Kinetochore-associated protein NSL1 homolog OS=Homo sapiens GN=NSL1 PE=1 SV=3 - [NSL1_HUMAN]	5,69	2	
Q86V85	Integral membrane protein GPR180 OS=Homo sapiens GN=GPR180 PE=2 SV=1 - [GP180_HUMAN]	5,68	2	1,178
P48382	DNA-binding protein RFX5 OS=Homo sapiens GN=RFX5 PE=1 SV=1 - [RFX5_HUMAN]	5,68	2	
Q53HC9	Protein TSSC1 OS=Homo sapiens GN=TSSC1 PE=1 SV=2 - [TSSC1_HUMAN]	5,68	2	
P50336	Protoporphyrinogen oxidase OS=Homo sapiens GN=PPOX PE=1 SV=1 - [PPOX_HUMAN]	5,66	2	1,488
P40123	Adenylyl cyclase-associated protein 2 OS=Homo sapiens GN=CAP2 PE=1 SV=1 - [CAP2_HUMAN]	5,66	2	
O43772	Mitochondrial carnitine/acylcarnitine carrier protein OS=Homo sapiens GN=SLC25A20 PE=1 SV=1 - [MCAT_HUMAN]	5,65	2	1,483
P42785	Lysosomal Pro-X carboxypeptidase OS=Homo sapiens GN=PRCP PE=1 SV=1 - [PCP_HUMAN]	5,65	2	1,350
Q86W33	Transmembrane protein adipocyte-associated 1 OS=Homo sapiens GN=TPRA1 PE=1 SV=1 - [TPRA1_HUMAN]	5,63	2	1,226
Q96EA4	Protein Spindly OS=Homo sapiens GN=CCDC99 PE=1 SV=2 - [SPDLY_HUMAN]	5,62	2	0,999
Q05932	Folylpolyglutamate synthase, mitochondrial OS=Homo sapiens GN=FPGS PE=1 SV=3 - [FOLC_HUMAN]	5,62	2	0,853
Q9Y6I3	Epsin-1 OS=Homo sapiens GN=EPN1 PE=1 SV=2 - [EPN1_HUMAN]	5,56	2	0,721
Q96MW5	Conserved oligomeric Golgi complex subunit 8 OS=Homo sapiens GN=COG8 PE=1 SV=2 - [COG8_HUMAN]	5,56	2	0,929
Q9NWK9	Box C/D snoRNA protein 1 OS=Homo sapiens GN=ZNHIT6 PE=1 SV=1 - [BCD1_HUMAN]	5,53	2	
Q8NEM2	SHC SH2 domain-binding protein 1 OS=Homo sapiens GN=SHCBP1 PE=1 SV=3 - [SHCBP_HUMAN]	5,51	2	1,059
Q06546	GA-binding protein alpha chain OS=Homo sapiens GN=GABPA PE=1 SV=1 - [GABPA_HUMAN]	5,51	2	
Q13867	Bleomycin hydrolase OS=Homo sapiens GN=BLMH PE=1 SV=1 - [BLMH_HUMAN]	5,49	2	
Q06265	Exosome complex component RRP45 OS=Homo sapiens GN=EXOSC9 PE=1 SV=3 - [EXOS9_HUMAN]	5,47	2	0,894
P23497	Nuclear autoantigen Sp-100 OS=Homo sapiens GN=SP100 PE=1 SV=3 - [SP100_HUMAN]	5,46	2	0,619
Q8TBZ3	WD repeat-containing protein 20 OS=Homo sapiens GN=WDR20 PE=1 SV=2 - [WDR20_HUMAN]	5,45	2	0,956
Q8NFF5	FAD synthase OS=Homo sapiens GN=FLAD1 PE=1 SV=1 - [FAD1_HUMAN]	5,45	2	1,220
Q9H5K3	Protein kinase-like protein SgK196 OS=Homo sapiens GN=SGK196 PE=2 SV=1 - [SG196_HUMAN]	5,43	2	1,006
Q49A26	Putative oxidoreductase GLYR1 OS=Homo sapiens GN=GLYR1 PE=1 SV=3 - [GLYR1_HUMAN]	5,42	2	0,694
Q7Z406	Myosin-14 OS=Homo sapiens GN=MYH14 PE=1 SV=2 - [MYH14_HUMAN]	5,41	2	
Q9P0J1	[Pyruvate dehydrogenase [acetyl-transferring]]-phosphatase 1, mitochondrial OS=Homo sapiens GN=PDP1 PE=1 SV=3 - [PDP1_HUMAN]	5,40	2	
Q68CQ7	Glycosyltransferase 8 domain-containing protein 1 OS=Homo sapiens GN=GLT8D1 PE=1 SV=2 - [GL8D1_HUMAN]	5,39	2	
Q96M27	Protein PRRC1 OS=Homo sapiens GN=PRRC1 PE=1 SV=1 - [PRRC1_HUMAN]	5,39	2	
P19971	Thymidine phosphorylase OS=Homo sapiens GN=TYMP PE=1 SV=2 - [TYPH_HUMAN]	5,39	2	
O43826	Glucose-6-phosphate translocase OS=Homo sapiens GN=SLC37A4 PE=1 SV=1 - [G6PT1_HUMAN]	5,36	2	0,770
Q7L2H7	Eukaryotic translation initiation factor 3 subunit M OS=Homo sapiens GN=EIF3M PE=1 SV=1 - [EIF3M_HUMAN]	5,35	2	0,745
P32519	ETS-related transcription factor Elf-1 OS=Homo sapiens GN=ELF1 PE=1 SV=2 - [ELF1_HUMAN]	5,33	2	
Q9UM44	HERV-H LTR-associating protein 2 OS=Homo sapiens GN=HHLA2 PE=2 SV=1 - [HHLA2_HUMAN]	5,31	2	0,246
Q5VZ89-3	Isoform 3 of DENN domain-containing protein 4C OS=Homo sapiens GN=DENND4C - [DEN4C_HUMAN]	5,31	2	
Q8IZP0	Abl interactor 1 OS=Homo sapiens GN=ABI1 PE=1 SV=4 - [ABI1_HUMAN]	5,31	2	
Q9H5Q4	Dimethyladenosine transferase 2, mitochondrial OS=Homo sapiens GN=TFB2M PE=1 SV=1 - [TFB2M_HUMAN]	5,30	2	1,072
O14681	Etoposide-induced protein 2.4 homolog OS=Homo sapiens GN=EI24 PE=1 SV=4 - [EI24_HUMAN]	5,29	2	1,183
Q9NRZ5	1-acyl-sn-glycerol-3-phosphate acyltransferase delta OS=Homo sapiens GN=AGPAT4 PE=1 SV=1 - [PLCD_HUMAN]	5,29	2	1,731
Q96BI3	Gamma-secretase subunit APH-1A OS=Homo sapiens GN=APH1A PE=1 SV=1 - [APH1A_HUMAN]	5,28	2	1,245
Q5T6V5	UPF0553 protein C9orf64 OS=Homo sapiens GN=C9orf64 PE=1 SV=1 - [CI064_HUMAN]	5,28	2	1,024
P48637	Glutathione synthetase OS=Homo sapiens GN=GSS PE=1 SV=1 - [GSHB_HUMAN]	5,27	2	0,641
O95822	Malonyl-CoA decarboxylase, mitochondrial OS=Homo sapiens GN=MLYCD PE=1 SV=3 - [DCMC_HUMAN]	5,27	2	1,064
Q8NFH3	Nucleoporin Nup43 OS=Homo sapiens GN=NUP43 PE=1 SV=1 - [NUP43_HUMAN]	5,26	2	
O14967	Calmegin OS=Homo sapiens GN=CLGN PE=1 SV=1 - [CLGN_HUMAN]	5,25	2	1,491
Q8TCG2	Phosphatidylinositol 4-kinase type 2-beta OS=Homo sapiens GN=PI4K2B PE=1 SV=1 - [P4K2B_HUMAN]	5,20	2	0,770
Q6P996	Pyridoxal-dependent decarboxylase domain-containing protein 1 OS=Homo sapiens GN=PDXDC1 PE=1 SV=2 - [PDXD1_HUMAN]	5,20	2	
P50613	Cyclin-dependent kinase 7 OS=Homo sapiens GN=CDK7 PE=1 SV=1 - [CDK7_HUMAN]	5,20	2	
Q9NVC3	Putative sodium-coupled neutral amino acid transporter 7 OS=Homo sapiens GN=SLC38A7 PE=2 SV=1 - [S38A7_HUMAN]	5,19	2	1,706
Q13112	Chromatin assembly factor 1 subunit B OS=Homo sapiens GN=CHAF1B PE=1 SV=1 - [CAF1B_HUMAN]	5,19	2	
P34896	Serine hydroxymethyltransferase, cytosolic OS=Homo sapiens GN=SHMT1 PE=1 SV=1 - [GLYC_HUMAN]	5,18	2	0,828
P02765	Alpha-2-HS-glycoprotein OS=Homo sapiens GN=AHSG PE=1 SV=1 - [FETUA_HUMAN]	5,18	2	
Q86YV9	Hermansky-Pudlak syndrome 6 protein OS=Homo sapiens GN=HPS6 PE=1 SV=1 - [HPS6_HUMAN]	5,16	2	0,727
P63151	Serine/threonine-protein phosphatase 2A 55 kDa regulatory subunit B alpha isoform OS=Homo sapiens GN=PPP2R2A PE=1 SV=1 - [2AB/	5,15	2	0,925
Q99583	Max-binding protein MNT OS=Homo sapiens GN=MNT PE=1 SV=1 - [MNT_HUMAN]	5,15	2	
Q9NVX2	Notchless protein homolog 1 OS=Homo sapiens GN=NLE1 PE=1 SV=4 - [NLE1_HUMAN]	5,15	2	
Q5BKZ1	Zinc finger protein 326 OS=Homo sapiens GN=ZNF326 PE=1 SV=2 - [ZN326_HUMAN]	5,15	2	
O75052	Carboxyl-terminal PDZ ligand of neuronal nitric oxide synthase protein OS=Homo sapiens GN=NOS1AP PE=1 SV=3 - [CAPON_HUMAN]	5,14	2	0,776
Q08752	Peptidyl-prolyl cis-trans isomerase D OS=Homo sapiens GN=PPID PE=1 SV=3 - [PPID_HUMAN]	5,14	2	
O60678-2	Isoform 2 of Protein arginine N-methyltransferase 3 OS=Homo sapiens GN=PRMT3 - [ANM3_HUMAN]	5,12	2	1,043
Q9H9A5	CCR4-NOT transcription complex subunit 10 OS=Homo sapiens GN=CNOT10 PE=1 SV=1 - [CNOTA_HUMAN]	5,11	2	0,715
Q8WUA2	Peptidyl-prolyl cis-trans isomerase-like 4 OS=Homo sapiens GN=PPIL4 PE=1 SV=1 - [PPIL4_HUMAN]	5,08	2	
P08236	Beta-glucuronidase OS=Homo sapiens GN=GUSB PE=1 SV=2 - [BGLR_HUMAN]	5,07	2	0,916
Q969Z3	MOSC domain-containing protein 2, mitochondrial OS=Homo sapiens GN=MARC2 PE=1 SV=1 - [MOSC2_HUMAN]	5,07	2	1,153
P48163	NADP-dependent malic enzyme OS=Homo sapiens GN=ME1 PE=1 SV=1 - [MAOX_HUMAN]	5,07	2	
P40938	Replication factor C subunit 3 OS=Homo sapiens GN=RFC3 PE=1 SV=2 - [RFC3_HUMAN]	5,06	2	0,852
Q96SU4	Oxysterol-binding protein-related protein 9 OS=Homo sapiens GN=OSBPL9 PE=1 SV=2 - [OSBL9_HUMAN]	5,03	2	0,709
Q96AG3	Solute carrier family 25 member 46 OS=Homo sapiens GN=SLC25A46 PE=1 SV=1 - [S2546_HUMAN]	5,02	2	1,423
Q9NYB0	Telomeric repeat-binding factor 2-interacting protein 1 OS=Homo sapiens GN=TERF2IP PE=1 SV=1 - [TE2IP_HUMAN]	5,01	2	
P53367-2	Isoform A of Arfaptin-1 OS=Homo sapiens GN=ARFIP1 - [ARFP1_HUMAN]	4,99	2	1,286
Q8N0V5	N-acetyllactosaminide beta-1,6-N-acetylglucosaminyl-transferase, isoform A OS=Homo sapiens GN=GCNT2 PE=2 SV=1 - [GNT2A_HUMA	4,98	2	1,301
Q9NPI6	mRNA-decapping enzyme 1A OS=Homo sapiens GN=DCP1A PE=1 SV=2 - [DCP1A_HUMAN]	4,98	2	
Q9HA77	Probable cysteine--tRNA ligase, mitochondrial OS=Homo sapiens GN=CARS2 PE=1 SV=1 - [SYCM_HUMAN]	4,96	2	
P45984	Mitogen-activated protein kinase 9 OS=Homo sapiens GN=MAPK9 PE=1 SV=2 - [MK09_HUMAN]	4,95	2	
Q9BTA9	WW domain-containing adapter protein with coiled-coil OS=Homo sapiens GN=WAC PE=1 SV=3 - [WAC_HUMAN]	4,95	2	
Q6MZP7	Protein lin-54 homolog OS=Homo sapiens GN=LIN54 PE=1 SV=3 - [LIN54_HUMAN]	4,94	2	
P78552	Interleukin-13 receptor subunit alpha-1 OS=Homo sapiens GN=IL13RA1 PE=1 SV=1 - [I13R1_HUMAN]	4,92	2	0,740
Q8IXH7	Negative elongation factor C/D OS=Homo sapiens GN=TH1L PE=1 SV=2 - [NELFD_HUMAN]	4,92	2	
Q06787	Fragile X mental retardation protein 1 OS=Homo sapiens GN=FMR1 PE=1 SV=1 - [FMR1_HUMAN]	4,91	2	0,325
Q0VDF9	Heat shock 70 kDa protein 14 OS=Homo sapiens GN=HSPA14 PE=1 SV=1 - [HSP7E_HUMAN]	4,91	2	0,574

O60934	Nibrin OS=Homo sapiens GN=NBN PE=1 SV=1 - [NBN_HUMAN]	4,91	2			1,163
Q9H1B7	Interferon regulatory factor 2-binding protein-like OS=Homo sapiens GN=IRF2BPL PE=1 SV=1 - [I2BPL_HUMAN]	4,90	2			0,696
Q5F1R6	DnaJ homolog subfamily C member 21 OS=Homo sapiens GN=DNAJC21 PE=1 SV=2 - [DJC21_HUMAN]	4,90	2			1,122
Q6PCB8	Embigin OS=Homo sapiens GN=EMB PE=1 SV=1 - [EMB_HUMAN]	4,89	2	1,313	1,211	
P07204	Thrombomodulin OS=Homo sapiens GN=THBD PE=1 SV=2 - [TRBM_HUMAN]	4,87	2	0,777	0,421	
Q9UKN8	General transcription factor 3C polypeptide 4 OS=Homo sapiens GN=GTF3C4 PE=1 SV=2 - [TF3C4_HUMAN]	4,87	2		0,872	
Q15011	Homocysteine-responsive endoplasmic reticulum-resident ubiquitin-like domain member 1 protein OS=Homo sapiens GN=HERPUD1 PE=	4,86	2	0,968	1,194	
Q9HCD5	Nuclear receptor coactivator 5 OS=Homo sapiens GN=NCOA5 PE=1 SV=2 - [NCOA5_HUMAN]	4,84	2	0,687		1,191
Q96IF1	LIM domain-containing protein ajuba OS=Homo sapiens GN=AJUBA PE=1 SV=1 - [AJUBA_HUMAN]	4,83	2			
P08473	Neprilysin OS=Homo sapiens GN=MME PE=1 SV=2 - [NEP_HUMAN]	4,80	2	**3,075**		
P31749	RAC-alpha serine/threonine-protein kinase OS=Homo sapiens GN=AKT1 PE=1 SV=2 - [AKT1_HUMAN]	4,79	2	0,924	0,704	
P53677	AP-3 complex subunit mu-2 OS=Homo sapiens GN=AP3M2 PE=1 SV=1 - [AP3M2_HUMAN]	4,78	2	0,772	0,881	
P55081	Microfibrillar-associated protein 1 OS=Homo sapiens GN=MFAP1 PE=1 SV=2 - [MFAP1_HUMAN]	4,78	2			
Q9Y6X8	Zinc fingers and homeoboxes protein 2 OS=Homo sapiens GN=ZHX2 PE=1 SV=1 - [ZHX2_HUMAN]	4,78	2			
P36897	TGF-beta receptor type-1 OS=Homo sapiens GN=TGFBR1 PE=1 SV=1 - [TGFR1_HUMAN]	4,77	2	**2,234**		
P86790	Vacuolar fusion protein CCZ1 homolog B OS=Homo sapiens GN=CCZ1B PE=1 SV=1 - [CCZ1B_HUMAN]	4,77	2	0,944		
Q86VZ5	Phosphatidylcholine:ceramide cholinephosphotransferase 1 OS=Homo sapiens GN=SGMS1 PE=1 SV=2 - [SMS1_HUMAN]	4,77	2		0,858	
Q13480	GRB2-associated-binding protein 1 OS=Homo sapiens GN=GAB1 PE=1 SV=2 - [GAB1_HUMAN]	4,76	2	0,619		
P36896	Activin receptor type-1B OS=Homo sapiens GN=ACVR1B PE=1 SV=1 - [ACV1B_HUMAN]	4,75	2	0,863	0,866	
Q8TCB0	Interferon-induced protein 44 OS=Homo sapiens GN=IFI44 PE=2 SV=2 - [IFI44_HUMAN]	4,73	2	0,155	0,102	
Q9NXS2	Glutaminyl-peptide cyclotransferase-like protein OS=Homo sapiens GN=QPCTL PE=1 SV=2 - [QPCTL_HUMAN]	4,71	2	1,043	0,989	
Q9NVM9	Protein asunder homolog OS=Homo sapiens GN=Asun PE=1 SV=2 - [ASUN_HUMAN]	4,67	2	0,638		
Q8IWS0	PHD finger protein 6 OS=Homo sapiens GN=PHF6 PE=1 SV=1 - [PHF6_HUMAN]	4,66	2			1,174
O15091	Mitochondrial ribonuclease P protein 3 OS=Homo sapiens GN=KIAA0391 PE=1 SV=2 - [MRRP3_HUMAN]	4,63	2	0,570	0,547	
O43818	U3 small nucleolar RNA-interacting protein 2 OS=Homo sapiens GN=RRP9 PE=1 SV=1 - [U3IP2_HUMAN]	4,63	2			0,948
Q9UH65	Switch-associated protein 70 OS=Homo sapiens GN=SWAP70 PE=1 SV=1 - [SWP70_HUMAN]	4,62	2	1,067	1,564	
Q96RF0	Sorting nexin-18 OS=Homo sapiens GN=SNX18 PE=1 SV=2 - [SNX18_HUMAN]	4,62	2			0,614
O43815	Striatin OS=Homo sapiens GN=STRN PE=1 SV=4 - [STRN_HUMAN]	4,62	2			
Q8TBB5	Kelch domain-containing protein 4 OS=Homo sapiens GN=KLHDC4 PE=1 SV=1 - [KLDC4_HUMAN]	4,62	2			
O43159	Ribosomal RNA-processing protein 8 OS=Homo sapiens GN=RRP8 PE=1 SV=2 - [RRP8_HUMAN]	4,61	2		1,122	1,202
Q9HCM3-3	Isoform 3 of UPF0606 protein KIAA1549 OS=Homo sapiens GN=KIAA1549 - [K1549_HUMAN]	4,60	2	1,659	1,547	
Q6NXT6	Transmembrane anterior posterior transformation protein 1 homolog OS=Homo sapiens GN=TAPT1 PE=1 SV=1 - [TAPT1_HUMAN]	4,59	2	0,808	0,839	
Q9NX61	Transmembrane protein 161A OS=Homo sapiens GN=TMEM161A PE=2 SV=1 - [T161A_HUMAN]	4,59	2	1,094	1,496	
P49903	Selenide, water dikinase 1 OS=Homo sapiens GN=SEPHS1 PE=1 SV=2 - [SPS1_HUMAN]	4,59	2			
Q9NZV5	Selenoprotein N OS=Homo sapiens GN=SEPN1 PE=1 SV=5 - [SELN_HUMAN]	4,58	2	0,989	1,106	
Q9H7F4	Putative transmembrane protein 185B OS=Homo sapiens GN=TMEM185B PE=5 SV=2 - [T185B_HUMAN]	4,57	2		1,170	
Q8TAV0	Protein FAM76A OS=Homo sapiens GN=FAM76A PE=2 SV=1 - [FA76A_HUMAN]	4,56	2			
Q9NQ84	G-protein coupled receptor family C group 5 member C OS=Homo sapiens GN=GPRC5C PE=1 SV=2 - [GPC5C_HUMAN]	4,54	2	1,057	1,043	
Q5VV41	Rho guanine nucleotide exchange factor 16 OS=Homo sapiens GN=ARHGEF16 PE=1 SV=1 - [ARHGG_HUMAN]	4,51	2	0,713	0,594	
Q8IV08	Phospholipase D3 OS=Homo sapiens GN=PLD3 PE=1 SV=1 - [PLD3_HUMAN]	4,49	2	1,295	1,366	
Q14155-1	Isoform 1 of Rho guanine nucleotide exchange factor 7 OS=Homo sapiens GN=ARHGEF7 - [ARHG7_HUMAN]	4,49	2			1,139
O43823	A-kinase anchor protein 8 OS=Homo sapiens GN=AKAP8 PE=1 SV=1 - [AKAP8_HUMAN]	4,48	2			1,167
Q92536	Y+L amino acid transporter 2 OS=Homo sapiens GN=SLC7A6 PE=1 SV=3 - [YLAT2_HUMAN]	4,47	2	1,296	2,099	
Q6DN90	IQ motif and SEC7 domain-containing protein 1 OS=Homo sapiens GN=IQSEC1 PE=1 SV=1 - [IQEC1_HUMAN]	4,47	2	1,971		0,873
Q9Y653	G-protein coupled receptor 56 OS=Homo sapiens GN=GPR56 PE=1 SV=2 - [GPR56_HUMAN]	4,47	2	0,751	0,660	
Q03111	Protein ENL OS=Homo sapiens GN=MLLT1 PE=1 SV=2 - [ENL_HUMAN]	4,47	2			1,086
P78314	SH3 domain-binding protein 2 OS=Homo sapiens GN=SH3BP2 PE=1 SV=2 - [3BP2_HUMAN]	4,46	2		0,259	1,051
Q8NE00	Transmembrane protein 104 OS=Homo sapiens GN=TMEM104 PE=1 SV=2 - [TM104_HUMAN]	4,44	2	1,025	0,903	
Q16204	Coiled-coil domain-containing protein 6 OS=Homo sapiens GN=CCDC6 PE=1 SV=2 - [CCDC6_HUMAN]	4,43	2			
O94955	Rho-related BTB domain-containing protein 3 OS=Homo sapiens GN=RHOBTB3 PE=1 SV=2 - [RHBT3_HUMAN]	4,42	2	0,951	0,910	
Q9UBP0	Spastin OS=Homo sapiens GN=SPAST PE=1 SV=1 - [SPAST_HUMAN]	4,38	2	0,850	0,875	
Q7Z4F1	Low-density lipoprotein receptor-related protein 10 OS=Homo sapiens GN=LRP10 PE=1 SV=2 - [LRP10_HUMAN]	4,35	2		0,940	
Q05519	Serine/arginine-rich splicing factor 11 OS=Homo sapiens GN=SRSF11 PE=1 SV=1 - [SRS11_HUMAN]	4,34	2	0,780	0,735	0,952
P23219	Prostaglandin G/H synthase 1 OS=Homo sapiens GN=PTGS1 PE=1 SV=2 - [PGH1_HUMAN]	4,34	2	1,042	0,701	
Q9NWT1	p21-activated protein kinase-interacting protein 1 OS=Homo sapiens GN=PAK1IP1 PE=1 SV=2 - [PK1IP_HUMAN]	4,34	2			
P01009	Alpha-1-antitrypsin OS=Homo sapiens GN=SERPINA1 PE=1 SV=3 - [A1AT_HUMAN]	4,31	2	0,509	0,577	
P51003	Poly(A) polymerase alpha OS=Homo sapiens GN=PAPOLA PE=1 SV=4 - [PAPOA_HUMAN]	4,30	2		1,037	0,665
Q15054	DNA polymerase delta subunit 3 OS=Homo sapiens GN=POLD3 PE=1 SV=2 - [DPOD3_HUMAN]	4,29	2	0,733		
Q8TAT6	Nuclear protein localization protein 4 homolog OS=Homo sapiens GN=NPLOC4 PE=1 SV=3 - [NPL4_HUMAN]	4,28	2		0,899	1,033
A0MZ66	Shootin-1 OS=Homo sapiens GN=KIAA1598 PE=1 SV=4 - [SHOT1_HUMAN]	4,28	2			
Q7Z4Q2	HEAT repeat-containing protein 3 OS=Homo sapiens GN=HEATR3 PE=1 SV=2 - [HEAT3_HUMAN]	4,26	2	0,609		
Q8TDW0	Leucine-rich repeat-containing protein 8C OS=Homo sapiens GN=LRRC8C PE=1 SV=2 - [LRC8C_HUMAN]	4,23	2	1,028		
Q9HCL2	Glycerol-3-phosphate acyltransferase 1, mitochondrial OS=Homo sapiens GN=GPAM PE=1 SV=3 - [GPAT1_HUMAN]	4,23	2	1,234	1,128	
Q96T60	Bifunctional polynucleotide phosphatase/kinase OS=Homo sapiens GN=PNKP PE=1 SV=1 - [PNKP_HUMAN]	4,22	2			0,871
Q9UBB4	Ataxin-10 OS=Homo sapiens GN=ATXN10 PE=1 SV=1 - [ATX10_HUMAN]	4,21	2	0,722		
Q12834	Cell division cycle protein 20 homolog OS=Homo sapiens GN=CDC20 PE=1 SV=2 - [CDC20_HUMAN]	4,21	2			1,182
Q96P11	Putative methyltransferase NSUN5 OS=Homo sapiens GN=NSUN5 PE=1 SV=2 - [NSUN5_HUMAN]	4,20	2	0,951	0,799	
Q8IWB1	Inositol 1,4,5-trisphosphate receptor-interacting protein OS=Homo sapiens GN=ITPRIP PE=1 SV=1 - [IPRI_HUMAN]	4,20	2	1,448	1,197	
Q9BYX2	TBC1 domain family member 2A OS=Homo sapiens GN=TBC1D2 PE=1 SV=3 - [TBD2A_HUMAN]	4,20	2	0,671		0,565
Q9BUR4	Telomerase Cajal body protein 1 OS=Homo sapiens GN=WRAP53 PE=1 SV=1 - [WAP53_HUMAN]	4,20	2			
O75886	Signal transducing adapter molecule 2 OS=Homo sapiens GN=STAM2 PE=1 SV=1 - [STAM2_HUMAN]	4,19	2	1,180	1,348	
O43670	Zinc finger protein 207 OS=Homo sapiens GN=ZNF207 PE=1 SV=1 - [ZN207_HUMAN]	4,18	2	1,119		
Q4G176	Acyl-CoA synthetase family member 3, mitochondrial OS=Homo sapiens GN=ACSF3 PE=1 SV=3 - [ACSF3_HUMAN]	4,17	2	0,850		
Q8NHQ9	ATP-dependent RNA helicase DDX55 OS=Homo sapiens GN=DDX55 PE=1 SV=3 - [DDX55_HUMAN]	4,17	2			
Q8TCT6	Signal peptide peptidase-like 3 OS=Homo sapiens GN=SPPL3 PE=2 SV=1 - [PSL4_HUMAN]	4,16	2	0,686	0,993	
Q96SZ6	CDK5 regulatory subunit-associated protein 1 OS=Homo sapiens GN=CDK5RAP1 PE=1 SV=2 - [CK5P1_HUMAN]	4,16	2		0,661	
Q3MIX3	Uncharacterized aarF domain-containing protein kinase 5 OS=Homo sapiens GN=ADCK5 PE=2 SV=2 - [ADCK5_HUMAN]	4,14	2		1,005	
Q8IWW6	Rho GTPase-activating protein 12 OS=Homo sapiens GN=ARHGAP12 PE=1 SV=1 - [RHG12_HUMAN]	4,14	2			0,771
Q3B726	DNA-directed RNA polymerase I subunit RPA43 OS=Homo sapiens GN=TWISTNB PE=1 SV=1 - [RPA43_HUMAN]	4,14	2			
Q96P47	Arf-GAP with GTPase, ANK repeat and PH domain-containing protein 3 OS=Homo sapiens GN=AGAP3 PE=1 SV=2 - [AGAP3_HUMAN]	4,11	2	1,113	1,297	
P35367	Histamine H1 receptor OS=Homo sapiens GN=HRH1 PE=1 SV=1 - [HRH1_HUMAN]	4,11	2	1,126	0,864	
Q8WWK9	Cytoskeleton-associated protein 2 OS=Homo sapiens GN=CKAP2 PE=1 SV=1 - [CKAP2_HUMAN]	4,10	2	0,888	0,880	0,750
P04424	Argininosuccinate lyase OS=Homo sapiens GN=ASL PE=1 SV=4 - [ARLY_HUMAN]	4,09	2	0,910	0,456	
O14773	Tripeptidyl-peptidase 1 OS=Homo sapiens GN=TPP1 PE=1 SV=2 - [TPP1_HUMAN]	4,09	2	0,799	0,784	
P52756	RNA-binding protein 5 OS=Homo sapiens GN=RBM5 PE=1 SV=2 - [RBM5_HUMAN]	4,05	2			
Q9UKA2	F-box/LRR-repeat protein 4 OS=Homo sapiens GN=FBXL4 PE=1 SV=2 - [FBXL4_HUMAN]	4,03	2	1,227	1,213	
O00400	Acetyl-coenzyme A transporter 1 OS=Homo sapiens GN=SLC33A1 PE=1 SV=1 - [ACATN_HUMAN]	4,01	2	1,175	1,016	
Q9Y232	Chromodomain Y-like protein OS=Homo sapiens GN=CDYL PE=1 SV=2 - [CDYL1_HUMAN]	4,01	2			1,280
O75362	Zinc finger protein 217 OS=Homo sapiens GN=ZNF217 PE=1 SV=1 - [ZN217_HUMAN]	4,01	2			0,824
O95402	Mediator of RNA polymerase II transcription subunit 26 OS=Homo sapiens GN=MED26 PE=1 SV=2 - [MED26_HUMAN]	4,00	2			
Q9Y4C4	Malignant fibrous histiocytoma-amplified sequence 1 OS=Homo sapiens GN=MFHAS1 PE=1 SV=2 - [MFHA1_HUMAN]	3,99	2	0,566		
Q8WU79	Stromal membrane-associated protein 2 OS=Homo sapiens GN=SMAP2 PE=1 SV=1 - [SMAP2_HUMAN]	3,96	2	0,893	0,738	
Q14781	Chromobox protein homolog 2 OS=Homo sapiens GN=CBX2 PE=1 SV=2 - [CBX2_HUMAN]	3,95	2			0,954
Q7Z3B4	Nucleoporin p54 OS=Homo sapiens GN=NUP54 PE=1 SV=2 - [NUP54_HUMAN]	3,94	2	0,938		
Q9UNH7	Sorting nexin-6 OS=Homo sapiens GN=SNX6 PE=1 SV=1 - [SNX6_HUMAN]	3,94	2	0,757		
P05106	Integrin beta-3 OS=Homo sapiens GN=ITGB3 PE=1 SV=2 - [ITB3_HUMAN]	3,93	2	**2,137**	**3,279**	
Q68E01	Integrator complex subunit 3 OS=Homo sapiens GN=INTS3 PE=1 SV=1 - [INT3_HUMAN]	3,93	2			0,884
H0YDU8	Serine/threonine-protein phosphatase (Fragment) OS=Homo sapiens GN=PPP5C PE=3 SV=1 - [H0YDU8_HUMAN]	3,92	2			1,304
O95466	Formin-like protein 1 OS=Homo sapiens GN=FMNL1 PE=1 SV=3 - [FMNL_HUMAN]	3,91	2	1,698		
Q9BRX2	Protein pelota homolog OS=Homo sapiens GN=PELO PE=1 SV=2 - [PELO_HUMAN]	3,90	2	1,159		
Q96BJ8	Engulfment and cell motility protein 3 OS=Homo sapiens GN=ELMO3 PE=1 SV=3 - [ELMO3_HUMAN]	3,89	2	0,503		
F8VWL3	FYVE, RhoGEF and PH domain-containing protein 4 OS=Homo sapiens GN=FGD4 PE=4 SV=1 - [F8VWL3_HUMAN]	3,88	2			1,354
Q96BP3	Peptidylprolyl isomerase domain and WD repeat-containing protein 1 OS=Homo sapiens GN=PPWD1 PE=1 SV=1 - [PPWD1_HUMAN]	3,87	2			
Q9NRL3	Striatin-4 OS=Homo sapiens GN=STRN4 PE=1 SV=2 - [STRN4_HUMAN]	3,85	2	0,580		
Q96G46	tRNA-dihydrouridine(47) synthase [NAD(P)(+)]-like OS=Homo sapiens GN=DUS3L PE=1 SV=2 - [DUS3L_HUMAN]	3,85	2			
Q9P2K5	Myelin expression factor 2 OS=Homo sapiens GN=MYEF2 PE=1 SV=3 - [MYEF2_HUMAN]	3,83	2		0,508	
Q08AF3	Schlafen family member 5 OS=Homo sapiens GN=SLFN5 PE=1 SV=1 - [SLFN5_HUMAN]	3,82	2			
Q96ST2	Protein IWS1 homolog OS=Homo sapiens GN=IWS1 PE=1 SV=2 - [IWS1_HUMAN]	3,79	2	0,646		
Q96T88	E3 ubiquitin-protein ligase UHRF1 OS=Homo sapiens GN=UHRF1 PE=1 SV=1 - [UHRF1_HUMAN]	3,78	2			
Q15025	TNFAIP3-interacting protein 1 OS=Homo sapiens GN=TNIP1 PE=1 SV=2 - [TNIP1_HUMAN]	3,77	2	0,869		
Q6ZPD9	Protein dpy-19 homolog 3 OS=Homo sapiens GN=DPY19L3 PE=2 SV=1 - [D19L3_HUMAN]	3,77	2	1,167	1,053	
Q9P2C4	Transmembrane protein 181 OS=Homo sapiens GN=TMEM181 PE=1 SV=2 - [TM181_HUMAN]	3,76	2	1,270	0,695	
B3KRB7	Inhibitor of kappa light polypeptide gene enhancer in B-cells, kinase beta, isoform CRA_a OS=Homo sapiens GN=IKBKB PE=2 SV=1 - [	3,75	2	0,756	1,116	
Q9UPN7	Serine/threonine-protein phosphatase 6 regulatory subunit 1 OS=Homo sapiens GN=PPP6R1 PE=1 SV=5 - [PP6R1_HUMAN]	3,75	2	1,130		1,130
A4D1U4	Protein LCHN OS=Homo sapiens GN=LCHN PE=2 SV=1 - [LCHN_HUMAN]	3,74	2	1,895	1,882	
Q9P0V9	Septin-10 OS=Homo sapiens GN=SEPT10 PE=1 SV=2 - [SEP10_HUMAN]	3,74	2	1,355	0,853	
Q8NBW4	Putative sodium-coupled neutral amino acid transporter 9 OS=Homo sapiens GN=SLC38A9 PE=1 SV=2 - [S38A9_HUMAN]	3,74	2		1,549	
Q8IZ07	Ankyrin repeat domain-containing protein 13A OS=Homo sapiens GN=ANKRD13A PE=1 SV=3 - [AN13A_HUMAN]	3,73	2	0,793	1,013	
Q16512	Serine/threonine-protein kinase N1 OS=Homo sapiens GN=PKN1 PE=1 SV=2 - [PKN1_HUMAN]	3,72	2			1,165
Q8TF42	Ubiquitin-associated and SH3 domain-containing protein B OS=Homo sapiens GN=UBASH3B PE=1 SV=2 - [UBS3B_HUMAN]	3,70	2	1,269	0,771	
Q9NYV6	RNA polymerase I-specific transcription initiation factor RRN3 OS=Homo sapiens GN=RRN3 PE=1 SV=1 - [RRN3_HUMAN]	3,69	2	1,027		
O00268	Transcription initiation factor TFIID subunit 4 OS=Homo sapiens GN=TAF4 PE=1 SV=2 - [TAF4_HUMAN]	3,69	2			
Q8N3E9	1-phosphatidylinositol-4,5-bisphosphate phosphodiesterase delta-3 OS=Homo sapiens GN=PLCD3 PE=1 SV=3 - [PLCD3_HUMAN]	3,68	2	1,825	1,464	
Q15223	Poliovirus receptor-related protein 1 OS=Homo sapiens GN=PVRL1 PE=1 SV=3 - [PVRL1_HUMAN]	3,68	2	1,019	1,070	
Q32P28	Prolyl 3-hydroxylase 1 OS=Homo sapiens GN=LEPRE1 PE=1 SV=2 - [P3H1_HUMAN]	3,67	2			1,639
P98175	RNA-binding protein 10 OS=Homo sapiens GN=RBM10 PE=1 SV=3 - [RBM10_HUMAN]	3,66	2			
Q15031	Probable leucine--tRNA ligase, mitochondrial OS=Homo sapiens GN=LARS2 PE=1 SV=2 - [SYLM_HUMAN]	3,65	2	0,697		0,756
Q9BTY2	Plasma alpha-L-fucosidase OS=Homo sapiens GN=FUCA2 PE=1 SV=2 - [FUCO2_HUMAN]	3,64	2	0,879	0,561	
Q96II8	Leucine-rich repeat and calponin homology domain-containing protein 3 OS=Homo sapiens GN=LRCH3 PE=1 SV=2 - [LRCH3_HUMAN]	3,60	2		0,913	
E9PGK1	Annexin OS=Homo sapiens GN=ANXA6 PE=3 SV=1 - [E9PGK1_HUMAN]	3,59	2		1,247	
Q96PV6	Leukocyte receptor cluster member 8 OS=Homo sapiens GN=LENG8 PE=1 SV=2 - [LENG8_HUMAN]	3,59	2			1,474
P19447	TFIIH basal transcription factor complex helicase XPB subunit OS=Homo sapiens GN=ERCC3 PE=1 SV=1 - [ERCC3_HUMAN]	3,58	2	0,936		
Q86V21	Acetoacetyl-CoA synthetase OS=Homo sapiens GN=AACS PE=1 SV=1 - [AACS_HUMAN]	3,57	2			1,134
O60333-3	Isoform 3 of Kinesin-like protein KIF1B OS=Homo sapiens GN=KIF1B - [KIF1B_HUMAN]	3,56	2	1,052		
Q8NDX5	Polyhomeotic-like protein 3 OS=Homo sapiens GN=PHC3 PE=1 SV=1 - [PHC3_HUMAN]	3,56	2			1,041
P49023	Paxillin OS=Homo sapiens GN=PXN PE=1 SV=3 - [PAXI_HUMAN]	3,55	2	0,964		1,102
O43663	Protein regulator of cytokinesis 1 OS=Homo sapiens GN=PRC1 PE=1 SV=2 - [PRC1_HUMAN]	3,55	2			
Q9Y4G6	Talin-2 OS=Homo sapiens GN=TLN2 PE=1 SV=4 - [TLN2_HUMAN]	3,54	2		1,533	
P52735	Guanine nucleotide exchange factor VAV2 OS=Homo sapiens GN=VAV2 PE=1 SV=2 - [VAV2_HUMAN]	3,53	2		0,823	
Q96DM3	Uncharacterized protein C18orf8 OS=Homo sapiens GN=C18orf8 PE=1 SV=2 - [MIC1_HUMAN]	3,50	2	0,877	0,889	
Q9P2E5	Chondroitin sulfate glucuronyltransferase OS=Homo sapiens GN=CHPF2 PE=2 SV=2 - [CHPF2_HUMAN]	3,50	2	1,270	1,240	
Q9H1B5	Xylosyltransferase 2 OS=Homo sapiens GN=XYLT2 PE=2 SV=2 - [XYLT2_HUMAN]	3,47	2	0,786	0,600	
Q8TC07	TBC1 domain family member 15 OS=Homo sapiens GN=TBC1D15 PE=1 SV=2 - [TBC15_HUMAN]	3,47	2		1,069	1,067
Q13332	Receptor-type tyrosine-protein phosphatase S OS=Homo sapiens GN=PTPRS PE=1 SV=3 - [PTPRS_HUMAN]	3,44	2	1,024	1,825	
Q8NBF2	NHL repeat-containing protein 2 OS=Homo sapiens GN=NHLRC2 PE=1 SV=1 - [NHLC2_HUMAN]	3,44	2		0,779	
Q8N3V7-2	Isoform 2 of Synaptopodin OS=Homo sapiens GN=SYNPO - [SYNPO_HUMAN]	3,43	2			1,337
P25787	Proteasome subunit alpha type-2 OS=Homo sapiens GN=PSMA2 PE=1 SV=2 - [PSA2_HUMAN]	3,42	2		1,324	
Q9H4L5-4	Isoform 1d of Oxysterol-binding protein-related protein 3 OS=Homo sapiens GN=OSBPL3 - [OSBL3_HUMAN]	3,41	2			0,958
Q9NQ55	Suppressor of SWI4 1 homolog OS=Homo sapiens GN=PPAN PE=1 SV=1 - [SSF1_HUMAN]	3,38	2			
Q92854	Semaphorin-4D OS=Homo sapiens GN=SEMA4D PE=1 SV=1 - [SEM4D_HUMAN]	3,36	2	1,135		
P37173	TGF-beta receptor type-2 OS=Homo sapiens GN=TGFBR2 PE=1 SV=2 - [TGFR2_HUMAN]	3,35	2	0,886	1,271	
Q96JP5	E3 ubiquitin-protein ligase ZFP91 OS=Homo sapiens GN=ZFP91 PE=1 SV=1 - [ZFP91_HUMAN]	3,33	2	1,471		
Q96ST8	Centrosomal protein of 89 kDa OS=Homo sapiens GN=CEP89 PE=1 SV=3 - [CEP89_HUMAN]	3,32	2	1,513		
O00512	B-cell CLL/lymphoma 9 protein OS=Homo sapiens GN=BCL9 PE=1 SV=4 - [BCL9_HUMAN]	3,30	2			0,819
O15344	Midline-1 OS=Homo sapiens GN=MID1 PE=1 SV=1 - [TRI18_HUMAN]	3,30	2			
Q8WVB6	Chromosome transmission fidelity protein 18 homolog OS=Homo sapiens GN=CHTF18 PE=1 SV=1 - [CTF18_HUMAN]	3,28	2		0,837	0,720
Q8IXK2	Polypeptide N-acetylgalactosaminyltransferase 12 OS=Homo sapiens GN=GALNT12 PE=1 SV=3 - [GLT12_HUMAN]	3,27	2		0,505	
Q8TF05	Serine/threonine-protein phosphatase 4 regulatory subunit 1 OS=Homo sapiens GN=PPP4R1 PE=1 SV=1 - [PP4R1_HUMAN]	3,26	2	0,681		
Q8NBP0	Tetratricopeptide repeat protein 13 OS=Homo sapiens GN=TTC13 PE=2 SV=3 - [TTC13_HUMAN]	3,26	2	0,951		
O00471	Exocyst complex component 5 OS=Homo sapiens GN=EXOC5 PE=1 SV=1 - [EXOC5_HUMAN]	3,25	2	0,569	1,006	
Q9BQ70	Transcription factor 25 OS=Homo sapiens GN=TCF25 PE=1 SV=1 - [TCF25_HUMAN]	3,25	2	1,111	0,936	0,802
Q6J4K2	Sodium/potassium/calcium exchanger 6 OS=Homo sapiens GN=SLC24A6 PE=2 SV=2 - [NCKX6_HUMAN]	3,25	2	1,292	1,185	
Q9BVK2	Probable dolichyl pyrophosphate Glc1Man9GlcNAc2 alpha-1,3-glucosyltransferase OS=Homo sapiens GN=ALG8 PE=1 SV=2 - [ALG8_HUM	3,23	2		1,113	
Q9H269	Vacuolar protein sorting-associated protein 16 homolog OS=Homo sapiens GN=VPS16 PE=1 SV=2 - [VPS16_HUMAN]	3,22	2		0,734	
Q7Z478	ATP-dependent RNA helicase DHX29 OS=Homo sapiens GN=DHX29 PE=1 SV=2 - [DHX29_HUMAN]	3,21	2			0,894
Q9NP80	Calcium-independent phospholipase A2-gamma OS=Homo sapiens GN=PNPLA8 PE=1 SV=1 - [PLPL8_HUMAN]	3,20	2	0,988	0,926	
Q96AX1	Vacuolar protein sorting-associated protein 33A OS=Homo sapiens GN=VPS33A PE=1 SV=1 - [VP33A_HUMAN]	3,19	2			
Q13427	Peptidyl-prolyl cis-trans isomerase G OS=Homo sapiens GN=PPIG PE=1 SV=2 - [PPIG_HUMAN]	3,18	2			1,330
P52848	Bifunctional heparan sulfate N-deacetylase/N-sulfotransferase 1 OS=Homo sapiens GN=NDST1 PE=1 SV=1 - [NDST1_HUMAN]	3,17	2	1,549		
Q15269	Periodic tryptophan protein 2 homolog OS=Homo sapiens GN=PWP2 PE=1 SV=2 - [PWP2_HUMAN]	3,16	2	0,678	0,728	

Q9NRA8	Eukaryotic translation initiation factor 4E transporter OS=Homo sapiens GN=EIF4ENIF1 PE=1 SV=2 - [4ET_HUMAN]	3,15	2
P48506	Glutamate--cysteine ligase catalytic subunit OS=Homo sapiens GN=GCLC PE=1 SV=2 - [GSH1_HUMAN]	3,14	2
Q14202	Zinc finger MYM-type protein 3 OS=Homo sapiens GN=ZMYM3 PE=1 SV=2 - [ZMYM3_HUMAN]	3,14	2
Q9UKV8	Protein argonaute-2 OS=Homo sapiens GN=EIF2C2 PE=1 SV=3 - [AGO2_HUMAN]	3,14	2
Q9H329	Band 4.1-like protein 4B OS=Homo sapiens GN=EPB41L4B PE=2 SV=2 - [E41LB_HUMAN]	3,11	2
P46063	ATP-dependent DNA helicase Q1 OS=Homo sapiens GN=RECQL PE=1 SV=3 - [RECQ1_HUMAN]	3,08	2
Q86YV5	Tyrosine-protein kinase SgK223 OS=Homo sapiens GN=SGK223 PE=1 SV=3 - [SG223_HUMAN]	3,07	2
Q9HD43	Receptor-type tyrosine-protein phosphatase H OS=Homo sapiens GN=PTPRH PE=1 SV=3 - [PTPRH_HUMAN]	3,05	2
Q9ULH7	MKL/myocardin-like protein 2 OS=Homo sapiens GN=MKL2 PE=1 SV=3 - [MKL2_HUMAN]	3,03	2
Q9NXF1	Testis-expressed sequence 10 protein OS=Homo sapiens GN=TEX10 PE=1 SV=2 - [TEX10_HUMAN]	3,01	2
Q92615	La-related protein 4B OS=Homo sapiens GN=LARP4B PE=1 SV=3 - [LAR4B_HUMAN]	2,98	2
P51812	Ribosomal protein S6 kinase alpha-3 OS=Homo sapiens GN=RPS6KA3 PE=1 SV=1 - [KS6A3_HUMAN]	2,97	2
Q86UT6	NLR family member X1 OS=Homo sapiens GN=NLRX1 PE=1 SV=1 - [NLRX1_HUMAN]	2,97	2
P29083	General transcription factor IIE subunit 1 OS=Homo sapiens GN=GTF2E1 PE=1 SV=2 - [T2EA_HUMAN]	2,96	2
Q86UU0	B-cell CLL/lymphoma 9-like protein OS=Homo sapiens GN=BCL9L PE=1 SV=1 - [BCL9L_HUMAN]	2,94	2
P00519	Tyrosine-protein kinase ABL1 OS=Homo sapiens GN=ABL1 PE=1 SV=4 - [ABL1_HUMAN]	2,92	2
O00219	Hyaluronan synthase 3 OS=Homo sapiens GN=HAS3 PE=1 SV=3 - [HAS3_HUMAN]	2,89	2
Q04656	Copper-transporting ATPase 1 OS=Homo sapiens GN=ATP7A PE=1 SV=3 - [ATP7A_HUMAN]	2,87	2
Q8N4C8	Misshapen-like kinase 1 OS=Homo sapiens GN=MINK1 PE=1 SV=2 - [MINK1_HUMAN]	2,85	2
Q9UPN9	E3 ubiquitin-protein ligase TRIM33 OS=Homo sapiens GN=TRIM33 PE=1 SV=3 - [TRI33_HUMAN]	2,84	2
B8ZZT9	Sorting nexin 13, isoform CRA_g OS=Homo sapiens GN=SNX13 PE=4 SV=1 - [B8ZZT9_HUMAN]	2,82	2
Q4LE39	AT-rich interactive domain-containing protein 4B OS=Homo sapiens GN=ARID4B PE=1 SV=2 - [ARI4B_HUMAN]	2,82	2
Q70E73	Ras-associated and pleckstrin homology domains-containing protein 1 OS=Homo sapiens GN=RAPH1 PE=1 SV=3 - [RAPH1_HUMAN]	2,80	2
E9PGN5	Inter-alpha-trypsin inhibitor heavy chain H4 OS=Homo sapiens GN=ITIH4 PE=4 SV=1 - [E9PGN5_HUMAN]	2,78	2
O95479	GDH/6PGL endoplasmic bifunctional protein OS=Homo sapiens GN=H6PD PE=1 SV=2 - [G6PE_HUMAN]	2,78	2
P19021	Peptidyl-glycine alpha-amidating monooxygenase OS=Homo sapiens GN=PAM PE=1 SV=2 - [AMD_HUMAN]	2,77	2
G3V1C4	Cell division cycle 27, isoform CRA_b OS=Homo sapiens GN=CDC27 PE=4 SV=1 - [G3V1C4_HUMAN]	2,77	2
Q13625	Apoptosis-stimulating of p53 protein 2 OS=Homo sapiens GN=TP53BP2 PE=1 SV=2 - [ASPP2_HUMAN]	2,75	2
Q13206	Probable ATP-dependent RNA helicase DDX10 OS=Homo sapiens GN=DDX10 PE=1 SV=2 - [DDX10_HUMAN]	2,74	2
Q6IE81	Protein Jade-1 OS=Homo sapiens GN=PHF17 PE=1 SV=1 - [JADE1_HUMAN]	2,73	2
Q8ND04	Protein SMG8 OS=Homo sapiens GN=SMG8 PE=1 SV=1 - [SMG8_HUMAN]	2,72	2
Q9BZJ0	Crooked neck-like protein 1 OS=Homo sapiens GN=CRNKL1 PE=1 SV=4 - [CRNL1_HUMAN]	2,71	2
P23142	Fibulin-1 OS=Homo sapiens GN=FBLN1 PE=1 SV=4 - [FBLN1_HUMAN]	2,70	2
Q9UHW9	Solute carrier family 12 member 6 OS=Homo sapiens GN=SLC12A6 PE=1 SV=2 - [S12A6_HUMAN]	2,70	2
Q6UXY8	Transmembrane channel-like protein 5 OS=Homo sapiens GN=TMC5 PE=2 SV=3 - [TMC5_HUMAN]	2,68	2
Q9BST9	Rhotekin OS=Homo sapiens GN=RTKN PE=1 SV=2 - [RTKN_HUMAN]	2,66	2
Q7Z736	Pleckstrin homology domain-containing family H member 3 OS=Homo sapiens GN=PLEKHH3 PE=1 SV=2 - [PKHH3_HUMAN]	2,65	2
Q8NHU6	Tudor domain-containing protein 7 OS=Homo sapiens GN=TDRD7 PE=1 SV=2 - [TDRD7_HUMAN]	2,64	2
Q9H3S7	Tyrosine-protein phosphatase non-receptor type 23 OS=Homo sapiens GN=PTPN23 PE=1 SV=1 - [PTN23_HUMAN]	2,63	2
P11274	Breakpoint cluster region protein OS=Homo sapiens GN=BCR PE=1 SV=2 - [BCR_HUMAN]	2,60	2
Q6VY07	Phosphofurin acidic cluster sorting protein 1 OS=Homo sapiens GN=PACS1 PE=1 SV=2 - [PACS1_HUMAN]	2,60	2
Q9Y4C8	Probable RNA-binding protein 19 OS=Homo sapiens GN=RBM19 PE=1 SV=3 - [RBM19_HUMAN]	2,60	2
Q07617	Sperm-associated antigen 1 OS=Homo sapiens GN=SPAG1 PE=1 SV=3 - [SPAG1_HUMAN]	2,59	2
Q9UHI6	Probable ATP-dependent RNA helicase DDX20 OS=Homo sapiens GN=DDX20 PE=1 SV=2 - [DDX20_HUMAN]	2,55	2
Q9UNX4	WD repeat-containing protein 3 OS=Homo sapiens GN=WDR3 PE=1 SV=1 - [WDR3_HUMAN]	2,55	2
Q9BXL7	Caspase recruitment domain-containing protein 11 OS=Homo sapiens GN=CARD11 PE=1 SV=3 - [CAR11_HUMAN]	2,51	2
Q9H1N7	Adenosine 3'-phospho 5'-phosphosulfate transporter 2 OS=Homo sapiens GN=SLC35B3 PE=1 SV=1 - [S35B3_HUMAN]	2,49	2
Q96KG9	N-terminal kinase-like protein OS=Homo sapiens GN=SCYL1 PE=1 SV=1 - [NTKL_HUMAN]	2,48	2
Q96H55	Unconventional myosin-XIX OS=Homo sapiens GN=MYO19 PE=2 SV=2 - [MYO19_HUMAN]	2,47	2
P85037	Forkhead box protein K1 OS=Homo sapiens GN=FOXK1 PE=1 SV=1 - [FOXK1_HUMAN]	2,46	2
Q14562	ATP-dependent RNA helicase DHX8 OS=Homo sapiens GN=DHX8 PE=1 SV=1 - [DHX8_HUMAN]	2,46	2
P51788	Chloride channel protein 2 OS=Homo sapiens GN=CLCN2 PE=1 SV=2 - [CLCN2_HUMAN]	2,45	2
Q13608	Peroxisome assembly factor 2 OS=Homo sapiens GN=PEX6 PE=1 SV=2 - [PEX6_HUMAN]	2,45	2
Q9BZL4	Protein phosphatase 1 regulatory subunit 12C OS=Homo sapiens GN=PPP1R12C PE=1 SV=1 - [PP12C_HUMAN]	2,43	2
Q13620	Cullin-4B OS=Homo sapiens GN=CUL4B PE=1 SV=4 - [CUL4B_HUMAN]	2,41	2
O95490	Latrophilin-2 OS=Homo sapiens GN=LPHN2 PE=1 SV=2 - [LPHN2_HUMAN]	2,40	2
P35670	Copper-transporting ATPase 2 OS=Homo sapiens GN=ATP7B PE=1 SV=4 - [ATP7B_HUMAN]	2,39	2
O43909	Exostosin-like 3 OS=Homo sapiens GN=EXTL3 PE=2 SV=1 - [EXTL3_HUMAN]	2,39	2
Q13563	Polycystin-2 OS=Homo sapiens GN=PKD2 PE=1 SV=3 - [PKD2_HUMAN]	2,38	2
P28290	Sperm-specific antigen 2 OS=Homo sapiens GN=SSFA2 PE=1 SV=3 - [SSFA2_HUMAN]	2,38	2
Q13464	Rho-associated protein kinase 1 OS=Homo sapiens GN=ROCK1 PE=1 SV=1 - [ROCK1_HUMAN]	2,36	2
O94985	Calsyntenin-1 OS=Homo sapiens GN=CLSTN1 PE=1 SV=1 - [CSTN1_HUMAN]	2,34	2
Q9Y2L1	Exosome complex exonuclease RRP44 OS=Homo sapiens GN=DIS3 PE=1 SV=2 - [RRP44_HUMAN]	2,30	2
Q9UIA9	Exportin-7 OS=Homo sapiens GN=XPO7 PE=1 SV=3 - [XPO7_HUMAN]	2,30	2
Q6IN85	Serine/threonine-protein phosphatase 4 regulatory subunit 3A OS=Homo sapiens GN=SMEK1 PE=1 SV=1 - [P4R3A_HUMAN]	2,28	2
Q14644	Ras GTPase-activating protein 3 OS=Homo sapiens GN=RASA3 PE=1 SV=3 - [RASA3_HUMAN]	2,28	2
Q96RN5	Mediator of RNA polymerase II transcription subunit 15 OS=Homo sapiens GN=MED15 PE=1 SV=2 - [MED15_HUMAN]	2,28	2
Q8IWC1	MAP7 domain-containing protein 3 OS=Homo sapiens GN=MAP7D3 PE=1 SV=2 - [MA7D3_HUMAN]	2,28	2
Q15643	Thyroid receptor-interacting protein 11 OS=Homo sapiens GN=TRIP11 PE=1 SV=3 - [TRIPB_HUMAN]	2,27	2
Q6ZS17	Protein FAM65A OS=Homo sapiens GN=FAM65A PE=1 SV=1 - [FA65A_HUMAN]	2,21	2
Q12772	Sterol regulatory element-binding protein 2 OS=Homo sapiens GN=SREBF2 PE=1 SV=2 - [SRBP2_HUMAN]	2,19	2
Q9Y5B0	RNA polymerase II subunit A C-terminal domain phosphatase OS=Homo sapiens GN=CTDP1 PE=1 SV=3 - [CTDP1_HUMAN]	2,19	2
C9JKF1	Sterile alpha motif domain-containing protein 9 (Fragment) OS=Homo sapiens GN=SAMD9 PE=4 SV=1 - [C9JKF1_HUMAN]	2,18	2
Q9Y4B6-3	Isoform 3 of Protein VPRBP OS=Homo sapiens GN=VPRBP - [VPRBP_HUMAN]	2,17	2
Q14004	Cyclin-dependent kinase 13 OS=Homo sapiens GN=CDK13 PE=1 SV=2 - [CDK13_HUMAN]	2,12	2
Q13619	Cullin-4A OS=Homo sapiens GN=CUL4A PE=1 SV=3 - [CUL4A_HUMAN]	2,11	2
Q9Y4C2	Protein FAM115A OS=Homo sapiens GN=FAM115A PE=1 SV=3 - [F115A_HUMAN]	2,06	2
O60229	Kalirin OS=Homo sapiens GN=KALRN PE=1 SV=2 - [KALRN_HUMAN]	2,01	2
Q53SF7	Cordon-bleu protein-like 1 OS=Homo sapiens GN=COBLL1 PE=1 SV=2 - [COBL1_HUMAN]	1,99	2
Q96KQ7	Histone-lysine N-methyltransferase EHMT2 OS=Homo sapiens GN=EHMT2 PE=1 SV=3 - [EHMT2_HUMAN]	1,98	2
P22413	Ectonucleotide pyrophosphatase/phosphodiesterase family member 1 OS=Homo sapiens GN=ENPP1 PE=1 SV=2 - [ENPP1_HUMAN]	1,95	2
Q5T3U5	Multidrug resistance-associated protein 7 OS=Homo sapiens GN=ABCC10 PE=1 SV=1 - [MRP7_HUMAN]	1,94	2
Q15678	Tyrosine-protein phosphatase non-receptor type 14 OS=Homo sapiens GN=PTPN14 PE=1 SV=2 - [PTN14_HUMAN]	1,94	2
Q9BX63	Fanconi anemia group J protein OS=Homo sapiens GN=BRIP1 PE=1 SV=1 - [FANCJ_HUMAN]	1,92	2
O14617	AP-3 complex subunit delta-1 OS=Homo sapiens GN=AP3D1 PE=1 SV=1 - [AP3D1_HUMAN]	1,91	2
P53355	Death-associated protein kinase 1 OS=Homo sapiens GN=DAPK1 PE=1 SV=6 - [DAPK1_HUMAN]	1,89	2
O94851	Protein-methionine sulfoxide oxidase MICAL2 OS=Homo sapiens GN=MICAL2 PE=1 SV=1 - [MICA2_HUMAN]	1,87	2
Q10570	Cleavage and polyadenylation specificity factor subunit 1 OS=Homo sapiens GN=CPSF1 PE=1 SV=2 - [CPSF1_HUMAN]	1,87	2
A2VDJ0	Transmembrane protein 131-like OS=Homo sapiens GN=KIAA0922 PE=1 SV=2 - [T131L_HUMAN]	1,86	2
Q9UKJ3	G patch domain-containing protein 8 OS=Homo sapiens GN=GPATCH8 PE=1 SV=2 - [GPTC8_HUMAN]	1,86	2
Q9Y6X9	MORC family CW-type zinc finger protein 2 OS=Homo sapiens GN=MORC2 PE=1 SV=2 - [MORC2_HUMAN]	1,84	2
O60231	Putative pre-mRNA-splicing factor ATP-dependent RNA helicase DHX16 OS=Homo sapiens GN=DHX16 PE=1 SV=2 - [DHX16_HUMAN]	1,83	2
Q93100	Phosphorylase b kinase regulatory subunit beta OS=Homo sapiens GN=PHKB PE=1 SV=3 - [KPBB_HUMAN]	1,83	2
O96028	Probable histone-lysine N-methyltransferase NSD2 OS=Homo sapiens GN=WHSC1 PE=1 SV=1 - [NSD2_HUMAN]	1,83	2
O95248-4	Isoform 4 of Myotubularin-related protein 5 OS=Homo sapiens GN=SBF1 - [MTMR5_HUMAN]	1,80	2
Q9HCE1	Putative helicase MOV-10 OS=Homo sapiens GN=MOV10 PE=1 SV=2 - [MOV10_HUMAN]	1,79	2
Q9P2E3	NFX1-type zinc finger-containing protein 1 OS=Homo sapiens GN=ZNFX1 PE=1 SV=2 - [ZNFX1_HUMAN]	1,77	2
Q99570	Phosphoinositide 3-kinase regulatory subunit 4 OS=Homo sapiens GN=PIK3R4 PE=1 SV=3 - [PI3R4_HUMAN]	1,77	2
Q15714	TSC22 domain family protein 1 OS=Homo sapiens GN=TSC22D1 PE=1 SV=3 - [T22D1_HUMAN]	1,77	2
Q9ULM3	YEATS domain-containing protein 2 OS=Homo sapiens GN=YEATS2 PE=1 SV=2 - [YETS2_HUMAN]	1,76	2
P49917	DNA ligase 4 OS=Homo sapiens GN=LIG4 PE=1 SV=2 - [DNLI4_HUMAN]	1,76	2
Q52LW3	Rho GTPase-activating protein 29 OS=Homo sapiens GN=ARHGAP29 PE=1 SV=2 - [RHG29_HUMAN]	1,74	2
O95163	Elongator complex protein 1 OS=Homo sapiens GN=IKBKAP PE=1 SV=3 - [ELP1_HUMAN]	1,73	2
E9PMR6	Rho guanine nucleotide exchange factor 12 OS=Homo sapiens GN=ARHGEF12 PE=4 SV=1 - [E9PMR6_HUMAN]	1,73	2
Q5JSZ5	Protein PRRC2B OS=Homo sapiens GN=PRRC2B PE=1 SV=2 - [PRC2B_HUMAN]	1,70	2
Q6T4R5	Nance-Horan syndrome protein OS=Homo sapiens GN=NHS PE=1 SV=1 - [NHS_HUMAN]	1,66	2
P01024	Complement C3 OS=Homo sapiens GN=C3 PE=1 SV=2 - [CO3_HUMAN]	1,62	2
Q03701	CCAAT/enhancer-binding protein zeta OS=Homo sapiens GN=CEBPZ PE=1 SV=3 - [CEBPZ_HUMAN]	1,61	2
O14646-2	Isoform 2 of Chromodomain-helicase-DNA-binding protein 1 OS=Homo sapiens GN=CHD1 - [CHD1_HUMAN]	1,58	2
Q96QT4	Transient receptor potential cation channel subfamily M member 7 OS=Homo sapiens GN=TRPM7 PE=1 SV=1 - [TRPM7_HUMAN]	1,50	2
Q5T5Y3	Calmodulin-regulated spectrin-associated protein 1 OS=Homo sapiens GN=CAMSAP1 PE=1 SV=2 - [CAMP1_HUMAN]	1,50	2
Q9C0D5-2	Isoform 2 of Protein TANC1 OS=Homo sapiens GN=TANC1 - [TANC1_HUMAN]	1,48	2
Q92793	CREB-binding protein OS=Homo sapiens GN=CREBBP PE=1 SV=3 - [CBP_HUMAN]	1,47	2
Q92614	Unconventional myosin-XVIIIa OS=Homo sapiens GN=MYO18A PE=1 SV=3 - [MY18A_HUMAN]	1,46	2
Q8NI27	THO complex subunit 2 OS=Homo sapiens GN=THOC2 PE=1 SV=2 - [THOC2_HUMAN]	1,44	2
Q15154	Pericentriolar material 1 protein OS=Homo sapiens GN=PCM1 PE=1 SV=4 - [PCM1_HUMAN]	1,43	2
Q92797	Symplekin OS=Homo sapiens GN=SYMPK PE=1 SV=2 - [SYMPK_HUMAN]	1,41	2
P06400	Retinoblastoma-associated protein OS=Homo sapiens GN=RB1 PE=1 SV=2 - [RB_HUMAN]	1,40	2
H7BZ93	Histone-lysine N-methyltransferase SETD2 (Fragment) OS=Homo sapiens GN=SETD2 PE=4 SV=1 - [H7BZ93_HUMAN]	1,40	2
Q5VUA4	Zinc finger protein 318 OS=Homo sapiens GN=ZNF318 PE=1 SV=2 - [ZN318_HUMAN]	1,40	2
Q9NYU1	UDP-glucose:glycoprotein glucosyltransferase 2 OS=Homo sapiens GN=UGGT2 PE=1 SV=4 - [UGGG2_HUMAN]	1,39	2
Q7Z4S6	Kinesin-like protein KIF21A OS=Homo sapiens GN=KIF21A PE=1 SV=2 - [KI21A_HUMAN]	1,37	2
Q8N201	Integrator complex subunit 1 OS=Homo sapiens GN=INTS1 PE=1 SV=2 - [INT1_HUMAN]	1,37	2
Q08378	Golgin subfamily A member 3 OS=Homo sapiens GN=GOLGA3 PE=1 SV=2 - [GOGA3_HUMAN]	1,34	2
Q6PGP7	Tetratricopeptide repeat protein 37 OS=Homo sapiens GN=TTC37 PE=1 SV=1 - [TTC37_HUMAN]	1,34	2
Q05397	Focal adhesion kinase 1 OS=Homo sapiens GN=PTK2 PE=1 SV=2 - [FAK1_HUMAN]	1,33	2
Q99715-2	Isoform 2 of Collagen alpha-1(XII) chain OS=Homo sapiens GN=COL12A1 - [COCA1_HUMAN]	1,32	2
Q9BZH6	WD repeat-containing protein 11 OS=Homo sapiens GN=WDR11 PE=1 SV=1 - [WDR11_HUMAN]	1,31	2
Q96BY6	Dedicator of cytokinesis protein 10 OS=Homo sapiens GN=DOCK10 PE=1 SV=3 - [DOC10_HUMAN]	1,28	2
J3KP19	Signal-induced proliferation-associated 1-like protein 1 OS=Homo sapiens GN=SIPA1L1 PE=4 SV=1 - [J3KP19_HUMAN]	1,23	2
Q9Y618	Nuclear receptor corepressor 2 OS=Homo sapiens GN=NCOR2 PE=1 SV=2 - [NCOR2_HUMAN]	1,23	2
Q5TAX3	Terminal uridylyltransferase 4 OS=Homo sapiens GN=ZCCHC11 PE=1 SV=3 - [TUT4_HUMAN]	1,22	2
Q15811	Intersectin-1 OS=Homo sapiens GN=ITSN1 PE=1 SV=3 - [ITSN1_HUMAN]	1,22	2
Q8NFD5	AT-rich interactive domain-containing protein 1B OS=Homo sapiens GN=ARID1B PE=1 SV=2 - [ARI1B_HUMAN]	1,21	2
Q9UPU5	Ubiquitin carboxyl-terminal hydrolase 24 OS=Homo sapiens GN=USP24 PE=1 SV=3 - [UBP24_HUMAN]	1,18	2
Q5T5P2	Sickle tail protein homolog OS=Homo sapiens GN=SKT PE=1 SV=2 - [SKT_HUMAN]	1,18	2
O15013	Rho guanine nucleotide exchange factor 10 OS=Homo sapiens GN=ARHGEF10 PE=1 SV=4 - [ARHGA_HUMAN]	1,17	2
P35573	Glycogen debranching enzyme OS=Homo sapiens GN=AGL PE=1 SV=3 - [GDE_HUMAN]	1,17	2
Q9HCK8	Chromodomain-helicase-DNA-binding protein 8 OS=Homo sapiens GN=CHD8 PE=1 SV=5 - [CHD8_HUMAN]	1,16	2
Q7Z2Y5	Nik-related protein kinase OS=Homo sapiens GN=NRK PE=2 SV=2 - [NRK_HUMAN]	1,14	2
P09884	DNA polymerase alpha catalytic subunit OS=Homo sapiens GN=POLA1 PE=1 SV=2 - [DPOLA_HUMAN]	1,09	2
P50748	Kinetochore-associated protein 1 OS=Homo sapiens GN=KNTC1 PE=1 SV=1 - [KNTC1_HUMAN]	1,00	2
Q8IZQ1	WD repeat and FYVE domain-containing protein 3 OS=Homo sapiens GN=WDFY3 PE=1 SV=2 - [WDFY3_HUMAN]	0,99	2
Q68DK2	Zinc finger FYVE domain-containing protein 26 OS=Homo sapiens GN=ZFYVE26 PE=1 SV=3 - [ZFY26_HUMAN]	0,95	2
P25391	Laminin subunit alpha-1 OS=Homo sapiens GN=LAMA1 PE=1 SV=2 - [LAMA1_HUMAN]	0,88	2
Q92576	PHD finger protein 3 OS=Homo sapiens GN=PHF3 PE=1 SV=3 - [PHF3_HUMAN]	0,83	2
Q15652	Probable JmjC domain-containing histone demethylation protein 2C OS=Homo sapiens GN=JMJD1C PE=1 SV=2 - [JHD2C_HUMAN]	0,79	2
Q93008	Probable ubiquitin carboxyl-terminal hydrolase FAF-X OS=Homo sapiens GN=USP9X PE=1 SV=3 - [USP9X_HUMAN]	0,78	2
A2RRP1	Neuroblastoma-amplified sequence OS=Homo sapiens GN=NBAS PE=1 SV=2 - [NBAS_HUMAN]	0,76	2
Q7Z333	Probable helicase senataxin OS=Homo sapiens GN=SETX PE=1 SV=4 - [SETX_HUMAN]	0,75	2
Q3V6T2	Girdin OS=Homo sapiens GN=CCDC88A PE=1 SV=2 - [GRDN_HUMAN]	0,75	2
P25054	Adenomatous polyposis coli protein OS=Homo sapiens GN=APC PE=1 SV=2 - [APC_HUMAN]	0,53	2
J3QK84	Nebulin OS=Homo sapiens GN=NEB PE=4 SV=1 - [J3QK84_HUMAN]	0,28	2
C9JNG5	Protein disulfide-isomerase A6 (Fragment) OS=Homo sapiens GN=PDIA6 PE=4 SV=1 - [C9JNG5_HUMAN]	100,00	1
Q99880	Histone H2B type 1-L OS=Homo sapiens GN=HIST1H2BL PE=1 SV=3 - [H2B1L_HUMAN]	78,57	1
Q5QNW6	Histone H2B type 2-F OS=Homo sapiens GN=HIST2H2BF PE=1 SV=3 - [H2B2F_HUMAN]	76,98	1
O60814	Histone H2B type 1-K OS=Homo sapiens GN=HIST1H2BK PE=1 SV=3 - [H2B1K_HUMAN]	76,98	1
P58876	Histone H2B type 1-D OS=Homo sapiens GN=HIST1H2BD PE=1 SV=2 - [H2B1D_HUMAN]	76,98	1
P33778	Histone H2B type 1-B OS=Homo sapiens GN=HIST1H2BB PE=1 SV=2 - [H2B1B_HUMAN]	76,98	1
H0YKX9	Annexin (Fragment) OS=Homo sapiens GN=ANXA2 PE=3 SV=1 - [H0YKX9_HUMAN]	75,37	1
E9PKF3	Acetyl-CoA acetyltransferase, mitochondrial (Fragment) OS=Homo sapiens GN=ACAT1 PE=3 SV=1 - [E9PKF3_HUMAN]	62,96	1
H9KVC3	Interferon-induced GTP-binding protein Mx1 (Fragment) OS=Homo sapiens GN=MX1 PE=4 SV=1 - [H9KVC3_HUMAN]	61,54	1
O60361	Putative nucleoside diphosphate kinase OS=Homo sapiens GN=NME2P1 PE=5 SV=1 - [NDK8_HUMAN]	59,85	1
P15531	Nucleoside diphosphate kinase A OS=Homo sapiens GN=NME1 PE=1 SV=1 - [NDKA_HUMAN]	59,21	1
P62987	Ubiquitin-60S ribosomal protein L40 OS=Homo sapiens GN=UBA52 PE=1 SV=2 - [RL40_HUMAN]	58,59	1
P35613-3	Isoform 3 of Basigin OS=Homo sapiens GN=BSG - [BASI_HUMAN]	56,82	1
F5H867	4F2 cell-surface antigen heavy chain (Fragment) OS=Homo sapiens GN=SLC3A2 PE=4 SV=1 - [F5H867_HUMAN]	54,81	1
H7BZJ3	Thioredoxin (Fragment) OS=Homo sapiens GN=PDIA3 PE=3 SV=1 - [H7BZJ3_HUMAN]	54,47	1
F8VXV7	Periphilin-1 (Fragment) OS=Homo sapiens GN=PPHLN1 PE=4 SV=1 - [F8VXV7_HUMAN]	54,05	1
P01111	GTPase NRas OS=Homo sapiens GN=NRAS PE=1 SV=1 - [RASN_HUMAN]	52,38	1

D6RB85	Calnexin (Fragment) OS=Homo sapiens GN=CANX PE=3 SV=1 - [D6RB85_HUMAN]	52,08	1	[illegible]
F8WES2	S-methyl-5'-thioadenosine phosphorylase OS=Homo sapiens GN=MTAP PE=4 SV=1 - [F8WES2_HUMAN]	52,00	1	[illegible]
Q96KK5	Histone H2A type 1-H OS=Homo sapiens GN=HIST1H2AH PE=1 SV=3 - [H2A1H_HUMAN]	50,78	1	[illegible]
Q93077	Histone H2A type 1-C OS=Homo sapiens GN=HIST1H2AC PE=1 SV=3 - [H2A1C_HUMAN]	50,00	1	[illegible]
P01112	GTPase HRas OS=Homo sapiens GN=HRAS PE=1 SV=1 - [RASH_HUMAN]	49,74	1	[illegible]
F8W7C6	60S ribosomal protein L10 OS=Homo sapiens GN=RPL10 PE=4 SV=1 - [F8W7C6_HUMAN]	49,08	1	[illegible]
H0YE97	Phosphatidylinositol-binding clathrin assembly protein (Fragment) OS=Homo sapiens GN=PICALM PE=4 SV=1 - [H0YE97_HUMAN]	47,76	1	[illegible]
Q29963	HLA class I histocompatibility antigen, Cw-6 alpha chain OS=Homo sapiens GN=HLA-C PE=1 SV=2 - [1C06_HUMAN]	46,72	1	[illegible]
P12236	ADP/ATP translocase 3 OS=Homo sapiens GN=SLC25A6 PE=1 SV=4 - [ADT3_HUMAN]	45,97	1	[illegible]
Q15366-4	Isoform 4 of Poly(rC)-binding protein 2 OS=Homo sapiens GN=PCBP2 - [PCBP2_HUMAN]	45,62	1	[illegible]
P62979	Ubiquitin-40S ribosomal protein S27a OS=Homo sapiens GN=RPS27A PE=1 SV=2 - [RS27A_HUMAN]	45,51	1	[illegible]
H7C3W8	Leucine-rich PPR motif-containing protein, mitochondrial (Fragment) OS=Homo sapiens GN=LRPPRC PE=4 SV=1 - [H7C3W8_HUMAN]	44,16	1	[illegible]
J3QLN5	Vascular endothelial zinc finger 1 (Fragment) OS=Homo sapiens GN=VEZF1 PE=4 SV=1 - [J3QLN5_HUMAN]	43,75	1	[illegible]
P06753	Tropomyosin alpha-3 chain OS=Homo sapiens GN=TPM3 PE=1 SV=1 - [TPM3_HUMAN]	42,96	1	[illegible]
C9JZG2	Phosphoribosylamine--glycine ligase (Fragment) OS=Homo sapiens GN=GART PE=4 SV=1 - [C9JZG2_HUMAN]	42,65	1	[illegible]
P20290-2	Isoform 2 of Transcription factor BTF3 OS=Homo sapiens GN=BTF3 - [BTF3_HUMAN]	42,59	1	[illegible]
F8VVQ7	LIM domain and actin-binding protein 1 (Fragment) OS=Homo sapiens GN=LIMA1 PE=4 SV=1 - [F8VVQ7_HUMAN]	42,34	1	[illegible]
I6L8F9	Poly(rC)-binding protein 2 OS=Homo sapiens GN=PCBP2 PE=4 SV=1 - [I6L8F9_HUMAN]	41,55	1	[illegible]
P20290	Transcription factor BTF3 OS=Homo sapiens GN=BTF3 PE=1 SV=1 - [BTF3_HUMAN]	40,29	1	[illegible]
Q9UHB6-4	Isoform 4 of LIM domain and actin-binding protein 1 OS=Homo sapiens GN=LIMA1 - [LIMA1_HUMAN]	40,26	1	[illegible]
Q9UHB6	LIM domain and actin-binding protein 1 OS=Homo sapiens GN=LIMA1 PE=1 SV=1 - [LIMA1_HUMAN]	40,18	1	[illegible]
Q15836	Vesicle-associated membrane protein 3 OS=Homo sapiens GN=VAMP3 PE=1 SV=3 - [VAMP3_HUMAN]	40,00	1	[illegible]
F5H1N1	DnaJ homolog subfamily C member 11 OS=Homo sapiens GN=DNAJC11 PE=4 SV=1 - [F5H1N1_HUMAN]	39,92	1	[illegible]
Q5T9W8	Heat shock protein 90kDa alpha (Cytosolic), class B member 1 (Fragment) OS=Homo sapiens GN=HSP90AB1 PE=2 SV=1 - [Q5T9W8_H	38,69	1	[illegible]
P24844	Myosin regulatory light polypeptide 9 OS=Homo sapiens GN=MYL9 PE=1 SV=4 - [MYL9_HUMAN]	38,37	1	[illegible]
P30501	HLA class I histocompatibility antigen, Cw-2 alpha chain OS=Homo sapiens GN=HLA-C PE=1 SV=1 - [1C02_HUMAN]	38,25	1	[illegible]
H7BZ18	Multiple coagulation factor deficiency protein 2 (Fragment) OS=Homo sapiens GN=MCFD2 PE=4 SV=1 - [H7BZ18_HUMAN]	37,78	1	[illegible]
D6RFI0	Sideroflexin-1 (Fragment) OS=Homo sapiens GN=SFXN1 PE=4 SV=1 - [D6RFI0_HUMAN]	37,63	1	[illegible]
Q9NVH1	DnaJ homolog subfamily C member 11 OS=Homo sapiens GN=DNAJC11 PE=1 SV=2 - [DJC11_HUMAN]	37,57	1	[illegible]
Q8WUD1	Ras-related protein Rab-2B OS=Homo sapiens GN=RAB2B PE=1 SV=1 - [RAB2B_HUMAN]	37,50	1	[illegible]
Q8N2F6-4	Isoform 4 of Armadillo repeat-containing protein 10 OS=Homo sapiens GN=ARMC10 - [ARM10_HUMAN]	37,35	1	[illegible]
P63092	Guanine nucleotide-binding protein G(s) subunit alpha isoforms short OS=Homo sapiens GN=GNAS PE=1 SV=1 - [GNAS2_HUMAN]	36,55	1	[illegible]
P20336	Ras-related protein Rab-3A OS=Homo sapiens GN=RAB3A PE=1 SV=1 - [RAB3A_HUMAN]	36,36	1	[illegible]
P08134	Rho-related GTP-binding protein RhoC OS=Homo sapiens GN=RHOC PE=1 SV=1 - [RHOC_HUMAN]	36,27	1	[illegible]
P63092-2	Isoform Gnas-2 of Guanine nucleotide-binding protein G(s) subunit alpha isoforms short OS=Homo sapiens GN=GNAS - [GNAS2_HUMAN	36,05	1	[illegible]
Q9NVJ2	ADP-ribosylation factor-like protein 8B OS=Homo sapiens GN=ARL8B PE=1 SV=1 - [ARL8B_HUMAN]	36,02	1	[illegible]
Q96BM9	ADP-ribosylation factor-like protein 8A OS=Homo sapiens GN=ARL8A PE=1 SV=1 - [ARL8A_HUMAN]	36,02	1	[illegible]
P63092-3	Isoform 3 of Guanine nucleotide-binding protein G(s) subunit alpha isoforms short OS=Homo sapiens GN=GNAS - [GNAS2_HUMAN]	35,88	1	[illegible]
Q9UNX3	60S ribosomal protein L26-like 1 OS=Homo sapiens GN=RPL26L1 PE=1 SV=1 - [RL26L_HUMAN]	35,86	1	[illegible]
F8W914	Reticulon-4 OS=Homo sapiens GN=RTN4 PE=4 SV=1 - [F8W914_HUMAN]	35,07	1	[illegible]
P63027	Vesicle-associated membrane protein 2 OS=Homo sapiens GN=VAMP2 PE=1 SV=3 - [VAMP2_HUMAN]	34,48	1	[illegible]
Q9BSJ8-2	Isoform 2 of Extended synaptotagmin-1 OS=Homo sapiens GN=ESYT1 - [ESYT1_HUMAN]	34,20	1	[illegible]
H3BPR6	Protein BLOC1S6 (Fragment) OS=Homo sapiens GN=BLOC1S6 PE=4 SV=1 - [H3BPR6_HUMAN]	33,33	1	[illegible]
Q14697	Neutral alpha-glucosidase AB OS=Homo sapiens GN=GANAB PE=1 SV=3 - [GANAB_HUMAN]	32,73	1	[illegible]
P16190	HLA class I histocompatibility antigen, A-33 alpha chain OS=Homo sapiens GN=HLA-A PE=1 SV=3 - [1A33_HUMAN]	32,33	1	[illegible]
Q15149-4	Isoform 4 of Plectin OS=Homo sapiens GN=PLEC - [PLEC_HUMAN]	31,96	1	[illegible]
P07948-2	Isoform 2 of Tyrosine-protein kinase Lyn OS=Homo sapiens GN=LYN - [LYN_HUMAN]	30,35	1	[illegible]
H0YC70	Zinc finger protein 706 (Fragment) OS=Homo sapiens GN=ZNF706 PE=4 SV=1 - [H0YC70_HUMAN]	30,00	1	[illegible]
E5RG25	Ubiquitin-conjugating enzyme E2 W OS=Homo sapiens GN=UBE2W PE=4 SV=1 - [E5RG25_HUMAN]	30,00	1	[illegible]
P07948	Tyrosine-protein kinase Lyn OS=Homo sapiens GN=LYN PE=1 SV=3 - [LYN_HUMAN]	29,30	1	[illegible]
E9PIE4	Mitochondrial carrier homolog 2 (Fragment) OS=Homo sapiens GN=MTCH2 PE=3 SV=1 - [E9PIE4_HUMAN]	29,28	1	[illegible]
E5RG56	Aspartyl/asparaginyl beta-hydroxylase OS=Homo sapiens GN=ASPH PE=4 SV=1 - [E5RG56_HUMAN]	29,05	1	[illegible]
Q01105-2	Isoform 2 of Protein SET OS=Homo sapiens GN=SET - [SET_HUMAN]	28,88	1	[illegible]
F8VUH2	Protein OS-9 OS=Homo sapiens GN=OS9 PE=4 SV=1 - [F8VUH2_HUMAN]	28,62	1	[illegible]
H0YJN8	Maleylacetoacetate isomerase (Fragment) OS=Homo sapiens GN=GSTZ1 PE=4 SV=1 - [H0YJN8_HUMAN]	28,57	1	[illegible]
E9PCX7	NAD(P) transhydrogenase, mitochondrial OS=Homo sapiens GN=NNT PE=4 SV=1 - [E9PCX7_HUMAN]	28,27	1	[illegible]
Q8NI22	Multiple coagulation factor deficiency protein 2 OS=Homo sapiens GN=MCFD2 PE=1 SV=1 - [MCFD2_HUMAN]	28,08	1	[illegible]
Q8N2F6	Armadillo repeat-containing protein 10 OS=Homo sapiens GN=ARMC10 PE=1 SV=1 - [ARM10_HUMAN]	27,70	1	[illegible]
Q9UBQ0-2	Isoform 2 of Vacuolar protein sorting-associated protein 29 OS=Homo sapiens GN=VPS29 - [VPS29_HUMAN]	27,42	1	[illegible]
E9PSH0	CUGBP Elav-like family member 1 (Fragment) OS=Homo sapiens GN=CELF1 PE=4 SV=1 - [E9PSH0_HUMAN]	27,37	1	[illegible]
P62304	Small nuclear ribonucleoprotein E OS=Homo sapiens GN=SNRPE PE=1 SV=1 - [RUXE_HUMAN]	27,17	1	[illegible]
D6R9V5	Protein TMEM155 (Fragment) OS=Homo sapiens GN=TMEM155 PE=4 SV=1 - [D6R9V5_HUMAN]	27,14	1	[illegible]
Q9UDW1	Cytochrome b-c1 complex subunit 9 OS=Homo sapiens GN=UQCR10 PE=1 SV=3 - [QCR9_HUMAN]	26,98	1	[illegible]
Q8WTV0-4	Isoform 4 of Scavenger receptor class B member 1 OS=Homo sapiens GN=SCARB1 - [SCRB1_HUMAN]	26,79	1	[illegible]
H0YIU0	Endoplasmic reticulum mannosyl-oligosaccharide 1,2-alpha-mannosidase (Fragment) OS=Homo sapiens GN=MAN1B1 PE=4 SV=1 - [H0Y	26,61	1	[illegible]
P29353-7	Isoform 7 of SHC-transforming protein 1 OS=Homo sapiens GN=SHC1 - [SHC1_HUMAN]	26,16	1	[illegible]
Q96IX5	Up-regulated during skeletal muscle growth protein 5 OS=Homo sapiens GN=USMG5 PE=1 SV=1 - [USMG5_HUMAN]	25,86	1	[illegible]
C9J0F3	Plastin-1 (Fragment) OS=Homo sapiens GN=PLS1 PE=4 SV=1 - [C9J0F3_HUMAN]	25,83	1	[illegible]
J3KN66	Torsin-1A-interacting protein 1 OS=Homo sapiens GN=TOR1AIP1 PE=4 SV=1 - [J3KN66_HUMAN]	25,54	1	[illegible]
C9J359	Plastin-1 (Fragment) OS=Homo sapiens GN=PLS1 PE=4 SV=1 - [C9J359_HUMAN]	25,21	1	[illegible]
H0Y990	ATP-binding cassette sub-family E member 1 (Fragment) OS=Homo sapiens GN=ABCE1 PE=4 SV=1 - [H0Y990_HUMAN]	25,17	1	[illegible]
O75506	Heat shock factor-binding protein 1 OS=Homo sapiens GN=HSBP1 PE=1 SV=1 - [HSBP1_HUMAN]	25,00	1	[illegible]
Q8NHH9-3	Isoform 3 of Atlastin-2 OS=Homo sapiens GN=ATL2 - [ATLA2_HUMAN]	24,76	1	[illegible]
Q9Y5U9	Immediate early response 3-interacting protein 1 OS=Homo sapiens GN=IER3IP1 PE=1 SV=1 - [IR3IP_HUMAN]	24,39	1	[illegible]
G5E9Z2	Cisplatin resistance related protein CRR9p, isoform CRA_c OS=Homo sapiens GN=CLPTM1L PE=4 SV=1 - [G5E9Z2_HUMAN]	24,39	1	[illegible]
Q9UBQ0	Vacuolar protein sorting-associated protein 29 OS=Homo sapiens GN=VPS29 PE=1 SV=1 - [VPS29_HUMAN]	24,18	1	[illegible]
Q9H1X3-3	Isoform 3 of DnaJ homolog subfamily C member 25 OS=Homo sapiens GN=DNAJC25 - [DJC25_HUMAN]	24,18	1	[illegible]
P60763	Ras-related C3 botulinum toxin substrate 3 OS=Homo sapiens GN=RAC3 PE=1 SV=1 - [RAC3_HUMAN]	23,96	1	[illegible]
P04259	Keratin, type II cytoskeletal 6B OS=Homo sapiens GN=KRT6B PE=1 SV=5 - [K2C6B_HUMAN]	23,94	1	[illegible]
Q6UW78	UPF0723 protein C11orf83 OS=Homo sapiens GN=C11orf83 PE=1 SV=2 - [CK083_HUMAN]	23,66	1	[illegible]
E7ESP4	Integrin alpha-2 OS=Homo sapiens GN=ITGA2 PE=3 SV=1 - [E7ESP4_HUMAN]	23,57	1	[illegible]
Q9Y2S6	Coiled-coil domain-containing protein 72 OS=Homo sapiens GN=CCDC72 PE=1 SV=1 - [CCD72_HUMAN]	23,44	1	[illegible]
O14662-4	Isoform D of Syntaxin-16 OS=Homo sapiens GN=STX16 - [STX16_HUMAN]	23,05	1	[illegible]
I3L470	H(+)/Cl(-) exchange transporter 7 (Fragment) OS=Homo sapiens GN=CLCN7 PE=4 SV=1 - [I3L470_HUMAN]	22,69	1	[illegible]
D6RBR1	Calpastatin (Fragment) OS=Homo sapiens GN=CAST PE=4 SV=1 - [D6RBR1_HUMAN]	22,67	1	[illegible]
D6R973	Protein CDV3 homolog (Fragment) OS=Homo sapiens GN=CDV3 PE=4 SV=1 - [D6R973_HUMAN]	22,64	1	[illegible]
D6RHF4	UDP-glucose 6-dehydrogenase (Fragment) OS=Homo sapiens GN=UGDH PE=4 SV=1 - [D6RHF4_HUMAN]	22,64	1	[illegible]
Q71UM5	40S ribosomal protein S27-like OS=Homo sapiens GN=RPS27L PE=1 SV=3 - [RS27L_HUMAN]	22,62	1	[illegible]
O94925	Glutaminase kidney isoform, mitochondrial OS=Homo sapiens GN=GLS PE=1 SV=1 - [GLSK_HUMAN]	22,57	1	[illegible]
P84157	Matrix-remodeling-associated protein 7 OS=Homo sapiens GN=MXRA7 PE=1 SV=1 - [MXRA7_HUMAN]	22,55	1	[illegible]
P46108-2	Isoform Crk-I of Adapter molecule crk OS=Homo sapiens GN=CRK - [CRK_HUMAN]	22,55	1	[illegible]
P34931	Heat shock 70 kDa protein 1-like OS=Homo sapiens GN=HSPA1L PE=1 SV=2 - [HS71L_HUMAN]	22,46	1	[illegible]
P02538	Keratin, type II cytoskeletal 6A OS=Homo sapiens GN=KRT6A PE=1 SV=3 - [K2C6A_HUMAN]	22,16	1	[illegible]
Q8WTV0	Scavenger receptor class B member 1 OS=Homo sapiens GN=SCARB1 PE=1 SV=1 - [SCRB1_HUMAN]	21,56	1	[illegible]
O14662	Syntaxin-16 OS=Homo sapiens GN=STX16 PE=1 SV=3 - [STX16_HUMAN]	21,23	1	[illegible]
Q8TCT9-2	Isoform 2 of Minor histocompatibility antigen H13 OS=Homo sapiens GN=HM13 - [HM13_HUMAN]	21,13	1	[illegible]
D6RG34	Solute carrier family 35 member B1 (Fragment) OS=Homo sapiens GN=SLC35B1 PE=4 SV=1 - [D6RG34_HUMAN]	21,05	1	[illegible]
H3BTH4	ADP-dependent glucokinase (Fragment) OS=Homo sapiens GN=ADPGK PE=4 SV=1 - [H3BTH4_HUMAN]	21,05	1	[illegible]
Q58FF7	Putative heat shock protein HSP 90-beta-3 OS=Homo sapiens GN=HSP90AB3P PE=5 SV=1 - [H90B3_HUMAN]	20,94	1	[illegible]
J3KS54	Protein flightless-1 homolog OS=Homo sapiens GN=FLII PE=4 SV=1 - [J3KS54_HUMAN]	20,86	1	[illegible]
I3L0Q7	Uncharacterized protein (Fragment) OS=Homo sapiens PE=4 SV=1 - [I3L0Q7_HUMAN]	20,83	1	[illegible]
O60927	Protein phosphatase 1 regulatory subunit 11 OS=Homo sapiens GN=PPP1R11 PE=1 SV=1 - [PP1RB_HUMAN]	20,63	1	[illegible]
Q96GX2	Putative ataxin-7-like protein 3B OS=Homo sapiens GN=ATXN7L3B PE=3 SV=2 - [A7L3B_HUMAN]	20,62	1	[illegible]
P29353	SHC-transforming protein 1 OS=Homo sapiens GN=SHC1 PE=1 SV=4 - [SHC1_HUMAN]	20,58	1	[illegible]
H0Y624	Probable phospholipid-transporting ATPase IH (Fragment) OS=Homo sapiens GN=ATP11A PE=4 SV=1 - [H0Y624_HUMAN]	20,00	1	[illegible]
Q9Y333	U6 snRNA-associated Sm-like protein LSm2 OS=Homo sapiens GN=LSM2 PE=1 SV=1 - [LSM2_HUMAN]	20,00	1	[illegible]
H7BZT5	Zinc finger protein 185 (Fragment) OS=Homo sapiens GN=ZNF185 PE=4 SV=1 - [H7BZT5_HUMAN]	19,91	1	[illegible]
Q6UWS5	Protein PET117 homolog, mitochondrial OS=Homo sapiens GN=UNQ607/PRO1194 PE=2 SV=1 - [PT117_HUMAN]	19,75	1	[illegible]
P62891	60S ribosomal protein L39 OS=Homo sapiens GN=RPL39 PE=2 SV=2 - [RL39_HUMAN]	19,61	1	[illegible]
P84157-2	Isoform 2 of Matrix-remodeling-associated protein 7 OS=Homo sapiens GN=MXRA7 - [MXRA7_HUMAN]	19,41	1	[illegible]
P60059	Protein transport protein Sec61 subunit gamma OS=Homo sapiens GN=SEC61G PE=2 SV=1 - [SC61G_HUMAN]	19,12	1	[illegible]
Q96B49	Mitochondrial import receptor subunit TOM6 homolog OS=Homo sapiens GN=TOMM6 PE=1 SV=1 - [TOM6_HUMAN]	18,92	1	[illegible]
Q01628	Interferon-induced transmembrane protein 3 OS=Homo sapiens GN=IFITM3 PE=1 SV=2 - [IFM3_HUMAN]	18,80	1	[illegible]
B1APQ0	Pitrilysin metallopeptidase 1 (Fragment) OS=Homo sapiens GN=PITRM1 PE=4 SV=1 - [B1APQ0_HUMAN]	18,48	1	[illegible]
P26232-2	Isoform 2 of Catenin alpha-2 OS=Homo sapiens GN=CTNNA2 - [CTNA2_HUMAN]	18,45	1	[illegible]
Q8NHH9	Atlastin-2 OS=Homo sapiens GN=ATL2 PE=1 SV=2 - [ATLA2_HUMAN]	18,35	1	[illegible]
P24666	Low molecular weight phosphotyrosine protein phosphatase OS=Homo sapiens GN=ACP1 PE=1 SV=3 - [PPAC_HUMAN]	18,35	1	[illegible]
D6RAX2	C-terminal-binding protein 1 (Fragment) OS=Homo sapiens GN=CTBP1 PE=3 SV=1 - [D6RAX2_HUMAN]	18,18	1	[illegible]
H3BPP2	COX assembly mitochondrial protein 2 homolog OS=Homo sapiens GN=CMC2 PE=4 SV=1 - [H3BPP2_HUMAN]	18,18	1	[illegible]
P54652	Heat shock-related 70 kDa protein 2 OS=Homo sapiens GN=HSPA2 PE=1 SV=1 - [HSP72_HUMAN]	18,15	1	[illegible]
O95197-3	Isoform 3 of Reticulon-3 OS=Homo sapiens GN=RTN3 - [RTN3_HUMAN]	17,80	1	[illegible]
C9JEF4	Histone deacetylase 6 (Fragment) OS=Homo sapiens GN=HDAC6 PE=4 SV=1 - [C9JEF4_HUMAN]	17,65	1	[illegible]
H7BZK4	Sodium-dependent phosphate transporter 1 (Fragment) OS=Homo sapiens GN=SLC20A1 PE=4 SV=1 - [H7BZK4_HUMAN]	17,37	1	[illegible]
Q9BQE9	B-cell CLL/lymphoma 7 protein family member B OS=Homo sapiens GN=BCL7B PE=1 SV=1 - [BCL7B_HUMAN]	17,33	1	[illegible]
O95183	Vesicle-associated membrane protein 5 OS=Homo sapiens GN=VAMP5 PE=1 SV=1 - [VAMP5_HUMAN]	17,24	1	[illegible]
F8W764	Myotubularin-related protein 1 (Fragment) OS=Homo sapiens GN=MTMR1 PE=4 SV=1 - [F8W764_HUMAN]	17,24	1	[illegible]
O00148	ATP-dependent RNA helicase DDX39A OS=Homo sapiens GN=DDX39A PE=1 SV=2 - [DX39A_HUMAN]	17,10	1	[illegible]
H0YJ16	Serine/threonine-protein phosphatase 2A regulatory subunit B'' subunit gamma (Fragment) OS=Homo sapiens GN=PPP2R3C PE=4 SV=1	17,07	1	[illegible]
Q9UFG5	UPF0449 protein C19orf25 OS=Homo sapiens GN=C19orf25 PE=1 SV=2 - [CS025_HUMAN]	16,95	1	[illegible]
Q9NPA8	Enhancer of yellow 2 transcription factor homolog OS=Homo sapiens GN=ENY2 PE=1 SV=1 - [ENY2_HUMAN]	16,83	1	[illegible]
Q562R1	Beta-actin-like protein 2 OS=Homo sapiens GN=ACTBL2 PE=1 SV=2 - [ACTBL_HUMAN]	16,76	1	[illegible]
Q9H6K4-2	Isoform 2 of Optic atrophy 3 protein OS=Homo sapiens GN=OPA3 - [OPA3_HUMAN]	16,67	1	[illegible]
J3KSA7	Methyl-CpG-binding domain protein 2 (Fragment) OS=Homo sapiens GN=MBD2 PE=4 SV=1 - [J3KSA7_HUMAN]	16,67	1	[illegible]
F5GWH5	UPF0197 transmembrane protein C11orf10 OS=Homo sapiens GN=C11orf10 PE=4 SV=1 - [F5GWH5_HUMAN]	16,33	1	[illegible]
H0YF33	Nuclear autoantigenic sperm protein (Fragment) OS=Homo sapiens GN=NASP PE=4 SV=1 - [H0YF33_HUMAN]	16,32	1	[illegible]
F8VTR6	Rab-3A-interacting protein OS=Homo sapiens GN=RAB3IP PE=4 SV=1 - [F8VTR6_HUMAN]	16,17	1	[illegible]
Q14657	L antigen family member 3 OS=Homo sapiens GN=LAGE3 PE=1 SV=2 - [LAGE3_HUMAN]	16,08	1	[illegible]
P19012	Keratin, type I cytoskeletal 15 OS=Homo sapiens GN=KRT15 PE=1 SV=3 - [K1C15_HUMAN]	16,01	1	[illegible]
Q8NFI4	Putative protein FAM10A5 OS=Homo sapiens GN=ST13P5 PE=5 SV=1 - [F10A5_HUMAN]	15,99	1	[illegible]
Q9UJC5	SH3 domain-binding glutamic acid-rich-like protein 2 OS=Homo sapiens GN=SH3BGRL2 PE=1 SV=2 - [SH3L2_HUMAN]	15,89	1	[illegible]
Q9NZT1	Calmodulin-like protein 5 OS=Homo sapiens GN=CALML5 PE=1 SV=2 - [CALL5_HUMAN]	15,75	1	[illegible]
Q9BTX3	Transmembrane protein 208 OS=Homo sapiens GN=TMEM208 PE=2 SV=1 - [TM208_HUMAN]	15,61	1	[illegible]
O60220	Mitochondrial import inner membrane translocase subunit Tim8 A OS=Homo sapiens GN=TIMM8A PE=1 SV=1 - [TIM8A_HUMAN]	15,46	1	[illegible]
Q9P086	Mediator of RNA polymerase II transcription subunit 11 OS=Homo sapiens GN=MED11 PE=1 SV=2 - [MED11_HUMAN]	15,38	1	[illegible]
Q7Z4G1	COMM domain-containing protein 6 OS=Homo sapiens GN=COMMD6 PE=1 SV=1 - [COMD6_HUMAN]	15,29	1	[illegible]
Q9NRR8	CDC42 small effector protein 1 OS=Homo sapiens GN=CDC42SE1 PE=1 SV=1 - [C42S1_HUMAN]	15,19	1	[illegible]
P62306	Small nuclear ribonucleoprotein F OS=Homo sapiens GN=SNRPF PE=1 SV=1 - [RUXF_HUMAN]	15,12	1	[illegible]
Q8TCF1-2	Isoform 2 of AN1-type zinc finger protein 1 OS=Homo sapiens GN=ZFAND1 - [ZFAN1_HUMAN]	14,91	1	[illegible]
Q9NZ42	Gamma-secretase subunit PEN-2 OS=Homo sapiens GN=PSENEN PE=1 SV=1 - [PEN2_HUMAN]	14,85	1	[illegible]
F8VVM9	Protein PRRC2A OS=Homo sapiens GN=PRRC2A PE=4 SV=1 - [F8VVM9_HUMAN]	14,83	1	[illegible]
P56181	NADH dehydrogenase [ubiquinone] flavoprotein 3, mitochondrial OS=Homo sapiens GN=NDUFV3 PE=1 SV=2 - [NDUV3_HUMAN]	14,81	1	[illegible]
Q15771	Ras-related protein Rab-30 OS=Homo sapiens GN=RAB30 PE=1 SV=2 - [RAB30_HUMAN]	14,78	1	[illegible]
H3BQ61	S phase cyclin A-associated protein in the endoplasmic reticulum OS=Homo sapiens GN=SCAPER PE=4 SV=1 - [H3BQ61_HUMAN]	14,67	1	[illegible]
Q9BQ49	UPF0608 protein C19orf42 OS=Homo sapiens GN=C19orf42 PE=1 SV=2 - [CS042_HUMAN]	14,67	1	[illegible]
P27469	G0/G1 switch regulatory protein 2 OS=Homo sapiens GN=G0S2 PE=1 SV=1 - [G0S2_HUMAN]	14,56	1	[illegible]
Q9P003	Protein cornichon homolog 4 OS=Homo sapiens GN=CNIH4 PE=2 SV=1 - [CNIH4_HUMAN]	14,39	1	[illegible]
Q7Z4R8	UPF0669 protein C6orf120 OS=Homo sapiens GN=C6orf120 PE=1 SV=1 - [CF120_HUMAN]	14,14	1	[illegible]
P46108	Adapter molecule crk OS=Homo sapiens GN=CRK PE=1 SV=2 - [CRK_HUMAN]	14,14	1	[illegible]
Q8NHZ8	Anaphase-promoting complex subunit CDC26 OS=Homo sapiens GN=CDC26 PE=1 SV=1 - [CDC26_HUMAN]	14,12	1	[illegible]
Q96I36	Cytochrome c oxidase assembly protein C12orf62 OS=Homo sapiens GN=C12orf62 PE=2 SV=1 - [CL062_HUMAN]	14,04	1	[illegible]
Q9H3Y8	Pancreatic progenitor cell differentiation and proliferation factor OS=Homo sapiens GN=PPDPF PE=2 SV=1 - [PPDPF_HUMAN]	14,04	1	[illegible]
E5RI71	Ribonuclease UK114 OS=Homo sapiens GN=HRSP12 PE=4 SV=1 - [E5RI71_HUMAN]	14,04	1	[illegible]
P41223	Protein BUD31 homolog OS=Homo sapiens GN=BUD31 PE=1 SV=2 - [BUD31_HUMAN]	13,89	1	[illegible]
Q9UMR2	ATP-dependent RNA helicase DDX19B OS=Homo sapiens GN=DDX19B PE=1 SV=1 - [DD19B_HUMAN]	13,78	1	[illegible]
Q9GZP1	Neurensin-2 OS=Homo sapiens GN=NRSN2 PE=2 SV=1 - [NRSN2_HUMAN]	13,73	1	[illegible]
P56381	ATP synthase subunit epsilon, mitochondrial OS=Homo sapiens GN=ATP5E PE=1 SV=2 - [ATP5E_HUMAN]	13,73	1	[illegible]
O14907	Tax1-binding protein 3 OS=Homo sapiens GN=TAX1BP3 PE=1 SV=2 - [TX1B3_HUMAN]	13,71	1	[illegible]
Q8NBT2	Kinetochore protein Spc24 OS=Homo sapiens GN=SPC24 PE=1 SV=2 - [SPC24_HUMAN]	13,71	1	[illegible]
Q9HD20-3	Isoform C of Probable cation-transporting ATPase 13A1 OS=Homo sapiens GN=ATP13A1 - [AT131_HUMAN]	13,66	1	[illegible]
Q9BYX7	Putative beta-actin-like protein 3 OS=Homo sapiens GN=POTEKP PE=5 SV=1 - [ACTBM_HUMAN]	13,60	1	[illegible]

Q96BP2	Coiled-coil-helix-coiled-coil-helix domain-containing protein 1 OS=Homo sapiens GN=CHCHD1 PE=1 SV=1 - [CHCH1_HUMAN]	13,56	1	1,118	
H0Y327	Monofunctional C1-tetrahydrofolate synthase, mitochondrial (Fragment) OS=Homo sapiens GN=MTHFD1L PE=4 SV=1 - [H0Y327_HUMAN	13,55	1		1,052
J3KN91	Uncharacterized protein C12orf45 OS=Homo sapiens GN=C12orf45 PE=4 SV=1 - [J3KN91_HUMAN]	13,43	1		
D6RA89	Peroxisome biogenesis factor 10 OS=Homo sapiens GN=PEX10 PE=4 SV=1 - [D6RA89_HUMAN]	13,40	1		0,956
H0YCF4	Gem-associated protein 2 (Fragment) OS=Homo sapiens GN=GEMIN2 PE=4 SV=1 - [H0YCF4_HUMAN]	13,33	1	0,838	
Q9BZD6	Transmembrane gamma-carboxyglutamic acid protein 4 OS=Homo sapiens GN=PRRG4 PE=2 SV=1 - [TMG4_HUMAN]	13,27	1	1,043	
P63218	Guanine nucleotide-binding protein G(I)/G(S)/G(O) subunit gamma-5 OS=Homo sapiens GN=GNG5 PE=1 SV=3 - [GBG5_HUMAN]	13,24	1	0,503	0,467
Q9HAV0	Guanine nucleotide-binding protein subunit beta-4 OS=Homo sapiens GN=GNB4 PE=1 SV=3 - [GBB4_HUMAN]	13,24	1	1,547	
P03928	ATP synthase protein 8 OS=Homo sapiens GN=MT-ATP8 PE=1 SV=1 - [ATP8_HUMAN]	13,24	1	1,311	1,312
P0DJ93	UPF0766 protein C6orf228 OS=Homo sapiens GN=C6orf228 PE=3 SV=1 - [CF228_HUMAN]	13,19	1	1,331	1,074
P07311	Acylphosphatase-1 OS=Homo sapiens GN=ACYP1 PE=1 SV=2 - [ACYP1_HUMAN]	13,13	1		
H0Y2Q1	tRNA (adenine(58)-N(1))-methyltransferase catalytic subunit TRMT61A (Fragment) OS=Homo sapiens GN=TRMT61A PE=4 SV=1 - [H0Y	13,09	1		
P10176	Cytochrome c oxidase subunit 8A, mitochondrial OS=Homo sapiens GN=COX8A PE=1 SV=2 - [COX8A_HUMAN]	13,04	1	1,324	1,254
P03897	NADH-ubiquinone oxidoreductase chain 3 OS=Homo sapiens GN=MT-ND3 PE=1 SV=1 - [NU3M_HUMAN]	13,04	1	1,077	1,147
H3BUF6	Ataxin-2-like protein OS=Homo sapiens GN=ATXN2L PE=4 SV=1 - [H3BUF6_HUMAN]	13,01	1		
H3BRR0	Maspardin OS=Homo sapiens GN=SPG21 PE=4 SV=1 - [H3BRR0_HUMAN]	12,99	1		
O14493	Claudin-4 OS=Homo sapiens GN=CLDN4 PE=1 SV=1 - [CLD4_HUMAN]	12,92	1	0,844	0,666
Q53RY4	Keratinocyte-associated protein 3 OS=Homo sapiens GN=KRTCAP3 PE=2 SV=1 - [KCP3_HUMAN]	12,92	1	0,309	
F8W0B3	Voltage-gated hydrogen channel 1 (Fragment) OS=Homo sapiens GN=HVCN1 PE=4 SV=1 - [F8W0B3_HUMAN]	12,82	1	**2,333**	
Q9BTL3	RNMT-activating mini protein OS=Homo sapiens GN=FAM103A1 PE=1 SV=1 - [RAM_HUMAN]	12,71	1	0,871	
P24666-2	Isoform 2 of Low molecular weight phosphotyrosine protein phosphatase OS=Homo sapiens GN=ACP1 - [PPAC_HUMAN]	12,66	1		
P33552	Cyclin-dependent kinases regulatory subunit 2 OS=Homo sapiens GN=CKS2 PE=1 SV=1 - [CKS2_HUMAN]	12,66	1		
D6REC9	THO complex subunit 3 OS=Homo sapiens GN=THOC3 PE=4 SV=1 - [D6REC9_HUMAN]	12,65	1		
O43345	Zinc finger protein 208 OS=Homo sapiens GN=ZNF208 PE=2 SV=1 - [ZN208_HUMAN]	12,60	1		1,032
Q8TBQ9	Protein kish-A OS=Homo sapiens GN=TMEM167A PE=2 SV=1 - [KISHA_HUMAN]	12,50	1	0,494	
H3BMF1	Nuclear envelope phosphatase-regulatory subunit 1 (Fragment) OS=Homo sapiens GN=CNEP1R1 PE=4 SV=1 - [H3BMF1_HUMAN]	12,50	1	0,992	
O96033	Molybdopterin synthase sulfur carrier subunit OS=Homo sapiens GN=MOCS2 PE=1 SV=1 - [MOC2A_HUMAN]	12,50	1		
Q9NP90	Ras-related protein Rab-9B OS=Homo sapiens GN=RAB9B PE=1 SV=1 - [RAB9B_HUMAN]	12,44	1		1,012
B1ARP7	Chromosome 1 open reading frame 123 OS=Homo sapiens GN=C1orf123 PE=4 SV=1 - [B1ARP7_HUMAN]	12,41	1		
Q8WWM7	Ataxin-2-like protein OS=Homo sapiens GN=ATXN2L PE=1 SV=2 - [ATX2L_HUMAN]	12,37	1		
Q9NRF9	DNA polymerase epsilon subunit 3 OS=Homo sapiens GN=POLE3 PE=1 SV=1 - [DPOE3_HUMAN]	12,24	1	0,741	
Q96A73-3	Isoform 3 of Putative monooxygenase p33MONOX OS=Homo sapiens GN=KIAA1191 - [P33MX_HUMAN]	12,24	1		
I3L2B0	Clustered mitochondria protein homolog OS=Homo sapiens GN=KIAA0664 PE=3 SV=1 - [I3L2B0_HUMAN]	12,22	1		
G5E9N8	HCG37164, isoform CRA_c OS=Homo sapiens GN=LYSMD3 PE=4 SV=1 - [G5E9N8_HUMAN]	12,22	1		0,477
G3V599	Cutaneous T-cell lymphoma-associated antigen 5 OS=Homo sapiens GN=CTAGE5 PE=4 SV=1 - [G3V599_HUMAN]	12,10	1		1,054
E7EV59	V-type proton ATPase subunit C 1 OS=Homo sapiens GN=ATP6V1C1 PE=4 SV=1 - [E7EV59_HUMAN]	12,05	1	1,037	
P62942	Peptidyl-prolyl cis-trans isomerase FKBP1A OS=Homo sapiens GN=FKBP1A PE=1 SV=2 - [FKB1A_HUMAN]	12,04	1		
O15116	U6 snRNA-associated Sm-like protein LSm1 OS=Homo sapiens GN=LSM1 PE=1 SV=1 - [LSM1_HUMAN]	12,03	1		
I3L3T4	TOM1-like protein 1 OS=Homo sapiens GN=TOM1L1 PE=4 SV=1 - [I3L3T4_HUMAN]	12,00	1		
Q3ZAQ7	Vacuolar ATPase assembly integral membrane protein VMA21 OS=Homo sapiens GN=VMA21 PE=1 SV=1 - [VMA21_HUMAN]	11,88	1	0,680	0,657
Q8TDH9-2	Isoform 2 of Protein Muted homolog OS=Homo sapiens GN=MUTED - [MUTED_HUMAN]	11,82	1		
Q9BY43	Charged multivesicular body protein 4a OS=Homo sapiens GN=CHMP4A PE=1 SV=3 - [CHM4A_HUMAN]	11,71	1		
H0YEY4	ADP-sugar pyrophosphatase (Fragment) OS=Homo sapiens GN=NUDT5 PE=3 SV=1 - [H0YEY4_HUMAN]	11,68	1		
H7C5M1	RNA-binding protein 34 (Fragment) OS=Homo sapiens GN=RBM34 PE=4 SV=1 - [H7C5M1_HUMAN]	11,63	1		
Q15012	Lysosomal-associated transmembrane protein 4A OS=Homo sapiens GN=LAPTM4A PE=1 SV=1 - [LAP4A_HUMAN]	11,59	1	1,284	1,364
O75525	KH domain-containing, RNA-binding, signal transduction-associated protein 3 OS=Homo sapiens GN=KHDRBS3 PE=1 SV=1 - [KHDR3_HU	11,56	1		
Q08170	Serine/arginine-rich splicing factor 4 OS=Homo sapiens GN=SRSF4 PE=1 SV=2 - [SRSF4_HUMAN]	11,54	1		
Q9UMX0	Ubiquilin-1 OS=Homo sapiens GN=UBQLN1 PE=1 SV=2 - [UBQL1_HUMAN]	11,54	1		0,973
P07108	Acyl-CoA-binding protein OS=Homo sapiens GN=DBI PE=1 SV=2 - [ACBP_HUMAN]	11,49	1		
Q12962	Transcription initiation factor TFIID subunit 10 OS=Homo sapiens GN=TAF10 PE=1 SV=1 - [TAF10_HUMAN]	11,47	1		
F5H2Z7	Coiled-coil domain-containing protein 92 (Fragment) OS=Homo sapiens GN=CCDC92 PE=4 SV=1 - [F5H2Z7_HUMAN]	11,46	1	1,152	
O75935-3	Isoform 3 of Dynactin subunit 3 OS=Homo sapiens GN=DCTN3 - [DCTN3_HUMAN]	11,39	1		0,729
Q9Y3E1	Hepatoma-derived growth factor-related protein 3 OS=Homo sapiens GN=HDGFRP3 PE=1 SV=1 - [HDGR3_HUMAN]	11,33	1		
Q96DE0	U8 snoRNA-decapping enzyme OS=Homo sapiens GN=NUDT16 PE=1 SV=2 - [NUD16_HUMAN]	11,28	1		
C9JQD0	Quinone oxidoreductase-like protein 1 (Fragment) OS=Homo sapiens GN=CRYZL1 PE=4 SV=1 - [C9JQD0_HUMAN]	11,21	1		
A2ABB6	WD repeat domain 46 (Fragment) OS=Homo sapiens GN=WDR46 PE=4 SV=1 - [A2ABB6_HUMAN]	11,18	1		1,065
Q8N4T8-2	Isoform 2 of Carbonyl reductase family member 4 OS=Homo sapiens GN=CBR4 - [CBR4_HUMAN]	11,17	1	0,799	0,952
Q8WUN7	Ubiquitin domain-containing protein 2 OS=Homo sapiens GN=UBTD2 PE=1 SV=2 - [UBTD2_HUMAN]	11,11	1	1,123	
P80075	C-C motif chemokine 8 OS=Homo sapiens GN=CCL8 PE=1 SV=2 - [CCL8_HUMAN]	11,11	1	1,055	
Q86VU5	Catechol O-methyltransferase domain-containing protein 1 OS=Homo sapiens GN=COMTD1 PE=1 SV=1 - [CMTD1_HUMAN]	11,07	1	0,623	
P31321	cAMP-dependent protein kinase type I-beta regulatory subunit OS=Homo sapiens GN=PRKAR1B PE=1 SV=4 - [KAP1_HUMAN]	11,02	1		0,840
Q8IVP5	FUN14 domain-containing protein 1 OS=Homo sapiens GN=FUNDC1 PE=1 SV=1 - [FUND1_HUMAN]	10,97	1	1,196	1,210
P0DJ07	Uncharacterized protein C19orf79 OS=Homo sapiens GN=C19orf79 PE=4 SV=1 - [CS079_HUMAN]	10,96	1	1,219	
Q9NPB0	SAYSvFN domain-containing protein 1 OS=Homo sapiens GN=SAYSD1 PE=2 SV=1 - [SMDC1_HUMAN]	10,93	1	**2,300**	1,711
Q8N0X2-4	Isoform 4 of Sperm-associated antigen 16 protein OS=Homo sapiens GN=SPAG16 - [SPG16_HUMAN]	10,93	1		
P42771	Cyclin-dependent kinase inhibitor 2A, isoforms 1/2/3 OS=Homo sapiens GN=CDKN2A PE=1 SV=2 - [CD2A1_HUMAN]	10,90	1		0,760
Q9Y2V2	Calcium-regulated heat stable protein 1 OS=Homo sapiens GN=CARHSP1 PE=1 SV=2 - [CHSP1_HUMAN]	10,88	1	0,914	0,755
Q04760	Lactoylglutathione lyase OS=Homo sapiens GN=GLO1 PE=1 SV=4 - [LGUL_HUMAN]	10,87	1		
O14966	Ras-related protein Rab-7L1 OS=Homo sapiens GN=RAB7L1 PE=1 SV=1 - [RAB7L_HUMAN]	10,84	1	1,294	1,408
A8MTL9	Serpin-like protein HMSD OS=Homo sapiens GN=HMSD PE=2 SV=1 - [HMSD_HUMAN]	10,79	1		0,258
P20962	Parathymosin OS=Homo sapiens GN=PTMS PE=1 SV=2 - [PTMS_HUMAN]	10,78	1		
Q96D05	Uncharacterized protein C10orf35 OS=Homo sapiens GN=C10orf35 PE=1 SV=1 - [CJ035_HUMAN]	10,74	1		1,010
Q9H008	Phospholysine phosphohistidine inorganic pyrophosphate phosphatase OS=Homo sapiens GN=LHPP PE=1 SV=2 - [LHPP_HUMAN]	10,74	1		
Q969Q4	ADP-ribosylation factor-like protein 11 OS=Homo sapiens GN=ARL11 PE=1 SV=1 - [ARL11_HUMAN]	10,71	1		
Q6RW13	Type-1 angiotensin II receptor-associated protein OS=Homo sapiens GN=AGTRAP PE=1 SV=1 - [ATRAP_HUMAN]	10,69	1		1,017
E9PKP4	Processed macrophage colony-stimulating factor 1 (Fragment) OS=Homo sapiens GN=CSF1 PE=4 SV=1 - [E9PKP4_HUMAN]	10,62	1	1,592	
Q96HJ9	UPF0562 protein C7orf55 OS=Homo sapiens GN=C7orf55 PE=1 SV=2 - [CG055_HUMAN]	10,62	1		
A6ZKI3	Protein FAM127A OS=Homo sapiens GN=FAM127A PE=1 SV=1 - [F127A_HUMAN]	10,62	1		
Q9Y6G3	39S ribosomal protein L42, mitochondrial OS=Homo sapiens GN=MRPL42 PE=1 SV=1 - [RM42_HUMAN]	10,56	1	0,852	
H3BUS9	Myotubularin-related protein 10 OS=Homo sapiens GN=MTMR10 PE=4 SV=1 - [H3BUS9_HUMAN]	10,53	1		1,463
Q5SZ64	Splicing factor 3B subunit 4 (Fragment) OS=Homo sapiens GN=SF3B4 PE=2 SV=1 - [Q5SZ64_HUMAN]	10,53	1		
Q96AW1	Vesicular, overexpressed in cancer, prosurvival protein 1 OS=Homo sapiens GN=VOPP1 PE=2 SV=1 - [VOPP1_HUMAN]	10,47	1	0,334	
D6R9Y1	Polyadenylate-binding protein-interacting protein 2 OS=Homo sapiens GN=PAIP2 PE=4 SV=1 - [D6R9Y1_HUMAN]	10,45	1		
Q9UI30	tRNA methyltransferase 112 homolog OS=Homo sapiens GN=TRMT112 PE=1 SV=1 - [TR112_HUMAN]	10,40	1	0,901	
P35754	Glutaredoxin-1 OS=Homo sapiens GN=GLRX PE=1 SV=2 - [GLRX1_HUMAN]	10,38	1		
Q5VW38-2	Isoform 2 of Protein GPR107 OS=Homo sapiens GN=GPR107 - [GP107_HUMAN]	10,33	1	1,304	1,458
D6RAE7	Coiled-coil alpha-helical rod protein 1 (Fragment) OS=Homo sapiens GN=CCHCR1 PE=4 SV=1 - [D6RAE7_HUMAN]	10,32	1		
Q13243	Serine/arginine-rich splicing factor 5 OS=Homo sapiens GN=SRSF5 PE=1 SV=1 - [SRSF5_HUMAN]	10,29	1		
O95295	SNARE-associated protein Snapin OS=Homo sapiens GN=SNAPIN PE=1 SV=1 - [SNAPN_HUMAN]	10,29	1		
Q9Y3C4	TP53RK-binding protein OS=Homo sapiens GN=TPRKB PE=1 SV=1 - [TPRKB_HUMAN]	10,29	1		
Q5TGZ0	Mitochondrial inner membrane organizing system protein 1 OS=Homo sapiens GN=MINOS1 PE=1 SV=1 - [MOS1_HUMAN]	10,26	1	1,366	1,272
Q8IW50-3	Isoform 3 of Protein FAM219A OS=Homo sapiens GN=FAM219A - [F219A_HUMAN]	10,26	1	0,797	
Q5BKU9	Uncharacterized protein C17orf90 OS=Homo sapiens GN=C17orf90 PE=2 SV=1 - [CQ090_HUMAN]	10,20	1		
G5E994	G protein-coupled receptor 107, isoform CRA_c OS=Homo sapiens GN=GPR107 PE=4 SV=1 - [G5E994_HUMAN]	10,16	1	1,050	
O60783	28S ribosomal protein S14, mitochondrial OS=Homo sapiens GN=MRPS14 PE=1 SV=1 - [RT14_HUMAN]	10,16	1		
Q04900	Sialomucin core protein 24 OS=Homo sapiens GN=CD164 PE=1 SV=2 - [MUC24_HUMAN]	10,15	1		1,240
H7C1H6	Exostosin-1 (Fragment) OS=Homo sapiens GN=EXT1 PE=4 SV=1 - [H7C1H6_HUMAN]	10,14	1	1,140	
C9JCE0	AF4/FMR2 family member 4 (Fragment) OS=Homo sapiens GN=AFF4 PE=4 SV=1 - [C9JCE0_HUMAN]	10,14	1		
O95379	Tumor necrosis factor alpha-induced protein 8 OS=Homo sapiens GN=TNFAIP8 PE=2 SV=1 - [TFIP8_HUMAN]	10,10	1	0,719	1,000
Q58FF6	Putative heat shock protein HSP 90-beta 4 OS=Homo sapiens GN=HSP90AB4P PE=5 SV=1 - [H90B4_HUMAN]	10,10	1		
Q99627	COP9 signalosome complex subunit 8 OS=Homo sapiens GN=COPS8 PE=1 SV=1 - [CSN8_HUMAN]	10,05	1		
H7C1U1	HMG box transcription factor BBX (Fragment) OS=Homo sapiens GN=BBX PE=4 SV=1 - [H7C1U1_HUMAN]	10,00	1		
P04216	Thy-1 membrane glycoprotein OS=Homo sapiens GN=THY1 PE=1 SV=2 - [THY1_HUMAN]	9,94	1	0,749	
O15243	Leptin receptor gene-related protein OS=Homo sapiens GN=LEPROT PE=1 SV=1 - [OBRG_HUMAN]	9,92	1	1,834	1,337
Q9Y237	Peptidyl-prolyl cis-trans isomerase NIMA-interacting 4 OS=Homo sapiens GN=PIN4 PE=1 SV=1 - [PIN4_HUMAN]	9,92	1		
H0Y6C1	5'-nucleotidase domain-containing protein 1 (Fragment) OS=Homo sapiens GN=NT5DC1 PE=4 SV=1 - [H0Y6C1_HUMAN]	9,91	1		
E9PN08	E3 ubiquitin-protein ligase RNF5 OS=Homo sapiens GN=RNF5 PE=4 SV=1 - [E9PN08_HUMAN]	9,89	1		
H0YDZ1	Myotubularin-related protein 13 (Fragment) OS=Homo sapiens GN=SBF2 PE=4 SV=1 - [H0YDZ1_HUMAN]	9,85	1		1,151
Q9NX08	COMM domain-containing protein 8 OS=Homo sapiens GN=COMMD8 PE=1 SV=1 - [COMD8_HUMAN]	9,84	1		
P54852	Epithelial membrane protein 3 OS=Homo sapiens GN=EMP3 PE=2 SV=1 - [EMP3_HUMAN]	9,82	1	**7,208**	
E5RJL3	Aspartyl/asparaginyl beta-hydroxylase OS=Homo sapiens GN=ASPH PE=4 SV=1 - [E5RJL3_HUMAN]	9,82	1	1,334	1,208
Q9ULX9	Transcription factor MafF OS=Homo sapiens GN=MAFF PE=1 SV=2 - [MAFF_HUMAN]	9,76	1		
Q9NS18	Glutaredoxin-2, mitochondrial OS=Homo sapiens GN=GLRX2 PE=1 SV=1 - [GLRX2_HUMAN]	9,76	1		
Q8TDD1-2	Isoform 2 of ATP-dependent RNA helicase DDX54 OS=Homo sapiens GN=DDX54 - [DDX54_HUMAN]	9,75	1		
Q9UK76	Hematological and neurological expressed 1 protein OS=Homo sapiens GN=HN1 PE=1 SV=3 - [HN1_HUMAN]	9,74	1		
E9PJF6	AMP deaminase 2 (Fragment) OS=Homo sapiens GN=AMPD2 PE=4 SV=1 - [E9PJF6_HUMAN]	9,71	1		
Q8WZ82	Ovarian cancer-associated gene 2 protein OS=Homo sapiens GN=OVCA2 PE=1 SV=1 - [OVCA2_HUMAN]	9,69	1		0,731
Q9Y241	HIG1 domain family member 1A OS=Homo sapiens GN=HIGD1A PE=1 SV=1 - [HIG1A_HUMAN]	9,68	1	0,741	0,769
Q8TDD1	ATP-dependent RNA helicase DDX54 OS=Homo sapiens GN=DDX54 PE=1 SV=2 - [DDX54_HUMAN]	9,65	1		
Q15262-2	Isoform 2 of Receptor-type tyrosine-protein phosphatase kappa OS=Homo sapiens GN=PTPRK - [PTPRK_HUMAN]	9,58	1	1,289	1,318
Q9BZX2	Uridine-cytidine kinase 2 OS=Homo sapiens GN=UCK2 PE=1 SV=1 - [UCK2_HUMAN]	9,58	1		
P58557	rRNA maturation factor homolog OS=Homo sapiens GN=YBEY PE=2 SV=2 - [YBEY_HUMAN]	9,58	1		
Q9BRT3	Migration and invasion enhancer 1 OS=Homo sapiens GN=MIEN1 PE=1 SV=1 - [MIEN1_HUMAN]	9,57	1		
Q8N6M0	OTU domain-containing protein 6B OS=Homo sapiens GN=OTUD6B PE=1 SV=1 - [OTU6B_HUMAN]	9,56	1	0,962	
Q99735	Microsomal glutathione S-transferase 2 OS=Homo sapiens GN=MGST2 PE=1 SV=1 - [MGST2_HUMAN]	9,52	1	0,478	0,595
Q15262	Receptor-type tyrosine-protein phosphatase kappa OS=Homo sapiens GN=PTPRK PE=1 SV=2 - [PTPRK_HUMAN]	9,52	1	1,024	
Q96HA1	Nuclear envelope pore membrane protein POM 121 OS=Homo sapiens GN=POM121 PE=1 SV=2 - [P121A_HUMAN]	9,45	1	1,689	
H3BUB6	Secretory carrier-associated membrane protein 2 (Fragment) OS=Homo sapiens GN=SCAMP2 PE=4 SV=1 - [H3BUB6_HUMAN]	9,45	1	0,527	0,913
Q9GZP8	Immortalization up-regulated protein OS=Homo sapiens GN=IMUP PE=1 SV=1 - [IMUP_HUMAN]	9,43	1		1,211
Q9NQG1	Protein MANBAL OS=Homo sapiens GN=MANBAL PE=1 SV=1 - [MANBL_HUMAN]	9,41	1	1,462	1,552
H0YB07	Lymphocyte antigen 6K OS=Homo sapiens GN=LY6K PE=4 SV=1 - [H0YB07_HUMAN]	9,41	1		0,734
E5RGS4	Prefoldin subunit 1 OS=Homo sapiens GN=PFDN1 PE=4 SV=1 - [E5RGS4_HUMAN]	9,40	1		
Q5VZF2	Muscleblind-like protein 2 OS=Homo sapiens GN=MBNL2 PE=1 SV=2 - [MBNL2_HUMAN]	9,38	1	1,009	
Q9H4I9	UPF0466 protein C22orf32, mitochondrial OS=Homo sapiens GN=C22orf32 PE=2 SV=1 - [CV032_HUMAN]	9,35	1	1,178	1,101
C9JF62	NEDD8-conjugating enzyme UBE2F (Fragment) OS=Homo sapiens GN=UBE2F PE=3 SV=1 - [C9JF62_HUMAN]	9,35	1		
P49458	Signal recognition particle 9 kDa protein OS=Homo sapiens GN=SRP9 PE=1 SV=2 - [SRP09_HUMAN]	9,30	1		
P43308	Translocon-associated protein subunit beta OS=Homo sapiens GN=SSR2 PE=1 SV=1 - [SSRB_HUMAN]	9,29	1	0,899	0,889
Q5SVS4	Kidney mitochondrial carrier protein 1 OS=Homo sapiens GN=SLC25A30 PE=2 SV=1 - [KMCP1_HUMAN]	9,28	1	**3,398**	
Q0VAQ4	Small cell adhesion glycoprotein OS=Homo sapiens GN=SMAGP PE=1 SV=1 - [SMAGP_HUMAN]	9,28	1	1,157	
Q5VUM1	UPF0369 protein C6orf57 OS=Homo sapiens GN=C6orf57 PE=2 SV=1 - [CF057_HUMAN]	9,26	1		
H0Y9L8	MYC-induced nuclear antigen (Fragment) OS=Homo sapiens GN=MINA PE=4 SV=1 - [H0Y9L8_HUMAN]	9,26	1		
Q969T7	Cytosolic 5'-nucleotidase III-like protein OS=Homo sapiens GN=NT5C3L PE=1 SV=3 - [5NT3L_HUMAN]	9,25	1		
B4DQE6	Transmembrane protein 39B OS=Homo sapiens GN=TMEM39B PE=2 SV=1 - [B4DQE6_HUMAN]	9,22	1	0,980	
Q9NWU2	Glucose-induced degradation protein 8 homolog OS=Homo sapiens GN=GID8 PE=1 SV=1 - [GID8_HUMAN]	9,21	1		
Q9GZU7	Carboxy-terminal domain RNA polymerase II polypeptide A small phosphatase 1 OS=Homo sapiens GN=CTDSP1 PE=1 SV=1 - [CTDS1_I	9,20	1		0,839
E9PAX8	Periphilin-1 OS=Homo sapiens GN=PPHLN1 PE=4 SV=1 - [E9PAX8_HUMAN]	9,20	1		
Q9Y2Y0	ADP-ribosylation factor-like protein 2-binding protein OS=Homo sapiens GN=ARL2BP PE=1 SV=1 - [AR2BP_HUMAN]	9,20	1		
Q16626	Male-enhanced antigen 1 OS=Homo sapiens GN=MEA1 PE=1 SV=2 - [MEA1_HUMAN]	9,19	1		
Q7L2R6	Zinc finger protein 765 OS=Homo sapiens GN=ZNF765 PE=2 SV=2 - [ZN765_HUMAN]	9,18	1		
C9JLY3	Zinc finger protein 800 (Fragment) OS=Homo sapiens GN=ZNF800 PE=4 SV=1 - [C9JLY3_HUMAN]	9,18	1		
Q9H098	Protein FAM107B OS=Homo sapiens GN=FAM107B PE=1 SV=1 - [F107B_HUMAN]	9,16	1		
E9PBV3	Suprabasin OS=Homo sapiens GN=SBSN PE=4 SV=1 - [E9PBV3_HUMAN]	9,15	1	1,600	1,339
Q9C005	Protein dpy-30 homolog OS=Homo sapiens GN=DPY30 PE=1 SV=1 - [DPY30_HUMAN]	9,09	1		
E9PGZ2	Leucine-rich repeat flightless-interacting protein 1 OS=Homo sapiens GN=LRRFIP1 PE=4 SV=1 - [E9PGZ2_HUMAN]	9,06	1		
Q4VC05	B-cell CLL/lymphoma 7 protein family member A OS=Homo sapiens GN=BCL7A PE=1 SV=1 - [BCL7A_HUMAN]	9,05	1		
Q99757	Thioredoxin, mitochondrial OS=Homo sapiens GN=TXN2 PE=1 SV=2 - [THIOM_HUMAN]	9,04	1		1,019
D6RB58	Phosphoethanolamine/phosphocholine phosphatase (Fragment) OS=Homo sapiens GN=PHOSPHO1 PE=4 SV=1 - [D6RB58_HUMAN]	9,04	1		0,595
Q9NYP7	Elongation of very long chain fatty acids protein 5 OS=Homo sapiens GN=ELOVL5 PE=1 SV=1 - [ELOV5_HUMAN]	9,03	1	1,949	
Q9NR56	Muscleblind-like protein 1 OS=Homo sapiens GN=MBNL1 PE=1 SV=2 - [MBNL1_HUMAN]	9,02	1	0,952	
Q8N0T1	Uncharacterized protein C8orf59 OS=Homo sapiens GN=C8orf59 PE=1 SV=2 - [CH059_HUMAN]	9,00	1		
P30154	Serine/threonine-protein phosphatase 2A 65 kDa regulatory subunit A beta isoform OS=Homo sapiens GN=PPP2R1B PE=1 SV=3 - [2AAB	8,99	1		0,755
C9IZW8	NADH dehydrogenase [ubiquinone] 1 beta subcomplex subunit 2, mitochondrial OS=Homo sapiens GN=NDUFB2 PE=4 SV=1 - [C9IZW8_H	8,97	1	1,502	1,756
Q9NQP4	Prefoldin subunit 4 OS=Homo sapiens GN=PFDN4 PE=1 SV=1 - [PFD4_HUMAN]	8,96	1		
P22466	Galanin peptides OS=Homo sapiens GN=GAL PE=1 SV=3 - [GALA_HUMAN]	8,94	1		
O76095	Protein JTB OS=Homo sapiens GN=JTB PE=1 SV=1 - [JTB_HUMAN]	8,90	1	1,057	1,099
H7C561	Splicing factor 1 (Fragment) OS=Homo sapiens GN=SF1 PE=4 SV=1 - [H7C561_HUMAN]	8,90	1	1,776	1,575
Q9HD15	Steroid receptor RNA activator 1 OS=Homo sapiens GN=SRA1 PE=1 SV=1 - [SRA1_HUMAN]	8,90	1		
A5PLL7	Transmembrane protein 189 OS=Homo sapiens GN=TMEM189 PE=2 SV=3 - [TM189_HUMAN]	8,89	1	0,734	
Q9Y342	Plasmolipin OS=Homo sapiens GN=PLLP PE=1 SV=1 - [PLLP_HUMAN]	8,79	1	0,935	
Q6UW01	Cerebellin-3 OS=Homo sapiens GN=CBLN3 PE=1 SV=1 - [CBLN3_HUMAN]	8,78	1		

E5RGZ5	Fatty acid desaturase 2 (Fragment) OS=Homo sapiens GN=FADS2 PE=4 SV=1 - [E5RGZ5_HUMAN]	8,74	1
P15374	Ubiquitin carboxyl-terminal hydrolase isozyme L3 OS=Homo sapiens GN=UCHL3 PE=1 SV=1 - [UCHL3_HUMAN]	8,70	1
Q13158	Protein FADD OS=Homo sapiens GN=FADD PE=1 SV=1 - [FADD_HUMAN]	8,65	1
Q9Y4Z0	U6 snRNA-associated Sm-like protein LSm4 OS=Homo sapiens GN=LSM4 PE=1 SV=1 - [LSM4_HUMAN]	8,63	1
Q9BW83	Intraflagellar transport protein 27 homolog OS=Homo sapiens GN=IFT27 PE=1 SV=1 - [IFT27_HUMAN]	8,60	1
Q4U2R6	39S ribosomal protein L51, mitochondrial OS=Homo sapiens GN=MRPL51 PE=1 SV=1 - [RM51_HUMAN]	8,59	1
Q5EBL8	PDZ domain-containing protein 11 OS=Homo sapiens GN=PDZD11 PE=1 SV=2 - [PDZ11_HUMAN]	8,57	1
Q9NPJ3	Acyl-coenzyme A thioesterase 13 OS=Homo sapiens GN=ACOT13 PE=1 SV=1 - [ACO13_HUMAN]	8,57	1
Q04941	Proteolipid protein 2 OS=Homo sapiens GN=PLP2 PE=1 SV=1 - [PLP2_HUMAN]	8,55	1
Q9BRG1	Vacuolar protein-sorting-associated protein 25 OS=Homo sapiens GN=VPS25 PE=1 SV=1 - [VPS25_HUMAN]	8,52	1
F8VVA0	Protein C12orf44 (Fragment) OS=Homo sapiens GN=C12orf44 PE=4 SV=1 - [F8VVA0_HUMAN]	8,51	1
P16220	Cyclic AMP-responsive element-binding protein 1 OS=Homo sapiens GN=CREB1 PE=1 SV=2 - [CREB1_HUMAN]	8,50	1
Q9UHD9	Ubiquilin-2 OS=Homo sapiens GN=UBQLN2 PE=1 SV=2 - [UBQL2_HUMAN]	8,49	1
A5A3E0	POTE ankyrin domain family member F OS=Homo sapiens GN=POTEF PE=1 SV=2 - [POTEF_HUMAN]	8,47	1
Q53GG5-3	Isoform 3 of PDZ and LIM domain protein 3 OS=Homo sapiens GN=PDLIM3 - [PDLI3_HUMAN]	8,47	1
Q9Y5J9	Mitochondrial import inner membrane translocase subunit Tim8 B OS=Homo sapiens GN=TIMM8B PE=1 SV=1 - [TIM8B_HUMAN]	8,43	1
O75607	Nucleoplasmin-3 OS=Homo sapiens GN=NPM3 PE=1 SV=3 - [NPM3_HUMAN]	8,43	1
Q5SXM8	DNL-type zinc finger protein OS=Homo sapiens GN=DNLZ PE=2 SV=1 - [DNLZ_HUMAN]	8,43	1
P61599	N-alpha-acetyltransferase 20 OS=Homo sapiens GN=NAA20 PE=1 SV=1 - [NAA20_HUMAN]	8,43	1
Q12974	Protein tyrosine phosphatase type IVA 2 OS=Homo sapiens GN=PTP4A2 PE=1 SV=1 - [TP4A2_HUMAN]	8,38	1
Q8N5G0	Uncharacterized protein C4orf52 OS=Homo sapiens GN=C4orf52 PE=1 SV=2 - [CD052_HUMAN]	8,33	1
E9PKR8	Sulfotransferase 1A2 (Fragment) OS=Homo sapiens GN=SULT1A2 PE=4 SV=1 - [E9PKR8_HUMAN]	8,33	1
Q7Z794	Keratin, type II cytoskeletal 1b OS=Homo sapiens GN=KRT77 PE=1 SV=3 - [K2C1B_HUMAN]	8,30	1
Q9H6E4	Coiled-coil domain-containing protein 134 OS=Homo sapiens GN=CCDC134 PE=1 SV=1 - [CC134_HUMAN]	8,30	1
Q8WUZ0	B-cell CLL/lymphoma 7 protein family member C OS=Homo sapiens GN=BCL7C PE=1 SV=3 - [BCL7C_HUMAN]	8,29	1
H7BXW7	Mitochondrial pyruvate carrier 1 OS=Homo sapiens GN=MPC1 PE=4 SV=1 - [H7BXW7_HUMAN]	8,26	1
P35244	Replication protein A 14 kDa subunit OS=Homo sapiens GN=RPA3 PE=1 SV=1 - [RFA3_HUMAN]	8,26	1
C9JQS8	WW domain-containing transcription regulator protein 1 (Fragment) OS=Homo sapiens GN=WWTR1 PE=4 SV=1 - [C9JQS8_HUMAN]	8,26	1
E9PPG8	Protein Hikeshi OS=Homo sapiens GN=C11orf73 PE=4 SV=1 - [E9PPG8_HUMAN]	8,25	1
Q9H2W2	Homeobox protein MIXL1 OS=Homo sapiens GN=MIXL1 PE=1 SV=1 - [MIXL1_HUMAN]	8,19	1
Q9NWV8-3	Isoform 3 of BRISC and BRCA1-A complex member 1 OS=Homo sapiens GN=BABAM1 - [BABA1_HUMAN]	8,19	1
H3BPE1	Microtubule-actin cross-linking factor 1, isoforms 1/2/3/5 OS=Homo sapiens GN=MACF1 PE=4 SV=1 - [H3BPE1_HUMAN]	8,14	1
Q5T2E7	Novel protein OS=Homo sapiens GN=RP11-190J1.9 PE=2 SV=1 - [Q5T2E7_HUMAN]	8,08	1
Q69YL0	Uncharacterized protein DKFZp762I1415 OS=Homo sapiens PE=4 SV=1 - [YC029_HUMAN]	8,08	1
Q9NRP0	Oligosaccharyltransferase complex subunit OSTC OS=Homo sapiens GN=OSTC PE=1 SV=1 - [OSTC_HUMAN]	8,05	1
H7C1U7	Probable cation-transporting ATPase 13A3 (Fragment) OS=Homo sapiens GN=ATP13A3 PE=4 SV=1 - [H7C1U7_HUMAN]	8,02	1
O95258	Brain mitochondrial carrier protein 1 OS=Homo sapiens GN=SLC25A14 PE=2 SV=1 - [UCP5_HUMAN]	8,00	1
P02652	Apolipoprotein A-II OS=Homo sapiens GN=APOA2 PE=1 SV=1 - [APOA2_HUMAN]	8,00	1
F8WBE0	Cytoskeleton-associated protein 2-like OS=Homo sapiens GN=CKAP2L PE=4 SV=1 - [F8WBE0_HUMAN]	7,98	1
Q9Y397	Palmitoyltransferase ZDHHC9 OS=Homo sapiens GN=ZDHHC9 PE=1 SV=2 - [ZDHC9_HUMAN]	7,97	1
Q9GZZ8	Extracellular glycoprotein lacritin OS=Homo sapiens GN=LACRT PE=1 SV=1 - [LACRT_HUMAN]	7,97	1
H0Y820	Serine/threonine-protein kinase Chk2 (Fragment) OS=Homo sapiens GN=CHEK2 PE=4 SV=1 - [H0Y820_HUMAN]	7,97	1
H7C2F2	CD99 antigen (Fragment) OS=Homo sapiens GN=CD99 PE=4 SV=1 - [H7C2F2_HUMAN]	7,96	1
O14683	Tumor protein p53-inducible protein 11 OS=Homo sapiens GN=TP53I11 PE=2 SV=2 - [P5I11_HUMAN]	7,94	1
G3V4Z4	Methylmalonate-semialdehyde dehydrogenase [acylating], mitochondrial OS=Homo sapiens GN=ALDH6A1 PE=3 SV=1 - [G3V4Z4_HUMAN	7,94	1
P51553	Isocitrate dehydrogenase [NAD] subunit gamma, mitochondrial OS=Homo sapiens GN=IDH3G PE=1 SV=1 - [IDH3G_HUMAN]	7,89	1
P48426	Phosphatidylinositol 5-phosphate 4-kinase type-2 alpha OS=Homo sapiens GN=PIP4K2A PE=1 SV=2 - [PI42A_HUMAN]	7,88	1
Q9Y5J5	Pleckstrin homology-like domain family A member 3 OS=Homo sapiens GN=PHLDA3 PE=1 SV=1 - [PHLA3_HUMAN]	7,87	1
P51397	Death-associated protein 1 OS=Homo sapiens GN=DAP PE=1 SV=3 - [DAP1_HUMAN]	7,84	1
O43709	Uncharacterized methyltransferase WBSCR22 OS=Homo sapiens GN=WBSCR22 PE=1 SV=2 - [WBS22_HUMAN]	7,83	1
H0YDV2	Coiled-coil domain-containing protein 73 (Fragment) OS=Homo sapiens GN=CCDC73 PE=4 SV=1 - [H0YDV2_HUMAN]	7,83	1
O60888	Protein CutA OS=Homo sapiens GN=CUTA PE=1 SV=2 - [CUTA_HUMAN]	7,82	1
Q9Y5W9	Sorting nexin-11 OS=Homo sapiens GN=SNX11 PE=2 SV=2 - [SNX11_HUMAN]	7,78	1
Q70IA6-2	Isoform 2 of MOB kinase activator 2 OS=Homo sapiens GN=MOB2 - [MOB2_HUMAN]	7,75	1
Q99643	Succinate dehydrogenase cytochrome b560 subunit, mitochondrial OS=Homo sapiens GN=SDHC PE=1 SV=1 - [C560_HUMAN]	7,69	1
P55957	BH3-interacting domain death agonist OS=Homo sapiens GN=BID PE=1 SV=1 - [BID_HUMAN]	7,69	1
Q9BQD7	Protein FAM173A OS=Homo sapiens GN=FAM173A PE=2 SV=1 - [F173A_HUMAN]	7,66	1
Q9H773	dCTP pyrophosphatase 1 OS=Homo sapiens GN=DCTPP1 PE=1 SV=1 - [DCTP1_HUMAN]	7,65	1
Q9NRR5	Ubiquilin-4 OS=Homo sapiens GN=UBQLN4 PE=1 SV=2 - [UBQL4_HUMAN]	7,65	1
P12035	Keratin, type II cytoskeletal 3 OS=Homo sapiens GN=KRT3 PE=1 SV=3 - [K2C3_HUMAN]	7,64	1
P11441	Ubiquitin-like protein 4A OS=Homo sapiens GN=UBL4A PE=1 SV=1 - [UBL4A_HUMAN]	7,64	1
P36639	7,8-dihydro-8-oxoguanine triphosphatase OS=Homo sapiens GN=NUDT1 PE=1 SV=3 - [8ODP_HUMAN]	7,61	1
O15127	Secretory carrier-associated membrane protein 2 OS=Homo sapiens GN=SCAMP2 PE=1 SV=2 - [SCAM2_HUMAN]	7,60	1
C9IZ32	TBC1 domain family member 23 (Fragment) OS=Homo sapiens GN=TBC1D23 PE=4 SV=1 - [C9IZ32_HUMAN]	7,60	1
Q9HCP0	Casein kinase I isoform gamma-1 OS=Homo sapiens GN=CSNK1G1 PE=1 SV=1 - [KC1G1_HUMAN]	7,58	1
O95471	Claudin-7 OS=Homo sapiens GN=CLDN7 PE=1 SV=4 - [CLD7_HUMAN]	7,58	1
Q5T013	Putative hydroxypyruvate isomerase OS=Homo sapiens GN=HYI PE=2 SV=2 - [HYI_HUMAN]	7,58	1
Q9UNL2	Translocon-associated protein subunit gamma OS=Homo sapiens GN=SSR3 PE=1 SV=1 - [SSRG_HUMAN]	7,57	1
P14209	CD99 antigen OS=Homo sapiens GN=CD99 PE=1 SV=1 - [CD99_HUMAN]	7,57	1
Q86YL5	Uncharacterized protein C8orf42 OS=Homo sapiens GN=C8orf42 PE=2 SV=2 - [CH042_HUMAN]	7,57	1
O75204	Transmembrane protein 127 OS=Homo sapiens GN=TMEM127 PE=1 SV=1 - [TM127_HUMAN]	7,56	1
Q9H8T0	AKT-interacting protein OS=Homo sapiens GN=AKTIP PE=1 SV=1 - [AKTIP_HUMAN]	7,53	1
P61077	Ubiquitin-conjugating enzyme E2 D3 OS=Homo sapiens GN=UBE2D3 PE=1 SV=1 - [UB2D3_HUMAN]	7,48	1
P40121	Macrophage-capping protein OS=Homo sapiens GN=CAPG PE=1 SV=2 - [CAPG_HUMAN]	7,47	1
Q96CE8	Transmembrane 4 L6 family member 18 OS=Homo sapiens GN=TM4SF18 PE=2 SV=1 - [T4S18_HUMAN]	7,46	1
E5RHY1	Suppression of tumorigenicity 18 protein OS=Homo sapiens GN=ST18 PE=4 SV=1 - [E5RHY1_HUMAN]	7,45	1
Q8IV50	LysM and putative peptidoglycan-binding domain-containing protein 2 OS=Homo sapiens GN=LYSMD2 PE=1 SV=1 - [LYSM2_HUMAN]	7,44	1
Q96I45	Transmembrane protein 141 OS=Homo sapiens GN=TMEM141 PE=2 SV=1 - [TM141_HUMAN]	7,41	1
Q96CG8	Collagen triple helix repeat-containing protein 1 OS=Homo sapiens GN=CTHRC1 PE=1 SV=1 - [CTHR1_HUMAN]	7,41	1
Q9H2C2	Protein ARV1 OS=Homo sapiens GN=ARV1 PE=2 SV=1 - [ARV1_HUMAN]	7,38	1
C9JJQ5	Ras-related protein Rab-19 (Fragment) OS=Homo sapiens GN=RAB19 PE=3 SV=1 - [C9JJQ5_HUMAN]	7,38	1
Q5BVD1	TPA-induced transmembrane protein OS=Homo sapiens GN=TTMP PE=2 SV=2 - [TTMP_HUMAN]	7,37	1
P06132	Uroporphyrinogen decarboxylase OS=Homo sapiens GN=UROD PE=1 SV=2 - [DCUP_HUMAN]	7,36	1
Q9BV68	RING finger protein 126 OS=Homo sapiens GN=RNF126 PE=1 SV=1 - [RN126_HUMAN]	7,36	1
Q49AR2-3	Isoform 3 of UPF0489 protein C5orf22 OS=Homo sapiens GN=C5orf22 - [CE022_HUMAN]	7,36	1
Q9UHA3	Probable ribosome biogenesis protein RLP24 OS=Homo sapiens GN=RSL24D1 PE=1 SV=1 - [RLP24_HUMAN]	7,36	1
F5GWT5	Steroid hormone receptor ERR1 (Fragment) OS=Homo sapiens GN=ESRRA PE=3 SV=1 - [F5GWT5_HUMAN]	7,34	1
H0YB13	Stanniocalcin-2 (Fragment) OS=Homo sapiens GN=STC2 PE=4 SV=1 - [H0YB13_HUMAN]	7,32	1
Q96HA8	Protein N-terminal glutamine amidohydrolase OS=Homo sapiens GN=WDYHV1 PE=1 SV=2 - [NTAQ1_HUMAN]	7,32	1
Q86TS9	39S ribosomal protein L52, mitochondrial OS=Homo sapiens GN=MRPL52 PE=2 SV=2 - [RM52_HUMAN]	7,32	1
Q7Z7N9	Transmembrane protein 179B OS=Homo sapiens GN=TMEM179B PE=1 SV=1 - [T179B_HUMAN]	7,31	1
E9PN59	Fas apoptotic inhibitory molecule 3 (Fragment) OS=Homo sapiens GN=FAIM3 PE=4 SV=1 - [E9PN59_HUMAN]	7,28	1
Q5T9P3	Fas (TNF receptor superfamily, member 6) OS=Homo sapiens GN=FAS PE=2 SV=1 - [Q5T9P3_HUMAN]	7,27	1
F8WCR2	Ubiquinol-cytochrome c reductase complex chaperone CBP3 homolog OS=Homo sapiens GN=UQCC PE=4 SV=1 - [F8WCR2_HUMAN]	7,26	1
P17676	CCAAT/enhancer-binding protein beta OS=Homo sapiens GN=CEBPB PE=1 SV=2 - [CEBPB_HUMAN]	7,25	1
E7EV32	TRAF family member-associated NF-kappa-B activator (Fragment) OS=Homo sapiens GN=TANK PE=4 SV=1 - [E7EV32_HUMAN]	7,25	1
Q9H0U6	39S ribosomal protein L18, mitochondrial OS=Homo sapiens GN=MRPL18 PE=1 SV=1 - [RM18_HUMAN]	7,22	1
Q6P2H8	Transmembrane protein 53 OS=Homo sapiens GN=TMEM53 PE=2 SV=1 - [TMM53_HUMAN]	7,22	1
Q96N06	Uncharacterized protein C16orf55 OS=Homo sapiens GN=C16orf55 PE=2 SV=1 - [CP055_HUMAN]	7,19	1
H3BVA4	F-box only protein 22 (Fragment) OS=Homo sapiens GN=FBXO22 PE=4 SV=1 - [H3BVA4_HUMAN]	7,19	1
P56211	cAMP-regulated phosphoprotein 19 OS=Homo sapiens GN=ARPP19 PE=1 SV=2 - [ARP19_HUMAN]	7,14	1
F5GZP3	Tripartite motif-containing protein 59 OS=Homo sapiens GN=TRIM59 PE=4 SV=1 - [F5GZP3_HUMAN]	7,12	1
Q9GZP9	Derlin-2 OS=Homo sapiens GN=DERL2 PE=1 SV=1 - [DERL2_HUMAN]	7,11	1
Q53RE8	Ankyrin repeat domain-containing protein 39 OS=Homo sapiens GN=ANKRD39 PE=1 SV=1 - [ANR39_HUMAN]	7,10	1
Q8WVK2	U4/U6.U5 small nuclear ribonucleoprotein 27 kDa protein OS=Homo sapiens GN=SNRNP27 PE=1 SV=1 - [SNR27_HUMAN]	7,10	1
P01903	HLA class II histocompatibility antigen, DR alpha chain OS=Homo sapiens GN=HLA-DRA PE=1 SV=1 - [DRA_HUMAN]	7,09	1
Q9NWH2	Transmembrane protein 242 OS=Homo sapiens GN=TMEM242 PE=2 SV=1 - [TM242_HUMAN]	7,09	1
C9JGH7	Origin recognition complex subunit 4 (Fragment) OS=Homo sapiens GN=ORC4 PE=4 SV=1 - [C9JGH7_HUMAN]	7,09	1
H0YHR9	N-alpha-acetyltransferase 25, NatB auxiliary subunit (Fragment) OS=Homo sapiens GN=NAA25 PE=4 SV=1 - [H0YHR9_HUMAN]	7,09	1
Q9NQ34	Transmembrane protein 9B OS=Homo sapiens GN=TMEM9B PE=1 SV=1 - [TMM9B_HUMAN]	7,07	1
Q0VGL1	UPF0539 protein C7orf59 OS=Homo sapiens GN=C7orf59 PE=1 SV=1 - [CG059_HUMAN]	7,07	1
O60635	Tetraspanin-1 OS=Homo sapiens GN=TSPAN1 PE=1 SV=2 - [TSN1_HUMAN]	7,05	1
P53680	AP-2 complex subunit sigma OS=Homo sapiens GN=AP2S1 PE=1 SV=2 - [AP2S1_HUMAN]	7,04	1
Q9UI15	Transgelin-3 OS=Homo sapiens GN=TAGLN3 PE=1 SV=2 - [TAGL3_HUMAN]	7,04	1
O43414-3	Isoform 3 of ERI1 exoribonuclease 3 OS=Homo sapiens GN=ERI3 - [ERI3_HUMAN]	7,03	1
Q86Y79	Probable peptidyl-tRNA hydrolase OS=Homo sapiens GN=PTRH1 PE=1 SV=1 - [PTH_HUMAN]	7,01	1
P0CG38	POTE ankyrin domain family member I OS=Homo sapiens GN=POTEI PE=3 SV=1 - [POTEI_HUMAN]	6,98	1
Q587I9	Vesicle transport protein SFT2C OS=Homo sapiens GN=SFT2D3 PE=2 SV=1 - [SFT2C_HUMAN]	6,98	1
P41208	Centrin-2 OS=Homo sapiens GN=CETN2 PE=1 SV=1 - [CETN2_HUMAN]	6,98	1
Q8NBZ0	INO80 complex subunit E OS=Homo sapiens GN=INO80E PE=1 SV=1 - [IN80E_HUMAN]	6,97	1
P40222	Alpha-taxilin OS=Homo sapiens GN=TXLNA PE=1 SV=3 - [TXLNA_HUMAN]	6,96	1
Q9GZM3	DNA-directed RNA polymerase II subunit RPB11-b1 OS=Homo sapiens GN=POLR2J2 PE=1 SV=1 - [RPB1B_HUMAN]	6,96	1
Q5JXX1	Mortality factor 4 like 2 (Fragment) OS=Homo sapiens GN=MORF4L2 PE=2 SV=1 - [Q5JXX1_HUMAN]	6,96	1
C9JHW1	PERQ amino acid-rich with GYF domain-containing protein 2 (Fragment) OS=Homo sapiens GN=GIGYF2 PE=4 SV=1 - [C9JHW1_HUMAN]	6,94	1
Q6QNY0	Biogenesis of lysosome-related organelles complex 1 subunit 3 OS=Homo sapiens GN=BLOC1S3 PE=1 SV=1 - [BL1S3_HUMAN]	6,93	1
F8WEG1	Transmembrane protein 184A OS=Homo sapiens GN=TMEM184A PE=4 SV=1 - [F8WEG1_HUMAN]	6,92	1
Q9BYC8	39S ribosomal protein L32, mitochondrial OS=Homo sapiens GN=MRPL32 PE=1 SV=1 - [RM32_HUMAN]	6,91	1
Q9H5X1	MIP18 family protein FAM96A OS=Homo sapiens GN=FAM96A PE=1 SV=1 - [FA96A_HUMAN]	6,88	1
O95857	Tetraspanin-13 OS=Homo sapiens GN=TSPAN13 PE=2 SV=1 - [TSN13_HUMAN]	6,86	1
Q9Y337	Kallikrein-5 OS=Homo sapiens GN=KLK5 PE=1 SV=2 - [KLK5_HUMAN]	6,83	1
Q5RL73	RNA-binding protein 48 OS=Homo sapiens GN=RBM48 PE=2 SV=1 - [RBM48_HUMAN]	6,81	1
O15525	Transcription factor MafG OS=Homo sapiens GN=MAFG PE=1 SV=1 - [MAFG_HUMAN]	6,79	1
C9JMA6	Cysteine synthase (Fragment) OS=Homo sapiens GN=CBS PE=3 SV=1 - [C9JMA6_HUMAN]	6,78	1
A6NIH7	Protein unc-119 homolog B OS=Homo sapiens GN=UNC119B PE=1 SV=1 - [U119B_HUMAN]	6,77	1
C9IZD2	DNA polymerase delta subunit 2 (Fragment) OS=Homo sapiens GN=POLD2 PE=4 SV=1 - [C9IZD2_HUMAN]	6,76	1
Q9Y2B2	N-acetylglucosaminyl-phosphatidylinositol de-N-acetylase OS=Homo sapiens GN=PIGL PE=2 SV=1 - [PIGL_HUMAN]	6,75	1
Q9NX01	Thioredoxin-like protein 4B OS=Homo sapiens GN=TXNL4B PE=1 SV=1 - [TXN4B_HUMAN]	6,71	1
Q9NV35	Probable 8-oxo-dGTP diphosphatase NUDT15 OS=Homo sapiens GN=NUDT15 PE=1 SV=1 - [NUD15_HUMAN]	6,71	1
Q9BUF7	Crumbs protein homolog 3 OS=Homo sapiens GN=CRB3 PE=1 SV=3 - [CRUM3_HUMAN]	6,67	1
E5RIY0	Transmembrane protein 55A OS=Homo sapiens GN=TMEM55A PE=4 SV=1 - [E5RIY0_HUMAN]	6,63	1
H0YI57	Beta-1,4 N-acetylgalactosaminyltransferase 1 (Fragment) OS=Homo sapiens GN=B4GALNT1 PE=4 SV=1 - [H0YI57_HUMAN]	6,62	1
H0YCA5	Spermatogenesis-associated protein 5-like protein 1 (Fragment) OS=Homo sapiens GN=SPATA5L1 PE=4 SV=1 - [H0YCA5_HUMAN]	6,59	1
Q96K21	Zinc finger FYVE domain-containing protein 19 OS=Homo sapiens GN=ZFYVE19 PE=1 SV=3 - [ZFY19_HUMAN]	6,58	1
Q92879	CUGBP Elav-like family member 1 OS=Homo sapiens GN=CELF1 PE=1 SV=2 - [CELF1_HUMAN]	6,58	1
H0YGG4	Translation initiation factor eIF-2B subunit alpha (Fragment) OS=Homo sapiens GN=EIF2B1 PE=4 SV=1 - [H0YGG4_HUMAN]	6,58	1
Q8N5M9	Protein jagunal homolog 1 OS=Homo sapiens GN=JAGN1 PE=1 SV=1 - [JAGN1_HUMAN]	6,56	1
Q9NX24	H/ACA ribonucleoprotein complex subunit 2 OS=Homo sapiens GN=NHP2 PE=1 SV=1 - [NHP2_HUMAN]	6,54	1
Q53S33	BolA-like protein 3 OS=Homo sapiens GN=BOLA3 PE=2 SV=1 - [BOLA3_HUMAN]	6,54	1
Q9Y3E0	Vesicle transport protein GOT1B OS=Homo sapiens GN=GOLT1B PE=1 SV=1 - [GOT1B_HUMAN]	6,52	1
Q9UM13	Anaphase-promoting complex subunit 10 OS=Homo sapiens GN=ANAPC10 PE=1 SV=1 - [APC10_HUMAN]	6,49	1
Q5SR36	E74-like factor 3 (Ets domain transcription factor, epithelial-specific) (Fragment) OS=Homo sapiens GN=ELF3 PE=2 SV=1 - [Q5SR36_H	6,47	1
F8W6E2	Uncharacterized protein C20orf26 (Fragment) OS=Homo sapiens GN=C20orf26 PE=4 SV=1 - [F8W6E2_HUMAN]	6,47	1
Q96CX2	BTB/POZ domain-containing protein KCTD12 OS=Homo sapiens GN=KCTD12 PE=1 SV=1 - [KCD12_HUMAN]	6,46	1
P54315-2	Isoform 2 of Pancreatic lipase-related protein 1 OS=Homo sapiens GN=PNLIPRP1 - [LIPR1_HUMAN]	6,45	1
P24001	Interleukin-32 OS=Homo sapiens GN=IL32 PE=1 SV=3 - [IL32_HUMAN]	6,41	1
C9JIV5	Sideroflexin-5 (Fragment) OS=Homo sapiens GN=SFXN5 PE=4 SV=1 - [C9JIV5_HUMAN]	6,41	1
Q9UMX5	Neudesin OS=Homo sapiens GN=NENF PE=1 SV=1 - [NENF_HUMAN]	6,40	1
P36954	DNA-directed RNA polymerase II subunit RPB9 OS=Homo sapiens GN=POLR2I PE=1 SV=1 - [RPB9_HUMAN]	6,40	1
A8MXV4	Nucleoside diphosphate-linked moiety X motif 19, mitochondrial OS=Homo sapiens GN=NUDT19 PE=1 SV=1 - [NUD19_HUMAN]	6,40	1
H7C1L7	N-acetyl-D-glucosamine kinase (Fragment) OS=Homo sapiens GN=NAGK PE=4 SV=1 - [H7C1L7_HUMAN]	6,40	1
Q9UJ68	Mitochondrial peptide methionine sulfoxide reductase OS=Homo sapiens GN=MSRA PE=1 SV=1 - [MSRA_HUMAN]	6,38	1
O95807	Transmembrane protein 50A OS=Homo sapiens GN=TMEM50A PE=1 SV=1 - [TM50A_HUMAN]	6,37	1
P16035	Metalloproteinase inhibitor 2 OS=Homo sapiens GN=TIMP2 PE=1 SV=2 - [TIMP2_HUMAN]	6,36	1
Q6P4I2	WD repeat-containing protein 73 OS=Homo sapiens GN=WDR73 PE=1 SV=1 - [WDR73_HUMAN]	6,35	1
Q8WUR7	UPF0235 protein C15orf40 OS=Homo sapiens GN=C15orf40 PE=1 SV=1 - [CO040_HUMAN]	6,35	1
O60234	Glia maturation factor gamma OS=Homo sapiens GN=GMFG PE=1 SV=1 - [GMFG_HUMAN]	6,34	1
Q9H741	UPF0454 protein C12orf49 OS=Homo sapiens GN=C12orf49 PE=2 SV=1 - [CL049_HUMAN]	6,34	1
Q6P1K2	Polyamine-modulated factor 1 OS=Homo sapiens GN=PMF1 PE=1 SV=2 - [PMF1_HUMAN]	6,34	1
B0QY75	Adenylosuccinate lyase (Fragment) OS=Homo sapiens GN=ADSL PE=4 SV=1 - [B0QY75_HUMAN]	6,32	1
P36383	Gap junction gamma-1 protein OS=Homo sapiens GN=GJC1 PE=1 SV=2 - [CXG1_HUMAN]	6,31	1

O43567	E3 ubiquitin-protein ligase RNF13 OS=Homo sapiens GN=RNF13 PE=1 SV=1 - [RNF13_HUMAN]	6,30	1	0,913
Q6IBS0	Twinfilin-2 OS=Homo sapiens GN=TWF2 PE=1 SV=2 - [TWF2_HUMAN]	6,30	1	
Q9UBY8	Protein CLN8 OS=Homo sapiens GN=CLN8 PE=1 SV=3 - [CLN8_HUMAN]	6,29	1	1,433
Q5VYY1	Ankyrin repeat domain-containing protein 22 OS=Homo sapiens GN=ANKRD22 PE=2 SV=1 - [ANR22_HUMAN]	6,28	1	0,156
Q969Z4	Tumor necrosis factor receptor superfamily member 19L OS=Homo sapiens GN=RELT PE=1 SV=1 - [TR19L_HUMAN]	6,28	1	0,763
P25208	Nuclear transcription factor Y subunit beta OS=Homo sapiens GN=NFYB PE=1 SV=2 - [NFYB_HUMAN]	6,28	1	
O75558	Syntaxin-11 OS=Homo sapiens GN=STX11 PE=2 SV=1 - [STX11_HUMAN]	6,27	1	1,199
A1L170	Uncharacterized protein C1orf226 OS=Homo sapiens GN=C1orf226 PE=1 SV=1 - [CA226_HUMAN]	6,25	1	0,672
Q969L2	Protein MAL2 OS=Homo sapiens GN=MAL2 PE=1 SV=1 - [MAL2_HUMAN]	6,25	1	0,404
Q9Y3B6	UPF0172 protein FAM158A OS=Homo sapiens GN=FAM158A PE=2 SV=3 - [F158A_HUMAN]	6,25	1	
Q9UGC6	Regulator of G-protein signaling 17 OS=Homo sapiens GN=RGS17 PE=1 SV=2 - [RGS17_HUMAN]	6,19	1	0,897
Q9NP77	RNA polymerase II subunit A C-terminal domain phosphatase SSU72 OS=Homo sapiens GN=SSU72 PE=1 SV=1 - [SSU72_HUMAN]	6,19	1	
P29558	RNA-binding motif, single-stranded-interacting protein 1 OS=Homo sapiens GN=RBMS1 PE=1 SV=3 - [RBMS1_HUMAN]	6,16	1	0,895
Q86TG7-2	Isoform RF1 of Retrotransposon-derived protein PEG10 OS=Homo sapiens GN=PEG10 - [PEG10_HUMAN]	6,15	1	
H0Y4Y8	Fibrinogen C domain-containing protein 1 (Fragment) OS=Homo sapiens GN=FIBCD1 PE=4 SV=1 - [H0Y4Y8_HUMAN]	6,13	1	0,662
Q3SY84	Keratin, type II cytoskeletal 71 OS=Homo sapiens GN=KRT71 PE=1 SV=3 - [K2C71_HUMAN]	6,12	1	
Q8N6M3	Fat storage-inducing transmembrane protein 2 OS=Homo sapiens GN=FITM2 PE=2 SV=1 - [FITM2_HUMAN]	6,11	1	0,960
H3BSB3	M-phase phosphoprotein 6 OS=Homo sapiens GN=MPHOSPH6 PE=4 SV=1 - [H3BSB3_HUMAN]	6,11	1	
Q9UGM6	Tryptophan--tRNA ligase, mitochondrial OS=Homo sapiens GN=WARS2 PE=1 SV=1 - [SYWM_HUMAN]	6,11	1	
B4DJC6	Probable U3 small nucleolar RNA-associated protein 11 OS=Homo sapiens GN=UTP11L PE=2 SV=1 - [B4DJC6_HUMAN]	6,08	1	
Q5U3C3	Transmembrane protein 164 OS=Homo sapiens GN=TMEM164 PE=1 SV=1 - [TM164_HUMAN]	6,06	1	0,444
B3KSD6	Leucine zipper protein 4 OS=Homo sapiens GN=LUZP4 PE=2 SV=1 - [B3KSD6_HUMAN]	6,06	1	
O60487	Myelin protein zero-like protein 2 OS=Homo sapiens GN=MPZL2 PE=1 SV=1 - [MPZL2_HUMAN]	6,05	1	0,180
Q8IX05	CD302 antigen OS=Homo sapiens GN=CD302 PE=1 SV=1 - [CD302_HUMAN]	6,03	1	1,106
P0CK97	Solute carrier family 35 member E2 OS=Homo sapiens GN=SLC35E2 PE=2 SV=1 - [S35E2_HUMAN]	6,02	1	1,104
Q6P9G4	Transmembrane protein 154 OS=Homo sapiens GN=TMEM154 PE=2 SV=2 - [TM154_HUMAN]	6,01	1	
P49447	Cytochrome b561 OS=Homo sapiens GN=CYB561 PE=2 SV=2 - [CY561_HUMAN]	5,98	1	0,741
O60282	Kinesin heavy chain isoform 5C OS=Homo sapiens GN=KIF5C PE=1 SV=1 - [KIF5C_HUMAN]	5,96	1	
Q86TN4	tRNA 2'-phosphotransferase 1 OS=Homo sapiens GN=TRPT1 PE=1 SV=2 - [TRPT1_HUMAN]	5,93	1	
Q8N2U9	PQ-loop repeat-containing protein 1 OS=Homo sapiens GN=PQLC1 PE=2 SV=1 - [PQLC1_HUMAN]	5,90	1	0,989
O43741	5'-AMP-activated protein kinase subunit beta-2 OS=Homo sapiens GN=PRKAB2 PE=1 SV=1 - [AAKB2_HUMAN]	5,88	1	0,575
Q5RKV6	Exosome complex component MTR3 OS=Homo sapiens GN=EXOSC6 PE=1 SV=1 - [EXOS6_HUMAN]	5,88	1	0,932
P18440	Arylamine N-acetyltransferase 1 OS=Homo sapiens GN=NAT1 PE=1 SV=2 - [ARY1_HUMAN]	5,86	1	0,708
H0Y8H1	Cysteine-rich hydrophobic domain 2 protein (Fragment) OS=Homo sapiens GN=CHIC2 PE=4 SV=1 - [H0Y8H1_HUMAN]	5,84	1	0,885
Q13432	Protein unc-119 homolog A OS=Homo sapiens GN=UNC119 PE=1 SV=1 - [U119A_HUMAN]	5,83	1	
O95758	Polypyrimidine tract-binding protein 3 OS=Homo sapiens GN=PTBP3 PE=1 SV=2 - [PTBP3_HUMAN]	5,80	1	
Q96MW1	Coiled-coil domain-containing protein 43 OS=Homo sapiens GN=CCDC43 PE=1 SV=2 - [CCD43_HUMAN]	5,80	1	
O00221	NF-kappa-B inhibitor epsilon OS=Homo sapiens GN=NFKBIE PE=1 SV=3 - [IKBE_HUMAN]	5,80	1	
P48730	Casein kinase I isoform delta OS=Homo sapiens GN=CSNK1D PE=1 SV=2 - [KC1D_HUMAN]	5,78	1	
Q8N2H4	Protein SYS1 homolog OS=Homo sapiens GN=SYS1 PE=1 SV=1 - [SYS1_HUMAN]	5,77	1	1,319
P49674	Casein kinase I isoform epsilon OS=Homo sapiens GN=CSNK1E PE=1 SV=1 - [KC1E_HUMAN]	5,77	1	
Q00535-2	Isoform 2 of Cyclin-dependent kinase 5 OS=Homo sapiens GN=CDK5 - [CDK5_HUMAN]	5,77	1	
P23258	Tubulin gamma-1 chain OS=Homo sapiens GN=TUBG1 PE=1 SV=2 - [TBG1_HUMAN]	5,76	1	0,748
O75340	Programmed cell death protein 6 OS=Homo sapiens GN=PDCD6 PE=1 SV=1 - [PDCD6_HUMAN]	5,76	1	
P13051	Uracil-DNA glycosylase OS=Homo sapiens GN=UNG PE=1 SV=2 - [UNG_HUMAN]	5,75	1	0,600
Q9GZN8	UPF0687 protein C20orf27 OS=Homo sapiens GN=C20orf27 PE=1 SV=3 - [CT027_HUMAN]	5,75	1	
Q12999	Tetraspanin-31 OS=Homo sapiens GN=TSPAN31 PE=2 SV=1 - [TSN31_HUMAN]	5,71	1	0,862
Q96T52	Mitochondrial inner membrane protease subunit 2 OS=Homo sapiens GN=IMMP2L PE=2 SV=1 - [IMP2L_HUMAN]	5,71	1	
Q9H7E9	UPF0488 protein C8orf33 OS=Homo sapiens GN=C8orf33 PE=1 SV=1 - [CH033_HUMAN]	5,68	1	
Q71F23-2	Isoform 2 of Centromere protein U OS=Homo sapiens GN=MLF1IP - [CENPU_HUMAN]	5,68	1	
Q9Y4U1	Methylmalonic aciduria and homocystinuria type C protein OS=Homo sapiens GN=MMACHC PE=1 SV=3 - [MMAC_HUMAN]	5,67	1	0,586
Q13595	Transformer-2 protein homolog alpha OS=Homo sapiens GN=TRA2A PE=1 SV=1 - [TRA2A_HUMAN]	5,67	1	
Q13901	Nuclear nucleic acid-binding protein C1D OS=Homo sapiens GN=C1D PE=1 SV=1 - [C1D_HUMAN]	5,67	1	
P01135	Protransforming growth factor alpha OS=Homo sapiens GN=TGFA PE=1 SV=1 - [TGFA_HUMAN]	5,63	1	1,033
B7ZBK4	General transcription factor IIIA (Fragment) OS=Homo sapiens GN=GTF3A PE=4 SV=1 - [B7ZBK4_HUMAN]	5,62	1	
Q92882	Osteoclast-stimulating factor 1 OS=Homo sapiens GN=OSTF1 PE=1 SV=2 - [OSTF1_HUMAN]	5,61	1	0,598
Q15437	Protein transport protein Sec23B OS=Homo sapiens GN=SEC23B PE=1 SV=2 - [SC23B_HUMAN]	5,61	1	0,220
Q9Y5N5	HemK methyltransferase family member 2 OS=Homo sapiens GN=N6AMT1 PE=1 SV=3 - [HEMK2_HUMAN]	5,61	1	
Q0GE19	Sodium/bile acid cotransporter 7 OS=Homo sapiens GN=SLC10A7 PE=2 SV=1 - [NTCP7_HUMAN]	5,59	1	0,749
Q96MG7	Melanoma-associated antigen G1 OS=Homo sapiens GN=NDNL2 PE=1 SV=1 - [MAGG1_HUMAN]	5,59	1	
O95456	Proteasome assembly chaperone 1 OS=Homo sapiens GN=PSMG1 PE=1 SV=1 - [PSMG1_HUMAN]	5,56	1	0,858
E7EQD2	Solute carrier family 43 member 3 OS=Homo sapiens GN=SLC43A3 PE=4 SV=1 - [E7EQD2_HUMAN]	5,56	1	
Q9Y221	60S ribosome subunit biogenesis protein NIP7 homolog OS=Homo sapiens GN=NIP7 PE=1 SV=1 - [NIP7_HUMAN]	5,56	1	
Q99967	Cbp/p300-interacting transactivator 2 OS=Homo sapiens GN=CITED2 PE=1 SV=2 - [CITE2_HUMAN]	5,56	1	
P62995	Transformer-2 protein homolog beta OS=Homo sapiens GN=TRA2B PE=1 SV=1 - [TRA2B_HUMAN]	5,56	1	
Q6UWV2	Myelin protein zero-like protein 3 OS=Homo sapiens GN=MPZL3 PE=1 SV=1 - [MPZL3_HUMAN]	5,53	1	
Q5M7Z0	RING finger and transmembrane domain-containing protein 1 OS=Homo sapiens GN=RNFT1 PE=2 SV=2 - [RNFT1_HUMAN]	5,52	1	0,959
Q96GZ6	Solute carrier family 41 member 3 OS=Homo sapiens GN=SLC41A3 PE=2 SV=2 - [S41A3_HUMAN]	5,52	1	1,500
P61218	DNA-directed RNA polymerases I, II, and III subunit RPABC2 OS=Homo sapiens GN=POLR2F PE=1 SV=1 - [RPAB2_HUMAN]	5,51	1	
F8W8E2	Cytohesin-2 (Fragment) OS=Homo sapiens GN=CYTH2 PE=4 SV=1 - [F8W8E2_HUMAN]	5,51	1	
E9PC50	PERQ amino acid-rich with GYF domain-containing protein 2 OS=Homo sapiens GN=GIGYF2 PE=4 SV=2 - [E9PC50_HUMAN]	5,50	1	1,358
Q86XS8	E3 ubiquitin-protein ligase RNF130 OS=Homo sapiens GN=RNF130 PE=1 SV=1 - [GOLI_HUMAN]	5,49	1	0,953
Q5TC12	ATP synthase mitochondrial F1 complex assembly factor 1 OS=Homo sapiens GN=ATPAF1 PE=1 SV=1 - [ATPF1_HUMAN]	5,49	1	0,757
I3L410	PHD finger protein 23 (Fragment) OS=Homo sapiens GN=PHF23 PE=4 SV=1 - [I3L410_HUMAN]	5,47	1	
Q9NP55	BPI fold-containing family A member 1 OS=Homo sapiens GN=BPIFA1 PE=1 SV=1 - [BPIA1_HUMAN]	5,47	1	
Q9NX76	CKLF-like MARVEL transmembrane domain-containing protein 6 OS=Homo sapiens GN=CMTM6 PE=1 SV=1 - [CKLF6_HUMAN]	5,46	1	1,159
P62256	Ubiquitin-conjugating enzyme E2 H OS=Homo sapiens GN=UBE2H PE=1 SV=1 - [UBE2H_HUMAN]	5,46	1	
Q712K3	Ubiquitin-conjugating enzyme E2 R2 OS=Homo sapiens GN=UBE2R2 PE=1 SV=1 - [UB2R2_HUMAN]	5,46	1	
O75446	Histone deacetylase complex subunit SAP30 OS=Homo sapiens GN=SAP30 PE=1 SV=1 - [SAP30_HUMAN]	5,45	1	0,781
Q9NU53	Uncharacterized protein C6orf72 OS=Homo sapiens GN=C6orf72 PE=2 SV=1 - [CF072_HUMAN]	5,45	1	1,056
Q92903	Phosphatidate cytidylyltransferase 1 OS=Homo sapiens GN=CDS1 PE=2 SV=2 - [CDS1_HUMAN]	5,42	1	
Q86YH6-2	Isoform 2 of Decaprenyl-diphosphate synthase subunit 2 OS=Homo sapiens GN=PDSS2 - [DLP1_HUMAN]	5,42	1	
A6NCS6	Uncharacterized protein C2orf72 OS=Homo sapiens GN=C2orf72 PE=2 SV=2 - [CB072_HUMAN]	5,42	1	
Q9H3J6	Probable peptide chain release factor C12orf65, mitochondrial OS=Homo sapiens GN=C12orf65 PE=2 SV=1 - [CL065_HUMAN]	5,42	1	
Q8N2K1	Ubiquitin-conjugating enzyme E2 J2 OS=Homo sapiens GN=UBE2J2 PE=1 SV=3 - [UB2J2_HUMAN]	5,41	1	1,041
A6NG35	Cyclic AMP-dependent transcription factor ATF-6 beta OS=Homo sapiens GN=ATF6B PE=4 SV=2 - [A6NG35_HUMAN]	5,41	1	0,938
Q96EL2	28S ribosomal protein S24, mitochondrial OS=Homo sapiens GN=MRPS24 PE=1 SV=1 - [RT24_HUMAN]	5,39	1	1,053
J3KTG5	Nuclear prelamin A recognition factor (Fragment) OS=Homo sapiens GN=NARF PE=4 SV=1 - [J3KTG5_HUMAN]	5,39	1	
Q9Y294	Histone chaperone ASF1A OS=Homo sapiens GN=ASF1A PE=1 SV=1 - [ASF1A_HUMAN]	5,39	1	
Q9BV86	Alpha N-terminal protein methyltransferase 1A OS=Homo sapiens GN=METTL11A PE=1 SV=3 - [NTM1A_HUMAN]	5,38	1	1,018
P00414	Cytochrome c oxidase subunit 3 OS=Homo sapiens GN=MT-CO3 PE=1 SV=2 - [COX3_HUMAN]	5,36	1	1,148
Q9P0S9	Transmembrane protein 14C OS=Homo sapiens GN=TMEM14C PE=1 SV=1 - [TM14C_HUMAN]	5,36	1	1,749
O00458	Interferon-related developmental regulator 1 OS=Homo sapiens GN=IFRD1 PE=1 SV=4 - [IFRD1_HUMAN]	5,32	1	1,169
Q14442	Phosphatidylinositol N-acetylglucosaminyltransferase subunit H OS=Homo sapiens GN=PIGH PE=1 SV=1 - [PIGH_HUMAN]	5,32	1	0,890
Q9NZE8	39S ribosomal protein L35, mitochondrial OS=Homo sapiens GN=MRPL35 PE=2 SV=3 - [RM35_HUMAN]	5,32	1	1,239
P82970	High mobility group nucleosome-binding domain-containing protein 5 OS=Homo sapiens GN=HMGN5 PE=1 SV=1 - [HMGN5_HUMAN]	5,32	1	
Q8N6S5	ADP-ribosylation factor-like protein 6-interacting protein 6 OS=Homo sapiens GN=ARL6IP6 PE=1 SV=1 - [AR6P6_HUMAN]	5,31	1	0,849
O60573	Eukaryotic translation initiation factor 4E type 2 OS=Homo sapiens GN=EIF4E2 PE=1 SV=1 - [IF4E2_HUMAN]	5,31	1	
Q9H4F1	Alpha-N-acetyl-neuraminyl-2,3-beta-galactosyl-1,3-N-acetyl-galactosaminide alpha-2,6-sialyltransferase OS=Homo sapiens GN=ST6GALN	5,30	1	1,253
Q63HQ0	AP-1 complex-associated regulatory protein OS=Homo sapiens GN=AP1AR PE=1 SV=1 - [AP1AR_HUMAN]	5,30	1	
O14791	Apolipoprotein L1 OS=Homo sapiens GN=APOL1 PE=1 SV=5 - [APOL1_HUMAN]	5,28	1	
O14880	Microsomal glutathione S-transferase 3 OS=Homo sapiens GN=MGST3 PE=1 SV=1 - [MGST3_HUMAN]	5,26	1	1,961
Q8WZA0	Protein LZIC OS=Homo sapiens GN=LZIC PE=1 SV=1 - [LZIC_HUMAN]	5,26	1	
Q9H4A5	Golgi phosphoprotein 3-like OS=Homo sapiens GN=GOLPH3L PE=1 SV=1 - [GLP3L_HUMAN]	5,26	1	
Q92804	TATA-binding protein-associated factor 2N OS=Homo sapiens GN=TAF15 PE=1 SV=1 - [RBP56_HUMAN]	5,24	1	0,964
Q9HAF1	Chromatin modification-related protein MEAF6 OS=Homo sapiens GN=MEAF6 PE=1 SV=1 - [EAF6_HUMAN]	5,24	1	
Q96E09	Protein FAM122A OS=Homo sapiens GN=FAM122A PE=1 SV=1 - [F122A_HUMAN]	5,23	1	
Q86U25	Full-length cDNA clone CS0DA007YG23 of Neuroblastoma of Homo sapiens (human) OS=Homo sapiens GN=TTC8 PE=2 SV=1 - [Q86U25	5,21	1	
P98172	Ephrin-B1 OS=Homo sapiens GN=EFNB1 PE=1 SV=1 - [EFNB1_HUMAN]	5,20	1	0,993
Q9Y6Q5	AP-1 complex subunit mu-2 OS=Homo sapiens GN=AP1M2 PE=1 SV=4 - [AP1M2_HUMAN]	5,20	1	
Q15102	Platelet-activating factor acetylhydrolase IB subunit gamma OS=Homo sapiens GN=PAFAH1B3 PE=1 SV=1 - [PA1B3_HUMAN]	5,19	1	
P54687	Branched-chain-amino-acid aminotransferase, cytosolic OS=Homo sapiens GN=BCAT1 PE=1 SV=3 - [BCAT1_HUMAN]	5,18	1	1,272
P59780	AP-3 complex subunit sigma-2 OS=Homo sapiens GN=AP3S2 PE=2 SV=1 - [AP3S2_HUMAN]	5,18	1	
Q9H3H3	UPF0696 protein C11orf68 OS=Homo sapiens GN=C11orf68 PE=1 SV=2 - [CK068_HUMAN]	5,18	1	
P57764	Gasdermin-D OS=Homo sapiens GN=GSDMD PE=1 SV=1 - [GSDMD_HUMAN]	5,17	1	
Q9NWS0	PIH1 domain-containing protein 1 OS=Homo sapiens GN=PIH1D1 PE=1 SV=1 - [PIHD1_HUMAN]	5,17	1	
Q9UJG1	Motile sperm domain-containing protein 1 OS=Homo sapiens GN=MOSPD1 PE=2 SV=1 - [MSPD1_HUMAN]	5,16	1	0,677
P05496	ATP synthase lipid-binding protein, mitochondrial OS=Homo sapiens GN=ATP5G1 PE=2 SV=2 - [AT5G1_HUMAN]	5,15	1	2,186
P16298	Serine/threonine-protein phosphatase 2B catalytic subunit beta isoform OS=Homo sapiens GN=PPP3CB PE=1 SV=2 - [PP2BB_HUMAN]	5,15	1	1,027
O95983	Methyl-CpG-binding domain protein 3 OS=Homo sapiens GN=MBD3 PE=1 SV=1 - [MBD3_HUMAN]	5,15	1	
P30559	Oxytocin receptor OS=Homo sapiens GN=OXTR PE=1 SV=2 - [OXYR_HUMAN]	5,14	1	1,386
Q9BWL3	Uncharacterized protein C1orf43 OS=Homo sapiens GN=C1orf43 PE=1 SV=1 - [CA043_HUMAN]	5,14	1	1,371
Q9NW97	Transmembrane protein 51 OS=Homo sapiens GN=TMEM51 PE=1 SV=1 - [TMM51_HUMAN]	5,14	1	0,936
Q9NR77	Peroxisomal membrane protein 2 OS=Homo sapiens GN=PXMP2 PE=1 SV=3 - [PXMP2_HUMAN]	5,13	1	1,045
Q5VZ19-2	Isoform 2 of Tudor domain-containing protein 10 OS=Homo sapiens GN=TDRD10 - [TDR10_HUMAN]	5,13	1	1,680
Q96EH3	Mitochondrial assembly of ribosomal large subunit protein 1 OS=Homo sapiens GN=MALSU1 PE=1 SV=1 - [MASU1_HUMAN]	5,13	1	
B4DV88	Peroxisomal membrane protein 11A OS=Homo sapiens GN=PEX11A PE=2 SV=1 - [B4DV88_HUMAN]	5,09	1	1,244
Q9H1Y0	Autophagy protein 5 OS=Homo sapiens GN=ATG5 PE=1 SV=2 - [ATG5_HUMAN]	5,09	1	
P41440	Folate transporter 1 OS=Homo sapiens GN=SLC19A1 PE=1 SV=3 - [S19A1_HUMAN]	5,08	1	0,888
Q9NPF4	Probable tRNA threonylcarbamoyladenosine biosynthesis protein OSGEP OS=Homo sapiens GN=OSGEP PE=1 SV=1 - [OSGEP_HUMAN]	5,07	1	
Q9NWZ3-2	Isoform 2 of Interleukin-1 receptor-associated kinase 4 OS=Homo sapiens GN=IRAK4 - [IRAK4_HUMAN]	5,06	1	1,132
Q86UY6	N-alpha-acetyltransferase 40 OS=Homo sapiens GN=NAA40 PE=2 SV=1 - [NAA40_HUMAN]	5,06	1	
Q9H974	Queuine tRNA-ribosyltransferase subunit QTRTD1 OS=Homo sapiens GN=QTRTD1 PE=1 SV=1 - [QTRD1_HUMAN]	5,06	1	
P27448	MAP/microtubule affinity-regulating kinase 3 OS=Homo sapiens GN=MARK3 PE=1 SV=4 - [MARK3_HUMAN]	5,05	1	
F8W1K6	Plexin-C1 OS=Homo sapiens GN=PLXNC1 PE=4 SV=1 - [F8W1K6_HUMAN]	5,05	1	
Q7L211	Abhydrolase domain-containing protein 13 OS=Homo sapiens GN=ABHD13 PE=1 SV=1 - [ABHDD_HUMAN]	5,04	1	0,906
Q9NWT8	Aurora kinase A-interacting protein OS=Homo sapiens GN=AURKAIP1 PE=1 SV=1 - [AKIP_HUMAN]	5,03	1	2,085
Q9Y6M9	NADH dehydrogenase [ubiquinone] 1 beta subcomplex subunit 9 OS=Homo sapiens GN=NDUFB9 PE=1 SV=3 - [NDUB9_HUMAN]	5,03	1	1,496
Q9H4A6	Golgi phosphoprotein 3 OS=Homo sapiens GN=GOLPH3 PE=1 SV=1 - [GOLP3_HUMAN]	5,03	1	
J3KSM2	MARVEL domain-containing protein 3 OS=Homo sapiens GN=MARVELD3 PE=4 SV=1 - [J3KSM2_HUMAN]	5,02	1	1,232
Q86U06	Probable RNA-binding protein 23 OS=Homo sapiens GN=RBM23 PE=1 SV=1 - [RBM23_HUMAN]	5,01	1	
H0YF22	REST corepressor 3 (Fragment) OS=Homo sapiens GN=RCOR3 PE=4 SV=1 - [H0YF22_HUMAN]	5,00	1	
H3BUB4	Snurportin-1 (Fragment) OS=Homo sapiens GN=SNUPN PE=4 SV=1 - [H3BUB4_HUMAN]	5,00	1	
P56937	3-keto-steroid reductase OS=Homo sapiens GN=HSD17B7 PE=1 SV=1 - [DHB7_HUMAN]	4,99	1	
Q9BUA3	Uncharacterized protein C11orf84 OS=Homo sapiens GN=C11orf84 PE=1 SV=3 - [CK084_HUMAN]	4,99	1	
D3YTH9	NADPH-dependent diflavin oxidoreductase 1 OS=Homo sapiens GN=NDOR1 PE=4 SV=1 - [D3YTH9_HUMAN]	4,99	1	
Q96CB8	Integrator complex subunit 12 OS=Homo sapiens GN=INTS12 PE=1 SV=1 - [INT12_HUMAN]	4,98	1	
Q9BR61	Acyl-CoA-binding domain-containing protein 6 OS=Homo sapiens GN=ACBD6 PE=1 SV=1 - [ACBD6_HUMAN]	4,96	1	
Q96SI1	BTB/POZ domain-containing protein KCTD15 OS=Homo sapiens GN=KCTD15 PE=1 SV=1 - [KCD15_HUMAN]	4,95	1	
Q8WUD6	Cholinephosphotransferase 1 OS=Homo sapiens GN=CHPT1 PE=1 SV=1 - [CHPT1_HUMAN]	4,93	1	1,322
P83876	Thioredoxin-like protein 4A OS=Homo sapiens GN=TXNL4A PE=1 SV=1 - [TXN4A_HUMAN]	4,93	1	
Q9H9Q2	COP9 signalosome complex subunit 7b OS=Homo sapiens GN=COPS7B PE=1 SV=1 - [CSN7B_HUMAN]	4,92	1	0,801
Q9NUI1-3	Isoform 3 of Peroxisomal 2,4-dienoyl-CoA reductase OS=Homo sapiens GN=DECR2 - [DECR2_HUMAN]	4,92	1	1,671
Q8NCG5	Carbohydrate sulfotransferase 4 OS=Homo sapiens GN=CHST4 PE=1 SV=2 - [CHST4_HUMAN]	4,92	1	
Q9UIV1	CCR4-NOT transcription complex subunit 7 OS=Homo sapiens GN=CNOT7 PE=1 SV=3 - [CNOT7_HUMAN]	4,91	1	
Q56P03	E2F-associated phosphoprotein OS=Homo sapiens GN=EAPP PE=1 SV=4 - [EAPP_HUMAN]	4,91	1	
Q9Y448-2	Isoform 2 of Small kinetochore-associated protein OS=Homo sapiens GN=SKAP - [SKAP_HUMAN]	4,90	1	
Q9NUM3	Zinc transporter ZIP9 OS=Homo sapiens GN=SLC39A9 PE=2 SV=2 - [S39A9_HUMAN]	4,89	1	1,632
P15428	15-hydroxyprostaglandin dehydrogenase [NAD(+)] OS=Homo sapiens GN=HPGD PE=1 SV=1 - [PGDH_HUMAN]	4,89	1	0,396
Q13454	Tumor suppressor candidate 3 OS=Homo sapiens GN=TUSC3 PE=1 SV=1 - [TUSC3_HUMAN]	4,89	1	**2,522**
O43251-6	Isoform 6 of RNA binding protein fox-1 homolog 2 OS=Homo sapiens GN=RBFOX2 - [RFOX2_HUMAN]	4,89	1	
Q13188	Serine/threonine-protein kinase 3 OS=Homo sapiens GN=STK3 PE=1 SV=2 - [STK3_HUMAN]	4,89	1	
Q9NYZ1	Protein FAM18B1 OS=Homo sapiens GN=FAM18B1 PE=2 SV=2 - [F18B1_HUMAN]	4,88	1	0,747
Q7L804	Rab11 family-interacting protein 2 OS=Homo sapiens GN=RAB11FIP2 PE=1 SV=1 - [RFIP2_HUMAN]	4,88	1	0,939
Q8WWY8	Lipase member H OS=Homo sapiens GN=LIPH PE=1 SV=1 - [LIPH_HUMAN]	4,88	1	
G3V2F7	HCG2044781 OS=Homo sapiens GN=TMEM189 PE=4 SV=1 - [G3V2F7_HUMAN]	4,86	1	
B7Z4R0	ETS domain-containing transcription factor ERF OS=Homo sapiens GN=ERF PE=2 SV=1 - [B7Z4R0_HUMAN]	4,86	1	
Q15819	Ubiquitin-conjugating enzyme E2 variant 2 OS=Homo sapiens GN=UBE2V2 PE=1 SV=4 - [UB2V2_HUMAN]	4,83	1	
Q9NYT0	Pleckstrin-2 OS=Homo sapiens GN=PLEK2 PE=1 SV=1 - [PLEK2_HUMAN]	4,82	1	0,429
Q9BRU9	rRNA-processing protein UTP23 homolog OS=Homo sapiens GN=UTP23 PE=2 SV=2 - [UTP23_HUMAN]	4,82	1	

Q00169	Phosphatidylinositol transfer protein alpha isoform OS=Homo sapiens GN=PITPNA PE=1 SV=2 - [PIPNA_HUMAN]	4,81	1	0,709
Q8N205-2	Isoform 2 of Nesprin-4 OS=Homo sapiens GN=SYNE4 - [SYNE4_HUMAN]	4,81	1	
Q15007	Pre-mRNA-splicing regulator WTAP OS=Homo sapiens GN=WTAP PE=1 SV=2 - [FL2D_HUMAN]	4,80	1	1,101
F8W6Z1	Cystinosin OS=Homo sapiens GN=CTNS PE=4 SV=1 - [F8W6Z1_HUMAN]	4,79	1	1,826
Q13596	Sorting nexin-1 OS=Homo sapiens GN=SNX1 PE=1 SV=3 - [SNX1_HUMAN]	4,79	1	
Q9H1K1	Iron-sulfur cluster assembly enzyme ISCU, mitochondrial OS=Homo sapiens GN=ISCU PE=1 SV=2 - [ISCU_HUMAN]	4,79	1	
P42575-2	Isoform 2 of Caspase-2 OS=Homo sapiens GN=CASP2 - [CASP2_HUMAN]	4,79	1	
C9J6G3	Geranylgeranyl pyrophosphate synthase (Fragment) OS=Homo sapiens GN=GGPS1 PE=3 SV=1 - [C9J6G3_HUMAN]	4,78	1	0,889
O00212	Rho-related GTP-binding protein RhoD OS=Homo sapiens GN=RHOD PE=1 SV=2 - [RHOD_HUMAN]	4,76	1	0,760
O15315-5	Isoform 5 of DNA repair protein RAD51 homolog 2 OS=Homo sapiens GN=RAD51B - [RA51B_HUMAN]	4,76	1	
P35680-3	Isoform C of Hepatocyte nuclear factor 1-beta OS=Homo sapiens GN=HNF1B - [HNF1B_HUMAN]	4,76	1	
P49354	Protein farnesyltransferase/geranylgeranyltransferase type-1 subunit alpha OS=Homo sapiens GN=FNTA PE=1 SV=1 - [FNTA_HUMAN]	4,75	1	0,736
Q53H96	Pyrroline-5-carboxylate reductase 3 OS=Homo sapiens GN=PYCRL PE=1 SV=2 - [P5CR3_HUMAN]	4,74	1	0,763
Q01664	Transcription factor AP-4 OS=Homo sapiens GN=TFAP4 PE=1 SV=2 - [TFAP4_HUMAN]	4,73	1	
O95630	STAM-binding protein OS=Homo sapiens GN=STAMBP PE=1 SV=1 - [STABP_HUMAN]	4,72	1	0,844
Q13043	Serine/threonine-protein kinase 4 OS=Homo sapiens GN=STK4 PE=1 SV=2 - [STK4_HUMAN]	4,72	1	
O43581	Synaptotagmin-7 OS=Homo sapiens GN=SYT7 PE=1 SV=3 - [SYT7_HUMAN]	4,71	1	0,847
O15194	CTD small phosphatase-like protein OS=Homo sapiens GN=CTDSPL PE=1 SV=2 - [CTDSL_HUMAN]	4,71	1	
P56559	ADP-ribosylation factor-like protein 4C OS=Homo sapiens GN=ARL4C PE=1 SV=1 - [ARL4C_HUMAN]	4,69	1	0,976
Q8IVJ1	Solute carrier family 41 member 1 OS=Homo sapiens GN=SLC41A1 PE=2 SV=2 - [S41A1_HUMAN]	4,68	1	1,033
Q9C004	Protein sprouty homolog 4 OS=Homo sapiens GN=SPRY4 PE=1 SV=2 - [SPY4_HUMAN]	4,68	1	
P05187	Alkaline phosphatase, placental type OS=Homo sapiens GN=ALPP PE=1 SV=2 - [PPB1_HUMAN]	4,67	1	0,369
G3V3G9	DDB1- and CUL4-associated factor 8 OS=Homo sapiens GN=DCAF8 PE=4 SV=1 - [G3V3G9_HUMAN]	4,66	1	
Q14116	Interleukin-18 OS=Homo sapiens GN=IL18 PE=1 SV=1 - [IL18_HUMAN]	4,66	1	
C9JRZ8	Aldo-keto reductase family 1 member B15 OS=Homo sapiens GN=AKR1B15 PE=2 SV=1 - [AK1BF_HUMAN]	4,65	1	
O95197	Reticulon-3 OS=Homo sapiens GN=RTN3 PE=1 SV=2 - [RTN3_HUMAN]	4,65	1	0,862
Q9NPG8	Probable palmitoyltransferase ZDHHC4 OS=Homo sapiens GN=ZDHHC4 PE=1 SV=1 - [ZDHC4_HUMAN]	4,65	1	0,839
Q16342	Programmed cell death protein 2 OS=Homo sapiens GN=PDCD2 PE=1 SV=2 - [PDCD2_HUMAN]	4,65	1	
Q9BXJ7	Protein amnionless OS=Homo sapiens GN=AMN PE=1 SV=2 - [AMNLS_HUMAN]	4,64	1	1,318
O75528	Transcriptional adapter 3 OS=Homo sapiens GN=TADA3 PE=1 SV=1 - [TADA3_HUMAN]	4,63	1	
Q9Y2C4	Nuclease EXOG, mitochondrial OS=Homo sapiens GN=EXOG PE=1 SV=2 - [EXOG_HUMAN]	4,62	1	1,632
Q9Y2D2	UDP-N-acetylglucosamine transporter OS=Homo sapiens GN=SLC35A3 PE=2 SV=1 - [S35A3_HUMAN]	4,62	1	1,541
Q96DW6	Solute carrier family 25 member 38 OS=Homo sapiens GN=SLC25A38 PE=1 SV=1 - [S2538_HUMAN]	4,61	1	0,753
Q5JS37	NHL repeat-containing protein 3 OS=Homo sapiens GN=NHLRC3 PE=2 SV=1 - [NHLC3_HUMAN]	4,61	1	0,728
J3QQL9	tRNA-dihydrouridine(16/17) synthase [NAD(P)(+)]-like (Fragment) OS=Homo sapiens GN=DUS1L PE=4 SV=1 - [J3QQL9_HUMAN]	4,61	1	
Q7L5D6	Golgi to ER traffic protein 4 homolog OS=Homo sapiens GN=GET4 PE=1 SV=1 - [GET4_HUMAN]	4,59	1	
Q86XZ4	Spermatogenesis-associated serine-rich protein 2 OS=Homo sapiens GN=SPATS2 PE=1 SV=1 - [SPAS2_HUMAN]	4,59	1	
P51589	Cytochrome P450 2J2 OS=Homo sapiens GN=CYP2J2 PE=1 SV=2 - [CP2J2_HUMAN]	4,58	1	0,490
Q8NBK3-2	Isoform 2 of Sulfatase-modifying factor 1 OS=Homo sapiens GN=SUMF1 - [SUMF1_HUMAN]	4,58	1	1,040
P50750	Cyclin-dependent kinase 9 OS=Homo sapiens GN=CDK9 PE=1 SV=3 - [CDK9_HUMAN]	4,57	1	
O94768	Serine/threonine-protein kinase 17B OS=Homo sapiens GN=STK17B PE=1 SV=1 - [ST17B_HUMAN]	4,57	1	
H3BUA6	2-oxoglutarate and iron-dependent oxygenase domain-containing protein 1 (Fragment) OS=Homo sapiens GN=OGFOD1 PE=4 SV=1 - [H3	4,57	1	
Q9NZR1	Tropomodulin-2 OS=Homo sapiens GN=TMOD2 PE=1 SV=1 - [TMOD2_HUMAN]	4,56	1	**4,018**
Q9H4G4	Golgi-associated plant pathogenesis-related protein 1 OS=Homo sapiens GN=GLIPR2 PE=1 SV=3 - [GAPR1_HUMAN]	4,55	1	2,156
Q96BZ8	Leukocyte receptor cluster member 1 OS=Homo sapiens GN=LENG1 PE=1 SV=1 - [LENG1_HUMAN]	4,55	1	
Q7L592	Protein midA homolog, mitochondrial OS=Homo sapiens GN=C2orf56 PE=1 SV=1 - [MIDA_HUMAN]	4,54	1	0,617
Q8NHP1	Aflatoxin B1 aldehyde reductase member 4 OS=Homo sapiens GN=AKR7L PE=2 SV=6 - [ARK74_HUMAN]	4,53	1	0,488
B7Z4R5	P2X purinoceptor OS=Homo sapiens GN=P2RX4 PE=2 SV=1 - [B7Z4R5_HUMAN]	4,53	1	
Q9HB07	UPF0160 protein MYG1, mitochondrial OS=Homo sapiens GN=C12orf10 PE=1 SV=2 - [MYG1_HUMAN]	4,52	1	
O75554	WW domain-binding protein 4 OS=Homo sapiens GN=WBP4 PE=1 SV=1 - [WBP4_HUMAN]	4,52	1	
P48060	Glioma pathogenesis-related protein 1 OS=Homo sapiens GN=GLIPR1 PE=1 SV=3 - [GLIP1_HUMAN]	4,51	1	1,552
Q969X1	Protein lifeguard 3 OS=Homo sapiens GN=TMBIM1 PE=1 SV=2 - [LFG3_HUMAN]	4,50	1	1,062
O94763	Unconventional prefoldin RPB5 interactor 1 OS=Homo sapiens GN=URI1 PE=1 SV=3 - [RMP_HUMAN]	4,49	1	
Q92905	COP9 signalosome complex subunit 5 OS=Homo sapiens GN=COPS5 PE=1 SV=4 - [CSN5_HUMAN]	4,49	1	
Q96I15	Selenocysteine lyase OS=Homo sapiens GN=SCLY PE=1 SV=2 - [SCLY_HUMAN]	4,49	1	
Q15036	Sorting nexin-17 OS=Homo sapiens GN=SNX17 PE=1 SV=1 - [SNX17_HUMAN]	4,47	1	1,241
Q8TCT0	Ceramide kinase OS=Homo sapiens GN=CERK PE=1 SV=1 - [CERK1_HUMAN]	4,47	1	
E7EUC4	SNF-related serine/threonine-protein kinase OS=Homo sapiens GN=SNRK PE=4 SV=1 - [E7EUC4_HUMAN]	4,47	1	
P30408	Transmembrane 4 L6 family member 1 OS=Homo sapiens GN=TM4SF1 PE=1 SV=1 - [T4S1_HUMAN]	4,46	1	**2,158**
P10588	Nuclear receptor subfamily 2 group F member 6 OS=Homo sapiens GN=NR2F6 PE=1 SV=2 - [NR2F6_HUMAN]	4,46	1	0,706
C9J9D8	Aminoacylase-1 OS=Homo sapiens GN=ACY1 PE=4 SV=1 - [C9J9D8_HUMAN]	4,46	1	
B4DEM1	CMP-sialic acid transporter OS=Homo sapiens GN=SLC35A1 PE=2 SV=1 - [B4DEM1_HUMAN]	4,43	1	1,144
Q8N567	Zinc finger CCHC domain-containing protein 9 OS=Homo sapiens GN=ZCCHC9 PE=2 SV=2 - [ZCHC9_HUMAN]	4,43	1	
P00846	ATP synthase subunit a OS=Homo sapiens GN=MT-ATP6 PE=1 SV=1 - [ATP6_HUMAN]	4,42	1	
C9JSF2	Heat shock factor 2-binding protein (Fragment) OS=Homo sapiens GN=HSF2BP PE=4 SV=1 - [C9JSF2_HUMAN]	4,41	1	1,366
Q9NX46	Poly(ADP-ribose) glycohydrolase ARH3 OS=Homo sapiens GN=ADPRHL2 PE=1 SV=1 - [ARHL2_HUMAN]	4,41	1	
O75391	Sperm-associated antigen 7 OS=Homo sapiens GN=SPAG7 PE=1 SV=2 - [SPAG7_HUMAN]	4,41	1	
Q6NTF9	Rhomboid domain-containing protein 2 OS=Homo sapiens GN=RHBDD2 PE=2 SV=2 - [RHBD2_HUMAN]	4,40	1	0,511
O14521	Succinate dehydrogenase [ubiquinone] cytochrome b small subunit, mitochondrial OS=Homo sapiens GN=SDHD PE=1 SV=1 - [DHSD_HUI	4,40	1	1,101
O14787-2	Isoform 2 of Transportin-2 OS=Homo sapiens GN=TNPO2 - [TNPO2_HUMAN]	4,40	1	
Q9BQ51	Programmed cell death 1 ligand 2 OS=Homo sapiens GN=PDCD1LG2 PE=1 SV=2 - [PD1L2_HUMAN]	4,40	1	
Q9BTE1	Dynactin subunit 5 OS=Homo sapiens GN=DCTN5 PE=1 SV=1 - [DCTN5_HUMAN]	4,40	1	
Q01151	CD83 antigen OS=Homo sapiens GN=CD83 PE=1 SV=1 - [CD83_HUMAN]	4,39	1	0,942
C9JQY5	RING finger protein 121 OS=Homo sapiens GN=RNF121 PE=4 SV=1 - [C9JQY5_HUMAN]	4,39	1	1,044
Q8N488	RING1 and YY1-binding protein OS=Homo sapiens GN=RYBP PE=1 SV=2 - [RYBP_HUMAN]	4,39	1	
Q01167	Forkhead box protein K2 OS=Homo sapiens GN=FOXK2 PE=1 SV=3 - [FOXK2_HUMAN]	4,39	1	
Q14186	Transcription factor Dp-1 OS=Homo sapiens GN=TFDP1 PE=1 SV=1 - [TFDP1_HUMAN]	4,39	1	
H7C1K1	Alanyl-tRNA-editing protein Aarsd1 (Fragment) OS=Homo sapiens GN=AARSD1 PE=4 SV=1 - [H7C1K1_HUMAN]	4,38	1	
H0YBN1	Lysosomal-associated transmembrane protein 4B (Fragment) OS=Homo sapiens GN=LAPTM4B PE=4 SV=1 - [H0YBN1_HUMAN]	4,37	1	
Q8NE22	Uncharacterized protein C5orf35 OS=Homo sapiens GN=C5orf35 PE=2 SV=2 - [CE035_HUMAN]	4,35	1	1,811
Q9BX74	TM2 domain-containing protein 1 OS=Homo sapiens GN=TM2D1 PE=1 SV=1 - [TM2D1_HUMAN]	4,35	1	1,348
Q5MIZ7-5	Isoform 5 of Serine/threonine-protein phosphatase 4 regulatory subunit 3B OS=Homo sapiens GN=SMEK2 - [P4R3B_HUMAN]	4,35	1	
Q9NV31	U3 small nucleolar ribonucleoprotein protein IMP3 OS=Homo sapiens GN=IMP3 PE=1 SV=1 - [IMP3_HUMAN]	4,35	1	
Q9H305	LITAF-like protein OS=Homo sapiens GN=C16orf5 PE=2 SV=1 - [LITFL_HUMAN]	4,33	1	**3,349**
Q9BXY0	Protein MAK16 homolog OS=Homo sapiens GN=MAK16 PE=1 SV=2 - [MAK16_HUMAN]	4,33	1	
Q15172	Serine/threonine-protein phosphatase 2A 56 kDa regulatory subunit alpha isoform OS=Homo sapiens GN=PPP2R5A PE=1 SV=1 - [2A5A_I	4,32	1	
Q5JRG1	Nucleoporin like 1 (Fragment) OS=Homo sapiens GN=NUPL1 PE=2 SV=1 - [Q5JRG1_HUMAN]	4,32	1	0,678
Q8IYJ2	Uncharacterized protein C10orf67 OS=Homo sapiens GN=C10orf67 PE=2 SV=2 - [CJ067_HUMAN]	4,32	1	
C9JLQ8	Adipocyte enhancer-binding protein 1 (Fragment) OS=Homo sapiens GN=AEBP1 PE=4 SV=1 - [C9JLQ8_HUMAN]	4,32	1	
G3V1B8	Sjoegren syndrome/scleroderma autoantigen 1 OS=Homo sapiens GN=SSSCA1 PE=4 SV=1 - [G3V1B8_HUMAN]	4,32	1	
Q9UKZ1	UPF0760 protein C2orf29 OS=Homo sapiens GN=C2orf29 PE=1 SV=1 - [CB029_HUMAN]	4,31	1	0,703
Q5VVQ6	Ubiquitin thioesterase OTU1 OS=Homo sapiens GN=YOD1 PE=1 SV=1 - [OTU1_HUMAN]	4,31	1	0,857
C9JMH6	Alpha-2-antiplasmin (Fragment) OS=Homo sapiens GN=SERPINF2 PE=3 SV=1 - [C9JMH6_HUMAN]	4,31	1	
P23610	Factor VIII intron 22 protein OS=Homo sapiens GN=F8A1 PE=1 SV=2 - [F8I2_HUMAN]	4,31	1	
Q9NZD2	Glycolipid transfer protein OS=Homo sapiens GN=GLTP PE=1 SV=3 - [GLTP_HUMAN]	4,31	1	
O75907	Diacylglycerol O-acyltransferase 1 OS=Homo sapiens GN=DGAT1 PE=1 SV=2 - [DGAT1_HUMAN]	4,30	1	1,249
Q8TDX7	Serine/threonine-protein kinase Nek7 OS=Homo sapiens GN=NEK7 PE=1 SV=1 - [NEK7_HUMAN]	4,30	1	
Q9BU89	Deoxyhypusine hydroxylase OS=Homo sapiens GN=DOHH PE=1 SV=1 - [DOHH_HUMAN]	4,30	1	
Q12887	Protoheme IX farnesyltransferase, mitochondrial OS=Homo sapiens GN=COX10 PE=1 SV=3 - [COX10_HUMAN]	4,29	1	1,442
Q9BX68	Histidine triad nucleotide-binding protein 2, mitochondrial OS=Homo sapiens GN=HINT2 PE=1 SV=1 - [HINT2_HUMAN]	4,29	1	
P30086	Phosphatidylethanolamine-binding protein 1 OS=Homo sapiens GN=PEBP1 PE=1 SV=3 - [PEBP1_HUMAN]	4,28	1	0,827
Q9Y256	CAAX prenyl protease 2 OS=Homo sapiens GN=RCE1 PE=1 SV=1 - [FACE2_HUMAN]	4,26	1	0,803
P0C5W0	Paraneoplastic antigen-like protein 6B OS=Homo sapiens GN=PNMA6B PE=2 SV=1 - [PNM6B_HUMAN]	4,26	1	
Q8WUA8	Tsukushin OS=Homo sapiens GN=TSKU PE=2 SV=3 - [TSK_HUMAN]	4,25	1	
Q96GC5	39S ribosomal protein L48, mitochondrial OS=Homo sapiens GN=MRPL48 PE=1 SV=2 - [RM48_HUMAN]	4,25	1	
Q8N5I4	Dehydrogenase/reductase SDR family member on chromosome X OS=Homo sapiens GN=DHRSX PE=2 SV=2 - [DHRSX_HUMAN]	4,24	1	1,157
Q86SK9	Stearoyl-CoA desaturase 5 OS=Homo sapiens GN=SCD5 PE=2 SV=2 - [SCD5_HUMAN]	4,24	1	0,538
Q96RS6-3	Isoform 3 of NudC domain-containing protein 1 OS=Homo sapiens GN=NUDCD1 - [NUDC1_HUMAN]	4,23	1	
P05412	Transcription factor AP-1 OS=Homo sapiens GN=JUN PE=1 SV=2 - [JUN_HUMAN]	4,23	1	
Q9NXN4-2	Isoform 2 of Ganglioside-induced differentiation-associated protein 2 OS=Homo sapiens GN=GDAP2 - [GDAP2_HUMAN]	4,23	1	
J3KT41	SRC kinase-signaling inhibitor 1 (Fragment) OS=Homo sapiens GN=SRCIN1 PE=4 SV=1 - [J3KT41_HUMAN]	4,22	1	0,696
P24588	A-kinase anchor protein 5 OS=Homo sapiens GN=AKAP5 PE=1 SV=3 - [AKAP5_HUMAN]	4,22	1	
P19075	Tetraspanin-8 OS=Homo sapiens GN=TSPAN8 PE=1 SV=1 - [TSN8_HUMAN]	4,22	1	
Q676U5-4	Isoform 4 of Autophagy-related protein 16-1 OS=Homo sapiens GN=ATG16L1 - [A16L1_HUMAN]	4,21	1	0,801
Q7L3T8	Probable proline--tRNA ligase, mitochondrial OS=Homo sapiens GN=PARS2 PE=1 SV=1 - [SYPM_HUMAN]	4,21	1	
Q9H8M1	Coenzyme Q-binding protein COQ10 homolog B, mitochondrial OS=Homo sapiens GN=COQ10B PE=2 SV=1 - [CQ10B_HUMAN]	4,20	1	0,962
Q8NEY4-2	Isoform 2 of V-type proton ATPase subunit C 2 OS=Homo sapiens GN=ATP6V1C2 - [VATC2_HUMAN]	4,20	1	1,149
Q96DF8	Protein DGCR14 OS=Homo sapiens GN=DGCR14 PE=1 SV=1 - [DGC14_HUMAN]	4,20	1	
B4DS50	Dol-P-Man:Man(5)GlcNAc(2)-PP-Dol alpha-1,3-mannosyltransferase OS=Homo sapiens GN=ALG3 PE=2 SV=1 - [B4DS50_HUMAN]	4,19	1	
Q96EI5	Transcription elongation factor A protein-like 4 OS=Homo sapiens GN=TCEAL4 PE=1 SV=2 - [TCAL4_HUMAN]	4,19	1	
Q06587	E3 ubiquitin-protein ligase RING1 OS=Homo sapiens GN=RING1 PE=1 SV=2 - [RING1_HUMAN]	4,19	1	
Q12840	Kinesin heavy chain isoform 5A OS=Homo sapiens GN=KIF5A PE=1 SV=2 - [KIF5A_HUMAN]	4,17	1	
O14732	Inositol monophosphatase 2 OS=Homo sapiens GN=IMPA2 PE=1 SV=1 - [IMPA2_HUMAN]	4,17	1	1,087
Q15642	Cdc42-interacting protein 4 OS=Homo sapiens GN=TRIP10 PE=1 SV=3 - [CIP4_HUMAN]	4,16	1	1,406
O60518	Ran-binding protein 6 OS=Homo sapiens GN=RANBP6 PE=1 SV=2 - [RNBP6_HUMAN]	4,16	1	0,490
Q8IVD9	NudC domain-containing protein 3 OS=Homo sapiens GN=NUDCD3 PE=1 SV=3 - [NUDC3_HUMAN]	4,16	1	
Q7L523	Ras-related GTP-binding protein A OS=Homo sapiens GN=RRAGA PE=1 SV=1 - [RRAGA_HUMAN]	4,15	1	1,441
Q9Y6A4	UPF0468 protein C16orf80 OS=Homo sapiens GN=C16orf80 PE=1 SV=1 - [CP080_HUMAN]	4,15	1	
Q96BH1	E3 ubiquitin-protein ligase RNF25 OS=Homo sapiens GN=RNF25 PE=1 SV=1 - [RNF25_HUMAN]	4,14	1	0,606
Q9NZ53	Podocalyxin-like protein 2 OS=Homo sapiens GN=PODXL2 PE=1 SV=1 - [PDXL2_HUMAN]	4,13	1	
Q96A46	Mitoferrin-2 OS=Homo sapiens GN=SLC25A28 PE=2 SV=1 - [MFRN2_HUMAN]	4,12	1	1,082
F8VS03	Cyclic AMP-dependent transcription factor ATF-1 (Fragment) OS=Homo sapiens GN=ATF1 PE=4 SV=1 - [F8VS03_HUMAN]	4,12	1	
Q8NHU3	Phosphatidylcholine:ceramide cholinephosphotransferase 2 OS=Homo sapiens GN=SGMS2 PE=1 SV=1 - [SMS2_HUMAN]	4,11	1	0,840
Q03001-3	Isoform 3 of Dystonin OS=Homo sapiens GN=DST - [DYST_HUMAN]	4,11	1	
P28065	Proteasome subunit beta type-9 OS=Homo sapiens GN=PSMB9 PE=1 SV=2 - [PSB9_HUMAN]	4,11	1	
Q15633	RISC-loading complex subunit TARBP2 OS=Homo sapiens GN=TARBP2 PE=1 SV=3 - [TRBP2_HUMAN]	4,10	1	
Q96G25	Mediator of RNA polymerase II transcription subunit 8 OS=Homo sapiens GN=MED8 PE=1 SV=2 - [MED8_HUMAN]	4,10	1	
O75665-2	Isoform 2 of Oral-facial-digital syndrome 1 protein OS=Homo sapiens GN=OFD1 - [OFD1_HUMAN]	4,09	1	
Q96QD9	UAP56-interacting factor OS=Homo sapiens GN=FYTTD1 PE=1 SV=3 - [UIF_HUMAN]	4,09	1	
P02766	Transthyretin OS=Homo sapiens GN=TTR PE=1 SV=1 - [TTHY_HUMAN]	4,08	1	1,593
P04438	Ig heavy chain V-II region SESS OS=Homo sapiens PE=2 SV=1 - [HV208_HUMAN]	4,08	1	1,258
Q16651	Prostasin OS=Homo sapiens GN=PRSS8 PE=1 SV=1 - [PRSS8_HUMAN]	4,08	1	0,107
Q9NPD3	Exosome complex component RRP41 OS=Homo sapiens GN=EXOSC4 PE=1 SV=3 - [EXOS4_HUMAN]	4,08	1	1,345
Q13057	Bifunctional coenzyme A synthase OS=Homo sapiens GN=COASY PE=1 SV=4 - [COASY_HUMAN]	4,08	1	1,187
Q9BXC0	Hydroxycarboxylic acid receptor 1 OS=Homo sapiens GN=HCAR1 PE=1 SV=1 - [HCAR1_HUMAN]	4,05	1	1,230
Q96CP7	TLC domain-containing protein 1 OS=Homo sapiens GN=TLCD1 PE=2 SV=1 - [TLCD1_HUMAN]	4,05	1	1,054
O00291	Huntingtin-interacting protein 1 OS=Homo sapiens GN=HIP1 PE=1 SV=5 - [HIP1_HUMAN]	4,05	1	
Q6A1A2	Putative 3-phosphoinositide-dependent protein kinase 2 OS=Homo sapiens GN=PDPK2 PE=5 SV=1 - [PDPK2_HUMAN]	4,04	1	
Q96KB5	Lymphokine-activated killer T-cell-originated protein kinase OS=Homo sapiens GN=PBK PE=1 SV=3 - [TOPK_HUMAN]	4,04	1	
Q9Y388	RNA-binding motif protein, X-linked 2 OS=Homo sapiens GN=RBMX2 PE=1 SV=2 - [RBMX2_HUMAN]	4,04	1	
Q9H8W4	Pleckstrin homology domain-containing family F member 2 OS=Homo sapiens GN=PLEKHF2 PE=1 SV=1 - [PKHF2_HUMAN]	4,02	1	
Q5JVF3	PCI domain-containing protein 2 OS=Homo sapiens GN=PCID2 PE=1 SV=2 - [PCID2_HUMAN]	4,01	1	0,662
Q9BQT8	Mitochondrial 2-oxodicarboxylate carrier OS=Homo sapiens GN=SLC25A21 PE=1 SV=1 - [ODC_HUMAN]	4,01	1	0,757
B4DDT2	Xylulose kinase OS=Homo sapiens GN=XYLB PE=2 SV=1 - [B4DDT2_HUMAN]	4,01	1	
Q96A54	Adiponectin receptor protein 1 OS=Homo sapiens GN=ADIPOR1 PE=1 SV=1 - [ADR1_HUMAN]	4,00	1	1,512
Q9BVX2	Transmembrane protein 106C OS=Homo sapiens GN=TMEM106C PE=2 SV=1 - [T106C_HUMAN]	4,00	1	1,298
Q08334	Interleukin-10 receptor subunit beta OS=Homo sapiens GN=IL10RB PE=1 SV=2 - [I10R2_HUMAN]	4,00	1	
Q96N38-3	Isoform 3 of Zinc finger protein 714 OS=Homo sapiens GN=ZNF714 - [ZN714_HUMAN]	4,00	1	
O00459	Phosphatidylinositol 3-kinase regulatory subunit beta OS=Homo sapiens GN=PIK3R2 PE=1 SV=2 - [P85B_HUMAN]	3,98	1	1,066
Q8N4Q0	Zinc-binding alcohol dehydrogenase domain-containing protein 2 OS=Homo sapiens GN=ZADH2 PE=1 SV=1 - [ZADH2_HUMAN]	3,98	1	0,983
Q9H3P2	Negative elongation factor A OS=Homo sapiens GN=WHSC2 PE=1 SV=3 - [NELFA_HUMAN]	3,98	1	
O14965	Aurora kinase A OS=Homo sapiens GN=AURKA PE=1 SV=2 - [AURKA_HUMAN]	3,97	1	
Q96BW5-2	Isoform 2 of Phosphotriesterase-related protein OS=Homo sapiens GN=PTER - [PTER_HUMAN]	3,97	1	
P41247	Patatin-like phospholipase domain-containing protein 4 OS=Homo sapiens GN=PNPLA4 PE=1 SV=3 - [PLPL4_HUMAN]	3,95	1	
B4E024	Zinc finger CCHC domain-containing protein 7 OS=Homo sapiens GN=ZCCHC7 PE=2 SV=1 - [B4E024_HUMAN]	3,95	1	
Q96GC9	Vacuole membrane protein 1 OS=Homo sapiens GN=VMP1 PE=1 SV=1 - [VMP1_HUMAN]	3,94	1	1,088
Q9HAN9	Nicotinamide mononucleotide adenylyltransferase 1 OS=Homo sapiens GN=NMNAT1 PE=1 SV=1 - [NMNA1_HUMAN]	3,94	1	
Q96HR3	Mediator of RNA polymerase II transcription subunit 30 OS=Homo sapiens GN=MED30 PE=1 SV=1 - [MED30_HUMAN]	3,93	1	
E7ESZ5	Calcium-binding mitochondrial carrier protein SCaMC-3 OS=Homo sapiens GN=SLC25A23 PE=3 SV=1 - [E7ESZ5_HUMAN]	3,93	1	
Q6IQ49-3	Isoform 3 of Protein SDE2 homolog OS=Homo sapiens GN=SDE2 - [SDE2_HUMAN]	3,93	1	

P50897	Palmitoyl-protein thioesterase 1 OS=Homo sapiens GN=PPT1 PE=1 SV=1 - [PPT1_HUMAN]	3,92	1	0,889	0,767				1,025	1,018
Q8TB96	T-cell immunomodulatory protein OS=Homo sapiens GN=ITFG1 PE=1 SV=1 - [TIP_HUMAN]	3,92	1		0,881					0,984
G5E9E2	DNA-3-methyladenine glycosylase OS=Homo sapiens GN=MPG PE=3 SV=1 - [G5E9E2_HUMAN]	3,91	1	0,988					1,145	
B4DGA7	Kyphoscoliosis peptidase OS=Homo sapiens GN=KY PE=2 SV=1 - [B4DGA7_HUMAN]	3,90	1	1,096					1,150	
Q96EZ8	Microspherule protein 1 OS=Homo sapiens GN=MCRS1 PE=1 SV=1 - [MCRS1_HUMAN]	3,90	1			0,910				
E7EWY2	DNA topoisomerase OS=Homo sapiens GN=TOP3B PE=3 SV=1 - [E7EWY2_HUMAN]	3,90	1			1,040				
F5H0V4	MLX-interacting protein (Fragment) OS=Homo sapiens GN=MLXIP PE=4 SV=1 - [F5H0V4_HUMAN]	3,90	1					0,967		
Q9NQG6	Mitochondrial dynamic protein MID51 OS=Homo sapiens GN=SMCR7L PE=1 SV=1 - [MID51_HUMAN]	3,89	1	0,539					0,773	
Q9BQC3	Diphthamide biosynthesis protein 2 OS=Homo sapiens GN=DPH2 PE=1 SV=1 - [DPH2_HUMAN]	3,89	1		1,105			1,430		1,821
O14879	Interferon-induced protein with tetratricopeptide repeats 3 OS=Homo sapiens GN=IFIT3 PE=1 SV=1 - [IFIT3_HUMAN]	3,88	1		**0,318**					0,329
Q9H6Z4	Ran-binding protein 3 OS=Homo sapiens GN=RANBP3 PE=1 SV=1 - [RANB3_HUMAN]	3,88	1			0,884	0,910			
P0CAP2-3	Isoform 3 of DNA-directed RNA polymerase II subunit GRINL1A OS=Homo sapiens GN=POLR2M - [GRL1A_HUMAN]	3,86	1		0,762					0,892
Q13153	Serine/threonine-protein kinase PAK 1 OS=Homo sapiens GN=PAK1 PE=1 SV=2 - [PAK1_HUMAN]	3,85	1	0,762					0,883	
Q8IZ73	RNA pseudouridylate synthase domain-containing protein 2 OS=Homo sapiens GN=RPUSD2 PE=1 SV=2 - [RUSD2_HUMAN]	3,85	1			0,890				
Q9H2P9-2	Isoform 2 of Diphthine synthase OS=Homo sapiens GN=DPH5 - [DPH5_HUMAN]	3,85	1					1,182		
Q9H3M7	Thioredoxin-interacting protein OS=Homo sapiens GN=TXNIP PE=1 SV=1 - [TXNIP_HUMAN]	3,84	1	0,258					0,255	
E9PIC5	Choline transporter-like protein 3 OS=Homo sapiens GN=SLC44A3 PE=4 SV=1 - [E9PIC5_HUMAN]	3,84	1					0,680		
Q96BY9	Transmembrane protein 66 OS=Homo sapiens GN=TMEM66 PE=1 SV=1 - [TMM66_HUMAN]	3,83	1	1,212	1,062				0,999	0,873
Q8IVH4	Methylmalonic aciduria type A protein, mitochondrial OS=Homo sapiens GN=MMAA PE=1 SV=1 - [MMAA_HUMAN]	3,83	1	0,769					0,670	
Q9NQT4	Exosome complex component RRP46 OS=Homo sapiens GN=EXOSC5 PE=1 SV=1 - [EXOS5_HUMAN]	3,83	1				0,912			
Q9UNQ0	ATP-binding cassette sub-family G member 2 OS=Homo sapiens GN=ABCG2 PE=1 SV=3 - [ABCG2_HUMAN]	3,82	1		1,074					0,952
B7Z1L4	Serine protease hepsin OS=Homo sapiens GN=HPN PE=2 SV=1 - [B7Z1L4_HUMAN]	3,82	1		0,800					0,909
Q8NC67	Neuropilin and tolloid-like protein 2 OS=Homo sapiens GN=NETO2 PE=1 SV=1 - [NETO2_HUMAN]	3,81	1		1,167					1,378
P49642	DNA primase small subunit OS=Homo sapiens GN=PRIM1 PE=1 SV=1 - [PRI1_HUMAN]	3,81	1				0,836			
Q92597	Protein NDRG1 OS=Homo sapiens GN=NDRG1 PE=1 SV=1 - [NDRG1_HUMAN]	3,81	1			1,138				
Q9NZ63	Uncharacterized protein C9orf78 OS=Homo sapiens GN=C9orf78 PE=1 SV=1 - [CI078_HUMAN]	3,81	1					1,038		
Q5VZI3	Transmembrane protein C9orf91 OS=Homo sapiens GN=C9orf91 PE=2 SV=1 - [CI091_HUMAN]	3,80	1	1,019					1,242	
Q9BWQ6	Protein YIPF2 OS=Homo sapiens GN=YIPF2 PE=2 SV=1 - [YIPF2_HUMAN]	3,80	1	0,743					0,868	
A8MY84	Angiopoietin-related protein 4 OS=Homo sapiens GN=ANGPTL4 PE=4 SV=1 - [A8MY84_HUMAN]	3,80	1		1,436					0,856
E5RGH7	Heparan-alpha-glucosaminide N-acetyltransferase (Fragment) OS=Homo sapiens GN=HGSNAT PE=4 SV=1 - [E5RGH7_HUMAN]	3,80	1				0,913			
Q9BXJ8	Transmembrane protein 120A OS=Homo sapiens GN=TMEM120A PE=2 SV=1 - [T120A_HUMAN]	3,79	1	1,004					0,788	
P17097	Zinc finger protein 7 OS=Homo sapiens GN=ZNF7 PE=1 SV=1 - [ZNF7_HUMAN]	3,79	1					1,645		
Q9P0W2	SWI/SNF-related matrix-associated actin-dependent regulator of chromatin subfamily E member 1-related OS=Homo sapiens GN=HMG2	3,79	1			1,192				
Q9NUL5	UPF0515 protein C19orf66 OS=Homo sapiens GN=C19orf66 PE=2 SV=2 - [CS066_HUMAN]	3,78	1					1,039		
Q5VZ72	Izumo sperm-egg fusion protein 3 OS=Homo sapiens GN=IZUMO3 PE=2 SV=4 - [IZUM3_HUMAN]	3,77	1				0,765			
Q8NFI3	Cytosolic endo-beta-N-acetylglucosaminidase OS=Homo sapiens GN=ENGASE PE=1 SV=1 - [ENASE_HUMAN]	3,77	1					0,682		
Q9NVD7	Alpha-parvin OS=Homo sapiens GN=PARVA PE=1 SV=1 - [PARVA_HUMAN]	3,76	1	0,887				0,980	1,020	
Q14790	Caspase-8 OS=Homo sapiens GN=CASP8 PE=1 SV=1 - [CASP8_HUMAN]	3,76	1		0,692					0,808
O76080	AN1-type zinc finger protein 5 OS=Homo sapiens GN=ZFAND5 PE=1 SV=1 - [ZFAN5_HUMAN]	3,76	1				0,917			
Q8TB03	Uncharacterized protein CXorf38 OS=Homo sapiens GN=CXorf38 PE=1 SV=1 - [CX038_HUMAN]	3,76	1				0,987			
Q5TDH0	Protein DDI1 homolog 2 OS=Homo sapiens GN=DDI2 PE=1 SV=1 - [DDI2_HUMAN]	3,76	1					0,848		
Q9UGY1	Nucleolar protein 12 OS=Homo sapiens GN=NOL12 PE=1 SV=1 - [NOL12_HUMAN]	3,76	1					1,070		
Q6UWU4	Bombesin receptor-activated protein C6orf89 OS=Homo sapiens GN=C6orf89 PE=1 SV=1 - [CF089_HUMAN]	3,75	1	0,740	0,836				0,808	0,942
Q02833	Ras association domain-containing protein 7 OS=Homo sapiens GN=RASSF7 PE=2 SV=1 - [RASF7_HUMAN]	3,75	1		0,996					1,029
Q9BV79	Trans-2-enoyl-CoA reductase, mitochondrial OS=Homo sapiens GN=MECR PE=1 SV=2 - [MECR_HUMAN]	3,75	1		0,544			0,987		0,554
H3BTA2	Serine/threonine-protein phosphatase (Fragment) OS=Homo sapiens GN=PPP4C PE=3 SV=1 - [H3BTA2_HUMAN]	3,75	1					0,838		
Q3MIP1	Inositol 1,4,5-trisphosphate receptor-interacting protein-like 2 OS=Homo sapiens GN=ITPRIPL2 PE=2 SV=1 - [IPIL2_HUMAN]	3,74	1	1,147					1,310	
B9A064	Immunoglobulin lambda-like polypeptide 5 OS=Homo sapiens GN=IGLL5 PE=2 SV=2 - [IGLL5_HUMAN]	3,74	1	0,967					0,928	
Q13630	GDP-L-fucose synthase OS=Homo sapiens GN=TSTA3 PE=1 SV=1 - [FCL_HUMAN]	3,74	1				0,456	0,760		
Q9UL40	Zinc finger protein 346 OS=Homo sapiens GN=ZNF346 PE=1 SV=1 - [ZN346_HUMAN]	3,74	1				1,050	0,997		
Q9P031	Thyroid transcription factor 1-associated protein 26 OS=Homo sapiens GN=CCDC59 PE=1 SV=2 - [TAP26_HUMAN]	3,73	1				0,925			
Q9HBE1-2	Isoform 2 of POZ-, AT hook-, and zinc finger-containing protein 1 OS=Homo sapiens GN=PATZ1 - [PATZ1_HUMAN]	3,72	1			0,790				
P41231	P2Y purinoceptor 2 OS=Homo sapiens GN=P2RY2 PE=1 SV=4 - [P2RY2_HUMAN]	3,71	1							
Q6ZVX9	Progestin and adipoQ receptor family member 9 OS=Homo sapiens GN=PAQR9 PE=2 SV=1 - [PAQR9_HUMAN]	3,71	1		1,485	1,196				1,477
H0Y2S9	Myosin phosphatase Rho-interacting protein OS=Homo sapiens GN=MPRIP PE=4 SV=2 - [H0Y2S9_HUMAN]	3,71	1				0,976			
C9JRJ5	LIM domain-containing protein 1 OS=Homo sapiens GN=LIMD1 PE=4 SV=1 - [C9JRJ5_HUMAN]	3,71	1				0,962			
Q9NWU1	3-oxoacyl-[acyl-carrier-protein] synthase, mitochondrial OS=Homo sapiens GN=OXSM PE=1 SV=1 - [OXSM_HUMAN]	3,70	1				1,043	1,245		
Q9H446	RWD domain-containing protein 1 OS=Homo sapiens GN=RWDD1 PE=1 SV=1 - [RWDD1_HUMAN]	3,70	1					0,864		
Q6PL24	Protein TMED8 OS=Homo sapiens GN=TMED8 PE=1 SV=1 - [TMED8_HUMAN]	3,69	1	0,688		0,875		0,965	0,849	
Q9BRQ0	Pygopus homolog 2 OS=Homo sapiens GN=PYGO2 PE=1 SV=2 - [PYGO2_HUMAN]	3,69	1			0,930	0,813			
Q3KQZ1	Solute carrier family 25 member 35 OS=Homo sapiens GN=SLC25A35 PE=2 SV=1 - [S2535_HUMAN]	3,67	1		1,392					1,280
Q969E8	Pre-rRNA-processing protein TSR2 homolog OS=Homo sapiens GN=TSR2 PE=1 SV=1 - [TSR2_HUMAN]	3,66	1	1,004			1,059		0,847	
Q8NCA5	Protein FAM98A OS=Homo sapiens GN=FAM98A PE=1 SV=1 - [FA98A_HUMAN]	3,66	1			0,820	0,798	0,930		
Q96GA7	Serine dehydratase-like OS=Homo sapiens GN=SDSL PE=1 SV=1 - [SDSL_HUMAN]	3,65	1		0,647					0,902
D6R992	Transcription factor RelB OS=Homo sapiens GN=RELB PE=4 SV=1 - [D6R992_HUMAN]	3,65	1							
Q9Y314	Nitric oxide synthase-interacting protein OS=Homo sapiens GN=NOSIP PE=1 SV=1 - [NOSIP_HUMAN]	3,65	1				1,445			
E9PG83	Perilipin-2 OS=Homo sapiens GN=PLIN2 PE=4 SV=1 - [E9PG83_HUMAN]	3,65	1					1,249		
Q9NXX6	Non-structural maintenance of chromosomes element 4 homolog A OS=Homo sapiens GN=NSMCE4A PE=1 SV=2 - [NSE4A_HUMAN]	3,64	1			0,929				
Q7Z412	Peroxisome assembly protein 26 OS=Homo sapiens GN=PEX26 PE=1 SV=2 - [PEX26_HUMAN]	3,61	1	1,571					1,386	
Q0VAA5	PI-PLC X domain-containing protein 2 OS=Homo sapiens GN=PLCXD2 PE=1 SV=1 - [PLCX2_HUMAN]	3,61	1				1,007			
P08263	Glutathione S-transferase A1 OS=Homo sapiens GN=GSTA1 PE=1 SV=3 - [GSTA1_HUMAN]	3,60	1				3,801			
Q86WB0	Nuclear-interacting partner of ALK OS=Homo sapiens GN=ZC3HC1 PE=1 SV=1 - [NIPA_HUMAN]	3,59	1					1,428		
H7C2Z7	Mucin-16 OS=Homo sapiens GN=MUC16 PE=4 SV=1 - [H7C2Z7_HUMAN]	3,58	1		0,429					0,446
Q6E0U4	Dermokine OS=Homo sapiens GN=DMKN PE=1 SV=3 - [DMKN_HUMAN]	3,57	1	0,931					1,357	
Q9NXF8	Palmitoyltransferase ZDHHC7 OS=Homo sapiens GN=ZDHHC7 PE=2 SV=2 - [ZDHC7_HUMAN]	3,57	1		1,375					1,206
A6NFU8	Pyroglutamyl-peptidase 1-like protein OS=Homo sapiens GN=PGPEP1L PE=2 SV=4 - [PGPIL_HUMAN]	3,57	1				1,108			
Q99611	Selenide, water dikinase 2 OS=Homo sapiens GN=SEPHS2 PE=1 SV=3 - [SPS2_HUMAN]	3,57	1				0,766			
Q9UPY8	Microtubule-associated protein RP/EB family member 3 OS=Homo sapiens GN=MAPRE3 PE=1 SV=1 - [MARE3_HUMAN]	3,56	1	1,005					1,112	
Q5SR56	Hippocampus abundant transcript-like protein 1 OS=Homo sapiens GN=HIATL1 PE=2 SV=3 - [HIAL1_HUMAN]	3,56	1		1,292					1,096
Q9BSU1	UPF0183 protein C16orf70 OS=Homo sapiens GN=C16orf70 PE=1 SV=1 - [CP070_HUMAN]	3,55	1				0,940	1,027		
Q53S58	Transmembrane protein 177 OS=Homo sapiens GN=TMEM177 PE=2 SV=1 - [TM177_HUMAN]	3,54	1	1,236	1,180				0,967	0,909
Q7L5Y9	Macrophage erythroblast attacher OS=Homo sapiens GN=MAEA PE=1 SV=1 - [MAEA_HUMAN]	3,54	1			1,114				
Q99543	DnaJ homolog subfamily C member 2 OS=Homo sapiens GN=DNAJC2 PE=1 SV=4 - [DNJC2_HUMAN]	3,54	1					1,390		
Q13825	Methylglutaconyl-CoA hydratase, mitochondrial OS=Homo sapiens GN=AUH PE=1 SV=1 - [AUHM_HUMAN]	3,54	1					1,072		
Q9BYM8	RanBP-type and C3HC4-type zinc finger-containing protein 1 OS=Homo sapiens GN=RBCK1 PE=1 SV=2 - [HOIL1_HUMAN]	3,53	1			0,711				
Q8IXQ4	Uncharacterized protein KIAA1704 OS=Homo sapiens GN=KIAA1704 PE=1 SV=1 - [K1704_HUMAN]	3,53	1				0,867	1,108		
B4DDV3	NAD-dependent protein deacetylase sirtuin-6 OS=Homo sapiens GN=SIRT6 PE=2 SV=1 - [B4DDV3_HUMAN]	3,53	1							
O14494	Lipid phosphate phosphohydrolase 1 OS=Homo sapiens GN=PPAP2A PE=1 SV=1 - [LPP1_HUMAN]	3,52	1	1,686	1,334				1,244	1,077
Q8WUT9	Solute carrier family 25 member 43 OS=Homo sapiens GN=SLC25A43 PE=2 SV=2 - [S2543_HUMAN]	3,52	1	0,738					0,717	
P33908	Mannosyl-oligosaccharide 1,2-alpha-mannosidase IA OS=Homo sapiens GN=MAN1A1 PE=1 SV=3 - [MA1A1_HUMAN]	3,52	1		1,281					1,345
Q2TAY7	WD40 repeat-containing protein SMU1 OS=Homo sapiens GN=SMU1 PE=1 SV=2 - [SMU1_HUMAN]	3,51	1		0,651		0,874			0,967
Q969M3	Protein YIPF5 OS=Homo sapiens GN=YIPF5 PE=1 SV=1 - [YIPF5_HUMAN]	3,50	1	1,035	1,067				0,737	0,689
Q5VSG8	Glycoprotein endo-alpha-1,2-mannosidase-like protein OS=Homo sapiens GN=MANEAL PE=2 SV=1 - [MANEL_HUMAN]	3,50	1	1,008					0,950	
O75323	Protein NipSnap homolog 2 OS=Homo sapiens GN=GBAS PE=1 SV=1 - [NIPS2_HUMAN]	3,50	1	0,331	0,355		0,906	0,889	0,482	0,530
Q6UW56	All-trans retinoic acid-induced differentiation factor OS=Homo sapiens GN=ATRAID PE=1 SV=2 - [ARAID_HUMAN]	3,49	1	1,063	1,128				0,803	1,062
Q9H8J5	MANSC domain-containing protein 1 OS=Homo sapiens GN=MANSC1 PE=2 SV=1 - [MANS1_HUMAN]	3,48	1	0,520					0,588	
Q05048	Cleavage stimulation factor subunit 1 OS=Homo sapiens GN=CSTF1 PE=1 SV=1 - [CSTF1_HUMAN]	3,48	1		1,117		0,904			1,294
Q9Y608	Leucine-rich repeat flightless-interacting protein 2 OS=Homo sapiens GN=LRRFIP2 PE=1 SV=1 - [LRRF2_HUMAN]	3,47	1					1,042		
Q9NXR1	Nuclear distribution protein nudE homolog 1 OS=Homo sapiens GN=NDE1 PE=1 SV=2 - [NDE1_HUMAN]	3,47	1			1,302				
Q9NYZ3	G2 and S phase-expressed protein 1 OS=Homo sapiens GN=GTSE1 PE=1 SV=3 - [GTSE1_HUMAN]	3,47	1			0,733				
Q8TC26	Transmembrane protein 163 OS=Homo sapiens GN=TMEM163 PE=2 SV=1 - [TM163_HUMAN]	3,46	1	**4,685**	**2,004**				1,627	1,207
Q08495	Dematin OS=Homo sapiens GN=EPB49 PE=1 SV=3 - [DEMA_HUMAN]	3,46	1			1,238				
Q9UMX1-2	Isoform 2 of Suppressor of fused homolog OS=Homo sapiens GN=SUFU - [SUFU_HUMAN]	3,46	1				0,842			
Q96BR5	Sel1 repeat-containing protein 1 OS=Homo sapiens GN=SELRC1 PE=1 SV=2 - [SELR1_HUMAN]	3,46	1				1,065			
H0Y6R4	Rap guanine nucleotide exchange factor 1 (Fragment) OS=Homo sapiens GN=RAPGEF1 PE=4 SV=1 - [H0Y6R4_HUMAN]	3,45	1	0,836	0,962				0,573	0,815
O60240	Perilipin-1 OS=Homo sapiens GN=PLIN1 PE=1 SV=2 - [PLIN1_HUMAN]	3,45	1			0,501	0,864			
Q9H8M5	Metal transporter CNNM2 OS=Homo sapiens GN=CNNM2 PE=1 SV=2 - [CNNM2_HUMAN]	3,43	1		1,032					1,037
Q9NS91	E3 ubiquitin-protein ligase RAD18 OS=Homo sapiens GN=RAD18 PE=1 SV=2 - [RAD18_HUMAN]	3,43	1			1,235				
Q5TGI0	Uncharacterized protein C6orf168 OS=Homo sapiens GN=C6orf168 PE=2 SV=2 - [CF168_HUMAN]	3,42	1	1,757					1,177	
Q9H2V7	Protein spinster homolog 1 OS=Homo sapiens GN=SPNS1 PE=1 SV=1 - [SPNS1_HUMAN]	3,41	1	1,141	1,343				1,016	0,998
Q8NB16	Mixed lineage kinase domain-like protein OS=Homo sapiens GN=MLKL PE=1 SV=1 - [MLKL_HUMAN]	3,40	1		0,397			0,425		0,624
Q6DD87	Zinc finger protein 787 OS=Homo sapiens GN=ZNF787 PE=1 SV=2 - [ZN787_HUMAN]	3,40	1				1,131	1,147		
Q6P444	Protein FAM54A OS=Homo sapiens GN=FAM54A PE=2 SV=2 - [FA54A_HUMAN]	3,38	1	0,687	0,644				0,842	0,938
Q12824	SWI/SNF-related matrix-associated actin-dependent regulator of chromatin subfamily B member 1 OS=Homo sapiens GN=SMARCB1 PE=	3,38	1				0,818			
P53004	Biliverdin reductase A OS=Homo sapiens GN=BLVRA PE=1 SV=2 - [BIEA_HUMAN]	3,38	1					1,228		
Q9UKU7	Isobutyryl-CoA dehydrogenase, mitochondrial OS=Homo sapiens GN=ACAD8 PE=1 SV=1 - [ACAD8_HUMAN]	3,37	1		0,911					0,971
F8W9W8	Solute carrier organic anion transporter family member 2A1 OS=Homo sapiens GN=SLCO2A1 PE=4 SV=1 - [F8W9W8_HUMAN]	3,37	1		0,792					0,750
P15336	Cyclic AMP-dependent transcription factor ATF-2 OS=Homo sapiens GN=ATF2 PE=1 SV=4 - [ATF2_HUMAN]	3,37	1			1,122	1,028			
Q14249	Endonuclease G, mitochondrial OS=Homo sapiens GN=ENDOG PE=1 SV=4 - [NUCG_HUMAN]	3,37	1				1,024			
Q96FK6	WD repeat-containing protein 89 OS=Homo sapiens GN=WDR89 PE=1 SV=1 - [WDR89_HUMAN]	3,36	1				0,791	0,865		
O76081	Regulator of G-protein signaling 20 OS=Homo sapiens GN=RGS20 PE=1 SV=4 - [RGS20_HUMAN]	3,35	1	1,325					1,121	
Q9H2J4	Phosducin-like protein 3 OS=Homo sapiens GN=PDCL3 PE=1 SV=1 - [PDCL3_HUMAN]	3,35	1				0,906			
Q9Y5K5	Ubiquitin carboxyl-terminal hydrolase isozyme L5 OS=Homo sapiens GN=UCHL5 PE=1 SV=3 - [UCHL5_HUMAN]	3,34	1	1,101					0,983	
P41240	Tyrosine-protein kinase CSK OS=Homo sapiens GN=CSK PE=1 SV=1 - [CSK_HUMAN]	3,33	1		0,660					0,773
Q9NUN5	Probable lysosomal cobalamin transporter OS=Homo sapiens GN=LMBRD1 PE=1 SV=1 - [LMBD1_HUMAN]	3,33	1		1,623					1,071
Q8WXG1	Radical S-adenosyl methionine domain-containing protein 2 OS=Homo sapiens GN=RSAD2 PE=1 SV=1 - [RSAD2_HUMAN]	3,32	1	0,380					0,352	
Q8N6G5	Chondroitin sulfate N-acetylgalactosaminyltransferase 2 OS=Homo sapiens GN=CSGALNACT2 PE=2 SV=1 - [CGAT2_HUMAN]	3,32	1		1,117					1,375
Q5PRF9	Protein Smaug homolog 2 OS=Homo sapiens GN=SAMD4B PE=1 SV=1 - [SMAG2_HUMAN]	3,31	1			0,915				
Q9UJJ9	N-acetylglucosamine-1-phosphotransferase subunit gamma OS=Homo sapiens GN=GNPTG PE=1 SV=1 - [GNPTG_HUMAN]	3,28	1	0,878	0,972				0,835	0,784
Q9H0E2	Toll-interacting protein OS=Homo sapiens GN=TOLLIP PE=1 SV=1 - [TOLIP_HUMAN]	3,28	1		0,959		0,934			0,991
Q9BX73	TM2 domain-containing protein 2 OS=Homo sapiens GN=TM2D2 PE=2 SV=1 - [TM2D2_HUMAN]	3,27	1	2,152					0,954	
Q5T2R2-2	Isoform 2 of Decaprenyl-diphosphate synthase subunit 1 OS=Homo sapiens GN=PDSS1 - [DPS1_HUMAN]	3,27	1		0,662					0,651
O75794	Cell division cycle protein 123 homolog OS=Homo sapiens GN=CDC123 PE=1 SV=1 - [CD123_HUMAN]	3,27	1				1,053			
O94903	Proline synthase co-transcribed bacterial homolog protein OS=Homo sapiens GN=PROSC PE=1 SV=1 - [PROSC_HUMAN]	3,27	1					0,815		
Q9Y3I1	F-box only protein 7 OS=Homo sapiens GN=FBXO7 PE=1 SV=1 - [FBX7_HUMAN]	3,26	1				1,459	1,284		
Q9Y2S7	Polymerase delta-interacting protein 2 OS=Homo sapiens GN=POLDIP2 PE=1 SV=1 - [PDIP2_HUMAN]	3,26	1					0,890		
B7ZKK9	PPP2R5E protein OS=Homo sapiens GN=PPP2R5E PE=2 SV=1 - [B7ZKK9_HUMAN]	3,25	1	0,484					0,599	
Q92747	Actin-related protein 2/3 complex subunit 1A OS=Homo sapiens GN=ARPC1A PE=1 SV=2 - [ARC1A_HUMAN]	3,24	1	0,622	0,744				0,682	0,786
Q8TBC4	NEDD8-activating enzyme E1 catalytic subunit OS=Homo sapiens GN=UBA3 PE=1 SV=2 - [UBA3_HUMAN]	3,24	1	0,677	0,389			0,965	0,831	0,484
Q6P9H4	Connector enhancer of kinase suppressor of ras 3 OS=Homo sapiens GN=CNKSR3 PE=1 SV=1 - [CNKR3_HUMAN]	3,24	1	1,499					1,114	
Q96N21	Uncharacterized protein C17orf56 OS=Homo sapiens GN=C17orf56 PE=1 SV=1 - [CQ056_HUMAN]	3,24	1				0,968			
P78318	Immunoglobulin-binding protein 1 OS=Homo sapiens GN=IGBP1 PE=1 SV=1 - [IGBP1_HUMAN]	3,24	1					0,901		
Q86VR2	Protein FAM134C OS=Homo sapiens GN=FAM134C PE=1 SV=1 - [F134C_HUMAN]	3,22	1	1,237	1,071				1,225	0,925
Q9HAB8	Phosphopantothenate--cysteine ligase OS=Homo sapiens GN=PPCS PE=1 SV=2 - [PPCS_HUMAN]	3,22	1	0,765					0,883	
Q9H6T0-2	Isoform 2 of Epithelial splicing regulatory protein 2 OS=Homo sapiens GN=ESRP2 - [ESRP2_HUMAN]	3,21	1					0,929		
B4DWQ2	Activin receptor type-2A OS=Homo sapiens GN=ACVR2A PE=2 SV=1 - [B4DWQ2_HUMAN]	3,21	1		0,737					0,952
P32929	Cystathionine gamma-lyase OS=Homo sapiens GN=CTH PE=1 SV=3 - [CGL_HUMAN]	3,21	1				0,443			
Q13286	Battenin OS=Homo sapiens GN=CLN3 PE=1 SV=1 - [CLN3_HUMAN]	3,20	1	1,357					1,213	
Q53EL6	Programmed cell death protein 4 OS=Homo sapiens GN=PDCD4 PE=1 SV=2 - [PDCD4_HUMAN]	3,20	1					0,967		
Q8IWV2-2	Isoform 2 of Contactin-4 OS=Homo sapiens GN=CNTN4 - [CNTN4_HUMAN]	3,19	1			0,809				
Q5TGY6	Gardner-Rasheed feline sarcoma viral (V-fgr) oncogene homolog (Fragment) OS=Homo sapiens GN=FGR PE=2 SV=1 - [Q5TGY6_HUMAN	3,19	1					0,804		
B4E0K5	Mitogen-activated protein kinase 14 OS=Homo sapiens GN=MAPK14 PE=2 SV=1 - [B4E0K5_HUMAN]	3,18	1	1,207					0,864	
A8MXL6	Protein SEC13 homolog OS=Homo sapiens GN=SEC13 PE=4 SV=1 - [A8MXL6_HUMAN]	3,17	1	0,656					0,733	
Q96ME7	Zinc finger protein 512 OS=Homo sapiens GN=ZNF512 PE=1 SV=2 - [ZN512_HUMAN]	3,17	1			1,113	1,166			
O43639	Cytoplasmic protein NCK2 OS=Homo sapiens GN=NCK2 PE=1 SV=2 - [NCK2_HUMAN]	3,16	1			1,353				
Q96BT7	Alkylated DNA repair protein alkB homolog 8 OS=Homo sapiens GN=ALKBH8 PE=1 SV=2 - [ALKB8_HUMAN]	3,16	1			0,874				
Q92560	Ubiquitin carboxyl-terminal hydrolase BAP1 OS=Homo sapiens GN=BAP1 PE=1 SV=2 - [BAP1_HUMAN]	3,16	1			0,905	1,087			
Q96BW9	Mitochondrial translocator assembly and maintenance protein 41 homolog OS=Homo sapiens GN=TAMM41 PE=2 SV=1 - [TAM41_HUMA	3,16	1					0,927		
Q9H8P0	Probable polyprenol reductase OS=Homo sapiens GN=SRD5A3 PE=1 SV=1 - [PORED_HUMAN]	3,14	1	1,099	1,196				0,948	1,140
Q6UW49	Sperm equatorial segment protein 1 OS=Homo sapiens GN=SPESP1 PE=1 SV=2 - [SPESP_HUMAN]	3,14	1	0,665					0,640	
Q5VV23	Chromosome 10 open reading frame 63 OS=Homo sapiens GN=C10orf63 PE=2 SV=1 - [Q5VV23_HUMAN]	3,14	1	**2,680**					3,363	
P35813	Protein phosphatase 1A OS=Homo sapiens GN=PPM1A PE=1 SV=1 - [PPM1A_HUMAN]	3,14	1			0,943	0,955			
Q00688	Peptidyl-prolyl cis-trans isomerase FKBP3 OS=Homo sapiens GN=FKBP3 PE=1 SV=1 - [FKBP3_HUMAN]	3,13	1				0,776			
Q5T2D2	Trem-like transcript 2 protein OS=Homo sapiens GN=TREML2 PE=1 SV=2 - [TRML2_HUMAN]	3,12	1	0,633					0,695	
Q86V24	Adiponectin receptor protein 2 OS=Homo sapiens GN=ADIPOR2 PE=1 SV=1 - [ADR2_HUMAN]	3,11	1	0,814	1,015				1,031	1,106
P31483	Nucleolysin TIA-1 isoform p40 OS=Homo sapiens GN=TIA1 PE=1 SV=3 - [TIA1_HUMAN]	3,11	1					0,783		
O14929	Histone acetyltransferase type B catalytic subunit OS=Homo sapiens GN=HAT1 PE=1 SV=1 - [HAT1_HUMAN]	3,10	1					0,685		
P08833	Insulin-like growth factor-binding protein 1 OS=Homo sapiens GN=IGFBP1 PE=1 SV=1 - [IBP1_HUMAN]	3,09	1	1,217					1,110	
Q8N999	Uncharacterized protein C12orf29 OS=Homo sapiens GN=C12orf29 PE=1 SV=2 - [CL029_HUMAN]	3,08	1		0,761		0,987	0,934		0,829
Q86SY2	DDB1- and CUL4-associated factor 4 OS=Homo sapiens GN=DCAF4 PE=2 SV=1 - [Q86SY2_HUMAN]	3,08	1			0,939				

Q9BQ69	MACRO domain-containing protein 1 OS=Homo sapiens GN=MACROD1 PE=1 SV=2 - [MACD1_HUMAN]	3,08	1		
Q9BVC4-3	Isoform 2 of Target of rapamycin complex subunit LST8 OS=Homo sapiens GN=MLST8 - [LST8_HUMAN]	3,08	1		
Q8N5Y2	Male-specific lethal 3 homolog OS=Homo sapiens GN=MSL3 PE=1 SV=1 - [MS3L1_HUMAN]	3,07	1		
Q8N0Y2-2	Isoform 2 of Zinc finger protein 444 OS=Homo sapiens GN=ZNF444 - [ZN444_HUMAN]	3,07	1		
J3QRA1	Processed fractalkine OS=Homo sapiens GN=CX3CL1 PE=4 SV=1 - [J3QRA1_HUMAN]	3,06	1	1,262	
Q9BZJ4	Solute carrier family 25 member 39 OS=Homo sapiens GN=SLC25A39 PE=2 SV=2 - [S2539_HUMAN]	3,06	1		1,341
E7ETP9	Lysosome-associated membrane glycoprotein 3 OS=Homo sapiens GN=LAMP3 PE=4 SV=1 - [E7ETP9_HUMAN]	3,06	1		0,743
Q9NXE8	Pre-mRNA-splicing factor CWC25 homolog OS=Homo sapiens GN=CWC25 PE=1 SV=1 - [CWC25_HUMAN]	3,06	1		
Q5JU67-2	Isoform 2 of Uncharacterized protein C9orf117 OS=Homo sapiens GN=C9orf117 - [CI117_HUMAN]	3,06	1		
Q96NT5	Proton-coupled folate transporter OS=Homo sapiens GN=SLC46A1 PE=1 SV=1 - [PCFT_HUMAN]	3,05	1	0,946	
H0YA47	RUN and FYVE domain-containing protein 1 (Fragment) OS=Homo sapiens GN=RUFY1 PE=4 SV=1 - [H0YA47_HUMAN]	3,05	1		
Q02742	Beta-1,3-galactosyl-O-glycosyl-glycoprotein beta-1,6-N-acetylglucosaminyltransferase OS=Homo sapiens GN=GCNT1 PE=2 SV=2 - [GCN	3,04	1	1,334	
O43683	Mitotic checkpoint serine/threonine-protein kinase BUB1 OS=Homo sapiens GN=BUB1 PE=1 SV=1 - [BUB1_HUMAN]	3,04	1		
O60941	Dystrobrevin beta OS=Homo sapiens GN=DTNB PE=1 SV=1 - [DTNB_HUMAN]	3,03	1		1,087
Q9HD26	Golgi-associated PDZ and coiled-coil motif-containing protein OS=Homo sapiens GN=GOPC PE=1 SV=1 - [GOPC_HUMAN]	3,03	1		
Q9UJA5	tRNA (adenine(58)-N(1))-methyltransferase non-catalytic subunit TRM6 OS=Homo sapiens GN=TRMT6 PE=1 SV=1 - [TRM6_HUMAN]	3,02	1		
O14656	Torsin-1A OS=Homo sapiens GN=TOR1A PE=1 SV=1 - [TOR1A_HUMAN]	3,01	1		1,509
Q8WTR4	Glycerophosphodiester phosphodiesterase domain-containing protein 5 OS=Homo sapiens GN=GDPD5 PE=2 SV=2 - [GDPD5_HUMAN]	2,98	1	1,516	
P22794	Protein EVI2A OS=Homo sapiens GN=EVI2A PE=2 SV=2 - [EVI2A_HUMAN]	2,97	1	**2,827**	
Q9NVA4	Transmembrane protein 184C OS=Homo sapiens GN=TMEM184C PE=2 SV=2 - [T184C_HUMAN]	2,97	1	1,400	
Q15035	Translocating chain-associated membrane protein 2 OS=Homo sapiens GN=TRAM2 PE=1 SV=1 - [TRAM2_HUMAN]	2,97	1		0,959
Q5T4D3	Transmembrane and TPR repeat-containing protein 4 OS=Homo sapiens GN=TMTC4 PE=1 SV=2 - [TMTC4_HUMAN]	2,97	1		1,459
Q8TBC3	SH3KBP1-binding protein 1 OS=Homo sapiens GN=SHKBP1 PE=1 SV=2 - [SHKB1_HUMAN]	2,97	1		
P15927	Replication protein A 32 kDa subunit OS=Homo sapiens GN=RPA2 PE=1 SV=1 - [RFA2_HUMAN]	2,96	1		
Q96IR7	4-hydroxyphenylpyruvate dioxygenase-like protein OS=Homo sapiens GN=HPDL PE=1 SV=1 - [HPDL_HUMAN]	2,96	1		
Q96SZ5	2-aminoethanethiol dioxygenase OS=Homo sapiens GN=ADO PE=1 SV=2 - [AEDO_HUMAN]	2,96	1		
Q0JRZ9	FCH domain only protein 2 OS=Homo sapiens GN=FCHO2 PE=1 SV=2 - [FCHO2_HUMAN]	2,96	1		
P43250	G protein-coupled receptor kinase 6 OS=Homo sapiens GN=GRK6 PE=1 SV=2 - [GRK6_HUMAN]	2,95	1	0,782	
P14921	Protein C-ets-1 OS=Homo sapiens GN=ETS1 PE=1 SV=1 - [ETS1_HUMAN]	2,95	1	0,756	0,791
Q9BZD2	Equilibrative nucleoside transporter 3 OS=Homo sapiens GN=SLC29A3 PE=1 SV=3 - [S29A3_HUMAN]	2,95	1	1,216	1,442
F5H012	E3 ubiquitin-protein ligase TRIM21 OS=Homo sapiens GN=TRIM21 PE=4 SV=1 - [F5H012_HUMAN]	2,95	1	0,780	
Q96GM8	Target of EGR1 protein 1 OS=Homo sapiens GN=TOE1 PE=1 SV=1 - [TOE1_HUMAN]	2,94	1	0,656	
Q5T0D9	Tumor protein p63-regulated gene 1-like protein OS=Homo sapiens GN=TPRG1L PE=1 SV=1 - [TPRGL_HUMAN]	2,94	1		0,805
F5H853	Differentially-expressed in FDCP 6 homolog OS=Homo sapiens GN=DEF6 PE=4 SV=1 - [F5H853_HUMAN]	2,93	1		
Q96F63	Coiled-coil domain-containing protein 97 OS=Homo sapiens GN=CCDC97 PE=1 SV=1 - [CCD97_HUMAN]	2,92	1		
B1AL69	Cell division cycle 37 homolog (S. cerevisiae)-like 1 OS=Homo sapiens GN=CDC37L1 PE=4 SV=1 - [B1AL69_HUMAN]	2,92	1		
Q15276	Rab GTPase-binding effector protein 1 OS=Homo sapiens GN=RABEP1 PE=1 SV=2 - [RABE1_HUMAN]	2,90	1	1,155	
P23409	Myogenic factor 6 OS=Homo sapiens GN=MYF6 PE=1 SV=1 - [MYF6_HUMAN]	2,89	1	1,402	
O43422	52 kDa repressor of the inhibitor of the protein kinase OS=Homo sapiens GN=PRKRIR PE=2 SV=2 - [P52K_HUMAN]	2,89	1		
B7Z8K9	Tyrosine-protein phosphatase non-receptor type 3 OS=Homo sapiens GN=PTPN3 PE=2 SV=1 - [B7Z8K9_HUMAN]	2,89	1	1,532	
Q96LJ7	Dehydrogenase/reductase SDR family member 1 OS=Homo sapiens GN=DHRS1 PE=1 SV=1 - [DHRS1_HUMAN]	2,88	1	1,087	
Q8IW45	ATP-dependent (S)-NAD(P)H-hydrate dehydratase OS=Homo sapiens GN=CARKD PE=1 SV=1 - [NNRD_HUMAN]	2,88	1		1,126
Q9NQH7-3	Isoform 3 of Probable Xaa-Pro aminopeptidase 3 OS=Homo sapiens GN=XPNPEP3 - [XPP3_HUMAN]	2,88	1		0,832
Q14376	UDP-glucose 4-epimerase OS=Homo sapiens GN=GALE PE=1 SV=2 - [GALE_HUMAN]	2,87	1		0,807
Q15532	Protein SSXT OS=Homo sapiens GN=SS18 PE=1 SV=3 - [SSXT_HUMAN]	2,87	1		
Q15545	Transcription initiation factor TFIID subunit 7 OS=Homo sapiens GN=TAF7 PE=1 SV=1 - [TAF7_HUMAN]	2,87	1		
Q6YP21	Kynurenine--oxoglutarate transaminase 3 OS=Homo sapiens GN=CCBL2 PE=1 SV=1 - [KAT3_HUMAN]	2,86	1	0,839	0,782
Q92990	Glomulin OS=Homo sapiens GN=GLMN PE=1 SV=2 - [GLMN_HUMAN]	2,86	1		0,416
Q96FC7-2	Isoform 2 of Phytanoyl-CoA hydroxylase-interacting protein-like OS=Homo sapiens GN=PHYHIPL - [PHIPL_HUMAN]	2,86	1		
F2Z2I2	6-phosphofructo-2-kinase OS=Homo sapiens GN=PFKFB3 PE=4 SV=1 - [F2Z2I2_HUMAN]	2,86	1		
P17032	Zinc finger protein 37A OS=Homo sapiens GN=ZNF37A PE=2 SV=3 - [ZN37A_HUMAN]	2,85	1		0,337
Q8NAF0	Zinc finger protein 579 OS=Homo sapiens GN=ZNF579 PE=1 SV=2 - [ZN579_HUMAN]	2,85	1		
Q96BI1	Solute carrier family 22 member 18 OS=Homo sapiens GN=SLC22A18 PE=1 SV=3 - [S22AI_HUMAN]	2,83	1	0,419	0,481
Q8NDP7	Limb region 1 protein homolog OS=Homo sapiens GN=DKFZp547F222 PE=2 SV=1 - [Q8NDP7_HUMAN]	2,82	1	1,418	
Q6ZSR9	Uncharacterized protein FLJ45252 OS=Homo sapiens PE=1 SV=2 - [YJ005_HUMAN]	2,82	1		
Q86YZ3	Hornerin OS=Homo sapiens GN=HRNR PE=1 SV=2 - [HORN_HUMAN]	2,81	1	1,867	
P28702	Retinoic acid receptor RXR-beta OS=Homo sapiens GN=RXRB PE=1 SV=2 - [RXRB_HUMAN]	2,81	1		
Q96S55-2	Isoform 2 of ATPase WRNIP1 OS=Homo sapiens GN=WRNIP1 - [WRIP1_HUMAN]	2,81	1		
P53384	Cytosolic Fe-S cluster assembly factor NUBP1 OS=Homo sapiens GN=NUBP1 PE=1 SV=2 - [NUBP1_HUMAN]	2,81	1		
P49760	Dual specificity protein kinase CLK2 OS=Homo sapiens GN=CLK2 PE=1 SV=1 - [CLK2_HUMAN]	2,81	1		
Q00577	Transcriptional activator protein Pur-alpha OS=Homo sapiens GN=PURA PE=1 SV=2 - [PURA_HUMAN]	2,80	1		
Q96GS4	Uncharacterized protein C17orf59 OS=Homo sapiens GN=C17orf59 PE=1 SV=2 - [CQ059_HUMAN]	2,80	1		
Q9ULX6	A-kinase anchor protein 8-like OS=Homo sapiens GN=AKAP8L PE=1 SV=3 - [AKP8L_HUMAN]	2,79	1		
Q96HH9	GRAM domain-containing protein 3 OS=Homo sapiens GN=GRAMD3 PE=1 SV=1 - [GRAM3_HUMAN]	2,78	1	0,774	
Q9Y5P6	Mannose-1-phosphate guanyltransferase beta OS=Homo sapiens GN=GMPPB PE=1 SV=2 - [GMPPB_HUMAN]	2,78	1		0,859
Q8N0Z9	V-set and immunoglobulin domain-containing protein 10 OS=Homo sapiens GN=VSIG10 PE=2 SV=1 - [VSI10_HUMAN]	2,78	1		0,808
Q9UKF6	Cleavage and polyadenylation specificity factor subunit 3 OS=Homo sapiens GN=CPSF3 PE=1 SV=1 - [CPSF3_HUMAN]	2,78	1		
B4E1Q4	Serine/threonine-protein kinase RIO3 OS=Homo sapiens GN=RIOK3 PE=2 SV=1 - [B4E1Q4_HUMAN]	2,78	1		
Q6KCM7	Calcium-binding mitochondrial carrier protein SCaMC-2 OS=Homo sapiens GN=SLC25A25 PE=1 SV=1 - [SCMC2_HUMAN]	2,77	1	0,963	0,885
H3BTB7	Probable glutamate--tRNA ligase, mitochondrial OS=Homo sapiens GN=EARS2 PE=3 SV=1 - [H3BTB7_HUMAN]	2,77	1		0,689
Q9H7Y2	Uncharacterized protein FLJ14100 OS=Homo sapiens PE=1 SV=1 - [YA001_HUMAN]	2,76	1	1,586	
Q7L4I2	Arginine/serine-rich coiled-coil protein 2 OS=Homo sapiens GN=RSRC2 PE=1 SV=1 - [RSRC2_HUMAN]	2,76	1		
Q92791	Synaptonemal complex protein SC65 OS=Homo sapiens GN=LEPREL4 PE=1 SV=1 - [SC65_HUMAN]	2,75	1		
P49643	DNA primase large subunit OS=Homo sapiens GN=PRIM2 PE=1 SV=2 - [PRI2_HUMAN]	2,75	1		
P49137	MAP kinase-activated protein kinase 2 OS=Homo sapiens GN=MAPKAPK2 PE=1 SV=1 - [MAPK2_HUMAN]	2,75	1		
Q92685	Dol-P-Man:Man(5)GlcNAc(2)-PP-Dol alpha-1,3-mannosyltransferase OS=Homo sapiens GN=ALG3 PE=1 SV=1 - [ALG3_HUMAN]	2,74	1	2,097	1,159
P49406	39S ribosomal protein L19, mitochondrial OS=Homo sapiens GN=MRPL19 PE=1 SV=2 - [RM19_HUMAN]	2,74	1	0,733	0,712
Q14155	Rho guanine nucleotide exchange factor 7 OS=Homo sapiens GN=ARHGEF7 PE=1 SV=2 - [ARHG7_HUMAN]	2,74	1		
Q96MU6	Zinc finger protein 778 OS=Homo sapiens GN=ZNF778 PE=2 SV=3 - [ZN778_HUMAN]	2,74	1		
Q86VN1-2	Isoform 2 of Vacuolar protein-sorting-associated protein 36 OS=Homo sapiens GN=VPS36 - [VPS36_HUMAN]	2,74	1		
Q96ES7	SAGA-associated factor 29 homolog OS=Homo sapiens GN=CCDC101 PE=1 SV=1 - [SGF29_HUMAN]	2,73	1		
Q96B23	Uncharacterized protein C18orf25 OS=Homo sapiens GN=C18orf25 PE=1 SV=2 - [CR025_HUMAN]	2,73	1		
Q86TL2	Transmembrane protein 110 OS=Homo sapiens GN=TMEM110 PE=2 SV=1 - [TM110_HUMAN]	2,72	1	0,954	0,899
Q7RTX8	Putative HIN1-like protein OS=Homo sapiens GN=HIN1L PE=5 SV=1 - [HIN1L_HUMAN]	2,71	1	0,822	
Q96GW9	Methionine--tRNA ligase, mitochondrial OS=Homo sapiens GN=MARS2 PE=1 SV=2 - [SYMM_HUMAN]	2,70	1	0,724	0,431
Q8N8Q8	Mitochondrial inner membrane protein COX18 OS=Homo sapiens GN=COX18 PE=2 SV=1 - [COX18_HUMAN]	2,70	1		1,475
Q9UKL0	REST corepressor 1 OS=Homo sapiens GN=RCOR1 PE=1 SV=1 - [RCOR1_HUMAN]	2,70	1		
Q7Z404-1	Isoform 2 of Transmembrane channel-like protein 4 OS=Homo sapiens GN=TMC4 - [TMC4_HUMAN]	2,69	1	0,149	
Q8NB90	Spermatogenesis-associated protein 5 OS=Homo sapiens GN=SPATA5 PE=1 SV=3 - [SPAT5_HUMAN]	2,69	1		
P33897	ATP-binding cassette sub-family D member 1 OS=Homo sapiens GN=ABCD1 PE=1 SV=2 - [ABCD1_HUMAN]	2,68	1	0,872	0,350
Q5T4F4	Protrudin OS=Homo sapiens GN=ZFYVE27 PE=1 SV=1 - [ZFY27_HUMAN]	2,68	1		0,996
O95685	Protein phosphatase 1 regulatory subunit 3D OS=Homo sapiens GN=PPP1R3D PE=1 SV=1 - [PPR3D_HUMAN]	2,68	1		
Q9BYJ9	YTH domain family protein 1 OS=Homo sapiens GN=YTHDF1 PE=1 SV=1 - [YTHD1_HUMAN]	2,68	1		
Q9UEE9	Craniofacial development protein 1 OS=Homo sapiens GN=CFDP1 PE=1 SV=1 - [CFDP1_HUMAN]	2,68	1		
Q9UGV2	Protein NDRG3 OS=Homo sapiens GN=NDRG3 PE=1 SV=2 - [NDRG3_HUMAN]	2,67	1	1,149	1,397
Q9Y5T5	Ubiquitin carboxyl-terminal hydrolase 16 OS=Homo sapiens GN=USP16 PE=1 SV=1 - [UBP16_HUMAN]	2,67	1		
O15229	Kynurenine 3-monooxygenase OS=Homo sapiens GN=KMO PE=1 SV=2 - [KMO_HUMAN]	2,67	1	0,162	
Q9UJA2	Cardiolipin synthase OS=Homo sapiens GN=CRLS1 PE=2 SV=1 - [CRLS1_HUMAN]	2,66	1	1,334	1,290
P17538	Chymotrypsinogen B OS=Homo sapiens GN=CTRB1 PE=2 SV=1 - [CTRB1_HUMAN]	2,66	1	0,956	0,910
Q9Y5B8	Nucleoside diphosphate kinase 7 OS=Homo sapiens GN=NME7 PE=1 SV=1 - [NDK7_HUMAN]	2,66	1	0,823	
Q8IWE2	Protein NOXP20 OS=Homo sapiens GN=FAM114A1 PE=1 SV=2 - [NXP20_HUMAN]	2,66	1		1,078
Q93038	Tumor necrosis factor receptor superfamily member 25 OS=Homo sapiens GN=TNFRSF25 PE=1 SV=2 - [TNR25_HUMAN]	2,64	1	0,693	
Q13077	TNF receptor-associated factor 1 OS=Homo sapiens GN=TRAF1 PE=1 SV=1 - [TRAF1_HUMAN]	2,64	1	2,228	1,928
Q8N5A5	Zinc finger CCCH-type with G patch domain-containing protein OS=Homo sapiens GN=ZGPAT PE=1 SV=3 - [ZGPAT_HUMAN]	2,64	1		
Q14542	Equilibrative nucleoside transporter 2 OS=Homo sapiens GN=SLC29A2 PE=1 SV=3 - [S29A2_HUMAN]	2,63	1		1,703
Q9H9S5	Fukutin-related protein OS=Homo sapiens GN=FKRP PE=1 SV=1 - [FKRP_HUMAN]	2,63	1		1,076
Q9H6E5	Speckle targeted PIP5K1A-regulated poly(A) polymerase OS=Homo sapiens GN=TUT1 PE=1 SV=2 - [STPAP_HUMAN]	2,63	1		
Q15173	Serine/threonine-protein phosphatase 2A 56 kDa regulatory subunit beta isoform OS=Homo sapiens GN=PPP2R5B PE=1 SV=1 - [2A5B_H	2,62	1		
Q9UKG9	Peroxisomal carnitine O-octanoyltransferase OS=Homo sapiens GN=CROT PE=1 SV=2 - [OCTC_HUMAN]	2,61	1	1,610	
Q02086	Transcription factor Sp2 OS=Homo sapiens GN=SP2 PE=1 SV=3 - [SP2_HUMAN]	2,61	1		
Q8NC26-2	Isoform 2 of Zinc finger protein 114 OS=Homo sapiens GN=ZNF114 - [ZN114_HUMAN]	2,61	1		
Q8N8Z6-2	Isoform 2 of Discoidin, CUB and LCCL domain-containing protein 1 OS=Homo sapiens GN=DCBLD1 - [DCBD1_HUMAN]	2,60	1	0,942	
P03891	NADH-ubiquinone oxidoreductase chain 2 OS=Homo sapiens GN=MT-ND2 PE=1 SV=2 - [NU2M_HUMAN]	2,59	1	1,453	
Q9NVE7	Pantothenate kinase 4 OS=Homo sapiens GN=PANK4 PE=1 SV=1 - [PANK4_HUMAN]	2,59	1		
Q6IQ23-3	Isoform 3 of Pleckstrin homology domain-containing family A member 7 OS=Homo sapiens GN=PLEKHA7 - [PKHA7_HUMAN]	2,59	1		
Q15750	TGF-beta-activated kinase 1 and MAP3K7-binding protein 1 OS=Homo sapiens GN=TAB1 PE=1 SV=1 - [TAB1_HUMAN]	2,58	1	0,995	
O60687	Sushi repeat-containing protein SRPX2 OS=Homo sapiens GN=SRPX2 PE=1 SV=1 - [SRPX2_HUMAN]	2,58	1		0,720
Q16206-2	Isoform 2 of Ecto-NOX disulfide-thiol exchanger 2 OS=Homo sapiens GN=ENOX2 - [ENOX2_HUMAN]	2,58	1		
P29279	Connective tissue growth factor OS=Homo sapiens GN=CTGF PE=1 SV=2 - [CTGF_HUMAN]	2,58	1		
O95801	Tetratricopeptide repeat protein 4 OS=Homo sapiens GN=TTC4 PE=1 SV=3 - [TTC4_HUMAN]	2,58	1		
Q96S59-2	Isoform 2 of Ran-binding protein 9 OS=Homo sapiens GN=RANBP9 - [RANB9_HUMAN]	2,58	1		
Q96JB5	CDK5 regulatory subunit-associated protein 3 OS=Homo sapiens GN=CDK5RAP3 PE=1 SV=2 - [CK5P3_HUMAN]	2,57	1	0,930	
Q9H6Y7	E3 ubiquitin-protein ligase RNF167 OS=Homo sapiens GN=RNF167 PE=1 SV=1 - [RN167_HUMAN]	2,57	1	1,093	1,016
Q92796	Disks large homolog 3 OS=Homo sapiens GN=DLG3 PE=1 SV=2 - [DLG3_HUMAN]	2,57	1		1,075
Q8IXY8	Peptidyl-prolyl cis-trans isomerase-like 6 OS=Homo sapiens GN=PPIL6 PE=2 SV=1 - [PPIL6_HUMAN]	2,57	1		
P06280	Alpha-galactosidase A OS=Homo sapiens GN=GLA PE=1 SV=1 - [AGAL_HUMAN]	2,56	1	1,022	
P08651	Nuclear factor 1 C-type OS=Homo sapiens GN=NFIC PE=1 SV=2 - [NFIC_HUMAN]	2,56	1		
Q9H3F6	BTB/POZ domain-containing adapter for CUL3-mediated RhoA degradation protein 3 OS=Homo sapiens GN=KCTD10 PE=1 SV=1 - [BACI	2,56	1		
Q9H0M0-6	Isoform 6 of NEDD4-like E3 ubiquitin-protein ligase WWP1 OS=Homo sapiens GN=WWP1 - [WWP1_HUMAN]	2,56	1		
Q9Y2Z9-2	Isoform 2 of Ubiquinone biosynthesis monooxygenase COQ6 OS=Homo sapiens GN=COQ6 - [COQ6_HUMAN]	2,56	1		
P01876	Ig alpha-1 chain C region OS=Homo sapiens GN=IGHA1 PE=1 SV=2 - [IGHA1_HUMAN]	2,55	1		1,269
Q9UP83-3	Isoform 3 of Conserved oligomeric Golgi complex subunit 5 OS=Homo sapiens GN=COG5 - [COG5_HUMAN]	2,55	1		0,758
Q96JB2	Conserved oligomeric Golgi complex subunit 3 OS=Homo sapiens GN=COG3 PE=1 SV=3 - [COG3_HUMAN]	2,54	1	0,543	
O15455	Toll-like receptor 3 OS=Homo sapiens GN=TLR3 PE=1 SV=1 - [TLR3_HUMAN]	2,54	1		0,837
Q96FZ2	UPF0361 protein C3orf37 OS=Homo sapiens GN=C3orf37 PE=1 SV=1 - [CC037_HUMAN]	2,54	1		
H3BLU9	Tetratricopeptide repeat protein 24 (Fragment) OS=Homo sapiens GN=TTC24 PE=4 SV=1 - [H3BLU9_HUMAN]	2,54	1		
Q14088	Ras-related protein Rab-33A OS=Homo sapiens GN=RAB33A PE=1 SV=2 - [RB33A_HUMAN]	2,53	1		1,416
F5H804	Nuclear protein MDM1 OS=Homo sapiens GN=MDM1 PE=4 SV=1 - [F5H804_HUMAN]	2,53	1		
Q86TU7	Histone-lysine N-methyltransferase setd3 OS=Homo sapiens GN=SETD3 PE=1 SV=1 - [SETD3_HUMAN]	2,53	1		
C9J3F6	TBC1 domain family member 5 OS=Homo sapiens GN=TBC1D5 PE=4 SV=1 - [C9J3F6_HUMAN]	2,53	1		
Q9BVI4	Nucleolar complex protein 4 homolog OS=Homo sapiens GN=NOC4L PE=1 SV=1 - [NOC4L_HUMAN]	2,52	1		1,010
P00973	2'-5'-oligoadenylate synthase 1 OS=Homo sapiens GN=OAS1 PE=1 SV=4 - [OAS1_HUMAN]	2,50	1		0,588
Q9BV36	Melanophilin OS=Homo sapiens GN=MLPH PE=1 SV=1 - [MELPH_HUMAN]	2,50	1		
Q7Z6B0	Coiled-coil domain-containing protein 91 OS=Homo sapiens GN=CCDC91 PE=1 SV=2 - [CCD91_HUMAN]	2,49	1		
O94808	Glucosamine--fructose-6-phosphate aminotransferase [isomerizing] 2 OS=Homo sapiens GN=GFPT2 PE=1 SV=3 - [GFPT2_HUMAN]	2,49	1		
Q9UBX7	Kallikrein-11 OS=Homo sapiens GN=KLK11 PE=1 SV=2 - [KLK11_HUMAN]	2,48	1	1,277	1,630
A6NGB9	WAS/WASL-interacting protein family member 3 OS=Homo sapiens GN=WIPF3 PE=2 SV=3 - [WIPF3_HUMAN]	2,48	1		
Q86X53	Glutamate-rich protein 1 OS=Homo sapiens GN=ERICH1 PE=1 SV=1 - [ERIC1_HUMAN]	2,48	1		
Q17RY0-3	Isoform 3 of Cytoplasmic polyadenylation element-binding protein 4 OS=Homo sapiens GN=CPEB4 - [CPEB4_HUMAN]	2,48	1		
P53667	LIM domain kinase 1 OS=Homo sapiens GN=LIMK1 PE=1 SV=3 - [LIMK1_HUMAN]	2,47	1	1,041	0,801
Q8NBF6	Late secretory pathway protein AVL9 homolog OS=Homo sapiens GN=AVL9 PE=1 SV=1 - [AVL9_HUMAN]	2,47	1		
Q96A29	GDP-fucose transporter 1 OS=Homo sapiens GN=SLC35C1 PE=1 SV=1 - [FUCT1_HUMAN]	2,47	1	1,659	1,788
Q9BSC4	Nucleolar protein 10 OS=Homo sapiens GN=NOL10 PE=1 SV=1 - [NOL10_HUMAN]	2,47	1		
P52306	Rap1 GTPase-GDP dissociation stimulator 1 OS=Homo sapiens GN=RAP1GDS1 PE=1 SV=3 - [GDS1_HUMAN]	2,47	1		
Q3YEC7	Rab-like protein 1 OS=Homo sapiens GN=PARF PE=1 SV=2 - [RBEL1_HUMAN]	2,47	1		
Q7L775	EPM2A-interacting protein 1 OS=Homo sapiens GN=EPM2AIP1 PE=1 SV=1 - [EPMIP_HUMAN]	2,47	1		
F8WEM1	Ras-associating and dilute domain-containing protein OS=Homo sapiens GN=RADIL PE=4 SV=1 - [F8WEM1_HUMAN]	2,46	1		0,926
Q9UIJ5	Palmitoyltransferase ZDHHC2 OS=Homo sapiens GN=ZDHHC2 PE=2 SV=1 - [ZDHC2_HUMAN]	2,45	1	1,320	
Q9NVF7	F-box only protein 28 OS=Homo sapiens GN=FBXO28 PE=1 SV=1 - [FBX28_HUMAN]	2,45	1	1,019	
H7BXR5	Sorting nexin-29 OS=Homo sapiens GN=SNX29 PE=4 SV=1 - [H7BXR5_HUMAN]	2,45	1		
Q86WJ1	Chromodomain-helicase-DNA-binding protein 1-like OS=Homo sapiens GN=CHD1L PE=1 SV=2 - [CHD1L_HUMAN]	2,45	1		
Q15311	RalA-binding protein 1 OS=Homo sapiens GN=RALBP1 PE=1 SV=3 - [RBP1_HUMAN]	2,44	1	1,287	
Q13098	COP9 signalosome complex subunit 1 OS=Homo sapiens GN=GPS1 PE=1 SV=4 - [CSN1_HUMAN]	2,44	1	0,622	
Q8IWB7	WD repeat and FYVE domain-containing protein 1 OS=Homo sapiens GN=WDFY1 PE=1 SV=1 - [WDFY1_HUMAN]	2,44	1		0,721
Q16348-2	Isoform 2 of Solute carrier family 15 member 2 OS=Homo sapiens GN=SLC15A2 - [S15A2_HUMAN]	2,44	1		
A6NCL7	Ankyrin repeat domain-containing protein 33B OS=Homo sapiens GN=ANKRD33B PE=4 SV=1 - [AN33B_HUMAN]	2,43	1	1,398	
P49641	Alpha-mannosidase 2x OS=Homo sapiens GN=MAN2A2 PE=2 SV=3 - [MA2A2_HUMAN]	2,43	1	1,812	
Q9BS92	Protein NipSnap homolog 3B OS=Homo sapiens GN=NIPSNAP3B PE=2 SV=1 - [NPS3B_HUMAN]	2,43	1		1,800
P00439	Phenylalanine-4-hydroxylase OS=Homo sapiens GN=PAH PE=1 SV=1 - [PH4H_HUMAN]	2,43	1		0,993
Q9NV88	Integrator complex subunit 9 OS=Homo sapiens GN=INTS9 PE=1 SV=2 - [INT9_HUMAN]	2,43	1		

Q9Y3L3	SH3 domain-binding protein 1 OS=Homo sapiens GN=SH3BP1 PE=1 SV=3 - [3BP1_HUMAN]	2,43	1
Q8NFG4	Folliculin OS=Homo sapiens GN=FLCN PE=1 SV=1 - [FLCN_HUMAN]	2,42	1
P61812	Transforming growth factor beta-2 OS=Homo sapiens GN=TGFB2 PE=1 SV=1 - [TGFB2_HUMAN]	2,42	1
P14859	POU domain, class 2, transcription factor 1 OS=Homo sapiens GN=POU2F1 PE=1 SV=2 - [PO2F1_HUMAN]	2,42	1
Q9Y613	FH1/FH2 domain-containing protein 1 OS=Homo sapiens GN=FHOD1 PE=1 SV=3 - [FHOD1_HUMAN]	2,41	1
Q71SY5	Mediator of RNA polymerase II transcription subunit 25 OS=Homo sapiens GN=MED25 PE=1 SV=2 - [MED25_HUMAN]	2,41	1
Q9BYV8	Centrosomal protein of 41 kDa OS=Homo sapiens GN=CEP41 PE=1 SV=1 - [CEP41_HUMAN]	2,41	1
Q8N5P1	Zinc finger CCCH domain-containing protein 8 OS=Homo sapiens GN=ZC3H8 PE=1 SV=2 - [ZC3H8_HUMAN]	2,41	1
P21580	Tumor necrosis factor alpha-induced protein 3 OS=Homo sapiens GN=TNFAIP3 PE=1 SV=1 - [TNAP3_HUMAN]	2,41	1
Q1ED39	Protein C16orf88 OS=Homo sapiens GN=C16orf88 PE=1 SV=1 - [CP088_HUMAN]	2,40	1
Q15554	Telomeric repeat-binding factor 2 OS=Homo sapiens GN=TERF2 PE=1 SV=2 - [TERF2_HUMAN]	2,40	1
O95394	Phosphoacetylglucosamine mutase OS=Homo sapiens GN=PGM3 PE=1 SV=1 - [AGM1_HUMAN]	2,40	1
Q7Z3J2	UPF0505 protein C16orf62 OS=Homo sapiens GN=C16orf62 PE=1 SV=2 - [CP062_HUMAN]	2,39	1
O95238	SAM pointed domain-containing Ets transcription factor OS=Homo sapiens GN=SPDEF PE=1 SV=1 - [SPDEF_HUMAN]	2,39	1
Q8IX07	Zinc finger protein ZFPM1 OS=Homo sapiens GN=ZFPM1 PE=1 SV=2 - [FOG1_HUMAN]	2,39	1
Q15572-5	Isoform 5 of TATA box-binding protein-associated factor RNA polymerase I subunit C OS=Homo sapiens GN=TAF1C - [TAF1C_HUMAN]	2,39	1
P02790	Hemopexin OS=Homo sapiens GN=HPX PE=1 SV=2 - [HEMO_HUMAN]	2,38	1
Q9H9T3	Elongator complex protein 3 OS=Homo sapiens GN=ELP3 PE=1 SV=2 - [ELP3_HUMAN]	2,38	1
O75648	Mitochondrial tRNA-specific 2-thiouridylase 1 OS=Homo sapiens GN=TRMU PE=1 SV=2 - [MTU1_HUMAN]	2,38	1
Q9C0B9	Zinc finger CCHC domain-containing protein 2 OS=Homo sapiens GN=ZCCHC2 PE=1 SV=6 - [ZCHC2_HUMAN]	2,38	1
Q9BZ67	FERM domain-containing protein 8 OS=Homo sapiens GN=FRMD8 PE=1 SV=1 - [FRMD8_HUMAN]	2,37	1
Q6J9G0	Tyrosine-protein kinase STYK1 OS=Homo sapiens GN=STYK1 PE=2 SV=4 - [STYK1_HUMAN]	2,37	1
Q9UL03	Integrator complex subunit 6 OS=Homo sapiens GN=INTS6 PE=1 SV=1 - [INT6_HUMAN]	2,37	1
Q7Z6M4	mTERF domain-containing protein 2 OS=Homo sapiens GN=MTERFD2 PE=1 SV=3 - [MTER2_HUMAN]	2,36	1
P48029	Sodium- and chloride-dependent creatine transporter 1 OS=Homo sapiens GN=SLC6A8 PE=1 SV=1 - [SC6A8_HUMAN]	2,36	1
Q32P41	tRNA (guanine(37)-N1)-methyltransferase OS=Homo sapiens GN=TRMT5 PE=1 SV=2 - [TRM5_HUMAN]	2,36	1
P25105	Platelet-activating factor receptor OS=Homo sapiens GN=PTAFR PE=1 SV=1 - [PTAFR_HUMAN]	2,34	1
P14373	Zinc finger protein RFP OS=Homo sapiens GN=TRIM27 PE=1 SV=1 - [TRI27_HUMAN]	2,34	1
Q5T7W7	Thiosulfate sulfurtransferase/rhodanese-like domain-containing protein 2 OS=Homo sapiens GN=TSTD2 PE=1 SV=1 - [TSTD2_HUMAN]	2,33	1
Q2TAL8	Glutamine-rich protein 1 OS=Homo sapiens GN=QRICH1 PE=1 SV=1 - [QRIC1_HUMAN]	2,32	1
J3KSE4	Protein AF-17 (Fragment) OS=Homo sapiens GN=MLLT6 PE=4 SV=1 - [J3KSE4_HUMAN]	2,32	1
Q8IYJ3	Synaptotagmin-like protein 1 OS=Homo sapiens GN=SYTL1 PE=1 SV=1 - [SYTL1_HUMAN]	2,31	1
Q9BUM1	Glucose-6-phosphatase 3 OS=Homo sapiens GN=G6PC3 PE=1 SV=2 - [G6PC3_HUMAN]	2,31	1
H0YCD7	Oxysterol-binding protein (Fragment) OS=Homo sapiens GN=OSBPL5 PE=3 SV=1 - [H0YCD7_HUMAN]	2,31	1
O14641	Segment polarity protein dishevelled homolog DVL-2 OS=Homo sapiens GN=DVL2 PE=1 SV=1 - [DVL2_HUMAN]	2,31	1
O15083	ERC protein 2 OS=Homo sapiens GN=ERC2 PE=1 SV=3 - [ERC2_HUMAN]	2,30	1
P16383	GC-rich sequence DNA-binding factor 2 OS=Homo sapiens GN=GCFC2 PE=1 SV=2 - [GCFC2_HUMAN]	2,30	1
Q9H074	Polyadenylate-binding protein-interacting protein 1 OS=Homo sapiens GN=PAIP1 PE=1 SV=1 - [PAIP1_HUMAN]	2,30	1
B7Z7S5	Protein AF-9 OS=Homo sapiens GN=MLLT3 PE=2 SV=1 - [B7Z7S5_HUMAN]	2,30	1
Q9BXL6	Caspase recruitment domain-containing protein 14 OS=Homo sapiens GN=CARD14 PE=1 SV=2 - [CAR14_HUMAN]	2,29	1
A6NFI3	Zinc finger protein 316 OS=Homo sapiens GN=ZNF316 PE=1 SV=1 - [ZN316_HUMAN]	2,29	1
Q8IWA0	WD repeat-containing protein 75 OS=Homo sapiens GN=WDR75 PE=1 SV=1 - [WDR75_HUMAN]	2,29	1
Q53GS7	Nucleoporin GLE1 OS=Homo sapiens GN=GLE1 PE=1 SV=2 - [GLE1_HUMAN]	2,29	1
Q8IV48	3'-5' exoribonuclease 1 OS=Homo sapiens GN=ERI1 PE=1 SV=3 - [ERI1_HUMAN]	2,29	1
P51795	H(+)/Cl(-) exchange transporter 5 OS=Homo sapiens GN=CLCN5 PE=1 SV=1 - [CLCN5_HUMAN]	2,28	1
Q13888	General transcription factor IIH subunit 2 OS=Homo sapiens GN=GTF2H2 PE=1 SV=1 - [TF2H2_HUMAN]	2,28	1
P30304-2	Isoform 2 of M-phase inducer phosphatase 1 OS=Homo sapiens GN=CDC25A - [MPIP1_HUMAN]	2,27	1
P54792-2	Isoform Short of Putative Segment polarity protein dishevelled homolog DVL-1-like OS=Homo sapiens GN=DVL1L1 - [DVL1L_HUMAN]	2,27	1
Q3KP66	Uncharacterized protein C1orf106 OS=Homo sapiens GN=C1orf106 PE=2 SV=2 - [CA106_HUMAN]	2,26	1
Q6NT55	Cytochrome P450 4F22 OS=Homo sapiens GN=CYP4F22 PE=2 SV=1 - [CP4FN_HUMAN]	2,26	1
P45985	Dual specificity mitogen-activated protein kinase kinase 4 OS=Homo sapiens GN=MAP2K4 PE=1 SV=1 - [MP2K4_HUMAN]	2,26	1
Q04864	Proto-oncogene c-Rel OS=Homo sapiens GN=REL PE=1 SV=1 - [REL_HUMAN]	2,26	1
P00742	Coagulation factor X OS=Homo sapiens GN=F10 PE=1 SV=2 - [FA10_HUMAN]	2,25	1
Q9UN70	Protocadherin gamma-C3 OS=Homo sapiens GN=PCDHGC3 PE=1 SV=1 - [PCDGK_HUMAN]	2,25	1
Q8IZN3	Probable palmitoyltransferase ZDHHC14 OS=Homo sapiens GN=ZDHHC14 PE=1 SV=1 - [ZDH14_HUMAN]	2,25	1
Q6P499	NIPA-like protein 3 OS=Homo sapiens GN=NIPAL3 PE=1 SV=1 - [NPAL3_HUMAN]	2,22	1
Q8WWB5	PIH1 domain-containing protein 2 OS=Homo sapiens GN=PIH1D2 PE=2 SV=1 - [PIHD2_HUMAN]	2,22	1
Q9BSE4	Homocysteine-responsive endoplasmic reticulum-resident ubiquitin-like domain member 2 protein OS=Homo sapiens GN=HERPUD2 PE=	2,22	1
P26012	Integrin beta-8 OS=Homo sapiens GN=ITGB8 PE=1 SV=1 - [ITB8_HUMAN]	2,21	1
O60858	E3 ubiquitin-protein ligase TRIM13 OS=Homo sapiens GN=TRIM13 PE=1 SV=2 - [TRI13_HUMAN]	2,21	1
P17030-2	Isoform 2 of Zinc finger protein 25 OS=Homo sapiens GN=ZNF25 - [ZNF25_HUMAN]	2,21	1
Q96T98	Protocadherin-psi1 OS=Homo sapiens GN=PCDHB17 PE=2 SV=1 - [Q96T98_HUMAN]	2,21	1
Q96IV0-2	Isoform 2 of Peptide-N(4)-(N-acetyl-beta-glucosaminyl)asparagine amidase OS=Homo sapiens GN=NGLY1 - [NGLY1_HUMAN]	2,20	1
P29400	Collagen alpha-5(IV) chain OS=Homo sapiens GN=COL4A5 PE=1 SV=2 - [CO4A5_HUMAN]	2,20	1
Q9NZC9	SWI/SNF-related matrix-associated actin-dependent regulator of chromatin subfamily A-like protein 1 OS=Homo sapiens GN=SMARCAL1	2,20	1
Q06330	Recombining binding protein suppressor of hairless OS=Homo sapiens GN=RBPJ PE=1 SV=3 - [SUH_HUMAN]	2,20	1
Q6NXE6	Armadillo repeat-containing protein 6 OS=Homo sapiens GN=ARMC6 PE=1 SV=2 - [ARMC6_HUMAN]	2,20	1
Q9NZ71-5	Isoform 5 of Regulator of telomere elongation helicase 1 OS=Homo sapiens GN=RTEL1 - [RTEL1_HUMAN]	2,20	1
Q96RR1	Twinkle protein, mitochondrial OS=Homo sapiens GN=PEO1 PE=1 SV=1 - [PEO1_HUMAN]	2,19	1
P00488	Coagulation factor XIII A chain OS=Homo sapiens GN=F13A1 PE=1 SV=4 - [F13A_HUMAN]	2,19	1
G5E9I1	Male-specific lethal 2 homolog OS=Homo sapiens GN=MSL2 PE=4 SV=1 - [G5E9I1_HUMAN]	2,19	1
Q8WTS6	Histone-lysine N-methyltransferase SETD7 OS=Homo sapiens GN=SETD7 PE=1 SV=1 - [SETD7_HUMAN]	2,19	1
Q13451	Peptidyl-prolyl cis-trans isomerase FKBP5 OS=Homo sapiens GN=FKBP5 PE=1 SV=2 - [FKBP5_HUMAN]	2,19	1
Q14642	Type I inositol-1,4,5-trisphosphate 5-phosphatase OS=Homo sapiens GN=INPP5A PE=2 SV=1 - [I5P1_HUMAN]	2,18	1
Q96NL6	Sodium channel and clathrin linker 1 OS=Homo sapiens GN=SCLT1 PE=1 SV=2 - [SCLT1_HUMAN]	2,18	1
H0YLW6	SH2 domain-containing adapter protein F OS=Homo sapiens GN=SHF PE=4 SV=1 - [H0YLW6_HUMAN]	2,18	1
P98153	Integral membrane protein DGCR2/IDD OS=Homo sapiens GN=DGCR2 PE=1 SV=1 - [IDD_HUMAN]	2,18	1
Q8TBE7	Transmembrane protein 22 OS=Homo sapiens GN=TMEM22 PE=1 SV=3 - [TMM22_HUMAN]	2,18	1
Q14CN2	Calcium-activated chloride channel regulator 4 OS=Homo sapiens GN=CLCA4 PE=1 SV=2 - [CLCA4_HUMAN]	2,18	1
Q9H9E3	Conserved oligomeric Golgi complex subunit 4 OS=Homo sapiens GN=COG4 PE=1 SV=3 - [COG4_HUMAN]	2,17	1
Q14162	Scavenger receptor class F member 1 OS=Homo sapiens GN=SCARF1 PE=1 SV=3 - [SREC_HUMAN]	2,17	1
Q6NXR4	TELO2-interacting protein 2 OS=Homo sapiens GN=TTI2 PE=1 SV=1 - [TTI2_HUMAN]	2,17	1
Q6UWJ1-3	Isoform 3 of Transmembrane and coiled-coil domain-containing protein 3 OS=Homo sapiens GN=TMCO3 - [TMCO3_HUMAN]	2,17	1
Q9BXI6	TBC1 domain family member 10A OS=Homo sapiens GN=TBC1D10A PE=1 SV=1 - [TB10A_HUMAN]	2,17	1
Q14181	DNA polymerase alpha subunit B OS=Homo sapiens GN=POLA2 PE=1 SV=2 - [DPOA2_HUMAN]	2,17	1
Q99871	HAUS augmin-like complex subunit 7 OS=Homo sapiens GN=HAUS7 PE=1 SV=3 - [HAUS7_HUMAN]	2,17	1
Q9H7D7-2	Isoform 2 of WD repeat-containing protein 26 OS=Homo sapiens GN=WDR26 - [WDR26_HUMAN]	2,17	1
O95396	Adenylyltransferase and sulfurtransferase MOCS3 OS=Homo sapiens GN=MOCS3 PE=1 SV=1 - [MOCS3_HUMAN]	2,17	1
Q14409	Putative glycerol kinase 3 OS=Homo sapiens GN=GK3P PE=5 SV=2 - [GLPK3_HUMAN]	2,17	1
Q6ZUS5	Coiled-coil domain-containing protein 121 OS=Homo sapiens GN=CCDC121 PE=2 SV=1 - [CC121_HUMAN]	2,16	1
Q5SRI9	Glycoprotein endo-alpha-1,2-mannosidase OS=Homo sapiens GN=MANEA PE=1 SV=1 - [MANEA_HUMAN]	2,16	1
Q96H96	4-hydroxybenzoate polyprenyltransferase, mitochondrial OS=Homo sapiens GN=COQ2 PE=1 SV=1 - [COQ2_HUMAN]	2,16	1
Q8NI60	Chaperone activity of bc1 complex-like, mitochondrial OS=Homo sapiens GN=ADCK3 PE=1 SV=1 - [ADCK3_HUMAN]	2,16	1
Q92888-2	Isoform 2 of Rho guanine nucleotide exchange factor 1 OS=Homo sapiens GN=ARHGEF1 - [ARHG1_HUMAN]	2,16	1
Q8N6R0	Methyltransferase-like protein 13 OS=Homo sapiens GN=METTL13 PE=1 SV=1 - [MTL13_HUMAN]	2,15	1
P54802	Alpha-N-acetylglucosaminidase OS=Homo sapiens GN=NAGLU PE=1 SV=2 - [ANAG_HUMAN]	2,15	1
Q8NFK1	Gap junction gamma-3 protein OS=Homo sapiens GN=GJC3 PE=2 SV=1 - [CXG3_HUMAN]	2,15	1
A3KN83	Protein strawberry notch homolog 1 OS=Homo sapiens GN=SBNO1 PE=1 SV=1 - [SBNO1_HUMAN]	2,15	1
Q7Z698	Sprouty-related, EVH1 domain-containing protein 2 OS=Homo sapiens GN=SPRED2 PE=1 SV=2 - [SPRE2_HUMAN]	2,15	1
Q9Y263	Phospholipase A-2-activating protein OS=Homo sapiens GN=PLAA PE=1 SV=2 - [PLAP_HUMAN]	2,14	1
Q8TCG1-2	Isoform 2 of Protein CIP2A OS=Homo sapiens GN=KIAA1524 - [CIP2A_HUMAN]	2,14	1
Q9Y6U3-3	Isoform 3 of Adseverin OS=Homo sapiens GN=SCIN - [ADSV_HUMAN]	2,14	1
O14793	Growth/differentiation factor 8 OS=Homo sapiens GN=MSTN PE=1 SV=1 - [GDF8_HUMAN]	2,13	1
Q9P0U4	CpG-binding protein OS=Homo sapiens GN=CXXC1 PE=1 SV=2 - [CXXC1_HUMAN]	2,13	1
Q9UJX4	Anaphase-promoting complex subunit 5 OS=Homo sapiens GN=ANAPC5 PE=1 SV=2 - [APC5_HUMAN]	2,12	1
Q6ZN55	Zinc finger protein 574 OS=Homo sapiens GN=ZNF574 PE=1 SV=2 - [ZN574_HUMAN]	2,12	1
Q8N806	Putative E3 ubiquitin-protein ligase UBR7 OS=Homo sapiens GN=UBR7 PE=1 SV=2 - [UBR7_HUMAN]	2,12	1
Q5BKT4	Dol-P-Glc:Glc(2)Man(9)GlcNAc(2)-PP-Dol alpha-1,2-glucosyltransferase OS=Homo sapiens GN=ALG10 PE=1 SV=1 - [AG10A_HUMAN]	2,11	1
Q9BRB3	Phosphatidylinositol N-acetylglucosaminyltransferase subunit Q OS=Homo sapiens GN=PIGQ PE=1 SV=3 - [PIGQ_HUMAN]	2,11	1
Q8NBP5	Major facilitator superfamily domain-containing protein 9 OS=Homo sapiens GN=MFSD9 PE=2 SV=2 - [MFSD9_HUMAN]	2,11	1
P28347	Transcriptional enhancer factor TEF-1 OS=Homo sapiens GN=TEAD1 PE=1 SV=2 - [TEAD1_HUMAN]	2,11	1
Q96T21	Selenocysteine insertion sequence-binding protein 2 OS=Homo sapiens GN=SECISBP2 PE=1 SV=2 - [SEBP2_HUMAN]	2,11	1
Q9Y2X7	ARF GTPase-activating protein GIT1 OS=Homo sapiens GN=GIT1 PE=1 SV=2 - [GIT1_HUMAN]	2,10	1
Q9NZM3-4	Isoform 4 of Intersectin-2 OS=Homo sapiens GN=ITSN2 - [ITSN2_HUMAN]	2,10	1
O43148	mRNA cap guanine-N7 methyltransferase OS=Homo sapiens GN=RNMT PE=1 SV=1 - [MCES_HUMAN]	2,10	1
P56270	Myc-associated zinc finger protein OS=Homo sapiens GN=MAZ PE=1 SV=1 - [MAZ_HUMAN]	2,10	1
Q9Y2G5	GDP-fucose protein O-fucosyltransferase 2 OS=Homo sapiens GN=POFUT2 PE=1 SV=3 - [OFUT2_HUMAN]	2,10	1
Q8NDF8	PAP-associated domain-containing protein 5 OS=Homo sapiens GN=PAPD5 PE=1 SV=2 - [PAPD5_HUMAN]	2,10	1
Q6N075-2	Isoform 2 of Major facilitator superfamily domain-containing protein 5 OS=Homo sapiens GN=MFSD5 - [MFSD5_HUMAN]	2,09	1
Q502W6	von Willebrand factor A domain-containing protein 3B OS=Homo sapiens GN=VWA3B PE=2 SV=3 - [VWA3B_HUMAN]	2,09	1
Q9UDY8-2	Isoform 2 of Mucosa-associated lymphoid tissue lymphoma translocation protein 1 OS=Homo sapiens GN=MALT1 - [MALT1_HUMAN]	2,09	1
Q9UK39	Nocturnin OS=Homo sapiens GN=CCRN4L PE=2 SV=2 - [NOCT_HUMAN]	2,09	1
Q96F46	Interleukin-17 receptor A OS=Homo sapiens GN=IL17RA PE=1 SV=2 - [I17RA_HUMAN]	2,08	1
Q5T160	Probable arginine--tRNA ligase, mitochondrial OS=Homo sapiens GN=RARS2 PE=1 SV=1 - [SYRM_HUMAN]	2,08	1
Q9HC16	DNA dC->dU-editing enzyme APOBEC-3G OS=Homo sapiens GN=APOBEC3G PE=1 SV=1 - [ABC3G_HUMAN]	2,08	1
Q9Y6V7	Probable ATP-dependent RNA helicase DDX49 OS=Homo sapiens GN=DDX49 PE=1 SV=1 - [DDX49_HUMAN]	2,07	1
O43913	Origin recognition complex subunit 5 OS=Homo sapiens GN=ORC5 PE=1 SV=1 - [ORC5_HUMAN]	2,07	1
P15309	Prostatic acid phosphatase OS=Homo sapiens GN=ACPP PE=1 SV=3 - [PPAP_HUMAN]	2,07	1
Q86UE8	Serine/threonine-protein kinase tousled-like 2 OS=Homo sapiens GN=TLK2 PE=1 SV=2 - [TLK2_HUMAN]	2,07	1
Q9H8Y5	Ankyrin repeat and zinc finger domain-containing protein 1 OS=Homo sapiens GN=ANKZF1 PE=1 SV=1 - [ANKZ1_HUMAN]	2,07	1
Q8IZ52	Chondroitin sulfate synthase 2 OS=Homo sapiens GN=CHPF PE=1 SV=2 - [CHSS2_HUMAN]	2,06	1
Q16760	Diacylglycerol kinase delta OS=Homo sapiens GN=DGKD PE=1 SV=4 - [DGKD_HUMAN]	2,06	1
Q9BRD0-2	Isoform 2 of BUD13 homolog OS=Homo sapiens GN=BUD13 - [BUD13_HUMAN]	2,06	1
Q5HYJ3	Protein FAM76B OS=Homo sapiens GN=FAM76B PE=1 SV=3 - [FA76B_HUMAN]	2,06	1
Q24JP5	Transmembrane protein 132A OS=Homo sapiens GN=TMEM132A PE=2 SV=1 - [T132A_HUMAN]	2,05	1
Q1MSJ5-2	Isoform 2 of Centrosome and spindle pole-associated protein 1 OS=Homo sapiens GN=CSPP1 - [CSPP1_HUMAN]	2,05	1
Q9NV70-2	Isoform 2 of Exocyst complex component 1 OS=Homo sapiens GN=EXOC1 - [EXOC1_HUMAN]	2,05	1
Q9BRP4	Proteasomal ATPase-associated factor 1 OS=Homo sapiens GN=PAAF1 PE=1 SV=2 - [PAAF1_HUMAN]	2,04	1
F5H4B1	Melanoma antigen preferentially-expressed in tumors OS=Homo sapiens GN=PRAME PE=4 SV=1 - [F5H4B1_HUMAN]	2,03	1
Q9NRA2	Sialin OS=Homo sapiens GN=SLC17A5 PE=1 SV=2 - [S17A5_HUMAN]	2,02	1
E7ENV5	Zinc finger protein 623 OS=Homo sapiens GN=ZNF623 PE=4 SV=1 - [E7ENV5_HUMAN]	2,02	1
P02787	Serotransferrin OS=Homo sapiens GN=TF PE=1 SV=3 - [TRFE_HUMAN]	2,01	1
Q8NCR0	UDP-GalNAc:beta-1,3-N-acetylgalactosaminyltransferase 2 OS=Homo sapiens GN=B3GALNT2 PE=1 SV=1 - [B3GL2_HUMAN]	2,00	1
Q96M02	Centrosomal protein C10orf90 OS=Homo sapiens GN=C10orf90 PE=2 SV=2 - [CJ090_HUMAN]	2,00	1
Q86X95	Corepressor interacting with RBPJ 1 OS=Homo sapiens GN=CIR1 PE=1 SV=1 - [CIR1_HUMAN]	2,00	1
Q9NYQ3	Hydroxyacid oxidase 2 OS=Homo sapiens GN=HAO2 PE=2 SV=1 - [HAOX2_HUMAN]	1,99	1
Q8WUH2	Transforming growth factor-beta receptor-associated protein 1 OS=Homo sapiens GN=TGFBRAP1 PE=1 SV=1 - [TGFA1_HUMAN]	1,98	1
Q8WUQ7	Uncharacterized protein C19orf29 OS=Homo sapiens GN=C19orf29 PE=1 SV=3 - [CS029_HUMAN]	1,98	1
Q8IW35	Centrosomal protein of 97 kDa OS=Homo sapiens GN=CEP97 PE=1 SV=1 - [CEP97_HUMAN]	1,97	1
Q9UGQ3	Solute carrier family 2, facilitated glucose transporter member 6 OS=Homo sapiens GN=SLC2A6 PE=1 SV=2 - [GTR6_HUMAN]	1,97	1
Q9H0G5	Nuclear speckle splicing regulatory protein 1 OS=Homo sapiens GN=NSRP1 PE=1 SV=1 - [NSRP1_HUMAN]	1,97	1
Q96EP0	RING finger protein 31 OS=Homo sapiens GN=RNF31 PE=1 SV=1 - [RNF31_HUMAN]	1,96	1
Q96EP1	E3 ubiquitin-protein ligase CHFR OS=Homo sapiens GN=CHFR PE=1 SV=2 - [CHFR_HUMAN]	1,96	1
Q9H237	Protein-cysteine N-palmitoyltransferase porcupine OS=Homo sapiens GN=PORCN PE=1 SV=2 - [PORCN_HUMAN]	1,95	1
Q8N8Q9	Magnesium transporter NIPA2 OS=Homo sapiens GN=NIPA2 PE=2 SV=1 - [NIPA2_HUMAN]	1,94	1
Q9NP61	ADP-ribosylation factor GTPase-activating protein 3 OS=Homo sapiens GN=ARFGAP3 PE=1 SV=1 - [ARFG3_HUMAN]	1,94	1
B8ZZI9	Aftiphilin OS=Homo sapiens GN=AFTPH PE=4 SV=1 - [B8ZZI9_HUMAN]	1,94	1
Q86UW9	Protein deltex-2 OS=Homo sapiens GN=DTX2 PE=1 SV=3 - [DTX2_HUMAN]	1,93	1
O43462	Membrane-bound transcription factor site-2 protease OS=Homo sapiens GN=MBTPS2 PE=1 SV=1 - [MBTP2_HUMAN]	1,93	1
Q9P2G1	Ankyrin repeat and IBR domain-containing protein 1 OS=Homo sapiens GN=ANKIB1 PE=1 SV=3 - [AKIB1_HUMAN]	1,93	1
Q9UEY8-2	Isoform 1 of Gamma-adducin OS=Homo sapiens GN=ADD3 - [ADDG_HUMAN]	1,93	1
Q96SY0	UPF0464 protein C15orf44 OS=Homo sapiens GN=C15orf44 PE=1 SV=2 - [CO044_HUMAN]	1,93	1
P07225	Vitamin K-dependent protein S OS=Homo sapiens GN=PROS1 PE=1 SV=1 - [PROS_HUMAN]	1,92	1
Q92543	Sorting nexin-19 OS=Homo sapiens GN=SNX19 PE=1 SV=2 - [SNX19_HUMAN]	1,92	1
Q96FS4	Signal-induced proliferation-associated protein 1 OS=Homo sapiens GN=SIPA1 PE=1 SV=1 - [SIPA1_HUMAN]	1,92	1
Q8N884	Protein MB21D1 OS=Homo sapiens GN=MB21D1 PE=1 SV=2 - [M21D1_HUMAN]	1,92	1
J3QSD7	Cyclin-dependent kinase 12 (Fragment) OS=Homo sapiens GN=CDK12 PE=4 SV=1 - [J3QSD7_HUMAN]	1,92	1
P12645	Bone morphogenetic protein 3 OS=Homo sapiens GN=BMP3 PE=1 SV=2 - [BMP3_HUMAN]	1,91	1
Q8TB22	Spermatogenesis-associated protein 20 OS=Homo sapiens GN=SPATA20 PE=2 SV=3 - [SPT20_HUMAN]	1,91	1
Q7L7X3	Serine/threonine-protein kinase TAO1 OS=Homo sapiens GN=TAOK1 PE=1 SV=1 - [TAOK1_HUMAN]	1,90	1

E9PJY3	Centrosomal protein KIAA1731 (Fragment) OS=Homo sapiens GN=KIAA1731 PE=4 SV=1 - [E9PJY3_HUMAN]	1,90	1	
O95342	Bile salt export pump OS=Homo sapiens GN=ABCB11 PE=1 SV=2 - [ABCBB_HUMAN]	1,89	1	**3,833**
Q8N2G8	GH3 domain-containing protein OS=Homo sapiens GN=GHDC PE=1 SV=2 - [GHDC_HUMAN]	1,89	1	
Q9UKD1	Glucocorticoid modulatory element-binding protein 2 OS=Homo sapiens GN=GMEB2 PE=1 SV=1 - [GMEB2_HUMAN]	1,89	1	
Q9NW75	G patch domain-containing protein 2 OS=Homo sapiens GN=GPATCH2 PE=1 SV=1 - [GPTC2_HUMAN]	1,89	1	
O95453	Poly(A)-specific ribonuclease PARN OS=Homo sapiens GN=PARN PE=1 SV=1 - [PARN_HUMAN]	1,88	1	
Q8WUA4-2	Isoform 2 of General transcription factor 3C polypeptide 2 OS=Homo sapiens GN=GTF3C2 - [TF3C2_HUMAN]	1,88	1	
P42695	Condensin-2 complex subunit D3 OS=Homo sapiens GN=NCAPD3 PE=1 SV=2 - [CNDD3_HUMAN]	1,87	1	1,174
O75044	SLIT-ROBO Rho GTPase-activating protein 2 OS=Homo sapiens GN=SRGAP2 PE=1 SV=2 - [FNBP2_HUMAN]	1,87	1	0,961
Q13547	Histone deacetylase 1 OS=Homo sapiens GN=HDAC1 PE=1 SV=1 - [HDAC1_HUMAN]	1,87	1	
Q6P1L5	Protein FAM117B OS=Homo sapiens GN=FAM117B PE=1 SV=2 - [F117B_HUMAN]	1,87	1	
Q7Z2Z2-2	Isoform 2 of Elongation factor Tu GTP-binding domain-containing protein 1 OS=Homo sapiens GN=EFTUD1 - [ETUD1_HUMAN]	1,87	1	
Q6ZNA5	Ferric-chelate reductase 1 OS=Homo sapiens GN=FRRS1 PE=2 SV=2 - [FRRS1_HUMAN]	1,86	1	0,900
P35240	Merlin OS=Homo sapiens GN=NF2 PE=1 SV=1 - [MERL_HUMAN]	1,85	1	1,744
Q9BTW9	Tubulin-specific chaperone D OS=Homo sapiens GN=TBCD PE=1 SV=2 - [TBCD_HUMAN]	1,85	1	
Q9P253	Vacuolar protein sorting-associated protein 18 homolog OS=Homo sapiens GN=VPS18 PE=1 SV=2 - [VPS18_HUMAN]	1,85	1	
Q15345	Leucine-rich repeat-containing protein 41 OS=Homo sapiens GN=LRRC41 PE=1 SV=3 - [LRC41_HUMAN]	1,85	1	
P00156	Cytochrome b OS=Homo sapiens GN=MT-CYB PE=1 SV=1 - [CYB_HUMAN]	1,84	1	0,720
Q9Y2I9	TBC1 domain family member 30 OS=Homo sapiens GN=TBC1D30 PE=1 SV=2 - [TBC30_HUMAN]	1,84	1	1,204
Q9BW62	Katanin p60 ATPase-containing subunit A-like 1 OS=Homo sapiens GN=KATNAL1 PE=2 SV=1 - [KATL1_HUMAN]	1,84	1	
O94888	UBX domain-containing protein 7 OS=Homo sapiens GN=UBXN7 PE=1 SV=2 - [UBXN7_HUMAN]	1,84	1	
Q9BX69	Caspase recruitment domain-containing protein 6 OS=Homo sapiens GN=CARD6 PE=1 SV=2 - [CARD6_HUMAN]	1,83	1	
Q92805	Golgin subfamily A member 1 OS=Homo sapiens GN=GOLGA1 PE=1 SV=3 - [GOGA1_HUMAN]	1,83	1	
Q9BRR8	G patch domain-containing protein 1 OS=Homo sapiens GN=GPATCH1 PE=1 SV=1 - [GPTC1_HUMAN]	1,83	1	
O75449	Katanin p60 ATPase-containing subunit A1 OS=Homo sapiens GN=KATNA1 PE=1 SV=1 - [KTNA1_HUMAN]	1,83	1	
G3V1P0	Pogo transposable element with KRAB domain OS=Homo sapiens GN=POGK PE=4 SV=1 - [G3V1P0_HUMAN]	1,83	1	
Q9HCN3	Transmembrane protein 8A OS=Homo sapiens GN=TMEM8A PE=1 SV=3 - [TMM8A_HUMAN]	1,82	1	
B3KTU7	Gamma-tubulin complex component 2 OS=Homo sapiens GN=TUBGCP2 PE=2 SV=1 - [B3KTU7_HUMAN]	1,82	1	
Q8NFH8-4	Isoform 4 of RalBP1-associated Eps domain-containing protein 2 OS=Homo sapiens GN=REPS2 - [REPS2_HUMAN]	1,82	1	
Q9UJX6	Anaphase-promoting complex subunit 2 OS=Homo sapiens GN=ANAPC2 PE=1 SV=1 - [ANC2_HUMAN]	1,82	1	
P54646	5'-AMP-activated protein kinase catalytic subunit alpha-2 OS=Homo sapiens GN=PRKAA2 PE=1 SV=2 - [AAPK2_HUMAN]	1,81	1	0,931
O00445	Synaptotagmin-5 OS=Homo sapiens GN=SYT5 PE=2 SV=2 - [SYT5_HUMAN]	1,81	1	
Q9Y2B1	Transmembrane protein 5 OS=Homo sapiens GN=TMEM5 PE=2 SV=1 - [TMEM5_HUMAN]	1,81	1	
Q9Y4G2	Pleckstrin homology domain-containing family M member 1 OS=Homo sapiens GN=PLEKHM1 PE=1 SV=3 - [PKHM1_HUMAN]	1,80	1	0,764
E7EM93	Peroxisomal NADH pyrophosphatase NUDT12 OS=Homo sapiens GN=NUDT12 PE=4 SV=1 - [E7EM93_HUMAN]	1,80	1	1,039
Q9Y2J4-3	Isoform 3 of Angiomotin-like protein 2 OS=Homo sapiens GN=AMOTL2 - [AMOL2_HUMAN]	1,80	1	1,454
O95251	Histone acetyltransferase KAT7 OS=Homo sapiens GN=KAT7 PE=1 SV=1 - [KAT7_HUMAN]	1,80	1	
Q9NV58	E3 ubiquitin-protein ligase RNF19A OS=Homo sapiens GN=RNF19A PE=1 SV=3 - [RN19A_HUMAN]	1,79	1	1,401
Q9Y6D5	Brefeldin A-inhibited guanine nucleotide-exchange protein 2 OS=Homo sapiens GN=ARFGEF2 PE=1 SV=3 - [BIG2_HUMAN]	1,79	1	
Q13131	5'-AMP-activated protein kinase catalytic subunit alpha-1 OS=Homo sapiens GN=PRKAA1 PE=1 SV=4 - [AAPK1_HUMAN]	1,79	1	
Q5VWP2	Protein FAM46C OS=Homo sapiens GN=FAM46C PE=2 SV=1 - [FA46C_HUMAN]	1,79	1	
P49902	Cytosolic purine 5'-nucleotidase OS=Homo sapiens GN=NT5C2 PE=1 SV=1 - [5NTC_HUMAN]	1,78	1	0,784
Q9HCI6	Uncharacterized protein KIAA1586 OS=Homo sapiens GN=KIAA1586 PE=2 SV=2 - [K1586_HUMAN]	1,78	1	1,030
P10909	Clusterin OS=Homo sapiens GN=CLU PE=1 SV=1 - [CLUS_HUMAN]	1,78	1	1,625
P15289	Arylsulfatase A OS=Homo sapiens GN=ARSA PE=1 SV=3 - [ARSA_HUMAN]	1,78	1	1,212
Q05655	Protein kinase C delta type OS=Homo sapiens GN=PRKCD PE=1 SV=2 - [KPCD_HUMAN]	1,78	1	1,103
Q8WYA6	Beta-catenin-like protein 1 OS=Homo sapiens GN=CTNNBL1 PE=1 SV=1 - [CTBL1_HUMAN]	1,78	1	
Q9UKV0	Histone deacetylase 9 OS=Homo sapiens GN=HDAC9 PE=1 SV=2 - [HDAC9_HUMAN]	1,78	1	
Q15464	SH2 domain-containing adapter protein B OS=Homo sapiens GN=SHB PE=1 SV=2 - [SHB_HUMAN]	1,77	1	0,793
Q8N3X1	Formin-binding protein 4 OS=Homo sapiens GN=FNBP4 PE=1 SV=3 - [FNBP4_HUMAN]	1,77	1	
Q14145	Kelch-like ECH-associated protein 1 OS=Homo sapiens GN=KEAP1 PE=1 SV=2 - [KEAP1_HUMAN]	1,76	1	0,748
P51805	Plexin-A3 OS=Homo sapiens GN=PLXNA3 PE=1 SV=2 - [PLXA3_HUMAN]	1,76	1	0,860
B4DKD1	Protein tweety homolog 2 OS=Homo sapiens GN=TTYH2 PE=2 SV=1 - [B4DKD1_HUMAN]	1,75	1	0,989
Q9HB90	Ras-related GTP-binding protein C OS=Homo sapiens GN=RRAGC PE=1 SV=1 - [RRAGC_HUMAN]	1,75	1	
Q5VWQ0	Round spermatid basic protein 1 OS=Homo sapiens GN=RSBN1 PE=2 SV=2 - [RSBN1_HUMAN]	1,75	1	
Q5SZL2	Centrosomal protein of 85 kDa-like OS=Homo sapiens GN=CEP85L PE=1 SV=1 - [CE85L_HUMAN]	1,74	1	1,864
E7ETT3	Rod cGMP-specific 3',5'-cyclic phosphodiesterase subunit beta OS=Homo sapiens GN=PDE6B PE=4 SV=1 - [E7ETT3_HUMAN]	1,74	1	1,173
P18858	DNA ligase 1 OS=Homo sapiens GN=LIG1 PE=1 SV=1 - [DNLI1_HUMAN]	1,74	1	
Q9BSL1	Ubiquitin-associated domain-containing protein 1 OS=Homo sapiens GN=UBAC1 PE=1 SV=1 - [UBAC1_HUMAN]	1,73	1	
A1L020	RNA-binding protein MEX3A OS=Homo sapiens GN=MEX3A PE=1 SV=1 - [MEX3A_HUMAN]	1,73	1	
P08151	Zinc finger protein GLI1 OS=Homo sapiens GN=GLI1 PE=1 SV=1 - [GLI1_HUMAN]	1,72	1	
Q5VIR6	Vacuolar protein sorting-associated protein 53 homolog OS=Homo sapiens GN=VPS53 PE=1 SV=1 - [VPS53_HUMAN]	1,72	1	
Q9UBF8	Phosphatidylinositol 4-kinase beta OS=Homo sapiens GN=PI4KB PE=1 SV=1 - [PI4KB_HUMAN]	1,72	1	
Q6UWE0-2	Isoform 2 of E3 ubiquitin-protein ligase LRSAM1 OS=Homo sapiens GN=LRSAM1 - [LRSM1_HUMAN]	1,72	1	
Q5NDL2	Uncharacterized glycosyltransferase AER61 OS=Homo sapiens GN=AER61 PE=2 SV=1 - [AER61_HUMAN]	1,71	1	1,247
Q53FZ2	Acyl-coenzyme A synthetase ACSM3, mitochondrial OS=Homo sapiens GN=ACSM3 PE=1 SV=2 - [ACSM3_HUMAN]	1,71	1	0,669
Q04446	1,4-alpha-glucan-branching enzyme OS=Homo sapiens GN=GBE1 PE=1 SV=3 - [GLGB_HUMAN]	1,71	1	1,065
D6RF76	Coiled-coil domain-containing protein 112 OS=Homo sapiens GN=CCDC112 PE=4 SV=1 - [D6RF76_HUMAN]	1,69	1	0,855
Q96LT4	Sphingomyelin synthase-related protein 1 OS=Homo sapiens GN=SAMD8 PE=2 SV=2 - [SAMD8_HUMAN]	1,69	1	
O60346	PH domain leucine-rich repeat-containing protein phosphatase 1 OS=Homo sapiens GN=PHLPP1 PE=1 SV=3 - [PHLP1_HUMAN]	1,69	1	
Q7Z569	BRCA1-associated protein OS=Homo sapiens GN=BRAP PE=1 SV=2 - [BRAP_HUMAN]	1,69	1	
Q9H4B0	Probable tRNA threonylcarbamoyladenosine biosynthesis protein OSGEPL1 OS=Homo sapiens GN=OSGEPL1 PE=2 SV=2 - [OSGP2_HUMA	1,69	1	
Q9NNW5	WD repeat-containing protein 6 OS=Homo sapiens GN=WDR6 PE=1 SV=1 - [WDR6_HUMAN]	1,69	1	
A0AV02	Solute carrier family 12 member 8 OS=Homo sapiens GN=SLC12A8 PE=2 SV=3 - [S12A8_HUMAN]	1,68	1	0,815
Q04756	Hepatocyte growth factor activator OS=Homo sapiens GN=HGFAC PE=1 SV=1 - [HGFA_HUMAN]	1,68	1	0,515
Q9UPQ8	Dolichol kinase OS=Homo sapiens GN=DOLK PE=1 SV=1 - [DOLK_HUMAN]	1,67	1	0,841
Q9P2F8-2	Isoform 2 of Signal-induced proliferation-associated 1-like protein 2 OS=Homo sapiens GN=SIPA1L2 - [SI1L2_HUMAN]	1,67	1	0,845
Q13144	Translation initiation factor eIF-2B subunit epsilon OS=Homo sapiens GN=EIF2B5 PE=1 SV=3 - [EI2BE_HUMAN]	1,66	1	0,740
Q8IY33	MICAL-like protein 2 OS=Homo sapiens GN=MICALL2 PE=1 SV=1 - [MILK2_HUMAN]	1,66	1	
Q7L4P6	BEN domain-containing protein 5 OS=Homo sapiens GN=BEND5 PE=1 SV=1 - [BEND5_HUMAN]	1,66	1	
Q63ZE4	Solute carrier family 22 member 10 OS=Homo sapiens GN=SLC22A10 PE=2 SV=2 - [S22AA_HUMAN]	1,66	1	
B5MEB7	ATR-interacting protein OS=Homo sapiens GN=ATRIP PE=4 SV=2 - [B5MEB7_HUMAN]	1,66	1	
E9PKN0	Pre-mRNA cleavage complex 2 protein Pcf11 (Fragment) OS=Homo sapiens GN=PCF11 PE=4 SV=1 - [E9PKN0_HUMAN]	1,66	1	
Q53R41	FAST kinase domain-containing protein 1 OS=Homo sapiens GN=FASTKD1 PE=1 SV=1 - [FAKD1_HUMAN]	1,65	1	0,576
O95935	T-box transcription factor TBX18 OS=Homo sapiens GN=TBX18 PE=2 SV=3 - [TBX18_HUMAN]	1,65	1	
Q9BV10	Dol-P-Man:Man(7)GlcNAc(2)-PP-Dol alpha-1,6-mannosyltransferase OS=Homo sapiens GN=ALG12 PE=1 SV=1 - [ALG12_HUMAN]	1,64	1	0,795
Q96C24	Synaptotagmin-like protein 4 OS=Homo sapiens GN=SYTL4 PE=1 SV=2 - [SYTL4_HUMAN]	1,64	1	
Q86UV5	Ubiquitin carboxyl-terminal hydrolase 48 OS=Homo sapiens GN=USP48 PE=1 SV=1 - [UBP48_HUMAN]	1,64	1	
Q6ZU65	Ubinuclein-2 OS=Homo sapiens GN=UBN2 PE=1 SV=2 - [UBN2_HUMAN]	1,63	1	
Q13033	Striatin-3 OS=Homo sapiens GN=STRN3 PE=1 SV=3 - [STRN3_HUMAN]	1,63	1	
P13497	Bone morphogenetic protein 1 OS=Homo sapiens GN=BMP1 PE=1 SV=2 - [BMP1_HUMAN]	1,62	1	1,244
D6RA70	Probable ATP-dependent RNA helicase YTHDC2 OS=Homo sapiens GN=YTHDC2 PE=4 SV=1 - [D6RA70_HUMAN]	1,62	1	0,989
Q96MX3	Zinc finger protein 48 OS=Homo sapiens GN=ZNF48 PE=2 SV=2 - [ZNF48_HUMAN]	1,62	1	
Q9BZQ6	ER degradation-enhancing alpha-mannosidase-like 3 OS=Homo sapiens GN=EDEM3 PE=1 SV=2 - [EDEM3_HUMAN]	1,61	1	1,256
P52732	Kinesin-like protein KIF11 OS=Homo sapiens GN=KIF11 PE=1 SV=2 - [KIF11_HUMAN]	1,61	1	
Q3ZCT8	Kelch repeat and BTB domain-containing protein 12 OS=Homo sapiens GN=KBTBD12 PE=2 SV=2 - [KBTBC_HUMAN]	1,61	1	
Q96EB6	NAD-dependent deacetylase sirtuin-1 OS=Homo sapiens GN=SIRT1 PE=1 SV=2 - [SIRT1_HUMAN]	1,61	1	
Q8IXT5	RNA-binding protein 12B OS=Homo sapiens GN=RBM12B PE=1 SV=2 - [RB12B_HUMAN]	1,60	1	
Q9ULG6	Cell cycle progression protein 1 OS=Homo sapiens GN=CCPG1 PE=1 SV=3 - [CCPG1_HUMAN]	1,59	1	0,852
P19823	Inter-alpha-trypsin inhibitor heavy chain H2 OS=Homo sapiens GN=ITIH2 PE=1 SV=2 - [ITIH2_HUMAN]	1,59	1	1,297
Q9BVQ7	Spermatogenesis-associated protein 5-like protein 1 OS=Homo sapiens GN=SPATA5L1 PE=1 SV=2 - [SPA5L_HUMAN]	1,59	1	1,069
Q96JC1	Vam6/Vps39-like protein OS=Homo sapiens GN=VPS39 PE=1 SV=2 - [VPS39_HUMAN]	1,58	1	0,903
Q68DH5	LMBR1 domain-containing protein 2 OS=Homo sapiens GN=LMBRD2 PE=1 SV=1 - [LMBD2_HUMAN]	1,58	1	1,196
Q02040	A-kinase anchor protein 17A OS=Homo sapiens GN=AKAP17A PE=1 SV=2 - [AK17A_HUMAN]	1,58	1	
P10636	Microtubule-associated protein tau OS=Homo sapiens GN=MAPT PE=1 SV=5 - [TAU_HUMAN]	1,58	1	
Q567U6	Coiled-coil domain-containing protein 93 OS=Homo sapiens GN=CCDC93 PE=1 SV=2 - [CCD93_HUMAN]	1,58	1	
J3KN33	Centlein OS=Homo sapiens GN=CNTLN PE=4 SV=1 - [J3KN33_HUMAN]	1,57	1	
O00192	Armadillo repeat protein deleted in velo-cardio-facial syndrome OS=Homo sapiens GN=ARVCF PE=1 SV=1 - [ARVC_HUMAN]	1,56	1	0,962
Q13163	Dual specificity mitogen-activated protein kinase kinase 5 OS=Homo sapiens GN=MAP2K5 PE=1 SV=2 - [MP2K5_HUMAN]	1,56	1	
P13569	Cystic fibrosis transmembrane conductance regulator OS=Homo sapiens GN=CFTR PE=1 SV=3 - [CFTR_HUMAN]	1,55	1	
O43699	Sialic acid-binding Ig-like lectin 6 OS=Homo sapiens GN=SIGLEC6 PE=1 SV=2 - [SIGL6_HUMAN]	1,55	1	
Q6NSJ0	Uncharacterized family 31 glucosidase KIAA1161 OS=Homo sapiens GN=KIAA1161 PE=1 SV=2 - [K1161_HUMAN]	1,54	1	
Q6PCB5	Round spermatid basic protein 1-like protein OS=Homo sapiens GN=RSBN1L PE=1 SV=2 - [RSBNL_HUMAN]	1,54	1	
O95834	Echinoderm microtubule-associated protein-like 2 OS=Homo sapiens GN=EML2 PE=2 SV=1 - [EMAL2_HUMAN]	1,54	1	
Q6XYQ8	Synaptotagmin-10 OS=Homo sapiens GN=SYT10 PE=2 SV=1 - [SYT10_HUMAN]	1,53	1	1,387
G3V2M6	Signal transducer and activator of transcription 2 OS=Homo sapiens GN=STAT2 PE=4 SV=1 - [G3V2M6_HUMAN]	1,53	1	0,836
Q96MM7-2	Isoform 2 of Heparan-sulfate 6-O-sulfotransferase 2 OS=Homo sapiens GN=HS6ST2 - [H6ST2_HUMAN]	1,53	1	
Q9UDT6	CAP-Gly domain-containing linker protein 2 OS=Homo sapiens GN=CLIP2 PE=1 SV=1 - [CLIP2_HUMAN]	1,53	1	
Q96RL1	BRCA1-A complex subunit RAP80 OS=Homo sapiens GN=UIMC1 PE=1 SV=2 - [UIMC1_HUMAN]	1,53	1	
H7C5R1	Ceruloplasmin (Fragment) OS=Homo sapiens GN=CP PE=4 SV=1 - [H7C5R1_HUMAN]	1,53	1	
P32455	Interferon-induced guanylate-binding protein 1 OS=Homo sapiens GN=GBP1 PE=1 SV=2 - [GBP1_HUMAN]	1,52	1	0,751
Q8NE62	Choline dehydrogenase, mitochondrial OS=Homo sapiens GN=CHDH PE=2 SV=2 - [CHDH_HUMAN]	1,52	1	1,265
Q96T83	Sodium/hydrogen exchanger 7 OS=Homo sapiens GN=SLC9A7 PE=1 SV=1 - [SL9A7_HUMAN]	1,52	1	0,919
Q99856	AT-rich interactive domain-containing protein 3A OS=Homo sapiens GN=ARID3A PE=1 SV=2 - [ARI3A_HUMAN]	1,52	1	
P16066	Atrial natriuretic peptide receptor 1 OS=Homo sapiens GN=NPR1 PE=1 SV=1 - [ANPRA_HUMAN]	1,51	1	0,908
Q96MT1	RING finger protein 145 OS=Homo sapiens GN=RNF145 PE=2 SV=2 - [RN145_HUMAN]	1,51	1	0,619
P01008	Antithrombin-III OS=Homo sapiens GN=SERPINC1 PE=1 SV=1 - [ANT3_HUMAN]	1,51	1	
Q8N5C6	S1 RNA-binding domain-containing protein 1 OS=Homo sapiens GN=SRBD1 PE=1 SV=2 - [SRBD1_HUMAN]	1,51	1	
P02462	Collagen alpha-1(IV) chain OS=Homo sapiens GN=COL4A1 PE=1 SV=3 - [CO4A1_HUMAN]	1,50	1	1,571
Q5T925	Novel protein (KIAA0894) OS=Homo sapiens GN=RP11-476E15.3 PE=4 SV=1 - [Q5T925_HUMAN]	1,50	1	0,580
Q86YS3-2	Isoform 2 of Rab11 family-interacting protein 4 OS=Homo sapiens GN=RAB11FIP4 - [RFIP4_HUMAN]	1,50	1	1,170
Q96JF0-2	Isoform 2 of Beta-galactoside alpha-2,6-sialyltransferase 2 OS=Homo sapiens GN=ST6GAL2 - [SIAT2_HUMAN]	1,50	1	0,871
O60245	Protocadherin-7 OS=Homo sapiens GN=PCDH7 PE=1 SV=2 - [PCDH7_HUMAN]	1,50	1	
O14958	Calsequestrin-2 OS=Homo sapiens GN=CASQ2 PE=1 SV=2 - [CASQ2_HUMAN]	1,50	1	
Q86XM0	Transmembrane protein 146 OS=Homo sapiens GN=TMEM146 PE=2 SV=3 - [TM146_HUMAN]	1,50	1	
Q96GP6	Scavenger receptor class F member 2 OS=Homo sapiens GN=SCARF2 PE=1 SV=4 - [SREC2_HUMAN]	1,49	1	
H0Y3Q9	Protein FAM214A (Fragment) OS=Homo sapiens GN=FAM214A PE=4 SV=2 - [H0Y3Q9_HUMAN]	1,49	1	
B7WP74	Pre-mRNA-splicing factor CWC22 homolog (Fragment) OS=Homo sapiens GN=CWC22 PE=4 SV=1 - [B7WP74_HUMAN]	1,48	1	0,922
F5H402	Aryl hydrocarbon receptor nuclear translocator-like protein 2 OS=Homo sapiens GN=ARNTL2 PE=4 SV=1 - [F5H402_HUMAN]	1,48	1	1,754
P02774	Vitamin D-binding protein OS=Homo sapiens GN=GC PE=1 SV=1 - [VTDB_HUMAN]	1,48	1	
F5H4S9	Tight junction protein ZO-3 OS=Homo sapiens GN=TJP3 PE=4 SV=1 - [F5H4S9_HUMAN]	1,47	1	1,017
Q96SB8	Structural maintenance of chromosomes protein 6 OS=Homo sapiens GN=SMC6 PE=1 SV=2 - [SMC6_HUMAN]	1,47	1	
Q8WV93	Lactation elevated protein 1 OS=Homo sapiens GN=LACE1 PE=2 SV=2 - [LACE1_HUMAN]	1,46	1	
Q8IYH5-4	Isoform 4 of ZZ-type zinc finger-containing protein 3 OS=Homo sapiens GN=ZZZ3 - [ZZZ3_HUMAN]	1,46	1	
Q96PE2	Rho guanine nucleotide exchange factor 17 OS=Homo sapiens GN=ARHGEF17 PE=1 SV=1 - [ARHGH_HUMAN]	1,45	1	
D6REY2	Colorectal mutant cancer protein OS=Homo sapiens GN=MCC PE=4 SV=1 - [D6REY2_HUMAN]	1,44	1	0,541
Q6PJI9	WD repeat-containing protein 59 OS=Homo sapiens GN=WDR59 PE=1 SV=2 - [WDR59_HUMAN]	1,44	1	
Q8WZA9	Immunity-related GTPase family Q protein OS=Homo sapiens GN=IRGQ PE=1 SV=1 - [IRGQ_HUMAN]	1,44	1	
Q12774	Rho guanine nucleotide exchange factor 5 OS=Homo sapiens GN=ARHGEF5 PE=1 SV=3 - [ARHG5_HUMAN]	1,44	1	
Q9H2F5	Enhancer of polycomb homolog 1 OS=Homo sapiens GN=EPC1 PE=1 SV=1 - [EPC1_HUMAN]	1,44	1	
A6NC98-5	Isoform 5 of Coiled-coil domain-containing protein 88B OS=Homo sapiens GN=CCDC88B - [CC88B_HUMAN]	1,44	1	
P42226	Signal transducer and activator of transcription 6 OS=Homo sapiens GN=STAT6 PE=1 SV=1 - [STAT6_HUMAN]	1,42	1	0,688
Q14153	Protein FAM53B OS=Homo sapiens GN=FAM53B PE=1 SV=2 - [FA53B_HUMAN]	1,42	1	
O14523	C2 domain-containing protein 2-like OS=Homo sapiens GN=C2CD2L PE=1 SV=3 - [C2C2L_HUMAN]	1,42	1	
Q8WWZ7-2	Isoform 2 of ATP-binding cassette sub-family A member 5 OS=Homo sapiens GN=ABCA5 - [ABCA5_HUMAN]	1,41	1	2,056
Q9UMF0	Intercellular adhesion molecule 5 OS=Homo sapiens GN=ICAM5 PE=1 SV=3 - [ICAM5_HUMAN]	1,41	1	
P20585	DNA mismatch repair protein Msh3 OS=Homo sapiens GN=MSH3 PE=1 SV=4 - [MSH3_HUMAN]	1,41	1	
F8VXI9	ARF GTPase-activating protein GIT2 OS=Homo sapiens GN=GIT2 PE=4 SV=1 - [F8VXI9_HUMAN]	1,41	1	
Q9UJF2	Ras GTPase-activating protein nGAP OS=Homo sapiens GN=RASAL2 PE=1 SV=2 - [NGAP_HUMAN]	1,40	1	0,520
Q9Y6Y8	SEC23-interacting protein OS=Homo sapiens GN=SEC23IP PE=1 SV=1 - [S23IP_HUMAN]	1,40	1	1,117
Q12933	TNF receptor-associated factor 2 OS=Homo sapiens GN=TRAF2 PE=1 SV=2 - [TRAF2_HUMAN]	1,40	1	1,073
Q15399	Toll-like receptor 1 OS=Homo sapiens GN=TLR1 PE=1 SV=3 - [TLR1_HUMAN]	1,40	1	
Q9P260	LisH domain and HEAT repeat-containing protein KIAA1468 OS=Homo sapiens GN=KIAA1468 PE=1 SV=2 - [K1468_HUMAN]	1,40	1	
O95359	Transforming acidic coiled-coil-containing protein 2 OS=Homo sapiens GN=TACC2 PE=1 SV=3 - [TACC2_HUMAN]	1,39	1	0,958
Q9NVR5-2	Isoform 2 of Protein kintoun OS=Homo sapiens GN=DNAAF2 - [KTU_HUMAN]	1,39	1	
Q58DX5	Inactive N-acetylated-alpha-linked acidic dipeptidase-like protein 2 OS=Homo sapiens GN=NAALADL2 PE=1 SV=3 - [NADL2_HUMAN]	1,38	1	0,321
O00418	Eukaryotic elongation factor 2 kinase OS=Homo sapiens GN=EEF2K PE=1 SV=2 - [EF2K_HUMAN]	1,38	1	1,363
O43156	TELO2-interacting protein 1 homolog OS=Homo sapiens GN=TTI1 PE=1 SV=3 - [TTI1_HUMAN]	1,38	1	1,987
P24386	Rab proteins geranylgeranyltransferase component A 1 OS=Homo sapiens GN=CHM PE=1 SV=3 - [RAE1_HUMAN]	1,38	1	
Q9Y5I1	Protocadherin alpha-11 OS=Homo sapiens GN=PCDHA11 PE=2 SV=1 - [PCDAB_HUMAN]	1,37	1	

Q9C0B7	Transmembrane and coiled-coil domain-containing protein 7 OS=Homo sapiens GN=TMCO7 PE=2 SV=2 - [TMCO7_HUMAN]	1,37	1		[illegible]
Q8N1G0	Zinc finger protein 687 OS=Homo sapiens GN=ZNF687 PE=1 SV=1 - [ZN687_HUMAN]	1,37	1		[illegible]
O95486	Protein transport protein Sec24A OS=Homo sapiens GN=SEC24A PE=1 SV=2 - [SC24A_HUMAN]	1,37	1		[illegible]
Q8IU81	Interferon regulatory factor 2-binding protein 1 OS=Homo sapiens GN=IRF2BP1 PE=1 SV=1 - [I2BP1_HUMAN]	1,37	1		[illegible]
Q9UID3-2	Isoform 2 of Vacuolar protein sorting-associated protein 51 homolog OS=Homo sapiens GN=VPS51 - [VPS51_HUMAN]	1,37	1		[illegible]
Q6ZSZ5	Rho guanine nucleotide exchange factor 18 OS=Homo sapiens GN=ARHGEF18 PE=1 SV=3 - [ARHGI_HUMAN]	1,36	1		[illegible]
Q494U1	Pleckstrin homology domain-containing family N member 1 OS=Homo sapiens GN=PLEKHN1 PE=1 SV=2 - [PKHN1_HUMAN]	1,36	1		[illegible]
O43896	Kinesin-like protein KIF1C OS=Homo sapiens GN=KIF1C PE=1 SV=3 - [KIF1C_HUMAN]	1,36	1		[illegible]
P50443	Sulfate transporter OS=Homo sapiens GN=SLC26A2 PE=1 SV=2 - [S26A2_HUMAN]	1,35	1	1,611	[illegible]
O75170	Serine/threonine-protein phosphatase 6 regulatory subunit 2 OS=Homo sapiens GN=PPP6R2 PE=1 SV=2 - [PP6R2_HUMAN]	1,35	1		[illegible]
Q9BQS8	FYVE and coiled-coil domain-containing protein 1 OS=Homo sapiens GN=FYCO1 PE=1 SV=3 - [FYCO1_HUMAN]	1,35	1		[illegible]
P02788-2	Isoform DeltaLf of Lactotransferrin OS=Homo sapiens GN=LTF - [TRFL_HUMAN]	1,35	1		[illegible]
O94804	Serine/threonine-protein kinase 10 OS=Homo sapiens GN=STK10 PE=1 SV=1 - [STK10_HUMAN]	1,34	1	1,026	[illegible]
O75113	NEDD4-binding protein 1 OS=Homo sapiens GN=N4BP1 PE=1 SV=4 - [N4BP1_HUMAN]	1,34	1	0,686	[illegible]
P07199	Major centromere autoantigen B OS=Homo sapiens GN=CENPB PE=1 SV=2 - [CENPB_HUMAN]	1,34	1		[illegible]
P04003	C4b-binding protein alpha chain OS=Homo sapiens GN=C4BPA PE=1 SV=2 - [C4BPA_HUMAN]	1,34	1		[illegible]
Q9UFH2-3	Isoform 3 of Dynein heavy chain 17, axonemal OS=Homo sapiens GN=DNAH17 - [DYH17_HUMAN]	1,34	1		[illegible]
Q6VMQ6	Activating transcription factor 7-interacting protein 1 OS=Homo sapiens GN=ATF7IP PE=1 SV=3 - [MCAF1_HUMAN]	1,34	1		[illegible]
A8MVW0	Protein FAM171A2 OS=Homo sapiens GN=FAM171A2 PE=1 SV=1 - [F1712_HUMAN]	1,33	1	1,911	[illegible]
Q12770	Sterol regulatory element-binding protein cleavage-activating protein OS=Homo sapiens GN=SCAP PE=1 SV=4 - [SCAP_HUMAN]	1,33	1		[illegible]
O95625	Zinc finger and BTB domain-containing protein 11 OS=Homo sapiens GN=ZBTB11 PE=1 SV=2 - [ZBT11_HUMAN]	1,33	1		[illegible]
P10398	Serine/threonine-protein kinase A-Raf OS=Homo sapiens GN=ARAF PE=1 SV=2 - [ARAF_HUMAN]	1,32	1		[illegible]
Q9UPP1	Histone lysine demethylase PHF8 OS=Homo sapiens GN=PHF8 PE=1 SV=3 - [PHF8_HUMAN]	1,32	1		[illegible]
O94762	ATP-dependent DNA helicase Q5 OS=Homo sapiens GN=RECQL5 PE=1 SV=2 - [RECQ5_HUMAN]	1,31	1		[illegible]
Q5T5C0	Syntaxin-binding protein 5 OS=Homo sapiens GN=STXBP5 PE=1 SV=1 - [STXB5_HUMAN]	1,30	1	0,780	[illegible]
Q9C0E2	Exportin-4 OS=Homo sapiens GN=XPO4 PE=1 SV=2 - [XPO4_HUMAN]	1,30	1		[illegible]
O95786	Probable ATP-dependent RNA helicase DDX58 OS=Homo sapiens GN=DDX58 PE=1 SV=2 - [DDX58_HUMAN]	1,30	1		[illegible]
Q7Z589-2	Isoform 2 of Protein EMSY OS=Homo sapiens GN=EMSY - [EMSY_HUMAN]	1,30	1		[illegible]
Q96RU3	Formin-binding protein 1 OS=Homo sapiens GN=FNBP1 PE=1 SV=2 - [FNBP1_HUMAN]	1,30	1		[illegible]
P35869	Aryl hydrocarbon receptor OS=Homo sapiens GN=AHR PE=1 SV=2 - [AHR_HUMAN]	1,30	1		[illegible]
O95427	GPI ethanolamine phosphate transferase 1 OS=Homo sapiens GN=PIGN PE=1 SV=1 - [PIGN_HUMAN]	1,29	1	1,316	[illegible]
Q15052	Rho guanine nucleotide exchange factor 6 OS=Homo sapiens GN=ARHGEF6 PE=1 SV=2 - [ARHG6_HUMAN]	1,29	1	1,308	[illegible]
H0YGW5	Low-density lipoprotein receptor-related protein 6 (Fragment) OS=Homo sapiens GN=LRP6 PE=4 SV=1 - [H0YGW5_HUMAN]	1,29	1	1,119	[illegible]
O43306	Adenylate cyclase type 6 OS=Homo sapiens GN=ADCY6 PE=1 SV=2 - [ADCY6_HUMAN]	1,28	1	0,892	[illegible]
E7EV07	Rho guanine nucleotide exchange factor 4 OS=Homo sapiens GN=ARHGEF4 PE=4 SV=1 - [E7EV07_HUMAN]	1,28	1	0,567	[illegible]
Q8TDY8	Immunoglobulin superfamily DCC subclass member 4 OS=Homo sapiens GN=IGDCC4 PE=2 SV=1 - [IGDC4_HUMAN]	1,28	1		[illegible]
E7ETZ8	Microtubule-associated protein 9 OS=Homo sapiens GN=MAP9 PE=4 SV=1 - [E7ETZ8_HUMAN]	1,28	1		[illegible]
Q5VTL8	Pre-mRNA-splicing factor 38B OS=Homo sapiens GN=PRPF38B PE=1 SV=1 - [PR38B_HUMAN]	1,28	1		[illegible]
Q08AM6	Protein VAC14 homolog OS=Homo sapiens GN=VAC14 PE=1 SV=1 - [VAC14_HUMAN]	1,28	1		[illegible]
G3V506	Protein KIAA0317 (Fragment) OS=Homo sapiens GN=KIAA0317 PE=4 SV=1 - [G3V506_HUMAN]	1,27	1	0,846	[illegible]
P12109	Collagen alpha-1(VI) chain OS=Homo sapiens GN=COL6A1 PE=1 SV=3 - [CO6A1_HUMAN]	1,26	1	1,029	[illegible]
Q9HD67	Unconventional myosin-X OS=Homo sapiens GN=MYO10 PE=1 SV=3 - [MYO10_HUMAN]	1,26	1		[illegible]
Q9ULP9	TBC1 domain family member 24 OS=Homo sapiens GN=TBC1D24 PE=1 SV=2 - [TBC24_HUMAN]	1,25	1	0,916	[illegible]
Q9UBT7-2	Isoform 2 of Alpha-catulin OS=Homo sapiens GN=CTNNAL1 - [CTNL1_HUMAN]	1,25	1	1,028	[illegible]
P78332	RNA-binding protein 6 OS=Homo sapiens GN=RBM6 PE=1 SV=5 - [RBM6_HUMAN]	1,25	1		[illegible]
Q9BPU6	Dihydropyrimidinase-related protein 5 OS=Homo sapiens GN=DPYSL5 PE=1 SV=1 - [DPYL5_HUMAN]	1,24	1	1,292	[illegible]
F5GZ53	T-lymphoma invasion and metastasis-inducing protein 1 OS=Homo sapiens GN=TIAM1 PE=4 SV=1 - [F5GZ53_HUMAN]	1,24	1	0,377	[illegible]
Q9H0E3	Histone deacetylase complex subunit SAP130 OS=Homo sapiens GN=SAP130 PE=1 SV=1 - [SP130_HUMAN]	1,24	1		[illegible]
E9PCU0	Collagen alpha-1(XI) chain OS=Homo sapiens GN=COL11A1 PE=4 SV=1 - [E9PCU0_HUMAN]	1,24	1		[illegible]
O60244	Mediator of RNA polymerase II transcription subunit 14 OS=Homo sapiens GN=MED14 PE=1 SV=2 - [MED14_HUMAN]	1,24	1		[illegible]
Q9NPI1	Bromodomain-containing protein 7 OS=Homo sapiens GN=BRD7 PE=1 SV=1 - [BRD7_HUMAN]	1,23	1		[illegible]
I3L2K5	Zinc finger CCCH domain-containing protein 7A (Fragment) OS=Homo sapiens GN=ZC3H7A PE=4 SV=1 - [I3L2K5_HUMAN]	1,23	1		[illegible]
A6ND36	Protein FAM83G OS=Homo sapiens GN=FAM83G PE=1 SV=2 - [FA83G_HUMAN]	1,22	1	1,685	[illegible]
Q9H0R1	MHD domain-containing death-inducing protein OS=Homo sapiens GN=MUDENG PE=2 SV=2 - [MUDEN_HUMAN]	1,22	1		[illegible]
O14802	DNA-directed RNA polymerase III subunit RPC1 OS=Homo sapiens GN=POLR3A PE=1 SV=2 - [RPC1_HUMAN]	1,22	1		[illegible]
Q9Y219	Protein jagged-2 OS=Homo sapiens GN=JAG2 PE=1 SV=3 - [JAG2_HUMAN]	1,21	1	1,339	[illegible]
O60658	High affinity cAMP-specific and IBMX-insensitive 3',5'-cyclic phosphodiesterase 8A OS=Homo sapiens GN=PDE8A PE=1 SV=2 - [PDE8A_	1,21	1		[illegible]
Q13393	Phospholipase D1 OS=Homo sapiens GN=PLD1 PE=1 SV=1 - [PLD1_HUMAN]	1,21	1		[illegible]
O14529	Homeobox protein cut-like 2 OS=Homo sapiens GN=CUX2 PE=1 SV=4 - [CUX2_HUMAN]	1,21	1		[illegible]
Q8WU17	E3 ubiquitin-protein ligase RNF139 OS=Homo sapiens GN=RNF139 PE=1 SV=1 - [RN139_HUMAN]	1,20	1	1,044	[illegible]
Q9UKY4	Protein O-mannosyl-transferase 2 OS=Homo sapiens GN=POMT2 PE=1 SV=2 - [POMT2_HUMAN]	1,20	1		[illegible]
O75175	CCR4-NOT transcription complex subunit 3 OS=Homo sapiens GN=CNOT3 PE=1 SV=1 - [CNOT3_HUMAN]	1,20	1		[illegible]
Q09472	Histone acetyltransferase p300 OS=Homo sapiens GN=EP300 PE=1 SV=2 - [EP300_HUMAN]	1,20	1		[illegible]
D8MCT0	Leucine-rich repeat-containing G protein-coupled receptor 5 OS=Homo sapiens GN=LGR5 PE=2 SV=1 - [D8MCT0_HUMAN]	1,20	1		[illegible]
O43719	HIV Tat-specific factor 1 OS=Homo sapiens GN=HTATSF1 PE=1 SV=1 - [HTSF1_HUMAN]	1,19	1		[illegible]
O15375	Monocarboxylate transporter 6 OS=Homo sapiens GN=SLC16A5 PE=2 SV=1 - [MOT6_HUMAN]	1,19	1		[illegible]
E7ETZ7	Histone-lysine N-methyltransferase MLL5 OS=Homo sapiens GN=MLL5 PE=4 SV=2 - [E7ETZ7_HUMAN]	1,19	1		[illegible]
P00751	Complement factor B OS=Homo sapiens GN=CFB PE=1 SV=2 - [CFAB_HUMAN]	1,18	1	0,590	[illegible]
O75197	Low-density lipoprotein receptor-related protein 5 OS=Homo sapiens GN=LRP5 PE=1 SV=2 - [LRP5_HUMAN]	1,18	1		[illegible]
H0YJ14	Fanconi anemia group M protein (Fragment) OS=Homo sapiens GN=FANCM PE=4 SV=1 - [H0YJ14_HUMAN]	1,18	1		[illegible]
Q9H8M2	Bromodomain-containing protein 9 OS=Homo sapiens GN=BRD9 PE=1 SV=2 - [BRD9_HUMAN]	1,17	1	1,567	[illegible]
Q96P48	Arf-GAP with Rho-GAP domain, ANK repeat and PH domain-containing protein 1 OS=Homo sapiens GN=ARAP1 PE=1 SV=3 - [ARAP1_HU	1,17	1		[illegible]
O15042	U2 snRNP-associated SURP motif-containing protein OS=Homo sapiens GN=U2SURP PE=1 SV=2 - [SR140_HUMAN]	1,17	1		[illegible]
Q15047	Histone-lysine N-methyltransferase SETDB1 OS=Homo sapiens GN=SETDB1 PE=1 SV=1 - [SETB1_HUMAN]	1,16	1		[illegible]
P30414	NK-tumor recognition protein OS=Homo sapiens GN=NKTR PE=1 SV=2 - [NKTR_HUMAN]	1,16	1		[illegible]
Q8NCW6	Polypeptide N-acetylgalactosaminyltransferase 11 OS=Homo sapiens GN=GALNT11 PE=2 SV=2 - [GLT11_HUMAN]	1,15	1		[illegible]
Q494V2	Coiled-coil domain-containing protein 37 OS=Homo sapiens GN=CCDC37 PE=1 SV=1 - [CCD37_HUMAN]	1,15	1		[illegible]
P15144	Aminopeptidase N OS=Homo sapiens GN=ANPEP PE=1 SV=4 - [AMPN_HUMAN]	1,14	1	0,852	[illegible]
Q5SVZ6	Zinc finger MYM-type protein 1 OS=Homo sapiens GN=ZMYM1 PE=1 SV=1 - [ZMYM1_HUMAN]	1,14	1	0,475	[illegible]
A6NI28	Rho GTPase-activating protein 42 OS=Homo sapiens GN=ARHGAP42 PE=1 SV=3 - [RHG42_HUMAN]	1,14	1		[illegible]
O94829	Importin-13 OS=Homo sapiens GN=IPO13 PE=1 SV=3 - [IPO13_HUMAN]	1,14	1		[illegible]
Q8NCN5	Pyruvate dehydrogenase phosphatase regulatory subunit, mitochondrial OS=Homo sapiens GN=PDPR PE=1 SV=2 - [PDPR_HUMAN]	1,14	1		[illegible]
Q9H0E9	Bromodomain-containing protein 8 OS=Homo sapiens GN=BRD8 PE=1 SV=2 - [BRD8_HUMAN]	1,13	1		[illegible]
J3KNJ3	N-acetylated-alpha-linked acidic dipeptidase 2 OS=Homo sapiens GN=NAALAD2 PE=4 SV=1 - [J3KNJ3_HUMAN]	1,13	1		[illegible]
O94880	PHD finger protein 14 OS=Homo sapiens GN=PHF14 PE=1 SV=2 - [PHF14_HUMAN]	1,13	1		[illegible]
Q8TD19	Serine/threonine-protein kinase Nek9 OS=Homo sapiens GN=NEK9 PE=1 SV=2 - [NEK9_HUMAN]	1,12	1		[illegible]
Q9Y2X9	Zinc finger protein 281 OS=Homo sapiens GN=ZNF281 PE=1 SV=1 - [ZN281_HUMAN]	1,12	1		[illegible]
P22670	MHC class II regulatory factor RFX1 OS=Homo sapiens GN=RFX1 PE=1 SV=2 - [RFX1_HUMAN]	1,12	1		[illegible]
Q7Z3E2	Uncharacterized protein C10orf118 OS=Homo sapiens GN=C10orf118 PE=2 SV=2 - [CJ118_HUMAN]	1,11	1	0,972	[illegible]
C9JR62	Coiled-coil domain-containing protein 141 (Fragment) OS=Homo sapiens GN=CCDC141 PE=4 SV=1 - [C9JR62_HUMAN]	1,11	1		[illegible]
Q8IYI6	Exocyst complex component 8 OS=Homo sapiens GN=EXOC8 PE=1 SV=2 - [EXOC8_HUMAN]	1,10	1	1,381	[illegible]
Q9P2J2	Protein turtle homolog A OS=Homo sapiens GN=IGSF9 PE=1 SV=2 - [TUTLA_HUMAN]	1,10	1	0,791	[illegible]
Q9BTE3	Mini-chromosome maintenance complex-binding protein OS=Homo sapiens GN=MCMBP PE=1 SV=2 - [MCMBP_HUMAN]	1,09	1	0,805	[illegible]
Q9H254	Spectrin beta chain, brain 3 OS=Homo sapiens GN=SPTBN4 PE=1 SV=2 - [SPTN4_HUMAN]	1,09	1	0,946	[illegible]
Q5T5X7	BEN domain-containing protein 3 OS=Homo sapiens GN=BEND3 PE=1 SV=1 - [BEND3_HUMAN]	1,09	1		[illegible]
Q6P5W5	Zinc transporter ZIP4 OS=Homo sapiens GN=SLC39A4 PE=1 SV=3 - [S39A4_HUMAN]	1,08	1		[illegible]
Q5T1M5	FK506-binding protein 15 OS=Homo sapiens GN=FKBP15 PE=1 SV=2 - [FKB15_HUMAN]	1,07	1	1,190	[illegible]
Q9BVA0	Katanin p80 WD40-containing subunit B1 OS=Homo sapiens GN=KATNB1 PE=1 SV=1 - [KTNB1_HUMAN]	1,07	1		[illegible]
Q9H270	Vacuolar protein sorting-associated protein 11 homolog OS=Homo sapiens GN=VPS11 PE=1 SV=1 - [VPS11_HUMAN]	1,06	1	0,833	[illegible]
Q6ZTR5-2	Isoform 2 of Uncharacterized protein CXorf22 OS=Homo sapiens GN=CXorf22 - [CX022_HUMAN]	1,06	1	1,610	[illegible]
Q9BV73	Centrosome-associated protein CEP250 OS=Homo sapiens GN=CEP250 PE=1 SV=2 - [CP250_HUMAN]	1,06	1		[illegible]
Q5TCX8	Mitogen-activated protein kinase kinase kinase MLK4 OS=Homo sapiens GN=MLK4 PE=1 SV=1 - [M3KL4_HUMAN]	1,06	1		[illegible]
Q8N0X7	Spartin OS=Homo sapiens GN=SPG20 PE=1 SV=1 - [SPG20_HUMAN]	1,05	1		[illegible]
Q9UPN4-3	Isoform 3 of 5-azacytidine-induced protein 1 OS=Homo sapiens GN=AZI1 - [AZI1_HUMAN]	1,05	1		[illegible]
Q8WVC0	RNA polymerase-associated protein LEO1 OS=Homo sapiens GN=LEO1 PE=1 SV=1 - [LEO1_HUMAN]	1,05	1		[illegible]
Q96DR7	Rho guanine nucleotide exchange factor 26 OS=Homo sapiens GN=ARHGEF26 PE=1 SV=4 - [ARHGQ_HUMAN]	1,03	1	0,868	[illegible]
O95980	Reversion-inducing cysteine-rich protein with Kazal motifs OS=Homo sapiens GN=RECK PE=1 SV=1 - [RECK_HUMAN]	1,03	1	1,242	[illegible]
Q9NYY3	Serine/threonine-protein kinase PLK2 OS=Homo sapiens GN=PLK2 PE=1 SV=3 - [PLK2_HUMAN]	1,02	1	**2,067**	[illegible]
Q15147	1-phosphatidylinositol-4,5-bisphosphate phosphodiesterase beta-4 OS=Homo sapiens GN=PLCB4 PE=1 SV=3 - [PLCB4_HUMAN]	1,02	1	1,244	[illegible]
Q9UPN6	Protein SCAF8 OS=Homo sapiens GN=SCAF8 PE=1 SV=1 - [SCAF8_HUMAN]	1,02	1		[illegible]
E7EP70	Breast cancer type 1 susceptibility protein OS=Homo sapiens GN=BRCA1 PE=4 SV=1 - [E7EP70_HUMAN]	1,02	1		[illegible]
Q96EN8	Molybdenum cofactor sulfurase OS=Homo sapiens GN=MOCOS PE=1 SV=2 - [MOCOS_HUMAN]	1,01	1	1,171	[illegible]
Q5VXJ5	Synaptonemal complex protein 1 (Fragment) OS=Homo sapiens GN=SYCP1 PE=2 SV=1 - [Q5VXJ5_HUMAN]	1,01	1		[illegible]
Q9UGR2	Zinc finger CCCH domain-containing protein 7B OS=Homo sapiens GN=ZC3H7B PE=1 SV=1 - [Z3H7B_HUMAN]	1,01	1		[illegible]
P16452	Erythrocyte membrane protein band 4.2 OS=Homo sapiens GN=EPB42 PE=1 SV=3 - [EPB42_HUMAN]	1,01	1		[illegible]
O75448	Mediator of RNA polymerase II transcription subunit 24 OS=Homo sapiens GN=MED24 PE=1 SV=1 - [MED24_HUMAN]	1,01	1		[illegible]
Q00653	Nuclear factor NF-kappa-B p100 subunit OS=Homo sapiens GN=NFKB2 PE=1 SV=4 - [NFKB2_HUMAN]	1,00	1	1,359	[illegible]
Q03468	DNA excision repair protein ERCC-6 OS=Homo sapiens GN=ERCC6 PE=1 SV=1 - [ERCC6_HUMAN]	1,00	1		[illegible]
Q9BZC7	ATP-binding cassette sub-family A member 2 OS=Homo sapiens GN=ABCA2 PE=1 SV=3 - [ABCA2_HUMAN]	0,99	1		[illegible]
Q9UM47	Neurogenic locus notch homolog protein 3 OS=Homo sapiens GN=NOTCH3 PE=1 SV=2 - [NOTC3_HUMAN]	0,99	1		[illegible]
O94832	Unconventional myosin-Id OS=Homo sapiens GN=MYO1D PE=1 SV=2 - [MYO1D_HUMAN]	0,99	1		[illegible]
P28340	DNA polymerase delta catalytic subunit OS=Homo sapiens GN=POLD1 PE=1 SV=2 - [DPOD1_HUMAN]	0,99	1		[illegible]
Q9H8V3	Protein ECT2 OS=Homo sapiens GN=ECT2 PE=1 SV=4 - [ECT2_HUMAN]	0,98	1	0,951	[illegible]
Q5T481	RNA-binding protein 20 OS=Homo sapiens GN=RBM20 PE=1 SV=3 - [RBM20_HUMAN]	0,98	1	1,005	[illegible]
Q9Y4C1	Lysine-specific demethylase 3A OS=Homo sapiens GN=KDM3A PE=1 SV=4 - [KDM3A_HUMAN]	0,98	1		[illegible]
Q5TCY1	Tau-tubulin kinase 1 OS=Homo sapiens GN=TTBK1 PE=1 SV=2 - [TTBK1_HUMAN]	0,98	1		[illegible]
Q9H9J4	Ubiquitin carboxyl-terminal hydrolase 42 OS=Homo sapiens GN=USP42 PE=1 SV=3 - [UBP42_HUMAN]	0,98	1		[illegible]
Q9NR82	Potassium voltage-gated channel subfamily KQT member 5 OS=Homo sapiens GN=KCNQ5 PE=1 SV=3 - [KCNQ5_HUMAN]	0,97	1	1,889	[illegible]
Q8IY22-3	Isoform 3 of C-Maf-inducing protein OS=Homo sapiens GN=CMIP - [CMIP_HUMAN]	0,97	1		[illegible]
Q96JJ3	Engulfment and cell motility protein 2 OS=Homo sapiens GN=ELMO2 PE=1 SV=2 - [ELMO2_HUMAN]	0,97	1		[illegible]
Q9HCM7	Fibrosin-1-like protein OS=Homo sapiens GN=FBRSL1 PE=1 SV=4 - [FBSL_HUMAN]	0,96	1	0,479	[illegible]
O15061	Synemin OS=Homo sapiens GN=SYNM PE=1 SV=2 - [SYNEM_HUMAN]	0,96	1		[illegible]
Q5SXM2	snRNA-activating protein complex subunit 4 OS=Homo sapiens GN=SNAPC4 PE=1 SV=1 - [SNPC4_HUMAN]	0,95	1		[illegible]
O75128	Protein cordon-bleu OS=Homo sapiens GN=COBL PE=1 SV=2 - [COBL_HUMAN]	0,95	1		[illegible]
Q9UHP3	Ubiquitin carboxyl-terminal hydrolase 25 OS=Homo sapiens GN=USP25 PE=1 SV=4 - [UBP25_HUMAN]	0,95	1		[illegible]
Q63ZY3	KN motif and ankyrin repeat domain-containing protein 2 OS=Homo sapiens GN=KANK2 PE=1 SV=1 - [KANK2_HUMAN]	0,94	1		[illegible]
O94898	Leucine-rich repeats and immunoglobulin-like domains protein 2 OS=Homo sapiens GN=LRIG2 PE=1 SV=3 - [LRIG2_HUMAN]	0,94	1	1,096	[illegible]
Q6P158	Putative ATP-dependent RNA helicase DHX57 OS=Homo sapiens GN=DHX57 PE=1 SV=2 - [DHX57_HUMAN]	0,94	1		[illegible]
B9ZVR7	Striated muscle preferentially-expressed protein kinase OS=Homo sapiens GN=SPEG PE=4 SV=1 - [B9ZVR7_HUMAN]	0,93	1		[illegible]
Q8TF46-3	Isoform 3 of DIS3-like exonuclease 1 OS=Homo sapiens GN=DIS3L - [DI3L1_HUMAN]	0,93	1		[illegible]
O60524	Nuclear export mediator factor NEMF OS=Homo sapiens GN=NEMF PE=1 SV=4 - [NEMF_HUMAN]	0,93	1		[illegible]
Q7L590	Protein MCM10 homolog OS=Homo sapiens GN=MCM10 PE=1 SV=2 - [MCM10_HUMAN]	0,91	1	1,055	[illegible]
Q86Y13	E3 ubiquitin-protein ligase DZIP3 OS=Homo sapiens GN=DZIP3 PE=1 SV=2 - [DZIP3_HUMAN]	0,91	1	1,083	[illegible]
Q7Z7A1	Centriolin OS=Homo sapiens GN=CNTRL PE=1 SV=2 - [CNTRL_HUMAN]	0,90	1	1,319	[illegible]
E7EN23	85 kDa calcium-independent phospholipase A2 OS=Homo sapiens GN=PLA2G6 PE=4 SV=1 - [E7EN23_HUMAN]	0,90	1		[illegible]
O75891	Cytosolic 10-formyltetrahydrofolate dehydrogenase OS=Homo sapiens GN=ALDH1L1 PE=1 SV=2 - [AL1L1_HUMAN]	0,89	1		[illegible]
Q86U86	Protein polybromo-1 OS=Homo sapiens GN=PBRM1 PE=1 SV=1 - [PB1_HUMAN]	0,89	1		[illegible]
Q99590	Protein SCAF11 OS=Homo sapiens GN=SCAF11 PE=1 SV=2 - [SCAFB_HUMAN]	0,89	1		[illegible]
Q14527	Helicase-like transcription factor OS=Homo sapiens GN=HLTF PE=1 SV=2 - [HLTF_HUMAN]	0,89	1		[illegible]
Q7Z392	Trafficking protein particle complex subunit 11 OS=Homo sapiens GN=TRAPPC11 PE=1 SV=2 - [TPC11_HUMAN]	0,88	1	1,056	[illegible]
Q75V66	Anoctamin-5 OS=Homo sapiens GN=ANO5 PE=1 SV=1 - [ANO5_HUMAN]	0,88	1	0,781	[illegible]
Q9Y2L5-2	Isoform 2 of Trafficking protein particle complex subunit 8 OS=Homo sapiens GN=TRAPPC8 - [TPPC8_HUMAN]	0,88	1		[illegible]
Q15542	Transcription initiation factor TFIID subunit 5 OS=Homo sapiens GN=TAF5 PE=1 SV=3 - [TAF5_HUMAN]	0,88	1		[illegible]
Q2NKX8	DNA excision repair protein ERCC-6-like OS=Homo sapiens GN=ERCC6L PE=1 SV=1 - [ERC6L_HUMAN]	0,88	1		[illegible]
Q9H4L7	SWI/SNF-related matrix-associated actin-dependent regulator of chromatin subfamily A containing DEAD/H box 1 OS=Homo sapiens GN:	0,88	1		[illegible]
Q8IVU3	Probable E3 ubiquitin-protein ligase HERC6 OS=Homo sapiens GN=HERC6 PE=2 SV=2 - [HERC6_HUMAN]	0,88	1		[illegible]
Q99707	Methionine synthase OS=Homo sapiens GN=MTR PE=1 SV=2 - [METH_HUMAN]	0,87	1		[illegible]
Q9UGU0	Transcription factor 20 OS=Homo sapiens GN=TCF20 PE=1 SV=3 - [TCF20_HUMAN]	0,87	1		[illegible]
Q5SYE7-2	Isoform 2 of NHS-like protein 1 OS=Homo sapiens GN=NHSL1 - [NHSL1_HUMAN]	0,87	1		[illegible]
Q5TGY3	AT-hook DNA-binding motif-containing protein 1 OS=Homo sapiens GN=AHDC1 PE=1 SV=1 - [AHDC1_HUMAN]	0,87	1		[illegible]
Q9NS87	Kinesin-like protein KIF15 OS=Homo sapiens GN=KIF15 PE=1 SV=1 - [KIF15_HUMAN]	0,86	1	1,101	[illegible]
Q9UNY4	Transcription termination factor 2 OS=Homo sapiens GN=TTF2 PE=1 SV=2 - [TTF2_HUMAN]	0,86	1	1,020	[illegible]
O43933	Peroxisome biogenesis factor 1 OS=Homo sapiens GN=PEX1 PE=1 SV=1 - [PEX1_HUMAN]	0,86	1		[illegible]
Q9BZQ8	Protein Niban OS=Homo sapiens GN=FAM129A PE=1 SV=1 - [NIBAN_HUMAN]	0,86	1		[illegible]
O75385	Serine/threonine-protein kinase ULK1 OS=Homo sapiens GN=ULK1 PE=1 SV=2 - [ULK1_HUMAN]	0,86	1		[illegible]
Q3MIN7	Ral guanine nucleotide dissociation stimulator-like 3 OS=Homo sapiens GN=RGL3 PE=1 SV=2 - [RGL3_HUMAN]	0,85	1		[illegible]
Q5VST9-5	Isoform 4 of Obscurin OS=Homo sapiens GN=OBSCN - [OBSCN_HUMAN]	0,84	1		[illegible]
Q5T200	Zinc finger CCCH domain-containing protein 13 OS=Homo sapiens GN=ZC3H13 PE=1 SV=1 - [ZC3HD_HUMAN]	0,84	1		[illegible]
Q8NA31	Coiled-coil domain-containing protein 79 OS=Homo sapiens GN=CCDC79 PE=2 SV=3 - [CCD79_HUMAN]	0,83	1	1,722	[illegible]
O15069	NAC-alpha domain-containing protein 1 OS=Homo sapiens GN=NACAD PE=2 SV=3 - [NACAD_HUMAN]	0,83	1		[illegible]

Q8TDM6-2	Isoform 2 of Disks large homolog 5 OS=Homo sapiens GN=DLG5 - [DLG5_HUMAN]	0,82	1		0,870					0,799					0,715			
Q96Q06	Perilipin-4 OS=Homo sapiens GN=PLIN4 PE=2 SV=2 - [PLIN4_HUMAN]	0,81	1	0,719					0,748					0,882				
Q8N7Z5	Ankyrin repeat domain-containing protein 31 OS=Homo sapiens GN=ANKRD31 PE=5 SV=2 - [ANR31_HUMAN]	0,80	1	1,304	1,125				0,835	0,570				3,863	2,922			
P06681	Complement C2 OS=Homo sapiens GN=C2 PE=1 SV=2 - [CO2_HUMAN]	0,80	1		0,765					1,835					2,602			
H0YMW2	A-kinase anchor protein 13 (Fragment) OS=Homo sapiens GN=AKAP13 PE=4 SV=1 - [H0YMW2_HUMAN]	0,80	1		0,843					0,814					1,063			
O43314	Inositol hexakisphosphate and diphosphoinositol-pentakisphosphate kinase 2 OS=Homo sapiens GN=PPIP5K2 PE=1 SV=3 - [VIP2_HUMAN	0,80	1				1,003	1,118				0,977	0,893				0,941	1,151
Q8NEB9	Phosphatidylinositol 3-kinase catalytic subunit type 3 OS=Homo sapiens GN=PIK3C3 PE=1 SV=1 - [PK3C3_HUMAN]	0,79	1			0,924	1,119				1,319	1,245				1,354	1,403	
Q9H9Y6	DNA-directed RNA polymerase I subunit RPA2 OS=Homo sapiens GN=POLR1B PE=1 SV=2 - [RPA2_HUMAN]	0,79	1				1,028	0,972				0,770	0,749				0,642	0,600
O94855	Protein transport protein Sec24D OS=Homo sapiens GN=SEC24D PE=1 SV=2 - [SC24D_HUMAN]	0,78	1	0,886					0,791					0,786				
Q96C45	Serine/threonine-protein kinase ULK4 OS=Homo sapiens GN=ULK4 PE=2 SV=2 - [ULK4_HUMAN]	0,78	1				1,166					1,142					1,928	
Q14159	Uncharacterized protein KIAA0146 OS=Homo sapiens GN=KIAA0146 PE=2 SV=2 - [K0146_HUMAN]	0,77	1	1,398	1,013				1,067	0,872				1,079	1,035			
Q9Y6J0	Calcineurin-binding protein cabin-1 OS=Homo sapiens GN=CABIN1 PE=1 SV=1 - [CABIN_HUMAN]	0,77	1			0,964					0,806					1,081		
Q92766	Ras-responsive element-binding protein 1 OS=Homo sapiens GN=RREB1 PE=1 SV=3 - [RREB1_HUMAN]	0,77	1			1,111					0,955					1,335		
Q9UPY3-2	Isoform 2 of Endoribonuclease Dicer OS=Homo sapiens GN=DICER1 - [DICER_HUMAN]	0,77	1					0,929					0,974					0,843
Q8TEK3	Histone-lysine N-methyltransferase, H3 lysine-79 specific OS=Homo sapiens GN=DOT1L PE=1 SV=2 - [DOT1L_HUMAN]	0,75	1			1,194	1,060				1,346	1,098				2,373	2,470	
O15056	Synaptojanin-2 OS=Homo sapiens GN=SYNJ2 PE=1 SV=3 - [SYNJ2_HUMAN]	0,74	1				1,349					1,194					1,124	
Q8IVE3	Pleckstrin homology domain-containing family H member 2 OS=Homo sapiens GN=PLEKHH2 PE=2 SV=2 - [PKHH2_HUMAN]	0,74	1					0,879					0,923					0,687
Q9P1Y6	PHD and RING finger domain-containing protein 1 OS=Homo sapiens GN=PHRF1 PE=1 SV=3 - [PHRF1_HUMAN]	0,73	1					0,937					0,733					1,023
F5H156	Armadillo repeat-containing protein 5 OS=Homo sapiens GN=ARMC5 PE=4 SV=1 - [F5H156_HUMAN]	0,72	1		1,862					2,109					3,273			
Q92547	DNA topoisomerase 2-binding protein 1 OS=Homo sapiens GN=TOPBP1 PE=1 SV=3 - [TOPB1_HUMAN]	0,72	1				0,777	0,941				0,887	1,162				0,605	0,856
Q8N283	Ankyrin repeat domain-containing protein 35 OS=Homo sapiens GN=ANKRD35 PE=2 SV=2 - [ANR35_HUMAN]	0,70	1		1,558					0,878					0,873			
Q5VWN6	Protein FAM208B OS=Homo sapiens GN=FAM208B PE=1 SV=1 - [F208B_HUMAN]	0,70	1				0,835	1,081				0,720	0,944				0,808	0,999
Q9NYI0	PH and SEC7 domain-containing protein 3 OS=Homo sapiens GN=PSD3 PE=1 SV=2 - [PSD3_HUMAN]	0,67	1	0,903	0,813				0,773	0,811				0,730	0,698			
P21359	Neurofibromin OS=Homo sapiens GN=NF1 PE=1 SV=2 - [NF1_HUMAN]	0,67	1	0,762					0,766					0,865				
Q8NB14	Ubiquitin carboxyl-terminal hydrolase 38 OS=Homo sapiens GN=USP38 PE=2 SV=2 - [UBP38_HUMAN]	0,67	1	1,699					1,717					0,819				
Q9Y2I7	1-phosphatidylinositol-3-phosphate 5-kinase OS=Homo sapiens GN=PIKFYVE PE=1 SV=3 - [FYV1_HUMAN]	0,67	1					1,324					0,851					0,941
Q8WVS4	WD repeat-containing protein 60 OS=Homo sapiens GN=WDR60 PE=1 SV=3 - [WDR60_HUMAN]	0,66	1	1,436					0,957					0,693				
Q5T5U3	Rho GTPase-activating protein 21 OS=Homo sapiens GN=ARHGAP21 PE=1 SV=1 - [RHG21_HUMAN]	0,66	1			1,324					1,020					2,706		
Q7Z460	CLIP-associating protein 1 OS=Homo sapiens GN=CLASP1 PE=1 SV=1 - [CLAP1_HUMAN]	0,65	1			1,386					1,423					1,970		
C9J815	Apolipoprotein B receptor OS=Homo sapiens GN=APOBR PE=4 SV=1 - [C9J815_HUMAN]	0,65	1				1,058					0,883					1,939	
Q96MR6	WD repeat-containing protein 65 OS=Homo sapiens GN=WDR65 PE=1 SV=3 - [WDR65_HUMAN]	0,64	1		1,278					1,296					1,826			
Q12769	Nuclear pore complex protein Nup160 OS=Homo sapiens GN=NUP160 PE=1 SV=3 - [NU160_HUMAN]	0,63	1	0,954					1,119					1,035				
Q6ZV73	FYVE, RhoGEF and PH domain-containing protein 6 OS=Homo sapiens GN=FGD6 PE=1 SV=2 - [FGD6_HUMAN]	0,63	1				1,146					1,421					3,388	
Q9UKN7	Unconventional myosin-XV OS=Homo sapiens GN=MYO15A PE=1 SV=2 - [MYO15_HUMAN]	0,62	1		1,071					1,125					0,950			
Q9UKZ4	Teneurin-1 OS=Homo sapiens GN=ODZ1 PE=1 SV=2 - [TEN1_HUMAN]	0,62	1			1,079					0,860					1,052		
Q12789-3	Isoform 2 of General transcription factor 3C polypeptide 1 OS=Homo sapiens GN=GTF3C1 - [TF3C1_HUMAN]	0,62	1				1,276					1,115					0,812	
Q5VW36	Focadhesin OS=Homo sapiens GN=FOCAD PE=1 SV=1 - [FOCAD_HUMAN]	0,61	1	0,905				0,972	0,672				1,049	0,627				0,528
Q9P2D3-3	Isoform 3 of HEAT repeat-containing protein 5B OS=Homo sapiens GN=HEATR5B - [HTR5B_HUMAN]	0,61	1	0,855					0,867					0,909				
Q9Y3R5-2	Isoform 2 of Protein dopey-2 OS=Homo sapiens GN=DOPEY2 - [DOP2_HUMAN]	0,61	1	0,584					0,593					0,749				
Q06190	Serine/threonine-protein phosphatase 2A regulatory subunit B'' subunit alpha OS=Homo sapiens GN=PPP2R3A PE=1 SV=1 - [P2R3A_HUM	0,61	1				1,446					1,184					1,025	
Q6TFL3	Uncharacterized protein C9orf93 OS=Homo sapiens GN=C9orf93 PE=2 SV=1 - [CI093_HUMAN]	0,60	1		1,073					1,048					0,970			
Q8IZY2-2	Isoform 2 of ATP-binding cassette sub-family A member 7 OS=Homo sapiens GN=ABCA7 - [ABCA7_HUMAN]	0,60	1		0,877					0,811					1,254			
O95255	Multidrug resistance-associated protein 6 OS=Homo sapiens GN=ABCC6 PE=1 SV=2 - [MRP6_HUMAN]	0,60	1					0,912					1,043					1,344
P42684	Abelson tyrosine-protein kinase 2 OS=Homo sapiens GN=ABL2 PE=1 SV=1 - [ABL2_HUMAN]	0,59	1				1,254	1,373				1,189	1,350				0,670	0,718
Q7Z3U7	Protein MON2 homolog OS=Homo sapiens GN=MON2 PE=1 SV=2 - [MON2_HUMAN]	0,58	1		0,636					0,665					0,718			
O60287	Nucleolar pre-ribosomal-associated protein 1 OS=Homo sapiens GN=URB1 PE=1 SV=4 - [NPA1P_HUMAN]	0,57	1		0,675					0,856					0,975			
P78504	Protein jagged-1 OS=Homo sapiens GN=JAG1 PE=1 SV=3 - [JAG1_HUMAN]	0,57	1		0,797					0,676					1,382			
Q5SNV9	Uncharacterized protein C1orf167 OS=Homo sapiens GN=C1orf167 PE=2 SV=2 - [CA167_HUMAN]	0,54	1		0,812	0,942	0,974	1,057		1,099	0,861	0,966	0,988		1,247	0,423	0,522	0,634
Q96BY7	Autophagy-related protein 2 homolog B OS=Homo sapiens GN=ATG2B PE=1 SV=5 - [ATG2B_HUMAN]	0,53	1				1,079					0,857					0,726	
Q66GS9	Centrosomal protein of 135 kDa OS=Homo sapiens GN=CEP135 PE=1 SV=2 - [CP135_HUMAN]	0,53	1					1,331					1,615					1,879
O14647-2	Isoform 2 of Chromodomain-helicase-DNA-binding protein 2 OS=Homo sapiens GN=CHD2 - [CHD2_HUMAN]	0,52	1	0,664					0,965					0,892				
Q5JR59	Microtubule-associated tumor suppressor candidate 2 OS=Homo sapiens GN=MTUS2 PE=1 SV=3 - [MTUS2_HUMAN]	0,51	1	1,441					1,013					0,859				
Q7Z7M0-2	Isoform 2 of Multiple epidermal growth factor-like domains protein 8 OS=Homo sapiens GN=MEGF8 - [MEGF8_HUMAN]	0,50	1	0,778					0,827					0,840				
Q9NTG1	Polycystic kidney disease and receptor for egg jelly-related protein OS=Homo sapiens GN=PKDREJ PE=2 SV=2 - [PKDRE_HUMAN]	0,49	1	0,951	**0,468**				0,625	0,532				0,978	0,749			
Q96Q15	Serine/threonine-protein kinase SMG1 OS=Homo sapiens GN=SMG1 PE=1 SV=3 - [SMG1_HUMAN]	0,49	1			1,088					1,008					1,154		
Q13439	Golgin subfamily A member 4 OS=Homo sapiens GN=GOLGA4 PE=1 SV=1 - [GOGA4_HUMAN]	0,49	1					0,753					0,946					1,002
Q9UBG0	C-type mannose receptor 2 OS=Homo sapiens GN=MRC2 PE=1 SV=2 - [MRC2_HUMAN]	0,47	1		**2,124**					1,184					1,026			
Q70CQ4	Ubiquitin carboxyl-terminal hydrolase 31 OS=Homo sapiens GN=USP31 PE=1 SV=2 - [UBP31_HUMAN]	0,44	1	0,754					0,988					1,103				
Q92817	Envoplakin OS=Homo sapiens GN=EVPL PE=1 SV=3 - [EVPL_HUMAN]	0,44	1			0,565					0,781					1,484		
P15822	Zinc finger protein 40 OS=Homo sapiens GN=HIVEP1 PE=1 SV=3 - [ZEP1_HUMAN]	0,44	1				1,248					1,425					2,271	
Q9Y4C0	Neurexin-3-alpha OS=Homo sapiens GN=NRXN3 PE=1 SV=4 - [NRX3A_HUMAN]	0,43	1	1,328					1,251					1,427				
Q13315	Serine-protein kinase ATM OS=Homo sapiens GN=ATM PE=1 SV=3 - [ATM_HUMAN]	0,43	1			1,218					1,051					0,759		
O94822	E3 ubiquitin-protein ligase listerin OS=Homo sapiens GN=LTN1 PE=1 SV=6 - [LTN1_HUMAN]	0,40	1		1,836					2,109					1,483			
Q9HCM1	Uncharacterized protein C12orf35 OS=Homo sapiens GN=C12orf35 PE=1 SV=3 - [CL035_HUMAN]	0,40	1				0,734					0,872					1,229	
Q86UK0	ATP-binding cassette sub-family A member 12 OS=Homo sapiens GN=ABCA12 PE=1 SV=3 - [ABCAC_HUMAN]	0,39	1	**0,268**					0,266					0,875				
Q6UB99	Ankyrin repeat domain-containing protein 11 OS=Homo sapiens GN=ANKRD11 PE=1 SV=3 - [ANR11_HUMAN]	0,38	1				1,012					1,176					1,610	
Q9NYC9	Dynein heavy chain 9, axonemal OS=Homo sapiens GN=DNAH9 PE=1 SV=3 - [DYH9_HUMAN]	0,33	1		0,648					0,714					0,884			
P49815	Tuberin OS=Homo sapiens GN=TSC2 PE=1 SV=2 - [TSC2_HUMAN]	0,33	1		1,423					1,267					1,441			
P20930	Filaggrin OS=Homo sapiens GN=FLG PE=1 SV=3 - [FILA_HUMAN]	0,32	1	1,842					1,916					1,463				
Q15413	Ryanodine receptor 3 OS=Homo sapiens GN=RYR3 PE=1 SV=3 - [RYR3_HUMAN]	0,31	1	1,375					1,370					1,477				
Q01484;I6L894	Ankyrin-2 OS=Homo sapiens GN=ANK2 PE=1 SV=3 - [ANK2_HUMAN]	0,31	1				1,687					0,908					0,581	
Q8NEZ4	Histone-lysine N-methyltransferase MLL3 OS=Homo sapiens GN=MLL3 PE=1 SV=3 - [MLL3_HUMAN]	0,29	1	1,017	1,113				0,834	0,827				2,275	3,374			
Q5D862	Filaggrin-2 OS=Homo sapiens GN=FLG2 PE=1 SV=1 - [FILA2_HUMAN]	0,29	1	1,518	1,066				1,460	1,178				0,969	0,972			
Q9P225	Dynein heavy chain 2, axonemal OS=Homo sapiens GN=DNAH2 PE=1 SV=3 - [DYH2_HUMAN]	0,29	1					1,084					0,821					1,150
E9PF99	Centrosomal protein of 192 kDa OS=Homo sapiens GN=CEP192 PE=4 SV=1 - [E9PF99_HUMAN]	0,28	1	0,696					0,775					0,843				
P46100	Transcriptional regulator ATRX OS=Homo sapiens GN=ATRX PE=1 SV=5 - [ATRX_HUMAN]	0,28	1				1,173					0,906					2,289	
P13611	Versican core protein OS=Homo sapiens GN=VCAN PE=1 SV=3 - [CSPG2_HUMAN]	0,24	1				1,315					1,669					0,582	
Q7Z7G8-2	Isoform 2 of Vacuolar protein sorting-associated protein 13B OS=Homo sapiens GN=VPS13B - [VP13B_HUMAN]	0,23	1	1,142					1,454					1,033				
Q5T011	Protein SZT2 OS=Homo sapiens GN=SZT2 PE=2 SV=3 - [SZT2_HUMAN]	0,17	1	0,987					0,720					0,781				
Q9NZJ4	Sacsin OS=Homo sapiens GN=SACS PE=1 SV=2 - [SACS_HUMAN]	0,15	1		1,056					1,246					1,038			
Q8WZ42-3	Isoform 3 of Titin OS=Homo sapiens GN=TTN - [TITIN_HUMAN]	0,08	1			1,261					1,257					2,659		
P62807	Histone H2B type 1-C/E/F/G/I OS=Homo sapiens GN=HIST1H2BC PE=1 SV=4 - [H2B1C_HUMAN]	76,98	0															
Q99877	Histone H2B type 1-N OS=Homo sapiens GN=HIST1H2BN PE=1 SV=3 - [H2B1N_HUMAN]	76,98	0															
P16188	HLA class I histocompatibility antigen, A-30 alpha chain OS=Homo sapiens GN=HLA-A PE=1 SV=2 - [1A30_HUMAN]	48,22	0															
P30492	HLA class I histocompatibility antigen, B-54 alpha chain OS=Homo sapiens GN=HLA-B PE=2 SV=1 - [1B54_HUMAN]	47,51	0															
P30464	HLA class I histocompatibility antigen, B-15 alpha chain OS=Homo sapiens GN=HLA-B PE=1 SV=1 - [1B15_HUMAN]	44,48	0															
P30475	HLA class I histocompatibility antigen, B-39 alpha chain OS=Homo sapiens GN=HLA-B PE=2 SV=1 - [1B39_HUMAN]	44,48	0															
P30466	HLA class I histocompatibility antigen, B-18 alpha chain OS=Homo sapiens GN=HLA-B PE=1 SV=1 - [1B18_HUMAN]	44,20	0															
P30462	HLA class I histocompatibility antigen, B-14 alpha chain OS=Homo sapiens GN=HLA-B PE=1 SV=1 - [1B14_HUMAN]	43,92	0															
P18464	HLA class I histocompatibility antigen, B-51 alpha chain OS=Homo sapiens GN=HLA-B PE=1 SV=1 - [1B51_HUMAN]	43,37	0															
P10319	HLA class I histocompatibility antigen, B-58 alpha chain OS=Homo sapiens GN=HLA-B PE=2 SV=1 - [1B58_HUMAN]	43,37	0															
O19617	HLA class I antigen OS=Homo sapiens GN=HLA-C PE=2 SV=1 - [O19617_HUMAN]	43,17	0															
P30460	HLA class I histocompatibility antigen, B-8 alpha chain OS=Homo sapiens GN=HLA-B PE=1 SV=1 - [1B08_HUMAN]	38,95	0															
P30483	HLA class I histocompatibility antigen, B-45 alpha chain OS=Homo sapiens GN=HLA-B PE=2 SV=1 - [1B45_HUMAN]	37,02	0															
Q31612	HLA class I histocompatibility antigen, B-73 alpha chain OS=Homo sapiens GN=HLA-B PE=1 SV=1 - [1B73_HUMAN]	33,06	0															
Q04826	HLA class I histocompatibility antigen, B-40 alpha chain OS=Homo sapiens GN=HLA-B PE=1 SV=1 - [1B40_HUMAN]	30,66	0															
Q5T2N8	ATPase family AAA domain-containing protein 3C OS=Homo sapiens GN=ATAD3C PE=1 SV=2 - [ATD3C_HUMAN]	28,22	0															
Q9UPN3-3	Isoform 3 of Microtubule-actin cross-linking factor 1, isoforms 1/2/3/5 OS=Homo sapiens GN=MACF1 - [MACF1_HUMAN]	11,16	0															
F8W6A0	Periphilin-1 OS=Homo sapiens GN=PPHLN1 PE=4 SV=1 - [F8W6A0_HUMAN]	7,99	0															
Q8NEY8-3	Isoform 3 of Periphilin-1 OS=Homo sapiens GN=PPHLN1 - [PPHLN_HUMAN]	6,68	0															
Q99456	Keratin, type I cytoskeletal 12 OS=Homo sapiens GN=KRT12 PE=1 SV=1 - [K1C12_HUMAN]	5,06	0															

Q8TDM6-2													4,29	0,82	1	2				
Q96Q06					2,44	0,81	1	1												
Q8N7Z5	42,51	0,37	1	1	2,10	0,43	1	1	25,72	0,43	1	1	3,75	0,43	1	2				
P06681									27,35	0,80	1	1								
H0YMW2													2,68	0,80	1	1				
O43314																				
Q8NEB9																	37,41	0,79	1	1
Q9H9Y6																				
O94855	25,56	0,78	1	1	2,40	0,78	1	1												
Q96C45																				
Q14159	26,82	0,77	1	1					31,69	0,77	1	1	2,23	0,77	1	1				
Q9Y6J0																	43,60	0,77	1	1
Q92766																	53,53	0,77	1	1
Q9UPY3-2																				
Q8TEK3																	48,85	0,75	1	1
O15056																				
Q8IVE3																				
Q9P1Y6																				
F5H156													1,90	0,72	1	1				
Q92547																				
Q8N283													1,84	0,70	1	1				
Q5VWN6																				
Q9NYI0	29,86	0,67	1	2	2,39	0,67	1	1	104,03	0,67	1	5	8,39	0,67	1	4				
P21359	48,79	0,42	1	1	4,18	0,42	1	1	0,00	0,25	1	1								
Q8NB14	30,20	0,67	1	1	2,18	0,67	1	1												
Q9Y2I7																				
Q8WVS4	23,14	0,66	1	1					51,27	0,66	1	2	2,41	0,66	1	1				
Q5T5U3																	48,21	0,66	1	1
Q7Z460																	37,02	0,65	1	1
C9J815																				
Q96MR6									30,47	0,64	1	1	2,33	0,64	1	1				
Q12769	37,32	0,63	1	3	11,95	0,63	1	4												
Q6ZV73																				
Q9UKN7	45,50	0,20	1	3	6,73	0,20	1	3	33,26	0,62	2	2	2,13	0,20	1	1				
Q9UKZ4																				
Q12789-3																				
Q5VW36					2,07	0,61	1	1												
Q9P2D3-3					3,02	0,61	1	1												
Q9Y3R5-2					3,53	0,61	1	1												
Q06190																				
Q6TFL3									68,83	0,60	1	2	5,60	0,60	1	2				
Q8IZY2-2													1,82	0,60	1	1				
O95255																				
P42684																				
Q7Z3U7									55,58	0,58	1	1	3,08	0,58	1	1				
O60287									41,87	0,57	1	1	3,06	0,57	1	1				
P78504									34,32	0,57	1	1	2,43	0,57	1	1				
Q5SNV9									69,22	0,54	1	3	6,35	0,54	1	3	164,66	0,54	1	6
Q96BY7																				
Q66GS9																				
O14647-2					1,86	0,52	1	1												
Q5JR59					1,62	0,51	1	1												
Q7Z7M0-2					2,45	0,50	1	1												
Q9NTG1	0,00	0,49	1	1	2,14	0,49	1	1					2,39	0,49	1	1				
Q96Q15																	58,89	0,49	1	1
Q13439																				
Q9UBG0													1,79	0,47	1	1				
Q70CQ4	43,60	0,44	1	1																
Q92817																	22,35	0,44	1	1
P15822																				
Q9Y4C0	67,64	0,43	1	4	10,92	0,43	1	4												
Q13315																	37,81	0,43	1	1
O94822									23,71	0,40	1	2								
Q9HCM1																				
Q86UK0	22,74	0,39	1	1																
Q6UB99																				
Q9NYC9					2,61	0,16	1	1	30,15	0,33	2	3	9,50	0,33	2	4				
P49815									23,55	0,33	1	1								
P20930	23,37	0,32	1	1	3,32	0,32	1	1												
Q15413					0,00	0,18	1	1												
Q01484;I6L894																				
Q8NEZ4	38,61	0,14	1	1	1,92	0,14	1	1	39,38	0,14	1	1								
Q5D862	26,84	0,29	1	1									2,19	0,29	1	1				
Q9P225																				
E9PF99					2,49	0,28	1	1												
P46100																				
P13611																				
Q7Z7G8-2					2,66	0,23	1	1												
Q5T011	30,68	0,17	1	1																
Q9NZJ4									51,86	0,15	1	3	9,48	0,15	1	4				
Q8WZ42-3																				
P62807	4064,60	52,38	7	119	467,42	47,62	6	114	2121,42	44,44	6	73	239,11	39,68	5	75	1434,96	55,56	9	42
Q99877	4064,60	52,38	7	119	467,42	47,62	6	114	2121,42	44,44	6	73	239,11	39,68	5	75	1328,96	55,56	9	36
P16188	1837,90	46,30	15	58	296,42	48,22	16	69	1198,44	36,99	11	39	175,32	38,90	12	43				
P30492	825,82	38,40	10	27	123,55	38,40	9	28	814,49	22,38	6	26	108,80	29,01	7	30	40,86	3,87	1	1
P30464	1109,36	35,36	9	34	146,01	35,36	9	38	945,40	23,20	6	30	121,62	29,83	7	35				
P30475	1171,88	35,36	9	36	151,09	35,36	9	39	650,43	23,20	6	24	97,02	29,83	7	29				
P30466	1097,67	34,81	8	34	147,87	35,08	9	38	597,84	22,65	6	19	79,90	29,28	7	24				
P30462	864,17	34,53	8	27	127,71	34,81	9	29	462,17	22,38	6	15	66,96	29,01	7	19				
P18464	1027,71	33,98	8	33	168,80	34,25	9	42	804,03	26,24	7	23	100,99	32,87	8	29				
P10319	1015,64	34,25	9	33	164,26	34,25	9	41	911,86	26,52	7	31	124,56	33,15	8	37				
O19617					160,93	40,71	12	46					143,55	34,97	10	48				
P30460	1073,91	36,46	10	37	165,55	36,46	10	38	676,16	22,65	7	22	99,95	22,65	7	27				
P30483	726,02	30,39	8	24	107,56	30,39	7	24	518,01	15,75	4	21	82,22	22,38	5	25				
Q31612	872,48	30,30	7	28	135,36	30,58	8	39	694,09	28,37	7	24	95,39	28,37	7	33				
Q04826	908,94	30,66	9	33	142,03	27,62	7	32	669,48	16,02	5	25	85,40	12,98	4	23				
Q5T2N8	567,82	26,28	9	26	111,65	28,22	10	32	232,00	16,06	6	14	62,39	20,92	7	24				
Q9UPN3-3					164,61	7,28	27	41					88,95	3,70	14	21				
F8W6A0																				
Q8NEY8-3																				
Q99456	62,29	1,42	1	3	10,61	1,42	1	4	42,05	1,42	1	3	7,58	1,42	1	3				

Q8TDM6-2																					1579	176,9	6,93
Q96Q06																					1357	134,3	8,73
Q8N7Z5																					1873	210,7	6,20
P06681																					752	83,2	7,42
H0YMW2																					1745	186,0	4,75
O43314					78,16	0,80	1	2	6,37	0,80	1	2	48,82	0,80	1	1	2,78	0,80	1	1	1243	140,3	8,22
Q8NEB9	2,46	0,79	1	1	36,93	0,79	1	1	2,31	0,79	1	1									887	101,5	6,81
Q9H9Y6					34,33	0,79	1	1	2,75	0,79	1	1	60,74	0,79	1	1	3,03	0,79	1	1	1135	128,1	7,83
O94855																					1032	112,9	7,25
Q96C45									1,72	0,78	1	1									1275	142,4	6,29
Q14159																					915	100,3	6,62
Q9Y6J0	6,05	0,77	1	1																	2220	246,2	6,02
Q92766	3,73	0,77	1	1																	1687	181,3	6,98
Q9UPY3-2																	2,65	0,77	1	1	1829	208,3	5,73
Q8TEK3	5,54	0,75	1	1	27,98	0,75	1	1	4,13	0,75	1	1									1739	184,7	9,33
O15056					99,55	0,74	1	2	6,95	0,74	1	2									1496	165,4	7,34
Q8IVE3													20,06	0,74	1	1					1493	168,1	7,56
Q9P1Y6													23,34	0,73	1	1	3,48	0,73	1	1	1649	178,6	8,95
F5H156																					967	100,9	7,59
Q92547					61,01	0,72	1	1	3,18	0,72	1	1	18,73	0,72	1	1	3,44	0,72	1	1	1522	170,6	6,96
Q8N283					42,87	0,70	1	1	2,06	0,70	1	1									1001	109,9	6,02
Q5VWN6					33,27	0,45	1	1	3,30	0,45	1	1	28,14	0,45	1	1	5,23	0,70	2	2	2430	268,7	5,90
Q9NYI0																					1048	116,0	5,99
P21359																					2839	319,2	7,39
Q8NB14																					1042	116,5	6,21
Q9Y2I7													23,60	0,67	1	1	3,66	0,67	1	1	2098	237,0	6,70
Q8WVS4																					1066	122,5	7,31
Q5T5U3	4,78	0,66	1	1																	1957	217,2	7,80
Q7Z460	3,04	0,65	1	1																	1538	169,3	9,03
C9J815									2,11	0,65	1	1									1069	113,0	4,37
Q96MR6																					1250	144,9	5,80
Q12769																					1436	162,0	5,50
Q6ZV73					44,40	0,63	1	1	3,86	0,63	1	1									1430	160,7	7,03
Q9UKN7																					3530	395,0	9,17
Q9UKZ4	4,38	0,62	1	1																	2725	304,8	6,42
Q12789-3									3,23	0,62	1	1									2084	236,1	7,52
Q5VW36													31,04	0,61	1	1	3,22	0,61	1	1	1801	199,9	6,62
Q9P2D3-3																					1982	214,9	7,42
Q9Y3R5-2																					2291	257,2	6,40
Q06190					28,55	0,61	1	1	2,48	0,61	1	1									1150	130,2	5,21
Q6TFL3																					1326	152,7	6,81
Q8IZY2-2																					2008	218,4	6,98
O95255																	1,67	0,60	1	1	1503	164,8	8,50
P42684									1,82	0,59	1	1	45,57	0,59	1	1	2,04	0,59	1	1	1182	128,3	8,07
Q7Z3U7																					1718	190,4	6,10
O60287																					2271	254,2	6,47
P78504																					1218	133,7	6,06
Q5SNV9	14,10	0,54	1	6	311,94	0,54	1	14	30,10	0,54	1	14	131,29	0,54	1	5	11,47	0,54	1	5	1468	162,3	10,70
Q96BY7									2,44	0,53	1	1									2078	232,6	5,76
Q66GS9																	0,00	0,53	1	1	1140	133,4	6,21
O14647-2																					1739	200,4	7,84
Q5JR59																					1369	150,1	6,68
Q7Z7M0-2																					2778	295,7	6,86
Q9NTG1																					2253	255,3	9,11
Q96Q15	4,16	0,49	1	1																	3661	410,2	6,46
Q13439													52,41	0,49	1	1	4,03	0,49	1	1	2230	261,0	5,39
Q9UBG0																					1479	166,6	5,83
Q70CQ4																					1352	146,6	9,22
Q92817	2,77	0,44	1	1																	2033	231,5	6,96
P15822					25,25	0,44	2	2	2,07	0,22	1	1	0,00	0,22	1	1	2,13	0,22	1	1	2718	296,7	7,84
Q9Y4C0																					1643	180,5	5,54
Q13315	3,92	0,43	1	1																	3056	350,5	6,81
O94822																					1766	200,4	6,25
Q9HCM1					36,13	0,40	1	2	4,31	0,40	1	2									1747	194,7	8,78
Q86UK0																					2595	293,0	7,75
Q6UB99					19,76	0,38	1	1	2,82	0,38	1	1									2663	297,7	7,11
Q9NYC9																					4486	511,6	5,91
P49815																					1807	200,5	7,31
P20930																					4061	434,9	9,25
Q15413					35,29	0,12	1	2	3,69	0,12	1	2	37,33	0,12	1	2	5,89	0,12	1	3	4870	551,7	5,68
Q01484;I6L894					24,94	0,31	1	1	2,53	0,31	1	1									3924	430,0	5,12
Q8NEZ4					39,55	0,14	1	1	2,00	0,14	1	1									4911	541,0	6,49
Q5D862																					2391	247,9	8,31
Q9P225													26,11	0,29	1	1	2,92	0,29	1	1	4427	507,4	6,37
E9PF99																					2537	278,9	5,48
P46100					34,09	0,28	1	1	2,01	0,28	1	1									2492	282,4	6,58
P13611					28,24	0,24	1	1													3396	372,6	4,51
Q7Z7G8-2																					3997	445,7	6,46
Q5T011																					3432	377,8	6,27
Q9NZJ4																					4579	520,8	7,05
Q8WZ42-3	3,28	0,05	2	2					3,87	0,05	2	2									26926	2991,1	6,74
P62807	129,08	54,76	8	36	2611,34	61,11	9	91	270,76	52,38	8	84	2016,18	44,44	6	63	270,26	54,76	8	71	126	13,9	10,32
Q99877	129,08	54,76	8	36	2479,84	61,11	9	81	270,76	52,38	8	84	2016,18	44,44	6	63	270,26	54,76	8	71	126	13,9	10,32
P16188					39,45	6,58	2	2	10,03	6,58	2	3	46,39	6,30	2	2	9,56	5,75	2	3	365	40,9	6,10
P30492	3,42	3,87	1	1	39,45	6,63	2	2	10,03	6,63	2	3	25,39	3,59	1	1	5,50	3,04	1	2	362	40,4	6,30
P30464					39,45	6,63	2	2	10,03	6,63	2	3	25,39	3,59	1	1	5,50	3,04	1	2	362	40,4	6,30
P30475					39,45	6,63	2	2	10,03	6,63	2	3	25,39	3,59	1	1	5,50	3,04	1	2	362	40,3	6,15
P30466					39,45	3,59	1	1	3,62	3,59	1	1	25,39	3,59	1	1					362	40,3	6,76
P30462					39,45	3,59	1	1	3,62	3,59	1	1	25,39	3,59	1	1					362	40,3	5,83
P18464					39,45	3,59	1	1	3,62	3,59	1	1	25,39	3,59	1	1					362	40,5	6,39
P10319					39,45	6,63	2	2	10,03	6,63	2	3	25,39	3,59	1	1	8,41	6,08	2	3	362	40,3	6,30
O19617	3,42	3,83	1	1					10,03	6,56	2	4					16,33	12,30	4	5	366	40,7	5,87
P30460					39,45	3,59	1	1	3,62	3,59	1	1	46,39	6,35	2	2	4,06	2,76	1	1	362	40,3	5,71
P30483					39,45	6,63	2	2	10,03	6,63	2	3	25,39	3,59	1	1	8,41	6,08	2	3	362	40,4	6,47
Q31612					39,45	3,58	1	2	3,62	3,58	1	2	46,39	6,34	2	2	7,92	6,34	2	2	363	40,4	6,24
Q04826					39,45	6,63	2	2	3,62	3,59	1	1	46,39	6,35	2	2	4,06	2,76	1	1	362	40,5	6,54
Q5T2N8																					411	46,4	9,31
Q9UPN3-3	26,61	1,60	7	8					15,64	0,63	3	6					71,81	2,29	9	18	5374	613,8	5,36
F8W6A0	9,13	7,99	2	2																	363	41,1	8,57
Q8NEY8-3	9,13	6,68	2	2																	434	49,6	8,72
Q99456					0,00	1,42	1	1	2,43	1,42	1	1					4,47	3,64	2	2	494	53,5	4,78

Supplementary table 2b

Supplementary table 2b summarizes proteins that are significantly regulated in HCC827 cell lines that are resistant to 10 (ER10), 20 (ER20), or 30 (ER30) uM erlotinib relative to the sensitive HCC827 cell line. "+" indicate statistically significant regulated proteins after correction for multiple tests by the Benjamini-Hochberg method as determined by the Perseus software. SOL indicate solulable proteins and MEMB indicate proteins analyzed from the membrane fraction. Log2 transformed values that are negative indicate down-regulated proteins in the respective erlotinib-resistant cell lines relative to the HCC827 erlotinib-sensitive control cell line. Positive log2 transformed values indicate up-regulated proteins.

		Protein regulation						p values			
Accession	Description	Mean ER10 MEMB	Mean ER20 MEMB	Mean ER30 MEMB	Mean ER10 SOL	Mean ER20 SOL	Mean ER30 SOL	SignificantMEMB	p valueMEMB	SignificantSOL	p valueSOL
P61604	10 kDa heat shock protein, mitochondrial OS=Homo sapiens GN=HSPE1 PE=1 SV=2 - [CH10_HUMAN]	-0,72	-0,67	-0,49	-0,12	-0,21	1,10	+	0,000671529		0,267395418
P62258	14-3-3 protein epsilon OS=Homo sapiens GN=YWHAE PE=1 SV=1 - [1433E_HUMAN]	-0,21	-0,26	-0,16	0,02	0,06	-0,41	+	0,000168849		0,188262537
Q04917	14-3-3 protein eta OS=Homo sapiens GN=YWHAH PE=1 SV=4 - [1433F_HUMAN]	0,26	0,42	0,22	0,57	0,55	-0,40	+	0,000784307		0,26322229
Q9C0C2	182 kDa tankyrase-1-binding protein OS=Homo sapiens GN=TNKS1BP1 PE=1 SV=4 - [TB182_HUMAN]	-0,35	-0,14	-0,29	0,02	0,03	0,36	+	0,00701103		0,087302813
Q8N3E9	1-phosphatidylinositol-4,5-bisphosphate phosphodiesterase delta-3 OS=Homo sapiens GN=PLCD3 PE=1 SV=3 - [PLCD3_HUMAN]	0,71	0,75	0,31	#DIVISION/0!	#DIVISION/0!	#DIVISION/0!	+	0,001983261		1
P09543	2',3'-cyclic-nucleotide 3'-phosphodiesterase OS=Homo sapiens GN=CNP PE=1 SV=2 - [CN37_HUMAN]	-0,34	-0,48	-0,53	-0,08	-0,08	-0,19	+	0,002108031		0,017305663
P29728	2'-5'-oligoadenylate synthase 2 OS=Homo sapiens GN=OAS2 PE=1 SV=3 - [OAS2_HUMAN]	-1,56	-1,14	-1,33	-0,67	-0,41	0,02	+	0,000207771		0,22304134
Q16401	26S proteasome non-ATPase regulatory subunit 5 OS=Homo sapiens GN=PSMD5 PE=1 SV=3 - [PSMD5_HUMAN]	-0,23	-0,23	-0,15	-0,13	0,03	-0,34	+	0,00200338		0,327963395
Q9NVS2	28S ribosomal protein S18a, mitochondrial OS=Homo sapiens GN=MRPS18A PE=1 SV=1 - [RT18A_HUMAN]	0,31	0,19	0,50	0,49	0,23	1,19	+	0,003522197		0,003455389
Q9Y3D9	28S ribosomal protein S23, mitochondrial OS=Homo sapiens GN=MRPS23 PE=1 SV=2 - [RT23_HUMAN]	-0,51	-0,36	-0,28	#DIVISION/0!	#DIVISION/0!	#DIVISION/0!	+	0,00350725		1
P51398	28S ribosomal protein S29, mitochondrial OS=Homo sapiens GN=DAP3 PE=1 SV=1 - [RT29_HUMAN]	-0,27	-0,26	-0,24	0,00	0,15	-0,39	+	0,003126953		0,500141697
Q9UJ83	2-hydroxyacyl-CoA lyase 1 OS=Homo sapiens GN=HACL1 PE=1 SV=2 - [HACL1_HUMAN]	0,16	0,23	0,21	#DIVISION/0!	#DIVISION/0!	#DIVISION/0!	+	0,003299001		1
Q02218	2-oxoglutarate dehydrogenase, mitochondrial OS=Homo sapiens GN=OGDH PE=1 SV=3 - [ODO1_HUMAN]	-0,40	-0,47	-0,30	0,10	-0,06	-0,45	+	0,000242868		0,150036743
P12694	2-oxoisovalerate dehydrogenase subunit alpha, mitochondrial OS=Homo sapiens GN=BCKDHA PE=1 SV=2 - [ODBA_HUMAN]	-0,47	-0,37	-0,45	0,05	-0,51	-0,10	+	0,00466948		0,384841992
Q9H2F3	3 beta-hydroxysteroid dehydrogenase type 7 OS=Homo sapiens GN=HSD3B7 PE=1 SV=2 - [3BHS7_HUMAN]	0,42	0,91	0,60	#DIVISION/0!	#DIVISION/0!	#DIVISION/0!	+	0,000896136		1
O95861	3'(2'),5'-bisphosphate nucleotidase 1 OS=Homo sapiens GN=BPNT1 PE=1 SV=1 - [BPNT1_HUMAN]	-0,68	-0,49	-0,61	-0,68	-0,42	-1,18	+	0,00015805		0,076171766
P52815	39S ribosomal protein L12, mitochondrial OS=Homo sapiens GN=MRPL12 PE=1 SV=2 - [RM12_HUMAN]	-0,33	-0,21	-0,21	0,33	0,17	1,09	+	0,000610971		0,007553853
Q9NQ50	39S ribosomal protein L40, mitochondrial OS=Homo sapiens GN=MRPL40 PE=1 SV=1 - [RM40_HUMAN]	-0,63	-0,63	-0,22	-0,03	-0,22	0,46	+	0,00694928		0,574742718
Q9BRJ2	39S ribosomal protein L45, mitochondrial OS=Homo sapiens GN=MRPL45 PE=1 SV=2 - [RM45_HUMAN]	0,25	0,44	0,38	0,64	0,70	0,13	+	0,000441194		0,007923929
Q15125	3-beta-hydroxysteroid-Delta(8),Delta(7)-isomerase OS=Homo sapiens GN=EBP PE=1 SV=3 - [EBP_HUMAN]	-0,50	-0,52	-0,13	#DIVISION/0!	#DIVISION/0!	#DIVISION/0!	+	0,006457484		1
Q6Y1H2	3-hydroxyacyl-CoA dehydratase 2 OS=Homo sapiens GN=PTPLB PE=1 SV=1 - [HACD2_HUMAN]	0,71	0,51	0,56	0,17	0,33	0,10	+	2,01E-05		0,114439728
P31937	3-hydroxyisobutyrate dehydrogenase, mitochondrial OS=Homo sapiens GN=HIBADH PE=1 SV=2 - [3HIDH_HUMAN]	-0,65	-0,88	-0,72	-0,20	-0,71	-0,43	+	3,05E-05		0,012568484
Q6NVY1	3-hydroxyisobutyryl-CoA hydrolase, mitochondrial OS=Homo sapiens GN=HIBCH PE=1 SV=2 - [HIBCH_HUMAN]	-0,53	-0,58	-0,43	-0,20	-0,31	-0,36	+	0,000426962		0,025088988
P39019	40S ribosomal protein S19 OS=Homo sapiens GN=RPS19 PE=1 SV=2 - [RS19_HUMAN]	-0,58	-0,26	-0,24	0,21	0,48	0,59	+	0,005041677	+	0,000381317
P08195	4F2 cell-surface antigen heavy chain OS=Homo sapiens GN=SLC3A2 PE=1 SV=3 - [4F2_HUMAN]	0,66	1,04	0,83	0,19	0,59	-0,29	+	7,43E-05		0,291289838
Q8TCD5	5'(3')-deoxyribonucleotidase, cytosolic type OS=Homo sapiens GN=NT5C PE=1 SV=2 - [NT5C_HUMAN]	-0,31	-0,38	-0,40	-0,11	-0,40	-1,06	+	0,005641315		0,202674312
Q9Y478	5'-AMP-activated protein kinase subunit beta-1 OS=Homo sapiens GN=PRKAB1 PE=1 SV=4 - [AAKB1_HUMAN]	-0,32	-0,18	-0,46	0,48	0,15	-0,73	+	0,004661688		0,931963955
P10809	60 kDa heat shock protein, mitochondrial OS=Homo sapiens GN=HSPD1 PE=1 SV=2 - [CH60_HUMAN]	-0,45	-0,55	-0,26	-0,14	-0,23	-0,07	+	[illegible]	[illegible]	[illegible]
P40429	60S ribosomal protein L13a OS=Homo sapiens GN=RPL13A PE=1 SV=2 - [RL13A_HUMAN]	-0,09	-0,21	-0,11	-0,22	0,03	-0,98	+	[illegible]	[illegible]	[illegible]
P46779	60S ribosomal protein L28 OS=Homo sapiens GN=RPL28 PE=1 SV=3 - [RL28_HUMAN]	0,44	0,28	0,43	0,12	0,26	-1,06	+	[illegible]	[illegible]	[illegible]
P62888	60S ribosomal protein L30 OS=Homo sapiens GN=RPL30 PE=1 SV=2 - [RL30_HUMAN]	-0,72	-0,44	-0,22	0,06	0,33	-0,39	+	[illegible]	[illegible]	[illegible]
P61927	60S ribosomal protein L37 OS=Homo sapiens GN=RPL37 PE=1 SV=2 - [RL37_HUMAN]	0,95	0,72	0,64	0,11	0,27	1,22	+	[illegible]	[illegible]	[illegible]
P11021	78 kDa glucose-regulated protein OS=Homo sapiens GN=HSPA5 PE=1 SV=2 - [GRP78_HUMAN]	0,23	0,20	0,20	0,03	-0,15	0,39	+	[illegible]	[illegible]	[illegible]
Q8NFV4	Abhydrolase domain-containing protein 11 OS=Homo sapiens GN=ABHD11 PE=1 SV=1 - [ABHDB_HUMAN]	-1,34	-0,80	-0,70	-0,77	-0,66	-0,62	+	[illegible]	[illegible]	[illegible]
Q13085	Acetyl-CoA carboxylase 1 OS=Homo sapiens GN=ACACA PE=1 SV=2 - [ACACA_HUMAN]	0,73	1,11	0,60	0,30	0,84	-0,25	+	[illegible]	[illegible]	[illegible]
Q92485	Acid sphingomyelinase-like phosphodiesterase 3b OS=Homo sapiens GN=SMPDL3B PE=2 SV=2 - [ASM3B_HUMAN]	-1,10	-1,16	-0,92	#DIVISION/0!	#DIVISION/0!	#DIVISION/0!	+	[illegible]	[illegible]	[illegible]
Q99798	Aconitate hydratase, mitochondrial OS=Homo sapiens GN=ACO2 PE=1 SV=2 - [ACON_HUMAN]	-0,17	-0,15	-0,23	0,20	-0,19	-0,28	+	[illegible]	[illegible]	[illegible]
Q12979	Active breakpoint cluster region-related protein OS=Homo sapiens GN=ABR PE=2 SV=2 - [ABR_HUMAN]	-0,56	-0,49	-0,52	-0,43	-0,14	-1,36	+	[illegible]	[illegible]	[illegible]
Q6JQN1	Acyl-CoA dehydrogenase family member 10 OS=Homo sapiens GN=ACAD10 PE=2 SV=1 - [ACD10_HUMAN]	-0,47	-0,57	-0,53	0,08	-0,18	-0,76	+	[illegible]	[illegible]	[illegible]
Q96CM8	Acyl-CoA synthetase family member 2, mitochondrial OS=Homo sapiens GN=ACSF2 PE=1 SV=2 - [ACSF2_HUMAN]	-0,40	-0,66	-0,17	0,09	-0,34	0,05	+	[illegible]	[illegible]	[illegible]
Q6P587	Acylpyruvase FAHD1, mitochondrial OS=Homo sapiens GN=FAHD1 PE=1 SV=2 - [FAHD1_HUMAN]	-0,47	-0,61	-0,66	#DIVISION/0!	#DIVISION/0!	#DIVISION/0!	+	[illegible]	[illegible]	[illegible]
P00813	Adenosine deaminase OS=Homo sapiens GN=ADA PE=1 SV=3 - [ADA_HUMAN]	-0,46	-0,76	-0,45	#DIVISION/0!	#DIVISION/0!	#DIVISION/0!	+	[illegible]	[illegible]	[illegible]
[illegible]	[illegible]	[illegible]	[illegible]	[illegible]	[illegible]	[illegible]	[illegible]	[illegible]	[illegible]	[illegible]	[illegible]

Log2 transformed relative protein expression in replicates of proteins in the membrane fractions and solulable fractions of erlotinib-resistant cell lines relative to the HCC827 cell line

Accession	Log2 MEMB1 ER10/HCC827	Log2 MEMB2 ER10/HCC827	Log2 MEMB1 ER20/HCC827	Log2 MEMB2 ER20/HCC827	Log2 MEMB1 ER30/HCC827	Log2 MEMB2 ER30/HCC827	Log2 SOL1 ER10/HCC827	Log2 SOL2 ER10/HCC827	Log2 SOL3 ER10/HCC827	Log2 SOL1 ER20/HCC827	Log2 SOL2 ER20/HCC827	Log2 SOL3 ER20/HCC827	Log2 SOL1 ER30/HCC827	Log2 SOL2 ER30/HCC827	Log2 SOL3 ER30/HCC827
P61604	-0,7278309	-0,3672422	-0,8131606	-0,521951	-0,6189604	-0,3622633	-0,1024958	-0,09789427	-0,1703671	-0,2449261	-0,2109985	-0,1692535	1,17521	1,266766	0,8542483
P62258	-0,220384	-0,2017765	-0,2680578	-0,247813	-0,1964629	-0,1210445	0,0037817	0,02251835	0,02389047	0,04349285	0,06848436	0,07527647	-0,4695929	-0,3110458	-0,4483858
Q04917	0,2793413	0,248834	0,4824284	0,3540363	0,2298876	0,2121051	0,6046422	0,6676995	0,4284695	0,350186	0,6970787	0,5966151	-1,060038	0,2634777	-0,4082968
Q9C0C2	-0,5046206	-0,3947955	-0,1461558	-0,1367503	-0,4133039	-0,1617163	0,07160284	-0,006384483	0,000302602	0,1788734	-0,09471145	-0,005139922	0,2072869	0,2823321	0,5921206
Q8N3E9	0,8677894	0,5500298	0,7804989	0,716375	0,2275192	0,3974715									
P09543	-0,1547962	-0,2706881	-0,4489491	-0,5032713	-0,4308724	-0,6377239	-0,07954063	0,02001586	-0,1795904	0,08896254	-0,1076672	-0,2199238	-0,175018	-0,2832706	-0,1079141
P29728	-1,932498	-1,191003	-1,328589	-0,958011	-1,511606	-1,141978	-0,6722122			-0,4051425			0,02076998		
Q16401	-0,2332202	-0,2295818	-0,1279822	-0,329784	-0,2036147	-0,09245709		0,06368558	-0,3149777		-0,02866186	0,08527913		-0,7362258	0,06461974
Q9NVS2	0,4038384	0,223355	0,2100862	0,1613943	0,5580489	0,4408589	0,402582	0,5546369	0,4990091	0,2698557	0,1821628	0,2375818	1,579352	0,9380845	1,040625
Q9Y3D9	-0,7003514	-0,3116371	-0,4851483	-0,2344815	-0,2896571	-0,2622322									
P51398	-0,4373721	-0,09281032	-0,2315833	-0,2935047	-0,3044482	-0,1799256	0,06894676	-0,2484852	0,1825131	0,03889702	0,1240669	0,2907738	-0,1133848	-0,8199174	-0,2272014
Q9UJ83	0,057347	0,2715492	0,2256782	0,2360834	0,1201999	0,3030371									
Q02218	-0,3647754	-0,4329359	-0,3754515	-0,5676414	-0,31361	-0,2784093	0,04672099	0,05877627	0,1962727	-0,08997852	-0,02133462	-0,08088866	-0,615616	-0,3126716	-0,4327631
P12694		-0,4687837		-0,3717417		-0,447419			0,05006074			-0,5052366			-0,09521233
Q9H2F3	0,4890665	0,3565522	0,9082584	0,90563	0,6192017	0,5757026									
O95861	-0,681338	-0,6780566	-0,3372407	-0,6395421	-0,5154005	-0,7098914			-0,676479			-0,4225313			-1,180014
P52815	-0,2713036	-0,3890495	-0,2553361	-0,163471	-0,2509502	-0,178076	0,5450341	0,2305078	0,2125356	0,3454579	0,1865402	-0,02435691	1,166235	0,995038	1,107864
Q9NQ50	-0,443729	-0,8233774	-0,5550221	-0,6979444	-0,03497705	-0,4043178	0,02048587	-0,2916428	0,1773047	-0,1820973	-0,4405995	-0,05013898	0,6407307	0,4027661	0,3415923
Q9BRJ2	0,2420375	0,2518822	0,3949594	0,48972	0,3013858	0,4590623	0,6045793	0,4113929	0,897385	0,8027702	0,4299225	0,8599309	0,6801062	0,1068721	-0,3963563
Q15125	-0,4193678	-0,5888232	-0,4861265	-0,5535647	-0,1721106	-0,08132272									
Q6Y1H2	0,6716443	0,7404984	0,5119299	0,5095027	0,5794867	0,5468719		0,3041966	0,04181761		0,4208482	0,2469201		0,4316394	-0,2385601
P31937	-0,678958	-0,6254513	-0,8102714	-0,945901	-0,6245052	-0,8188392		-0,02245904	-0,382366		-0,6303654	-0,7809008		-0,2269491	-0,6285796
[illegible]	[illegible]	[illegible]	[illegible]	[illegible]	[illegible]	[illegible]	[illegible]	[illegible]	[illegible]	[illegible]	[illegible]	[illegible]	[illegible]	[illegible]	[illegible]

O95299	NADH dehydrogenase [ubiquinone] 1 alpha subcomplex subunit 10, mitochondrial OS=Homo sapiens GN=NDUFA10 PE=1 SV=1 - [NDUA	0,47	0,35	0,32	-0,49	-0,29	-0,11	+	5,35E-05	
O95168	NADH dehydrogenase [ubiquinone] 1 beta subcomplex subunit 4 OS=Homo sapiens GN=NDUFB4 PE=1 SV=3 - [NDUB4_HUMAN]	0,24	0,35	0,19	#DIVISION/0!	#DIVISION/0!	#DIVISION/0!	+	0,002697409	
P17568	NADH dehydrogenase [ubiquinone] 1 beta subcomplex subunit 7 OS=Homo sapiens GN=NDUFB7 PE=1 SV=4 - [NDUB7_HUMAN]	0,29	0,43	0,16	#DIVISION/0!	#DIVISION/0!	#DIVISION/0!	+	0,002860948	
O43920	NADH dehydrogenase [ubiquinone] iron-sulfur protein 5 OS=Homo sapiens GN=NDUFS5 PE=1 SV=3 - [NDUS5_HUMAN]	0,45	0,53	0,14	0,53	0,34	1,53	+	0,004018092	
O96009	Napsin-A OS=Homo sapiens GN=NAPSA PE=1 SV=1 - [NAPSA_HUMAN]	-1,38	-1,07	-0,66	#DIVISION/0!	#DIVISION/0!	#DIVISION/0!	+	0,003524396	
Q92859	Neogenin OS=Homo sapiens GN=NEO1 PE=1 SV=2 - [NEO1_HUMAN]	0,29	0,47	0,39	#DIVISION/0!	#DIVISION/0!	#DIVISION/0!	+	0,004048714	
Q09666	Neuroblast differentiation-associated protein AHNAK OS=Homo sapiens GN=AHNAK PE=1 SV=2 - [AHNK_HUMAN]	0,90	0,98	0,61	0,41	0,76	1,17	+	0,000102741	+
A2RRP1	Neuroblastoma-amplified sequence OS=Homo sapiens GN=NBAS PE=1 SV=2 - [NBAS_HUMAN]	-0,56	-0,56	-0,38	#DIVISION/0!	#DIVISION/0!	#DIVISION/0!	+	0,001112723	
Q04721	Neurogenic locus notch homolog protein 2 OS=Homo sapiens GN=NOTCH2 PE=1 SV=3 - [NOTC2_HUMAN]	0,42	0,37	0,40	#DIVISION/0!	#DIVISION/0!	#DIVISION/0!	+	1,15E-05	
P62166	Neuronal calcium sensor 1 OS=Homo sapiens GN=NCS1 PE=1 SV=2 - [NCS1_HUMAN]	0,66	0,69	0,31	1,02	0,24	-0,05	+	0,000916049	
O14786	Neuropilin-1 OS=Homo sapiens GN=NRP1 PE=1 SV=3 - [NRP1_HUMAN]	-0,28	-0,39	-0,34	#DIVISION/0!	#DIVISION/0!	#DIVISION/0!	+	0,00352372	
P43007	Neutral amino acid transporter A OS=Homo sapiens GN=SLC1A4 PE=1 SV=1 - [SATT_HUMAN]	0,78	0,78	0,37	#DIVISION/0!	#DIVISION/0!	#DIVISION/0!	+	0,001223636	
Q6PIU2	Neutral cholesterol ester hydrolase 1 OS=Homo sapiens GN=NCEH1 PE=1 SV=3 - [NCEH1_HUMAN]	0,43	0,42	0,25	0,42	-0,01	-0,47	+	0,001459602	
P80188	Neutrophil gelatinase-associated lipocalin OS=Homo sapiens GN=LCN2 PE=1 SV=2 - [NGAL_HUMAN]	-1,95	-1,37	-1,21	#DIVISION/0!	#DIVISION/0!	#DIVISION/0!	+	0,001497554	
Q9UMS0	NFU1 iron-sulfur cluster scaffold homolog, mitochondrial OS=Homo sapiens GN=NFU1 PE=1 SV=2 - [NFU1_HUMAN]	-0,75	-0,72	-0,57	-0,20	-0,42	0,37	+	0,006333472	
Q6ZNB6	NF-X1-type zinc finger protein NFXL1 OS=Homo sapiens GN=NFXL1 PE=1 SV=2 - [NFXL1_HUMAN]	0,25	0,22	0,10	0,34	0,31	0,57	+	0,008103163	
Q13287	N-myc-interactor OS=Homo sapiens GN=NMI PE=1 SV=2 - [NMI_HUMAN]	-0,72	-0,73	-0,93	-0,74	-0,50	-0,89	+	0,007383401	
Q96E22	Nogo-B receptor OS=Homo sapiens GN=NUS1 PE=2 SV=1 - [NGBR_HUMAN]	0,65	0,57	0,72	#DIVISION/0!	#DIVISION/0!	#DIVISION/0!	+	0,004229702	
Q9C002	Normal mucosa of esophagus-specific gene 1 protein OS=Homo sapiens GN=NMES1 PE=2 SV=1 - [NMES1_HUMAN]	-0,91	-1,46	-1,85	-0,62	-1,09	-1,00	+	0,000494269	
P49321	Nuclear autoantigenic sperm protein OS=Homo sapiens GN=NASP PE=1 SV=2 - [NASP_HUMAN]	-0,44	-0,31	-0,33	-0,33	-0,26	0,30	+	5,33E-05	
O75694	Nuclear pore complex protein Nup155 OS=Homo sapiens GN=NUP155 PE=1 SV=1 - [NU155_HUMAN]	-0,42	-0,29	-0,28	0,20	-0,06	-0,53	+	0,002067915	
P67809	Nuclease-sensitive element-binding protein 1 OS=Homo sapiens GN=YBX1 PE=1 SV=3 - [YBOX1_HUMAN]	0,44	0,41	0,34	0,27	0,55	1,71	+	0,005081338	
Q02818	Nucleobindin-1 OS=Homo sapiens GN=NUCB1 PE=1 SV=4 - [NUCB1_HUMAN]	-0,72	-0,71	-0,60	-0,94	-1,25	1,11	+	2,35E-06	
E5RGW4	Nucleophosmin (Fragment) OS=Homo sapiens GN=NPM1 PE=4 SV=1 - [E5RGW4_HUMAN]	-0,21	-0,29	-0,26	0,39	0,30	0,77	+	8,78E-05	
Q9BTX1	Nucleoporin NDC1 OS=Homo sapiens GN=TMEM48 PE=1 SV=2 - [NDC1_HUMAN]	0,14	0,15	0,14	#DIVISION/0!	#DIVISION/0!	#DIVISION/0!	+	0,000143459	
P04181	Ornithine aminotransferase, mitochondrial OS=Homo sapiens GN=OAT PE=1 SV=1 - [OAT_HUMAN]	-1,47	-1,17	-0,91	-1,37	-1,24	-1,55	+	0,001635614	+
P10451	Osteopontin OS=Homo sapiens GN=SPP1 PE=1 SV=1 - [OSTP_HUMAN]	1,19	0,50	0,97	0,49	-0,25	2,20	+	0,001963327	
Q8TAD7	Overexpressed in colon carcinoma 1 protein OS=Homo sapiens GN=OCC1 PE=1 SV=2 - [OCC1_HUMAN]	1,14	0,81	0,47	#DIVISION/0!	#DIVISION/0!	#DIVISION/0!	+	0,00142278	
Q9BZF1	Oxysterol-binding protein-related protein 8 OS=Homo sapiens GN=OSBPL8 PE=1 SV=3 - [OSBL8_HUMAN]	0,37	0,47	0,28	-0,12	0,09	-0,36	+	0,000152951	
Q96RD7	Pannexin-1 OS=Homo sapiens GN=PANX1 PE=1 SV=4 - [PANX1_HUMAN]	0,26	0,33	0,45	#DIVISION/0!	#DIVISION/0!	#DIVISION/0!	+	0,000411049	
Q96HC4	PDZ and LIM domain protein 5 OS=Homo sapiens GN=PDLIM5 PE=1 SV=5 - [PDLI5_HUMAN]	-0,40	-0,10	-0,32	-0,13	-0,06	0,24	+	0,008061416	
O14908	PDZ domain-containing protein GIPC1 OS=Homo sapiens GN=GIPC1 PE=1 SV=2 - [GIPC1_HUMAN]	0,21	0,22	0,20	0,04	-0,12	0,14	+	0,002366285	
Q9Y3E5	Peptidyl-tRNA hydrolase 2, mitochondrial OS=Homo sapiens GN=PTRH2 PE=1 SV=1 - [PTH2_HUMAN]	-0,14	-0,10	-0,10	#DIVISION/0!	#DIVISION/0!	#DIVISION/0!	+	0,003438934	
O60437	Periplakin OS=Homo sapiens GN=PPL PE=1 SV=4 - [PEPL_HUMAN]	-1,36	-1,55	-1,17	-1,71	-1,12	0,25	+	0,004478424	
P30044	Peroxiredoxin-5, mitochondrial OS=Homo sapiens GN=PRDX5 PE=1 SV=4 - [PRDX5_HUMAN]	-0,61	-0,46	-0,81	0,13	-0,28	-0,88	+	0,000591303	
P56589	Peroxisomal biogenesis factor 3 OS=Homo sapiens GN=PEX3 PE=1 SV=1 - [PEX3_HUMAN]	-0,47	-0,47	-0,35	#DIVISION/0!	#DIVISION/0!	#DIVISION/0!	+	3,68E-05	
O96011	Peroxisomal membrane protein 11B OS=Homo sapiens GN=PEX11B PE=1 SV=1 - [PX11B_HUMAN]	-0,31	-0,81	-0,60	#DIVISION/0!	#DIVISION/0!	#DIVISION/0!	+	0,001908889	
O43808	Peroxisomal membrane protein PMP34 OS=Homo sapiens GN=SLC25A17 PE=1 SV=1 - [PM34_HUMAN]	-0,32	-0,21	-0,20	#DIVISION/0!	#DIVISION/0!	#DIVISION/0!	+	0,000709258	
Q9BYK8	Peroxisomal proliferator-activated receptor A-interacting complex 285 kDa protein OS=Homo sapiens GN=PRIC285 PE=1 SV=6 - [PR285	-1,21	-1,19	-1,04	-1,35	-1,25	-1,52	+	5,31E-05	
Q9Y285	Phenylalanine--tRNA ligase alpha subunit OS=Homo sapiens GN=FARSA PE=1 SV=3 - [SYFA_HUMAN]	-0,50	-0,52	-0,26	-0,32	0,06	-0,54	+	0,003687194	
Q9NSD9	Phenylalanine--tRNA ligase beta subunit OS=Homo sapiens GN=FARSB PE=1 SV=3 - [SYFB_HUMAN]	-0,36	-0,52	-0,27	-0,18	-0,08	-1,12	+	0,001473124	
Q9BTU6	Phosphatidylinositol 4-kinase type 2-alpha OS=Homo sapiens GN=PI4K2A PE=1 SV=1 - [P4K2A_HUMAN]	0,21	0,14	0,17	0,32	0,10	-0,77	+	0,003983673	
Q8TCG2	Phosphatidylinositol 4-kinase type 2-beta OS=Homo sapiens GN=PI4K2B PE=1 SV=1 - [P4K2B_HUMAN]	-0,24	-0,43	-0,49	#DIVISION/0!	#DIVISION/0!	#DIVISION/0!	+	0,001852689	
