# Supplementary material for: Combined FGFR and Akt pathway inhibition abrogates growth of FGFR1 overexpressing EGFR-TKI-resistant NSCLC cells
Source: NPJ Precis Oncol. 2021 Jul 15;5:65. doi: 10.1038/s41698-021-00208-w (PMC8282882; doi:10.1038/s41698-021-00208-w)
Supplement: Supplementary file 2 — Reporting summary. [file 41698_2021_208_MOESM2_ESM.pdf]

nature research

Corresponding author(s): Henrik J. Ditzel,

Last updated by author(s): Jun 2, 2021

Reporting Summary

Nature Research wishes to improve the reproducibility of the work that we publish. This form provides structure for consistency and transparency in reporting. For further information on Nature Research policies, see our Editorial Policies and the Editorial Policy Checklist.

Statistics

For all statistical analyses, confirm that the following items are present in the figure legend, table legend, main text, or Methods section.

n/a	Confirmed	
☐	☒	The exact sample size (*n*) for each experimental group/condition, given as a discrete number and unit of measurement
☐	☒	A statement on whether measurements were taken from distinct samples or whether the same sample was measured repeatedly
☐	☒	The statistical test(s) used AND whether they are one- or two-sided<br>*Only common tests should be described solely by name; describe more complex techniques in the Methods section.*
☐	☒	A description of all covariates tested
☐	☒	A description of any assumptions or corrections, such as tests of normality and adjustment for multiple comparisons
☐	☒	A full description of the statistical parameters including central tendency (e.g. means) or other basic estimates (e.g. regression coefficient) AND variation (e.g. standard deviation) or associated estimates of uncertainty (e.g. confidence intervals)
☐	☒	For null hypothesis testing, the test statistic (e.g. *F*, *t*, *r*) with confidence intervals, effect sizes, degrees of freedom and *P* value noted<br>*Give P values as exact values whenever suitable.*
☒	☐	For Bayesian analysis, information on the choice of priors and Markov chain Monte Carlo settings
☐	☒	For hierarchical and complex designs, identification of the appropriate level for tests and full reporting of outcomes
☒	☐	Estimates of effect sizes (e.g. Cohen's *d*, Pearson's *r*), indicating how they were calculated

Our web collection on statistics for biologists contains articles on many of the points above.

Software and code

Policy information about availability of computer code

Data collection	Proteome Discoverer version 1.4.0.288,
Data analysis	The SEQUEST search engine and Mascot search Engine (v. 2.2.3) integrated with Proteome Discoverer, GraphPad Prism, Stata/IC 16.0, QIAGEN Clinical Insight Analyze software

For manuscripts utilizing custom algorithms or software that are central to the research but not yet described in published literature, software must be made available to editors and reviewers. We strongly encourage code deposition in a community repository (e.g. GitHub). See the Nature Research guidelines for submitting code & software for further information.

Data

Policy information about availability of data

All manuscripts must include a data availability statement. This statement should provide the following information, where applicable:

- Accession codes, unique identifiers, or web links for publicly available datasets
- A list of figures that have associated raw data
- A description of any restrictions on data availability

Data availability
The mass spectrometry proteomics data generated during the study are publicly available in the PRIDE repository under the accession number https://identifiers.org/pride.project: PXD011803. Username: reviewer91580@ebi.ac.uk Password: 6b2nhXyt . Survival analyses and immunohistochemistry data are not publicly available to protect patient privacy, but will be made available to authorized researchers who have an approved Institutional Review Board application and have obtained approval from Dexeus Quirón University Hospital and Germans Trias i Pujol Hospital. Please contact the corresponding author with data access requests. The NSG data generated during the study are publicly available in the NCBI repository under the accession number https://www.ncbi.nlm.nih.gov/bioproject/?term=PRJNA524804. Sample accession numbers: SAMN11035315 (PC9-GR4); SAMN11035323 (11-18GR5) and https://www.ncbi.nlm.nih.gov/

bioproject/?term=PRJNA734250 sample accession numbers: SAMN19487316 (PC9GR4AZD2); SAMN19487317 (HCC827); SAMN19487318 (ER10); SAMN19487319 (ER20); SAMN19487320 (ER30). All other datasets generated during the study will be made available upon reasonable request to the corresponding author, Dr. Henrik Ditzel, email address: hditzel@health.sdu.dk. Uncropped Western blots are part of the supplementary information.

Field-specific reporting

Please select the one below that is the best fit for your research. If you are not sure, read the appropriate sections before making your selection.

☒ Life sciences ☐ Behavioural & social sciences ☐ Ecological, evolutionary & environmental sciences

For a reference copy of the document with all sections, see nature.com/documents/nr-reporting-summary-flat.pdf

Life sciences study design

All studies must disclose on these points even when the disclosure is negative.

Sample size	Power calculations were performed to determine that the number of animals and tumor samples should be sufficient to determine significant differences between the different groups.
Data exclusions	Tumors were excluded from the final analysis if volumes at randomization were not evaluable.
Replication	All experiments were performed at least twice, but more often three times.
Randomization	Tumor size was measured weekly by calipers, and after 15 days the mice were randomized to administration of the different drug combinations
Blinding	Evaluation of the immunohistochemistry staining was performed by a skilled breast pathologist in a blinded setup.

Reporting for specific materials, systems and methods

We require information from authors about some types of materials, experimental systems and methods used in many studies. Here, indicate whether each material, system or method listed is relevant to your study. If you are not sure if a list item applies to your research, read the appropriate section before selecting a response.

Materials & experimental systems

n/a	Involved in the study	
☐	☒	Antibodies
☐	☒	Eukaryotic cell lines
☒	☐	Palaeontology and archaeology
☐	☒	Animals and other organisms
☐	☒	Human research participants
☐	☒	Clinical data
☒	☐	Dual use research of concern

Methods

n/a	Involved in the study	
☒	☐	ChIP-seq
☒	☐	Flow cytometry
☒	☐	MRI-based neuroimaging

Antibodies

Antibodies used	Western Blot primary antibodies: anti-EGFR (#HPA001200, pEGFR (#3777), FGFR1 (#9740), pFGFR1 (#3476), anti-MET (#3148), anti-pMET (#3077), anti-AXL (#AB154), anti-Akt (#4685), anti-pAkt (#4060), anti-PRAS40 (#2691), anti-pPRAS40 (#2997), anti-PTEN (#9556), anti-EKR1/2 (#9102), anti-pERK1/2 (#4370), anti-mTOR (#2983), anti-FOXO3a (#2497), antip-FOXO3a (#2599), anti-S6 (#2317), anti-pS6 (#4858), anti-β-actin (#ab6276) ON at 4°C. All antibodies were purchased from Cell Signaling Technology except the anti-EGFR (Sigma Aldrich), anti-AXL (R&D Systems) and anti- β-actin (abcam).<br><br>Immunohistochemistry antibodies: pAkt (Cell Signaling Technology, #4060), phospho-PRAS40 (Cell Signaling Technology, #2997).
Validation	All antibodies are commercially available and have been validated by the company for Western blotting and/or immunohistochemistry.

Eukaryotic cell lines

Policy information about cell lines

Cell line source(s)	The parental HCC827 cells were purchased from the ATCC.<br>Parental PC9 cells were kindly provided by F. Hoffman-La Roche Ltd with the authorization of Dr. Mayumi Ono (Kyushu

	University, Fukuoka, Japan).<br>Parental 11-18 cells were kindly provided by Dr. Mayumi Ono.
Authentication	All cell lines were authenticated by Short Tandem Repeat (STR) DNA profiling.
Mycoplasma contamination	All cell lines were tested negative for mycoplasma contamination using Lonza MycoAlert Kit.
Commonly misidentified lines<br>(See ICLAC register)	N/A

Animals and other organisms

Policy information about studies involving animals; ARRIVE guidelines recommended for reporting animal research

Laboratory animals	The recipients were 7-week-old female CB-17 SCID mice, which were purchased from Taconic.
Wild animals	N/A
Field-collected samples	N/A
Ethics oversight	All animal experiments were approved by The Experimental Animal Committee of The Danish Ministry of Justice and were performed at the animal core facility at University of Southern Denmark.

Note that full information on the approval of the study protocol must also be provided in the manuscript.

Human research participants

Policy information about studies involving human research participants

Population characteristics	The patient cohort consisted of EGFR mutated lung cancer tissues from 40 patients collected from Dexeus Quirón University Hospital (Barcelona, Spain), Germans Trias i Pujol Hospital, (Badalona, Spain) and Fundación Santa Fe de Bogotá (Colombia). All tumors were EGFR mutated (29% Exon 19 deletion, 10 % L858R and 1 % other), all patients recieved EGFR-TKI (55 % Erlotinib, 42% Gefitinib and 3 % Afatinib), all patients were stage III or IV and 15 % had bone and 15 % brain metastasis.
Recruitment	The patient cohort consisted of EGFR-mutated NSCLC patients diagnosed in the Dexeus Quirón University Hospital (Barcelona, Spain), Germans Trias i Pujol Hospital, (Badalona, Spain) and Fundación Santa Fe de Bogotá (Colombia). The patients were treated with first line EGFR-TKIs (pre-treatment samples).
Ethics oversight	Studies were conducted in accordance with the Declaration of Helsinki and all relevant ethical regulations for work with human participants, under an approved protocol of the Institutional Review Board of Dexeus Quirón University Hospital and Germans Trias i Pujol Hospital. Samples were de-identified for patient confidentiality and informed written consent, also approved by the Institutional Review Boards, was obtained from all subjects.

Note that full information on the approval of the study protocol must also be provided in the manuscript.

Clinical data

Policy information about clinical studies

All manuscripts should comply with the ICMJE guidelines for publication of clinical research and a completed CONSORT checklist must be included with all submissions.

Clinical trial registration	N/A
Study protocol	N/A
Data collection	N/A
Outcomes	N/A
